# Population-based plasma lipidomics reveals developmental changes in metabolism and signatures of obesity risk: a mother-offspring cohort study

**DOI:** 10.1186/s12916-022-02432-y

**Published:** 2022-07-25

**Authors:** Sartaj Ahmad Mir, Li Chen, Satvika Burugupalli, Bo Burla, Shanshan Ji, Adam Alexander T. Smith, Kothandaraman Narasimhan, Adaikalavan Ramasamy, Karen Mei-Ling Tan, Kevin Huynh, Corey Giles, Ding Mei, Gerard Wong, Fabian Yap, Kok Hian Tan, Fiona Collier, Richard Saffery, Peter Vuillermin, Anne K. Bendt, David Burgner, Anne-Louise Ponsonby, Yung Seng Lee, Yap Seng Chong, Peter D. Gluckman, Johan G. Eriksson, Peter J. Meikle, Markus R. Wenk, Neerja Karnani

**Affiliations:** 1 Department of Biochemistry, Yong Loo Lin School of Medicine, National University of Singapore, Singapore, 117596 Singapore; 2 Singapore Lipidomics Incubator, Life Sciences Institute, National University of Singapore, Singapore, Singapore; 3 Singapore Institute for Clinical Sciences, Agency for Science, Technology and Research (A*STAR), Brenner Centre for Molecular Medicine, 30 Medical Drive, Singapore, 117609 Singapore; 4 Metabolomics Laboratory, Baker Heart and Diabetes Institute, 75 Commercial Road, Melbourne, VIC 3004 Australia; 5 KK Women’s and Children’s Hospital, Singapore, Singapore; 6 School of Medicine, Deakin University, Geelong, Australia; 7 Child Health Research Unit, Barwon Health, Geelong, Australia; 8 Murdoch Children’s Research Institute, University of Melbourne, Parkville, Australia; 9 The Florey Institute of Neuroscience and Mental Health, Parkville, Australia; 10 Department of Pediatrics, Yong Loo Lin School of Medicine, National University of Singapore, Singapore, Singapore; 11 Department of Obstetrics and Gynaecology, Yong Loo Lin School of Medicine, National University of Singapore, Singapore, Singapore; 12 Centre for Human Evolution, Adaptation and Disease, Liggins Institute, University of Auckland, Auckland, New Zealand; 13 Folkhalsan Research Center, Helsinki, Finland; 14 Department of General Practice and Primary Health Care, University of Helsinki, Helsinki, Finland; 15 DataHub Division, Bioinformatics Institute, Agency for Science, Technology and Research, Singapore, Singapore

**Keywords:** Lipidomics, Gestation, Intergenerational, Development, Lipids and fatty acids, Metabolomics, Maternal-fetal, Adiposity

## Abstract

**Background:**

Lipids play a vital role in health and disease, but changes to their circulating levels and the link with obesity remain poorly characterized in expecting mothers and their offspring in early childhood.

**Methods:**

LC-MS/MS-based quantitation of 480 lipid species was performed on 2491 plasma samples collected at 4 time points in the mother-offspring Asian cohort GUSTO (Growing Up in Singapore Towards healthy Outcomes). These 4 time points constituted samples collected from mothers at 26–28 weeks of gestation (*n*=752) and 4–5 years postpartum (*n*=650), and their offspring at birth (*n*=751) and 6 years of age (*n*=338). Linear regression models were used to identify the pregnancy and developmental age-specific variations in the plasma lipidomic profiles, and their association with obesity risk. An independent birth cohort (*n*=1935), the Barwon Infant Study (BIS), comprising mother-offspring dyads of Caucasian origin was used for validation.

**Results:**

Levels of 36% of the profiled lipids were significantly higher (absolute fold change > 1.5 and P_adj_ < 0.05) in antenatal maternal circulation as compared to the postnatal phase, with phosphatidylethanolamine levels changing the most. Compared to antenatal maternal lipids, cord blood showed lower concentrations of most lipid species (79%) except lysophospholipids and acylcarnitines. Changes in lipid concentrations from birth to 6 years of age were much higher in magnitude (log_2_FC=−2.10 to 6.25) than the changes observed between a 6-year-old child and an adult (postnatal mother) (log_2_FC=−0.68 to 1.18). Associations of cord blood lipidomic profiles with birth weight displayed distinct trends compared to the lipidomic profiles associated with child BMI at 6 years. Comparison of the results between the child and adult BMI identified similarities in association with consistent trends (*R*^2^=0.75). However, large number of lipids were associated with BMI in adults (67%) compared to the children (29%). Pre-pregnancy BMI was specifically associated with decrease in the levels of phospholipids, sphingomyelin, and several triacylglycerol species in pregnancy.

**Conclusions:**

In summary, our study provides a detailed landscape of the in utero lipid environment provided by the gestating mother to the growing fetus, and the magnitude of changes in plasma lipidomic profiles from birth to early childhood. We identified the effects of adiposity on the circulating lipid levels in pregnant and non-pregnant women as well as offspring at birth and at 6 years of age. Additionally, the pediatric vs maternal overlap of the circulating lipid phenotype of obesity risk provides intergenerational insights and early opportunities to track and intervene the onset of metabolic adversities.

**Clinical trial registration:**

This birth cohort is a prospective observational study, which was registered on 1 July 2010 under the identifier NCT01174875.

**Supplementary Information:**

The online version contains supplementary material available at 10.1186/s12916-022-02432-y.

## Background

Pregnancy is associated with changes to lipid metabolism as the gestation progresses [1]. These changes are essential to maintain a constant supply of nutrients to the gestating mother and the growing fetus [2]. Maternal dyslipidemia has been associated with pregnancy-related complications and perinatal outcomes such as preeclampsia, gestational diabetes, preterm birth, and macrosomia [3, 4]. There is also mounting evidence that in obese glucose-tolerant mothers, lipids contribute more strongly to excess fetal fat accretion than glucose [5, 6]. In utero nutrition plays a crucial role in fetal development. Sub-optimal in utero experience may not only influence the birth outcomes, but also alter the developmental programming to elevate the risk for chronic diseases later in life [7, 8]. However, molecular mechanisms, whereby antenatal nutrition affects fetal development, remain underexplored. Population-based studies have increased our understanding of metabolism related to maternal and child health outcomes [9–13]. However, there is a lack of population-level lipidomic studies to define longitudinal and intergenerational lipidomic profiles, as well as their association with adiposity. Here, we studied the changes to plasma lipidome of women during pregnancy (antenatal vs postnatal plasma), and their offspring during development (cord blood and 6-year-old child plasma) in the GUSTO (Growing Up in Singapore Towards healthy Outcomes) birth cohort. We also delineated the associations of plasma lipidomic profiles with pediatric and adult adiposity and studied their overlap. We validated these findings in an independent birth cohort, the Barwon Infant Study (BIS), comprising mother-offspring dyads of different ethnic origin (Caucasian), recruited using an unselected antenatal sampling frame in the southeast of Australia. This study provides a valuable resource for future developmental origins of health and disease (DOHaD) research.

## Methods

### Study participants and clinical characteristics

GUSTO (Growing Up in Singapore Towards healthy Outcomes) is a prospective mother–offspring cohort study in Singapore consisting of 1247 women recruited between June 2009 and September 2010 [14]. Study participants were of Chinese, Malay, or Indian ethnicity with both parents from the same ethnic background. Written informed consent was obtained at recruitment, and ethics approval for the study was granted by the Institute Review Board of KK Women’s and Children’s Hospital (KKH) and National University Hospital (NUH). This study was registered under ClinicalTrials.gov on 1 July 2010 under the identifier NCT01174875. The study was approved by Singhealth Centralised Institutional Review Board (CIRB) under CIRB Ref: 2018/2767. The study was conducted in accordance with the Declaration of Helsinki, and the lipidomics study was approved by the Institutional Review Board of National University of Singapore # H-17-055E. All subjects gave their informed consent for inclusion before they participated in this study.

Data on maternal age, ethnicity, education level, and self-reported pre-pregnancy weights were collected from the participants during recruitment. Pre-pregnancy body mass index (ppBMI) was calculated as pre-pregnancy weight divided by height squared. Antenatal BMI was calculated as weight measured at 26–28 weeks of gestation divided by height squared. Gestational weight gain (GWG) up to 26–28 weeks of pregnancy and total GWG were calculated by subtracting self-reported pre-pregnancy weights from weights measured at 26–28 weeks of gestation and weights measured at delivery, respectively. For offspring born to the women enrolled in this study, information on gestational age, birth weight, sex, and birth order were retrieved from birth delivery reports. At postnatal follow-up visit, age, weight, and height of mothers were collected at 4–5 years after delivery. Age, weight, and height of children were collected at 6-year-old. Postnatal maternal BMI and child BMI were calculated accordingly.

The Barwon Infant Study (BIS) is a major birth cohort study being conducted by the Child Health Research Unit (CHRU) at Barwon Health in collaboration with the Murdoch Children’s Research Institute (MCRI) and Deakin University [15]. The association studies with birth weight and pre-pregnancy BMI were used for replication of findings in GUSTO cohort.

### Sample selection, preparation and experimental design

A total of 2519 plasma samples collected after overnight fasting from GUSTO cohort were selected for this lipidomics study. These consisted of antenatal maternal samples (*n*=763) collected at 26–28 weeks of pregnancy, cord blood samples (*n*=767) collected at delivery, postnatal maternal (*n*=651) collected at 4–5 years after delivery, and child samples (*n*=338) collected at 6 years of age. All plasma samples were prepared in 10 aliquots for lipid extraction. A pooled quality control was prepared from a subset of the representative samples from the study. This pooled QC was then used to prepare a batch QCs (BQC) and a technical QCs (TQC). These QCs serve well-defined roles of monitoring and correcting for biases introduced during sample preparation, LC-MS/MS-based measurements as well as for determining signal-to-noise ratios, coefficient of variation of individual lipid species, linearity and stability of the analytical platform. A stratified randomization strategy was used to allocate the samples into seven batches. Paired samples were measured in the same batch. At the same time, blanks, process blanks (PBLK), and aliquots of reference plasma samples from NIST-1950 were interspersed in between the samples. QCs and blanks were processed and analyzed along with the study samples within each batch.

### Lipid extraction and LC-MS/MS analysis

The samples were extracted according to the stratified randomization template and 438 samples (study samples as well as QCs) were extracted in one batch with a total of seven batches for the current study. Lipid extraction was carried out using butanol: methanol (extraction solvent) in a ratio of 1:1 containing 10 mM ammonium formate and deuterated or non-physiological internal standards as described previously [16]. A total of 100 μL of extraction solvent was added to each sample, vortexed for 10 s followed by sonication for 60 min with temperature maintained at 18–22 °C. The samples were centrifuged at speed of 13,000 rpm for 10 min. The supernatant (80 μL) was collected in mass spectrometry compatible vials and stored at −80 °C for LC-MS/MS. These lipid extracts were analyzed by using Agilent 6490 QQQ mass spectrometer interfaced with an Agilent 1290 series HPLC system. Lipids were separated on a ZORBAX RRHD UHPLC C_18_ column (2.1×100 mm 1.8 mm, Agilent Technologies) with the thermostat set at 45 °C. Targeted LC-MS/MS was performed in ESI positive ion mode with dynamic multiple reaction monitoring (dMRM). Mass spectrometry settings and MRM transitions for each lipid class, subclass, and individual species were kept as described previously [17, 18]. The following mass spectrometer parameters were used: gas temperature, 150 °C, gas flow rate 17L/min, nebulizer 20psi, sheath gas temperature 200 °C, capillary voltage 3500V, and sheath gas flow 10L/min. Isolation widths were set to unit resolution for both Q1 and Q3. QC samples were analyzed along with the samples to monitor sample extraction efficiency as well as LC-MS performance and were subsequently used to do batch corrections.

### Pre-processing of data

Peak integration was carried out in MassHunter Quantitative software (Agilent Technologies) to select area of each individual lipid species. Manual inspection was carried out to ensure that correct peaks were picked at specific retention time. Peak areas along with retention times were exported as .csv for further analysis. These data were filtered by using a cut-off for signal-to-noise ratio (S/N>10) and linear response (*R*^2^>0.8) of each lipid species. Peak areas of lipid species were normalized to their class specific internal standard as described previously [17, 18]. Batch QCs was used to correct signal drifts across the batches based on LOESS [19] regression method (locally polynomial regression fitting, span=0.75, R version 3.5.1). Following that, lipid species were dropped if quality control coefficient of variation was greater than 20%. These stringent quality control steps enabled us to measure lipid species with precision (average CV = 9.8%). The approach applied here provides relative quantitation as the use of isotope-labeled internal standards required for absolute quantitation of each lipid species under investigation is not feasible. Finally, a total of 480 lipid species representing 25 lipid classes were used for the downstream data analysis. In addition, we removed 27 outlier samples based on principal component analysis (Additional file 1: Fig. S1) and one cord blood sample without sex information which resulted in 2491 samples for further downstream analysis.

### Statistical analysis

All lipidomics data were log_10_ transformed for the downstream analyses. The unsupervised principal component analysis of lipid species data from all the samples was applied to estimate overall differences between the four sample groups (antenatal mother (*n*=752), cord blood (*n*=751), postnatal mother (*n*=650), and child (*n*=338)). Postnatal maternal plasma lipidomic profiles (average age = 36 years and not pregnant) was used as a proxy for adult samples for comparison with child lipidomic profiles. As the sample size of each group is different, both cross-sectional and paired analyses were applied to four comparative lipidomics studies (postnatal vs. antenatal, cord blood vs. antenatal, child vs. cord blood and adult vs. child). Cross-sectional analysis used all the available samples in each group. Linear regression models were applied using lipid levels as outcomes and groups as predictors. Potential confounders were examined by checking their associations with the top ten principal components of the specific lipidomic dataset in each comparison study and then the selected confounders were adjusted in the regression models accordingly. In the comparison study of antenatal and postnatal lipidomic profiles, ethnicity, maternal age, maternal education level, and pre-pregnancy BMI were adjusted. For the comparison between antenatal and cord blood lipidomic profiles, ethnicity, maternal age, maternal education level, pre-pregnancy BMI, infant sex, gestational age, and birth weight were adjusted. Sex, ethnicity, maternal education level, gestational age, and birth weight were adjusted in the comparison study of cord blood and child lipidomic profiles. For the comparison between child and adult (postnatal mother) lipidomic profiles, sex, ethnicity, BMI, and maternal education level were adjusted. In addition, paired *t*-test was also applied to compare group difference using paired samples with a much smaller sample size (Additional file 1: Fig. S2; postnatal vs. antenatal: 381 pairs; cord blood vs. antenatal: 445 pairs; child vs. cord blood: 237 pairs and adult vs. child: 272 pairs) and the results were compared to the linear regression results. The regression coefficients (*β*) were converted to log_2_ fold change (FC=10^β^). The adjusted *p*-values (*P*_adj_) were calculated by the Benjamini-Hochberg (BH) method. The lipid species with |FC|>1.5 and *P*_adj_<0.05 were considered significant in the above comparative studies.

Similarly, association of lipids with BMI or birth weight was studied in the four sample groups using linear regression. Both ppBMI and antenatal BMI were studied in antenatal lipidome. ppBMI was studied after accounting for the effects of maternal ethnicity, education, age, and gestational weight gain at 26–28 weeks of pregnancy. Antenatal BMI was studied after the adjustment of maternal ethnicity, education, and age. Child BMI and adult (postnatal) BMI were examined in child lipidome and postnatal mother lipidome, respectively. Child BMI was studied after the adjustment of child sex, ethnicity, and maternal education, while adult BMI was studied after accounting for the effects of ethnicity, maternal education, and age. As birth weight (BW) is widely used as a risk factor for overweight/obesity in pediatrics [20], BW was investigated in cord blood lipidome after the adjustment of sex, ethnicity, maternal age, maternal education, pre-pregnancy BMI, total gestational weight gain, gestational age, and birth order. The regression coefficients (*β*) were converted to % change in lipid concentration per unit BMI (ppBMI, child BMI and adult BMI), or % change in lipid concentration per 100 grams of BW (% change = (10^β^− 1) × 100). The findings from GUSTO study were replicated in the Barwon Infant Study (BIS) cohort. As described before, linear regression was applied to investigate the association between ppBMI and antenatal lipidomic profiles (pregnant state), and the association between BW and lipidomic profiles of cord blood plasma. The ppBMI and BW results were compared between GUSTO and BIS. Scatter plots of effect sizes in two cohorts were used to highlight the linear trend and the overlapping lipid species in the two cohorts. The overlapping percentage of associated lipid species was calculated in each study. Finally, meta-analysis was performed for ppBMI and BW studies in the two cohorts using inverse variance-weighted average method [21]. Meta-analysis results were presented in forest plot and volcano plot. The lipid species with the adjusted *p*-value (*P*_adj_) < 0.05 were considered to be significantly associated with BMI. All the association analyses were implemented in MATLAB R2019b.

## Results

### Overview of plasma lipidomics

**Fig. 1:**
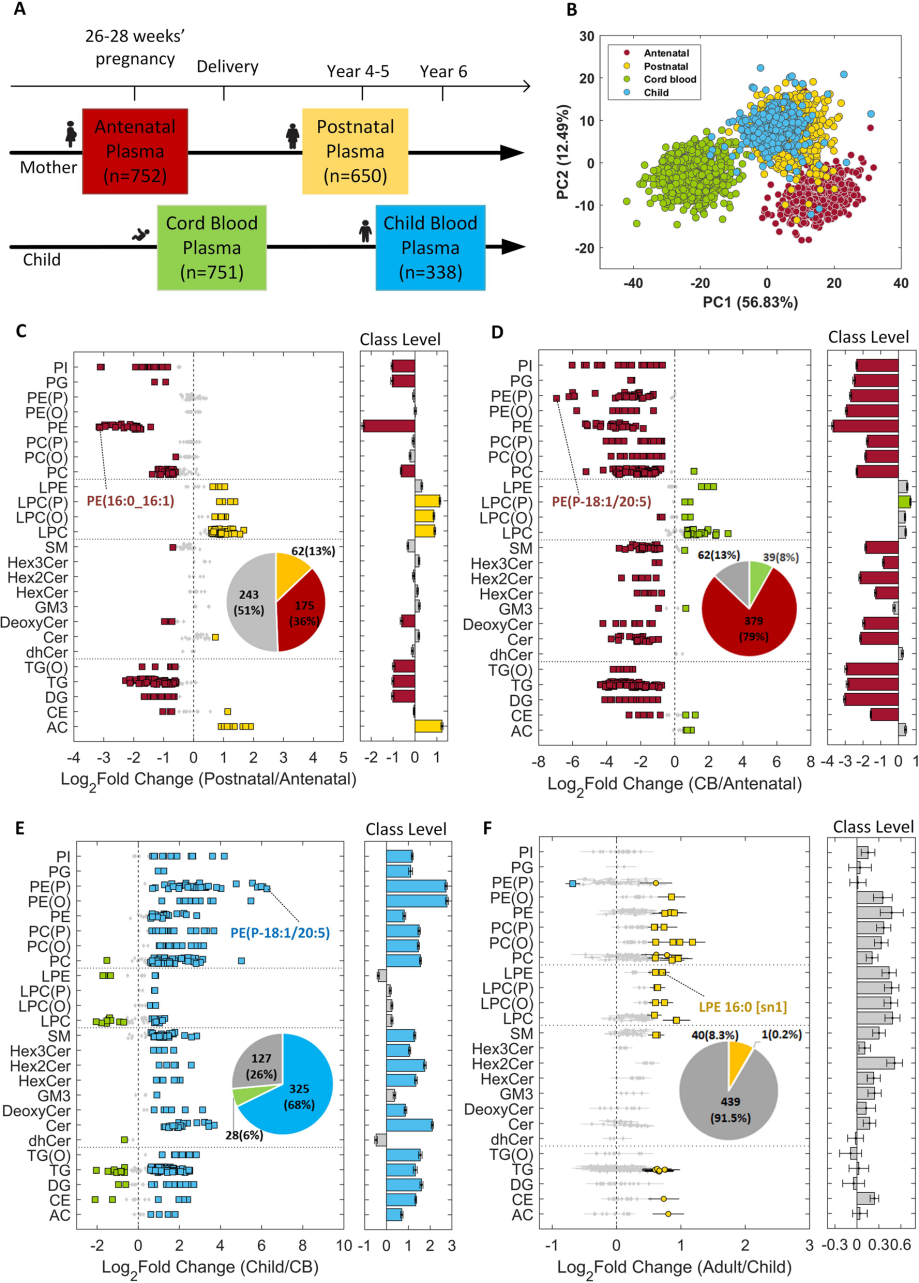
Temporal and developmental alterations to the circulatory lipids in the GUSTO cohort: **A** Antenatal and postnatal plasma collection time points for mother-offspring dyads in GUSTO cohort. **B** PCA plot of lipidomics data (*n*=2491). **C** Postnatal vs. antenatal changes in maternal lipidomic profiles. **D** Comparison of maternal antenatal plasma with cord blood (CB) lipidomic profiles. **E** Changes in child lipidomic profiles between birth and 6 years of age. **F** Comparison of pediatric (6-year-old child) and adult (postnatal mothers) plasma lipidomes. The most significant lipid species based on adjusted *p*-values are labeled in c-f. Effect size is shown as log_2_ of fold change. Error bars indicate 95% confidence interval. Diamond—*P*_adj_ ≥ 0.05 or |FC|≤1.5 (gray), circle—*P*_adj_ <0.05 and |FC|>1.5, and square—*P*_adj_ <1.00E−10 and |FC|>1.5

Demographic and anthropometric measures of the study participants are provided in Table 1. A total of 480 lipid species representing 25 lipid classes were profiled across 2491 maternal and offspring plasma samples, covering 4-time points (antenatal (*n*=752) and postnatal (*n*=650) for mothers, and at birth (*n*=751) and 6 years of age (*n*=338) for their offspring) as illustrated in Fig. 1A. The combined unsupervised principal component analysis (PCA) identified two major components, with the first explaining 56.83% of the variance, and the second explaining 12.49% of the variance in the total study population. The PCA plot showed distinct clusters of mothers during gestation and post-pregnancy (antenatal vs postnatal), and also of their offspring at birth (cord blood) and 6 years of age (Fig. 1B). The overlap in the clusters of postnatal mothers and their 6-year-old offspring indicated the emerging similarities between the pediatric and adult lipidomic profiles.

**Table 1:** Demographic and anthropometric characteristics of the study participants

Antenatal Mothers (26-28 weeks’ pregnancy) ***n***=752			Postnatal Mothers (4–5 years after delivery) ***n***=650		
Variable	*n*	Mean (±STD)	Variable	*n*	Mean (±STD)
Ethnicity			Ethnicity		
Chinese	400	-	Chinese	375	-
Malay	198	-	Malay	160	-
Indian	154	-	Indian	115	-
Maternal education level			Maternal education level		
Secondary and below	217	-	Secondary and below	194	-
Post-secondary	259	-	Post-secondary	223	-
University	270	-	University	228	-
Maternal age (years)	752	30.68 (±5.11)	Maternal age (years)	585	35.68 (±5.15)
Pre-pregnancy body mass index (BMI, kg/m^2^)	699	22.84 (±4.33)	Pre-pregnancy BMI (kg/m^2^)	596	22.65 (±4.34)
Antenatal BMI (kg/m^2^)	739	26.31 (±4.36)	BMI (kg/m^2^)	594	24.55 (±5.23)
Gestational weight gain (kg)	700	8.62 (±4.31)			
**Newborns**			**Children (6-year-old)**		
***n*=751**			***n*=338**		
Variable	*n*	Mean (±STD)	Variable	*n*	Mean (±STD)
Ethnicity			Ethnicity		
Chinese	368	-	Chinese	195	-
Malay	225	-	Malay	89	-
Indian	158	-	Indian	54	-
Sex			Sex		
Male	401	-	Male	185	-
Female	350	-	Female	153	-
Maternal education level			Maternal education level		
Secondary and below	251	-	Secondary and below	100	-
Post-secondary	284	-	Post-secondary	120	-
University	205	-	University	116	-
Gestational age (weeks)	751	38.74 (±1.37)	Gestational age (weeks)	338	38.79 (±1.63)
Birth weight (kg)	751	3.10 (±0.44)	Birth weight (kg)	338	3.10 (±0.46)
Maternal age (years)	751	30.20 (±5.20)	Child age (years)	330	6.06 (±0.09)
Pre-pregnancy BMI (kg/m^2^)	679	22.97 (±4.61)	Child BMI (kg/m^2^)	330	15.62 (±2.33)
Total gestational weight gain (kg)	658	13.50 (±5.76)			
Birth order					
1	330	-			
>1	421	-

### Comparison of mother-offspring lipidomes

Subsequent analyses characterize and compare the lipidomic profiles of antenatal and postnatal mothers and their offspring using linear regression models and paired *t*-test (see the “Methods” section and Additional file 1: Table S1A-C). As both results were similar (Additional file 1: Fig. S3), only the linear regression results are discussed in this section.

### Alterations to maternal plasma lipidome during pregnancy

Alterations to the plasma lipids during pregnancy (antenatal vs postnatal) were assessed using linear regression models. Thirty-six percent of the profiled plasma lipids were substantially elevated in pregnancy (Fig. 1C). These primarily included phospholipids, such as phosphatidylcholine (PC), phosphatidylethanolamine (PE), phosphatidylinositol (PI), and phosphatidylglycerol (PG), with PE showing the most significant increase. PE (16:0_16:1) showed the greatest difference in circulation (log_2_FC=−3.16 and *P*_adj_<1.00E−30). Docosahexaenoic acid (DHA) containing lipid species like PE (16:0_22:6) and PE (18:0_22:6) and arachidonic acid (ARA) containing PE (16:0_20:4) and PE (18:0_20:4) were also elevated (Additional file 1: Table S1B). The total PC to PE ratio was lower in pregnancy (Additional file 1: Fig. S4A). Additionally, ratios of DHA-PC to their corresponding DHA-PE species decreased by twofold or more in antenatal vs postnatal circulation (Additional file 1: Fig. S4B), suggesting DHA-PE species in maternal plasma as an important source for maternal-fetal transfer of DHA. Acylcarnitine (AC) species showed a general decrease in pregnancy. Lysophosphatidylcholine (LPC), lysophosphatidylethanolamine (LPE), alkyllysophosphatidylcholine (LPC (O)), and alkenyllysophosphatidylcholine (LPC (P)) were also low in pregnancy. Ether-linked phospholipids including alkylphosphatidylcholine (PC(O)), alkenylphosphatidylcholine (PC(P)), alkenylphosphatidylethanolamine (PE(P)), and alkylphosphatidylethanolamine (PE(O)) did not show a significant change in antenatal compared to the postnatal plasma except PC(O-16:0/22:6) that was increased in pregnancy. Among the sphingolipids, only deoxy-ceramide lipids and SM (40:3) were significantly increased whereas Cer(d19:1/24:0) was decreased in pregnancy. Neutral lipids including triacylglycerol (TG), alkyl-diacylglycerol (TG(O)), and diacylglycerol (DG) were elevated in pregnancy. Cholesteryl esters (CE) species showed a divergent trend with a decrease in the levels of CE (20:5) whereas CE (16:1), CE (24:5), and CE (24:6) were increased in pregnancy (Additional file 1: Table S1B).

### Comparison of antenatal maternal and fetal cord blood lipidomes

To develop further insights into the maternal-fetal transfer of lipids, antenatal lipidomic profiles of mothers were compared to that of the offspring cord blood. Circulating levels of 79% of the profiled lipids were substantially higher in maternal plasma than in cord blood (Fig. 1D. Concentration of all glycerophospholipid classes were lower in cord blood, while lysoglycerophospholipid classes were mostly higher with the exception of LPC(O-24:0) and LPC(O-24:1). Levels of PUFA (ARA, dihomo-γ-linolenic acid (DGLA), and DHA) containing lipid species were elevated in cord blood. The highest fold change was observed in ARA containing lipids such as LPC (20:4) and LPE (20:4) (log_2_FC > 2). Overall, PUFA containing LPC represented 27% of the total LPCs in cord blood, compared to 13% in antenatal maternal blood (Additional file 1: Fig. S4C), indicating a higher fetal demand for these lipids to support its growth and development. Sphingolipid lipid species were mostly low in cord blood. Acylcarnitine species were increased in cord blood. In summary, there was a generalized increase in acylcarnitines and lysophopholipids, but most of the complex lipids were present at lower levels in cord blood.

### Alterations to plasma lipids in early childhood, and a comparison with the adult lipidome

To interrogate the changes in lipidomic profiles in early childhood, we compared the plasma lipidomic profiles of the child at birth with that at 6 years of age. A marked change (log_2_FC=−2.10 to 6.25) in the offspring lipidome was observed 6 years post-birth, with 68% of the lipids showing a relatively higher abundance, and 6% showing a lower abundance in circulation (Fig. 1E). The lipid species that were present at higher concentrations in cord blood included PUFA containing lysoglycerophospholipid species such as LPC(20:4), LPC(22:6), LPE(20:4), and LPE (22:6) as well several diacylglycerol and triacylglycerol species such as DG (16:0_22:6), TG (50:4) [NL-20:4], TG (56:8) [NL-22:6], and TG (58:8) [NL-22:6].

We also compared the 6-year-old pediatric and adult (postnatal mother: mean age 36 years) plasma lipidome using linear regression models. There was a substantial overlap between the pediatric and adult plasma lipidomic profiles, and the differences were smaller in magnitude than those observed in pregnancy, and at birth. Levels of 40 (8%) lipid species in adults, and one lipid (PE(P-18:0/22:4)) in children, were higher (Fig. 1F). The lipids with significantly increased levels in adults were phospholipid and triacylglycerol species that predominantly contained eicosapentaenoic acid (EPA) and docosahexaenoic acid (DHA). The PE-plasmalogen (PE(P)) species that were present at higher concentration in adults contained EPA whereas those present at higher concentration in child plasma contained adrenic acid (AdA) (Additional file 1: Table S1B).

### Plasma lipids associated with mother and child adiposity, and the intergenerational link

**Fig. 2:**
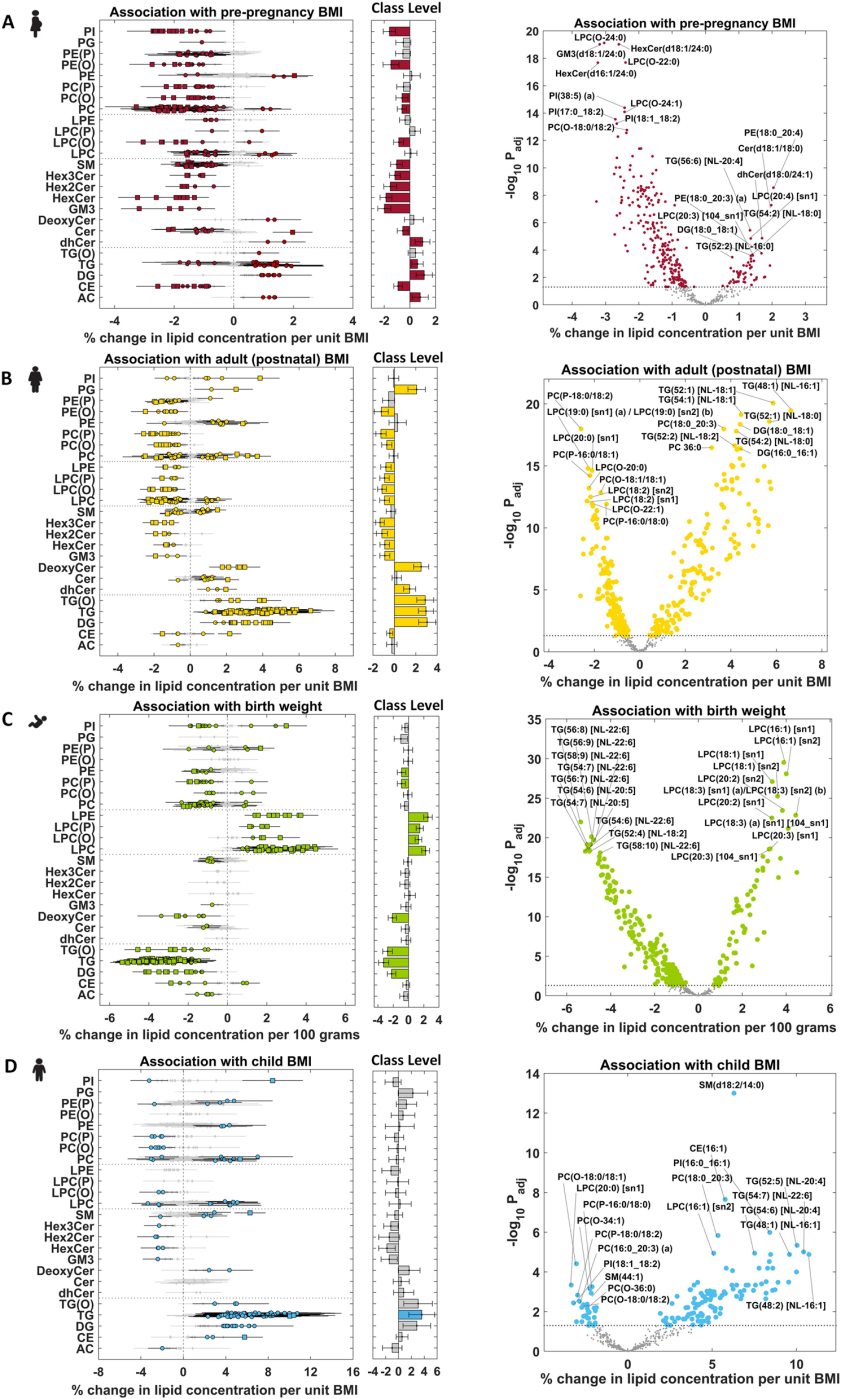
Association of maternal and child adiposity with plasma lipidomic profiles: **A** Association of pre-pregnancy BMI with antenatal plasma lipidome (pregnant state). **B** Association of maternal BMI with postnatal plasma lipidome (non-pregnant state). **C** Association of birth weight (BW) with cord blood plasma lipidome. **D** Association of child BMI with plasma lipidome at 6 years of age. The top 20 lipid species with the directionality of association (10 positive and 10 negative) with adiposity measures in each study group are shown in volcano plots. The horizontal dotted line indicates *P*_adj_=0.05 in each volcano plot. Effect sizes are shown as % change in lipid concentration per unit change in BMI, or per 100 g change in birth weight. Error bars indicate 95% confidence interval. Diamond—*P*_adj_ ≥ 0.05 (gray), circle—*P*_adj_ <0.05, and square—*P*_adj_ <1.00E−5 in forest plots

**Fig. 3:**
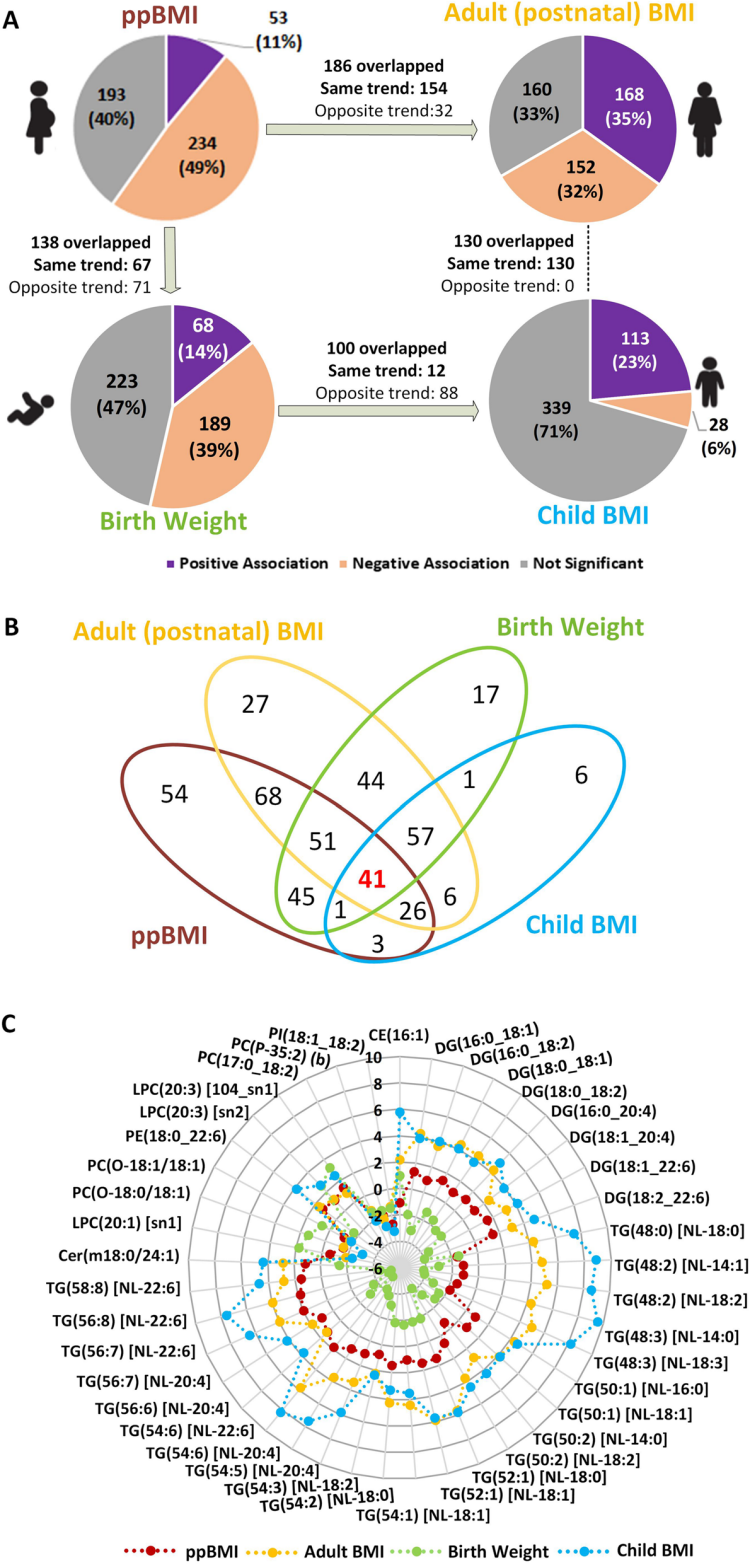
Comparison of plasma lipidomic profiles associated with mother and child adiposity: **A** Pie chart comparing the percent overlap and directionality of association in the four studies. **B** Venn-diagram of lipids species that passed significance in the four association studies. **C** Effect sizes of 41 lipid species that overlapped between the four studies

Adiposity is often associated with the changes in plasma lipidomic profiles, though less is known about these changes in physiological states such as pregnancy and during child development. To investigate this at depth, we studied the plasma lipids associated with mother and child adiposity, and the intergenerational similarities between the two. Four different regression models were implemented to identify the lipids associated with adiposity in each study group. Forest plots and bar plots were generated to visualize % change in the concentration of lipid species and lipid classes, respectively (Fig. 2A–D; detailed in Additional file 1: Table S2). The top 20 lipid species with the directionality of association (10 positive and 10 negative) with adiposity measures in each study group are shown through volcano plots, and the overlap between the subject groups is provided in Fig. 3 and Additional file 1: Table S3. Scatter plots comparing the effect sizes and adjusted *p*-values in the four association studies are shown in Additional file 1: Fig. S5.

### Maternal adiposity and plasma lipids

As maternal weight undergoes changes during pregnancy, antenatal BMI was higher than pre-pregnancy BMI (ppBMI) in average but with strong correlation (*R*^2^=0.85, Additional File 1: Fig. S6 and Table 1). The association results of both BMIs with antenatal lipidomic profiles were also highly correlated (*R*^2^=0.99, Additional File 1: Fig. S7 and Table S2B-C). As ppBMI is the more commonly used obesity measure in the field to study pregnancy-related outcomes, only the ppBMI results are discussed in this section. All 287 lipid species (60% of total) showed significant associations with ppBMI, of which 234 had positive and 53 had negative associations (Figs. 2A and 3A). Most of the glycerophospholipids including ether-linked species showed a negative association with ppBMI, except for PUFA containing phosphatidylcholine species including PC(18:0_20:3) and PC(18:0_20:4), and phosphatidylethanolamine species such as PE(16:0_20:4), PE(18:0_20:4) and PE(18:0_22:6). Lysophosphatidylcholine species were negatively associated with maternal BMI except omega-6 fatty acid containing species including LPC(20:3) and LPC(20:4). Most of the triacylglycerol species were positively associated with ppBMI except those containing long chain fatty acids such as FA 14:0, FA 14:1, FA 16:1, and FA 18:3 that showed a negative association. Sphingolipids, including sphingomyelin, ceramide, and mono-, di-, and trihexosylceramide as well as GM_3_-gangliosides, were negatively associated whereas deoxy-ceramide and dihydroceramide were positively associated with ppBMI. Acylcarnitine were positively and cholesteryl esters were negatively associated with ppBMI. Similar analysis post-pregnancy identified 320 lipid species (67%), with 168 positively associated and 152 negatively associated with postnatal BMI (Figs. 2B and 3A). Glycerophospholipids showed divergent trends in associations; lipid species containing polyunsaturated omega-6 fatty acids including LPC(20:3), PC(18:0_20:3), PC(18:1_20:3), PE(18:0_20:3), PI(16:0_20:4), and PE(18:0_22:4) were positively associated with BMI. Upon comparison, 186 BMI-associated lipid species overlapped between the pregnant and non-pregnant states (Additional file 1: Table S3A). Among these, 154 lipid species (83%) showed consistent trends (Fig. 3A and Additional file 1: Fig. S8A), while the remaining 32 (17%) had opposite trends. The lipid species that showed consistent trends belonged to ether-linked phospholipid classes including PC(O), PC(P), PE(O), and PE(P) as well as LPC(O) and LPC(P) that were negatively associated. Similarly, mono-, di-, and trihexosylceramide and GM_3_-gangliosides were negatively associated whereas deoxy-ceramide and dihydroceramide showed positive association. Scatter plot comparing % change in the two studies showed a positive trend with *R*^2^=0.28 (Additional file 1: Fig. S5A-B). Top 20 lipid species associated with BMI in pregnant and non-pregnant states are indicated in the volcano plots in Fig. 2A, B, respectively.

### Child adiposity and plasma lipids

We next examined the relationship between plasma lipids and child adiposity at birth and 6 years of age. Since the inter-individual variation in BMI at birth is subtle and a less reliable measure, we instead used birth weight (BW) as a surrogate measure for adiposity. Association analysis with cord blood plasma lipids identified 257 (53%) lipid species to be significantly associated with BW (Figs. 2C and 3A). Among these, 68 had positive and 189 had a negative association. LPC, LPC(O), LPC(P), and LPE were positively associated, while TG, TG(O), and DG were negatively associated with BW. TG species containing DHA showed strong negative association with BW, e.g.; (TG(56:8) [NL-22:6]: −5.36% change per 100 g increase and *P*_adj_=1.02E−22) whereas several LPC and LPE species containing FA 16:1 and FA 18:1 as well as omega-6 PUFA such as LPC(20:3), LPC(20:4), and LPE(20:4) were positively associated with BW. LPC(16:1) showed a strong positive association, with 3.89% increase in LPC(16:1) (sn1) (*P*_adj_=2.92E−30) and a 4.00% increase in LPC(16:1) (sn2) (*P*_adj_=8.10E−29) per 100 g of BW.

To further compare the lipids linked with adiposity at birth and in early childhood, associations between lipidomic profiles of 6-year-old children and their BMI were compared with BW-associated lipids. Plasma levels of 141 (29%, 113 high and 28 low) lipid species were substantially altered in children with higher BMI at age 6 (Figs. 2D and 3A). The number of lipid species associated with BMI in children was lower across the lipid classes except that of TG species that showed positive associations. Glycerophospholipid classes showed divergent associations; several PUFA containing species PC (16:1_20:4), PC (18:0_20:3) and PE (18:0_20:3) as well as plasmalogen species PE(P-16:0/20:3), PE(P-16:0/20:5) and PE(P-18:0/20:3) were positively associated with BMI. Although a few sphingolipid species were associated with BMI in children, SM(d18:2/14:0) showed strong positive association (6.29% change per unit BMI and *P*_adj_=9.93E−14). Furthermore, sex-stratified analysis identified several differences in association of lipidomic profiles with BW (Additional file 1: Fig. S9A-B). A higher number of lipid species were associated with BW in males (48%) as compared to females (31%). These differences were mainly driven by phospholipids including ether-linked phospholipids, acylcarnitines, diacylglycerol, and triacylglycerol species that showed a stronger association in males (Additional file 1: Table S4). Similarly, there were differences in associations of lipidomic profiles with BMI between male and female offspring at 6 years (Additional file 1: Fig. S9C-D). Comparison of BW- and 6-year BMI-associated lipids showed very different trends for most lipid classes. Around 88% of overlapping lipid species showed opposite trends and only 12 had a consistent trend (Fig. 3A, Additional file 1: Fig. S8C and Table S3C). Scatter plot of the effect sizes in two studies showed a negative trend with *R*^2^=0.31 (Additional file 1: Fig. S5E). These findings indicate that the cord blood lipidome was associated with BW; however, these associations become weaker in early childhood, potentially due to postnatal exposures (Additional file 1: Fig. S5F).

### Intergenerational similarities in lipids associated with obesity risk

There is compelling evidence indicating that offspring of obese/overweight mothers bear a higher risk of having obesogenic growth trajectories and developing metabolic dysfunction/disease during their life-course [22]. Aligned with these reports, we found a significant correlation between the mothers and their offspring BMI. Having identified the plasma lipids linked with mother and child obesity, we leveraged these findings to explore the intergenerational similarities through lipid signatures. Around 138 common lipid species were associated with ppBMI and BW, with 67 showing consistent trends (Fig. 3A, Additional file 1: Fig. S8B and Table S3B). Comparison of child BMI-lipid associations with postnatal maternal-BMI lipids identified a 92% (130 of 141) overlap between the two, with consistent trends (Fig. 3A and Additional file 1: Table S3D). Scatter plot of effect sizes confirmed a positive trend and strong correlation (*R*^2^=0.75) between the two (Additional file 1: Fig. S5G-H). This observation not only confirmed the intergenerational link of obesity risk through lipidomic profiles but also indicated an early onset in childhood. To study the longitudinal and intergenerational obesogenic lipid signatures, we compared the adiposity-associated lipids in all the 4 study groups (Fig. 3A) and identified 41 overlapping lipid species (Fig. 3B, C and Additional file 1: Table S3E). The directionality of association for most of the lipids showed opposite trend between BW and the other three studies, indicating that some lipids associated with adiposity in early childhood and adulthood may be beneficial for optimal fetal growth.

### Replication of obesity-linked lipid species in an ethnically diverse birth cohort

**Fig. 4:**
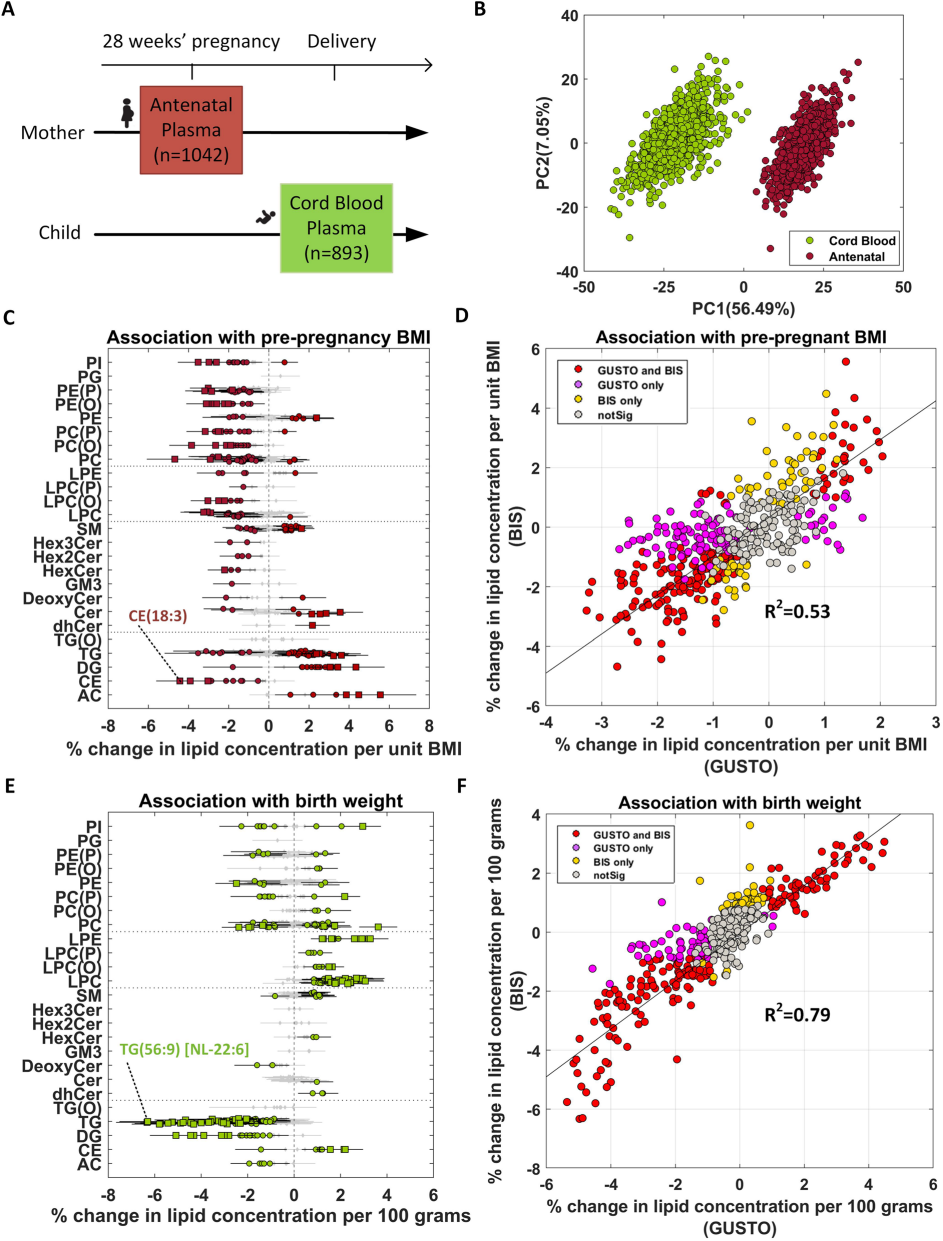
Replication of GUSTO identified ppBMI and BW lipid signatures in Barwon Infant Study (BIS). **A** Sample collection at two time points. **B** PCA plot of lipidomics data (*n*=1935). **C** Association of pre-pregnancy BMI (ppBMI) with antenatal plasma lipidome. **D** Scatter plot of effect sizes in GUSTO and BIS for ppBMI study. **E** Association of birth weight (BW) with cord blood plasma lipidome. The most significant lipid species based on adjusted *p*-values are labeled in **C** and **E**. Effect sizes are shown as % change in lipid concentration per unit change in BMI, or per 100 g change in birth weight. Error bars indicate 95% confidence interval. Diamond—*P*_adj_ ≥ 0.05 (gray), circle—*P*_adj_ <0.05, and square—*P*_adj_ <1.00E−5. **F** Scatter plot of effect sizes in GUSTO and BIS for BW study. Red—significant in both cohorts, purple—only significant in GUSTO, yellow—only significant in BIS, and gray—not significant in both cohorts

To investigate if the obesity-linked lipids were population-specific or were independent of the ethnic effects and geographical location, we compared the findings from Asian-centric GUSTO cohort in Singapore with Caucasian-centric Barwon Infant Study (BIS) from Australia (Fig. 4A). Due to the unavailability of the postnatal maternal and 6-year child plasma samples, we restricted the replication analysis to antenatal maternal blood (*n*=1042) and cord blood lipids (*n*=893). Similar to GUSTO, PCA plot of BIS data also revealed distinct clusters for antenatal maternal and offspring cord blood samples (Fig. 4B). Out of 480 lipid species profiled in GUSTO, 464 lipids were also profiled in BIS and used for subsequent association analysis with maternal ppBMI and offspring BW (Additional file 1: Table S5).

Two hundred thirty-six lipid species showed a significant association with maternal BMI, of which 80 had positive and 156 had a negative association (Fig. 4C). One hundred seventy of 236 (72%) lipid species overlapped with BMI-lipids identified in GUSTO mothers, and 80% of 464 lipid species had the same directionality of effect sizes in the two cohorts. Comparison of effect sizes in both the cohorts showed a significant positive trend with *R*^2^=0.53, though the effect sizes were generally larger in BIS subjects (Fig. 4D). This could be due to a higher number of obese women or higher degree of metabolic dysregulation in the BIS cohort.

BW-lipid analysis identified 224 affected lipid species in BIS cord blood samples, of which 108 had positive and 116 had a negative association (Fig. 4E). 176 of 224 (79%) lipids overlapped between the two cohorts. 81% of 464 lipid species showed the same directionality of effect sizes in the two cohorts, and their effect sizes were highly correlated (*R*^2^=0.79) (Fig. 4F). Lipid species with discordant trends in the two cohorts can potentially be population-specific (Asian vs Australian). For example, SM showed a negative association with ppBMI and BW in GUSTO, while it exhibited divergent trend with ppBMI and positive association with BW in BIS.

Meta-analysis in the two cohorts identified 331 BMI- and 295 BW-associated lipid species, respectively (Additional file 1: Table S5, Fig. S10A and Fig. S10C). Top significant lipid species from the meta-analysis results are labeled in volcano plots (Additional file 1: Fig. S10B and Fig. S10D). One hundred nineteen of 138 lipid species identified in ppBMI vs BW comparison in GUSTO (Fig. 3A and Additional file 1: Table S3B) were also significant in the meta-analysis, and these also included 35 of 41 lipid species identified in the 4-group intergenerational obesity risk analysis in GUSTO (Fig. 3C).

## Discussion

Cross-sectional and prospective assessment of mother and child plasma lipidomic profiles in our study provided novel insights into the antenatal and postnatal lipid species in circulation. It also identified similarities between child and adult lipidomic profiles, and the intergenerational overlap in the plasma lipids associated with adiposity. Characterization of plasma lipidomic profiles not only confirmed the previous reports on the substantial changes in the lipid levels in pregnancy [1, 23, 24], but also provided an in-depth profile of lipidomic changes covering 480 lipid species from 25 lipid classes. Around 36% of the profiled lipids increased in circulation in pregnancy, with phosphatidylethanolamine levels changing the most. Compared to the gestating mother, cord blood showed lower concentration of most lipids (79%), and higher concentration of lysophospholipids, indicating the specific developmental needs of the growing fetus. A substantial number of proteins involved in lipid metabolism are expressed in fetal tissues [25], but the fetus itself is unable to independently support and maintain the plasma lipid pools. Hence the maternal-fetal transport of lipids through placenta supports these needs of the growing fetus. A decrease in the levels of lysophospholipids but elevated levels of phospholipids in maternal circulation are suggestive of the decrease in the activity of phospholipase A2 (PLA2) and lecithin-cholesterol acyltransferase (LCAT) [26] enzymes in mid to late gestation to facilitate the placental uptake of intact phospholipids for further metabolism and transport of lyso-PL to fetus. Among the phospholipids we also observed a decrease in the PC to PE ratio, especially in the phospholipids containing PUFA, such as DHA, indicating the preference for accumulation of DHA-PE in maternal circulation. Correspondingly, we also observed higher levels of phospholipid products including DHA-LPE and DHA-LPC in cord blood. A probe into the percentage contribution of polyunsaturated fatty acid composition of lysophosphatidylcholine in cord blood plasma revealed a higher percentage of PUFA (27%) than maternal (antenatal and postnatal) and child 6-year plasma (Additional file 1: Fig. S4). Previous stable isotope labeling experiments have shown that majority of the labeled fatty acids were incorporated into phospholipids with a significant enrichment of labeled DHA in cord blood compared to maternal and placental samples [27]. The PUFA in PE and PC are predominantly present at the *sn2* position of the phospholipid backbone, and the placental endothelial lipases cleave the *sn1* fatty acid allowing the fetal uptake and transport of lyso-PL containing PUFA [28]. Recent studies have identified MFSD2A as the lyso-PL transporter in placenta [29] as well as brain [30]. Transporters like MFSD2A provide an insight into the maternal-fetal transfer of PUFA containing lyso-PL and their enrichment in cord blood plasma. As PUFA are particularly important for brain and retina development [31–33], an increase in their levels in cord blood indicates their specific requirements for the growing fetus. Overall, we demonstrate that PUFA containing phospholipids are enriched in maternal circulation, but their breakdown products (lyso-PL) are depleted in circulation, which corroborates with their placental uptake and transport to the fetus.

Among the increased lyphospholipids in cord blood we also observed an increase in several ether-linked species including LPC(O) and LPE(P). The ether-linked phospholipids are carriers of atypical and very long chain fatty acids that are primarily synthesized in peroxisomes [34]. Ether lipids have been shown to be co-regulated with cholesterol [35, 36] and sphingolipids [37], and together are involved in membrane homeostasis and membrane trafficking [38]. Similarly, several other lipids from SM, GM_3_-gangliosides, CE, and most of the AC were also elevated in cord blood. These data establish that maternal-fetal transport of lipids is much more complex than previously appreciated. However, detailed studies are needed to provide mechanistic insights into the transfer and metabolism of these diverse lipid species. At the same time, mother’s own metabolic requirements during pregnancy must be considered while interpreting the increase and decrease of various lipid species in maternal circulation. For example, the decrease in maternal acylcarnitine can occur either due to the increased fetal demand, increased beta-oxidation of fatty acids, or accelerated renal clearance during pregnancy [39]. Carnitine is required not only for the fetal growth and maturation, but its adequate status is also critical for the mother, as she needs sufficient amounts to support her energy metabolism during pregnancy. Blood carnitine levels are known to steadily decline during pregnancy, and its deficiency may require administration of exogenous carnitines to avoid metabolic crisis [40, 41]. Although in normal pregnancies a secondary carnitine deficiency may not be noticeable but in complicated pregnancy, this deficiency can be an additional risk factor. Further, maternal and child plasma lipidomic profiles may be influenced by genetics, microbiome, diet, and physical activity [42–47]. Modification to dietary intake in the form of food consumption and supplements has been of particular interest in pregnancy [48–51]. Diets and supplements such as omega-3 PUFA may impact the maternal plasma lipidomic profiles with a subsequent effect on the fetal lipids. However, as demonstrated here because of the diversity of the lipids in maternal and fetal circulation, not only fatty acids (PUFA) are essential but also other constituents such as choline, carnitine, methionine, and serine may play an important role as building blocks and cofactors for the synthesis of diverse lipid species.

Post-birth, significant changes were evident in the offspring plasma lipidome by 6 years of age. The early childhood lipidome also showed a strong resemblance to the postnatal plasma lipidome of the mother (adult), although there were some differences in the plasma lipidomic profiles that included an increase in around 40 lipids that mainly contained EPA and DHA. However, there were higher levels of adrenic acid containing PE-plasmalogens in children. These lipid species may represent an essential pathway of ARA utilization to meet increasing demands for neuronal growth and development in early childhood [52–54]. As is evident from our analyses, levels of 68% of the profiled lipid species increased in circulation with age from birth to 6 years, and 8% of lipids increased in circulation from child to adult. The changes in lipids from birth to 6 years of age (log_2_FC=−2.10 to 6.25) were much higher in magnitude than those observed between pediatric and adult subjects (log_2_FC=−0.68 to 1.18) (Fig. 1E, F). These findings indicate that lipidomic profiles are age-specific and undergo a significant change in their levels in circulation to support growth and development during early childhood.

Our study also advances current understanding of the association between lipids and adiposity, especially in the context of the mother and child, and their intergenerational link. We observed 83% of the overlapped lipid species associated with BMI in pregnant vs non-pregnant states with consistent trend, suggesting a physiological state independent association of these lipids with adiposity. Among these, all the lipid species from deoxy-ceramide, diacylglycerol, and alkyl-diacylglycerol classes showed a positive association with BMI, while ether-linked phospholipids and glycosphingolipids showed negative associations. These observations are also consistent with previous lipidomic studies of BMI in adult populations [17, 55, 56]. Several lipid species from Cer, SM, PC, PE, and LPC showed a positive or negative association with BMI in both pregnant and non-pregnant women depending on the composition of individual lipids. Notably, we also observed 17% of the overlapping BMI-associated lipids had opposite trends in pregnancy compared to the non-pregnant state. This was more apparent in 8 lipid classes (AC, CE, TG, PC, PE, PI, SM, and Cer). All the TG lipid species showed a positive association with BMI in non-pregnant state; several saturated and monounsaturated fatty acid containing TG showed a negative association with BMI in pregnancy. These TG species contained myristic acid, myristoleic acid, stearic acid, linoleic acid, and linolenic acid. Myristic acid containing LPC (14:0) and CE (14:0) also decreased with increasing ppBMI. Dietary intake of myristic acid and alpha-linolenic acid has been shown to modify plasma and erythrocyte lipid profiles [57] and increase the incorporation of DHA in cholesteryl esters [58]. In agreement with other reports in non-pregnant population, these myristic acid containing TG were positively associated with BMI in postnatal phase in our study. Studies have also shown that decreasing TG myristic levels enhanced insulin sensitivity in weight loss intervention [59]. Later phase of pregnancy is associated with increase in insulin resistance [60], necessary for maintaining increased levels of nutrients for fetal transfer, and these data suggest that obesity-related decrease in the levels of TG-myristic acid may alter the physiological insulin homeostasis in pregnancy. Further, the decrease in antenatal plasma levels of several phospholipid, sphingomyelin, and triacylglycerol lipid species in context of higher ppBMI is of particular interest. These are some of the lipid species that showed the most significant changes in antenatal vs postnatal circulation and the decrease in their circulating levels demonstrate the effects of pre-pregnancy adiposity on the plasma lipidomic profiles in pregnancy. Hence, divergent trends of lipid-BMI associations may arise due to the alterations in lipid metabolism in women during pregnancy. Future work focusing on these lipids will provide deeper mechanistic insights into the effects of obesogenic environment on lipid metabolism during gestation.

Around 53% of the cord blood lipids were associated with BW. Lysophospholipids irrespective of the subclass such as LPC, LPC(O), LPE, and LPE(P) showed a positive association with BW. Similarly, all the DG, TG, and TG(O) as well as DeoxyCer showed negative associations with BW. The associations of LPC and LPE were independent of the fatty acid chain length and degree of unsaturation, in agreement with other studies [61]. LPC (16:1) showed the most significant positive association with BW [62] and PUFA containing TG particularly those with DHA were among the most significantly negatively associated lipids. Here, we have extended these findings to several other lipid classes including ether-linked phospholipids, hexosylceramides and deoxy-ceramides. There were sex-stratified associations of cord blood lipid species with BW with more lipids showing associations in males as compared to the females. These differences were more pronounced in phospholipids including ether-linked phospholipids. Comparison of cord blood lipidomic profiles associated with birth weight showed opposite trends to that of the child 6-year-old lipidomic profiles and BMI. The plasma lipidomic profiles at 6 years of age showed sex-stratified differential association with BMI [63]. These differences were more pronounced in PUFA containing LPC that showed a higher increase in percentage with increasing BMI in females. These observations are in agreement with the previous reports on gender-related differences in associations of LPC with age [56]. Only 12% of the lipids, belonging to 6 lipid classes (AC, CE, LPC, PC, PC(P) and PI; Additional file 1: Fig. S8C) showed consistent trends in associations between offspring BW and BMI at 6 years. The divergent trends between BW-lipids and child BMI-lipids could be due the metabolic adaptations to the change in nutrition from in utero maternal supply to intermittent feeding and subsequent changes to the composition of diet in early childhood. Notably, lipid species associated with child BMI were very similar to those associated with adult BMI, although the later was associated with a large number of lipids (67%). This overlap in the effects of adiposity on 6-year-old child and postnatal mothers may be influenced by the similarities in the diet apart from other factors such as genetics and shared lifestyle. At the same time, the distinction in the child and adult associations reflects the age-dependent effects. For example, in children, SM(d18:2/14:0), CE (16:1), PI (16:0_16:1), and PC (18:0_20:3) as well as several PUFA containing TG showed a significant positive association with BMI whereas in adults, TG containing palmitoleic acid, stearic acid, and oleic acid were the most positively associated lipids. These later TG species represent a signature of increased de novo lipogenesis (DNL). Previous studies have shown DNL in obese subjects can lead to lipotoxicity and metabolic disease [64, 65]. Several omega-6 containing lipid species were significantly associated with ppBMI, birthweight, and child BMI, suggesting these represent intergenerational signatures of obesity risk. Validation of ppBMI and BW findings in an independent birth cohort (BIS) identified lipid signatures that were both shared and unique to each cohort, although majority of the lipids(~80%) remained consistent and had the same directionality in the two cohorts.

This study has a few limitations. Our analyses and interpretations of antenatal lipids are restricted to lipid levels in mid-pregnancy, as the blood samples were collected at only one time point in the cohort. Future studies in cohorts with multiple sampling may help profile longitudinal lipid trajectories from preconception to postnatal period, and also study causal relationship between lipids and obesity risk in women. The lack of postnatal maternal and offspring 6-year blood samples in BIS limited our ability to replicate some of the findings from the GUSTO cohort. Nevertheless, comparison of the antenatal and cord blood lipidomic profiles between the two cohorts identified a significant overlap between the obesity-linked lipid signatures. Pre-pregnancy maternal weight and maternal education level were self-reported during recruitment. Hence, bias may exist in the self-reported variables. However, self-reported ppBMI was highly correlated with BMI taken during the recruitment visit in early pregnancy (Additional file 1: Fig. S6). Last, the observations in this study may be limited by lack of relevant dietary patterns/habits information.

## Conclusions

In summary, we describe the population-based developmental and intergenerational landscape of the circulating lipidome. The differential levels of diverse lipid species in cord blood and their associations with BW illustrate the complexity of maternal-fetal transfer of lipids. Future studies are needed for better understanding of the complex interaction between maternal dietary intake, incorporation of fatty acids into lipid fractions and subsequent transport to the developing fetus. The lipids that were significantly different between maternal and fetal circulation were affected by pre-pregnancy BMI, highlighting the importance of managing obesity before pregnancy. Similarly, the sex-stratified associations of lipidome with adiposity measures at birth and early childhood could provide insights into sex differences in cardiometabolic disease. Thus, our study provides an important resource for future research targeting early nutritional interventions to benefit maternal and child metabolic health.

## Supplementary Information

**Additional file 1: Fig. S1.** Outlier detection by principal component analysis of the lipidomics data at four time points. **Fig. S2.** Number of pairs between the participants in antenatal, postnatal, cord blood and child lipidomes. **Fig. S3.** Comparison of the results of linear regression and paired t-test. **Fig. S4.** Ratios of PC to PE levels and lysophospholipids (lyso-PL). **Fig. S5.** Scatter plots of effect sizes and adjusted p-values of plasma lipids associated with mother and child adiposity. **Fig. S6.** Correlation of pre-pregnancy, antenatal and postnatal maternal body mass indices (BMIs). **Fig. S7.** Scatter plot of effect sizes in the pre-pregnancy and antenatal BMI studies. **Fig. S8.** Overlapping plasma lipid signatures associated with mother and child adiposity. **Fig. S9.** Sex difference in the birth weight and child BMI studies. **Fig. S10.** Forest plots and volcano plots of the meta-analysis results in GUSTO and BIS for ppBMI and BW studies. **Table S1A.** Comparison of lipid classes across four sample groups (antenatal, postnatal, cord blood and 6-year-old child) using linear regression model. **Table S1B.** Comparison of lipids species across four sample groups (antenatal, postnatal, cord blood and 6-year-old child) using linear regression model. **Table S1C.** Comparison of lipids species across four sample groups (antenatal, postnatal, cord blood and 6-year-old child) using paired t-test. **Table S2A.** Association of lipid classes with BMI or BW in four sample groups (antenatal, postnatal, cord blood and 6-year-old child) in terms of levels. **Table S2B.** Association of lipid species with BMI or BW in four sample groups (antenatal, postnatal, cord blood and 6-year-old child) in terms of levels. **Table S2C.** Association of antenatal lipidomic profiles with antenatal BMI. **Table S3A.** The overlapping lipid species between the ppBMI and postnatal BMI studies. **Table S3B.** The overlapping lipid species between the ppBMI and birth weight studies. **Table S3C.** The overlapping lipid species between the birth weight and 6-year-old child BMI studies. **Table S3D.** The overlapping lipid species between the 6-year-old child BMI and adult (postnatal mother) BMI studies. **Table S3E.** Overlapping lipid species in the four lipid-adiposity association studies. **Table S4A.** Sex difference in the birth weight and child BMI studies in terms of lipid class levels. **Table S4B.** Sex difference in the birth weight and child BMI in terms of species levels. **Table S5.** Association studies of lipids with pre-pregnancy BMI and birth weight in Barwon Infant Study (BIS), and the meta-analysis results of GUSTO and BIS.

## Data Availability

The data supporting the findings and figures in this study are provided in the supplementary materials. Data are not publicly available due to ethical restrictions but can be obtained from the authors upon reasonable request and subject to appropriate approvals, including from the GUSTO cohort’s Executive Committee.

## Abbreviations

AC: Acylcarnitine
ARA: Arachidonic acid
BMI: Body mass index
CE: Cholesteryl ester
Cer: Ceramide
deoxyCer: Deoxy-ceramide
DG: Diacylglycerol
DHA: Docosahexaenoic acid
dhCer: Dihydroceramide
GM3: GM3 ganglioside
Hex2Cer: Dihexosylceramide
Hex3Cer: Trihexosylceramide
HexCer: Monohexosylceramide
LC-MS/MS: Liquid chromatography mass spectrometry
LPC: Lysophosphatidylcholine
LPC(O): Lysoalkylphosphatidylcholine
LPC(P): Lysoalkenylphosphatidylcholine
LPE: Lysophosphatidylethanolamine
LPE(P): Lysoalkenylphosphatidylethanolamine
LPI: Lysophosphatidylinositol
NL: Neutral loss
PC: Phosphatidylcholine
PC(O): Alkylphosphatidylcholine
PC(P): Alkenylphosphatidylcholine
PE: Phosphatidylethanolamine
PE(O): Alkylphosphatidylethanolamine
PE(P): Alkenylphosphatidylethanolamine
PG: Phosphatidylglycerol
PI: Phosphatidylinositol
SM: Sphingomyelin
TG: Triacylglycerol
TG(O): Alkyl-diacylglycerol
