# Supplementary material for: Population-based plasma lipidomics reveals developmental changes in metabolism and signatures of obesity risk: a mother-offspring cohort study
Source: BMC Med. 2022 Jul 25;20:242. doi: 10.1186/s12916-022-02432-y (PMC9310480; doi:10.1186/s12916-022-02432-y)
Supplement: Supplementary file 1 — Additional file 1: Fig. S1. Outlier detection by principal component analysis of the lipidomics data at four time points. Fig. S2. Number of pairs between the participants in antenatal, postnatal, cord blood and child lipidomes. Fig. S3. Comparison of the results of linear regression and paired t-test. Fig. S4. Ratios of PC to PE levels and lysophospholipids (lyso-PL). Fig. S5. Scatter plots of effect sizes and adjusted p-values of plasma lipids associated with mother and child adiposity. Fig. S6. Correlation of pre-pregnancy, antenatal and postnatal maternal body mass indices (BMIs). Fig. S7. Scatter plot of effect sizes in the pre-pregnancy and antenatal BMI studies. Fig. S8. Overlapping plasma lipid signatures associated with mother and child adiposity. Fig. S9. Sex difference in the birth weight and child BMI studies. Fig. S10. Forest plots and volcano plots of the meta-analysis results in GUSTO and BIS for ppBMI and BW studies. Table S1A. Comparison of lipid classes across four sample groups (antenatal, postnatal, cord blood and 6-year-old child) using linear regression model. Table S1B. Comparison of lipids species across four sample groups (antenatal, postnatal, cord blood and 6-year-old child) using linear regression model. Table S1C. Comparison of lipids species across four sample groups (antenatal, postnatal, cord blood and 6-year-old child) using paired t-test. Table S2A. Association of lipid classes with BMI or BW in four sample groups (antenatal, postnatal, cord blood and 6-year-old child) in terms of levels. Table S2B. Association of lipid species with BMI or BW in four sample groups (antenatal, postnatal, cord blood and 6-year-old child) in terms of levels. Table S2C. Association of antenatal lipidomic profiles with antenatal BMI. Table S3A. The overlapping lipid species between the ppBMI and postnatal BMI studies. Table S3B. The overlapping lipid species between the ppBMI and birth weight studies. Table S3C. The overlapping lipid species between the [file 12916_2022_2432_MOESM1_ESM.pdf]

Supplementary Information

Population-based plasma lipidomics reveals developmental changes in metabolism and signatures of obesity risk: a mother-offspring cohort study

Sartaj Ahmad Mir, Li Chen, Satvika Burugupalli, Bo Burla, Shanshan Ji, Adam Alexander T. Smith, Kothandaraman Narasimhan, Adaikalavan Ramasamy, Karen Mei-Ling Tan, Kevin Huynh, Corey Giles, Ding Mei, Gerard Wong, Fabian Yap, Kok Hian Tan, Fiona Collier, Richard Saffery, Peter Vuillermin, Anne K. Bendt, David Burgner, Anne-Louise Ponsonby, Yung Seng Lee, Yap Seng Chong, Peter D Gluckman, Johan G. Eriksson, Peter J. Meikle, Markus R. Wenk and Neerja Karnani

Additional File 1: Fig. S1 – 10 and Table S1 – 5

Fig. S1. Outlier detection by principal component analysis of the lipidomics data at four time points
Fig. S2. Number of pairs between the participants in antenatal, postnatal, cord blood and child lipidomes
Fig. S3. Comparison of the results of linear regression and paired t-test
Fig. S4. Ratios of PC to PE levels and lysophospholipids (lysoPL)
Fig. S5. Scatter plots of effect sizes and adjusted p-values of plasma lipids associated with mother and child adiposity
Fig. S6. Correlation of pre-pregnancy, antenatal and postnatal maternal body mass indices (BMIs)
Fig. S7. Scatter plot of effect sizes in the pre-pregnancy and antenatal BMI studies.
Fig. S8. Overlapping plasma lipid signatures associated with mother and child adiposity
Fig. S9. Sex difference in the birth weight and child BMI studies
Fig. S10. Forest plots and volcano plots of the meta-analysis results in GUSTO and BIS for ppBMI and BW studies
Table S1A. Comparison of lipid classes across four sample groups (antenatal, postnatal, cord blood and 6-year-old child) using linear regression model
Table S1B. Comparison of lipid species across four sample groups (antenatal, postnatal, cord blood and 6-year-old child) using linear regression model
Table S1C. Comparison of lipid species across four sample groups (antenatal, postnatal, cord blood and 6-year-old child) using paired t-test.
Table S2A. Association of lipid classes with BMI or BW in four sample groups (antenatal, postnatal, cord blood and 6-year-old child) in terms of levels
Table S2B. Association of lipid species with BMI or BW in four sample groups (antenatal, postnatal, cord blood and 6-year-old child) in terms of levels
Table S2C. Association of antenatal lipidomic profiles with antenatal BMI
Table S3A. The overlapping lipid species between the ppBMI and postnatal BMI studies
Table S3B. The overlapping lipid species between the ppBMI and birth weight studies
Table S3C. The overlapping lipid species between the birth weight and 6-year-old child BMI studies
Table S3D. The overlapping lipid species between the 6-year-old child BMI and adult (postnatal mother) BMI studies
Table S3E. The overlapping lipid species in the four lipid-adiposity association studies
Table S4A. Sex difference in the birth weight and child BMI studies in terms of lipid class levels
Table S4B. Sex difference in the birth weight and child BMI in terms of species levels
Table S5. Association studies of lipids with pre-pregnancy BMI and birth weight in Barwon Infant Study (BIS), and the meta-analysis results of GUSTO and BIS

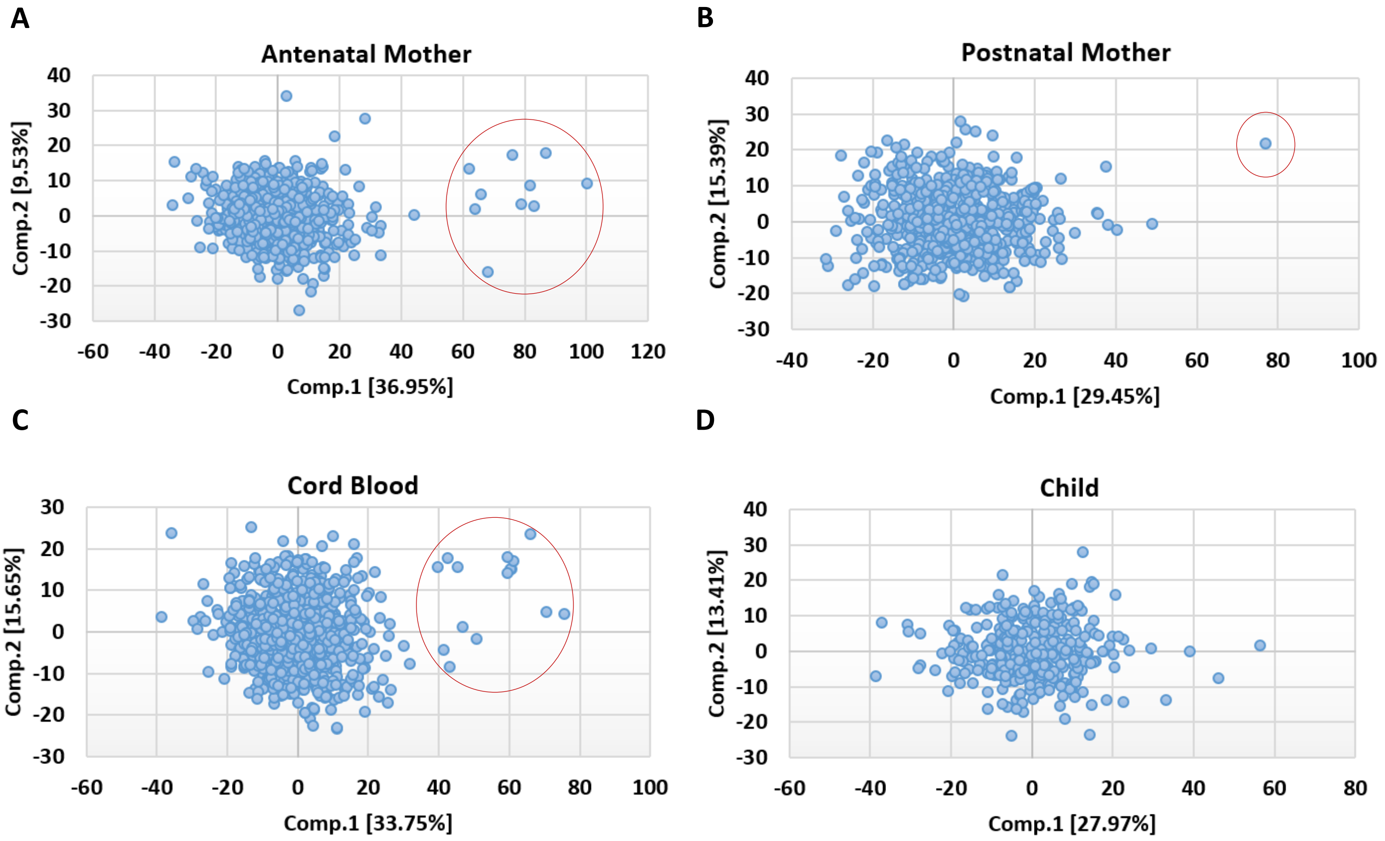

Fig. S1. Outlier detection by principal component analysis of the lipidomics data at four time points: (**A**) Antenatal mother (n=763). Eleven outliers are detected. (**B**) Postnatal mother (n=651). One outlier is detected (**C**) Cord blood (n=767). Fifteen outliers are detected. (**D**) child (n=338). No outlier is detected. The subjects within red ellipses are outliers.

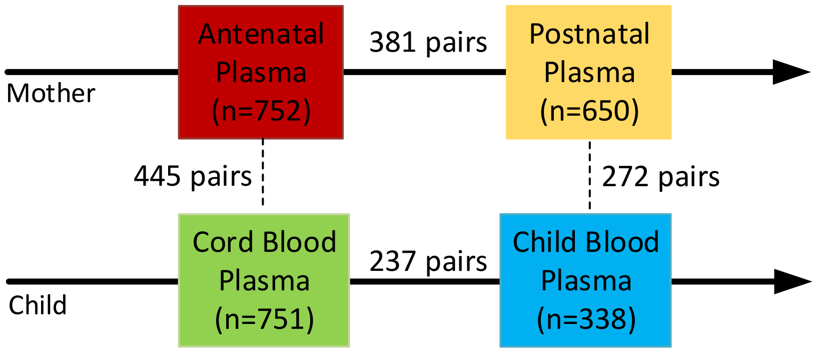

Fig. S2. Number of pairs between the participants in antenatal, postnatal, cord blood and child lipidomes

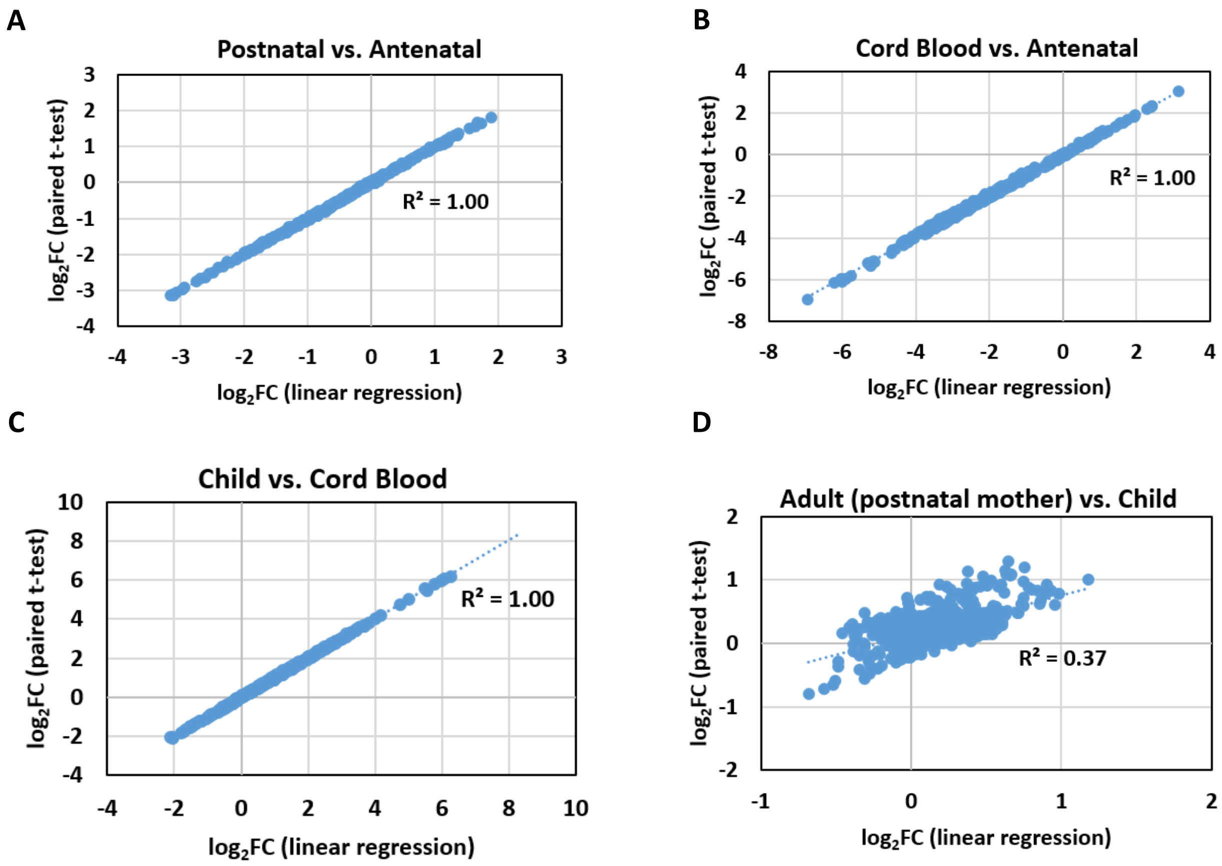

Fig. S3. Comparison of the results of linear regression and paired t-test: (**A**) Postnatal vs. Antenatal. (**B**) Cord Blood vs. Antenatal. (**C**) Child vs. Cord Blood. (**D**) Adult (postnatal mother) vs. Child.

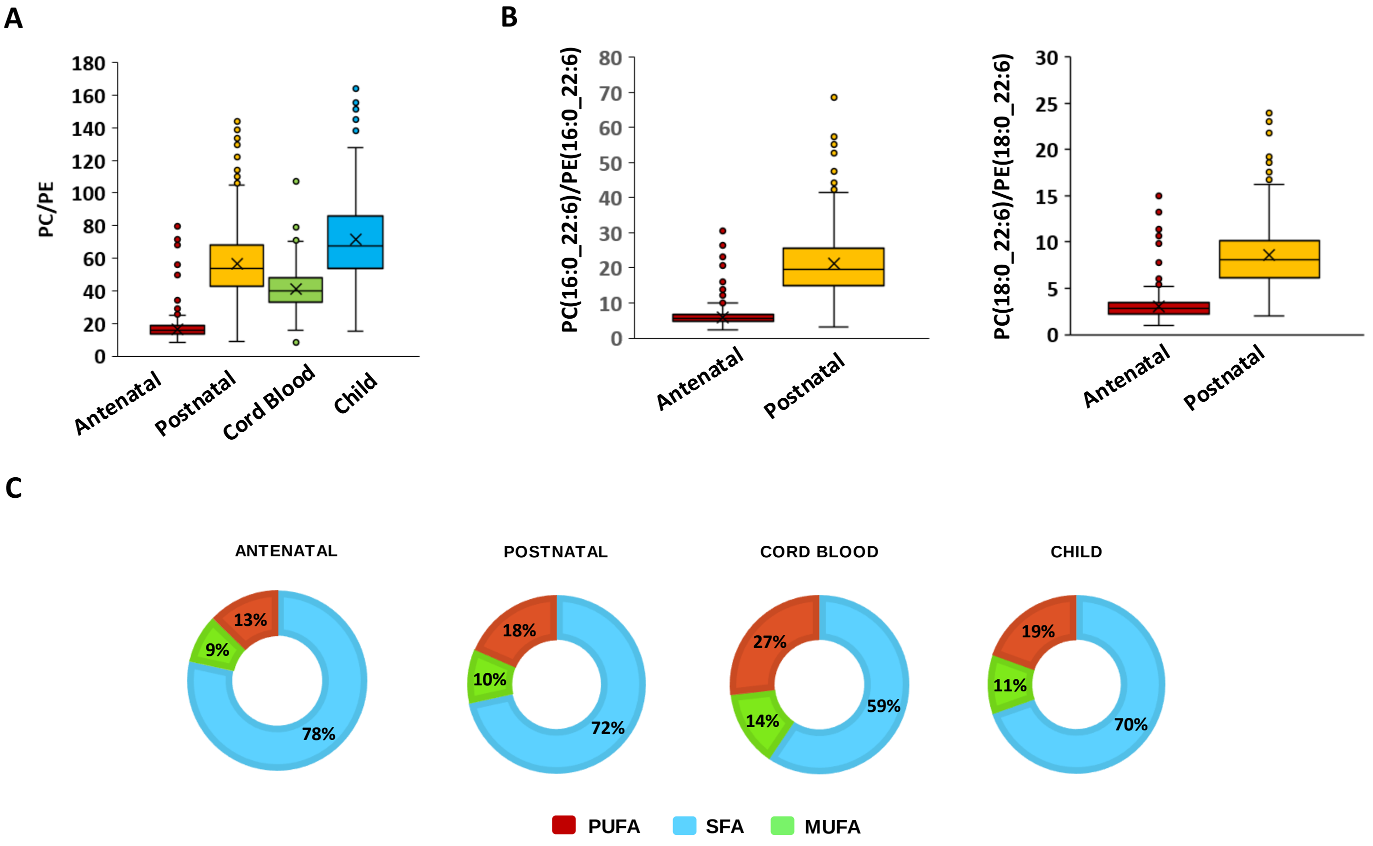

Fig. S4. Ratios of PC to PE levels and lysophospholipids (lysoPL): (**A**) Ratios of total PC to total PE levels in four sample groups. (**B**) Ratios of PC and PE lipid species containing DHA in antenatal and postnatal samples. (**C**) Percentage of lysoPL containing saturated fatty acid (SFA), monounsaturated fatty acid (MUFA) and polyunsaturated fatty acid (PUFA) in antenatal, postnatal, cord blood and child plasma.

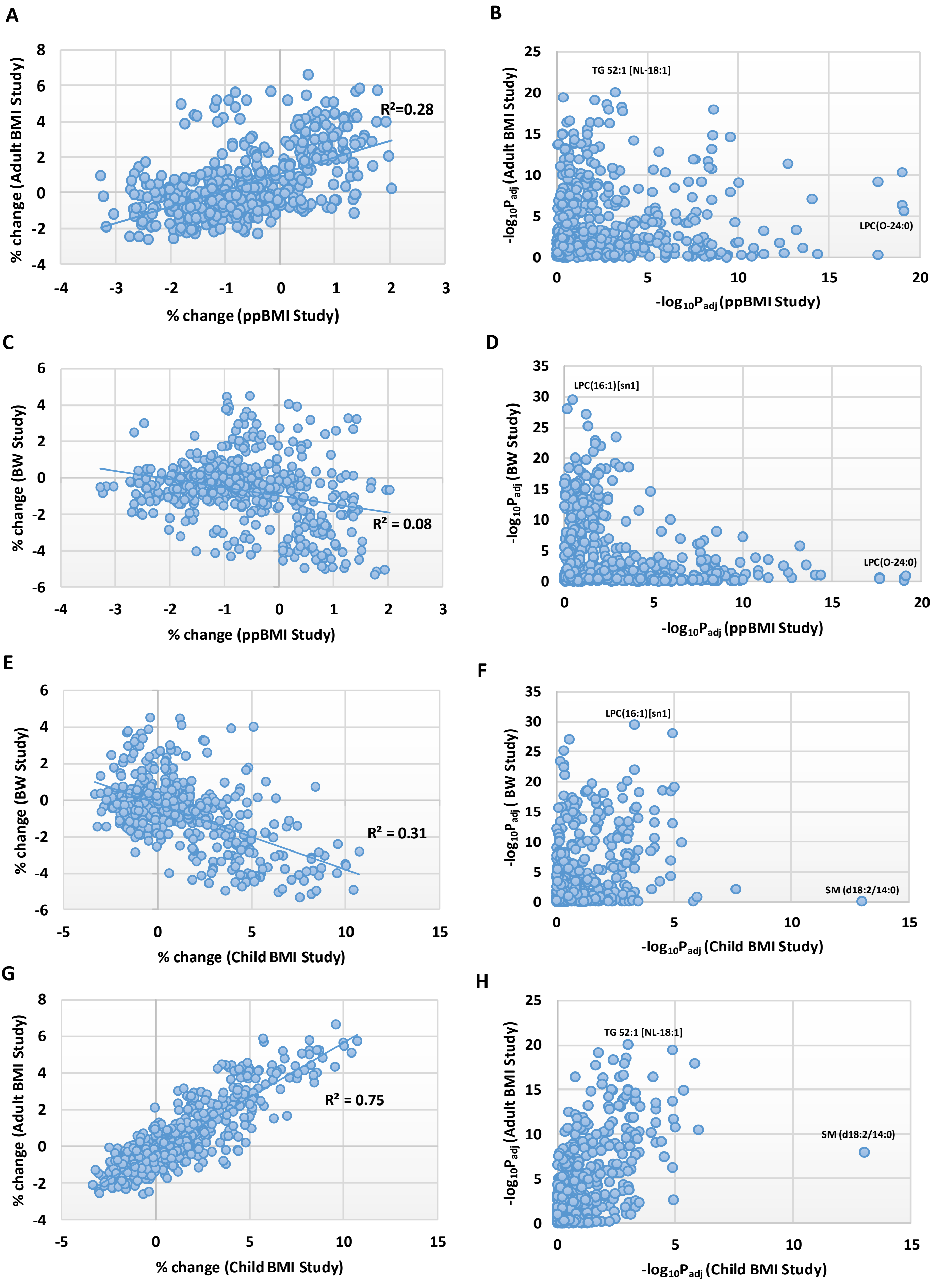

Fig. S5. Scatter plots of effect sizes and adjusted p-values of circulatory lipids associated with mother and child adiposity: (**A-B**) Pre-pregnancy BMI study vs. Adult (postnatal mother) BMI study. (**C-D**) Pre-pregnancy BMI study vs. BW study. (**E-F**) BW study vs. Child BMI study (6 years of age). (**G-H**) Child BMI study vs. Adult (postnatal mother) BMI study. Effect size is shown as % change in lipid concentration per unit BMI for BMI associations and per 100 grams for birth weight. The most significant lipid species are labelled in **B**,**D**,**F** and **H**.

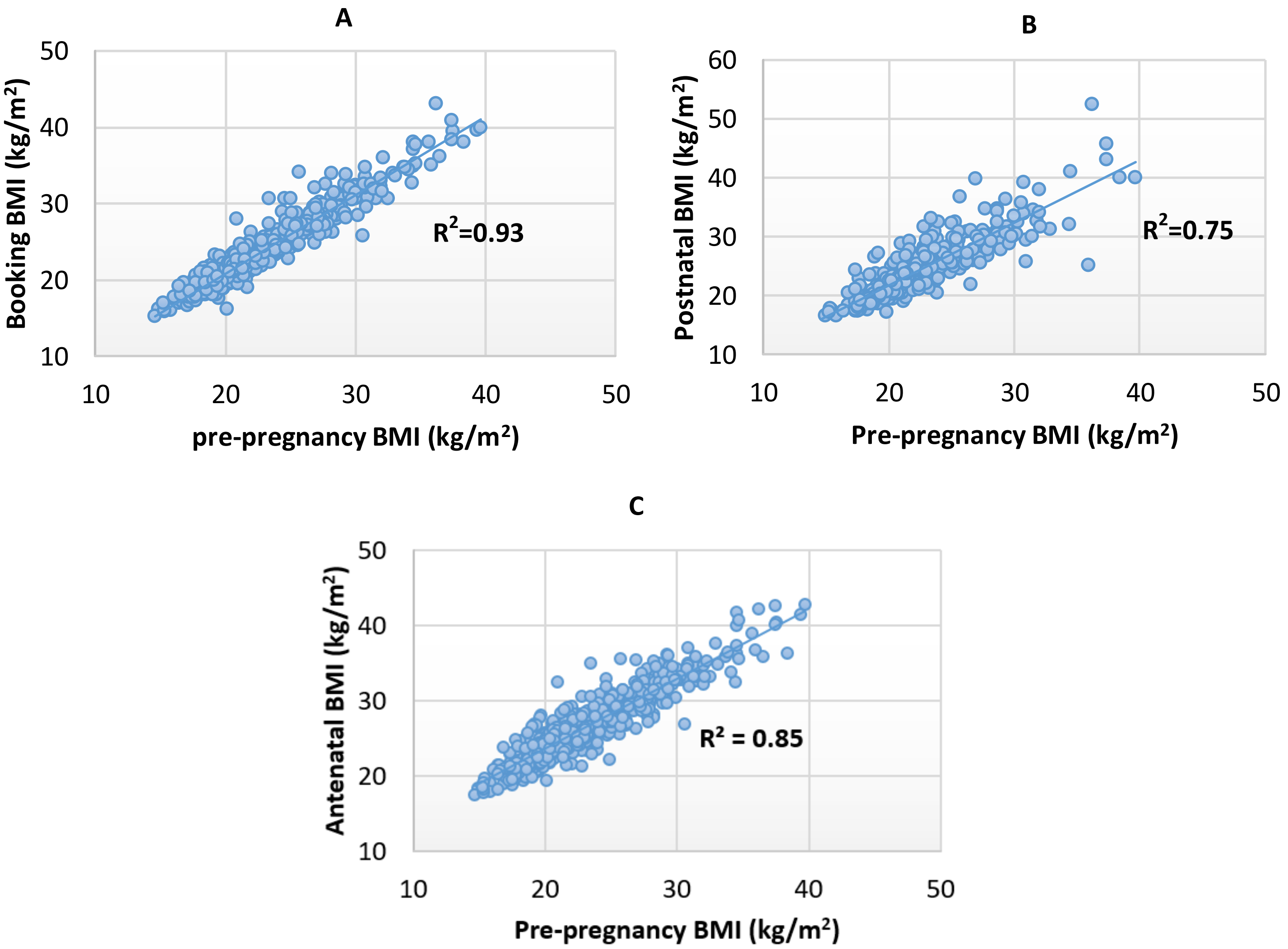

Fig. S6. Correlation of pre-pregnancy, antenatal and postnatal maternal body mass indices (BMIs): (**A**) Pre-pregnancy BMI vs booking BMI measured at the first clinical visit. (**B**) Pre-pregnancy BMI vs postnatal BMI (4-5 years after delivery). (**C**) Pre-pregnancy BMI vs. antenatal BMI (26-28 weeks of gestation)

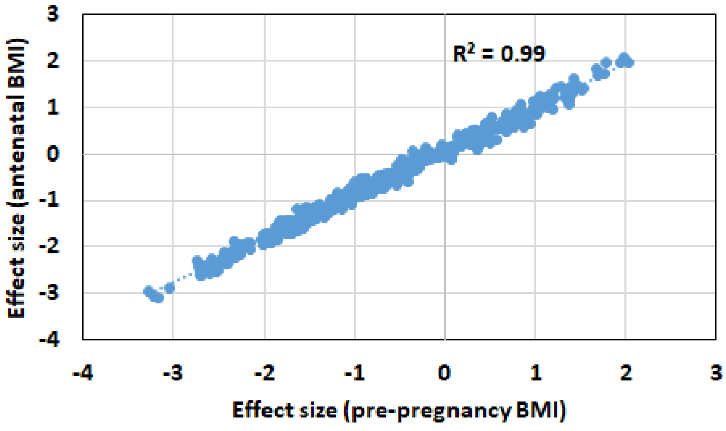

Fig. S7. Scatter plot of effect sizes in the pre-pregnancy and antenatal BMI studies. Effect size is % change in lipid concentration per unit BMI (kg/m^2)

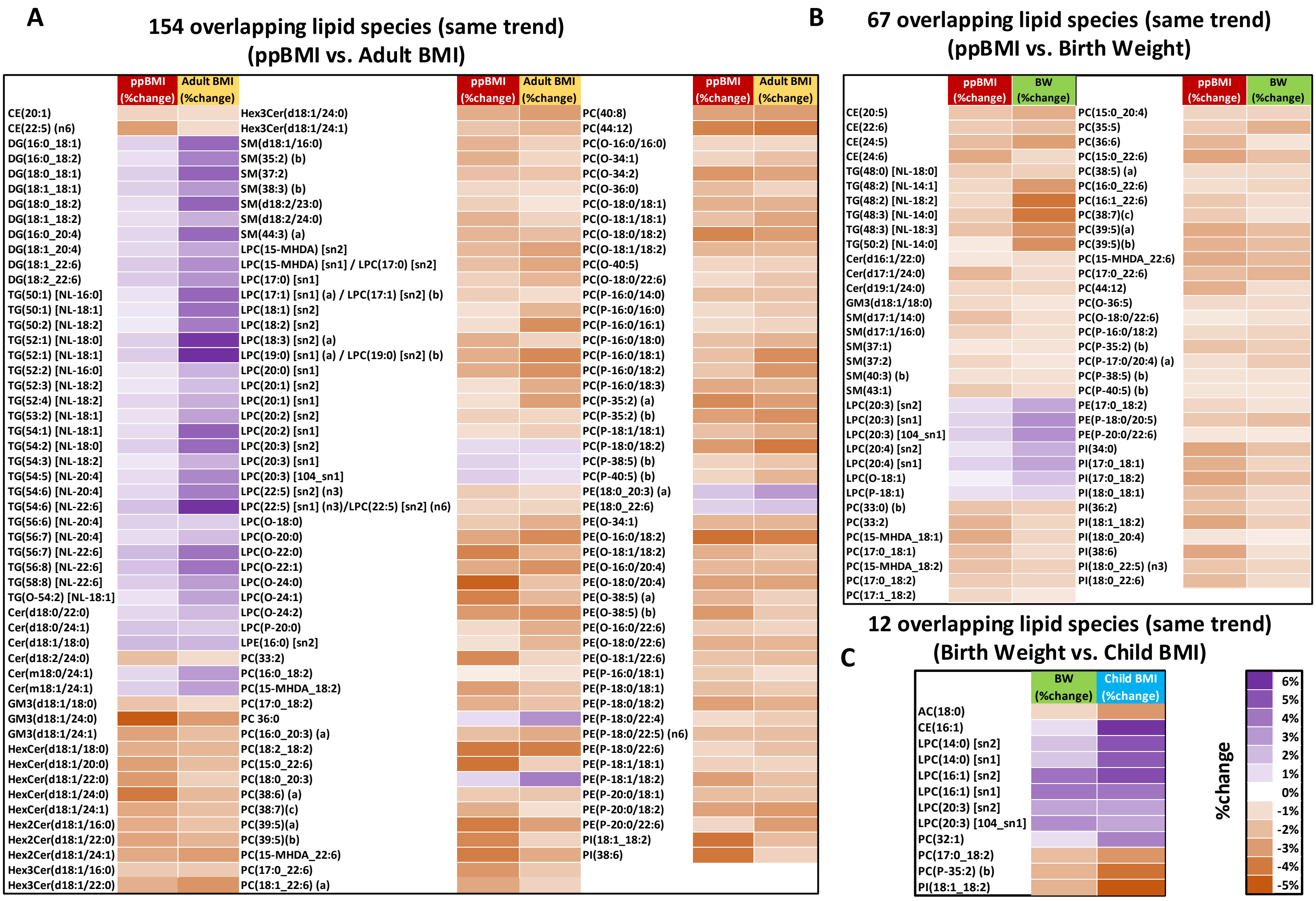

Fig. S8. Overlapping circulatory lipid signatures associated with mother and child adiposity: (**A**) Heat map of 154 overlapping lipid species with the same directionality of association between antenatal ppBMI study and adult (postnatal mother) BMI study. (**B**) Heat map of 67 overlapping lipid species with the same association trend between antenatal ppBMI study and cord blood BW study. (**C**) Heat map of 12 overlapping lipid species with the same association trend between cord blood BW study and child BMI study.

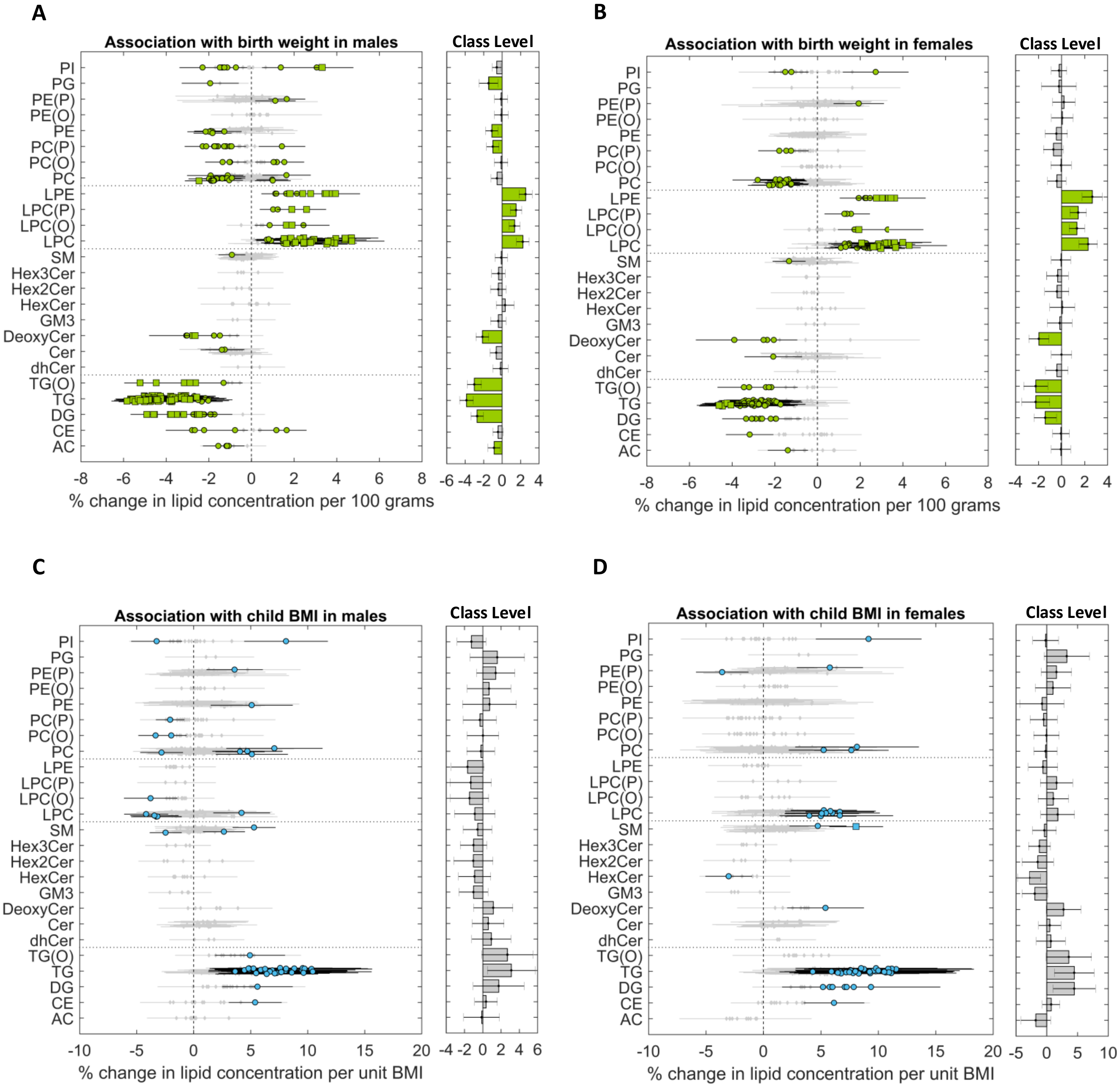

Fig. S9. Sex difference in the birth weight and child BMI studies: (**A**) Association with birth weight in males. (**B**) Association with birth weight in females. (**C**) Association with child BMI in males. (**D**) Association with child BMI in females. Diamond – $P_{adj} \geq 0.05$ (grey), circle – $P_{adj} < 0.05$ and square – $P_{adj} < 1.00E\text{-}5$.

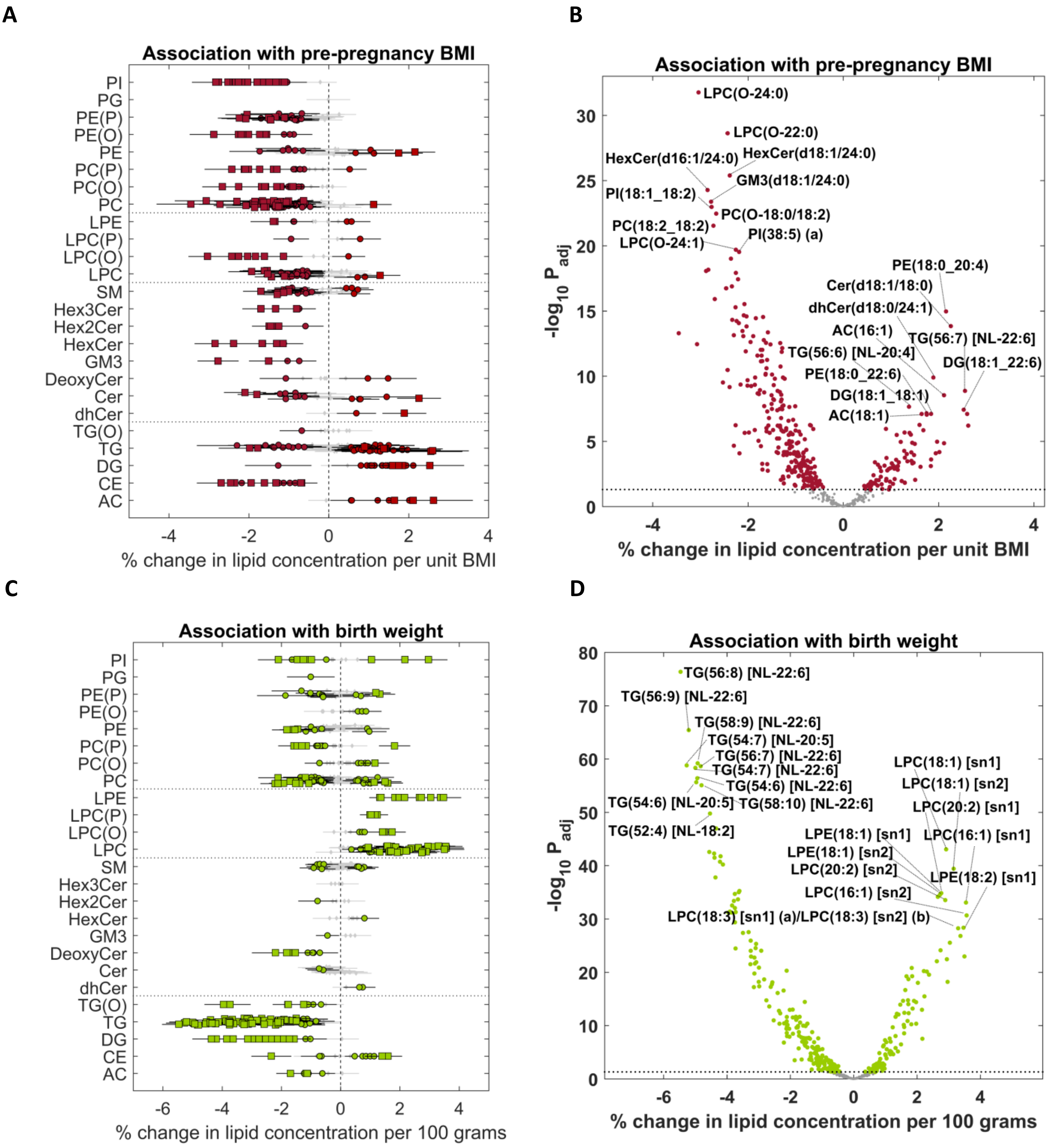

Fig. S10. Forest plots and volcano plots of the meta-analysis results in GUSTO and BIS for ppBMI (**A** and **B**) and BW studies (**C** and **D**). Diamond – $P_{adj} \geq 0.05$ (grey), circle – $P_{adj} < 0.05$ and square – $P_{adj} < 1.00E-5$ in forest plots (**A** and **C**). Top 10 significant lipid species with positive and negative association are labelled in volcano plots (**B** and **D**, dotted line – $P_{adj} = 0.05$).

Table S1A. Comparison of lipid classes across four sample groups (antenatal, postnatal, cord blood and 6-year-old child) using linear regression model. Related to Figure 1.

	**From antenatal to postnatal**				**From antenatal to cord blood**				**From birth to child**				**From child to adult (postnatal)**			
	adjusted for ethnicity, maternal age, maternal education level and pre-pregnancy BMI				adjusted for ethnicity, maternal age, maternal education level, pre-pregnancy BMI, sex, gestational age and birth weight				adjusted for sex, ethnicity, maternal education level, gestational age and birth weight				adjusted for sex, ethnicity, BMI and maternal education level			
Lipid Class	log_2FC (Postnatal/Antenatal)	95%CI	p-value	p-value(BH)	log_2FC (CordBlood/Antenatal)	95%CI	p-value	p-value(BH)	log_2FC (Child/CordBlood)	95%CI	p-value	p-value(BH)	log_2FC (Adult/Child)	95%CI	p-value	p-value(BH)
AC	1.23	(1.19-1.28)	**2.31E-293**	**1.92E-292**	0.40	(0.34-0.46)	**9.89E-38**	**1.08E-37**	0.70	(0.64-0.76)	**1.82E-107**	**2.68E-107**	0.04	(-0.06-0.14)	4.23E-01	5.28E-01
CE	-0.05	(-0.07--0.02)	**1.05E-03**	**1.19E-03**	-1.56	(-1.6--1.52)	**0.00E+00**	**0.00E+00**	1.34	(1.3-1.38)	**0.00E+00**	**0.00E+00**	0.24	(0.18-0.3)	**5.16E-15**	**2.15E-14**
DG	-1.01	(-1.08--0.95)	**1.67E-161**	**4.18E-161**	-3.04	(-3.11--2.96)	**0.00E+00**	**0.00E+00**	1.60	(1.53-1.68)	**1.74E-236**	**3.62E-236**	-0.05	(-0.19-0.1)	5.50E-01	6.54E-01
TG	-1.00	(-1.06--0.95)	**5.58E-211**	**1.99E-210**	-2.87	(-2.95--2.79)	**0.00E+00**	**0.00E+00**	1.32	(1.23-1.41)	**8.51E-134**	**1.42E-133**	0.03	(-0.11-0.16)	6.98E-01	7.58E-01
TG(O)	-0.97	(-1.03--0.91)	**8.38E-160**	**1.91E-159**	-2.95	(-3.03--2.87)	**0.00E+00**	**0.00E+00**	1.55	(1.47-1.63)	**1.27E-191**	**2.26E-191**	-0.09	(-0.24-0.06)	2.60E-01	3.42E-01
dhCer	-0.12	(-0.17--0.07)	**7.00E-06**	**8.75E-06**	0.22	(0.16- 0.28)	**8.89E-13**	**8.89E-13**	-0.48	(-0.54--0.42)	**1.01E-50**	**1.27E-50**	-0.02	(-0.13-0.09)	7.40E-01	7.71E-01
Cer	0.18	(0.14- 0.22)	**4.54E-19**	**6.67E-19**	-2.14	(-2.19--2.09)	**0.00E+00**	**0.00E+00**	2.10	(2.05- 2.15)	**0.00E+00**	**0.00E+00**	0.17	(0.09-0.26)	**7.48E-05**	**1.25E-04**
DeoxyCer	-0.61	(-0.67--0.55)	**1.63E-69**	**3.39E-69**	-1.96	(-2.03--1.88)	**0.00E+00**	**0.00E+00**	0.87	(0.8-0.93)	**2.58E-118**	**4.03E-118**	0.13	(0-0.25)	5.46E-02	7.58E-02
GM3	0.20	(0.16-0.25)	**7.95E-18**	**1.10E-17**	-0.25	(-0.31--0.19)	**1.61E-15**	**1.67E-15**	0.37	(0.31-0.43)	**3.97E-32**	**4.51E-32**	0.24	(0.15-0.33)	**2.64E-07**	**5.49E-07**
HexCer	0.12	(0.08-0.16)	**1.31E-08**	**1.72E-08**	-1.30	(-1.36--1.24)	**1.24E-247**	**1.83E-247**	1.35	(1.29-1.41)	**2.88E-238**	**6.54E-238**	0.23	(0.14-0.32)	**5.09E-07**	**9.78E-07**
Hex2Cer	-0.07	(-0.12--0.02)	**3.61E-03**	**3.92E-03**	-2.17	(-2.23--2.11)	**0.00E+00**	**0.00E+00**	1.76	(1.69-1.82)	**4.98E-304**	**1.38E-303**	0.51	(0.41-0.62)	**1.79E-20**	**2.24E-19**
Hex3Cer	0.19	(0.15-0.22)	**2.80E-22**	**4.38E-22**	-0.85	(-0.9--0.8)	**1.89E-171**	**2.62E-171**	1.06	(1.01-1.11)	**3.87E-212**	**7.44E-212**	0.11	(0.03-0.19)	**7.60E-03**	**1.12E-02**
SM	-0.32	(-0.36--0.28)	**1.64E-51**	**3.16E-51**	-1.85	(-1.9--1.8)	**0.00E+00**	**0.00E+00**	1.29	(1.24-1.34)	**2.07E-298**	**5.17E-298**	0.30	(0.22-0.39)	**6.36E-12**	**1.77E-11**
LPC	0.90	(0.86-0.95)	**7.17E-246**	**2.99E-245**	0.40	(0.36-0.45)	**7.90E-60**	**8.98E-60**	0.25	(0.2-0.3)	**3.53E-21**	**3.67E-21**	0.49	(0.38-0.59)	**5.78E-20**	**3.61E-19**
LPC(O)	0.87	(0.83-0.9)	**8.75E-280**	**5.47E-279**	0.37	(0.33-0.41)	**1.82E-66**	**2.17E-66**	0.25	(0.2-0.29)	**7.27E-25**	**7.91E-25**	0.46	(0.37-0.56)	**8.65E-22**	**2.16E-20**
LPC(P)	1.14	(1.1-1.18)	**0.00E+00**	**0.00E+00**	0.68	(0.63-0.72)	**1.26E-163**	**1.66E-163**	0.17	(0.13-0.22)	**3.49E-12**	**3.49E-12**	0.47	(0.38-0.57)	**3.10E-20**	**2.59E-19**
LPE	0.31	(0.27-0.36)	**1.57E-46**	**2.80E-46**	0.49	(0.45-0.54)	**4.94E-88**	**6.17E-88**	-0.38	(-0.43--0.33)	**1.79E-49**	**2.14E-49**	0.44	(0.34-0.54)	**2.79E-18**	**1.39E-17**
PC	-0.63	(-0.67--0.6)	**5.16E-194**	**1.61E-193**	-2.35	(-2.39--2.3)	**0.00E+00**	**0.00E+00**	1.56	(1.51-1.6)	**0.00E+00**	**0.00E+00**	0.21	(0.13-0.29)	**6.15E-07**	**1.10E-06**
PC(O)	-0.22	(-0.27--0.18)	**4.62E-24**	**7.70E-24**	-1.85	(-1.91--1.8)	**0.00E+00**	**0.00E+00**	1.46	(1.41-1.51)	**0.00E+00**	**0.00E+00**	0.33	(0.24-0.42)	**4.38E-12**	**1.37E-11**
PC(P)	-0.08	(-0.13--0.04)	**5.40E-04**	**6.43E-04**	-1.76	(-1.81--1.7)	**0.00E+00**	**0.00E+00**	1.50	(1.44-1.55)	**6.78E-307**	**2.12E-306**	0.37	(0.27-0.46)	**2.66E-13**	**9.49E-13**
PE	-2.36	(-2.42--2.29)	**0.00E+00**	**0.00E+00**	-3.70	(-3.76--3.64)	**0.00E+00**	**0.00E+00**	0.82	(0.75-0.88)	**4.98E-100**	**6.55E-100**	0.48	(0.32-0.63)	**3.67E-09**	**9.17E-09**
PE(O)	0.02	(-0.04-0.07)	5.93E-01	5.93E-01	-2.94	(-3--2.87)	**0.00E+00**	**0.00E+00**	2.77	(2.71-2.83)	**0.00E+00**	**0.00E+00**	0.35	(0.23-0.48)	**7.57E-08**	**1.72E-07**
PE(P)	-0.06	(-0.11--0.01)	**2.80E-02**	**2.92E-02**	-2.70	(-2.76--2.64)	**0.00E+00**	**0.00E+00**	2.73	(2.67-2.78)	**0.00E+00**	**0.00E+00**	0.01	(-0.1-0.12)	8.06E-01	8.06E-01
PG	-1.04	(-1.1--0.98)	**1.19E-173**	**3.31E-173**	-2.50	(-2.58--2.42)	**0.00E+00**	**0.00E+00**	1.11	(1.02-1.2)	**5.87E-105**	**8.15E-105**	0.04	(-0.11-0.2)	5.88E-01	6.68E-01
PI	-1.04	(-1.08--1)	**8.26E-279**	**4.13E-278**	-2.36	(-2.4--2.31)	**0.00E+00**	**0.00E+00**	1.18	(1.14-1.22)	**0.00E+00**	**0.00E+00**	0.15	(0.06-0.25)	**1.21E-03**	**1.89E-03**

Table S1B. Comparison of lipid species across four sample groups (antenatal, postnatal, cord blood and 6-year-old child) using linear regression model. Related to Figure 1.

Studies			From antenatal to postnatal				From antenatal to cord blood				From birth to child				From child to adult (postnatal)			
covariates			adjusted for ethnicity, maternal age, maternal education level and pre-pregnancy BMI				adjusted for ethnicity, maternal age, maternal education level, pre-pregnancy BMI, sex, gestational age and birth weight				adjusted for sex, ethnicity, maternal education level, gestational age and birth weight				adjusted for sex, ethnicity, BMI and maternal education level			
No.	Class	Lipid Species	log_2FC (Postnatal/Antenatal)	95%CI	p-value	p-value(BH)	log_2FC (CordBlood/Antenatal)	95%CI	p-value	p-value(BH)	log_2FC (Child/CordBlood)	95%CI	p-value	p-value(BH)	log_2FC (Adult/Child)	95%CI	p-value	p-value(BH)
1	AC	AC(12:0)	1.64	(1.56-1.72)	4.90E-214	3.02E-213	-0.04	(-0.13-0.04)	3.28E-01	3.30E-01	1.80	(1.72-1.87)	7.53E-254	2.56E-253	-0.22	(-0.38--0.06)	7.68E-03	1.22E-02
2	AC	AC(13:0)	1.21	(1.09-1.32)	1.72E-81	3.69E-81	0.47	(0.34-0.6)	1.49E-12	1.60E-12	-0.19	(-0.31--0.06)	3.21E-03	3.34E-03	0.80	(0.56-1.05)	1.98E-10	8.54E-10
3	AC	AC(14:0)	1.10	(1.05-1.16)	2.16E-211	1.29E-210	0.84	(0.77-0.9)	8.03E-113	9.99E-113	0.27	(0.2-0.33)	1.04E-15	1.18E-15	-0.15	(-0.27--0.03)	1.12E-02	1.74E-02
4	AC	AC(14:1)	1.90	(1.81-1.99)	4.36E-237	3.13E-236	0.23	(0.14-0.33)	2.81E-06	2.97E-06	1.79	(1.7-1.87)	1.03E-228	3.18E-228	-0.33	(-0.51--0.16)	1.69E-04	3.40E-04
5	AC	AC(14:2)	1.74	(1.65-1.83)	1.03E-225	6.84E-225	0.50	(0.41-0.59)	1.71E-26	1.88E-26	1.45	(1.37-1.52)	2.19E-206	5.98E-206	-0.34	(-0.51--0.18)	3.93E-05	9.03E-05
6	AC	AC(16:0)	0.92	(0.88-0.96)	9.06E-251	7.63E-250	0.67	(0.61-0.72)	3.29E-112	4.07E-112	0.04	(-0.01-0.09)	1.15E-01	1.17E-01	0.16	(0.08-0.24)	8.22E-05	1.75E-04
7	AC	AC(16:1)	1.36	(1.29-1.42)	3.38E-236	2.38E-235	0.98	(0.9-1.05)	1.05E-112	1.30E-112	0.13	(0.06-0.21)	2.88E-04	3.02E-04	-0.02	(-0.15-0.1)	7.14E-01	7.54E-01
8	AC	AC(18:0)	1.02	(0.98-1.06)	1.26E-271	1.35E-270	0.34	(0.28-0.39)	1.98E-33	2.19E-33	0.61	(0.56-0.66)	2.16E-93	3.51E-93	0.11	(0.02-0.2)	1.53E-02	2.28E-02
9	AC	AC(18:1)	1.19	(1.13-1.24)	8.43E-255	7.23E-254	-0.08	(-0.15--0.01)	2.03E-02	2.08E-02	1.02	(0.95-1.08)	1.82E-159	3.77E-159	0.17	(0.07-0.27)	5.91E-04	1.09E-03
10	AC	AC(18:2)	1.12	(1.07-1.17)	3.00E-243	2.29E-242	0.73	(0.67-0.8)	1.26E-91	1.50E-91	0.11	(0.06-0.17)	1.21E-04	1.28E-04	0.20	(0.1-0.29)	4.58E-05	1.03E-04
11	CE	CE(14:0)	-0.49	(-0.54--0.43)	1.14E-68	2.28E-68	-0.46	(-0.51--0.4)	7.57E-53	8.71E-53	0.02	(-0.04-0.07)	5.52E-01	5.55E-01	-0.12	(-0.23-0)	4.39E-02	6.00E-02
12	CE	CE(16:1)	-0.79	(-0.86--0.73)	1.50E-106	3.99E-106	0.24	(0.16-0.31)	1.99E-10	2.13E-10	-1.26	(-1.32--1.2)	2.07E-216	5.95E-216	-0.06	(-0.18-0.06)	3.02E-01	3.57E-01
13	CE	CE(18:0)	0.15	(0.1-0.2)	1.68E-10	2.12E-10	0.65	(0.59-0.71)	1.30E-77	1.51E-77	-0.57	(-0.64--0.5)	2.27E-54	3.13E-54	0.16	(0.05-0.27)	4.11E-03	6.74E-03
14	CE	CE(18:1)	-0.29	(-0.32--0.26)	1.34E-86	3.00E-86	-0.85	(-0.89--0.81)	6.17E-266	1.06E-265	0.51	(0.47-0.55)	6.21E-122	1.13E-121	0.08	(0.03-0.14)	3.91E-03	6.43E-03
15	CE	CE(18:2)	-0.12	(-0.15--0.09)	4.21E-15	5.63E-15	-2.69	(-2.73--2.65)	0.00E+00	0.00E+00	2.41	(2.36-2.45)	0.00E+00	0.00E+00	0.22	(0.15-0.28)	5.10E-11	2.42E-10
16	CE	CE(18:3)	0.11	(0.06-0.17)	5.13E-05	5.99E-05	-2.13	(-2.21--2.06)	0.00E+00	0.00E+00	2.21	(2.13-2.29)	0.00E+00	0.00E+00	0.03	(-0.1-0.15)	6.70E-01	7.18E-01
17	CE	CE(20:1)	0.00	(-0.04-0.04)	8.75E-01	8.79E-01	0.14	(0.07-0.21)	3.37E-05	3.53E-05	-0.19	(-0.26--0.12)	8.39E-08	9.05E-08	0.23	(0.14-0.32)	1.00E-06	2.87E-06
18	CE	CE(20:2)	-0.48	(-0.52--0.45)	6.19E-130	1.92E-129	-0.46	(-0.51--0.41)	4.52E-62	5.24E-62	-0.13	(-0.18--0.08)	1.11E-06	1.18E-06	0.18	(0.1-0.25)	2.59E-06	7.02E-06
19	CE	CE(20:4)	0.56	(0.5-0.61)	4.79E-84	1.05E-83	0.39	(0.33-0.45)	3.18E-34	3.54E-34	0.02	(-0.04-0.08)	4.73E-01	4.77E-01	0.32	(0.21-0.42)	2.74E-09	1.03E-08
20	CE	CE(20:5)	1.14	(1.02-1.25)	3.01E-73	6.20E-73	-1.44	(-1.58--1.31)	1.78E-86	2.09E-86	1.97	(1.85-2.1)	2.64E-148	5.23E-148	0.73	(0.49-0.97)	3.03E-09	1.12E-08
21	CE	CE(22:5) (n6)	-0.23	(-0.28--0.19)	9.65E-22	1.36E-21	-0.44	(-0.5--0.37)	1.61E-35	1.80E-35	0.32	(0.25-0.38)	1.48E-21	1.76E-21	-0.04	(-0.13-0.05)	4.18E-01	4.76E-01
22	CE	CE(22:6)	-0.33	(-0.4--0.27)	3.09E-23	4.41E-23	-1.84	(-1.93--1.76)	7.97E-252	1.29E-251	0.97	(0.88-1.06)	2.31E-84	3.62E-84	0.53	(0.38-0.67)	2.09E-12	1.18E-11
23	CE	CE(24:5)	-1.03	(-1.09--0.97)	8.76E-169	3.86E-168	1.21	(1.11-1.3)	4.78E-111	5.88E-111	-2.10	(-2.2--2)	1.29E-226	3.90E-226	0.01	(-0.12-0.14)	9.12E-01	9.27E-01
24	CE	CE(24:6)	-0.73	(-0.79--0.66)	4.32E-91	9.83E-91	-0.34	(-0.43--0.25)	6.45E-13	6.97E-13	-0.26	(-0.35--0.16)	4.64E-08	5.02E-08	-0.03	(-0.16-0.11)	7.11E-01	7.54E-01
25	DG	DG(16:0_16:1)	-1.60	(-1.69--1.51)	3.19E-195	1.74E-194	-2.14	(-2.24--2.04)	7.24E-248	1.16E-247	0.21	(0.12-0.29)	6.67E-06	7.09E-06	-0.26	(-0.43--0.08)	4.83E-03	7.84E-03
26	DG	DG(14:0_18:2)	-1.65	(-1.76--1.55)	1.97E-159	7.76E-159	-3.87	(-4--3.75)	0.00E+00	0.00E+00	1.73	(1.59-1.86)	2.18E-110	3.76E-110	-0.20	(-0.45-0.04)	1.07E-01	1.38E-01
27	DG	DG(16:0_18:1)	-1.60	(-1.67--1.52)	5.56E-247	4.52E-246	-3.28	(-3.36--3.2)	0.00E+00	0.00E+00	1.38	(1.3-1.46)	2.98E-160	6.24E-160	-0.24	(-0.42--0.07)	5.59E-03	9.04E-03
28	DG	DG(16:1_18:1)	-1.11	(-1.19--1.03)	6.92E-126	2.08E-125	-2.44	(-2.53--2.35)	0.00E+00	0.00E+00	0.79	(0.7-0.88)	1.86E-58	2.64E-58	-0.09	(-0.27-0.1)	3.62E-01	4.20E-01
29	DG	DG(16:0_18:2)	-1.47	(-1.54--1.4)	1.78E-231	1.22E-230	-3.72	(-3.81--3.62)	0.00E+00	0.00E+00	1.73	(1.64-1.83)	7.74E-183	1.83E-182	-0.02	(-0.18-0.15)	8.50E-01	8.81E-01
30	DG	DG(18:0_18:1)	-0.97	(-1.04--0.9)	4.89E-130	1.53E-129	-2.58	(-2.66--2.51)	0.00E+00	0.00E+00	1.36	(1.28-1.44)	3.99E-167	8.63E-167	-0.30	(-0.47--0.14)	3.08E-04	5.91E-04
31	DG	DG(18:1_18:1)	-0.88	(-0.94--0.81)	2.33E-111	6.35E-111	-3.29	(-3.37--3.21)	0.00E+00	0.00E+00	2.06	(1.98-2.14)	1.46E-289	5.99E-289	-0.10	(-0.25-0.06)	2.36E-01	2.84E-01
32	DG	DG(18:0_18:2)	-1.06	(-1.14--0.98)	7.58E-129	2.32E-128	-2.90	(-2.99--2.8)	0.00E+00	0.00E+00	1.49	(1.39-1.59)	1.67E-137	3.20E-137	-0.22	(-0.4--0.04)	1.69E-02	2.49E-02
33	DG	DG(18:1_18:2)	-0.78	(-0.85--0.71)	6.54E-95	1.57E-94	-3.81	(-3.9--3.73)	0.00E+00	0.00E+00	2.47	(2.38-2.56)	0.00E+00	0.00E+00	0.13	(-0.03-0.28)	1.07E-01	1.38E-01
34	DG	DG(18:2_18:2)	-0.65	(-0.73--0.56)	2.07E-48	3.63E-48	-4.12	(-4.23--4)	0.00E+00	0.00E+00	2.70	(2.59-2.82)	9.97E-267	3.71E-266	0.34	(0.16-0.52)	2.80E-04	5.43E-04
35	DG	DG(18:1_18:3)	-0.47	(-0.54--0.39)	4.12E-32	6.32E-32	-3.27	(-3.37--3.18)	0.00E+00	0.00E+00	2.30	(2.2-2.4)	1.50E-252	5.07E-252	0.08	(-0.1-0.26)	3.97E-01	4.55E-01
36	DG	DG(16:0_20:4)	-1.55	(-1.63--1.46)	4.30E-193	2.27E-192	-1.43	(-1.53--1.33)	2.29E-142	3.00E-142	-0.48	(-0.57--0.38)	7.21E-23	8.63E-23	-0.13	(-0.31-0.06)	1.83E-01	2.24E-01
37	DG	DG(18:1_20:3)	-0.89	(-0.96--0.82)	4.14E-111	1.12E-110	-1.13	(-1.21--1.05)	1.04E-136	1.36E-136	0.25	(0.17-0.33)	2.13E-09	2.33E-09	-0.31	(-0.47--0.15)	1.60E-04	3.25E-04
38	DG	DG(18:1_20:4)	-0.70	(-0.76--0.63)	3.18E-83	6.87E-83	-1.60	(-1.68--1.52)	6.80E-225	1.05E-224	0.50	(0.42-0.58)	1.28E-32	1.61E-32	0.06	(-0.08-0.21)	3.85E-01	4.44E-01
39	DG	DG(16:0_22:5)	-1.33	(-1.41--1.25)	1.70E-167	7.27E-167	-1.07	(-1.16--0.97)	3.88E-91	4.61E-91	-0.61	(-0.7--0.52)	2.28E-35	2.93E-35	-0.01	(-0.19-0.16)	8.82E-01	9.06E-01
40	DG	DG(18:2_20:4)	-0.70	(-0.77--0.62)	1.01E-64	1.95E-64	-1.94	(-2.04--1.84)	3.73E-213	5.59E-213	0.65	(0.55-0.75)	1.47E-34	1.88E-34	0.21	(0.04-0.37)	1.44E-02	2.17E-02
41	DG	DG(16:0_22:6)	-1.65	(-1.75--1.54)	6.60E-154	2.48E-153	-1.37	(-1.49--1.25)	3.10E-97	3.73E-97	-0.97	(-1.08--0.85)	3.97E-53	5.45E-53	0.06	(-0.17-0.3)	6.09E-01	6.67E-01
42	DG	DG(18:1_22:5)	-0.86	(-0.93--0.78)	1.51E-97	3.65E-97	-1.00	(-1.09--0.91)	2.39E-89	2.83E-89	-0.11	(-0.2--0.02)	1.85E-02	1.90E-02	-0.04	(-0.2-0.12)	6.09E-01	6.67E-01
43	DG	DG(18:1_22:6)	-0.86	(-0.96--0.76)	5.80E-61	1.10E-60	-1.29	(-1.4--1.19)	1.26E-104	1.53E-104	-0.31	(-0.41--0.21)	2.05E-09	2.24E-09	0.26	(0.05-0.47)	1.37E-02	2.06E-02
44	DG	DG(18:2_22:6)	-0.85	(-0.95--0.75)	4.19E-57	7.74E-57	-1.65	(-1.77--1.52)	2.59E-126	3.31E-126	-0.20	(-0.32--0.07)	1.72E-03	1.80E-03	0.50	(0.28-0.73)	9.89E-06	2.40E-05
45	TG	TG(48:0) [NL-18:0]	-1.16	(-1.26--1.05)	4.85E-94	1.14E-93	-1.72	(-1.82--1.63)	6.42E-192	9.26E-192	0.49	(0.38-0.6)	6.90E-18	8.02E-18	-0.38	(-0.63--0.13)	3.09E-03	5.17E-03

46	TG	TG(48:1) [NL-16:1]	-2.28	(-2.38--2.17)	3.16E-238	2.30E-237	-2.74	(-2.86--2.62)	9.15E-264	1.54E-263	-0.12	(-0.24-0)	4.57E-02	4.68E-02	-0.31	(-0.55--0.07)	1.08E-02	1.69E-02
47	TG	TG(48:1) [NL-18:1]	-1.89	(-2--1.79)	1.09E-189	5.70E-189	-3.74	(-3.85--3.63)	0.00E+00	0.00E+00	1.36	(1.23-1.49)	2.02E-82	3.13E-82	-0.22	(-0.49-0.05)	1.09E-01	1.40E-01
48	TG	TG(48:2) [NL-14:0]	-2.11	(-2.22--1.99)	6.65E-202	3.80E-201	-3.96	(-4.08--3.84)	0.00E+00	0.00E+00	1.09	(0.96-1.22)	7.45E-57	1.04E-56	-0.02	(-0.29-0.26)	9.01E-01	9.20E-01
49	TG	TG(48:2) [NL-14:1]	-1.79	(-1.89--1.68)	3.03E-167	1.29E-166	-3.76	(-3.87--3.65)	0.00E+00	0.00E+00	1.55	(1.43-1.67)	5.38E-112	9.38E-112	-0.29	(-0.55--0.03)	2.69E-02	3.87E-02
50	TG	TG(48:2) [NL-16:1]	-1.97	(-2.09--1.85)	4.27E-169	1.90E-168	-2.64	(-2.77--2.51)	3.86E-232	5.99E-232	-0.18	(-0.3--0.06)	3.87E-03	4.02E-03	-0.03	(-0.29-0.23)	8.46E-01	8.78E-01
51	TG	TG(48:2) [NL-18:2]	-2.10	(-2.21--1.98)	8.74E-195	4.66E-194	-4.38	(-4.5--4.25)	0.00E+00	0.00E+00	1.53	(1.39-1.66)	8.23E-92	1.32E-91	-0.02	(-0.3-0.26)	8.85E-01	9.08E-01
52	TG	TG(48:3) [NL-14:0]	-1.70	(-1.81--1.58)	2.52E-143	8.65E-143	-3.45	(-3.57--3.32)	0.00E+00	0.00E+00	0.88	(0.75-1.01)	5.32E-38	6.92E-38	0.13	(-0.14-0.4)	3.52E-01	4.10E-01
53	TG	TG(48:3) [NL-16:1]	-1.54	(-1.64--1.43)	9.23E-139	3.03E-138	-2.38	(-2.49--2.26)	8.38E-238	1.33E-237	0.02	(-0.09-0.12)	7.76E-01	7.78E-01	0.12	(-0.11-0.35)	3.21E-01	3.76E-01
54	TG	TG(48:3) [NL-18:3]	-1.74	(-1.87--1.61)	4.90E-126	1.48E-125	-3.44	(-3.57--3.32)	0.00E+00	0.00E+00	1.15	(1.03-1.27)	8.24E-67	1.21E-66	-0.07	(-0.36-0.23)	6.60E-01	7.12E-01
55	TG	TG(49:1) [NL-17:1]	-1.70	(-1.78--1.62)	3.15E-246	2.52E-245	-2.44	(-2.53--2.35)	0.00E+00	0.00E+00	0.34	(0.26-0.42)	2.24E-15	2.54E-15	-0.18	(-0.35--0.01)	4.29E-02	5.90E-02
56	TG	TG(50:1) [NL-14:0]	-1.11	(-1.2--1.02)	3.27E-103	8.49E-103	-1.95	(-2.05--1.86)	9.66E-233	1.51E-232	0.69	(0.58-0.8)	1.56E-32	1.96E-32	-0.46	(-0.69--0.23)	9.70E-05	2.02E-04
57	TG	TG(50:1) [NL-16:0]	-1.61	(-1.68--1.54)	5.35E-266	5.35E-265	-2.93	(-3.01--2.84)	0.00E+00	0.00E+00	0.96	(0.86-1.06)	5.68E-72	8.54E-72	-0.20	(-0.37--0.02)	2.55E-02	3.68E-02
58	TG	TG(50:1) [NL-18:1]	-1.56	(-1.63--1.49)	3.39E-261	3.12E-260	-2.90	(-2.98--2.81)	0.00E+00	0.00E+00	0.96	(0.86-1.06)	4.43E-73	6.73E-73	-0.16	(-0.33-0.01)	6.96E-02	9.23E-02
59	TG	TG(50:2) [NL-14:0]	-0.96	(-1.04--0.89)	2.77E-114	7.82E-114	-3.12	(-3.22--3.03)	0.00E+00	0.00E+00	1.70	(1.59-1.82)	3.03E-138	5.83E-138	-0.06	(-0.27-0.14)	5.58E-01	6.20E-01
60	TG	TG(50:2) [NL-16:1]	-1.16	(-1.23--1.08)	5.65E-151	2.02E-150	-2.43	(-2.53--2.33)	2.00E-286	3.61E-286	0.60	(0.48-0.71)	3.08E-24	3.73E-24	-0.02	(-0.21-0.16)	8.02E-01	8.37E-01
61	TG	TG(50:2) [NL-18:1]	-1.03	(-1.1--0.96)	2.29E-139	7.63E-139	-2.62	(-2.72--2.52)	0.00E+00	0.00E+00	0.95	(0.83-1.06)	4.65E-54	6.39E-54	-0.01	(-0.2-0.17)	8.88E-01	9.09E-01
62	TG	TG(50:2) [NL-18:2]	-1.50	(-1.56--1.43)	3.11E-262	2.99E-261	-3.51	(-3.62--3.4)	0.00E+00	0.00E+00	1.43	(1.31-1.55)	5.03E-100	8.26E-100	0.00	(-0.17-0.16)	9.74E-01	9.78E-01
63	TG	TG(50:3) [NL-14:0]	-1.30	(-1.38--1.21)	2.74E-144	9.46E-144	-4.10	(-4.21--3.99)	0.00E+00	0.00E+00	2.00	(1.87-2.12)	1.06E-152	2.13E-152	0.17	(-0.06-0.4)	1.40E-01	1.75E-01
64	TG	TG(50:3) [NL-14:1]	-1.01	(-1.08--0.94)	2.14E-126	6.50E-126	-3.12	(-3.21--3.03)	0.00E+00	0.00E+00	1.73	(1.62-1.83)	4.24E-168	9.21E-168	-0.15	(-0.33-0.04)	1.24E-01	1.57E-01
65	TG	TG(50:3) [NL-16:1]	-1.38	(-1.47--1.3)	1.69E-160	6.69E-160	-3.06	(-3.17--2.96)	0.00E+00	0.00E+00	0.63	(0.52-0.75)	1.06E-24	1.29E-24	0.24	(0.03-0.45)	2.82E-02	4.04E-02
66	TG	TG(50:3) [NL-18:2]	-1.28	(-1.36--1.2)	3.48E-157	1.34E-156	-3.60	(-3.71--3.49)	0.00E+00	0.00E+00	1.30	(1.18-1.42)	3.94E-80	6.04E-80	0.26	(0.05-0.47)	1.37E-02	2.06E-02
67	TG	TG(50:3) [NL-18:3]	-1.71	(-1.8--1.61)	8.25E-186	4.08E-185	-3.67	(-3.78--3.56)	0.00E+00	0.00E+00	1.25	(1.13-1.37)	1.73E-77	2.64E-77	-0.01	(-0.24-0.22)	9.25E-01	9.39E-01
68	TG	TG(50:4) [NL-14:0]	-1.29	(-1.39--1.18)	1.02E-111	2.79E-111	-3.88	(-4--3.76)	0.00E+00	0.00E+00	1.70	(1.58-1.82)	3.31E-125	6.15E-125	0.31	(0.07-0.56)	1.11E-02	1.72E-02
69	TG	TG(50:4) [NL-18:3]	-1.15	(-1.25--1.04)	8.31E-92	1.90E-91	-2.94	(-3.05--2.82)	0.00E+00	0.00E+00	0.97	(0.86-1.09)	8.64E-57	1.20E-56	0.14	(-0.09-0.37)	2.43E-01	2.92E-01
70	TG	TG(50:4) [NL-20:4]	-2.14	(-2.27--2)	7.15E-163	2.94E-162	-2.11	(-2.25--1.96)	4.42E-144	5.82E-144	-0.67	(-0.8--0.53)	2.01E-21	2.36E-21	-0.10	(-0.39-0.19)	5.03E-01	5.63E-01
71	TG	TG(51:1) [NL-17:0]	-1.46	(-1.54--1.38)	2.49E-198	1.37E-197	-2.94	(-3.03--2.84)	0.00E+00	0.00E+00	1.14	(1.04-1.23)	4.56E-92	7.34E-92	-0.34	(-0.54--0.15)	5.23E-04	9.77E-04
72	TG	TG(51:2) [NL-15:0]	-0.95	(-1.02--0.88)	1.33E-123	3.88E-123	-3.63	(-3.73--3.54)	0.00E+00	0.00E+00	2.15	(2.04-2.25)	1.25E-208	3.42E-208	0.00	(-0.18-0.18)	9.73E-01	9.78E-01
73	TG	TG(51:2) [NL-17:0]	-1.35	(-1.43--1.27)	3.61E-187	1.81E-186	-3.37	(-3.46--3.27)	0.00E+00	0.00E+00	1.36	(1.25-1.46)	4.38E-110	7.54E-110	-0.04	(-0.23-0.15)	6.64E-01	7.13E-01
74	TG	TG(51:2) [NL-17:1]	-1.28	(-1.35--1.2)	1.75E-188	8.96E-188	-3.00	(-3.09--2.92)	0.00E+00	0.00E+00	1.15	(1.06-1.23)	2.25E-109	3.83E-109	-0.04	(-0.22-0.13)	6.29E-01	6.84E-01
75	TG	TG(52:1) [NL-18:0]	-1.39	(-1.48--1.31)	4.15E-163	1.72E-162	-2.70	(-2.8--2.6)	0.00E+00	0.00E+00	1.06	(0.95-1.17)	7.70E-71	1.16E-70	-0.41	(-0.62--0.21)	1.04E-04	2.16E-04
76	TG	TG(52:1) [NL-18:1]	-1.30	(-1.39--1.22)	4.99E-153	1.84E-152	-2.59	(-2.69--2.49)	0.00E+00	0.00E+00	0.99	(0.89-1.1)	1.12E-64	1.64E-64	-0.39	(-0.59--0.18)	1.88E-04	3.73E-04
77	TG	TG(52:2) [NL-16:0]	-0.62	(-0.67--0.58)	4.69E-125	1.39E-124	-2.67	(-2.74--2.6)	0.00E+00	0.00E+00	1.69	(1.6-1.77)	5.95E-218	1.74E-217	-0.04	(-0.16-0.07)	4.86E-01	5.45E-01
78	TG	TG(52:2) [NL-18:2]	-0.96	(-1.03--0.89)	3.61E-134	1.15E-133	-2.30	(-2.4--2.21)	1.37E-280	2.42E-280	0.96	(0.85-1.07)	8.09E-62	1.17E-61	-0.15	(-0.31-0.02)	8.12E-02	1.06E-01
79	TG	TG(52:3) [NL-16:1]	-0.43	(-0.49--0.37)	4.20E-44	7.12E-44	-2.11	(-2.21--2.02)	9.79E-270	1.70E-269	1.05	(0.95-1.16)	8.71E-76	1.33E-75	0.12	(-0.03-0.27)	1.12E-01	1.43E-01
80	TG	TG(52:3) [NL-18:2]	-0.61	(-0.66--0.56)	6.80E-113	1.90E-112	-3.57	(-3.67--3.48)	0.00E+00	0.00E+00	2.32	(2.21-2.43)	1.39E-227	4.22E-227	0.17	(0.05-0.3)	6.42E-03	1.03E-02
81	TG	TG(52:4) [NL-16:1]	-0.73	(-0.8--0.65)	1.54E-71	3.13E-71	-3.21	(-3.32--3.11)	0.00E+00	0.00E+00	1.44	(1.32-1.56)	2.34E-101	3.87E-101	0.40	(0.21-0.59)	4.13E-05	9.35E-05
82	TG	TG(52:4) [NL-18:2]	-0.92	(-1--0.85)	1.03E-101	2.62E-101	-4.34	(-4.46--4.22)	0.00E+00	0.00E+00	2.37	(2.24-2.49)	3.17E-186	7.68E-186	0.46	(0.26-0.65)	4.94E-06	1.26E-05
83	TG	TG(52:4) [NL-18:3]	-0.87	(-0.94--0.79)	5.45E-92	1.25E-91	-3.90	(-4.01--3.8)	0.00E+00	0.00E+00	2.27	(2.16-2.38)	3.12E-210	8.71E-210	0.22	(0.02-0.41)	2.86E-02	4.06E-02
84	TG	TG(52:5) [NL-18:3]	-0.91	(-1.01--0.82)	3.44E-72	7.06E-72	-3.82	(-3.93--3.7)	0.00E+00	0.00E+00	1.98	(1.87-2.09)	1.89E-175	4.36E-175	0.36	(0.14-0.57)	9.94E-04	1.78E-03
85	TG	TG(52:5) [NL-20:4]	-1.62	(-1.73--1.51)	9.17E-145	3.21E-144	-1.32	(-1.45--1.19)	1.59E-78	1.86E-78	-1.20	(-1.32--1.07)	9.50E-69	1.41E-68	0.10	(-0.13-0.33)	3.98E-01	4.56E-01
86	TG	TG(52:5) [NL-20:5]	-1.24	(-1.36--1.12)	1.42E-83	3.08E-83	-2.16	(-2.3--2.03)	2.22E-165	3.08E-165	-0.07	(-0.2-0.05)	2.39E-01	2.42E-01	0.37	(0.13-0.61)	2.59E-03	4.40E-03
87	TG	TG(53:2) [NL-18:1]	-0.75	(-0.81--0.68)	2.02E-94	4.82E-94	-2.82	(-2.9--2.74)	0.00E+00	0.00E+00	1.76	(1.67-1.84)	4.64E-217	1.35E-216	-0.19	(-0.34--0.03)	1.76E-02	2.58E-02
88	TG	TG(54:1) [NL-18:1]	-0.87	(-0.94--0.8)	4.70E-110	1.26E-109	-2.47	(-2.56--2.38)	0.00E+00	0.00E+00	1.40	(1.3-1.5)	4.13E-123	7.59E-123	-0.38	(-0.55--0.22)	5.29E-06	1.34E-05
89	TG	TG(54:2) [NL-18:0]	-0.72	(-0.79--0.65)	2.01E-78	4.24E-78	-2.46	(-2.55--2.37)	0.00E+00	0.00E+00	1.42	(1.33-1.52)	5.62E-137	1.07E-136	-0.26	(-0.42--0.1)	1.84E-03	3.18E-03
90	TG	TG(54:2) [NL-20:1]	-1.83	(-1.9--1.75)	7.34E-294	9.78E-293	-4.08	(-4.17--3.98)	0.00E+00	0.00E+00	2.05	(1.96-2.15)	1.80E-239	5.82E-239	-0.19	(-0.36--0.02)	2.46E-02	3.56E-02
91	TG	TG(54:3) [NL-18:1]	-0.22	(-0.28--0.17)	1.53E-16	2.07E-16	-2.16	(-2.24--2.09)	0.00E+00	0.00E+00	1.71	(1.63-1.79)	9.12E-223	2.72E-222	-0.08	(-0.2-0.04)	1.82E-01	2.23E-01
92	TG	TG(54:3) [NL-18:2]	-0.37	(-0.42--0.31)	1.28E-37	2.05E-37	-2.52	(-2.61--2.43)	0.00E+00	0.00E+00	1.66	(1.57-1.75)	1.31E-187	3.18E-187	0.05	(-0.07-0.18)	4.00E-01	4.57E-01
93	TG	TG(54:4) [NL-18:2]	-0.23	(-0.28--0.17)	1.06E-13	1.39E-13	-3.24	(-3.34--3.14)	0.00E+00	0.00E+00	2.50	(2.39-2.61)	7.34E-246	2.43E-245	0.14	(0-0.28)	4.84E-02	6.58E-02
94	TG	TG(54:4) [NL-20:3]	-1.19	(-1.26--1.12)	1.34E-176	6.24E-176	-1.65	(-1.75--1.55)	5.80E-181	8.16E-181	0.12	(0.02-0.23)	1.83E-02	1.89E-02	-0.22	(-0.39--0.06)	7.93E-03	1.25E-02
95	TG	TG(54:5) [NL-18:3]	-0.47	(-0.55--0.39)	5.20E-28	7.73E-28	-3.63	(-3.74--3.53)	0.00E+00	0.00E+00	2.49	(2.39-2.6)	9.16E-255	3.14E-254	0.23	(0.04-0.43)	1.91E-02	2.80E-02
96	TG	TG(54:5) [NL-20:4]	-0.90	(-0.96--0.83)	4.74E-125	1.40E-124	-1.71	(-1.82--1.61)	1.31E-166	1.82E-166	0.08	(-0.03-0.2)	1.42E-01	1.44E-01	0.16	(0.01-0.32)	4.03E-02	5.59E-02
97	TG	TG(54:6) [NL-18:3]	-0.59	(-0.69--0.48)	6.58E-28	9.71E-28	-3.83	(-3.95--3.71)	0.00E+00	0.00E+00	2.47	(2.35-2.58)	1.04E-238	3.32E-238	0.41	(0.19-0.63)	3.19E-04	6.08E-04

98	TG	TG(54:6) [NL-20:4]	-1.15	(-1.23--1.07)	**1.09E-137**	**3.53E-137**	-2.10	(-2.21--1.99)	**4.80E-200**	**7.05E-200**	-0.10	(-0.22-0.02)	1.02E-01	1.04E-01	0.39	(0.2-0.57)	**3.97E-05**	**9.06E-05**
99	TG	TG(54:6) [NL-20:5]	-0.48	(-0.59--0.38)	**2.65E-18**	**3.64E-18**	-2.43	(-2.56--2.3)	**2.70E-200**	**3.98E-200**	0.75	(0.61-0.88)	**4.18E-27**	**5.15E-27**	0.63	(0.4-0.87)	**1.71E-07**	**5.19E-07**
100	TG	TG(54:6) [NL-22:6]	-2.12	(-2.24--2.01)	**3.98E-195**	**2.14E-194**	-1.67	(-1.81--1.53)	**4.29E-104**	**5.21E-104**	-1.47	(-1.61--1.34)	**2.01E-82**	**3.12E-82**	0.19	(-0.07-0.44)	1.58E-01	1.96E-01
101	TG	TG(54:7) [NL-20:5]	-0.45	(-0.56--0.34)	**1.76E-14**	**2.32E-14**	-2.18	(-2.32--2.04)	**1.20E-158**	**1.64E-158**	0.45	(0.32-0.58)	**1.48E-11**	**1.64E-11**	0.75	(0.52-0.99)	**3.39E-10**	**1.44E-09**
102	TG	TG(54:7) [NL-22:6]	-1.56	(-1.68--1.44)	**4.06E-115**	**1.16E-114**	-0.77	(-0.9--0.63)	**8.71E-27**	**9.61E-27**	-2.04	(-2.17--1.91)	**4.94E-148**	**9.75E-148**	0.38	(0.12-0.63)	**4.50E-03**	**7.34E-03**
103	TG	TG(56:6) [NL-20:4]	0.06	(0.01-0.1)	**1.70E-02**	**1.84E-02**	-1.21	(-1.3--1.12)	**3.91E-123**	**4.95E-123**	0.60	(0.5-0.7)	**2.47E-31**	**3.08E-31**	0.30	(0.2-0.41)	**3.95E-08**	**1.30E-07**
104	TG	TG(56:6) [NL-22:5]	-0.62	(-0.68--0.55)	**2.10E-71**	**4.26E-71**	-1.12	(-1.22--1.02)	**1.99E-94**	**2.37E-94**	-0.18	(-0.28--0.08)	**6.22E-04**	**6.52E-04**	0.20	(0.05-0.35)	**8.38E-03**	**1.32E-02**
105	TG	TG(56:7) [NL-20:4]	-0.52	(-0.58--0.46)	**4.34E-55**	**7.92E-55**	-1.80	(-1.9--1.69)	**1.20E-184**	**1.70E-184**	0.48	(0.37-0.59)	**1.36E-16**	**1.57E-16**	0.36	(0.21-0.51)	**2.24E-06**	**6.10E-06**
106	TG	TG(56:7) [NL-20:5]	0.07	(-0.03-0.16)	1.94E-01	2.04E-01	-2.09	(-2.21--1.97)	**3.52E-187**	**4.98E-187**	1.10	(0.99-1.22)	**2.15E-69**	**3.21E-69**	0.60	(0.39-0.81)	**2.23E-08**	**7.54E-08**
107	TG	TG(56:7) [NL-22:5]	-0.96	(-1.03--0.88)	**1.14E-112**	**3.13E-112**	-1.67	(-1.77--1.56)	**1.51E-162**	**2.08E-162**	-0.33	(-0.44--0.23)	**2.74E-09**	**2.98E-09**	0.42	(0.25-0.6)	**3.69E-06**	**9.63E-06**
108	TG	TG(56:7) [NL-22:6]	-1.23	(-1.33--1.12)	**6.53E-98**	**1.59E-97**	-1.74	(-1.87--1.61)	**6.82E-125**	**8.67E-125**	-0.67	(-0.8--0.54)	**1.07E-22**	**1.27E-22**	0.48	(0.24-0.72)	**1.03E-04**	**2.14E-04**
109	TG	TG(56:8) [NL-20:4]	-0.67	(-0.75--0.59)	**1.09E-55**	**2.01E-55**	-1.15	(-1.26--1.05)	**1.40E-85**	**1.64E-85**	-0.25	(-0.36--0.15)	**3.06E-06**	**3.25E-06**	0.39	(0.22-0.56)	**5.86E-06**	**1.46E-05**
110	TG	TG(56:8) [NL-20:5]	-0.09	(-0.19-0.02)	1.17E-01	1.24E-01	-1.97	(-2.1--1.84)	**2.42E-155**	**3.27E-155**	0.83	(0.72-0.95)	**5.75E-42**	**7.59E-42**	0.75	(0.53-0.96)	**2.50E-11**	**1.24E-10**
111	TG	TG(56:8) [NL-22:6]	-1.33	(-1.44--1.22)	**7.02E-102**	**1.81E-101**	-1.72	(-1.86--1.59)	**3.34E-112**	**4.12E-112**	-0.99	(-1.12--0.86)	**6.66E-44**	**8.83E-44**	0.65	(0.4-0.89)	**2.57E-07**	**7.62E-07**
112	TG	TG(56:9) [NL-22:6]	-0.97	(-1.08--0.87)	**2.50E-66**	**4.90E-66**	-1.05	(-1.18--0.92)	**2.66E-53**	**3.07E-53**	-1.17	(-1.3--1.05)	**1.98E-67**	**2.91E-67**	0.63	(0.4-0.85)	**4.93E-08**	**1.60E-07**
113	TG	TG(58:10) [NL-22:6]	-0.83	(-0.93--0.72)	**2.99E-51**	**5.31E-51**	-0.92	(-1.05--0.8)	**7.73E-45**	**8.79E-45**	-1.05	(-1.16--0.93)	**5.09E-59**	**7.28E-59**	0.66	(0.44-0.88)	**6.07E-09**	**2.19E-08**
114	TG	TG(58:8) [NL-22:6]	-0.89	(-0.99--0.79)	**2.64E-60**	**4.96E-60**	-1.18	(-1.3--1.06)	**2.46E-73**	**2.86E-73**	-0.82	(-0.94--0.7)	**7.08E-37**	**9.16E-37**	0.53	(0.3-0.76)	**6.96E-06**	**1.71E-05**
115	TG	TG(58:9) [NL-22:6]	-0.96	(-1.06--0.85)	**1.92E-66**	**3.77E-66**	-1.30	(-1.43--1.18)	**1.16E-81**	**1.37E-81**	-0.84	(-0.97--0.72)	**1.77E-38**	**2.31E-38**	0.67	(0.44-0.89)	**1.34E-08**	**4.78E-08**
116	TG(O)	TG(O-50:1) [NL-16:0]	-0.61	(-0.67--0.55)	**2.41E-77**	**5.02E-77**	-3.21	(-3.28--3.14)	**0.00E+00**	**0.00E+00**	2.64	(2.57-2.72)	**0.00E+00**	**0.00E+00**	-0.11	(-0.26-0.04)	1.38E-01	1.73E-01
117	TG(O)	TG(O-50:1) [NL-17:1]	-1.72	(-1.8--1.64)	**3.59E-246**	**2.83E-245**	-2.48	(-2.57--2.39)	**0.00E+00**	**0.00E+00**	0.35	(0.26-0.43)	**3.29E-15**	**3.72E-15**	-0.17	(-0.35-0)	**4.92E-02**	6.65E-02
118	TG(O)	TG(O-50:1) [NL-18:1]	-0.49	(-0.56--0.42)	**1.36E-40**	**2.23E-40**	-3.35	(-3.43--3.28)	**0.00E+00**	**0.00E+00**	2.83	(2.75-2.91)	**0.00E+00**	**0.00E+00**	0.04	(-0.13-0.21)	6.64E-01	7.13E-01
119	TG(O)	TG(O-50:2) [NL-18:2]	-0.54	(-0.6--0.48)	**3.60E-66**	**6.99E-66**	-2.67	(-2.74--2.6)	**0.00E+00**	**0.00E+00**	2.02	(1.94-2.09)	**8.53E-306**	**3.94E-305**	-0.03	(-0.17-0.11)	7.10E-01	7.54E-01
120	TG(O)	TG(O-52:2) [NL-16:0]	-0.77	(-0.85--0.7)	**1.30E-74**	**2.69E-74**	-3.26	(-3.35--3.16)	**0.00E+00**	**0.00E+00**	2.45	(2.35-2.55)	**5.33E-271**	**2.03E-270**	-0.31	(-0.49--0.12)	**1.04E-03**	**1.85E-03**
121	TG(O)	TG(O-52:2) [NL-17:1]	-1.28	(-1.35--1.21)	**6.56E-188**	**3.31E-187**	-3.03	(-3.11--2.94)	**0.00E+00**	**0.00E+00**	1.16	(1.07-1.25)	**1.93E-109**	**3.30E-109**	-0.05	(-0.22-0.13)	6.09E-01	6.67E-01
122	TG(O)	TG(O-52:2) [NL-18:1]	-0.68	(-0.76--0.6)	**6.82E-55**	**1.24E-54**	-3.18	(-3.28--3.08)	**0.00E+00**	**0.00E+00**	2.49	(2.38-2.59)	**4.19E-259**	**1.46E-258**	-0.32	(-0.51--0.12)	**1.47E-03**	**2.56E-03**
123	TG(O)	TG(O-54:2) [NL-18:1]	-0.74	(-0.81--0.68)	**3.63E-94**	**8.57E-94**	-2.82	(-2.9--2.74)	**0.00E+00**	**0.00E+00**	1.75	(1.67-1.84)	**4.23E-216**	**1.21E-215**	-0.18	(-0.33--0.02)	**2.40E-02**	**3.48E-02**
124	TG(O)	TG(O-54:4) [NL-17:1]	-0.65	(-0.73--0.58)	**1.71E-61**	**3.24E-61**	-3.35	(-3.45--3.24)	**0.00E+00**	**0.00E+00**	1.80	(1.69-1.91)	**7.38E-160**	**1.54E-159**	0.35	(0.18-0.52)	**8.08E-05**	**1.72E-04**
125	TG(O)	TG(O-54:4) [NL-18:2]	-0.62	(-0.7--0.54)	**1.01E-50**	**1.80E-50**	-3.63	(-3.74--3.51)	**0.00E+00**	**0.00E+00**	2.14	(2.02-2.26)	**2.50E-183**	**5.97E-183**	0.38	(0.2-0.57)	**5.90E-05**	**1.27E-04**
126	dhCer	dhCer(d18:0/22:0)	-0.05	(-0.11-0)	6.91E-02	7.36E-02	0.09	(0.03-0.16)	**4.40E-03**	**4.57E-03**	-0.25	(-0.32--0.19)	**1.04E-14**	**1.17E-14**	-0.10	(-0.22-0.02)	1.07E-01	1.38E-01
127	dhCer	dhCer(d18:0/24:0)	0.02	(-0.03-0.08)	3.88E-01	4.03E-01	0.46	(0.39-0.52)	**1.07E-41**	**1.21E-41**	-0.50	(-0.57--0.44)	**3.45E-50**	**4.65E-50**	-0.04	(-0.15-0.07)	4.72E-01	5.30E-01
128	dhCer	dhCer(d18:0/24:1)	-0.36	(-0.42--0.3)	**4.60E-29**	**6.89E-29**	0.03	(-0.04-0.11)	3.97E-01	3.98E-01	-0.67	(-0.75--0.6)	**4.21E-57**	**5.91E-57**	0.10	(-0.03-0.24)	1.44E-01	1.79E-01
129	Cer	Cer(d16:1/22:0)	0.07	(0.02-0.13)	**9.09E-03**	**9.93E-03**	-2.36	(-2.43--2.29)	**0.00E+00**	**0.00E+00**	2.22	(2.15-2.3)	**0.00E+00**	**0.00E+00**	0.00	(-0.12-0.13)	9.71E-01	9.78E-01
130	Cer	Cer(d16:1/23:0)	0.12	(0.06-0.18)	**1.00E-04**	**1.16E-04**	-3.18	(-3.28--3.08)	**0.00E+00**	**0.00E+00**	3.23	(3.11-3.34)	**0.00E+00**	**0.00E+00**	-0.14	(-0.27-0)	5.21E-02	7.00E-02
131	Cer	Cer(d16:1/24:0)	0.31	(0.26-0.37)	**1.57E-26**	**2.28E-26**	-2.66	(-2.73--2.59)	**0.00E+00**	**0.00E+00**	2.85	(2.77-2.92)	**0.00E+00**	**0.00E+00**	0.07	(-0.06-0.2)	2.79E-01	3.31E-01
132	Cer	Cer(d16:1/24:1)	0.17	(0.12-0.23)	**1.86E-09**	**2.33E-09**	-2.07	(-2.15--2)	**0.00E+00**	**0.00E+00**	2.01	(1.93-2.09)	**1.53E-273**	**5.98E-273**	0.13	(0.01-0.26)	**3.09E-02**	**4.35E-02**
133	Cer	Cer(d17:1/22:0)	0.24	(0.18-0.3)	**1.03E-15**	**1.38E-15**	-1.87	(-1.95--1.79)	**4.56E-264**	**7.70E-264**	1.97	(1.88-2.05)	**1.61E-248**	**5.37E-248**	-0.03	(-0.15-0.1)	7.02E-01	7.48E-01
134	Cer	Cer(d17:1/23:0)	0.23	(0.17-0.29)	**1.49E-13**	**1.95E-13**	-3.32	(-3.44--3.21)	**0.00E+00**	**0.00E+00**	3.42	(3.29-3.55)	**1.20E-292**	**4.96E-292**	-0.04	(-0.18-0.1)	5.93E-01	6.55E-01
135	Cer	Cer(d17:1/24:0)	0.43	(0.38-0.48)	**2.44E-55**	**4.47E-55**	-2.04	(-2.11--1.97)	**0.00E+00**	**0.00E+00**	2.40	(2.32-2.47)	**0.00E+00**	**0.00E+00**	0.04	(-0.08-0.16)	4.69E-01	5.28E-01
136	Cer	Cer(d17:1/24:1)	0.26	(0.2-0.31)	**2.71E-20**	**3.77E-20**	-2.20	(-2.27--2.12)	**0.00E+00**	**0.00E+00**	2.20	(2.12-2.28)	**2.97E-300**	**1.31E-299**	0.22	(0.1-0.34)	**2.78E-04**	**5.39E-04**
137	Cer	Cer(d18:1/16:0)	-0.27	(-0.31--0.23)	**5.49E-37**	**8.73E-37**	-2.08	(-2.14--2.03)	**0.00E+00**	**0.00E+00**	1.61	(1.56-1.67)	**0.00E+00**	**0.00E+00**	0.22	(0.13-0.31)	**9.07E-07**	**2.61E-06**
138	Cer	Cer(d18:1/18:0)	-0.13	(-0.19--0.08)	**4.60E-06**	**5.51E-06**	-1.62	(-1.69--1.55)	**4.59E-280**	**8.10E-280**	1.25	(1.19-1.32)	**1.01E-194**	**2.54E-194**	-0.03	(-0.15-0.09)	6.36E-01	6.91E-01
139	Cer	Cer(d18:1/20:0)	-0.19	(-0.24--0.14)	**1.44E-14**	**1.91E-14**	-2.05	(-2.11--1.99)	**0.00E+00**	**0.00E+00**	1.53	(1.47-1.59)	**2.78E-274**	**1.09E-273**	0.21	(0.11-0.31)	**8.77E-05**	**1.84E-04**
140	Cer	Cer(d18:1/22:0)	0.14	(0.1-0.18)	**1.56E-10**	**1.97E-10**	-2.24	(-2.29--2.19)	**0.00E+00**	**0.00E+00**	2.13	(2.08-2.19)	**0.00E+00**	**0.00E+00**	0.11	(0.02-0.21)	**1.94E-02**	**2.83E-02**
141	Cer	Cer(d18:1/23:0)	0.08	(0.04-0.12)	**3.09E-04**	**3.54E-04**	-3.73	(-3.8--3.67)	**0.00E+00**	**0.00E+00**	3.69	(3.62-3.76)	**0.00E+00**	**0.00E+00**	0.05	(-0.05-0.15)	3.23E-01	3.78E-01
142	Cer	Cer(d18:1/24:0)	0.24	(0.2-0.28)	**5.48E-28**	**8.12E-28**	-1.97	(-2.03--1.92)	**0.00E+00**	**0.00E+00**	2.05	(1.99-2.1)	**0.00E+00**	**0.00E+00**	0.13	(0.04-0.22)	**4.54E-03**	**7.38E-03**
143	Cer	Cer(d18:1/24:1)	0.01	(-0.04-0.05)	7.40E-01	7.46E-01	-2.23	(-2.29--2.16)	**0.00E+00**	**0.00E+00**	1.91	(1.85-1.97)	**0.00E+00**	**0.00E+00**	0.32	(0.23-0.42)	**9.38E-11**	**4.37E-10**
144	Cer	Cer(d18:2/16:0)	-0.39	(-0.44--0.33)	**3.09E-40**	**5.00E-40**	-2.21	(-2.28--2.13)	**0.00E+00**	**0.00E+00**	1.32	(1.24-1.4)	**2.55E-157**	**5.23E-157**	0.44	(0.32-0.56)	**1.64E-12**	**9.61E-12**
145	Cer	Cer(d18:2/22:0)	0.43	(0.38-0.48)	**3.15E-57**	**5.85E-57**	-1.43	(-1.5--1.37)	**1.19E-258**	**1.97E-258**	1.50	(1.44-1.57)	**2.28E-250**	**7.67E-250**	0.32	(0.21-0.44)	**1.49E-08**	**5.27E-08**
146	Cer	Cer(d18:2/23:0)	0.36	(0.3-0.41)	**1.73E-38**	**2.77E-38**	-3.30	(-3.39--3.22)	**0.00E+00**	**0.00E+00**	3.43	(3.35-3.52)	**0.00E+00**	**0.00E+00**	0.23	(0.12-0.35)	**7.37E-05**	**1.58E-04**
147	Cer	Cer(d18:2/24:0)	0.52	(0.47-0.57)	**2.50E-84**	**5.50E-84**	-1.73	(-1.79--1.66)	**0.00E+00**	**0.00E+00**	1.94	(1.88-2.01)	**0.00E+00**	**0.00E+00**	0.39	(0.29-0.5)	**9.10E-13**	**5.39E-12**
148	Cer	Cer(d18:2/24:1)	0.39	(0.34-0.44)	**2.50E-48**	**4.37E-48**	-1.50	(-1.57--1.43)	**6.62E-264**	**1.12E-263**	1.48	(1.41-1.55)	**3.19E-234**	**1.00E-233**	0.48	(0.37-0.59)	**1.05E-17**	**1.25E-16**
149	Cer	Cer(d19:1/24:0)	0.72	(0.65-0.8)	**3.91E-66**	**7.56E-66**	-0.95	(-1.05--0.86)	**8.83E-81**	**1.03E-80**	1.78	(1.69-1.87)	**6.50E-197**	**1.67E-196**	-0.38	(-0.56--0.21)	**2.07E-05**	**4.91E-05**

150	Cer	Cer(d19:1/24:1)	0.47	(0.38-0.56)	**1.28E-24**	**1.85E-24**	-1.30	(-1.41--1.19)	**2.73E-102**	**3.30E-102**	1.68	(1.57-1.79)	**4.24E-145**	**8.28E-145**	-0.20	(-0.4--0.01)	**4.33E-02**	5.93E-02
151	DeoxyCer	Cer(m18:0/22:0)	-0.90	(-0.98--0.83)	**7.50E-114**	**2.10E-113**	-2.36	(-2.45--2.27)	**0.00E+00**	**0.00E+00**	1.25	(1.16-1.34)	**4.66E-126**	**8.71E-126**	-0.19	(-0.33--0.04)	**1.23E-02**	**1.90E-02**
152	DeoxyCer	Cer(m18:0/23:0)	-0.93	(-1.01--0.85)	**1.54E-101**	**3.90E-101**	-4.22	(-4.37--4.07)	**0.00E+00**	**0.00E+00**	3.13	(2.97-3.29)	**6.84E-196**	**1.74E-195**	-0.26	(-0.42--0.09)	**2.43E-03**	**4.16E-03**
153	DeoxyCer	Cer(m18:0/24:0)	-0.72	(-0.79--0.65)	**1.37E-83**	**3.00E-83**	-2.29	(-2.37--2.2)	**0.00E+00**	**0.00E+00**	1.37	(1.29-1.44)	**1.48E-183**	**3.56E-183**	-0.09	(-0.22-0.05)	2.08E-01	2.53E-01
154	DeoxyCer	Cer(m18:0/24:1)	-0.98	(-1.05--0.9)	**9.17E-115**	**2.60E-114**	-3.03	(-3.13--2.93)	**0.00E+00**	**0.00E+00**	1.62	(1.52-1.72)	**5.41E-153**	**1.09E-152**	0.02	(-0.14-0.17)	8.10E-01	8.44E-01
155	DeoxyCer	Cer(m18:1/22:0)	-0.53	(-0.6--0.45)	**1.94E-40**	**3.16E-40**	-1.47	(-1.56--1.38)	**4.62E-176**	**6.44E-176**	0.28	(0.2-0.36)	**3.46E-11**	**3.80E-11**	0.24	(0.08-0.4)	**3.74E-03**	**6.18E-03**
156	DeoxyCer	Cer(m18:1/23:0)	-0.55	(-0.63--0.47)	**1.50E-38**	**2.42E-38**	-3.46	(-3.61--3.32)	**8.74E-276**	**1.52E-275**	2.28	(2.12-2.44)	**4.73E-131**	**8.91E-131**	0.16	(-0.02-0.33)	7.72E-02	1.01E-01
157	DeoxyCer	Cer(m18:1/24:0)	-0.33	(-0.4--0.26)	**1.09E-20**	**1.52E-20**	-1.31	(-1.4--1.23)	**1.39E-159**	**1.90E-159**	0.42	(0.34-0.5)	**5.98E-24**	**7.23E-24**	0.28	(0.13-0.42)	**1.93E-04**	**3.82E-04**
158	DeoxyCer	Cer(m18:1/24:1)	-0.56	(-0.63--0.48)	**1.31E-43**	**2.19E-43**	-2.06	(-2.15--1.97)	**9.31E-256**	**1.52E-255**	0.77	(0.68-0.86)	**8.58E-57**	**1.20E-56**	0.35	(0.2-0.51)	**1.17E-05**	**2.80E-05**
159	GM3	GM3(d18:1/18:0)	0.49	(0.44-0.53)	**6.86E-81**	**1.46E-80**	0.65	(0.59-0.71)	**1.05E-89**	**1.24E-89**	-0.13	(-0.18--0.07)	**1.76E-05**	**1.86E-05**	0.07	(-0.02-0.17)	1.40E-01	1.75E-01
160	GM3	GM3(d18:1/22:0)	0.08	(0.02-0.13)	**5.33E-03**	**5.90E-03**	-0.95	(-1.03--0.87)	**6.35E-100**	**7.66E-100**	0.90	(0.82-0.99)	**9.00E-88**	**1.43E-87**	0.15	(0.04-0.27)	**6.89E-03**	**1.11E-02**
161	GM3	GM3(d18:1/24:0)	0.52	(0.47-0.57)	**6.00E-72**	**1.23E-71**	0.19	(0.11-0.26)	**4.75E-07**	**5.04E-07**	0.34	(0.27-0.41)	**1.63E-21**	**1.92E-21**	0.27	(0.16-0.37)	**6.14E-07**	**1.79E-06**
162	GM3	GM3(d18:1/24:1)	0.03	(-0.02-0.08)	2.76E-01	2.89E-01	-0.49	(-0.56--0.42)	**3.80E-39**	**4.27E-39**	0.37	(0.3-0.44)	**1.11E-23**	**1.34E-23**	0.39	(0.28-0.5)	**1.05E-11**	**5.56E-11**
163	HexCer	HexCer(d16:1/22:0)	0.05	(-0.01-0.11)	7.71E-02	8.17E-02	-2.22	(-2.32--2.11)	**2.12E-235**	**3.35E-235**	2.03	(1.92-2.15)	**8.46E-174**	**1.93E-173**	0.22	(0.09-0.36)	**8.73E-04**	**1.59E-03**
164	HexCer	HexCer(d16:1/24:0)	0.37	(0.31-0.43)	**1.78E-32**	**2.74E-32**	-1.70	(-1.8--1.6)	**1.22E-179**	**1.71E-179**	1.78	(1.67-1.88)	**4.70E-164**	**1.00E-163**	0.31	(0.18-0.45)	**3.55E-06**	**9.37E-06**
165	HexCer	HexCer(d18:1/16:0)	0.08	(0.04-0.12)	**1.81E-04**	**2.08E-04**	-1.70	(-1.77--1.64)	**3.55E-307**	**6.65E-307**	1.85	(1.78-1.92)	**1.80E-298**	**7.79E-298**	0.00	(-0.09-0.09)	9.84E-01	9.86E-01
166	HexCer	HexCer(d18:1/18:0)	-0.14	(-0.19--0.08)	**1.50E-06**	**1.81E-06**	-1.30	(-1.38--1.21)	**2.43E-158**	**3.30E-158**	0.95	(0.87-1.04)	**2.01E-88**	**3.21E-88**	0.42	(0.29-0.54)	**1.18E-10**	**5.31E-10**
167	HexCer	HexCer(d18:1/20:0)	-0.10	(-0.15--0.05)	**1.03E-04**	**1.19E-04**	-1.39	(-1.46--1.31)	**4.54E-191**	**6.52E-191**	1.11	(1.03-1.19)	**1.54E-124**	**2.85E-124**	0.34	(0.24-0.45)	**5.96E-10**	**2.51E-09**
168	HexCer	HexCer(d18:1/22:0)	0.11	(0.07-0.16)	**8.86E-07**	**1.07E-06**	-1.81	(-1.87--1.75)	**0.00E+00**	**0.00E+00**	1.74	(1.68-1.8)	**4.88E-308**	**2.34E-307**	0.35	(0.25-0.45)	**7.19E-12**	**3.88E-11**
169	HexCer	HexCer(d18:1/24:0)	0.25	(0.21-0.3)	**6.16E-27**	**8.95E-27**	-0.77	(-0.83--0.7)	**8.61E-103**	**1.04E-102**	0.87	(0.81-0.94)	**1.45E-121**	**2.63E-121**	0.37	(0.28-0.47)	**1.77E-13**	**1.15E-12**
170	HexCer	HexCer(d18:1/24:1)	0.02	(-0.03-0.07)	4.82E-01	4.98E-01	-1.22	(-1.29--1.16)	**1.08E-204**	**1.59E-204**	1.20	(1.14-1.27)	**1.59E-190**	**3.94E-190**	0.25	(0.15-0.36)	**3.03E-06**	**8.12E-06**
171	Hex2Cer	Hex2Cer(d16:1/16:0)	0.00	(-0.05-0.06)	9.37E-01	9.37E-01	-3.13	(-3.22--3.04)	**0.00E+00**	**0.00E+00**	2.58	(2.48-2.67)	**1.95E-298**	**8.35E-298**	0.42	(0.29-0.55)	**1.87E-10**	**8.15E-10**
172	Hex2Cer	Hex2Cer(d18:1/16:0)	-0.14	(-0.19--0.09)	**1.01E-08**	**1.24E-08**	-2.31	(-2.37--2.25)	**0.00E+00**	**0.00E+00**	1.81	(1.75-1.88)	**3.41E-302**	**1.53E-301**	0.53	(0.42-0.64)	**4.02E-20**	**6.23E-19**
173	Hex2Cer	Hex2Cer(d18:1/22:0)	0.22	(0.17-0.27)	**3.90E-17**	**5.34E-17**	-1.09	(-1.17--1.02)	**5.16E-148**	**6.86E-148**	1.01	(0.94-1.09)	**5.50E-117**	**9.77E-117**	0.46	(0.34-0.57)	**6.11E-15**	**4.65E-14**
174	Hex2Cer	Hex2Cer(d18:1/24:1)	0.34	(0.28-0.39)	**5.63E-33**	**8.68E-33**	-1.57	(-1.64--1.51)	**7.23E-265**	**1.23E-264**	1.73	(1.66-1.81)	**3.65E-264**	**1.33E-263**	0.47	(0.35-0.59)	**2.56E-14**	**1.89E-13**
175	Hex2Cer	Hex2Cer(d18:2/16:0)	-0.01	(-0.06-0.03)	5.56E-01	5.67E-01	-1.93	(-2--1.86)	**0.00E+00**	**0.00E+00**	1.38	(1.31-1.45)	**9.58E-196**	**2.42E-195**	0.57	(0.46-0.67)	**1.81E-23**	**4.34E-22**
176	Hex3Cer	Hex3Cer(d18:1/16:0)	0.02	(-0.02-0.05)	3.68E-01	3.83E-01	-1.06	(-1.11--1.01)	**2.03E-233**	**3.19E-233**	1.03	(0.98-1.08)	**9.21E-206**	**2.50E-205**	0.12	(0.04-0.2)	**2.79E-03**	**4.72E-03**
177	Hex3Cer	Hex3Cer(d18:1/22:0)	0.39	(0.34-0.45)	**2.67E-43**	**4.46E-43**	-1.16	(-1.24--1.08)	**4.62E-145**	**6.09E-145**	1.72	(1.64-1.8)	**1.19E-231**	**3.68E-231**	0.05	(-0.06-0.16)	3.84E-01	4.44E-01
178	Hex3Cer	Hex3Cer(d18:1/24:0)	0.54	(0.49-0.59)	**1.56E-85**	**3.46E-85**	-0.05	(-0.12-0.02)	1.88E-01	1.90E-01	0.74	(0.67-0.81)	**2.44E-83**	**3.80E-83**	0.07	(-0.04-0.17)	2.02E-01	2.46E-01
179	Hex3Cer	Hex3Cer(d18:1/24:1)	0.35	(0.3-0.4)	**8.59E-44**	**1.45E-43**	-0.91	(-0.98--0.85)	**2.62E-127**	**3.37E-127**	1.27	(1.2-1.34)	**2.34E-192**	**5.82E-192**	0.15	(0.05-0.25)	**2.46E-03**	**4.21E-03**
180	SM	SM(d17:1/14:0)	-0.16	(-0.22--0.11)	**2.52E-10**	**3.17E-10**	-1.62	(-1.68--1.57)	**0.00E+00**	**0.00E+00**	1.16	(1.1-1.22)	**5.58E-201**	**1.48E-200**	0.20	(0.08-0.32)	**8.78E-04**	**1.59E-03**
181	SM	SM(d18:0/14:0)	-0.23	(-0.28--0.18)	**2.36E-19**	**3.26E-19**	0.10	(0.05-0.15)	**1.72E-04**	**1.80E-04**	-0.43	(-0.48--0.38)	**1.69E-52**	**2.31E-52**	-0.02	(-0.13-0.09)	7.36E-01	7.75E-01
182	SM	SM(d18:1/14:0)/SM(d16:1/16:0)	-0.20	(-0.24--0.16)	**1.15E-19**	**1.59E-19**	-1.88	(-1.93--1.83)	**0.00E+00**	**0.00E+00**	1.43	(1.37-1.48)	**1.51E-307**	**7.09E-307**	0.12	(0.02-0.21)	**1.58E-02**	**2.34E-02**
183	SM	SM(d18:2/14:0)	-0.18	(-0.22--0.13)	**3.78E-14**	**4.97E-14**	-1.05	(-1.1--1)	**6.38E-229**	**9.85E-229**	0.33	(0.27-0.38)	**2.50E-32**	**3.12E-32**	0.26	(0.16-0.35)	**1.85E-07**	**5.56E-07**
184	SM	SM(d17:1/16:0)	-0.14	(-0.18--0.1)	**2.73E-11**	**3.49E-11**	-1.88	(-1.93--1.83)	**0.00E+00**	**0.00E+00**	1.52	(1.47-1.57)	**0.00E+00**	**0.00E+00**	0.20	(0.1-0.29)	**4.19E-05**	**9.45E-05**
185	SM	SM(d18:1/16:0)	-0.32	(-0.37--0.27)	**1.83E-37**	**2.92E-37**	-2.31	(-2.36--2.25)	**0.00E+00**	**0.00E+00**	1.70	(1.65-1.76)	**0.00E+00**	**0.00E+00**	0.42	(0.32-0.53)	**7.69E-16**	**6.84E-15**
186	SM	SM(d18:2/16:0)	-0.17	(-0.2--0.13)	**2.98E-21**	**4.20E-21**	-1.16	(-1.21--1.12)	**0.00E+00**	**0.00E+00**	0.54	(0.49-0.58)	**1.64E-98**	**2.67E-98**	0.38	(0.31-0.45)	**7.29E-24**	**2.06E-22**
187	SM	SM(34:3)	-0.15	(-0.19--0.11)	**7.39E-12**	**9.51E-12**	-1.16	(-1.21--1.1)	**8.84E-268**	**1.52E-267**	0.62	(0.57-0.67)	**1.26E-92**	**2.04E-92**	0.17	(0.08-0.27)	**5.25E-04**	**9.77E-04**
188	SM	SM(d18:2/17:0)	-0.10	(-0.14--0.06)	**1.88E-06**	**2.27E-06**	-1.18	(-1.23--1.13)	**3.42E-282**	**6.10E-282**	0.70	(0.65-0.75)	**2.06E-121**	**3.72E-121**	0.28	(0.19-0.36)	**7.18E-10**	**2.95E-09**
189	SM	SM(35:2) (b)	-0.32	(-0.37--0.27)	**2.35E-31**	**3.56E-31**	-2.48	(-2.55--2.41)	**0.00E+00**	**0.00E+00**	1.78	(1.71-1.85)	**6.56E-279**	**2.60E-278**	0.46	(0.34-0.58)	**4.12E-14**	**2.99E-13**
190	SM	SM(d18:1/18:0)/SM(d16:1/20:0)	-0.34	(-0.38--0.29)	**8.03E-48**	**1.40E-47**	-1.04	(-1.09--0.98)	**1.63E-213**	**2.45E-213**	0.52	(0.46-0.57)	**7.87E-70**	**1.18E-69**	0.14	(0.05-0.23)	**3.73E-03**	**6.17E-03**
191	SM	SM(d18:2/18:1)	-0.24	(-0.28--0.2)	**1.01E-31**	**1.55E-31**	-1.60	(-1.65--1.55)	**0.00E+00**	**0.00E+00**	1.11	(1.06-1.17)	**7.91E-228**	**2.42E-227**	0.14	(0.05-0.22)	**1.21E-03**	**2.13E-03**
192	SM	SM(37:1)	-0.33	(-0.38--0.29)	**4.37E-41**	**7.23E-41**	-2.27	(-2.33--2.21)	**0.00E+00**	**0.00E+00**	1.65	(1.58-1.71)	**1.85E-294**	**7.74E-294**	0.25	(0.14-0.35)	**3.04E-06**	**8.12E-06**
193	SM	SM(37:2)	-0.39	(-0.45--0.34)	**5.65E-46**	**9.72E-46**	-1.98	(-2.04--1.92)	**0.00E+00**	**0.00E+00**	1.05	(0.99-1.11)	**2.54E-170**	**5.65E-170**	0.63	(0.52-0.74)	**2.77E-26**	**1.66E-24**
194	SM	SM(d18:1/20:0)/SM(d16:1/22:0)	-0.34	(-0.38--0.3)	**6.67E-68**	**1.32E-67**	-1.41	(-1.46--1.37)	**0.00E+00**	**0.00E+00**	0.91	(0.86-0.96)	**2.42E-196**	**6.19E-196**	0.13	(0.05-0.21)	**1.01E-03**	**1.80E-03**
195	SM	SM(d18:2/20:0)	-0.43	(-0.47--0.38)	**1.10E-67**	**2.16E-67**	-0.92	(-0.98--0.86)	**4.22E-150**	**5.65E-150**	0.16	(0.1-0.22)	**8.75E-07**	**9.35E-07**	0.38	(0.28-0.48)	**3.19E-13**	**1.99E-12**
196	SM	SM(38:3) (a)	-0.43	(-0.47--0.39)	**1.93E-86**	**4.28E-86**	-1.16	(-1.21--1.11)	**1.03E-257**	**1.69E-257**	0.34	(0.29-0.39)	**6.09E-36**	**7.84E-36**	0.26	(0.17-0.34)	**1.87E-09**	**7.22E-09**
197	SM	SM(38:3) (b)	-0.33	(-0.39--0.28)	**1.74E-33**	**2.71E-33**	-2.09	(-2.16--2.03)	**0.00E+00**	**0.00E+00**	1.24	(1.17-1.31)	**6.48E-186**	**1.56E-185**	0.61	(0.49-0.73)	**4.74E-22**	**9.10E-21**
198	SM	SM(d16:1/23:0)/SM(d17:1/22:0)	-0.29	(-0.33--0.25)	**1.33E-42**	**2.21E-42**	-2.61	(-2.66--2.56)	**0.00E+00**	**0.00E+00**	2.25	(2.2-2.3)	**0.00E+00**	**0.00E+00**	-0.03	(-0.12-0.07)	5.67E-01	6.29E-01
199	SM	SM(d18:1/22:0)/SM(d16:1/24:0)	-0.35	(-0.39--0.31)	**5.48E-59**	**1.03E-58**	-1.97	(-2.02--1.92)	**0.00E+00**	**0.00E+00**	1.46	(1.41-1.51)	**0.00E+00**	**0.00E+00**	0.10	(0.01-0.18)	**2.85E-02**	**4.06E-02**
200	SM	SM(d16:1/24:1)	-0.34	(-0.39--0.3)	**3.76E-47**	**6.50E-47**	-2.11	(-2.16--2.05)	**0.00E+00**	**0.00E+00**	1.50	(1.45-1.56)	**1.54E-297**	**6.53E-297**	0.16	(0.06-0.26)	**1.71E-03**	**2.97E-03**
201	SM	SM(d18:2/22:0)	-0.12	(-0.16--0.08)	**8.02E-09**	**9.95E-09**	-1.17	(-1.22--1.12)	**2.24E-270**	**3.89E-270**	0.76	(0.7-0.81)	**1.16E-139**	**2.25E-139**	0.32	(0.23-0.4)	**1.28E-13**	**8.52E-13**

202	SM	SM(40:3) (a)	-0.69	(-0.74--0.65)	**5.43E-162**	**2.19E-161**	-1.53	(-1.58--1.47)	**0.00E+00**	**0.00E+00**	0.39	(0.34-0.45)	**6.70E-41**	**8.81E-41**	0.39	(0.29-0.48)	**2.45E-15**	**2.03E-14**
203	SM	SM(40:3) (b)	-0.44	(-0.49--0.39)	**1.73E-63**	**3.31E-63**	-1.93	(-1.99--1.87)	**0.00E+00**	**0.00E+00**	1.23	(1.16-1.29)	**1.48E-197**	**3.83E-197**	0.26	(0.15-0.37)	**6.31E-06**	**1.56E-05**
204	SM	SM(41:0)	-0.46	(-0.5--0.42)	**4.49E-98**	**1.10E-97**	-1.90	(-1.97--1.83)	**0.00E+00**	**0.00E+00**	1.47	(1.39-1.55)	**6.63E-200**	**1.75E-199**	-0.10	(-0.19--0.01)	**2.84E-02**	**4.06E-02**
205	SM	SM(41:1)	-0.42	(-0.46--0.38)	**1.03E-93**	**2.40E-93**	-3.27	(-3.33--3.22)	**0.00E+00**	**0.00E+00**	2.84	(2.78-2.9)	**0.00E+00**	**0.00E+00**	-0.03	(-0.12-0.05)	4.38E-01	4.99E-01
206	SM	SM(d17:1/24:1)	-0.36	(-0.4--0.32)	**3.19E-63**	**6.08E-63**	-1.78	(-1.83--1.73)	**0.00E+00**	**0.00E+00**	1.24	(1.19-1.3)	**4.57E-262**	**1.65E-261**	0.17	(0.08-0.26)	**2.53E-04**	**4.95E-04**
207	SM	SM(d18:2/23:0)	-0.19	(-0.23--0.15)	**1.15E-23**	**1.65E-23**	-2.92	(-2.98--2.87)	**0.00E+00**	**0.00E+00**	2.59	(2.53-2.65)	**0.00E+00**	**0.00E+00**	0.18	(0.11-0.26)	**5.79E-06**	**1.45E-05**
208	SM	SM(d18:1/24:0)	-0.20	(-0.24--0.16)	**1.86E-22**	**2.64E-22**	-1.22	(-1.27--1.17)	**1.26E-281**	**2.24E-281**	0.96	(0.91-1.01)	**4.06E-198**	**1.05E-197**	0.09	(0-0.17)	**3.96E-02**	5.51E-02
209	SM	SM(d18:1/24:1)	-0.55	(-0.6--0.51)	**9.06E-102**	**2.32E-101**	-1.89	(-1.95--1.84)	**0.00E+00**	**0.00E+00**	1.09	(1.04-1.15)	**5.54E-217**	**1.60E-216**	0.30	(0.21-0.4)	**1.29E-09**	**5.19E-09**
210	SM	SM(d18:2/24:0)	-0.05	(-0.09--0.02)	**2.79E-03**	**3.13E-03**	-1.02	(-1.07--0.98)	**4.71E-243**	**7.49E-243**	0.78	(0.73-0.82)	**4.03E-157**	**8.22E-157**	0.32	(0.25-0.39)	**9.02E-17**	**9.21E-16**
211	SM	SM(43:1)	0.05	(0.01-0.09)	**2.68E-02**	**2.89E-02**	-0.87	(-0.94--0.81)	**4.04E-123**	**5.10E-123**	1.03	(0.96-1.1)	**2.25E-138**	**4.35E-138**	-0.09	(-0.18-0)	6.13E-02	8.18E-02
212	SM	SM(44:1)	0.21	(0.17-0.24)	**9.73E-28**	**1.43E-27**	0.59	(0.54-0.63)	**3.58E-109**	**4.40E-109**	-0.32	(-0.37--0.28)	**3.64E-39**	**4.76E-39**	0.04	(-0.04-0.11)	3.19E-01	3.76E-01
213	SM	SM(44:2)	0.01	(-0.03-0.06)	5.36E-01	5.48E-01	0.19	(0.14-0.24)	**1.71E-12**	**1.84E-12**	-0.28	(-0.33--0.23)	**1.09E-28**	**1.35E-28**	0.18	(0.09-0.27)	**5.10E-05**	**1.12E-04**
214	SM	SM(44:3) (a)	0.00	(-0.04-0.04)	8.80E-01	8.81E-01	0.25	(0.2-0.3)	**1.73E-22**	**1.88E-22**	-0.50	(-0.54--0.45)	**1.31E-84**	**2.07E-84**	0.39	(0.31-0.47)	**4.89E-20**	**7.34E-19**
215	LPC	LPC(14:0) [sn2]	0.36	(0.29-0.43)	**6.81E-24**	**9.79E-24**	0.44	(0.37-0.5)	**1.22E-36**	**1.37E-36**	-0.31	(-0.37--0.24)	**1.53E-21**	**1.80E-21**	0.11	(-0.05-0.26)	1.74E-01	2.14E-01
216	LPC	LPC(14:0) [sn1]	0.39	(0.32-0.45)	**7.58E-28**	**1.12E-27**	0.33	(0.27-0.4)	**8.14E-24**	**8.90E-24**	-0.15	(-0.21--0.09)	**4.81E-07**	**5.15E-07**	0.03	(-0.12-0.18)	7.05E-01	7.50E-01
217	LPC	LPC(15:0) [sn2]	0.59	(0.54-0.65)	**1.37E-88**	**3.07E-88**	0.06	(0.01-0.12)	**3.01E-02**	**3.06E-02**	0.26	(0.2-0.32)	**5.78E-16**	**6.64E-16**	0.25	(0.12-0.38)	**1.67E-04**	**3.36E-04**
218	LPC	LPC(15:0) [sn1]	0.60	(0.56-0.65)	**6.72E-106**	**1.78E-105**	-0.07	(-0.13--0.02)	**5.90E-03**	**6.10E-03**	0.39	(0.33-0.45)	**1.69E-36**	**2.18E-36**	0.30	(0.18-0.42)	**1.20E-06**	**3.37E-06**
219	LPC	LPC(16:0) [sn2]	0.59	(0.55-0.63)	**9.88E-151**	**3.51E-150**	0.06	(0.02-0.11)	**3.59E-03**	**3.73E-03**	0.22	(0.17-0.26)	**1.21E-18**	**1.41E-18**	0.49	(0.39-0.58)	**4.01E-23**	**9.17E-22**
220	LPC	LPC(16:0) [sn1]	0.70	(0.66-0.75)	**2.46E-151**	**8.94E-151**	-0.04	(-0.09-0.02)	1.68E-01	1.70E-01	0.36	(0.3-0.42)	**6.75E-35**	**8.64E-35**	0.59	(0.48-0.7)	**4.60E-24**	**1.47E-22**
221	LPC	LPC(16:1) [sn2]	0.83	(0.77-0.89)	**1.31E-133**	**4.16E-133**	1.93	(1.86-1.99)	**0.00E+00**	**0.00E+00**	-1.43	(-1.49--1.37)	**3.48E-261**	**1.24E-260**	0.28	(0.16-0.39)	**4.60E-06**	**1.18E-05**
222	LPC	LPC(16:1) [sn1]	0.86	(0.81-0.91)	**1.87E-169**	**8.54E-169**	1.95	(1.89-2.01)	**0.00E+00**	**0.00E+00**	-1.39	(-1.45--1.34)	**5.15E-267**	**1.93E-266**	0.25	(0.14-0.36)	**1.16E-05**	**2.80E-05**
223	LPC	LPC(15-MHDA) [sn2]	0.94	(0.87-1)	**8.31E-130**	**2.56E-129**	0.43	(0.35-0.51)	**1.78E-25**	**1.96E-25**	0.23	(0.14-0.31)	**4.40E-07**	**4.73E-07**	0.36	(0.19-0.52)	**2.22E-05**	**5.25E-05**
224	LPC	LPC(15-MHDA) [sn1] / LPC(17:0) [sn2]	0.93	(0.87-0.99)	**1.62E-167**	**7.02E-167**	0.00	(-0.06-0.07)	8.88E-01	8.88E-01	0.66	(0.59-0.73)	**1.79E-66**	**2.62E-66**	0.41	(0.27-0.55)	**5.50E-09**	**2.00E-08**
225	LPC	LPC(17:0) [sn1]	0.88	(0.83-0.92)	**1.61E-213**	**9.81E-213**	-0.37	(-0.42--0.33)	**5.70E-48**	**6.50E-48**	0.97	(0.92-1.03)	**3.05E-182**	**7.18E-182**	0.46	(0.35-0.57)	**2.88E-15**	**2.34E-14**
226	LPC	LPC(17:1) [sn1] (a) / LPC(17:1) [sn2] (b)	0.85	(0.8-0.9)	**8.19E-166**	**3.45E-165**	1.09	(1.03-1.15)	**4.73E-209**	**7.03E-209**	-0.53	(-0.59--0.47)	**3.07E-58**	**4.34E-58**	0.39	(0.26-0.52)	**1.63E-09**	**6.40E-09**
227	LPC	LPC(18:0) [sn2]	1.18	(1.13-1.22)	**0.00E+00**	**0.00E+00**	0.02	(-0.02-0.07)	2.93E-01	2.95E-01	0.97	(0.92-1.02)	**6.74E-205**	**1.82E-204**	0.31	(0.21-0.42)	**5.11E-09**	**1.87E-08**
228	LPC	LPC(18:0) [sn1]	1.20	(1.16-1.24)	**0.00E+00**	**0.00E+00**	-0.03	(-0.08-0.01)	1.46E-01	1.48E-01	1.06	(1.01-1.12)	**2.64E-210**	**7.42E-210**	0.31	(0.2-0.43)	**5.14E-08**	**1.64E-07**
229	LPC	LPC(18:1) [sn2]	1.05	(1-1.1)	**2.09E-240**	**1.54E-239**	0.74	(0.68-0.79)	**1.62E-125**	**2.07E-125**	0.17	(0.11-0.23)	**1.48E-08**	**1.60E-08**	0.48	(0.37-0.6)	**1.92E-15**	**1.61E-14**
230	LPC	LPC(18:1) [sn1]	1.12	(1.07-1.16)	**9.44E-308**	**1.30E-306**	0.88	(0.83-0.93)	**2.73E-192**	**3.95E-192**	0.20	(0.15-0.26)	**4.68E-14**	**5.25E-14**	0.36	(0.25-0.46)	**2.93E-11**	**1.44E-10**
231	LPC	LPC(18:2) [sn2]	1.24	(1.19-1.29)	**0.00E+00**	**0.00E+00**	0.34	(0.28-0.39)	**1.67E-33**	**1.86E-33**	0.84	(0.78-0.89)	**7.57E-137**	**1.44E-136**	0.48	(0.37-0.59)	**1.90E-17**	**2.17E-16**
232	LPC	LPC(18:2) [sn1]	1.67	(1.62-1.72)	**0.00E+00**	**0.00E+00**	0.83	(0.77-0.88)	**2.22E-139**	**2.90E-139**	0.77	(0.72-0.83)	**7.76E-114**	**1.37E-113**	0.51	(0.39-0.62)	**2.81E-16**	**2.60E-15**
233	LPC	LPC(18:3) [sn2] (a)	0.71	(0.65-0.78)	**9.31E-90**	**2.11E-89**	-0.52	(-0.58--0.45)	**8.29E-51**	**9.50E-51**	1.29	(1.22-1.35)	**4.19E-210**	**1.16E-209**	0.22	(0.08-0.37)	**2.94E-03**	**4.93E-03**
234	LPC	LPC(18:3) [sn1] (a)/LPC(18:3) [sn2] (b)	1.24	(1.18-1.31)	**7.95E-205**	**4.65E-204**	1.06	(0.98-1.13)	**2.76E-140**	**3.62E-140**	0.29	(0.21-0.36)	**1.17E-13**	**1.30E-13**	0.15	(0-0.3)	5.76E-02	7.73E-02
235	LPC	LPC(18:3) (a) [sn1] [104_sn1]	1.31	(1.25-1.38)	**3.01E-214**	**1.88E-213**	1.10	(1.03-1.18)	**1.57E-146**	**2.07E-146**	0.31	(0.24-0.38)	**6.17E-16**	**7.05E-16**	0.18	(0.02-0.33)	**2.42E-02**	**3.51E-02**
236	LPC	LPC(19:0) [sn1] (a) / LPC(19:0) [sn2] (b)	0.83	(0.78-0.87)	**5.18E-199**	**2.89E-198**	-0.40	(-0.45--0.34)	**1.31E-40**	**1.48E-40**	1.04	(0.97-1.1)	**5.08E-164**	**1.08E-163**	0.45	(0.34-0.56)	**4.00E-15**	**3.14E-14**
237	LPC	LPC(20:0) [sn1]	0.96	(0.92-1)	**1.17E-266**	**1.19E-265**	-0.32	(-0.36--0.28)	**2.44E-44**	**2.77E-44**	1.18	(1.13-1.23)	**6.10E-283**	**2.48E-282**	0.42	(0.32-0.52)	**6.50E-16**	**5.89E-15**
238	LPC	LPC(20:1) [sn2]	0.65	(0.6-0.69)	**4.59E-139**	**1.52E-138**	-0.29	(-0.34--0.25)	**4.01E-34**	**4.46E-34**	0.79	(0.74-0.84)	**1.31E-147**	**2.58E-147**	0.42	(0.31-0.53)	**6.68E-14**	**4.65E-13**
239	LPC	LPC(20:1) [sn1]	0.77	(0.73-0.82)	**8.69E-177**	**4.13E-176**	-0.23	(-0.28--0.19)	**2.18E-23**	**2.37E-23**	0.86	(0.81-0.91)	**4.35E-178**	**1.01E-177**	0.47	(0.36-0.57)	**6.60E-17**	**7.04E-16**
240	LPC	LPC(20:2) [sn2]	0.24	(0.19-0.28)	**3.38E-24**	**4.87E-24**	0.45	(0.4-0.51)	**1.24E-51**	**1.43E-51**	-0.31	(-0.37--0.25)	**1.72E-24**	**2.09E-24**	0.38	(0.28-0.49)	**3.77E-13**	**2.32E-12**
241	LPC	LPC(20:2) [sn1]	0.60	(0.56-0.64)	**1.12E-132**	**3.54E-132**	0.93	(0.88-0.98)	**1.02E-192**	**1.48E-192**	-0.42	(-0.47--0.36)	**7.42E-45**	**9.89E-45**	0.38	(0.28-0.48)	**1.64E-13**	**1.08E-12**
242	LPC	LPC(20:3) [sn2]	0.37	(0.31-0.42)	**4.77E-34**	**7.45E-34**	1.65	(1.59-1.71)	**0.00E+00**	**0.00E+00**	-1.33	(-1.39--1.27)	**2.58E-237**	**8.21E-237**	0.05	(-0.08-0.17)	4.68E-01	5.28E-01
243	LPC	LPC(20:3) [sn1]	0.88	(0.82-0.94)	**1.48E-142**	**4.99E-142**	2.40	(2.33-2.46)	**0.00E+00**	**0.00E+00**	-1.54	(-1.6--1.47)	**1.91E-264**	**7.07E-264**	0.08	(-0.05-0.21)	2.35E-01	2.83E-01
244	LPC	LPC(20:3) [104_sn1]	0.93	(0.87-0.99)	**3.80E-151**	**1.37E-150**	2.43	(2.37-2.5)	**0.00E+00**	**0.00E+00**	-1.53	(-1.59--1.46)	**2.10E-260**	**7.35E-260**	0.07	(-0.06-0.2)	2.76E-01	3.28E-01
245	LPC	LPC(20:4) [sn2]	0.87	(0.83-0.92)	**8.47E-205**	**4.90E-204**	2.43	(2.37-2.48)	**0.00E+00**	**0.00E+00**	-1.79	(-1.85--1.74)	**0.00E+00**	**0.00E+00**	0.41	(0.3-0.51)	**4.58E-14**	**3.23E-13**
246	LPC	LPC(20:4) [sn1]	1.34	(1.29-1.39)	**0.00E+00**	**0.00E+00**	3.13	(3.08-3.19)	**0.00E+00**	**0.00E+00**	-2.05	(-2.11--1.99)	**0.00E+00**	**0.00E+00**	0.49	(0.37-0.6)	**1.64E-16**	**1.61E-15**
247	LPC	LPC(20:5) [sn2]	1.19	(1.1-1.29)	**4.81E-105**	**1.27E-104**	0.72	(0.61-0.83)	**5.81E-37**	**6.52E-37**	-0.28	(-0.38--0.17)	**1.67E-07**	**1.80E-07**	0.93	(0.72-1.14)	**1.35E-17**	**1.58E-16**
248	LPC	LPC(20:5) [sn1]	1.54	(1.44-1.64)	**1.67E-153**	**6.23E-153**	1.24	(1.13-1.35)	**6.00E-95**	**7.18E-95**	-0.45	(-0.55--0.35)	**6.00E-17**	**6.94E-17**	0.93	(0.72-1.15)	**3.27E-17**	**3.65E-16**
249	LPC	LPC(22:4) [sn2]	0.23	(0.17-0.28)	**3.40E-16**	**4.58E-16**	0.95	(0.89-1.02)	**1.65E-155**	**2.23E-155**	-0.47	(-0.53--0.41)	**1.28E-48**	**1.72E-48**	-0.11	(-0.23-0.01)	7.31E-02	9.64E-02
250	LPC	LPC(22:4) [sn1]	0.63	(0.58-0.68)	**9.98E-99**	**2.46E-98**	1.76	(1.7-1.82)	**0.00E+00**	**0.00E+00**	-0.91	(-0.97--0.85)	**4.93E-147**	**9.65E-147**	-0.04	(-0.16-0.09)	5.58E-01	6.20E-01
251	LPC	LPC(22:5) [sn2] (n3)	0.90	(0.84-0.95)	**1.46E-167**	**6.39E-167**	0.49	(0.43-0.56)	**1.81E-43**	**2.05E-43**	0.26	(0.19-0.32)	**5.60E-14**	**6.25E-14**	0.34	(0.22-0.46)	**2.17E-08**	**7.39E-08**
252	LPC	LPC(22:5) [sn1] (n3)/LPC(22:5) [sn2] (n6)	1.07	(1.02-1.13)	**1.05E-226**	**7.13E-226**	0.79	(0.73-0.85)	**1.50E-118**	**1.88E-118**	0.28	(0.22-0.34)	**5.56E-20**	**6.51E-20**	0.22	(0.11-0.34)	**2.19E-04**	**4.29E-04**
253	LPC	LPC(22:5) [sn1] (n6)	0.67	(0.6-0.73)	**7.86E-78**	**1.65E-77**	1.54	(1.46-1.63)	**2.54E-206**	**3.76E-206**	-0.51	(-0.59--0.43)	**8.39E-33**	**1.06E-32**	-0.10	(-0.24-0.04)	1.63E-01	2.01E-01

254	LPC	LPC(22:5) (n3) [sn1] [104_sn1]	1.37	(1.31-1.42)	**1.35E-268**	**1.41E-267**	0.79	(0.72-0.86)	**2.21E-95**	**2.66E-95**	0.48	(0.41-0.54)	**2.73E-42**	**3.61E-42**	0.34	(0.21-0.46)	**1.36E-07**	**4.17E-07**
255	LPC	LPC(22:6) [sn2]	0.44	(0.38-0.5)	**2.36E-50**	**4.15E-50**	0.74	(0.68-0.8)	**8.25E-106**	**1.01E-105**	-0.53	(-0.6--0.47)	**1.06E-56**	**1.47E-56**	0.46	(0.33-0.58)	**2.08E-12**	**1.18E-11**
256	LPC	LPC(22:6) [sn1]	0.92	(0.86-0.98)	**1.99E-159**	**7.78E-159**	1.43	(1.36-1.49)	**8.90E-262**	**1.48E-261**	-0.71	(-0.77--0.64)	**2.11E-86**	**3.34E-86**	0.46	(0.33-0.59)	**1.15E-11**	**5.95E-11**
257	LPC(O)	LPC(O-16:0)	1.07	(1.03-1.11)	**0.00E+00**	**0.00E+00**	0.91	(0.86-0.95)	**9.99E-233**	**1.56E-232**	-0.15	(-0.2--0.1)	**1.17E-08**	**1.27E-08**	0.53	(0.43-0.64)	**2.18E-22**	**4.76E-21**
258	LPC(O)	LPC(O-18:0)	0.90	(0.86-0.95)	**3.94E-200**	**2.22E-199**	-0.20	(-0.26--0.15)	**8.06E-13**	**8.70E-13**	0.83	(0.77-0.89)	**1.88E-123**	**3.47E-123**	0.44	(0.32-0.56)	**1.92E-12**	**1.11E-11**
259	LPC(O)	LPC(O-18:1)	0.98	(0.94-1.02)	**4.01E-285**	**4.70E-284**	0.60	(0.55-0.64)	**5.89E-123**	**7.43E-123**	0.20	(0.15-0.25)	**9.84E-14**	**1.10E-13**	0.36	(0.26-0.46)	**1.07E-11**	**5.58E-11**
260	LPC(O)	LPC(O-20:0)	0.94	(0.89-0.98)	**7.14E-218**	**4.57E-217**	-0.09	(-0.16--0.02)	**1.21E-02**	**1.24E-02**	0.80	(0.73-0.88)	**8.89E-82**	**1.37E-81**	0.47	(0.35-0.59)	**4.06E-15**	**3.14E-14**
261	LPC(O)	LPC(O-20:1)	0.92	(0.85-0.99)	**6.51E-132**	**2.04E-131**	0.10	(0.02-0.19)	**1.41E-02**	**1.44E-02**	0.34	(0.26-0.42)	**8.46E-17**	**9.76E-17**	0.74	(0.61-0.88)	**5.17E-26**	**2.76E-24**
262	LPC(O)	LPC(O-22:0)	0.47	(0.43-0.52)	**9.97E-94**	**2.32E-93**	-0.13	(-0.17--0.08)	**6.07E-07**	**6.42E-07**	0.40	(0.36-0.45)	**7.91E-63**	**1.14E-62**	0.44	(0.36-0.53)	**5.49E-24**	**1.65E-22**
263	LPC(O)	LPC(O-22:1)	0.70	(0.65-0.75)	**6.78E-138**	**2.21E-137**	-0.09	(-0.14--0.03)	**1.31E-03**	**1.37E-03**	0.59	(0.53-0.64)	**2.31E-82**	**3.57E-82**	0.56	(0.45-0.67)	**3.09E-22**	**6.45E-21**
264	LPC(O)	LPC(O-24:0)	0.17	(0.13-0.22)	**2.48E-12**	**3.21E-12**	-0.86	(-0.91--0.8)	**1.74E-147**	**2.31E-147**	0.83	(0.78-0.88)	**4.34E-169**	**9.61E-169**	0.43	(0.34-0.52)	**1.07E-20**	**1.71E-19**
265	LPC(O)	LPC(O-24:1)	0.29	(0.24-0.33)	**2.52E-31**	**3.80E-31**	-0.74	(-0.8--0.69)	**1.01E-127**	**1.31E-127**	0.84	(0.79-0.89)	**1.83E-168**	**4.01E-168**	0.44	(0.35-0.54)	**1.31E-19**	**1.84E-18**
266	LPC(O)	LPC(O-24:2)	0.48	(0.43-0.53)	**2.81E-66**	**5.48E-66**	0.13	(0.08-0.19)	**3.54E-06**	**3.73E-06**	0.07	(0.02-0.12)	**9.02E-03**	**9.35E-03**	0.61	(0.5-0.72)	**7.06E-26**	**3.39E-24**
267	LPC(P)	LPC(P-16:0)	1.38	(1.34-1.42)	**0.00E+00**	**0.00E+00**	0.90	(0.85-0.94)	**2.45E-221**	**3.75E-221**	0.05	(0-0.1)	6.64E-02	6.79E-02	0.63	(0.52-0.73)	**1.44E-30**	**1.39E-28**
268	LPC(P)	LPC(P-18:0)	0.98	(0.94-1.02)	**1.40E-281**	**1.61E-280**	0.59	(0.54-0.63)	**1.81E-119**	**2.28E-119**	0.21	(0.16-0.26)	**3.68E-15**	**4.14E-15**	0.35	(0.25-0.45)	**2.20E-11**	**1.10E-10**
269	LPC(P)	LPC(P-18:1)	1.23	(1.18-1.28)	**5.32E-291**	**6.73E-290**	-0.07	(-0.12--0.01)	**2.53E-02**	**2.59E-02**	0.79	(0.72-0.85)	**7.64E-106**	**1.29E-105**	0.64	(0.52-0.76)	**3.04E-25**	**1.22E-23**
270	LPC(P)	LPC(P-20:0)	0.86	(0.81-0.91)	**3.90E-169**	**1.75E-168**	-0.07	(-0.13--0.01)	**1.35E-02**	**1.39E-02**	0.81	(0.75-0.87)	**2.92E-117**	**5.23E-117**	0.43	(0.31-0.55)	**7.83E-12**	**4.17E-11**
271	LPE	LPE(16:0) [sn2]	-0.19	(-0.24--0.14)	**9.60E-15**	**1.28E-14**	-0.05	(-0.1-0)	6.50E-02	6.61E-02	-0.53	(-0.58--0.47)	**2.70E-69**	**4.01E-69**	0.72	(0.6-0.83)	**9.17E-34**	**2.20E-31**
272	LPE	LPE(16:0) [sn1]	-0.26	(-0.3--0.21)	**2.90E-27**	**4.23E-27**	-0.14	(-0.19--0.09)	**4.47E-08**	**4.77E-08**	-0.51	(-0.56--0.46)	**2.19E-72**	**3.32E-72**	0.71	(0.6-0.82)	**1.55E-36**	**7.46E-34**
273	LPE	LPE(18:0) [sn2]	0.18	(0.14-0.22)	**9.26E-17**	**1.26E-16**	-0.26	(-0.3--0.21)	**2.06E-25**	**2.26E-25**	0.15	(0.1-0.19)	**2.37E-09**	**2.59E-09**	0.51	(0.42-0.61)	**2.15E-24**	**7.36E-23**
274	LPE	LPE(18:0) [sn1]	0.13	(0.08-0.17)	**3.87E-09**	**4.82E-09**	-0.36	(-0.4--0.31)	**7.99E-50**	**9.13E-50**	0.19	(0.14-0.24)	**4.76E-14**	**5.32E-14**	0.53	(0.43-0.63)	**1.66E-24**	**6.14E-23**
275	LPE	LPE(18:1) [sn2]	0.01	(-0.04-0.07)	6.18E-01	6.27E-01	-0.23	(-0.28--0.17)	**1.40E-15**	**1.51E-15**	0.21	(0.15-0.27)	**2.95E-11**	**3.26E-11**	0.25	(0.12-0.38)	**1.84E-04**	**3.67E-04**
276	LPE	LPE(18:1) [sn1]	0.05	(-0.01-0.1)	7.61E-02	8.08E-02	-0.28	(-0.34--0.23)	**8.21E-24**	**8.96E-24**	0.31	(0.25-0.37)	**8.46E-23**	**1.01E-22**	0.25	(0.11-0.38)	**2.98E-04**	**5.75E-04**
277	LPE	LPE(18:2) [sn2]	0.72	(0.66-0.78)	**6.14E-100**	**1.53E-99**	-0.16	(-0.22--0.09)	**1.09E-05**	**1.15E-05**	0.85	(0.78-0.92)	**2.37E-99**	**3.87E-99**	0.29	(0.15-0.43)	**3.35E-05**	**7.78E-05**
278	LPE	LPE(18:2) [sn1]	0.90	(0.84-0.96)	**6.95E-145**	**2.45E-144**	0.08	(0.01-0.14)	**2.74E-02**	**2.80E-02**	0.82	(0.75-0.89)	**8.28E-100**	**1.36E-99**	0.27	(0.13-0.4)	**1.11E-04**	**2.27E-04**
279	LPE	LPE(20:4) [sn1]	1.04	(1-1.09)	**2.02E-258**	**1.80E-257**	2.28	(2.22-2.34)	**0.00E+00**	**0.00E+00**	-1.35	(-1.4--1.29)	**1.83E-270**	**6.92E-270**	0.31	(0.21-0.4)	**2.45E-09**	**9.27E-09**
280	LPE	LPE(22:6) [sn2]	0.34	(0.29-0.4)	**2.66E-35**	**4.20E-35**	1.57	(1.51-1.63)	**0.00E+00**	**0.00E+00**	-1.66	(-1.72--1.6)	**7.89E-308**	**3.75E-307**	0.57	(0.45-0.69)	**5.75E-20**	**8.37E-19**
281	LPE	LPE(22:6) [sn1]	0.64	(0.58-0.69)	**3.21E-100**	**8.03E-100**	1.93	(1.87-1.99)	**0.00E+00**	**0.00E+00**	-1.72	(-1.78--1.66)	**0.00E+00**	**0.00E+00**	0.60	(0.49-0.72)	**1.26E-23**	**3.36E-22**
282	PC	PC(28:0)	-0.83	(-0.93--0.73)	**1.54E-54**	**2.79E-54**	-1.90	(-1.99--1.81)	**1.20E-229**	**1.86E-229**	1.03	(0.96-1.11)	**7.60E-118**	**1.37E-117**	0.02	(-0.19-0.23)	8.68E-01	8.96E-01
283	PC	PC(14:0_16:0)	-0.99	(-1.06--0.93)	**1.11E-151**	**4.05E-151**	-1.70	(-1.77--1.63)	**3.48E-279**	**6.09E-279**	0.58	(0.52-0.65)	**5.72E-59**	**8.15E-59**	0.18	(0.04-0.31)	**1.16E-02**	**1.80E-02**
284	PC	PC(31:0) (a)	-0.87	(-0.97--0.78)	**3.10E-64**	**5.95E-64**	-1.94	(-2.04--1.84)	**1.04E-210**	**1.56E-210**	1.26	(1.16-1.37)	**1.02E-100**	**1.68E-100**	-0.48	(-0.71--0.26)	**2.31E-05**	**5.44E-05**
285	PC	PC(31:0) (b)	-0.65	(-0.71--0.6)	**3.44E-94**	**8.17E-94**	-1.27	(-1.35--1.19)	**1.06E-158**	**1.44E-158**	0.56	(0.47-0.64)	**2.19E-33**	**2.78E-33**	-0.01	(-0.15-0.13)	8.75E-01	9.01E-01
286	PC	PC(31:1)	-1.10	(-1.18--1.02)	**8.99E-126**	**2.68E-125**	-1.66	(-1.74--1.57)	**2.76E-209**	**4.11E-209**	0.35	(0.27-0.44)	**5.83E-16**	**6.68E-16**	-0.07	(-0.24-0.11)	4.47E-01	5.07E-01
287	PC	PC(16:0_16:0)	-0.71	(-0.76--0.67)	**1.73E-162**	**7.02E-162**	-0.95	(-1.01--0.89)	**5.49E-160**	**7.54E-160**	0.06	(0-0.12)	**3.55E-02**	**3.65E-02**	0.27	(0.18-0.36)	**1.67E-08**	**5.81E-08**
288	PC	PC(32:1)	-1.19	(-1.26--1.12)	**4.60E-164**	**1.92E-163**	-1.12	(-1.21--1.04)	**2.96E-127**	**3.80E-127**	-0.17	(-0.24--0.09)	**1.57E-05**	**1.66E-05**	-0.14	(-0.29-0.01)	6.74E-02	8.96E-02
289	PC	PC(32:2)	-1.07	(-1.14--1.01)	**3.14E-159**	**1.21E-158**	-3.48	(-3.55--3.42)	**0.00E+00**	**0.00E+00**	2.29	(2.23-2.36)	**0.00E+00**	**0.00E+00**	0.02	(-0.13-0.18)	7.56E-01	7.94E-01
290	PC	PC(33:0) (a)	-0.80	(-0.87--0.73)	**6.49E-94**	**1.52E-93**	-1.18	(-1.26--1.09)	**3.97E-127**	**5.09E-127**	0.17	(0.08-0.27)	**2.79E-04**	**2.94E-04**	-0.01	(-0.18-0.16)	9.47E-01	9.57E-01
291	PC	PC(33:0) (b)	-0.37	(-0.42--0.32)	**1.09E-40**	**1.78E-40**	-1.54	(-1.61--1.46)	**7.08E-248**	**1.14E-247**	0.97	(0.9-1.05)	**1.84E-109**	**3.16E-109**	0.17	(0.05-0.29)	**7.28E-03**	**1.16E-02**
292	PC	PC(33:1)	-1.16	(-1.22--1.09)	**4.15E-189**	**2.14E-188**	-2.32	(-2.39--2.25)	**0.00E+00**	**0.00E+00**	1.08	(1.01-1.15)	**4.74E-153**	**9.59E-153**	0.02	(-0.12-0.16)	7.73E-01	8.10E-01
293	PC	PC(33:2)	-0.83	(-0.88--0.77)	**2.18E-140**	**7.32E-140**	-4.20	(-4.27--4.14)	**0.00E+00**	**0.00E+00**	3.13	(3.06-3.2)	**0.00E+00**	**0.00E+00**	0.28	(0.15-0.41)	**1.67E-05**	**3.99E-05**
294	PC	PC(16:0_18:0)	0.10	(0.06-0.14)	**5.36E-07**	**6.53E-07**	-0.81	(-0.87--0.76)	**6.94E-163**	**9.58E-163**	0.76	(0.71-0.81)	**2.58E-136**	**4.88E-136**	0.23	(0.15-0.32)	**1.34E-07**	**4.11E-07**
295	PC	PC(16:0_18:1)	-1.07	(-1.12--1.01)	**2.88E-216**	**1.82E-215**	-2.34	(-2.41--2.28)	**0.00E+00**	**0.00E+00**	1.24	(1.17-1.3)	**3.40E-199**	**8.92E-199**	0.10	(-0.03-0.22)	1.25E-01	1.59E-01
296	PC	PC(16:0_18:2)	-0.57	(-0.6--0.53)	**3.11E-180**	**1.49E-179**	-3.50	(-3.56--3.43)	**0.00E+00**	**0.00E+00**	2.79	(2.72-2.86)	**0.00E+00**	**0.00E+00**	0.24	(0.16-0.33)	**2.70E-08**	**9.06E-08**
297	PC	PC(16:1_18:2)	-0.69	(-0.75--0.64)	**2.22E-110**	**5.98E-110**	-2.96	(-3.03--2.9)	**0.00E+00**	**0.00E+00**	1.93	(1.87-2)	**0.00E+00**	**0.00E+00**	0.31	(0.19-0.43)	**1.83E-07**	**5.52E-07**
298	PC	PC(16:0_18:3) (a)	-1.06	(-1.12--1)	**2.61E-184**	**1.28E-183**	-3.64	(-3.71--3.56)	**0.00E+00**	**0.00E+00**	2.52	(2.45-2.6)	**0.00E+00**	**0.00E+00**	0.13	(0-0.27)	**4.89E-02**	6.63E-02
299	PC	PC(14:0_20:4)	-0.97	(-1.04--0.9)	**1.64E-119**	**4.70E-119**	-2.97	(-3.05--2.9)	**0.00E+00**	**0.00E+00**	1.76	(1.69-1.84)	**3.94E-273**	**1.51E-272**	0.11	(-0.05-0.28)	1.78E-01	2.18E-01
300	PC	PC(34:5)	-0.53	(-0.64--0.43)	**4.22E-21**	**5.92E-21**	-3.47	(-3.58--3.36)	**0.00E+00**	**0.00E+00**	2.21	(2.11-2.32)	**5.03E-234**	**1.57E-233**	0.62	(0.38-0.85)	**3.28E-07**	**9.66E-07**
301	PC	PC(15-MHDA_18:1)	-0.53	(-0.6--0.46)	**8.41E-45**	**1.43E-44**	-2.05	(-2.14--1.97)	**4.30E-295**	**7.85E-295**	1.51	(1.43-1.6)	**3.03E-174**	**6.92E-174**	-0.01	(-0.17-0.16)	9.27E-01	9.39E-01
302	PC	PC(17:0_18:1)	-0.57	(-0.62--0.52)	**9.17E-104**	**2.39E-103**	-1.91	(-1.97--1.86)	**0.00E+00**	**0.00E+00**	1.30	(1.25-1.36)	**5.03E-258**	**1.74E-257**	0.06	(-0.05-0.17)	2.65E-01	3.17E-01
303	PC	PC(15-MHDA_18:2)	-0.68	(-0.75--0.61)	**1.49E-68**	**2.96E-68**	-3.32	(-3.4--3.24)	**0.00E+00**	**0.00E+00**	2.51	(2.43-2.6)	**0.00E+00**	**0.00E+00**	0.20	(0.04-0.36)	**1.35E-02**	**2.05E-02**
304	PC	PC(17:0_18:2)	-0.46	(-0.51--0.41)	**1.27E-69**	**2.54E-69**	-3.32	(-3.38--3.26)	**0.00E+00**	**0.00E+00**	2.65	(2.58-2.71)	**0.00E+00**	**0.00E+00**	0.33	(0.22-0.44)	**1.89E-09**	**7.27E-09**
305	PC	PC(17:1_18:2)	-0.76	(-0.81--0.71)	**2.55E-144**	**8.86E-144**	-2.63	(-2.69--2.57)	**0.00E+00**	**0.00E+00**	1.66	(1.6-1.72)	**0.00E+00**	**0.00E+00**	0.12	(0-0.24)	**4.18E-02**	5.78E-02

306	PC	PC(15:0_20:3)	-0.32	(-0.35--0.3)	**4.66E-101**	**1.18E-100**	-2.84	(-2.91--2.77)	**0.00E+00**	**0.00E+00**	2.42	(2.34-2.5)	**0.00E+00**	**0.00E+00**	0.14	(0.07-0.21)	**5.45E-05**	**1.19E-04**
307	PC	PC(15:0_20:4)	-0.74	(-0.8--0.68)	**6.63E-105**	**1.74E-104**	-2.48	(-2.55--2.41)	**0.00E+00**	**0.00E+00**	1.28	(1.21-1.34)	**1.15E-187**	**2.82E-187**	0.46	(0.32-0.59)	**1.12E-10**	**5.06E-10**
308	PC	PC(35:5)	-0.29	(-0.41--0.18)	**2.40E-07**	**2.94E-07**	-3.24	(-3.36--3.12)	**0.00E+00**	**0.00E+00**	2.10	(1.99-2.22)	**4.45E-190**	**1.09E-189**	0.78	(0.55-1.02)	**1.74E-10**	**7.68E-10**
309	PC	PC(36:0)	-0.46	(-0.52--0.4)	**2.19E-50**	**3.87E-50**	1.13	(1.07-1.19)	**9.14E-218**	**1.38E-217**	-1.52	(-1.58--1.47)	**4.58E-307**	**2.14E-306**	-0.38	(-0.51--0.26)	**2.87E-09**	**1.07E-08**
310	PC	PC(18:0_18:1)	-0.34	(-0.39--0.28)	**4.23E-34**	**6.63E-34**	-1.24	(-1.3--1.19)	**1.16E-257**	**1.90E-257**	0.95	(0.9-1.01)	**4.74E-179**	**1.10E-178**	-0.10	(-0.21-0.01)	8.80E-02	1.15E-01
311	PC	PC(18:0_18:2)	-0.06	(-0.11--0.01)	**1.29E-02**	**1.41E-02**	-3.14	(-3.2--3.08)	**0.00E+00**	**0.00E+00**	3.02	(2.96-3.08)	**0.00E+00**	**0.00E+00**	0.10	(0-0.21)	6.13E-02	8.18E-02
312	PC	PC(16:0_20:3) (a)	-0.55	(-0.6--0.49)	**1.79E-80**	**3.80E-80**	-3.41	(-3.48--3.35)	**0.00E+00**	**0.00E+00**	2.72	(2.65-2.78)	**0.00E+00**	**0.00E+00**	0.42	(0.31-0.53)	**4.48E-13**	**2.69E-12**
313	PC	PC(16:0_20:3) (b)	-1.38	(-1.44--1.31)	**2.72E-241**	**2.04E-240**	-1.92	(-1.99--1.85)	**0.00E+00**	**0.00E+00**	0.39	(0.33-0.45)	**2.77E-34**	**3.53E-34**	0.00	(-0.13-0.13)	9.88E-01	9.88E-01
314	PC	PC(18:2_18:2)	-0.15	(-0.22--0.08)	**8.13E-06**	**9.68E-06**	-5.22	(-5.3--5.13)	**0.00E+00**	**0.00E+00**	5.02	(4.93-5.1)	**0.00E+00**	**0.00E+00**	0.52	(0.37-0.68)	**1.64E-11**	**8.36E-11**
315	PC	PC(16:1_20:4)	-0.61	(-0.66--0.55)	**6.60E-80**	**1.40E-79**	-1.43	(-1.5--1.37)	**1.55E-247**	**2.47E-247**	0.43	(0.37-0.49)	**1.71E-37**	**2.22E-37**	0.34	(0.22-0.47)	**4.74E-08**	**1.55E-07**
316	PC	PC(16:0_20:5)	-0.38	(-0.48--0.27)	**3.50E-12**	**4.52E-12**	-2.58	(-2.69--2.46)	**2.34E-265**	**3.98E-265**	1.34	(1.23-1.44)	**4.91E-109**	**8.33E-109**	0.90	(0.68-1.12)	**1.64E-15**	**1.40E-14**
317	PC	PC(36:6)	-1.15	(-1.23--1.07)	**6.42E-143**	**2.18E-142**	-3.30	(-3.38--3.22)	**0.00E+00**	**0.00E+00**	1.77	(1.7-1.85)	**2.92E-261**	**1.04E-260**	0.30	(0.12-0.47)	**9.02E-04**	**1.62E-03**
318	PC	PC(15-MHDA_20:4)	-0.82	(-0.89--0.75)	**8.14E-101**	**2.05E-100**	-1.78	(-1.86--1.71)	**1.75E-265**	**2.98E-265**	0.67	(0.58-0.75)	**2.08E-52**	**2.83E-52**	0.27	(0.12-0.43)	**6.20E-04**	**1.14E-03**
319	PC	PC(17:0_20:4)	-0.41	(-0.46--0.35)	**6.77E-44**	**1.14E-43**	-1.10	(-1.16--1.04)	**5.85E-188**	**8.36E-188**	0.29	(0.23-0.36)	**6.06E-20**	**7.07E-20**	0.41	(0.29-0.53)	**7.65E-11**	**3.60E-10**
320	PC	PC(15:0_22:6)	-0.83	(-0.9--0.77)	**7.04E-113**	**1.95E-112**	-2.79	(-2.87--2.72)	**0.00E+00**	**0.00E+00**	1.51	(1.44-1.59)	**5.42E-215**	**1.54E-214**	0.43	(0.28-0.57)	**1.67E-08**	**5.81E-08**
321	PC	PC(38:2)	-0.55	(-0.59--0.51)	**1.50E-119**	**4.33E-119**	-1.24	(-1.29--1.19)	**2.55E-297**	**4.68E-297**	0.59	(0.54-0.64)	**1.13E-101**	**1.88E-101**	0.10	(0.01-0.18)	**3.09E-02**	**4.35E-02**
322	PC	PC(18:0_20:3)	-0.46	(-0.53--0.4)	**1.76E-40**	**2.88E-40**	-0.21	(-0.27--0.14)	**2.76E-10**	**2.95E-10**	-0.30	(-0.36--0.24)	**1.70E-22**	**2.02E-22**	-0.37	(-0.51--0.24)	**8.97E-08**	**2.85E-07**
323	PC	PC(18:1_20:3)	-0.84	(-0.9--0.79)	**7.38E-157**	**2.81E-156**	-1.75	(-1.81--1.69)	**0.00E+00**	**0.00E+00**	0.72	(0.66-0.78)	**2.67E-105**	**4.48E-105**	0.10	(-0.01-0.21)	7.72E-02	1.01E-01
324	PC	PC(38:4) (b)	-1.09	(-1.16--1.03)	**3.24E-182**	**1.57E-181**	-1.82	(-1.89--1.75)	**0.00E+00**	**0.00E+00**	0.76	(0.69-0.83)	**2.59E-86**	**4.09E-86**	-0.17	(-0.31--0.04)	**1.28E-02**	**1.94E-02**
325	PC	PC(18:0_20:4)	0.08	(0.02-0.13)	**6.69E-03**	**7.36E-03**	-0.17	(-0.23--0.1)	**2.55E-07**	**2.71E-07**	-0.03	(-0.09-0.03)	3.60E-01	3.63E-01	0.18	(0.06-0.29)	**3.20E-03**	**5.32E-03**
326	PC	PC(38:5) (a)	-0.38	(-0.43--0.34)	**2.14E-57**	**3.98E-57**	-2.25	(-2.3--2.19)	**0.00E+00**	**0.00E+00**	1.56	(1.5-1.62)	**0.00E+00**	**0.00E+00**	0.35	(0.25-0.45)	**1.67E-11**	**8.44E-11**
327	PC	PC(38:5) (b)	-0.75	(-0.81--0.68)	**1.06E-89**	**2.39E-89**	-1.89	(-1.96--1.82)	**0.00E+00**	**0.00E+00**	0.88	(0.81-0.95)	**8.88E-115**	**1.57E-114**	0.22	(0.07-0.37)	**3.13E-03**	**5.22E-03**
328	PC	PC(38:6) (a)	-0.09	(-0.15--0.04)	**7.41E-04**	**8.41E-04**	-2.88	(-2.95--2.82)	**0.00E+00**	**0.00E+00**	2.42	(2.35-2.48)	**0.00E+00**	**0.00E+00**	0.60	(0.48-0.72)	**3.53E-22**	**7.07E-21**
329	PC	PC(16:0_22:6)	-0.86	(-0.92--0.8)	**1.05E-134**	**3.38E-134**	-1.64	(-1.71--1.57)	**1.44E-269**	**2.49E-269**	0.34	(0.26-0.41)	**1.95E-17**	**2.27E-17**	0.47	(0.33-0.61)	**1.04E-10**	**4.77E-10**
330	PC	PC(18:2_20:5)	0.15	(0.06-0.24)	**7.21E-04**	**8.20E-04**	-3.25	(-3.34--3.16)	**0.00E+00**	**0.00E+00**	2.81	(2.72-2.9)	**0.00E+00**	**0.00E+00**	0.86	(0.67-1.05)	**9.17E-18**	**1.16E-16**
331	PC	PC(16:1_22:6)	-0.71	(-0.77--0.65)	**1.23E-96**	**2.96E-96**	-1.73	(-1.79--1.66)	**0.00E+00**	**0.00E+00**	0.55	(0.49-0.61)	**1.56E-57**	**2.20E-57**	0.50	(0.37-0.64)	**4.27E-13**	**2.60E-12**
332	PC	PC(38:7)(c)	-0.84	(-0.89--0.79)	**3.32E-161**	**1.33E-160**	-1.90	(-1.96--1.85)	**0.00E+00**	**0.00E+00**	0.67	(0.62-0.73)	**1.37E-102**	**2.29E-102**	0.44	(0.33-0.56)	**7.56E-14**	**5.11E-13**
333	PC	PC(39:5)(a)	-0.54	(-0.62--0.47)	**2.59E-45**	**4.43E-45**	-2.18	(-2.26--2.09)	**0.00E+00**	**0.00E+00**	1.25	(1.17-1.34)	**5.89E-137**	**1.12E-136**	0.47	(0.31-0.63)	**1.97E-08**	**6.82E-08**
334	PC	PC(39:5)(b)	-0.67	(-0.74--0.6)	**7.58E-77**	**1.58E-76**	-2.24	(-2.32--2.17)	**0.00E+00**	**0.00E+00**	1.24	(1.16-1.32)	**2.06E-149**	**4.13E-149**	0.36	(0.21-0.52)	**3.72E-06**	**9.66E-06**
335	PC	PC(15-MHDA_22:6)	-0.46	(-0.53--0.39)	**5.16E-36**	**8.18E-36**	-2.17	(-2.25--2.08)	**0.00E+00**	**0.00E+00**	1.42	(1.34-1.5)	**3.56E-166**	**7.66E-166**	0.38	(0.22-0.53)	**1.56E-06**	**4.33E-06**
336	PC	PC(17:0_22:6)	-0.79	(-0.85--0.72)	**5.46E-99**	**1.35E-98**	-2.21	(-2.28--2.14)	**0.00E+00**	**0.00E+00**	0.98	(0.9-1.05)	**9.36E-111**	**1.63E-110**	0.48	(0.32-0.63)	**1.54E-09**	**6.12E-09**
337	PC	PC(18:0_22:5) (n6)	-0.23	(-0.3--0.16)	**1.02E-10**	**1.29E-10**	-1.78	(-1.88--1.67)	**2.15E-176**	**3.01E-176**	1.51	(1.4-1.62)	**5.45E-123**	**9.98E-123**	-0.11	(-0.26-0.03)	1.30E-01	1.64E-01
338	PC	PC(18:0_22:6)	-0.53	(-0.6--0.46)	**2.24E-51**	**3.99E-51**	-1.30	(-1.37--1.23)	**2.04E-187**	**2.90E-187**	0.41	(0.34-0.49)	**1.16E-25**	**1.42E-25**	0.16	(0.01-0.31)	**3.26E-02**	**4.57E-02**
339	PC	PC(40:7) (a)	-0.48	(-0.54--0.42)	**4.15E-53**	**7.47E-53**	-1.15	(-1.23--1.08)	**3.90E-149**	**5.20E-149**	0.47	(0.39-0.56)	**6.30E-29**	**7.81E-29**	0.33	(0.2-0.46)	**1.32E-06**	**3.67E-06**
340	PC	PC(18:1_22:6) (a)	-0.84	(-0.89--0.79)	**2.27E-169**	**1.03E-168**	-1.52	(-1.58--1.46)	**1.11E-285**	**1.99E-285**	0.36	(0.29-0.43)	**2.24E-25**	**2.75E-25**	0.38	(0.27-0.5)	**1.32E-10**	**5.89E-10**
341	PC	PC(40:8)	-0.29	(-0.34--0.24)	**7.54E-32**	**1.15E-31**	-1.51	(-1.57--1.45)	**3.78E-308**	**7.12E-308**	0.97	(0.91-1.03)	**8.28E-162**	**1.75E-161**	0.58	(0.48-0.69)	**5.20E-27**	**3.57E-25**
342	PC	PC(44:12)	-0.71	(-0.81--0.61)	**6.98E-41**	**1.15E-40**	-1.77	(-1.86--1.67)	**3.52E-195**	**5.14E-195**	0.48	(0.39-0.58)	**2.03E-23**	**2.44E-23**	0.96	(0.73-1.18)	**2.11E-16**	**2.02E-15**
343	PC(O)	PC(O-16:0/16:0)	-0.22	(-0.26--0.18)	**6.69E-29**	**9.97E-29**	-0.74	(-0.79--0.69)	**1.35E-131**	**1.75E-131**	0.47	(0.42-0.52)	**3.83E-59**	**5.49E-59**	0.22	(0.14-0.3)	**1.00E-07**	**3.15E-07**
344	PC(O)	PC(O-32:1)	-0.43	(-0.48--0.38)	**8.01E-59**	**1.50E-58**	-0.97	(-1.03--0.91)	**8.24E-154**	**1.11E-153**	0.25	(0.19-0.32)	**1.53E-15**	**1.73E-15**	0.41	(0.31-0.52)	**4.38E-14**	**3.14E-13**
345	PC(O)	PC(O-34:1)	-0.23	(-0.27--0.2)	**1.82E-31**	**2.77E-31**	-1.34	(-1.39--1.29)	**9.28E-303**	**1.72E-302**	1.08	(1.02-1.13)	**1.57E-211**	**4.43E-211**	0.25	(0.17-0.33)	**1.48E-09**	**5.94E-09**
346	PC(O)	PC(O-34:2)	-0.31	(-0.37--0.26)	**2.84E-27**	**4.16E-27**	-3.75	(-3.81--3.68)	**0.00E+00**	**0.00E+00**	3.18	(3.11-3.25)	**0.00E+00**	**0.00E+00**	0.61	(0.49-0.73)	**1.12E-21**	**2.07E-20**
347	PC(O)	PC(O-34:4)	0.03	(-0.06-0.12)	5.39E-01	5.51E-01	-2.50	(-2.59--2.41)	**0.00E+00**	**0.00E+00**	1.35	(1.26-1.43)	**1.43E-160**	**3.00E-160**	1.18	(0.98-1.38)	**6.55E-29**	**5.24E-27**
348	PC(O)	PC(O-35:4)	0.06	(-0.02-0.14)	1.70E-01	1.79E-01	-2.95	(-3.03--2.87)	**0.00E+00**	**0.00E+00**	2.14	(2.06-2.21)	**9.61E-299**	**4.19E-298**	0.98	(0.79-1.17)	**1.47E-23**	**3.72E-22**
349	PC(O)	PC(O-36:0)	-0.27	(-0.31--0.22)	**1.86E-30**	**2.79E-30**	-1.47	(-1.53--1.4)	**1.41E-260**	**2.35E-260**	1.17	(1.1-1.24)	**2.62E-172**	**5.88E-172**	0.17	(0.07-0.26)	**7.04E-04**	**1.29E-03**
350	PC(O)	PC(O-18:0/18:1)	-0.13	(-0.18--0.09)	**3.79E-10**	**4.75E-10**	-1.39	(-1.44--1.33)	**2.54E-304**	**4.72E-304**	1.29	(1.24-1.35)	**2.14E-264**	**7.83E-264**	0.21	(0.13-0.3)	**1.15E-06**	**3.26E-06**
351	PC(O)	PC(O-18:1/18:1)	-0.11	(-0.15--0.07)	**2.29E-07**	**2.82E-07**	-1.85	(-1.9--1.79)	**0.00E+00**	**0.00E+00**	1.63	(1.58-1.68)	**0.00E+00**	**0.00E+00**	0.35	(0.27-0.43)	**7.87E-17**	**8.21E-16**
352	PC(O)	PC(O-18:0/18:2)	-0.19	(-0.24--0.14)	**2.28E-13**	**2.98E-13**	-3.22	(-3.28--3.16)	**0.00E+00**	**0.00E+00**	2.91	(2.85-2.97)	**0.00E+00**	**0.00E+00**	0.43	(0.33-0.53)	**2.15E-16**	**2.02E-15**
353	PC(O)	PC(O-18:1/18:2)	-0.25	(-0.3--0.2)	**7.58E-23**	**1.08E-22**	-3.20	(-3.26--3.14)	**0.00E+00**	**0.00E+00**	2.76	(2.7-2.82)	**0.00E+00**	**0.00E+00**	0.46	(0.36-0.56)	**9.17E-18**	**1.16E-16**
354	PC(O)	PC(O-16:0/20:3)	-0.31	(-0.35--0.26)	**9.20E-35**	**1.45E-34**	-1.88	(-1.93--1.82)	**0.00E+00**	**0.00E+00**	1.48	(1.42-1.54)	**1.79E-273**	**6.91E-273**	0.16	(0.06-0.27)	**2.20E-03**	**3.79E-03**
355	PC(O)	PC(O-16:0/20:4)	-0.16	(-0.21--0.11)	**2.52E-11**	**3.24E-11**	-1.48	(-1.54--1.43)	**0.00E+00**	**0.00E+00**	1.16	(1.11-1.22)	**3.97E-223**	**1.19E-222**	0.28	(0.18-0.38)	**1.28E-07**	**3.96E-07**
356	PC(O)	PC(O-36:5)	0.12	(0.03-0.21)	**9.10E-03**	**9.93E-03**	-3.21	(-3.3--3.12)	**0.00E+00**	**0.00E+00**	2.57	(2.49-2.66)	**0.00E+00**	**0.00E+00**	0.88	(0.67-1.08)	**4.92E-17**	**5.37E-16**
357	PC(O)	PC(O-18:0/20:4)	-0.02	(-0.07-0.02)	3.17E-01	3.31E-01	-1.05	(-1.11--1)	**1.39E-220**	**2.12E-220**	1.06	(1-1.11)	**8.12E-209**	**2.24E-208**	0.12	(0.02-0.21)	**1.54E-02**	**2.28E-02**

358	PC(O)	PC(O-38:5)	-0.17	(-0.22--0.13)	3.06E-15	4.11E-15	-2.11	(-2.16--2.05)	0.00E+00	0.00E+00	1.87	(1.81-1.93)	0.00E+00	0.00E+00	0.21	(0.11-0.3)	2.85E-05	6.63E-05
359	PC(O)	PC(O-16:0/22:6)	-0.59	(-0.66--0.53)	9.17E-70	1.85E-69	-1.97	(-2.03--1.9)	0.00E+00	0.00E+00	1.02	(0.95-1.08)	2.91E-149	5.80E-149	0.42	(0.28-0.55)	1.92E-09	7.31E-09
360	PC(O)	PC(O-40:5)	-0.26	(-0.3--0.22)	4.35E-31	6.54E-31	-1.86	(-1.91--1.81)	0.00E+00	0.00E+00	1.57	(1.52-1.62)	0.00E+00	0.00E+00	0.18	(0.09-0.27)	8.69E-05	1.83E-04
361	PC(O)	PC(O-18:0/22:6)	-0.24	(-0.29--0.19)	1.62E-20	2.26E-20	-1.41	(-1.46--1.35)	1.11E-301	2.04E-301	1.06	(1.01-1.12)	2.81E-204	7.49E-204	0.23	(0.12-0.34)	2.33E-05	5.45E-05
362	PC(O)	PC(O-40:7) (a)	-0.44	(-0.5--0.38)	2.68E-47	4.65E-47	-2.21	(-2.27--2.15)	0.00E+00	0.00E+00	1.50	(1.43-1.56)	4.78E-261	1.69E-260	0.41	(0.29-0.53)	1.01E-10	4.68E-10
363	PC(P)	PC(P-16:0/14:0)	-0.24	(-0.3--0.19)	1.37E-17	1.88E-17	-0.93	(-1--0.86)	2.72E-131	3.52E-131	0.44	(0.37-0.51)	5.12E-34	6.50E-34	0.34	(0.22-0.47)	9.59E-08	3.03E-07
364	PC(P)	PC(P-16:0/16:0)	-0.03	(-0.08-0.01)	1.69E-01	1.78E-01	-0.76	(-0.82--0.7)	8.53E-115	1.06E-114	0.53	(0.47-0.59)	1.17E-60	1.69E-60	0.40	(0.3-0.5)	1.20E-15	1.05E-14
365	PC(P)	PC(P-16:0/16:1)	-0.33	(-0.39--0.28)	7.48E-34	1.17E-33	-0.83	(-0.9--0.77)	1.91E-115	2.39E-115	0.01	(-0.05-0.08)	6.54E-01	6.57E-01	0.59	(0.49-0.7)	2.98E-25	1.22E-23
366	PC(P)	PC(P-16:0/18:0)	-0.01	(-0.05-0.02)	5.18E-01	5.33E-01	-0.84	(-0.89--0.79)	1.83E-190	2.62E-190	0.75	(0.71-0.8)	1.69E-157	3.48E-157	0.31	(0.24-0.38)	9.77E-17	9.77E-16
367	PC(P)	PC(P-16:0/18:1)	0.02	(-0.03-0.06)	4.94E-01	5.09E-01	-0.93	(-0.99--0.87)	2.02E-164	2.79E-164	0.66	(0.6-0.71)	1.19E-104	1.98E-104	0.59	(0.49-0.68)	1.53E-31	2.44E-29
368	PC(P)	PC(P-16:0/18:2)	-0.09	(-0.14--0.04)	4.96E-04	5.67E-04	-3.52	(-3.59--3.46)	0.00E+00	0.00E+00	3.27	(3.2-3.33)	0.00E+00	0.00E+00	0.42	(0.31-0.53)	7.54E-14	5.11E-13
369	PC(P)	PC(P-16:0/18:3)	0.01	(-0.04-0.07)	6.07E-01	6.18E-01	-3.03	(-3.09--2.97)	0.00E+00	0.00E+00	2.81	(2.76-2.87)	0.00E+00	0.00E+00	0.40	(0.28-0.52)	3.29E-11	1.60E-10
370	PC(P)	PC(P-35:2) (a)	-0.20	(-0.28--0.13)	3.58E-07	4.37E-07	-3.72	(-3.8--3.63)	0.00E+00	0.00E+00	3.32	(3.24-3.4)	0.00E+00	0.00E+00	0.41	(0.23-0.58)	5.24E-06	1.33E-05
371	PC(P)	PC(P-35:2) (b)	-0.11	(-0.17--0.04)	1.00E-03	1.13E-03	-4.04	(-4.11--3.96)	0.00E+00	0.00E+00	3.68	(3.61-3.75)	0.00E+00	0.00E+00	0.57	(0.43-0.71)	4.06E-15	3.14E-14
372	PC(P)	PC(P-15:0/20:4) (b)	0.09	(0.03-0.16)	4.04E-03	4.50E-03	-1.60	(-1.68--1.53)	3.95E-255	6.42E-255	1.45	(1.37-1.52)	5.33E-199	1.39E-198	0.25	(0.1-0.4)	1.04E-03	1.85E-03
373	PC(P)	PC(P-18:1/18:1)	-0.06	(-0.11--0.02)	8.60E-03	9.45E-03	-1.13	(-1.2--1.07)	7.07E-188	1.01E-187	0.84	(0.78-0.9)	4.16E-117	7.43E-117	0.47	(0.37-0.56)	1.53E-21	2.71E-20
374	PC(P)	PC(P-18:0/18:2)	-0.02	(-0.07-0.03)	4.30E-01	4.45E-01	-3.29	(-3.35--3.23)	0.00E+00	0.00E+00	3.20	(3.15-3.26)	0.00E+00	0.00E+00	0.40	(0.29-0.5)	1.96E-13	1.26E-12
375	PC(P)	PC(P-16:0/20:4)	-0.12	(-0.18--0.07)	1.98E-06	2.37E-06	-1.22	(-1.28--1.16)	7.29E-221	1.11E-220	1.00	(0.94-1.06)	4.31E-166	9.24E-166	0.22	(0.11-0.33)	4.94E-05	1.09E-04
376	PC(P)	PC(P-16:0/20:5)	0.19	(0.09-0.28)	9.83E-05	1.14E-04	-2.98	(-3.08--2.88)	0.00E+00	0.00E+00	2.55	(2.46-2.64)	0.00E+00	0.00E+00	0.74	(0.53-0.94)	5.19E-12	2.86E-11
377	PC(P)	PC(P-17:0/20:4) (a)	-0.12	(-0.19--0.05)	7.93E-04	8.98E-04	-1.95	(-2.03--1.87)	2.55E-296	4.68E-296	1.73	(1.65-1.81)	3.43E-222	1.02E-221	0.21	(0.05-0.38)	1.25E-02	1.92E-02
378	PC(P)	PC(P-17:0/20:4) (b)	0.10	(0.03-0.16)	2.56E-03	2.87E-03	-1.72	(-1.79--1.64)	9.54E-280	1.68E-279	1.59	(1.52-1.66)	1.15E-236	3.64E-236	0.37	(0.23-0.52)	2.25E-07	6.72E-07
379	PC(P)	PC(P-18:0/20:4)	0.19	(0.14-0.24)	3.64E-14	4.80E-14	-1.06	(-1.12--1)	1.24E-199	1.82E-199	1.28	(1.22-1.33)	1.18E-244	3.89E-244	0.14	(0.03-0.24)	1.29E-02	1.96E-02
380	PC(P)	PC(P-38:5) (a)	-0.08	(-0.13--0.03)	3.08E-03	3.44E-03	-2.01	(-2.07--1.94)	0.00E+00	0.00E+00	1.68	(1.62-1.74)	0.00E+00	0.00E+00	0.33	(0.22-0.44)	1.78E-09	6.95E-09
381	PC(P)	PC(P-38:5) (b)	-0.21	(-0.26--0.16)	4.77E-15	6.36E-15	-1.38	(-1.45--1.32)	1.77E-250	2.84E-250	1.12	(1.05-1.18)	1.92E-171	4.28E-171	0.16	(0.04-0.27)	7.20E-03	1.15E-02
382	PC(P)	PC(P-16:0/22:6)	-0.45	(-0.51--0.39)	3.89E-42	6.47E-42	-1.88	(-1.95--1.81)	0.00E+00	0.00E+00	1.17	(1.1-1.24)	1.05E-173	2.39E-173	0.38	(0.25-0.52)	3.12E-08	1.03E-07
383	PC(P)	PC(P-20:0/20:4)	-0.18	(-0.23--0.13)	1.57E-12	2.04E-12	-0.96	(-1.02--0.9)	5.29E-155	7.11E-155	0.97	(0.91-1.03)	1.31E-157	2.72E-157	0.03	(-0.08-0.13)	6.27E-01	6.84E-01
384	PC(P)	PC(P-40:5) (b)	-0.05	(-0.1-0)	6.14E-02	6.55E-02	-1.24	(-1.3--1.19)	5.33E-259	8.82E-259	1.17	(1.12-1.23)	7.56E-243	2.47E-242	0.21	(0.11-0.31)	4.11E-05	9.34E-05
385	PC(P)	PC(P-18:0/22:6)	-0.01	(-0.07-0.05)	7.22E-01	7.29E-01	-1.80	(-1.87--1.74)	0.00E+00	0.00E+00	1.73	(1.67-1.79)	1.80E-305	8.23E-305	0.26	(0.13-0.39)	1.39E-04	2.83E-04
386	PE	PE(16:0_16:1)	-3.16	(-3.27--3.05)	0.00E+00	0.00E+00	-3.71	(-3.82--3.6)	0.00E+00	0.00E+00	0.13	(0.02-0.23)	1.90E-02	1.96E-02	0.11	(-0.13-0.35)	3.86E-01	4.44E-01
387	PE	PE(16:0_18:1)	-2.76	(-2.83--2.68)	0.00E+00	0.00E+00	-4.34	(-4.42--4.27)	0.00E+00	0.00E+00	1.25	(1.17-1.33)	1.59E-153	3.24E-153	0.24	(0.06-0.42)	7.61E-03	1.21E-02
388	PE	PE(16:0_18:2)	-2.98	(-3.06--2.89)	0.00E+00	0.00E+00	-5.25	(-5.34--5.17)	0.00E+00	0.00E+00	1.88	(1.79-1.96)	2.99E-239	9.62E-239	0.40	(0.21-0.6)	4.67E-05	1.04E-04
389	PE	PE(16:1_18:2)	-2.41	(-2.5--2.33)	0.00E+00	0.00E+00	-3.23	(-3.33--3.14)	0.00E+00	0.00E+00	0.53	(0.46-0.61)	2.56E-40	3.36E-40	0.27	(0.11-0.44)	1.41E-03	2.47E-03
390	PE	PE(16:0_18:3) (a)	-2.95	(-3.04--2.86)	0.00E+00	0.00E+00	-4.63	(-4.72--4.55)	0.00E+00	0.00E+00	1.48	(1.4-1.57)	1.35E-173	3.05E-173	0.18	(-0.02-0.37)	7.25E-02	9.58E-02
391	PE	PE(16:0_18:3) (b)	-2.00	(-2.09--1.91)	6.60E-260	5.98E-259	-2.95	(-3.04--2.86)	0.00E+00	0.00E+00	0.85	(0.76-0.94)	1.43E-63	2.07E-63	-0.05	(-0.26-0.16)	6.25E-01	6.83E-01
392	PE	PE(17:0_18:2)	-2.10	(-2.16--2.03)	0.00E+00	0.00E+00	-3.73	(-3.79--3.66)	0.00E+00	0.00E+00	1.36	(1.3-1.43)	7.17E-222	2.11E-221	0.26	(0.1-0.41)	1.42E-03	2.49E-03
393	PE	PE(18:0_18:1)	-2.12	(-2.19--2.05)	0.00E+00	0.00E+00	-4.63	(-4.7--4.56)	0.00E+00	0.00E+00	2.21	(2.13-2.29)	0.00E+00	0.00E+00	0.09	(-0.08-0.26)	2.76E-01	3.28E-01
394	PE	PE(18:1_18:1)	-1.88	(-1.95--1.82)	0.00E+00	0.00E+00	-3.26	(-3.34--3.17)	0.00E+00	0.00E+00	1.24	(1.15-1.34)	4.58E-113	8.05E-113	0.11	(-0.05-0.26)	1.76E-01	2.16E-01
395	PE	PE(18:0_18:2)	-1.92	(-1.99--1.85)	0.00E+00	0.00E+00	-5.13	(-5.2--5.06)	0.00E+00	0.00E+00	2.84	(2.76-2.91)	0.00E+00	0.00E+00	0.21	(0.04-0.38)	1.61E-02	2.38E-02
396	PE	PE(18:1_18:2)	-2.56	(-2.64--2.48)	0.00E+00	0.00E+00	-5.16	(-5.24--5.08)	0.00E+00	0.00E+00	2.34	(2.25-2.42)	0.00E+00	0.00E+00	0.39	(0.21-0.58)	3.89E-05	8.98E-05
397	PE	PE(16:0_20:3)	-2.70	(-2.78--2.62)	0.00E+00	0.00E+00	-3.65	(-3.73--3.56)	0.00E+00	0.00E+00	0.68	(0.6-0.76)	4.27E-53	5.84E-53	0.19	(0.01-0.38)	4.19E-02	5.78E-02
398	PE	PE(16:0_20:4)	-2.49	(-2.56--2.43)	0.00E+00	0.00E+00	-2.89	(-2.96--2.82)	0.00E+00	0.00E+00	0.00	(-0.08-0.07)	9.25E-01	9.25E-01	0.46	(0.3-0.63)	5.04E-08	1.62E-07
399	PE	PE(16:1_20:4)	-1.96	(-2.03--1.88)	0.00E+00	0.00E+00	-1.86	(-1.94--1.77)	1.33E-254	2.15E-254	-0.38	(-0.45--0.31)	2.17E-23	2.60E-23	0.30	(0.16-0.45)	4.86E-05	1.08E-04
400	PE	PE(16:0_20:5)	-1.94	(-2.03--1.85)	8.70E-233	6.05E-232	-3.28	(-3.37--3.18)	0.00E+00	0.00E+00	0.65	(0.56-0.73)	1.25E-45	1.67E-45	0.75	(0.55-0.95)	2.55E-13	1.61E-12
401	PE	PE(17:0_20:4)	-1.81	(-1.88--1.75)	0.00E+00	0.00E+00	-2.77	(-2.84--2.7)	0.00E+00	0.00E+00	0.58	(0.51-0.65)	1.70E-50	2.31E-50	0.36	(0.21-0.51)	3.55E-06	9.37E-06
402	PE	PE(18:0_20:3) (a)	-1.88	(-1.96--1.8)	1.97E-292	2.56E-291	-2.47	(-2.55--2.4)	0.00E+00	0.00E+00	0.25	(0.18-0.32)	3.15E-11	3.47E-11	-0.04	(-0.21-0.13)	6.43E-01	6.97E-01
403	PE	PE(18:0_20:4)	-1.43	(-1.49--1.37)	1.38E-288	1.65E-287	-2.29	(-2.35--2.23)	0.00E+00	0.00E+00	0.40	(0.34-0.47)	6.36E-32	7.93E-32	0.35	(0.22-0.49)	7.02E-07	2.03E-06
404	PE	PE(38:5) (a)	-2.33	(-2.4--2.26)	0.00E+00	0.00E+00	-3.31	(-3.38--3.24)	0.00E+00	0.00E+00	0.54	(0.47-0.61)	1.97E-44	2.62E-44	0.53	(0.37-0.69)	2.90E-10	1.24E-09
405	PE	PE(38:5) (b)	-2.22	(-2.3--2.14)	0.00E+00	0.00E+00	-3.76	(-3.85--3.67)	0.00E+00	0.00E+00	1.10	(1-1.2)	9.71E-90	1.55E-89	0.44	(0.25-0.62)	4.32E-06	1.12E-05
406	PE	PE(16:0_22:6)	-2.61	(-2.69--2.54)	0.00E+00	0.00E+00	-3.78	(-3.86--3.71)	0.00E+00	0.00E+00	0.35	(0.27-0.44)	6.39E-16	7.29E-16	0.84	(0.65-1.03)	1.03E-17	1.25E-16
407	PE	PE(17:0_22:6)	-1.87	(-1.94--1.8)	0.00E+00	0.00E+00	-2.84	(-2.91--2.76)	0.00E+00	0.00E+00	0.40	(0.32-0.47)	2.59E-26	3.19E-26	0.55	(0.39-0.7)	6.97E-12	3.80E-11
408	PE	PE(18:0_22:4)	-1.76	(-1.83--1.68)	7.99E-281	8.92E-280	-2.46	(-2.54--2.37)	0.00E+00	0.00E+00	0.61	(0.53-0.68)	2.33E-50	3.16E-50	-0.16	(-0.31--0.01)	3.94E-02	5.50E-02
409	PE	PE(18:0_22:5) (n3)	-1.78	(-1.85--1.71)	0.00E+00	0.00E+00	-3.49	(-3.57--3.41)	0.00E+00	0.00E+00	1.18	(1.09-1.26)	3.64E-129	6.83E-129	0.28	(0.11-0.44)	8.26E-04	1.51E-03

410	PE	PE(18:0_22:5) (n6)	-1.87	(-1.95--1.78)	**7.23E-262**	**6.80E-261**	-3.05	(-3.14--2.95)	**0.00E+00**	**0.00E+00**	0.93	(0.84-1.03)	**4.94E-72**	**7.46E-72**	0.07	(-0.11-0.25)	4.50E-01	5.09E-01
411	PE	PE(18:0_22:6)	-2.01	(-2.09--1.94)	**0.00E+00**	**0.00E+00**	-3.62	(-3.7--3.53)	**0.00E+00**	**0.00E+00**	0.68	(0.59-0.77)	**5.40E-46**	**7.24E-46**	0.58	(0.4-0.76)	**6.69E-10**	**2.77E-09**
412	PE	PE(40:7)	-3.13	(-3.21--3.05)	**0.00E+00**	**0.00E+00**	-4.36	(-4.44--4.28)	**0.00E+00**	**0.00E+00**	0.44	(0.35-0.52)	**8.83E-23**	**1.05E-22**	0.90	(0.7-1.09)	**1.56E-18**	**2.14E-17**
413	PE(O)	PE(O-34:1)	-0.09	(-0.14--0.03)	**1.42E-03**	**1.60E-03**	-3.47	(-3.56--3.39)	**0.00E+00**	**0.00E+00**	3.52	(3.43-3.61)	**0.00E+00**	**0.00E+00**	0.15	(0.03-0.27)	**1.27E-02**	**1.94E-02**
414	PE(O)	PE(O-16:0/18:2)	-0.16	(-0.23--0.08)	**2.18E-05**	**2.58E-05**	-5.76	(-5.9--5.62)	**0.00E+00**	**0.00E+00**	5.48	(5.33-5.63)	**0.00E+00**	**0.00E+00**	0.54	(0.38-0.69)	**3.62E-11**	**1.74E-10**
415	PE(O)	PE(O-18:1/18:2)	0.06	(0-0.13)	5.95E-02	6.36E-02	-3.00	(-3.09--2.91)	**0.00E+00**	**0.00E+00**	2.90	(2.82-2.99)	**0.00E+00**	**0.00E+00**	0.32	(0.18-0.46)	**8.78E-06**	**2.15E-05**
416	PE(O)	PE(O-16:0/20:4)	0.14	(0.07-0.21)	**4.57E-05**	**5.37E-05**	-3.07	(-3.15--2.99)	**0.00E+00**	**0.00E+00**	3.02	(2.93-3.1)	**0.00E+00**	**0.00E+00**	0.35	(0.2-0.5)	**5.37E-06**	**1.35E-05**
417	PE(O)	PE(O-36:5)	-0.22	(-0.32--0.12)	**2.76E-05**	**3.24E-05**	-3.40	(-3.5--3.31)	**0.00E+00**	**0.00E+00**	2.50	(2.42-2.58)	**0.00E+00**	**0.00E+00**	0.85	(0.64-1.07)	**1.58E-14**	**1.18E-13**
418	PE(O)	PE(O-16:0/22:4)	-0.09	(-0.14--0.04)	**8.50E-04**	**9.60E-04**	-1.25	(-1.32--1.19)	**8.24E-219**	**1.25E-218**	1.15	(1.08-1.21)	**3.93E-181**	**9.20E-181**	0.10	(-0.02-0.21)	9.50E-02	1.24E-01
419	PE(O)	PE(O-18:0/20:4)	0.22	(0.14-0.29)	**6.21E-09**	**7.72E-09**	-2.78	(-2.87--2.7)	**0.00E+00**	**0.00E+00**	2.80	(2.71-2.88)	**0.00E+00**	**0.00E+00**	0.33	(0.17-0.5)	**5.81E-05**	**1.26E-04**
420	PE(O)	PE(O-38:5) (a)	0.16	(0.1-0.23)	**6.00E-07**	**7.30E-07**	-3.65	(-3.72--3.57)	**0.00E+00**	**0.00E+00**	3.64	(3.57-3.71)	**0.00E+00**	**0.00E+00**	0.29	(0.14-0.43)	**1.07E-04**	**2.20E-04**
421	PE(O)	PE(O-38:5) (b)	-0.07	(-0.14--0.01)	**3.08E-02**	**3.31E-02**	-2.25	(-2.33--2.17)	**0.00E+00**	**0.00E+00**	2.02	(1.94-2.1)	**3.65E-280**	**1.46E-279**	0.34	(0.2-0.49)	**3.61E-06**	**9.47E-06**
422	PE(O)	PE(O-16:0/22:6)	-0.22	(-0.28--0.16)	**4.71E-13**	**6.12E-13**	-2.92	(-2.99--2.85)	**0.00E+00**	**0.00E+00**	2.48	(2.41-2.55)	**0.00E+00**	**0.00E+00**	0.42	(0.28-0.55)	**1.18E-09**	**4.79E-09**
423	PE(O)	PE(O-18:0/22:6)	0.08	(0.02-0.13)	**8.79E-03**	**9.64E-03**	-2.28	(-2.34--2.22)	**0.00E+00**	**0.00E+00**	2.21	(2.15-2.27)	**0.00E+00**	**0.00E+00**	0.31	(0.19-0.44)	**1.05E-06**	**2.97E-06**
424	PE(O)	PE(O-18:1/22:6)	-0.11	(-0.16--0.05)	**9.61E-05**	**1.12E-04**	-2.57	(-2.63--2.51)	**0.00E+00**	**0.00E+00**	2.29	(2.23-2.35)	**0.00E+00**	**0.00E+00**	0.36	(0.24-0.49)	**1.06E-08**	**3.79E-08**
425	PE(P)	PE(P-16:0/18:1)	-0.08	(-0.13--0.04)	**5.41E-04**	**6.17E-04**	-1.29	(-1.35--1.23)	**7.45E-263**	**1.25E-262**	1.63	(1.58-1.69)	**0.00E+00**	**0.00E+00**	-0.27	(-0.37--0.17)	**1.20E-07**	**3.73E-07**
426	PE(P)	PE(P-16:0/18:2)	-0.17	(-0.23--0.11)	**8.40E-08**	**1.03E-07**	-4.68	(-4.75--4.6)	**0.00E+00**	**0.00E+00**	4.76	(4.68-4.84)	**0.00E+00**	**0.00E+00**	-0.13	(-0.26-0)	5.15E-02	6.94E-02
427	PE(P)	PE(P-16:0/20:3)	-0.08	(-0.14--0.01)	**1.70E-02**	**1.84E-02**	-2.07	(-2.15--2)	**0.00E+00**	**0.00E+00**	2.32	(2.25-2.4)	**0.00E+00**	**0.00E+00**	-0.48	(-0.62--0.35)	**3.27E-12**	**1.82E-11**
428	PE(P)	PE(P-16:0/20:4)	-0.01	(-0.07-0.06)	7.69E-01	7.73E-01	-2.82	(-2.89--2.74)	**0.00E+00**	**0.00E+00**	2.91	(2.84-2.99)	**0.00E+00**	**0.00E+00**	-0.12	(-0.26-0.02)	1.05E-01	1.37E-01
429	PE(P)	PE(P-16:0/20:5)	-0.04	(-0.16-0.08)	5.20E-01	5.33E-01	-6.00	(-6.16--5.84)	**0.00E+00**	**0.00E+00**	5.55	(5.38-5.72)	**0.00E+00**	**0.00E+00**	0.46	(0.2-0.71)	**3.95E-04**	**7.46E-04**
430	PE(P)	PE(P-16:0/22:4)	-0.23	(-0.28--0.18)	**1.31E-19**	**1.82E-19**	0.01	(-0.05-0.07)	7.03E-01	7.04E-01	0.32	(0.26-0.38)	**8.21E-25**	**1.00E-24**	-0.51	(-0.61--0.4)	**6.69E-21**	**1.11E-19**
431	PE(P)	PE(P-16:0/22:5) (n3)	-0.05	(-0.1-0)	**4.44E-02**	**4.77E-02**	-2.95	(-3.01--2.88)	**0.00E+00**	**0.00E+00**	2.98	(2.92-3.05)	**0.00E+00**	**0.00E+00**	-0.09	(-0.21-0.02)	1.10E-01	1.41E-01
432	PE(P)	PE(P-16:0/22:5) (n6)	-0.38	(-0.43--0.32)	**1.15E-38**	**1.85E-38**	-1.26	(-1.33--1.19)	**4.86E-195**	**7.06E-195**	1.27	(1.2-1.34)	**3.45E-183**	**8.21E-183**	-0.35	(-0.47--0.22)	**2.90E-08**	**9.67E-08**
433	PE(P)	PE(P-16:0/22:6)	-0.43	(-0.48--0.38)	**7.08E-53**	**1.27E-52**	-2.16	(-2.22--2.1)	**0.00E+00**	**0.00E+00**	1.63	(1.57-1.69)	**1.15E-294**	**4.83E-294**	0.18	(0.06-0.29)	**2.49E-03**	**4.24E-03**
434	PE(P)	PE(P-17:0/20:4) (a)	0.17	(0.09-0.25)	**2.47E-05**	**2.91E-05**	-2.11	(-2.19--2.04)	**0.00E+00**	**0.00E+00**	2.04	(1.97-2.11)	**0.00E+00**	**0.00E+00**	0.22	(0.05-0.39)	**1.23E-02**	**1.90E-02**
435	PE(P)	PE(P-17:0/20:4) (b)	0.20	(0.13-0.27)	**3.57E-08**	**4.41E-08**	-2.39	(-2.47--2.32)	**0.00E+00**	**0.00E+00**	2.30	(2.23-2.37)	**0.00E+00**	**0.00E+00**	0.35	(0.19-0.5)	**9.42E-06**	**2.30E-05**
436	PE(P)	PE(P-17:0/22:6) (a)	-0.17	(-0.24--0.1)	**4.70E-06**	**5.61E-06**	-1.94	(-2.02--1.86)	**6.55E-292**	**1.19E-291**	1.47	(1.4-1.55)	**1.75E-192**	**4.38E-192**	0.34	(0.18-0.5)	**4.82E-05**	**1.07E-04**
437	PE(P)	PE(P-17:0/22:6) (b)	-0.13	(-0.2--0.07)	**1.12E-04**	**1.29E-04**	-2.45	(-2.52--2.37)	**0.00E+00**	**0.00E+00**	1.98	(1.91-2.05)	**0.00E+00**	**0.00E+00**	0.47	(0.32-0.62)	**6.51E-10**	**2.72E-09**
438	PE(P)	PE(P-18:0/18:1)	-0.02	(-0.08-0.03)	4.12E-01	4.27E-01	-2.96	(-3.03--2.89)	**0.00E+00**	**0.00E+00**	3.27	(3.21-3.34)	**0.00E+00**	**0.00E+00**	-0.19	(-0.31--0.08)	**8.92E-04**	**1.61E-03**
439	PE(P)	PE(P-18:0/18:2)	-0.07	(-0.13--0.01)	**1.37E-02**	**1.49E-02**	-5.89	(-5.97--5.81)	**0.00E+00**	**0.00E+00**	6.06	(5.98-6.14)	**0.00E+00**	**0.00E+00**	-0.03	(-0.16-0.1)	6.48E-01	7.01E-01
440	PE(P)	PE(P-18:0/20:3)	-0.03	(-0.09-0.03)	2.87E-01	3.00E-01	-2.33	(-2.39--2.27)	**0.00E+00**	**0.00E+00**	2.52	(2.47-2.58)	**0.00E+00**	**0.00E+00**	-0.28	(-0.4--0.17)	**1.71E-06**	**4.72E-06**
441	PE(P)	PE(P-18:0/20:4)	0.32	(0.26-0.39)	**7.50E-22**	**1.06E-21**	-2.97	(-3.05--2.89)	**0.00E+00**	**0.00E+00**	3.29	(3.22-3.36)	**0.00E+00**	**0.00E+00**	0.07	(-0.07-0.21)	3.26E-01	3.80E-01
442	PE(P)	PE(P-18:0/20:5)	0.17	(0.05-0.29)	**6.48E-03**	**7.15E-03**	-6.21	(-6.37--6.06)	**0.00E+00**	**0.00E+00**	5.80	(5.64-5.95)	**0.00E+00**	**0.00E+00**	0.61	(0.36-0.86)	**2.65E-06**	**7.14E-06**
443	PE(P)	PE(P-18:0/22:4)	-0.05	(-0.11-0)	**4.71E-02**	5.05E-02	-0.10	(-0.17--0.03)	**5.50E-03**	**5.70E-03**	0.83	(0.76-0.89)	**1.84E-105**	**3.09E-105**	-0.68	(-0.79--0.57)	**3.65E-31**	**4.37E-29**
444	PE(P)	PE(P-18:0/22:5) (n3)	0.22	(0.17-0.27)	**6.61E-17**	**9.01E-17**	-2.43	(-2.5--2.37)	**0.00E+00**	**0.00E+00**	2.79	(2.73-2.85)	**0.00E+00**	**0.00E+00**	-0.06	(-0.16-0.05)	3.15E-01	3.71E-01
445	PE(P)	PE(P-18:0/22:5) (n6)	-0.12	(-0.17--0.06)	**1.22E-05**	**1.45E-05**	-1.46	(-1.52--1.4)	**3.82E-283**	**6.85E-283**	1.69	(1.63-1.76)	**3.92E-301**	**1.74E-300**	-0.22	(-0.34--0.1)	**3.91E-04**	**7.41E-04**
446	PE(P)	PE(P-18:0/22:6)	0.01	(-0.05-0.07)	7.16E-01	7.25E-01	-2.50	(-2.57--2.44)	**0.00E+00**	**0.00E+00**	2.53	(2.47-2.59)	**0.00E+00**	**0.00E+00**	0.14	(0.01-0.27)	**2.89E-02**	**4.09E-02**
447	PE(P)	PE(P-18:1/18:1)	-0.17	(-0.21--0.12)	**6.16E-11**	**7.82E-11**	-2.39	(-2.45--2.33)	**0.00E+00**	**0.00E+00**	2.64	(2.58-2.69)	**0.00E+00**	**0.00E+00**	-0.26	(-0.36--0.15)	**1.81E-06**	**4.96E-06**
448	PE(P)	PE(P-18:1/18:2)	-0.22	(-0.28--0.15)	**3.59E-11**	**4.57E-11**	-5.92	(-6.01--5.82)	**0.00E+00**	**0.00E+00**	5.98	(5.88-6.08)	**0.00E+00**	**0.00E+00**	-0.06	(-0.2-0.07)	3.55E-01	4.12E-01
449	PE(P)	PE(P-18:1/20:4)	0.13	(0.06-0.19)	**9.45E-05**	**1.10E-04**	-3.18	(-3.26--3.1)	**0.00E+00**	**0.00E+00**	3.42	(3.34-3.49)	**0.00E+00**	**0.00E+00**	-0.04	(-0.18-0.1)	5.72E-01	6.33E-01
450	PE(P)	PE(P-18:1/20:5)	-0.26	(-0.38--0.14)	**1.73E-05**	**2.05E-05**	-6.95	(-7.13--6.76)	**0.00E+00**	**0.00E+00**	6.25	(6.06-6.45)	**0.00E+00**	**0.00E+00**	0.52	(0.27-0.78)	**5.75E-05**	**1.25E-04**
451	PE(P)	PE(P-18:1/22:4)	-0.20	(-0.25--0.14)	**3.07E-11**	**3.92E-11**	-0.20	(-0.27--0.12)	**2.70E-07**	**2.86E-07**	0.69	(0.62-0.76)	**4.72E-68**	**6.94E-68**	-0.58	(-0.7--0.46)	**2.22E-21**	**3.80E-20**
452	PE(P)	PE(P-18:1/22:5) (a)	0.08	(0.02-0.13)	**4.92E-03**	**5.45E-03**	-2.50	(-2.56--2.43)	**0.00E+00**	**0.00E+00**	2.66	(2.6-2.72)	**0.00E+00**	**0.00E+00**	0.02	(-0.09-0.14)	6.82E-01	7.29E-01
453	PE(P)	PE(P-18:1/22:6) (a)	-0.37	(-0.43--0.31)	**1.95E-33**	**3.02E-33**	-2.96	(-3.03--2.89)	**0.00E+00**	**0.00E+00**	2.56	(2.49-2.62)	**0.00E+00**	**0.00E+00**	0.17	(0.04-0.3)	**9.78E-03**	**1.53E-02**
454	PE(P)	PE(P-20:0/18:1)	-0.11	(-0.17--0.06)	**4.69E-05**	**5.49E-05**	-3.50	(-3.62--3.38)	**0.00E+00**	**0.00E+00**	4.02	(3.89-4.15)	**0.00E+00**	**0.00E+00**	-0.52	(-0.63--0.4)	**6.06E-18**	**8.09E-17**
455	PE(P)	PE(P-20:0/18:2)	-0.07	(-0.12--0.02)	**4.13E-03**	**4.58E-03**	-3.29	(-3.34--3.24)	**0.00E+00**	**0.00E+00**	3.81	(3.76-3.86)	**0.00E+00**	**0.00E+00**	-0.31	(-0.42--0.2)	**2.13E-08**	**7.31E-08**
456	PE(P)	PE(P-20:0/20:4)	0.42	(0.37-0.47)	**2.45E-54**	**4.42E-54**	-1.57	(-1.65--1.5)	**4.72E-235**	**7.42E-235**	2.41	(2.33-2.49)	**0.00E+00**	**0.00E+00**	-0.28	(-0.39--0.17)	**4.21E-07**	**1.23E-06**
457	PE(P)	PE(P-20:0/22:6)	-0.12	(-0.16--0.07)	**9.70E-07**	**1.17E-06**	-1.87	(-1.92--1.81)	**0.00E+00**	**0.00E+00**	1.97	(1.92-2.03)	**0.00E+00**	**0.00E+00**	-0.01	(-0.11-0.09)	8.53E-01	8.82E-01
458	PE(P)	PE(P-20:1/20:4)	0.35	(0.27-0.44)	**1.53E-16**	**2.07E-16**	-2.86	(-2.96--2.75)	**0.00E+00**	**0.00E+00**	3.04	(2.94-3.14)	**0.00E+00**	**0.00E+00**	0.22	(0.04-0.4)	**1.52E-02**	**2.27E-02**
459	PG	PG(34:2)	-1.30	(-1.38--1.23)	**5.76E-175**	**2.66E-174**	-2.53	(-2.64--2.43)	**9.18E-287**	**1.66E-286**	0.97	(0.85-1.08)	**2.13E-54**	**2.95E-54**	0.12	(-0.07-0.3)	2.16E-01	2.62E-01
460	PG	PG(36:1)	-0.93	(-1--0.86)	**8.11E-121**	**2.36E-120**	-2.60	(-2.7--2.5)	**6.20E-305**	**1.16E-304**	1.25	(1.13-1.36)	**3.36E-84**	**5.25E-84**	0.01	(-0.17-0.19)	9.07E-01	9.25E-01
461	PI	PI(16:0/16:0)	-3.08	(-3.17--2.99)	**0.00E+00**	**0.00E+00**	-4.55	(-4.65--4.46)	**0.00E+00**	**0.00E+00**	1.23	(1.14-1.33)	**1.01E-118**	**1.83E-118**	0.11	(-0.08-0.3)	2.67E-01	3.19E-01

462	PI	PI(16:0_16:1)	-1.94	(-2.04--1.84)	**4.93E-220**	**3.24E-219**	-2.64	(-2.74--2.54)	**0.00E+00**	**0.00E+00**	0.54	(0.45-0.62)	**4.54E-33**	**5.74E-33**	-0.36	(-0.56--0.17)	**3.11E-04**	**5.95E-04**
463	PI	PI(34:0)	-3.12	(-3.21--3.03)	**0.00E+00**	**0.00E+00**	-5.31	(-5.44--5.19)	**0.00E+00**	**0.00E+00**	1.64	(1.52-1.77)	**4.45E-112**	**7.80E-112**	0.37	(0.17-0.58)	**4.66E-04**	**8.77E-04**
464	PI	PI(34:1)	-1.63	(-1.7--1.57)	**0.00E+00**	**0.00E+00**	-4.31	(-4.38--4.25)	**0.00E+00**	**0.00E+00**	2.57	(2.51-2.63)	**0.00E+00**	**0.00E+00**	0.09	(-0.05-0.22)	2.20E-01	2.65E-01
465	PI	PI(17:0_18:1)	-1.46	(-1.52--1.4)	**9.61E-290**	**1.18E-288**	-4.28	(-4.37--4.19)	**0.00E+00**	**0.00E+00**	2.65	(2.55-2.75)	**6.49E-305**	**2.94E-304**	0.03	(-0.12-0.17)	7.24E-01	7.63E-01
466	PI	PI(17:0_18:2)	-1.44	(-1.51--1.38)	**2.02E-277**	**2.21E-276**	-4.39	(-4.48--4.29)	**0.00E+00**	**0.00E+00**	2.64	(2.53-2.74)	**8.74E-283**	**3.53E-282**	0.11	(-0.03-0.25)	1.16E-01	1.48E-01
467	PI	PI(18:0_18:1)	-1.69	(-1.75--1.62)	**0.00E+00**	**6.40E-308**	-6.03	(-6.1--5.95)	**0.00E+00**	**0.00E+00**	4.18	(4.11-4.26)	**0.00E+00**	**0.00E+00**	0.28	(0.14-0.43)	**1.73E-04**	**3.46E-04**
468	PI	PI(36:2)	-1.41	(-1.46--1.36)	**0.00E+00**	**0.00E+00**	-5.19	(-5.25--5.13)	**0.00E+00**	**0.00E+00**	3.61	(3.55-3.67)	**0.00E+00**	**0.00E+00**	0.20	(0.09-0.31)	**6.07E-04**	**1.12E-03**
469	PI	PI(18:1_18:2)	-1.17	(-1.23--1.12)	**7.59E-219**	**4.93E-218**	-2.99	(-3.06--2.93)	**0.00E+00**	**0.00E+00**	1.74	(1.68-1.8)	**0.00E+00**	**0.00E+00**	0.26	(0.13-0.4)	**8.43E-05**	**1.78E-04**
470	PI	PI(16:0_20:3) (a)	-1.44	(-1.5--1.38)	**1.12E-265**	**1.10E-264**	-1.94	(-2--1.88)	**0.00E+00**	**0.00E+00**	0.70	(0.65-0.75)	**1.92E-110**	**3.33E-110**	-0.26	(-0.39--0.13)	**1.27E-04**	**2.60E-04**
471	PI	PI(16:0_20:3) (b)	-1.04	(-1.11--0.97)	**3.19E-156**	**1.21E-155**	-0.72	(-0.8--0.64)	**1.03E-61**	**1.19E-61**	-0.13	(-0.21--0.06)	**6.75E-04**	**7.06E-04**	-0.21	(-0.35--0.07)	**2.83E-03**	**4.76E-03**
472	PI	PI(16:0_20:4)	-1.05	(-1.1--0.99)	**3.13E-208**	**1.85E-207**	-1.11	(-1.17--1.05)	**4.67E-214**	**7.05E-214**	0.05	(-0.01-0.1)	8.69E-02	8.88E-02	-0.09	(-0.21-0.03)	1.43E-01	1.78E-01
473	PI	PI(18:0_20:2)	-1.72	(-1.79--1.66)	**0.00E+00**	**0.00E+00**	-3.34	(-3.43--3.25)	**0.00E+00**	**0.00E+00**	1.58	(1.49-1.67)	**2.93E-174**	**6.73E-174**	0.15	(0.01-0.29)	**3.40E-02**	**4.76E-02**
474	PI	PI(18:0_20:3) (a)	-0.52	(-0.56--0.47)	**1.39E-93**	**3.21E-93**	-1.47	(-1.52--1.42)	**0.00E+00**	**0.00E+00**	0.80	(0.75-0.85)	**1.75E-168**	**3.85E-168**	0.17	(0.07-0.27)	**5.16E-04**	**9.68E-04**
475	PI	PI(18:0_20:4)	-0.48	(-0.52--0.43)	**1.31E-81**	**2.83E-81**	-1.42	(-1.47--1.38)	**0.00E+00**	**0.00E+00**	0.78	(0.73-0.82)	**3.79E-168**	**8.26E-168**	0.18	(0.09-0.28)	**2.11E-04**	**4.15E-04**
476	PI	PI(38:5) (a)	-0.86	(-0.91--0.81)	**9.79E-177**	**4.61E-176**	-0.73	(-0.79--0.67)	**1.66E-105**	**2.02E-105**	-0.22	(-0.27--0.16)	**3.82E-13**	**4.24E-13**	0.11	(0-0.22)	**4.52E-02**	6.17E-02
477	PI	PI(38:6)	-1.34	(-1.4--1.28)	**4.72E-257**	**4.12E-256**	-1.79	(-1.85--1.73)	**0.00E+00**	**0.00E+00**	0.50	(0.44-0.55)	**1.29E-57**	**1.82E-57**	0.04	(-0.09-0.17)	5.35E-01	5.98E-01
478	PI	PI(18:0_22:4)	-1.26	(-1.32--1.2)	**1.54E-250**	**1.27E-249**	-1.82	(-1.88--1.75)	**0.00E+00**	**0.00E+00**	0.57	(0.51-0.63)	**2.36E-68**	**3.49E-68**	-0.02	(-0.14-0.11)	7.94E-01	8.31E-01
479	PI	PI(18:0_22:5) (n3)	-0.46	(-0.52--0.39)	**1.11E-45**	**1.91E-45**	-2.65	(-2.73--2.58)	**0.00E+00**	**0.00E+00**	1.88	(1.81-1.95)	**0.00E+00**	**0.00E+00**	0.20	(0.07-0.33)	**1.95E-03**	**3.37E-03**
480	PI	PI(18:0_22:6)	-1.29	(-1.35--1.23)	**1.85E-243**	**1.44E-242**	-2.53	(-2.6--2.47)	**0.00E+00**	**0.00E+00**	1.18	(1.12-1.24)	**1.69E-204**	**4.55E-204**	0.10	(-0.03-0.23)	1.33E-01	1.67E-01

Table S1C. Comparison of lipid species across four sample groups (antenatal, postnatal, cord blood and 6-year-old child) using paired t-test.

Studies		From antenatal to postnatal (381 pairs)				From antenatal to cord blood (445 pairs)				From birth to child (237 pairs)				From child to adult (postnatal) (272 pairs)			
No.	Lipid Species	$\log_2$FC (Postnatal/Antenatal)	95%CI	p-value	p-value(BH)	$\log_2$FC (CordBlood/Antenatal)	95%CI	p-value	p-value(BH)	$\log_2$FC (Child/CordBlood)	95%CI	p-value	p-value(BH)	$\log_2$FC (Adult/Child)	95%CI	p-value	p-value(BH)
1	AC(12:0)	1.56	(1.46,1.66)	**8.65E-108**	**4.66E-107**	0.00	(-0.08,0.08)	9.62E-01	9.64E-01	1.81	(1.69,1.92)	**1.61E-85**	**4.72E-85**	-0.13	(-0.24,-0.02)	**2.13E-02**	**2.55E-02**
2	AC(13:0)	1.12	(1,1.24)	**2.67E-57**	**5.22E-57**	0.57	(0.48,0.66)	**7.33E-32**	**8.10E-32**	-0.24	(-0.42,-0.07)	**5.58E-03**	**5.84E-03**	0.85	(0.68,1.02)	**4.38E-20**	**2.15E-19**
3	AC(14:0)	1.06	(0.99,1.13)	**6.39E-105**	**3.07E-104**	0.86	(0.81,0.92)	**9.23E-111**	**1.19E-110**	0.29	(0.2,0.38)	**2.05E-09**	**2.37E-09**	-0.03	(-0.11,0.06)	5.25E-01	5.54E-01
4	AC(14:1)	1.81	(1.7,1.92)	**8.37E-110**	**4.62E-109**	0.29	(0.21,0.38)	**2.22E-11**	**2.36E-11**	1.82	(1.7,1.95)	**1.11E-79**	**2.89E-79**	-0.15	(-0.27,-0.04)	**1.12E-02**	**1.37E-02**
5	AC(14:2)	1.65	(1.54,1.75)	**1.19E-106**	**6.00E-106**	0.50	(0.42,0.58)	**1.34E-30**	**1.48E-30**	1.45	(1.35,1.56)	**2.58E-73**	**6.06E-73**	-0.20	(-0.31,-0.08)	**5.88E-04**	**8.09E-04**
6	AC(16:0)	0.90	(0.86,0.95)	**1.93E-129**	**1.54E-128**	0.67	(0.63,0.71)	**1.55E-109**	**1.99E-109**	0.07	(0,0.13)	**4.36E-02**	**4.53E-02**	0.19	(0.14,0.25)	**3.38E-11**	**8.69E-11**
7	AC(16:1)	1.31	(1.23,1.38)	**3.65E-113**	**2.22E-112**	1.03	(0.97,1.1)	**4.53E-113**	**5.88E-113**	0.17	(0.08,0.27)	**6.52E-04**	**6.89E-04**	0.20	(0.11,0.29)	**1.38E-05**	**2.14E-05**
8	AC(18:0)	1.00	(0.95,1.06)	**1.14E-134**	**1.05E-133**	0.32	(0.27,0.36)	**4.65E-37**	**5.19E-37**	0.61	(0.54,0.67)	**3.01E-44**	**4.88E-44**	0.07	(0.01,0.14)	**2.00E-02**	**2.40E-02**
9	AC(18:1)	1.15	(1.09,1.22)	**4.29E-118**	**2.90E-117**	-0.06	(-0.12,0)	6.95E-02	7.11E-02	1.06	(0.98,1.14)	**9.58E-72**	**2.19E-71**	0.24	(0.17,0.3)	**4.75E-12**	**1.33E-11**
10	AC(18:2)	1.08	(1.02,1.14)	**1.61E-118**	**1.10E-117**	0.76	(0.7,0.81)	**2.48E-91**	**3.03E-91**	0.15	(0.08,0.22)	**6.27E-05**	**6.75E-05**	0.23	(0.17,0.3)	**3.63E-12**	**1.03E-11**
11	CE(14:0)	-0.49	(-0.55,-0.43)	**7.19E-45**	**1.25E-44**	-0.49	(-0.54,-0.44)	**2.65E-57**	**3.06E-57**	0.01	(-0.08,0.09)	8.78E-01	8.78E-01	-0.02	(-0.1,0.06)	6.22E-01	6.42E-01
12	CE(16:1)	-0.82	(-0.9,-0.75)	**2.64E-71**	**6.44E-71**	0.16	(0.09,0.23)	**1.41E-05**	**1.47E-05**	-1.21	(-1.3,-1.12)	**5.04E-75**	**1.23E-74**	0.26	(0.18,0.34)	**5.49E-10**	**1.26E-09**
13	CE(18:0)	0.17	(0.13,0.21)	**1.80E-13**	**2.35E-13**	0.54	(0.48,0.6)	**2.50E-59**	**2.90E-59**	-0.49	(-0.57,-0.41)	**9.54E-28**	**1.32E-27**	0.15	(0.08,0.22)	**6.24E-05**	**9.29E-05**
14	CE(18:1)	-0.31	(-0.33,-0.28)	**2.04E-76**	**5.43E-76**	-0.91	(-0.94,-0.87)	**1.53E-183**	**2.60E-183**	0.53	(0.49,0.58)	**1.05E-59**	**1.98E-59**	0.08	(0.04,0.11)	**1.36E-05**	**2.12E-05**
15	CE(18:2)	-0.15	(-0.18,-0.12)	**2.75E-21**	**3.87E-21**	-2.75	(-2.78,-2.71)	**0.00E+00**	**0.00E+00**	2.41	(2.36,2.46)	**4.55E-189**	**4.13E-187**	0.20	(0.15,0.24)	**1.18E-14**	**4.06E-14**
16	CE(18:3)	0.05	(-0.02,0.11)	1.41E-01	1.50E-01	-2.21	(-2.28,-2.14)	**2.80E-224**	**6.81E-224**	2.22	(2.13,2.31)	**7.62E-123**	**4.69E-122**	0.08	(-0.01,0.16)	9.53E-02	1.08E-01
17	CE(20:1)	0.00	(-0.05,0.04)	8.32E-01	8.39E-01	0.06	(-0.01,0.12)	7.74E-02	7.91E-02	-0.15	(-0.24,-0.07)	**4.92E-04**	**5.23E-04**	0.14	(0.08,0.21)	**2.43E-05**	**3.70E-05**
18	CE(20:2)	-0.50	(-0.54,-0.47)	**3.38E-95**	**1.31E-94**	-0.54	(-0.59,-0.49)	**6.59E-72**	**7.77E-72**	-0.14	(-0.21,-0.08)	**2.66E-05**	**2.87E-05**	0.18	(0.13,0.23)	**3.39E-12**	**9.75E-12**
19	CE(20:4)	0.50	(0.45,0.55)	**1.96E-64**	**4.33E-64**	0.33	(0.28,0.38)	**2.48E-30**	**2.72E-30**	-0.03	(-0.11,0.04)	3.45E-01	3.50E-01	0.14	(0.07,0.21)	**8.96E-05**	**1.32E-04**
20	CE(20:5)	1.06	(0.95,1.17)	**1.15E-56**	**2.25E-56**	-1.49	(-1.59,-1.4)	**9.14E-117**	**1.21E-116**	1.91	(1.75,2.07)	**2.41E-64**	**4.82E-64**	0.60	(0.45,0.76)	**1.79E-13**	**5.70E-13**
21	CE(22:5) (n6)	-0.26	(-0.31,-0.21)	**9.85E-25**	**1.44E-24**	-0.49	(-0.55,-0.44)	**1.16E-48**	**1.32E-48**	0.30	(0.22,0.38)	**3.75E-12**	**4.48E-12**	-0.09	(-0.15,-0.03)	**2.03E-03**	**2.64E-03**
22	CE(22:6)	-0.36	(-0.42,-0.3)	**2.45E-26**	**3.59E-26**	-1.87	(-1.94,-1.81)	**1.67E-204**	**3.25E-204**	0.89	(0.78,1.01)	**2.08E-39**	**3.23E-39**	0.52	(0.43,0.61)	**8.74E-25**	**6.66E-24**
23	CE(24:5)	-1.07	(-1.13,-1)	**1.69E-111**	**9.87E-111**	1.11	(1.03,1.19)	**2.11E-94**	**2.63E-94**	-2.08	(-2.21,-1.95)	**8.80E-88**	**2.69E-87**	-0.07	(-0.16,0.02)	1.16E-01	1.29E-01
24	CE(24:6)	-0.83	(-0.9,-0.76)	**1.52E-76**	**4.08E-76**	-0.47	(-0.55,-0.39)	**6.60E-26**	**7.21E-26**	-0.21	(-0.33,-0.09)	**5.12E-04**	**5.42E-04**	-0.12	(-0.21,-0.02)	**1.40E-02**	**1.71E-02**
25	DG(16:0_16:1)	-1.57	(-1.67,-1.46)	**1.88E-102**	**8.67E-102**	-2.08	(-2.17,-1.99)	**1.08E-165**	**1.68E-165**	0.24	(0.13,0.35)	**3.04E-05**	**3.28E-05**	0.31	(0.18,0.43)	**1.23E-06**	**2.10E-06**
26	DG(14:0_18:2)	-1.65	(-1.78,-1.53)	**4.04E-89**	**1.33E-88**	-3.76	(-3.88,-3.64)	**1.31E-222**	**3.09E-222**	1.74	(1.56,1.91)	**9.07E-51**	**1.59E-50**	0.40	(0.22,0.58)	**1.52E-05**	**2.34E-05**
27	DG(16:0_18:1)	-1.60	(-1.68,-1.52)	**1.25E-138**	**1.25E-137**	-3.20	(-3.28,-3.12)	**2.00E-269**	**8.75E-269**	1.42	(1.31,1.54)	**2.40E-66**	**4.96E-66**	0.27	(0.15,0.39)	**1.29E-05**	**2.01E-05**
28	DG(16:1_18:1)	-1.13	(-1.22,-1.04)	**1.77E-77**	**4.84E-77**	-2.34	(-2.43,-2.26)	**5.82E-195**	**1.06E-194**	0.84	(0.71,0.97)	**9.89E-29**	**1.39E-28**	0.45	(0.32,0.58)	**6.19E-11**	**1.56E-10**
29	DG(16:0_18:2)	-1.48	(-1.55,-1.4)	**3.77E-136**	**3.62E-135**	-3.65	(-3.74,-3.56)	**4.47E-266**	**1.83E-265**	1.75	(1.63,1.88)	**5.73E-76**	**1.44E-75**	0.47	(0.35,0.58)	**3.00E-14**	**1.00E-13**
30	DG(18:0_18:1)	-0.99	(-1.07,-0.92)	**2.51E-90**	**8.80E-90**	-2.55	(-2.62,-2.47)	**6.14E-238**	**1.78E-237**	1.38	(1.27,1.49)	**1.26E-66**	**2.62E-66**	0.25	(0.13,0.36)	**3.62E-05**	**5.47E-05**
31	DG(18:1_18:1)	-0.92	(-0.99,-0.84)	**2.19E-81**	**6.40E-81**	-3.20	(-3.28,-3.13)	**7.35E-274**	**3.43E-273**	2.10	(1.99,2.21)	**8.18E-103**	**3.22E-102**	0.27	(0.16,0.38)	**1.65E-06**	**2.81E-06**
32	DG(18:0_18:2)	-1.09	(-1.17,-1.01)	**1.66E-93**	**6.21E-93**	-2.88	(-2.97,-2.78)	**8.60E-213**	**1.81E-212**	1.51	(1.38,1.64)	**9.88E-60**	**1.87E-59**	0.37	(0.25,0.5)	**1.55E-08**	**3.12E-08**
33	DG(18:1_18:2)	-0.83	(-0.9,-0.76)	**5.07E-75**	**1.31E-74**	-3.71	(-3.8,-3.63)	**2.12E-276**	**1.03E-275**	2.51	(2.39,2.63)	**4.26E-112**	**2.07E-111**	0.48	(0.37,0.58)	**5.76E-17**	**2.47E-16**
34	DG(18:2_18:2)	-0.72	(-0.8,-0.63)	**2.70E-49**	**4.91E-49**	-4.05	(-4.16,-3.94)	**1.32E-248**	**4.42E-248**	2.75	(2.61,2.89)	**1.76E-103**	**7.05E-103**	0.70	(0.57,0.82)	**5.67E-23**	**3.63E-22**
35	DG(18:1_18:3)	-0.50	(-0.58,-0.42)	**1.18E-30**	**1.83E-30**	-3.17	(-3.26,-3.08)	**3.43E-238**	**9.97E-238**	2.32	(2.18,2.46)	**1.75E-90**	**5.63E-90**	0.43	(0.31,0.56)	**2.54E-11**	**6.60E-11**
36	DG(16:0_20:4)	-1.57	(-1.66,-1.47)	**1.95E-116**	**1.28E-115**	-1.40	(-1.49,-1.32)	**1.44E-117**	**1.92E-117**	-0.54	(-0.66,-0.41)	**1.29E-15**	**1.59E-15**	0.33	(0.21,0.46)	**2.60E-07**	**4.74E-07**
37	DG(18:1_20:3)	-0.93	(-1,-0.85)	**8.58E-84**	**2.66E-83**	-1.05	(-1.12,-0.97)	**4.12E-97**	**5.16E-97**	0.22	(0.11,0.33)	**1.06E-04**	**1.14E-04**	-0.06	(-0.17,0.04)	2.40E-01	2.59E-01
38	DG(18:1_20:4)	-0.75	(-0.82,-0.69)	**1.22E-75**	**3.22E-75**	-1.57	(-1.64,-1.49)	**2.89E-159**	**4.39E-159**	0.45	(0.34,0.55)	**1.65E-15**	**2.03E-15**	0.34	(0.24,0.43)	**1.69E-11**	**4.41E-11**
39	DG(16:0_22:5)	-1.35	(-1.43,-1.26)	**1.73E-103**	**8.15E-103**	-0.97	(-1.06,-0.88)	**2.23E-71**	**2.62E-71**	-0.69	(-0.81,-0.57)	**5.43E-23**	**7.18E-23**	0.28	(0.16,0.41)	**1.07E-05**	**1.69E-05**
40	DG(18:2_20:4)	-0.75	(-0.82,-0.68)	**1.10E-64**	**2.44E-64**	-1.89	(-1.98,-1.8)	**2.38E-153**	**3.57E-153**	0.63	(0.49,0.76)	**2.99E-17**	**3.75E-17**	0.53	(0.42,0.64)	**1.81E-18**	**8.43E-18**
41	DG(16:0_22:6)	-1.68	(-1.79,-1.58)	**1.73E-107**	**9.12E-107**	-1.31	(-1.41,-1.22)	**1.09E-94**	**1.36E-94**	-1.00	(-1.15,-0.84)	**4.87E-28**	**6.78E-28**	0.69	(0.53,0.85)	**1.10E-15**	**4.27E-15**
42	DG(18:1_22:5)	-0.92	(-1,-0.84)	**2.68E-71**	**6.50E-71**	-0.93	(-1.01,-0.85)	**1.04E-72**	**1.23E-72**	-0.16	(-0.3,-0.03)	**1.42E-02**	**1.48E-02**	0.18	(0.08,0.29)	**1.02E-03**	**1.38E-03**
43	DG(18:1_22:6)	-0.92	(-1.01,-0.82)	**4.96E-58**	**9.84E-58**	-1.24	(-1.33,-1.15)	**1.66E-99**	**2.09E-99**	-0.33	(-0.46,-0.2)	**7.23E-07**	**8.02E-07**	0.71	(0.57,0.85)	**1.48E-20**	**7.62E-20**
44	DG(18:2_22:6)	-0.92	(-1.02,-0.82)	**8.85E-52**	**1.66E-51**	-1.59	(-1.69,-1.48)	**5.74E-105**	**7.30E-105**	-0.25	(-0.4,-0.11)	**7.49E-04**	**7.90E-04**	0.91	(0.76,1.06)	**8.68E-26**	**7.57E-25**
45	TG(48:0) [NL-18:0]	-1.12	(-1.24,-0.99)	**1.32E-51**	**2.47E-51**	-1.73	(-1.83,-1.64)	**2.53E-135**	**3.51E-135**	0.54	(0.38,0.7)	**1.60E-10**	**1.87E-10**	0.12	(-0.06,0.3)	1.77E-01	1.94E-01
46	TG(48:1) [NL-16:1]	-2.19	(-2.32,-2.06)	**3.03E-114**	**1.89E-113**	-2.58	(-2.69,-2.46)	**1.31E-163**	**2.02E-163**	-0.05	(-0.22,0.13)	6.01E-01	6.03E-01	0.49	(0.32,0.66)	**5.12E-08**	**9.87E-08**

47	TG(48:1) [NL-18:1]	-1.86	(-1.99,-1.74)	**1.23E-98**	**5.01E-98**	-3.57	(-3.68,-3.47)	**5.89E-231**	**1.56E-230**	1.41	(1.22,1.59)	**4.97E-36**	**7.51E-36**	0.36	(0.17,0.56)	**3.06E-04**	**4.32E-04**
48	TG(48:2) [NL-14:0]	-2.08	(-2.21,-1.94)	**5.89E-105**	**2.86E-104**	-3.78	(-3.89,-3.66)	**3.72E-226**	**9.41E-226**	1.15	(0.97,1.34)	**1.53E-26**	**2.09E-26**	0.64	(0.44,0.85)	**1.42E-09**	**3.12E-09**
49	TG(48:2) [NL-14:1]	-1.75	(-1.88,-1.62)	**1.26E-90**	**4.48E-90**	-3.62	(-3.74,-3.51)	**6.78E-224**	**1.64E-223**	1.62	(1.44,1.79)	**3.79E-47**	**6.42E-47**	0.34	(0.16,0.52)	**3.04E-04**	**4.31E-04**
50	TG(48:2) [NL-16:1]	-1.92	(-2.06,-1.78)	**4.29E-90**	**1.47E-89**	-2.46	(-2.58,-2.33)	**2.48E-143**	**3.54E-143**	-0.11	(-0.29,0.07)	2.16E-01	2.20E-01	0.71	(0.53,0.9)	**1.96E-13**	**6.18E-13**
51	TG(48:2) [NL-18:2]	-2.09	(-2.22,-1.96)	**3.85E-105**	**1.89E-104**	-4.23	(-4.35,-4.11)	**2.98E-238**	**8.73E-238**	1.58	(1.39,1.77)	**2.67E-40**	**4.20E-40**	0.66	(0.45,0.86)	**1.07E-09**	**2.38E-09**
52	TG(48:3) [NL-14:0]	-1.67	(-1.8,-1.54)	**3.16E-81**	**9.19E-81**	-3.25	(-3.37,-3.13)	**1.11E-190**	**1.94E-190**	0.96	(0.77,1.15)	**2.28E-19**	**2.95E-19**	0.74	(0.55,0.94)	**1.24E-12**	**3.69E-12**
53	TG(48:3) [NL-16:1]	-1.51	(-1.63,-1.39)	**1.39E-80**	**4.00E-80**	-2.19	(-2.31,-2.08)	**1.33E-141**	**1.87E-141**	0.10	(-0.06,0.25)	2.12E-01	2.16E-01	0.69	(0.53,0.85)	**3.13E-15**	**1.15E-14**
54	TG(48:3) [NL-18:3]	-1.70	(-1.85,-1.55)	**2.15E-71**	**5.28E-71**	-3.30	(-3.42,-3.18)	**1.31E-197**	**2.43E-197**	1.20	(1.02,1.38)	**2.93E-30**	**4.19E-30**	0.47	(0.26,0.68)	**1.86E-05**	**2.85E-05**
55	TG(49:1) [NL-17:1]	-1.66	(-1.75,-1.57)	**3.70E-127**	**2.87E-126**	-2.31	(-2.4,-2.23)	**1.56E-198**	**2.90E-198**	0.36	(0.25,0.47)	**5.94E-10**	**6.93E-10**	0.33	(0.21,0.44)	**1.11E-07**	**2.09E-07**
56	TG(50:1) [NL-14:0]	-1.10	(-1.21,-0.98)	**3.54E-58**	**7.13E-58**	-1.89	(-1.98,-1.8)	**2.54E-149**	**3.72E-149**	0.72	(0.57,0.87)	**1.82E-17**	**2.29E-17**	0.16	(-0.01,0.32)	6.14E-02	7.02E-02
57	TG(50:1) [NL-16:0]	-1.59	(-1.67,-1.51)	**4.00E-132**	**3.31E-131**	-2.80	(-2.88,-2.71)	**4.08E-233**	**1.10E-232**	0.99	(0.86,1.12)	**2.04E-35**	**3.05E-35**	0.27	(0.15,0.38)	**1.04E-05**	**1.65E-05**
58	TG(50:1) [NL-18:1]	-1.55	(-1.63,-1.47)	**1.07E-130**	**8.68E-130**	-2.80	(-2.88,-2.72)	**2.94E-237**	**8.45E-237**	1.00	(0.87,1.13)	**3.94E-37**	**5.98E-37**	0.30	(0.18,0.41)	**5.63E-07**	**9.93E-07**
59	TG(50:2) [NL-14:0]	-0.94	(-1.04,-0.85)	**9.76E-64**	**2.13E-63**	-2.91	(-3.01,-2.82)	**4.97E-219**	**1.14E-218**	1.78	(1.62,1.93)	**1.74E-61**	**3.38E-61**	0.30	(0.15,0.44)	**7.21E-05**	**1.07E-04**
60	TG(50:2) [NL-16:1]	-1.16	(-1.25,-1.07)	**2.31E-82**	**6.87E-82**	-2.22	(-2.32,-2.12)	**6.11E-163**	**9.33E-163**	0.65	(0.5,0.81)	**1.49E-14**	**1.81E-14**	0.49	(0.36,0.62)	**1.54E-12**	**4.57E-12**
61	TG(50:2) [NL-18:1]	-1.03	(-1.12,-0.95)	**1.63E-77**	**4.47E-77**	-2.41	(-2.51,-2.31)	**1.84E-179**	**3.04E-179**	1.01	(0.85,1.17)	**2.19E-28**	**3.05E-28**	0.48	(0.35,0.61)	**3.97E-12**	**1.12E-11**
62	TG(50:2) [NL-18:2]	-1.49	(-1.56,-1.41)	**2.63E-132**	**2.21E-131**	-3.35	(-3.46,-3.25)	**6.61E-222**	**1.55E-221**	1.46	(1.31,1.61)	**4.70E-48**	**8.03E-48**	0.48	(0.37,0.6)	**5.25E-15**	**1.88E-14**
63	TG(50:3) [NL-14:0]	-1.31	(-1.41,-1.21)	**8.08E-85**	**2.52E-84**	-3.90	(-4.01,-3.79)	**5.39E-244**	**1.72E-243**	2.06	(1.88,2.23)	**3.41E-62**	**6.69E-62**	0.66	(0.5,0.83)	**7.45E-14**	**2.43E-13**
64	TG(50:3) [NL-14:1]	-1.00	(-1.09,-0.91)	**3.68E-72**	**9.19E-72**	-2.95	(-3.03,-2.86)	**3.00E-236**	**8.41E-236**	1.80	(1.66,1.94)	**1.48E-67**	**3.14E-67**	0.26	(0.13,0.39)	**1.07E-04**	**1.56E-04**
65	TG(50:3) [NL-16:1]	-1.38	(-1.48,-1.28)	**1.16E-89**	**3.90E-89**	-2.87	(-2.97,-2.76)	**6.06E-193**	**1.08E-192**	0.71	(0.54,0.88)	**8.56E-15**	**1.04E-14**	0.89	(0.74,1.04)	**3.02E-26**	**2.78E-25**
66	TG(50:3) [NL-18:2]	-1.30	(-1.39,-1.21)	**3.03E-91**	**1.10E-90**	-3.40	(-3.51,-3.29)	**4.95E-217**	**1.10E-216**	1.36	(1.19,1.53)	**9.86E-39**	**1.51E-38**	0.85	(0.7,1)	**5.05E-25**	**3.97E-24**
67	TG(50:3) [NL-18:3]	-1.69	(-1.8,-1.58)	**3.97E-103**	**1.85E-102**	-3.48	(-3.58,-3.37)	**3.71E-222**	**8.73E-222**	1.27	(1.11,1.43)	**7.01E-37**	**1.06E-36**	0.60	(0.43,0.76)	**7.46E-12**	**2.03E-11**
68	TG(50:4) [NL-14:0]	-1.29	(-1.4,-1.18)	**1.11E-76**	**3.02E-76**	-3.69	(-3.81,-3.58)	**1.03E-219**	**2.37E-219**	1.72	(1.54,1.9)	**2.94E-50**	**5.15E-50**	0.78	(0.61,0.95)	**3.21E-17**	**1.41E-16**
69	TG(50:4) [NL-18:3]	-1.15	(-1.26,-1.03)	**1.78E-59**	**3.65E-59**	-2.75	(-2.86,-2.63)	**5.08E-177**	**8.29E-177**	1.01	(0.84,1.17)	**9.55E-27**	**1.31E-26**	0.69	(0.52,0.85)	**3.91E-15**	**1.42E-14**
70	TG(50:4) [NL-20:4]	-2.14	(-2.29,-1.98)	**1.99E-92**	**7.35E-92**	-1.96	(-2.09,-1.82)	**1.09E-102**	**1.37E-102**	-0.71	(-0.9,-0.52)	**2.84E-12**	**3.41E-12**	0.49	(0.28,0.7)	**5.47E-06**	**8.91E-06**
71	TG(51:1) [NL-17:0]	-1.44	(-1.53,-1.35)	**1.12E-108**	**6.12E-108**	-2.75	(-2.84,-2.66)	**4.57E-216**	**1.01E-215**	1.16	(1.02,1.3)	**2.79E-40**	**4.37E-40**	0.22	(0.09,0.36)	**1.37E-03**	**1.83E-03**
72	TG(51:2) [NL-15:0]	-0.97	(-1.05,-0.9)	**1.69E-83**	**5.17E-83**	-3.41	(-3.5,-3.32)	**7.89E-253**	**2.77E-252**	2.16	(2.02,2.31)	**1.99E-80**	**5.24E-80**	0.36	(0.23,0.49)	**6.36E-08**	**1.22E-07**
73	TG(51:2) [NL-17:0]	-1.35	(-1.43,-1.26)	**1.56E-106**	**7.71E-106**	-3.12	(-3.22,-3.03)	**7.23E-230**	**1.90E-229**	1.37	(1.22,1.52)	**8.35E-48**	**1.42E-47**	0.49	(0.35,0.62)	**1.30E-11**	**3.43E-11**
74	TG(51:2) [NL-17:1]	-1.27	(-1.35,-1.19)	**1.69E-107**	**9.04E-107**	-2.82	(-2.9,-2.74)	**8.48E-239**	**2.50E-238**	1.16	(1.03,1.29)	**6.62E-47**	**1.12E-46**	0.45	(0.33,0.58)	**6.09E-12**	**1.68E-11**
75	TG(52:1) [NL-18:0]	-1.39	(-1.49,-1.3)	**6.66E-102**	**2.99E-101**	-2.67	(-2.76,-2.57)	**8.29E-201**	**1.57E-200**	1.11	(0.95,1.26)	**4.36E-33**	**6.46E-33**	0.26	(0.12,0.4)	**4.21E-04**	**5.87E-04**
76	TG(52:1) [NL-18:1]	-1.29	(-1.38,-1.2)	**1.02E-93**	**3.85E-93**	-2.54	(-2.64,-2.45)	**1.28E-195**	**2.35E-195**	1.04	(0.89,1.19)	**3.67E-31**	**5.32E-31**	0.31	(0.17,0.45)	**2.20E-05**	**3.37E-05**
77	TG(52:2) [NL-16:0]	-0.64	(-0.69,-0.59)	**8.03E-83**	**2.42E-82**	-2.53	(-2.6,-2.46)	**7.54E-240**	**2.26E-239**	1.73	(1.63,1.84)	**1.68E-88**	**5.25E-88**	0.24	(0.17,0.32)	**1.86E-09**	**4.03E-09**
78	TG(52:2) [NL-18:2]	-0.96	(-1.03,-0.89)	**1.72E-85**	**5.39E-85**	-2.26	(-2.35,-2.17)	**3.21E-176**	**5.21E-176**	0.99	(0.85,1.13)	**1.65E-31**	**2.40E-31**	0.40	(0.29,0.51)	**3.90E-11**	**9.97E-11**
79	TG(52:3) [NL-16:1]	-0.43	(-0.5,-0.36)	**3.45E-29**	**5.28E-29**	-1.89	(-1.98,-1.8)	**3.00E-152**	**4.49E-152**	1.13	(0.99,1.27)	**6.21E-39**	**9.56E-39**	0.43	(0.33,0.53)	**6.46E-15**	**2.30E-14**
80	TG(52:3) [NL-18:2]	-0.65	(-0.7,-0.6)	**2.54E-80**	**7.26E-80**	-3.37	(-3.47,-3.27)	**1.76E-233**	**4.80E-233**	2.35	(2.21,2.5)	**2.99E-89**	**9.51E-89**	0.48	(0.39,0.56)	**4.55E-24**	**3.31E-23**
81	TG(52:4) [NL-16:1]	-0.79	(-0.87,-0.7)	**1.01E-54**	**1.95E-54**	-2.99	(-3.1,-2.88)	**4.12E-201**	**7.81E-201**	1.51	(1.34,1.67)	**1.37E-45**	**2.27E-45**	0.84	(0.7,0.97)	**2.13E-28**	**2.33E-27**
82	TG(52:4) [NL-18:2]	-0.96	(-1.04,-0.88)	**1.30E-76**	**3.52E-76**	-4.16	(-4.28,-4.04)	**5.22E-239**	**1.55E-238**	2.42	(2.25,2.6)	**2.85E-74**	**6.83E-74**	0.89	(0.76,1.02)	**2.49E-31**	**4.43E-30**
83	TG(52:4) [NL-18:3]	-0.90	(-0.98,-0.82)	**3.92E-69**	**9.18E-69**	-3.71	(-3.81,-3.61)	**3.24E-249**	**1.10E-248**	2.30	(2.14,2.45)	**3.13E-80**	**8.21E-80**	0.61	(0.48,0.74)	**3.52E-17**	**1.54E-16**
84	TG(52:5) [NL-18:3]	-0.93	(-1.03,-0.84)	**4.83E-58**	**9.62E-58**	-3.61	(-3.72,-3.5)	**1.71E-226**	**4.35E-226**	2.02	(1.86,2.18)	**6.33E-68**	**1.34E-67**	0.79	(0.64,0.93)	**1.71E-22**	**1.03E-21**
85	TG(52:5) [NL-20:4]	-1.62	(-1.74,-1.5)	**6.23E-88**	**2.02E-87**	-1.14	(-1.27,-1.02)	**2.91E-56**	**3.35E-56**	-1.23	(-1.41,-1.06)	**1.16E-32**	**1.72E-32**	0.74	(0.58,0.9)	**5.81E-17**	**2.47E-16**
86	TG(52:5) [NL-20:5]	-1.24	(-1.35,-1.12)	**1.66E-65**	**3.72E-65**	-2.06	(-2.17,-1.95)	**7.10E-138**	**9.90E-138**	-0.12	(-0.29,0.04)	1.30E-01	1.34E-01	0.94	(0.77,1.1)	**4.24E-23**	**2.79E-22**
87	TG(53:2) [NL-18:1]	-0.76	(-0.83,-0.69)	**4.13E-67**	**9.48E-67**	-2.65	(-2.73,-2.57)	**3.81E-225**	**9.47E-225**	1.78	(1.66,1.9)	**7.56E-81**	**2.03E-80**	0.20	(0.09,0.31)	**4.85E-04**	**6.73E-04**
88	TG(54:1) [NL-18:1]	-0.86	(-0.93,-0.78)	**1.04E-71**	**2.59E-71**	-2.43	(-2.52,-2.34)	**1.52E-203**	**2.91E-203**	1.42	(1.28,1.57)	**8.25E-52**	**1.47E-51**	0.20	(0.09,0.32)	**4.58E-04**	**6.38E-04**
89	TG(54:2) [NL-18:0]	-0.72	(-0.79,-0.65)	**1.90E-59**	**3.89E-59**	-2.41	(-2.5,-2.33)	**1.71E-202**	**3.27E-202**	1.47	(1.33,1.6)	**2.40E-56**	**4.46E-56**	0.32	(0.21,0.44)	**2.10E-08**	**4.18E-08**
90	TG(54:2) [NL-20:1]	-1.83	(-1.91,-1.76)	**1.72E-156**	**2.58E-155**	-3.98	(-4.06,-3.89)	**1.09E-291**	**7.60E-291**	2.10	(1.97,2.22)	**8.66E-91**	**2.83E-90**	0.12	(0.01,0.24)	**3.27E-02**	**3.87E-02**
91	TG(54:3) [NL-18:1]	-0.22	(-0.28,-0.16)	**1.17E-12**	**1.50E-12**	-2.02	(-2.1,-1.95)	**1.91E-194**	**3.48E-194**	1.77	(1.67,1.88)	**1.71E-88**	**5.30E-88**	0.12	(0.04,0.2)	**5.27E-03**	**6.62E-03**
92	TG(54:3) [NL-18:2]	-0.39	(-0.45,-0.34)	**7.37E-36**	**1.19E-35**	-2.45	(-2.53,-2.36)	**9.98E-208**	**2.00E-207**	1.69	(1.57,1.81)	**1.22E-75**	**3.03E-75**	0.44	(0.35,0.52)	**6.76E-20**	**3.28E-19**
93	TG(54:4) [NL-18:2]	-0.26	(-0.31,-0.2)	**1.93E-16**	**2.61E-16**	-3.06	(-3.16,-2.96)	**4.07E-215**	**8.88E-215**	2.57	(2.43,2.72)	**7.04E-95**	**2.45E-94**	0.35	(0.26,0.45)	**5.54E-12**	**1.54E-11**
94	TG(54:4) [NL-20:3]	-1.22	(-1.29,-1.14)	**7.36E-112**	**4.36E-111**	-1.51	(-1.6,-1.42)	**7.19E-117**	**9.56E-117**	0.12	(-0.02,0.26)	8.57E-02	8.82E-02	0.18	(0.07,0.29)	**1.42E-03**	**1.88E-03**
95	TG(54:5) [NL-18:3]	-0.51	(-0.6,-0.42)	**1.80E-26**	**2.65E-26**	-3.44	(-3.54,-3.34)	**4.31E-237**	**1.22E-236**	2.53	(2.38,2.68)	**2.84E-91**	**9.42E-91**	0.53	(0.4,0.66)	**1.21E-13**	**3.87E-13**
96	TG(54:5) [NL-20:4]	-0.94	(-1.01,-0.87)	**8.89E-90**	**3.00E-89**	-1.54	(-1.64,-1.44)	**2.69E-109**	**3.45E-109**	0.07	(-0.09,0.22)	3.99E-01	4.04E-01	0.57	(0.46,0.67)	**3.94E-23**	**2.63E-22**
97	TG(54:6) [NL-18:3]	-0.61	(-0.71,-0.51)	**2.15E-27**	**3.25E-27**	-3.66	(-3.78,-3.55)	**2.03E-223**	**4.90E-223**	2.50	(2.35,2.66)	**1.01E-85**	**2.99E-85**	0.67	(0.52,0.82)	**2.65E-16**	**1.06E-15**

98	TG(54:6) [NL-20:4]	-1.21	(-1.29,-1.13)	**2.67E-102**	**1.22E-101**	-1.93	(-2.04,-1.82)	**3.01E-129**	**4.13E-129**	-0.15	(-0.32,0.01)	6.74E-02	6.97E-02	0.87	(0.75,0.99)	**9.35E-35**	**2.81E-33**
99	TG(54:6) [NL-20:5]	-0.54	(-0.65,-0.44)	**1.28E-21**	**1.81E-21**	-2.32	(-2.43,-2.21)	**4.51E-152**	**6.73E-152**	0.69	(0.51,0.87)	**3.92E-13**	**4.72E-13**	1.08	(0.92,1.25)	**6.44E-30**	**8.59E-29**
100	TG(54:6) [NL-22:6]	-2.12	(-2.23,-2)	**1.13E-125**	**8.31E-125**	-1.54	(-1.66,-1.43)	**1.49E-92**	**1.83E-92**	-1.51	(-1.68,-1.33)	**2.02E-43**	**3.25E-43**	0.93	(0.76,1.11)	**1.93E-21**	**1.10E-20**
101	TG(54:7) [NL-20:5]	-0.51	(-0.62,-0.4)	**3.02E-18**	**4.14E-18**	-2.09	(-2.21,-1.98)	**4.67E-131**	**6.44E-131**	0.41	(0.23,0.59)	**9.28E-06**	**1.01E-05**	1.20	(1.03,1.37)	**1.09E-32**	**2.28E-31**
102	TG(54:7) [NL-22:6]	-1.58	(-1.71,-1.46)	**2.59E-82**	**7.66E-82**	-0.62	(-0.74,-0.51)	**2.05E-23**	**2.23E-23**	-2.05	(-2.21,-1.88)	**6.70E-66**	**1.37E-65**	1.14	(0.96,1.32)	**6.03E-28**	**6.30E-27**
103	TG(56:6) [NL-20:4]	-0.01	(-0.05,0.04)	7.88E-01	7.97E-01	-1.04	(-1.13,-0.96)	**2.21E-79**	**2.64E-79**	0.57	(0.44,0.7)	**3.64E-16**	**4.50E-16**	0.50	(0.43,0.56)	**3.60E-35**	**1.24E-33**
104	TG(56:6) [NL-22:5]	-0.66	(-0.73,-0.6)	**3.30E-64**	**7.27E-64**	-0.91	(-1,-0.81)	**2.59E-58**	**3.01E-58**	-0.23	(-0.37,-0.08)	**1.93E-03**	**2.03E-03**	0.51	(0.41,0.61)	**1.86E-20**	**9.50E-20**
105	TG(56:7) [NL-20:4]	-0.57	(-0.63,-0.51)	**2.53E-54**	**4.85E-54**	-1.64	(-1.73,-1.54)	**6.77E-124**	**9.15E-124**	0.42	(0.27,0.56)	**1.11E-07**	**1.25E-07**	0.63	(0.54,0.73)	**2.51E-30**	**3.54E-29**
106	TG(56:7) [NL-20:5]	0.01	(-0.09,0.11)	8.47E-01	8.52E-01	-2.00	(-2.1,-1.9)	**3.43E-149**	**5.01E-149**	1.07	(0.91,1.23)	**1.61E-29**	**2.30E-29**	0.93	(0.78,1.07)	**1.44E-29**	**1.78E-28**
107	TG(56:7) [NL-22:5]	-1.00	(-1.07,-0.92)	**1.64E-90**	**5.80E-90**	-1.48	(-1.58,-1.38)	**5.39E-101**	**6.79E-101**	-0.35	(-0.51,-0.19)	**1.84E-05**	**1.99E-05**	0.87	(0.74,1)	**6.37E-32**	**1.27E-30**
108	TG(56:7) [NL-22:6]	-1.27	(-1.37,-1.17)	**3.25E-83**	**9.87E-83**	-1.60	(-1.7,-1.49)	**2.05E-104**	**2.60E-104**	-0.70	(-0.86,-0.53)	**2.51E-14**	**3.04E-14**	1.05	(0.89,1.21)	**7.91E-31**	**1.27E-29**
109	TG(56:8) [NL-20:4]	-0.73	(-0.81,-0.66)	**4.09E-59**	**8.32E-59**	-1.06	(-1.16,-0.96)	**2.00E-70**	**2.34E-70**	-0.35	(-0.49,-0.21)	**2.88E-06**	**3.16E-06**	0.65	(0.54,0.76)	**3.32E-26**	**3.01E-25**
110	TG(56:8) [NL-20:5]	-0.14	(-0.24,-0.04)	**7.57E-03**	**8.43E-03**	-1.88	(-1.98,-1.77)	**6.99E-128**	**9.53E-128**	0.77	(0.61,0.93)	**7.65E-18**	**9.69E-18**	0.97	(0.82,1.12)	**3.09E-29**	**3.71E-28**
111	TG(56:8) [NL-22:6]	-1.39	(-1.49,-1.28)	**5.78E-86**	**1.83E-85**	-1.60	(-1.72,-1.48)	**6.56E-94**	**8.14E-94**	-1.02	(-1.2,-0.85)	**3.96E-24**	**5.26E-24**	1.29	(1.13,1.46)	**8.66E-39**	**4.63E-37**
112	TG(56:9) [NL-22:6]	-1.03	(-1.13,-0.93)	**3.88E-61**	**8.17E-61**	-0.92	(-1.03,-0.81)	**9.97E-50**	**1.14E-49**	-1.22	(-1.39,-1.06)	**1.16E-34**	**1.73E-34**	1.14	(0.99,1.3)	**7.51E-35**	**2.40E-33**
113	TG(58:10) [NL-22:6]	-0.87	(-0.97,-0.77)	**1.72E-50**	**3.18E-50**	-0.81	(-0.91,-0.7)	**9.21E-43**	**1.04E-42**	-1.12	(-1.28,-0.96)	**3.00E-31**	**4.37E-31**	1.08	(0.92,1.23)	**5.95E-33**	**1.43E-31**
114	TG(58:8) [NL-22:6]	-0.93	(-1.03,-0.84)	**5.28E-56**	**1.03E-55**	-1.03	(-1.13,-0.93)	**7.33E-67**	**8.56E-67**	-0.86	(-1.02,-0.7)	**5.35E-22**	**7.04E-22**	1.00	(0.84,1.15)	**6.19E-30**	**8.49E-29**
115	TG(58:9) [NL-22:6]	-1.02	(-1.12,-0.92)	**9.93E-62**	**2.12E-61**	-1.17	(-1.28,-1.07)	**2.13E-71**	**2.50E-71**	-0.88	(-1.05,-0.71)	**4.64E-21**	**6.07E-21**	1.09	(0.93,1.24)	**7.67E-33**	**1.75E-31**
116	TG(O-50:1) [NL-16:0]	-0.60	(-0.67,-0.54)	**2.05E-55**	**3.96E-55**	-3.19	(-3.25,-3.12)	**1.89E-302**	**1.82E-301**	2.67	(2.57,2.78)	**1.63E-128**	**1.24E-127**	-0.06	(-0.15,0.03)	1.96E-01	2.14E-01
117	TG(O-50:1) [NL-17:1]	-1.68	(-1.77,-1.59)	**1.04E-126**	**7.92E-126**	-2.35	(-2.43,-2.26)	**2.25E-197**	**4.15E-197**	0.37	(0.26,0.48)	**4.23E-10**	**4.95E-10**	0.33	(0.21,0.45)	**1.21E-07**	**2.27E-07**
118	TG(O-50:1) [NL-18:1]	-0.48	(-0.55,-0.41)	**7.81E-33**	**1.23E-32**	-3.37	(-3.44,-3.3)	**2.40E-296**	**2.02E-295**	2.85	(2.74,2.97)	**6.17E-128**	**4.36E-127**	0.08	(-0.02,0.18)	1.35E-01	1.50E-01
119	TG(O-50:2) [NL-18:2]	-0.57	(-0.63,-0.51)	**4.23E-51**	**7.87E-51**	-2.65	(-2.72,-2.59)	**2.70E-273**	**1.22E-272**	2.10	(2,2.2)	**3.78E-110**	**1.71E-109**	0.10	(0.01,0.19)	**2.48E-02**	**2.94E-02**
120	TG(O-52:2) [NL-16:0]	-0.78	(-0.86,-0.7)	**7.10E-60**	**1.48E-59**	-3.16	(-3.26,-3.06)	**2.77E-225**	**6.92E-225**	2.49	(2.34,2.64)	**2.36E-89**	**7.57E-89**	-0.01	(-0.13,0.11)	8.65E-01	8.80E-01
121	TG(O-52:2) [NL-17:1]	-1.28	(-1.36,-1.2)	**6.19E-107**	**3.19E-106**	-2.84	(-2.92,-2.76)	**4.06E-237**	**1.15E-236**	1.18	(1.05,1.3)	**4.39E-47**	**7.42E-47**	0.45	(0.33,0.58)	**8.57E-12**	**2.28E-11**
122	TG(O-52:2) [NL-18:1]	-0.66	(-0.74,-0.58)	**6.29E-45**	**1.10E-44**	-3.10	(-3.2,-3)	**1.17E-217**	**2.63E-217**	2.52	(2.36,2.67)	**1.73E-87**	**5.26E-87**	0.00	(-0.11,0.12)	9.40E-01	9.42E-01
123	TG(O-54:2) [NL-18:1]	-0.76	(-0.83,-0.69)	**6.08E-67**	**1.38E-66**	-2.65	(-2.73,-2.57)	**8.64E-225**	**2.11E-224**	1.78	(1.66,1.9)	**1.84E-80**	**4.88E-80**	0.20	(0.09,0.31)	**3.43E-04**	**4.82E-04**
124	TG(O-54:4) [NL-17:1]	-0.70	(-0.77,-0.62)	**6.88E-51**	**1.27E-50**	-3.07	(-3.17,-2.96)	**1.93E-209**	**3.91E-209**	1.80	(1.65,1.94)	**5.98E-66**	**1.23E-65**	0.70	(0.58,0.82)	**2.98E-24**	**2.20E-23**
125	TG(O-54:4) [NL-18:2]	-0.67	(-0.75,-0.6)	**1.38E-48**	**2.49E-48**	-3.39	(-3.51,-3.28)	**1.94E-213**	**4.15E-213**	2.16	(1.99,2.32)	**1.64E-70**	**3.63E-70**	0.69	(0.56,0.82)	**2.85E-21**	**1.59E-20**
126	dhCer(d18:0/22:0)	-0.01	(-0.07,0.05)	6.90E-01	7.05E-01	0.02	(-0.04,0.07)	6.12E-01	6.18E-01	-0.26	(-0.34,-0.17)	**2.28E-08**	**2.61E-08**	0.20	(0.12,0.27)	**6.86E-07**	**1.20E-06**
127	dhCer(d18:0/24:0)	0.05	(0,0.11)	5.11E-02	5.54E-02	0.41	(0.36,0.47)	**7.53E-38**	**8.43E-38**	-0.48	(-0.56,-0.4)	**4.01E-26**	**5.47E-26**	0.09	(0.02,0.16)	**1.41E-02**	**1.72E-02**
128	dhCer(d18:0/24:1)	-0.32	(-0.38,-0.26)	**1.44E-22**	**2.05E-22**	-0.01	(-0.08,0.07)	8.42E-01	8.46E-01	-0.65	(-0.75,-0.55)	**3.24E-27**	**4.47E-27**	0.33	(0.24,0.41)	**2.94E-12**	**8.57E-12**
129	Cer(d16:1/22:0)	0.08	(0.03,0.14)	**1.97E-03**	**2.21E-03**	-2.34	(-2.4,-2.28)	**1.79E-250**	**6.15E-250**	2.23	(2.14,2.33)	**1.21E-122**	**7.38E-122**	0.21	(0.12,0.29)	**2.66E-06**	**4.47E-06**
130	Cer(d16:1/23:0)	0.09	(0.03,0.15)	**4.57E-03**	**5.12E-03**	-3.18	(-3.27,-3.08)	**3.92E-228**	**1.02E-227**	3.27	(3.12,3.42)	**4.68E-114**	**2.44E-113**	0.06	(-0.03,0.16)	2.00E-01	2.17E-01
131	Cer(d16:1/24:0)	0.31	(0.25,0.36)	**3.72E-24**	**5.37E-24**	-2.68	(-2.75,-2.62)	**4.04E-274**	**1.90E-273**	2.84	(2.75,2.93)	**1.12E-148**	**1.45E-147**	0.15	(0.06,0.24)	**8.03E-04**	**1.10E-03**
132	Cer(d16:1/24:1)	0.19	(0.14,0.24)	**3.72E-13**	**4.81E-13**	-2.07	(-2.13,-2)	**3.17E-213**	**6.77E-213**	2.02	(1.93,2.12)	**2.46E-107**	**1.04E-106**	0.27	(0.19,0.35)	**7.05E-10**	**1.59E-09**
133	Cer(d17:1/22:0)	0.24	(0.18,0.3)	**8.04E-14**	**1.05E-13**	-1.88	(-1.95,-1.8)	**4.06E-179**	**6.68E-179**	1.94	(1.83,2.05)	**4.13E-95**	**1.45E-94**	0.13	(0.05,0.22)	**2.45E-03**	**3.17E-03**
134	Cer(d17:1/23:0)	0.23	(0.17,0.29)	**2.75E-13**	**3.56E-13**	-3.21	(-3.32,-3.11)	**2.68E-210**	**5.45E-210**	3.40	(3.23,3.56)	**7.83E-109**	**3.42E-108**	0.06	(-0.03,0.15)	1.90E-01	2.08E-01
135	Cer(d17:1/24:0)	0.43	(0.38,0.48)	**1.13E-49**	**2.07E-49**	-2.04	(-2.1,-1.97)	**8.51E-225**	**2.09E-224**	2.39	(2.3,2.48)	**5.93E-130**	**4.99E-129**	0.08	(0.01,0.16)	**3.67E-02**	**4.30E-02**
136	Cer(d17:1/24:1)	0.25	(0.2,0.3)	**9.42E-21**	**1.32E-20**	-2.16	(-2.23,-2.09)	**2.63E-212**	**5.50E-212**	2.20	(2.1,2.31)	**5.11E-113**	**2.58E-112**	0.26	(0.18,0.34)	**1.49E-09**	**3.23E-09**
137	Cer(d18:1/16:0)	-0.26	(-0.3,-0.22)	**3.91E-32**	**6.13E-32**	-2.08	(-2.12,-2.03)	**3.60E-283**	**1.99E-282**	1.62	(1.55,1.68)	**3.20E-124**	**2.08E-123**	0.18	(0.12,0.24)	**2.52E-09**	**5.42E-09**
138	Cer(d18:1/18:0)	-0.12	(-0.17,-0.07)	**1.11E-05**	**1.32E-05**	-1.61	(-1.67,-1.54)	**2.31E-182**	**3.87E-182**	1.23	(1.15,1.32)	**8.50E-77**	**2.16E-76**	0.23	(0.15,0.31)	**3.14E-08**	**6.18E-08**
139	Cer(d18:1/20:0)	-0.18	(-0.23,-0.13)	**1.55E-13**	**2.02E-13**	-2.02	(-2.07,-1.96)	**2.42E-253**	**8.60E-253**	1.54	(1.47,1.62)	**8.02E-108**	**3.41E-107**	0.31	(0.24,0.38)	**3.47E-16**	**1.38E-15**
140	Cer(d18:1/22:0)	0.15	(0.11,0.19)	**3.55E-12**	**4.50E-12**	-2.27	(-2.31,-2.22)	**9.11E-296**	**7.29E-295**	2.13	(2.07,2.2)	**2.49E-149**	**3.32E-148**	0.28	(0.22,0.34)	**1.65E-16**	**6.76E-16**
141	Cer(d18:1/23:0)	0.08	(0.04,0.12)	**2.74E-04**	**3.15E-04**	-3.78	(-3.84,-3.72)	**0.00E+00**	**0.00E+00**	3.72	(3.64,3.8)	**2.94E-187**	**2.02E-185**	0.17	(0.11,0.24)	**5.66E-07**	**9.96E-07**
142	Cer(d18:1/24:0)	0.23	(0.19,0.27)	**1.57E-24**	**2.28E-24**	-2.02	(-2.07,-1.97)	**2.26E-264**	**8.88E-264**	2.04	(1.97,2.1)	**1.42E-147**	**1.70E-146**	0.17	(0.11,0.23)	**7.51E-08**	**1.42E-07**
143	Cer(d18:1/24:1)	0.03	(-0.02,0.07)	2.08E-01	2.19E-01	-2.24	(-2.3,-2.19)	**6.68E-263**	**2.59E-262**	1.91	(1.83,1.99)	**2.27E-124**	**1.50E-123**	0.34	(0.28,0.4)	**8.98E-23**	**5.60E-22**
144	Cer(d18:2/16:0)	-0.37	(-0.43,-0.31)	**4.51E-31**	**7.02E-31**	-2.21	(-2.28,-2.14)	**4.97E-220**	**1.15E-219**	1.29	(1.18,1.4)	**1.62E-61**	**3.16E-61**	0.48	(0.4,0.56)	**2.44E-25**	**2.02E-24**
145	Cer(d18:2/22:0)	0.44	(0.39,0.49)	**1.07E-53**	**2.03E-53**	-1.48	(-1.53,-1.42)	**3.06E-193**	**5.51E-193**	1.50	(1.42,1.58)	**2.21E-99**	**8.43E-99**	0.42	(0.35,0.49)	**5.29E-25**	**4.09E-24**
146	Cer(d18:2/23:0)	0.36	(0.31,0.41)	**1.05E-39**	**1.74E-39**	-3.34	(-3.41,-3.26)	**1.75E-281**	**9.57E-281**	3.45	(3.35,3.55)	**3.25E-156**	**5.20E-155**	0.29	(0.22,0.37)	**1.24E-12**	**3.69E-12**
147	Cer(d18:2/24:0)	0.51	(0.46,0.56)	**2.10E-70**	**5.04E-70**	-1.78	(-1.84,-1.72)	**1.40E-212**	**2.94E-212**	1.93	(1.85,2.01)	**7.46E-123**	**4.65E-122**	0.34	(0.27,0.41)	**2.41E-19**	**1.15E-18**
148	Cer(d18:2/24:1)	0.40	(0.35,0.44)	**9.94E-46**	**1.75E-45**	-1.55	(-1.61,-1.49)	**2.32E-180**	**3.85E-180**	1.50	(1.41,1.58)	**1.27E-94**	**4.37E-94**	0.47	(0.4,0.54)	**9.26E-33**	**2.02E-31**

149	Cer(d19:1/24:0)	0.72	(0.65,0.8)	**7.44E-58**	**1.46E-57**	-0.94	(-1.02,-0.87)	**5.14E-79**	**6.12E-79**	1.73	(1.6,1.86)	**3.14E-73**	**7.34E-73**	-0.13	(-0.25,-0.01)	**3.29E-02**	**3.89E-02**
150	Cer(d19:1/24:1)	0.47	(0.38,0.55)	**1.46E-24**	**2.13E-24**	-1.27	(-1.37,-1.18)	**9.71E-93**	**1.20E-92**	1.64	(1.5,1.78)	**2.74E-61**	**5.28E-61**	0.08	(-0.05,0.21)	2.45E-01	2.63E-01
151	Cer(m18:0/22:0)	-0.90	(-0.97,-0.82)	**1.08E-71**	**2.67E-71**	-2.35	(-2.43,-2.27)	**2.38E-205**	**4.72E-205**	1.28	(1.17,1.39)	**6.27E-60**	**1.19E-59**	0.26	(0.16,0.35)	**1.58E-07**	**2.94E-07**
152	Cer(m18:0/23:0)	-0.96	(-1.04,-0.87)	**8.86E-71**	**2.14E-70**	-4.13	(-4.27,-4)	**4.57E-216**	**1.01E-215**	3.26	(3.05,3.47)	**1.53E-84**	**4.38E-84**	0.12	(0,0.24)	**4.55E-02**	5.27E-02
153	Cer(m18:0/24:0)	-0.72	(-0.79,-0.65)	**1.32E-62**	**2.87E-62**	-2.31	(-2.38,-2.25)	**1.09E-239**	**3.26E-239**	1.37	(1.28,1.47)	**1.34E-80**	**3.57E-80**	0.19	(0.09,0.28)	**9.04E-05**	**1.32E-04**
154	Cer(m18:0/24:1)	-0.93	(-1.01,-0.85)	**3.35E-72**	**8.43E-72**	-2.98	(-3.07,-2.89)	**1.70E-233**	**4.66E-233**	1.62	(1.5,1.75)	**7.03E-70**	**1.54E-69**	0.41	(0.31,0.51)	**2.35E-13**	**7.33E-13**
155	Cer(m18:1/22:0)	-0.54	(-0.61,-0.47)	**3.71E-39**	**6.11E-39**	-1.43	(-1.5,-1.36)	**8.20E-152**	**1.21E-151**	0.33	(0.22,0.43)	**3.35E-09**	**3.86E-09**	0.59	(0.48,0.7)	**1.35E-22**	**8.22E-22**
156	Cer(m18:1/23:0)	-0.54	(-0.62,-0.46)	**3.84E-33**	**6.08E-33**	-3.36	(-3.49,-3.22)	**8.10E-183**	**1.36E-182**	2.34	(2.15,2.53)	**3.19E-67**	**6.72E-67**	0.53	(0.41,0.66)	**2.02E-15**	**7.62E-15**
157	Cer(m18:1/24:0)	-0.33	(-0.4,-0.26)	**3.55E-20**	**4.96E-20**	-1.33	(-1.4,-1.26)	**3.36E-143**	**4.77E-143**	0.41	(0.31,0.51)	**1.59E-13**	**1.91E-13**	0.51	(0.41,0.61)	**2.85E-20**	**1.44E-19**
158	Cer(m18:1/24:1)	-0.56	(-0.63,-0.49)	**3.20E-43**	**5.44E-43**	-2.08	(-2.16,-2)	**4.14E-186**	**7.05E-186**	0.80	(0.68,0.93)	**7.96E-29**	**1.12E-28**	0.68	(0.57,0.78)	**1.41E-29**	**1.78E-28**
159	GM3(d18:1/18:0)	0.53	(0.48,0.57)	**1.14E-73**	**2.90E-73**	0.66	(0.61,0.72)	**9.71E-80**	**1.16E-79**	-0.13	(-0.2,-0.06)	**2.42E-04**	**2.58E-04**	-0.08	(-0.15,-0.02)	**1.62E-02**	**1.96E-02**
160	GM3(d18:1/22:0)	0.05	(0,0.11)	6.58E-02	7.10E-02	-0.98	(-1.05,-0.9)	**5.36E-89**	**6.49E-89**	0.88	(0.79,0.97)	**3.53E-50**	**6.15E-50**	0.12	(0.05,0.19)	**9.07E-04**	**1.23E-03**
161	GM3(d18:1/24:0)	0.48	(0.42,0.54)	**6.03E-46**	**1.07E-45**	0.13	(0.06,0.19)	**2.30E-04**	**2.39E-04**	0.34	(0.26,0.42)	**2.12E-14**	**2.57E-14**	-0.03	(-0.1,0.03)	3.25E-01	3.47E-01
162	GM3(d18:1/24:1)	0.03	(-0.02,0.09)	2.57E-01	2.68E-01	-0.52	(-0.59,-0.45)	**1.26E-42**	**1.41E-42**	0.37	(0.29,0.46)	**1.85E-15**	**2.26E-15**	0.12	(0.05,0.2)	**1.23E-03**	**1.65E-03**
163	HexCer(d16:1/22:0)	0.06	(0.01,0.12)	**3.18E-02**	**3.48E-02**	-2.18	(-2.27,-2.08)	**1.12E-164**	**1.73E-164**	2.04	(1.91,2.17)	**3.98E-86**	**1.18E-85**	0.24	(0.16,0.32)	**4.93E-09**	**1.04E-08**
164	HexCer(d16:1/24:0)	0.34	(0.29,0.4)	**4.06E-27**	**6.09E-27**	-1.75	(-1.84,-1.66)	**2.61E-143**	**3.71E-143**	1.74	(1.62,1.86)	**1.30E-75**	**3.21E-75**	0.27	(0.19,0.34)	**9.05E-11**	**2.22E-10**
165	HexCer(d18:1/16:0)	0.08	(0.04,0.12)	**1.16E-04**	**1.35E-04**	-1.82	(-1.88,-1.76)	**3.76E-212**	**7.82E-212**	1.86	(1.79,1.93)	**1.00E-127**	**6.88E-127**	-0.04	(-0.11,0.02)	1.56E-01	1.72E-01
166	HexCer(d18:1/18:0)	-0.11	(-0.17,-0.05)	**1.41E-04**	**1.63E-04**	-1.33	(-1.4,-1.26)	**1.76E-133**	**2.43E-133**	0.96	(0.86,1.06)	**4.24E-48**	**7.26E-48**	0.22	(0.14,0.3)	**8.84E-08**	**1.67E-07**
167	HexCer(d18:1/20:0)	-0.09	(-0.14,-0.05)	**1.33E-04**	**1.54E-04**	-1.40	(-1.47,-1.33)	**1.47E-150**	**2.17E-150**	1.14	(1.05,1.22)	**2.52E-71**	**5.65E-71**	0.17	(0.1,0.25)	**5.83E-06**	**9.45E-06**
168	HexCer(d18:1/22:0)	0.10	(0.06,0.14)	**2.72E-07**	**3.33E-07**	-1.89	(-1.94,-1.83)	**5.07E-232**	**1.36E-231**	1.77	(1.7,1.84)	**6.98E-127**	**4.72E-126**	0.20	(0.14,0.26)	**1.25E-09**	**2.76E-09**
169	HexCer(d18:1/24:0)	0.24	(0.2,0.28)	**1.18E-26**	**1.76E-26**	-0.85	(-0.9,-0.79)	**5.63E-108**	**7.21E-108**	0.89	(0.82,0.96)	**7.42E-71**	**1.66E-70**	0.16	(0.1,0.22)	**1.31E-06**	**2.24E-06**
170	HexCer(d18:1/24:1)	0.02	(-0.02,0.07)	2.87E-01	2.99E-01	-1.30	(-1.35,-1.24)	**1.65E-169**	**2.62E-169**	1.22	(1.15,1.29)	**4.63E-93**	**1.56E-92**	0.05	(-0.02,0.12)	1.40E-01	1.55E-01
171	Hex2Cer(d16:1/16:0)	0.03	(-0.02,0.08)	2.89E-01	3.00E-01	-3.15	(-3.22,-3.07)	**2.81E-265**	**1.14E-264**	2.59	(2.48,2.7)	**1.29E-122**	**7.72E-122**	0.59	(0.51,0.67)	**1.68E-35**	**6.21E-34**
172	Hex2Cer(d18:1/16:0)	-0.13	(-0.18,-0.09)	**9.14E-09**	**1.14E-08**	-2.34	(-2.39,-2.28)	**1.11E-271**	**4.95E-271**	1.82	(1.75,1.89)	**2.45E-132**	**2.18E-131**	0.37	(0.3,0.43)	**6.83E-24**	**4.82E-23**
173	Hex2Cer(d18:1/22:0)	0.23	(0.18,0.28)	**1.10E-18**	**1.52E-18**	-1.09	(-1.16,-1.02)	**1.97E-118**	**2.63E-118**	1.01	(0.93,1.09)	**1.16E-69**	**2.52E-69**	0.27	(0.2,0.34)	**8.93E-13**	**2.70E-12**
174	Hex2Cer(d18:1/24:1)	0.37	(0.33,0.42)	**2.02E-45**	**3.55E-45**	-1.62	(-1.68,-1.56)	**2.27E-199**	**4.26E-199**	1.75	(1.68,1.82)	**2.84E-128**	**2.09E-127**	0.20	(0.13,0.27)	**1.27E-08**	**2.60E-08**
175	Hex2Cer(d18:2/16:0)	0.02	(-0.03,0.06)	4.68E-01	4.83E-01	-1.99	(-2.05,-1.93)	**2.02E-225**	**5.08E-225**	1.39	(1.32,1.47)	**5.54E-100**	**2.13E-99**	0.57	(0.51,0.64)	**9.06E-45**	**8.70E-43**
176	Hex3Cer(d18:1/16:0)	0.00	(-0.04,0.03)	8.86E-01	8.89E-01	-1.09	(-1.13,-1.04)	**3.57E-175**	**5.77E-175**	1.02	(0.96,1.08)	**1.11E-93**	**3.79E-93**	0.00	(-0.06,0.05)	9.23E-01	9.27E-01
177	Hex3Cer(d18:1/22:0)	0.43	(0.38,0.49)	**4.17E-41**	**7.00E-41**	-1.19	(-1.26,-1.12)	**5.31E-115**	**6.98E-115**	1.73	(1.64,1.83)	**4.61E-99**	**1.74E-98**	-0.15	(-0.22,-0.07)	**2.26E-04**	**3.24E-04**
178	Hex3Cer(d18:1/24:0)	0.53	(0.48,0.58)	**7.29E-66**	**1.64E-65**	-0.10	(-0.16,-0.04)	**1.44E-03**	**1.49E-03**	0.78	(0.7,0.86)	**2.14E-49**	**3.69E-49**	-0.18	(-0.25,-0.11)	**2.84E-07**	**5.14E-07**
179	Hex3Cer(d18:1/24:1)	0.35	(0.3,0.4)	**1.95E-37**	**3.19E-37**	-0.95	(-1.01,-0.9)	**3.88E-115**	**5.12E-115**	1.27	(1.19,1.35)	**2.83E-85**	**8.18E-85**	-0.03	(-0.1,0.03)	3.25E-01	3.47E-01
180	SM(d17:1/14:0)	-0.18	(-0.22,-0.13)	**2.46E-14**	**3.24E-14**	-1.63	(-1.68,-1.59)	**1.14E-240**	**3.45E-240**	1.15	(1.07,1.23)	**2.29E-75**	**5.61E-75**	0.28	(0.2,0.36)	**2.80E-11**	**7.24E-11**
181	SM(d18:0/14:0)	-0.21	(-0.26,-0.16)	**6.88E-17**	**9.30E-17**	0.05	(0,0.1)	**3.13E-02**	**3.21E-02**	-0.42	(-0.5,-0.35)	**1.45E-25**	**1.97E-25**	0.13	(0.06,0.2)	**5.48E-04**	**7.56E-04**
182	SM(d18:1/14:0)/SM(d16:1/16:0)	-0.19	(-0.23,-0.16)	**2.17E-22**	**3.09E-22**	-1.92	(-1.96,-1.88)	**3.24E-291**	**2.13E-290**	1.43	(1.36,1.5)	**1.46E-112**	**7.14E-112**	0.26	(0.2,0.32)	**4.34E-15**	**1.57E-14**
183	SM(d18:2/14:0)	-0.18	(-0.22,-0.14)	**3.36E-16**	**4.50E-16**	-1.12	(-1.17,-1.08)	**7.67E-181**	**1.28E-180**	0.33	(0.26,0.41)	**8.37E-18**	**1.06E-17**	0.57	(0.5,0.64)	**4.36E-41**	**3.49E-39**
184	SM(d17:1/16:0)	-0.15	(-0.19,-0.12)	**4.55E-17**	**6.17E-17**	-1.91	(-1.95,-1.87)	**4.05E-292**	**2.90E-291**	1.50	(1.43,1.57)	**1.18E-115**	**6.36E-115**	0.21	(0.14,0.27)	**3.81E-10**	**8.84E-10**
185	SM(d18:1/16:0)	-0.33	(-0.37,-0.28)	**1.02E-35**	**1.65E-35**	-2.37	(-2.42,-2.32)	**2.40E-291**	**1.62E-290**	1.71	(1.63,1.78)	**3.05E-123**	**1.95E-122**	0.30	(0.23,0.37)	**1.22E-16**	**5.08E-16**
186	SM(d18:2/16:0)	-0.17	(-0.2,-0.14)	**7.00E-25**	**1.02E-24**	-1.25	(-1.29,-1.21)	**1.34E-230**	**3.53E-230**	0.55	(0.49,0.6)	**1.13E-54**	**2.06E-54**	0.50	(0.45,0.55)	**4.29E-57**	**1.03E-54**
187	SM(34:3)	-0.15	(-0.19,-0.11)	**2.66E-12**	**3.39E-12**	-1.19	(-1.23,-1.14)	**1.59E-193**	**2.87E-193**	0.62	(0.55,0.69)	**2.71E-43**	**4.33E-43**	0.37	(0.3,0.44)	**8.29E-22**	**4.79E-21**
188	SM(d18:2/17:0)	-0.10	(-0.14,-0.07)	**1.79E-08**	**2.21E-08**	-1.18	(-1.23,-1.14)	**1.01E-204**	**1.97E-204**	0.68	(0.61,0.74)	**2.37E-54**	**4.30E-54**	0.37	(0.31,0.43)	**8.01E-28**	**8.18E-27**
189	SM(35:2) (b)	-0.34	(-0.39,-0.29)	**3.42E-34**	**5.49E-34**	-2.57	(-2.62,-2.51)	**1.88E-278**	**9.80E-278**	1.80	(1.71,1.89)	**1.86E-106**	**7.69E-106**	0.37	(0.29,0.45)	**1.34E-17**	**6.03E-17**
190	SM(d18:1/18:0)/SM(d16:1/20:0)	-0.32	(-0.36,-0.28)	**1.64E-40**	**2.74E-40**	-1.07	(-1.12,-1.03)	**4.94E-163**	**7.58E-163**	0.52	(0.45,0.58)	**9.34E-36**	**1.40E-35**	0.20	(0.14,0.26)	**2.42E-10**	**5.74E-10**
191	SM(d18:2/18:1)	-0.22	(-0.26,-0.18)	**6.66E-27**	**9.96E-27**	-1.61	(-1.66,-1.57)	**2.07E-241**	**6.31E-241**	1.13	(1.06,1.19)	**1.04E-92**	**3.45E-92**	0.23	(0.17,0.28)	**3.81E-14**	**1.26E-13**
192	SM(37:1)	-0.34	(-0.39,-0.3)	**8.88E-43**	**1.51E-42**	-2.24	(-2.29,-2.19)	**1.07E-276**	**5.35E-276**	1.62	(1.55,1.7)	**1.10E-112**	**5.44E-112**	0.25	(0.19,0.32)	**1.58E-12**	**4.67E-12**
193	SM(37:2)	-0.41	(-0.46,-0.36)	**1.30E-42**	**2.19E-42**	-2.00	(-2.06,-1.94)	**6.15E-240**	**1.86E-239**	1.03	(0.95,1.11)	**1.39E-69**	**3.02E-69**	0.50	(0.43,0.58)	**1.99E-31**	**3.68E-30**
194	SM(d18:1/20:0)/SM(d16:1/22:0)	-0.34	(-0.37,-0.3)	**5.44E-59**	**1.10E-58**	-1.43	(-1.47,-1.39)	**4.26E-242**	**1.32E-241**	0.90	(0.84,0.96)	**4.37E-81**	**1.18E-80**	0.17	(0.12,0.22)	**1.84E-10**	**4.43E-10**
195	SM(d18:2/20:0)	-0.43	(-0.48,-0.39)	**1.16E-61**	**2.45E-61**	-1.01	(-1.06,-0.95)	**1.02E-141**	**1.44E-141**	0.15	(0.08,0.23)	**1.20E-04**	**1.29E-04**	0.38	(0.32,0.45)	**5.14E-26**	**4.57E-25**
196	SM(38:3) (a)	-0.43	(-0.47,-0.38)	**6.49E-64**	**1.42E-63**	-1.20	(-1.25,-1.16)	**3.59E-188**	**6.22E-188**	0.32	(0.25,0.38)	**5.92E-18**	**7.53E-18**	0.38	(0.32,0.44)	**8.99E-31**	**1.35E-29**
197	SM(38:3) (b)	-0.36	(-0.42,-0.31)	**6.93E-31**	**1.08E-30**	-2.14	(-2.2,-2.09)	**6.12E-253**	**2.16E-252**	1.23	(1.14,1.31)	**3.99E-74**	**9.54E-74**	0.47	(0.39,0.55)	**2.47E-26**	**2.33E-25**
198	SM(d16:1/23:0)/SM(d17:1/22:0)	-0.30	(-0.33,-0.26)	**1.19E-43**	**2.05E-43**	-2.63	(-2.68,-2.59)	**0.00E+00**	**0.00E+00**	2.23	(2.16,2.3)	**7.33E-152**	**1.07E-150**	0.07	(0.01,0.13)	**2.52E-02**	**2.98E-02**
199	SM(d18:1/22:0)/SM(d16:1/24:0)	-0.35	(-0.39,-0.31)	**2.03E-50**	**3.74E-50**	-2.02	(-2.07,-1.98)	**1.81E-288**	**1.09E-287**	1.45	(1.39,1.51)	**1.12E-119**	**6.45E-119**	0.17	(0.12,0.23)	**8.46E-10**	**1.90E-09**

200	SM(d16:1/24:1)	-0.33	(-0.37,-0.29)	**5.44E-47**	**9.70E-47**	-2.12	(-2.16,-2.07)	**2.38E-286**	**1.39E-285**	1.49	(1.42,1.56)	**6.58E-108**	**2.82E-107**	0.26	(0.2,0.32)	**8.33E-15**	**2.92E-14**
201	SM(d18:2/22:0)	-0.13	(-0.17,-0.1)	**1.23E-12**	**1.57E-12**	-1.25	(-1.29,-1.2)	**1.05E-204**	**2.05E-204**	0.75	(0.69,0.81)	**3.54E-64**	**7.04E-64**	0.33	(0.27,0.38)	**3.13E-27**	**3.07E-26**
202	SM(40:3) (a)	-0.69	(-0.74,-0.65)	**2.74E-107**	**1.43E-106**	-1.57	(-1.62,-1.52)	**7.42E-229**	**1.94E-228**	0.37	(0.29,0.44)	**2.21E-20**	**2.87E-20**	0.44	(0.38,0.5)	**1.28E-37**	**5.58E-36**
203	SM(40:3) (b)	-0.46	(-0.5,-0.41)	**3.55E-58**	**7.13E-58**	-1.98	(-2.02,-1.93)	**3.55E-270**	**1.56E-269**	1.21	(1.13,1.3)	**3.66E-79**	**9.38E-79**	0.24	(0.16,0.31)	**1.11E-09**	**2.46E-09**
204	SM(41:0)	-0.45	(-0.49,-0.42)	**2.96E-72**	**7.48E-72**	-1.96	(-2.02,-1.9)	**8.09E-218**	**1.83E-217**	1.45	(1.36,1.55)	**9.75E-84**	**2.73E-83**	-0.02	(-0.08,0.04)	5.33E-01	5.61E-01
205	SM(41:1)	-0.43	(-0.46,-0.39)	**6.27E-73**	**1.59E-72**	-3.33	(-3.38,-3.28)	**0.00E+00**	**0.00E+00**	2.83	(2.77,2.9)	**4.94E-176**	**1.83E-174**	0.03	(-0.03,0.08)	3.25E-01	3.47E-01
206	SM(d17:1/24:1)	-0.36	(-0.4,-0.33)	**5.29E-60**	**1.11E-59**	-1.81	(-1.85,-1.76)	**1.47E-268**	**6.25E-268**	1.23	(1.16,1.29)	**2.30E-97**	**8.57E-97**	0.16	(0.11,0.22)	**6.72E-08**	**1.28E-07**
207	SM(d18:2/23:0)	-0.21	(-0.24,-0.17)	**2.64E-28**	**4.03E-28**	-3.00	(-3.05,-2.95)	**0.00E+00**	**0.00E+00**	2.59	(2.52,2.66)	**6.11E-164**	**1.33E-162**	0.16	(0.11,0.22)	**7.05E-09**	**1.46E-08**
208	SM(d18:1/24:0)	-0.20	(-0.24,-0.17)	**2.72E-22**	**3.86E-22**	-1.25	(-1.3,-1.2)	**7.07E-199**	**1.32E-198**	0.95	(0.89,1.01)	**3.82E-83**	**1.06E-82**	0.05	(0,0.1)	**3.68E-02**	**4.30E-02**
209	SM(d18:1/24:1)	-0.55	(-0.6,-0.5)	**1.53E-75**	**4.02E-75**	-1.94	(-1.99,-1.89)	**2.31E-255**	**8.34E-255**	1.09	(1.02,1.16)	**2.98E-84**	**8.45E-84**	0.25	(0.19,0.31)	**2.20E-15**	**8.24E-15**
210	SM(d18:2/24:0)	-0.06	(-0.1,-0.03)	**2.15E-04**	**2.47E-04**	-1.07	(-1.11,-1.03)	**1.03E-186**	**1.77E-186**	0.76	(0.7,0.81)	**1.08E-71**	**2.45E-71**	0.20	(0.15,0.24)	**9.46E-15**	**3.29E-14**
211	SM(43:1)	0.04	(0,0.08)	**3.40E-02**	**3.72E-02**	-0.92	(-0.98,-0.86)	**4.34E-113**	**5.64E-113**	1.00	(0.92,1.09)	**2.69E-60**	**5.14E-60**	-0.12	(-0.19,-0.06)	**1.36E-04**	**1.97E-04**
212	SM(44:1)	0.20	(0.17,0.24)	**1.50E-26**	**2.22E-26**	0.54	(0.5,0.58)	**1.07E-91**	**1.31E-91**	-0.33	(-0.39,-0.28)	**3.48E-26**	**4.75E-26**	-0.06	(-0.11,-0.01)	**2.37E-02**	**2.82E-02**
213	SM(44:2)	0.01	(-0.03,0.05)	7.21E-01	7.30E-01	0.16	(0.12,0.21)	**1.63E-12**	**1.73E-12**	-0.30	(-0.36,-0.24)	**7.48E-19**	**9.63E-19**	0.09	(0.04,0.15)	**1.39E-03**	**1.85E-03**
214	SM(44:3) (a)	-0.02	(-0.06,0.02)	2.39E-01	2.51E-01	0.20	(0.16,0.24)	**1.12E-18**	**1.20E-18**	-0.52	(-0.58,-0.46)	**2.07E-45**	**3.41E-45**	0.23	(0.18,0.29)	**7.79E-16**	**3.07E-15**
215	LPC(14:0) [sn2]	0.39	(0.31,0.47)	**1.21E-19**	**1.68E-19**	0.39	(0.32,0.45)	**4.09E-28**	**4.48E-28**	-0.32	(-0.42,-0.22)	**4.65E-10**	**5.43E-10**	0.27	(0.15,0.38)	**5.32E-06**	**8.72E-06**
216	LPC(14:0) [sn1]	0.40	(0.32,0.48)	**8.04E-22**	**1.14E-21**	0.28	(0.22,0.35)	**8.91E-18**	**9.59E-18**	-0.17	(-0.26,-0.08)	**2.91E-04**	**3.10E-04**	0.26	(0.15,0.37)	**5.35E-06**	**8.73E-06**
217	LPC(15:0) [sn2]	0.60	(0.54,0.66)	**9.20E-60**	**1.90E-59**	0.11	(0.06,0.17)	**2.16E-05**	**2.25E-05**	0.23	(0.15,0.32)	**3.17E-07**	**3.55E-07**	0.25	(0.16,0.34)	**2.10E-07**	**3.87E-07**
218	LPC(15:0) [sn1]	0.61	(0.55,0.66)	**2.15E-70**	**5.15E-70**	-0.03	(-0.08,0.02)	1.84E-01	1.87E-01	0.36	(0.28,0.44)	**2.52E-15**	**3.08E-15**	0.28	(0.2,0.37)	**3.62E-10**	**8.44E-10**
219	LPC(16:0) [sn2]	0.60	(0.56,0.65)	**4.03E-90**	**1.39E-89**	0.00	(-0.04,0.04)	9.64E-01	9.64E-01	0.22	(0.15,0.29)	**3.52E-09**	**4.04E-09**	0.38	(0.31,0.44)	**1.18E-25**	**1.01E-24**
220	LPC(16:0) [sn1]	0.71	(0.66,0.76)	**4.53E-90**	**1.54E-89**	-0.09	(-0.14,-0.04)	**1.36E-04**	**1.42E-04**	0.36	(0.28,0.44)	**3.16E-16**	**3.93E-16**	0.45	(0.37,0.52)	**1.47E-26**	**1.41E-25**
221	LPC(16:1) [sn2]	0.84	(0.77,0.9)	**1.79E-82**	**5.37E-82**	1.84	(1.77,1.9)	**1.25E-193**	**2.26E-193**	-1.42	(-1.51,-1.33)	**3.32E-86**	**9.89E-86**	0.41	(0.33,0.48)	**3.61E-22**	**2.14E-21**
222	LPC(16:1) [sn1]	0.88	(0.82,0.94)	**3.62E-99**	**1.51E-98**	1.90	(1.83,1.96)	**7.98E-214**	**1.72E-213**	-1.38	(-1.47,-1.3)	**2.38E-87**	**7.19E-87**	0.36	(0.29,0.44)	**4.62E-19**	**2.17E-18**
223	LPC(15-MHDA) [sn2]	0.95	(0.88,1.02)	**1.13E-87**	**3.64E-87**	0.57	(0.5,0.64)	**5.93E-44**	**6.71E-44**	0.19	(0.07,0.31)	**1.99E-03**	**2.09E-03**	0.14	(0.03,0.26)	**1.06E-02**	**1.31E-02**
224	LPC(15-MHDA) [sn1] / LPC(17:0) [sn2]	0.93	(0.87,0.99)	**3.12E-100**	**1.34E-99**	0.08	(0.02,0.13)	**6.98E-03**	**7.19E-03**	0.63	(0.53,0.73)	**1.92E-28**	**2.69E-28**	0.20	(0.11,0.29)	**3.04E-05**	**4.60E-05**
225	LPC(17:0) [sn1]	0.86	(0.81,0.92)	**8.57E-114**	**5.27E-113**	-0.35	(-0.39,-0.31)	**4.27E-48**	**4.86E-48**	0.94	(0.86,1.02)	**2.90E-65**	**5.92E-65**	0.27	(0.2,0.35)	**1.60E-11**	**4.19E-11**
226	LPC(17:1) [sn1] (a) / LPC(17:1) [sn2] (b)	0.85	(0.78,0.91)	**8.22E-92**	**3.01E-91**	1.14	(1.09,1.2)	**6.84E-152**	**1.01E-151**	-0.56	(-0.64,-0.47)	**4.29E-29**	**6.10E-29**	0.28	(0.19,0.37)	**1.46E-09**	**3.19E-09**
227	LPC(18:0) [sn2]	1.18	(1.13,1.23)	**8.38E-158**	**1.39E-156**	-0.01	(-0.05,0.02)	4.59E-01	4.64E-01	0.98	(0.91,1.05)	**6.27E-75**	**1.52E-74**	0.23	(0.16,0.3)	**3.43E-10**	**8.06E-10**
228	LPC(18:0) [sn1]	1.20	(1.14,1.25)	**6.08E-156**	**8.84E-155**	-0.08	(-0.12,-0.04)	**2.49E-04**	**2.58E-04**	1.06	(0.98,1.13)	**5.87E-76**	**1.47E-75**	0.23	(0.15,0.3)	**8.45E-09**	**1.75E-08**
229	LPC(18:1) [sn2]	1.05	(0.99,1.11)	**1.46E-118**	**1.02E-117**	0.65	(0.6,0.71)	**3.56E-78**	**4.23E-78**	0.17	(0.09,0.26)	**8.92E-05**	**9.58E-05**	0.22	(0.14,0.29)	**5.64E-08**	**1.08E-07**
230	LPC(18:1) [sn1]	1.11	(1.06,1.17)	**3.21E-141**	**3.35E-140**	0.80	(0.75,0.85)	**7.16E-115**	**9.39E-115**	0.21	(0.13,0.28)	**2.25E-07**	**2.53E-07**	0.11	(0.04,0.18)	**1.78E-03**	**2.33E-03**
231	LPC(18:2) [sn2]	1.24	(1.18,1.31)	**7.23E-138**	**7.09E-137**	0.23	(0.18,0.28)	**3.43E-17**	**3.68E-17**	0.81	(0.73,0.89)	**3.50E-52**	**6.27E-52**	0.14	(0.07,0.22)	**2.50E-04**	**3.56E-04**
232	LPC(18:2) [sn1]	1.68	(1.61,1.75)	**1.29E-162**	**2.48E-161**	0.73	(0.68,0.79)	**4.15E-92**	**5.09E-92**	0.74	(0.66,0.82)	**6.78E-44**	**1.09E-43**	0.14	(0.06,0.23)	**7.75E-04**	**1.06E-03**
233	LPC(18:3) [sn2] (a)	0.71	(0.63,0.78)	**2.95E-52**	**5.58E-52**	-0.59	(-0.65,-0.53)	**5.39E-57**	**6.21E-57**	1.26	(1.16,1.36)	**4.68E-67**	**9.80E-67**	0.03	(-0.07,0.13)	5.60E-01	5.86E-01
234	LPC(18:3) [sn1] (a)/LPC(18:3) [sn2] (b)	1.25	(1.17,1.33)	**1.33E-99**	**5.66E-99**	0.99	(0.91,1.06)	**1.09E-90**	**1.32E-90**	0.28	(0.17,0.39)	**1.34E-06**	**1.48E-06**	-0.01	(-0.11,0.1)	8.72E-01	8.81E-01
235	LPC(18:3) (a) [sn1] [104_sn1]	1.31	(1.22,1.4)	**2.73E-100**	**1.18E-99**	1.02	(0.94,1.1)	**4.27E-92**	**5.23E-92**	0.30	(0.19,0.41)	**3.87E-07**	**4.32E-07**	-0.01	(-0.11,0.1)	8.71E-01	8.81E-01
236	LPC(19:0) [sn1] (a) / LPC(19:0) [sn2] (b)	0.81	(0.76,0.86)	**3.73E-112**	**2.24E-111**	-0.34	(-0.39,-0.29)	**8.55E-34**	**9.48E-34**	1.04	(0.96,1.12)	**5.50E-69**	**1.18E-68**	0.12	(0.04,0.19)	**2.22E-03**	**2.88E-03**
237	LPC(20:0) [sn1]	0.95	(0.9,1)	**2.89E-133**	**2.47E-132**	-0.35	(-0.39,-0.31)	**9.29E-50**	**1.06E-49**	1.19	(1.12,1.25)	**1.48E-96**	**5.37E-96**	0.11	(0.05,0.18)	**1.05E-03**	**1.42E-03**
238	LPC(20:1) [sn2]	0.66	(0.61,0.72)	**2.65E-76**	**7.03E-76**	-0.29	(-0.34,-0.25)	**1.28E-34**	**1.42E-34**	0.78	(0.71,0.85)	**3.55E-56**	**6.56E-56**	0.18	(0.1,0.26)	**5.85E-06**	**9.45E-06**
239	LPC(20:1) [sn1]	0.79	(0.73,0.84)	**8.74E-99**	**3.58E-98**	-0.24	(-0.28,-0.2)	**1.63E-25**	**1.77E-25**	0.85	(0.78,0.92)	**1.51E-62**	**2.98E-62**	0.18	(0.11,0.26)	**4.94E-06**	**8.12E-06**
240	LPC(20:2) [sn2]	0.23	(0.18,0.28)	**6.89E-18**	**9.42E-18**	0.37	(0.31,0.42)	**6.39E-34**	**7.10E-34**	-0.36	(-0.43,-0.28)	**8.03E-17**	**1.00E-16**	0.20	(0.13,0.26)	**3.59E-08**	**7.04E-08**
241	LPC(20:2) [sn1]	0.60	(0.55,0.65)	**7.06E-78**	**1.98E-77**	0.85	(0.8,0.9)	**7.89E-122**	**1.06E-121**	-0.46	(-0.54,-0.38)	**1.47E-24**	**1.99E-24**	0.18	(0.11,0.25)	**2.91E-07**	**5.25E-07**
242	LPC(20:3) [sn2]	0.37	(0.31,0.43)	**7.73E-30**	**1.19E-29**	1.52	(1.46,1.58)	**1.56E-192**	**2.76E-192**	-1.34	(-1.43,-1.26)	**2.70E-85**	**7.85E-85**	0.13	(0.06,0.21)	**5.42E-04**	**7.50E-04**
243	LPC(20:3) [sn1]	0.89	(0.83,0.96)	**3.40E-91**	**1.23E-90**	2.28	(2.22,2.34)	**5.13E-244**	**1.65E-243**	-1.54	(-1.64,-1.45)	**1.05E-90**	**3.39E-90**	0.12	(0.04,0.2)	**4.60E-03**	**5.82E-03**
244	LPC(20:3) [104_sn1]	0.94	(0.88,1.01)	**8.24E-95**	**3.16E-94**	2.32	(2.26,2.39)	**1.39E-243**	**4.42E-243**	-1.54	(-1.64,-1.45)	**1.17E-88**	**3.68E-88**	0.11	(0.03,0.19)	**7.49E-03**	**9.34E-03**
245	LPC(20:4) [sn2]	0.87	(0.82,0.92)	**2.69E-117**	**1.79E-116**	2.34	(2.29,2.39)	**2.50E-289**	**1.56E-288**	-1.83	(-1.9,-1.75)	**1.66E-124**	**1.11E-123**	0.28	(0.22,0.35)	**7.93E-16**	**3.09E-15**
246	LPC(20:4) [sn1]	1.32	(1.26,1.38)	**5.07E-152**	**6.76E-151**	3.06	(3.01,3.11)	**0.00E+00**	**0.00E+00**	-2.09	(-2.17,-2.01)	**7.12E-130**	**5.79E-129**	0.28	(0.21,0.35)	**2.27E-13**	**7.13E-13**
247	LPC(20:5) [sn2]	1.16	(1.06,1.25)	**6.68E-74**	**1.71E-73**	0.59	(0.52,0.66)	**3.31E-44**	**3.75E-44**	-0.34	(-0.48,-0.2)	**3.05E-06**	**3.35E-06**	0.83	(0.69,0.97)	**1.46E-25**	**1.23E-24**
248	LPC(20:5) [sn1]	1.51	(1.41,1.62)	**1.93E-98**	**7.79E-98**	1.13	(1.06,1.21)	**2.63E-106**	**3.36E-106**	-0.52	(-0.66,-0.38)	**1.00E-11**	**1.20E-11**	0.84	(0.69,0.98)	**2.69E-25**	**2.19E-24**
249	LPC(22:4) [sn2]	0.22	(0.16,0.28)	**2.55E-13**	**3.31E-13**	0.88	(0.82,0.93)	**1.66E-114**	**2.17E-114**	-0.50	(-0.58,-0.42)	**8.64E-28**	**1.20E-27**	-0.20	(-0.27,-0.13)	**1.45E-07**	**2.70E-07**
250	LPC(22:4) [sn1]	0.61	(0.56,0.67)	**5.36E-67**	**1.23E-66**	1.69	(1.64,1.75)	**1.93E-217**	**4.32E-217**	-0.92	(-1,-0.84)	**5.42E-61**	**1.04E-60**	-0.17	(-0.25,-0.1)	**1.25E-05**	**1.95E-05**

251	LPC(22:5) [sn2] (n3)	0.88	(0.82,0.94)	**1.65E-95**	**6.48E-95**	0.42	(0.36,0.47)	**4.27E-38**	**4.80E-38**	0.22	(0.13,0.31)	**3.63E-06**	**3.97E-06**	0.20	(0.13,0.28)	**1.12E-07**	**2.11E-07**
252	LPC(22:5) [sn1] (n3)/LPC(22:5) [sn2] (n6)	1.04	(0.98,1.1)	**3.77E-120**	**2.66E-119**	0.72	(0.67,0.78)	**8.65E-86**	**1.04E-85**	0.21	(0.13,0.29)	**4.17E-07**	**4.64E-07**	0.07	(-0.01,0.14)	8.30E-02	9.42E-02
253	LPC(22:5) [sn1] (n6)	0.68	(0.61,0.75)	**5.96E-58**	**1.18E-57**	1.48	(1.4,1.55)	**5.10E-140**	**7.14E-140**	-0.53	(-0.63,-0.42)	**1.64E-18**	**2.10E-18**	-0.30	(-0.39,-0.21)	**7.76E-11**	**1.94E-10**
254	LPC(22:5) (n3) [sn1] [104_sn1]	1.35	(1.28,1.42)	**2.39E-134**	**2.17E-133**	0.72	(0.66,0.78)	**1.34E-85**	**1.61E-85**	0.40	(0.31,0.5)	**3.21E-16**	**3.98E-16**	0.16	(0.08,0.24)	**1.34E-04**	**1.94E-04**
255	LPC(22:6) [sn2]	0.45	(0.39,0.5)	**1.20E-43**	**2.05E-43**	0.67	(0.61,0.72)	**1.71E-87**	**2.06E-87**	-0.60	(-0.69,-0.52)	**4.76E-31**	**6.88E-31**	0.31	(0.23,0.39)	**9.79E-14**	**3.15E-13**
256	LPC(22:6) [sn1]	0.92	(0.86,0.98)	**2.77E-102**	**1.26E-101**	1.35	(1.3,1.41)	**2.57E-179**	**4.23E-179**	-0.78	(-0.87,-0.69)	**6.85E-42**	**1.09E-41**	0.28	(0.2,0.36)	**1.04E-10**	**2.54E-10**
257	LPC(O-16:0)	1.08	(1.03,1.12)	**9.33E-157**	**1.44E-155**	0.86	(0.82,0.9)	**1.99E-158**	**3.01E-158**	-0.16	(-0.23,-0.09)	**1.05E-05**	**1.13E-05**	0.36	(0.29,0.42)	**2.70E-21**	**1.53E-20**
258	LPC(O-18:0)	0.89	(0.84,0.95)	**1.14E-110**	**6.53E-110**	-0.15	(-0.19,-0.1)	**5.15E-09**	**5.43E-09**	0.80	(0.72,0.88)	**1.96E-48**	**3.37E-48**	0.23	(0.15,0.32)	**4.46E-08**	**8.67E-08**
259	LPC(O-18:1)	0.99	(0.94,1.04)	**3.86E-142**	**4.21E-141**	0.56	(0.52,0.6)	**1.60E-96**	**2.01E-96**	0.19	(0.11,0.26)	**9.16E-07**	**1.01E-06**	0.23	(0.16,0.29)	**8.51E-11**	**2.11E-10**
260	LPC(O-20:0)	0.91	(0.86,0.96)	**8.82E-116**	**5.72E-115**	-0.03	(-0.09,0.04)	4.26E-01	4.31E-01	0.81	(0.71,0.91)	**2.32E-39**	**3.59E-39**	0.14	(0.06,0.22)	**4.06E-04**	**5.68E-04**
261	LPC(O-20:1)	0.97	(0.89,1.04)	**1.73E-81**	**5.09E-81**	0.01	(-0.06,0.09)	7.09E-01	7.14E-01	0.31	(0.2,0.42)	**3.74E-08**	**4.25E-08**	0.59	(0.5,0.67)	**8.39E-31**	**1.30E-29**
262	LPC(O-22:0)	0.47	(0.42,0.51)	**2.15E-71**	**5.28E-71**	-0.17	(-0.21,-0.13)	**1.27E-13**	**1.36E-13**	0.37	(0.32,0.42)	**4.71E-32**	**6.91E-32**	0.22	(0.17,0.28)	**5.11E-14**	**1.68E-13**
263	LPC(O-22:1)	0.70	(0.65,0.75)	**1.16E-97**	**4.66E-97**	-0.15	(-0.19,-0.1)	**3.34E-10**	**3.53E-10**	0.58	(0.51,0.65)	**2.00E-39**	**3.12E-39**	0.25	(0.17,0.32)	**5.43E-10**	**1.25E-09**
264	LPC(O-24:0)	0.14	(0.09,0.18)	**3.11E-09**	**3.91E-09**	-0.90	(-0.95,-0.85)	**1.04E-130**	**1.43E-130**	0.79	(0.74,0.85)	**5.47E-74**	**1.30E-73**	0.21	(0.15,0.27)	**6.54E-12**	**1.79E-11**
265	LPC(O-24:1)	0.26	(0.22,0.3)	**1.71E-28**	**2.62E-28**	-0.81	(-0.86,-0.76)	**6.29E-120**	**8.46E-120**	0.81	(0.75,0.88)	**3.23E-70**	**7.10E-70**	0.22	(0.16,0.28)	**8.25E-12**	**2.24E-11**
266	LPC(O-24:2)	0.45	(0.4,0.5)	**1.19E-53**	**2.27E-53**	0.06	(0.01,0.1)	**1.70E-02**	**1.75E-02**	0.02	(-0.05,0.09)	5.97E-01	6.01E-01	0.34	(0.26,0.41)	**5.74E-17**	**2.47E-16**
267	LPC(P-16:0)	1.37	(1.32,1.42)	**2.24E-180**	**9.77E-179**	0.86	(0.82,0.9)	**4.71E-161**	**7.18E-161**	0.05	(-0.02,0.12)	1.81E-01	1.85E-01	0.44	(0.37,0.51)	**4.38E-31**	**7.51E-30**
268	LPC(P-18:0)	0.99	(0.94,1.03)	**2.00E-141**	**2.14E-140**	0.56	(0.52,0.6)	**1.07E-93**	**1.32E-93**	0.21	(0.13,0.28)	**7.28E-08**	**8.23E-08**	0.23	(0.16,0.29)	**2.41E-10**	**5.74E-10**
269	LPC(P-18:1)	1.24	(1.18,1.29)	**1.82E-144**	**2.13E-143**	-0.10	(-0.15,-0.05)	**1.97E-04**	**2.05E-04**	0.78	(0.69,0.87)	**2.80E-44**	**4.55E-44**	0.52	(0.44,0.6)	**7.00E-29**	**8.00E-28**
270	LPC(P-20:0)	0.87	(0.81,0.92)	**3.13E-104**	**1.49E-103**	-0.12	(-0.17,-0.07)	**1.37E-06**	**1.44E-06**	0.81	(0.73,0.9)	**4.55E-52**	**8.11E-52**	0.18	(0.1,0.26)	**8.73E-06**	**1.39E-05**
271	LPE(16:0) [sn2]	-0.16	(-0.22,-0.11)	**1.02E-08**	**1.27E-08**	-0.13	(-0.18,-0.08)	**2.29E-07**	**2.41E-07**	-0.53	(-0.61,-0.45)	**1.94E-30**	**2.79E-30**	0.47	(0.4,0.54)	**1.28E-31**	**2.46E-30**
272	LPE(16:0) [sn1]	-0.24	(-0.29,-0.19)	**2.66E-18**	**3.66E-18**	-0.23	(-0.28,-0.18)	**1.77E-19**	**1.91E-19**	-0.51	(-0.58,-0.43)	**6.59E-30**	**9.42E-30**	0.48	(0.41,0.55)	**3.39E-34**	**9.05E-33**
273	LPE(18:0) [sn2]	0.22	(0.17,0.27)	**1.89E-17**	**2.58E-17**	-0.32	(-0.37,-0.28)	**4.59E-38**	**5.15E-38**	0.17	(0.1,0.24)	**2.00E-06**	**2.20E-06**	0.36	(0.3,0.42)	**2.59E-24**	**1.95E-23**
274	LPE(18:0) [sn1]	0.13	(0.08,0.18)	**5.51E-08**	**6.79E-08**	-0.43	(-0.47,-0.39)	**4.96E-62**	**5.78E-62**	0.20	(0.13,0.27)	**2.42E-08**	**2.76E-08**	0.37	(0.31,0.43)	**3.93E-25**	**3.14E-24**
275	LPE(18:1) [sn2]	0.05	(-0.02,0.12)	1.47E-01	1.55E-01	-0.29	(-0.34,-0.24)	**7.85E-25**	**8.54E-25**	0.20	(0.11,0.29)	**1.02E-05**	**1.12E-05**	0.09	(0,0.18)	**4.08E-02**	**4.75E-02**
276	LPE(18:1) [sn1]	0.07	(0.01,0.13)	**3.44E-02**	**3.75E-02**	-0.33	(-0.39,-0.28)	**1.63E-30**	**1.80E-30**	0.31	(0.22,0.4)	**5.22E-11**	**6.15E-11**	0.09	(0,0.18)	**4.82E-02**	5.55E-02
277	LPE(18:2) [sn2]	0.73	(0.66,0.81)	**5.08E-62**	**1.09E-61**	-0.26	(-0.33,-0.2)	**1.39E-14**	**1.49E-14**	0.86	(0.76,0.96)	**5.86E-44**	**9.47E-44**	0.13	(0.05,0.22)	**3.09E-03**	**3.98E-03**
278	LPE(18:2) [sn1]	0.92	(0.84,0.99)	**1.21E-79**	**3.42E-79**	-0.03	(-0.09,0.03)	3.17E-01	3.22E-01	0.84	(0.74,0.94)	**3.43E-43**	**5.47E-43**	0.12	(0.04,0.21)	**5.45E-03**	**6.83E-03**
279	LPE(20:4) [sn1]	1.03	(0.97,1.08)	**1.48E-124**	**1.07E-123**	2.19	(2.14,2.25)	**7.71E-263**	**2.96E-262**	-1.35	(-1.42,-1.27)	**6.01E-97**	**2.20E-96**	0.18	(0.12,0.24)	**1.88E-08**	**3.77E-08**
280	LPE(22:6) [sn2]	0.36	(0.3,0.42)	**4.81E-28**	**7.30E-28**	1.53	(1.47,1.58)	**8.90E-203**	**1.70E-202**	-1.68	(-1.77,-1.6)	**9.92E-105**	**4.04E-104**	0.51	(0.43,0.59)	**1.38E-29**	**1.78E-28**
281	LPE(22:6) [sn1]	0.62	(0.56,0.68)	**2.03E-62**	**4.40E-62**	1.87	(1.81,1.92)	**2.09E-233**	**5.66E-233**	-1.76	(-1.84,-1.68)	**8.29E-112**	**3.98E-111**	0.50	(0.42,0.57)	**5.79E-31**	**9.59E-30**
282	PC(28:0)	-0.79	(-0.9,-0.67)	**2.16E-34**	**3.47E-34**	-1.92	(-2.01,-1.84)	**4.21E-159**	**6.37E-159**	1.06	(0.95,1.18)	**3.75E-46**	**6.25E-46**	0.04	(-0.11,0.18)	6.16E-01	6.37E-01
283	PC(14:0_16:0)	-0.97	(-1.05,-0.9)	**2.56E-86**	**8.20E-86**	-1.74	(-1.81,-1.67)	**1.33E-182**	**2.23E-182**	0.59	(0.5,0.68)	**5.35E-29**	**7.57E-29**	0.15	(0.06,0.23)	**1.25E-03**	**1.67E-03**
284	PC(31:0) (a)	-0.83	(-0.93,-0.73)	**2.10E-45**	**3.68E-45**	-1.79	(-1.88,-1.7)	**5.90E-145**	**8.48E-145**	1.21	(1.06,1.36)	**5.26E-39**	**8.12E-39**	-0.28	(-0.44,-0.13)	**2.92E-04**	**4.14E-04**
285	PC(31:0) (b)	-0.64	(-0.7,-0.58)	**2.23E-67**	**5.17E-67**	-1.19	(-1.27,-1.12)	**3.06E-115**	**4.04E-115**	0.53	(0.43,0.64)	**1.50E-20**	**1.95E-20**	-0.01	(-0.09,0.08)	9.10E-01	9.16E-01
286	PC(31:1)	-1.09	(-1.18,-1)	**8.11E-78**	**2.26E-77**	-1.61	(-1.69,-1.53)	**5.63E-142**	**7.97E-142**	0.37	(0.25,0.48)	**2.67E-09**	**3.08E-09**	0.15	(0.03,0.27)	**1.64E-02**	**1.99E-02**
287	PC(16:0_16:0)	-0.71	(-0.76,-0.66)	**3.93E-99**	**1.63E-98**	-1.00	(-1.05,-0.94)	**1.05E-128**	**1.43E-128**	0.07	(0,0.14)	6.63E-02	6.87E-02	0.19	(0.13,0.25)	**3.48E-10**	**8.14E-10**
288	PC(32:1)	-1.20	(-1.29,-1.12)	**1.98E-94**	**7.53E-94**	-1.18	(-1.26,-1.1)	**1.39E-103**	**1.76E-103**	-0.13	(-0.24,-0.03)	**1.17E-02**	**1.23E-02**	0.12	(0.03,0.21)	**1.00E-02**	**1.24E-02**
289	PC(32:2)	-1.05	(-1.13,-0.97)	**1.28E-89**	**4.26E-89**	-3.52	(-3.59,-3.46)	**0.00E+00**	**0.00E+00**	2.30	(2.2,2.39)	**5.13E-119**	**2.90E-118**	0.16	(0.06,0.27)	**2.53E-03**	**3.28E-03**
290	PC(33:0) (a)	-0.81	(-0.88,-0.73)	**1.06E-68**	**2.47E-68**	-1.00	(-1.08,-0.92)	**1.55E-87**	**1.87E-87**	0.15	(0.02,0.28)	**2.18E-02**	**2.27E-02**	-0.01	(-0.12,0.11)	9.04E-01	9.12E-01
291	PC(33:0) (b)	-0.38	(-0.43,-0.32)	**2.54E-36**	**4.15E-36**	-1.45	(-1.51,-1.39)	**4.13E-166**	**6.47E-166**	0.96	(0.86,1.05)	**2.34E-52**	**4.21E-52**	0.11	(0.04,0.19)	**4.61E-03**	**5.82E-03**
292	PC(33:1)	-1.17	(-1.24,-1.1)	**6.03E-111**	**3.49E-110**	-2.30	(-2.36,-2.23)	**8.93E-242**	**2.75E-241**	1.05	(0.96,1.15)	**2.28E-58**	**4.28E-58**	0.08	(-0.01,0.18)	8.69E-02	9.84E-02
293	PC(33:2)	-0.83	(-0.88,-0.77)	**1.16E-96**	**4.59E-96**	-4.20	(-4.26,-4.15)	**0.00E+00**	**0.00E+00**	3.10	(3.01,3.19)	**6.57E-157**	**1.17E-155**	0.24	(0.16,0.33)	**4.87E-08**	**9.42E-08**
294	PC(16:0_18:0)	0.08	(0.05,0.12)	**2.42E-05**	**2.85E-05**	-0.86	(-0.91,-0.82)	**2.12E-135**	**2.95E-135**	0.77	(0.71,0.83)	**1.28E-66**	**2.65E-66**	0.18	(0.12,0.23)	**4.66E-09**	**9.90E-09**
295	PC(16:0_18:1)	-1.07	(-1.13,-1.01)	**7.65E-122**	**5.48E-121**	-2.39	(-2.45,-2.33)	**2.30E-263**	**8.98E-263**	1.27	(1.18,1.35)	**5.88E-81**	**1.58E-80**	0.07	(0,0.15)	5.10E-02	5.86E-02
296	PC(16:0_18:2)	-0.55	(-0.59,-0.52)	**1.55E-106**	**7.71E-106**	-3.55	(-3.6,-3.49)	**0.00E+00**	**0.00E+00**	2.80	(2.73,2.88)	**3.80E-167**	**1.01E-165**	0.18	(0.13,0.23)	**7.52E-11**	**1.89E-10**
297	PC(16:1_18:2)	-0.70	(-0.75,-0.64)	**9.18E-78**	**2.55E-77**	-3.01	(-3.06,-2.95)	**0.00E+00**	**0.00E+00**	1.95	(1.86,2.03)	**7.01E-119**	**3.91E-118**	0.37	(0.3,0.44)	**1.23E-20**	**6.48E-20**
298	PC(16:0_18:3) (a)	-1.04	(-1.11,-0.97)	**3.63E-101**	**1.58E-100**	-3.68	(-3.75,-3.61)	**0.00E+00**	**0.00E+00**	2.51	(2.42,2.6)	**1.84E-133**	**1.67E-132**	0.13	(0.05,0.22)	**1.88E-03**	**2.46E-03**
299	PC(14:0_20:4)	-0.97	(-1.05,-0.89)	**1.07E-77**	**2.94E-77**	-3.02	(-3.09,-2.95)	**1.38E-280**	**7.37E-280**	1.76	(1.65,1.86)	**8.48E-89**	**2.68E-88**	0.28	(0.16,0.4)	**7.89E-06**	**1.27E-05**
300	PC(34:5)	-0.55	(-0.66,-0.43)	**3.15E-19**	**4.35E-19**	-3.53	(-3.63,-3.44)	**1.85E-251**	**6.38E-251**	2.16	(2.01,2.31)	**3.36E-79**	**8.67E-79**	0.81	(0.64,0.98)	**4.07E-18**	**1.88E-17**
301	PC(15-MHDA_18:1)	-0.54	(-0.61,-0.46)	**6.96E-40**	**1.16E-39**	-1.93	(-2,-1.86)	**2.75E-191**	**4.83E-191**	1.47	(1.35,1.59)	**4.32E-65**	**8.76E-65**	-0.09	(-0.2,0.02)	1.01E-01	1.14E-01

302	PC(17:0_18:1)	-0.59	(-0.64,-0.54)	**3.15E-75**	**8.17E-75**	-1.88	(-1.92,-1.83)	**3.81E-260**	**1.41E-259**	1.29	(1.22,1.37)	**2.04E-94**	**6.99E-94**	0.01	(-0.06,0.08)	7.53E-01	7.69E-01
303	PC(15-MHDA_18:2)	-0.66	(-0.72,-0.59)	**3.71E-58**	**7.43E-58**	-3.26	(-3.33,-3.2)	**2.60E-299**	**2.31E-298**	2.48	(2.37,2.6)	**4.64E-113**	**2.37E-112**	0.08	(-0.03,0.19)	1.36E-01	1.51E-01
304	PC(17:0_18:2)	-0.47	(-0.51,-0.42)	**5.84E-60**	**1.22E-59**	-3.29	(-3.33,-3.24)	**0.00E+00**	**0.00E+00**	2.63	(2.55,2.71)	**2.39E-154**	**3.70E-153**	0.18	(0.1,0.25)	**3.91E-06**	**6.47E-06**
305	PC(17:1_18:2)	-0.77	(-0.82,-0.72)	**2.23E-95**	**8.70E-95**	-2.61	(-2.66,-2.56)	**0.00E+00**	**0.00E+00**	1.63	(1.55,1.71)	**6.28E-103**	**2.49E-102**	0.22	(0.14,0.3)	**2.06E-07**	**3.81E-07**
306	PC(15:0_20:3)	-0.30	(-0.33,-0.28)	**7.50E-65**	**1.67E-64**	-2.84	(-2.9,-2.77)	**8.21E-281**	**4.43E-280**	2.42	(2.33,2.5)	**3.91E-141**	**4.17E-140**	0.12	(0.08,0.16)	**2.09E-08**	**4.18E-08**
307	PC(15:0_20:4)	-0.78	(-0.84,-0.72)	**3.33E-90**	**1.16E-89**	-2.46	(-2.52,-2.4)	**5.47E-269**	**2.36E-268**	1.23	(1.13,1.33)	**3.58E-65**	**7.29E-65**	0.43	(0.34,0.52)	**1.56E-17**	**6.94E-17**
308	PC(35:5)	-0.34	(-0.45,-0.23)	**8.03E-10**	**1.01E-09**	-3.25	(-3.34,-3.16)	**1.49E-247**	**4.96E-247**	2.05	(1.89,2.21)	**5.72E-69**	**1.22E-68**	0.87	(0.7,1.04)	**1.34E-20**	**7.01E-20**
309	PC(36:0)	-0.43	(-0.48,-0.37)	**1.94E-39**	**3.21E-39**	1.06	(1.01,1.12)	**2.47E-148**	**3.60E-148**	-1.53	(-1.61,-1.46)	**5.78E-109**	**2.54E-108**	-0.01	(-0.09,0.07)	8.15E-01	8.31E-01
310	PC(18:0_18:1)	-0.35	(-0.41,-0.29)	**6.71E-27**	**1.00E-26**	-1.28	(-1.34,-1.23)	**2.43E-172**	**3.88E-172**	0.98	(0.91,1.06)	**2.26E-71**	**5.09E-71**	-0.01	(-0.08,0.06)	7.13E-01	7.32E-01
311	PC(18:0_18:2)	-0.07	(-0.12,-0.01)	**1.17E-02**	**1.30E-02**	-3.20	(-3.25,-3.15)	**0.00E+00**	**0.00E+00**	3.05	(2.97,3.13)	**7.85E-172**	**2.51E-170**	0.10	(0.03,0.17)	**6.64E-03**	**8.30E-03**
312	PC(16:0_20:3) (a)	-0.58	(-0.63,-0.53)	**5.90E-67**	**1.34E-66**	-3.44	(-3.5,-3.38)	**0.00E+00**	**0.00E+00**	2.71	(2.62,2.8)	**5.55E-143**	**6.20E-142**	0.19	(0.12,0.27)	**2.28E-06**	**3.85E-06**
313	PC(16:0_20:3) (b)	-1.39	(-1.46,-1.32)	**3.50E-135**	**3.29E-134**	-2.00	(-2.07,-1.93)	**4.78E-214**	**1.04E-213**	0.39	(0.31,0.48)	**6.35E-18**	**8.06E-18**	0.21	(0.13,0.3)	**4.37E-07**	**7.83E-07**
314	PC(18:2_18:2)	-0.16	(-0.23,-0.09)	**1.08E-05**	**1.29E-05**	-5.35	(-5.42,-5.28)	**0.00E+00**	**0.00E+00**	5.00	(4.88,5.12)	**4.60E-177**	**1.84E-175**	0.16	(0.05,0.26)	**4.91E-03**	**6.19E-03**
315	PC(16:1_20:4)	-0.61	(-0.67,-0.55)	**1.45E-61**	**3.07E-61**	-1.48	(-1.54,-1.42)	**6.95E-174**	**1.12E-173**	0.43	(0.33,0.52)	**1.24E-17**	**1.56E-17**	0.43	(0.35,0.5)	**4.29E-23**	**2.79E-22**
316	PC(16:0_20:5)	-0.42	(-0.52,-0.32)	**3.42E-15**	**4.52E-15**	-2.66	(-2.75,-2.58)	**1.23E-227**	**3.17E-227**	1.30	(1.16,1.44)	**9.68E-47**	**1.62E-46**	0.93	(0.78,1.08)	**5.25E-28**	**5.60E-27**
317	PC(36:6)	-1.15	(-1.23,-1.07)	**6.48E-93**	**2.41E-92**	-3.32	(-3.39,-3.25)	**3.29E-300**	**2.98E-299**	1.71	(1.61,1.82)	**8.74E-87**	**2.62E-86**	0.42	(0.3,0.55)	**1.60E-10**	**3.89E-10**
318	PC(15-MHDA_20:4)	-0.84	(-0.9,-0.78)	**2.63E-86**	**8.35E-86**	-1.74	(-1.81,-1.68)	**1.36E-187**	**2.34E-187**	0.61	(0.49,0.73)	**2.96E-20**	**3.84E-20**	0.25	(0.14,0.35)	**6.40E-06**	**1.03E-05**
319	PC(17:0_20:4)	-0.45	(-0.49,-0.4)	**1.37E-54**	**2.63E-54**	-1.06	(-1.12,-1.01)	**1.05E-149**	**1.54E-149**	0.26	(0.17,0.35)	**2.53E-08**	**2.88E-08**	0.33	(0.26,0.41)	**1.60E-15**	**6.11E-15**
320	PC(15:0_22:6)	-0.85	(-0.91,-0.79)	**3.27E-89**	**1.08E-88**	-2.74	(-2.8,-2.68)	**1.78E-288**	**1.08E-287**	1.43	(1.32,1.54)	**2.39E-72**	**5.53E-72**	0.41	(0.31,0.51)	**2.62E-14**	**8.79E-14**
321	PC(38:2)	-0.56	(-0.6,-0.51)	**9.10E-81**	**2.63E-80**	-1.29	(-1.33,-1.24)	**8.52E-204**	**1.64E-203**	0.57	(0.51,0.63)	**1.70E-46**	**2.85E-46**	0.17	(0.11,0.22)	**1.54E-08**	**3.12E-08**
322	PC(18:0_20:3)	-0.47	(-0.53,-0.4)	**2.35E-33**	**3.75E-33**	-0.29	(-0.35,-0.23)	**4.92E-20**	**5.33E-20**	-0.28	(-0.36,-0.19)	**8.47E-10**	**9.84E-10**	0.10	(0.01,0.19)	**3.38E-02**	**3.98E-02**
323	PC(18:1_20:3)	-0.86	(-0.92,-0.8)	**1.76E-101**	**7.75E-101**	-1.78	(-1.84,-1.73)	**1.26E-216**	**2.80E-216**	0.73	(0.65,0.81)	**7.10E-46**	**1.18E-45**	0.20	(0.12,0.27)	**2.20E-07**	**4.02E-07**
324	PC(38:4) (b)	-1.10	(-1.17,-1.03)	**6.30E-107**	**3.22E-106**	-1.78	(-1.85,-1.71)	**7.38E-188**	**1.27E-187**	0.75	(0.67,0.84)	**5.38E-43**	**8.55E-43**	-0.04	(-0.12,0.05)	3.91E-01	4.14E-01
325	PC(18:0_20:4)	0.03	(-0.02,0.08)	2.19E-01	2.30E-01	-0.20	(-0.25,-0.15)	**4.67E-13**	**4.98E-13**	-0.03	(-0.11,0.05)	3.95E-01	4.01E-01	0.25	(0.18,0.32)	**8.54E-12**	**2.28E-11**
326	PC(38:5) (a)	-0.41	(-0.46,-0.37)	**6.44E-52**	**1.21E-51**	-2.26	(-2.31,-2.21)	**1.67E-291**	**1.14E-290**	1.54	(1.47,1.62)	**4.76E-109**	**2.12E-108**	0.31	(0.24,0.38)	**4.75E-18**	**2.17E-17**
327	PC(38:5) (b)	-0.78	(-0.85,-0.71)	**2.34E-70**	**5.56E-70**	-1.94	(-2,-1.87)	**2.00E-207**	**3.97E-207**	0.86	(0.76,0.95)	**8.91E-45**	**1.46E-44**	0.30	(0.19,0.4)	**2.87E-08**	**5.70E-08**
328	PC(38:6) (a)	-0.13	(-0.18,-0.07)	**4.12E-06**	**4.94E-06**	-2.94	(-3,-2.88)	**2.89E-295**	**2.24E-294**	2.38	(2.29,2.47)	**8.17E-128**	**5.68E-127**	0.43	(0.35,0.51)	**7.19E-22**	**4.21E-21**
329	PC(16:0_22:6)	-0.88	(-0.94,-0.82)	**1.05E-101**	**4.66E-101**	-1.66	(-1.71,-1.6)	**5.33E-213**	**1.13E-212**	0.28	(0.19,0.37)	**1.59E-09**	**1.85E-09**	0.41	(0.33,0.49)	**2.92E-20**	**1.46E-19**
330	PC(18:2_20:5)	0.12	(0.03,0.21)	**1.04E-02**	**1.15E-02**	-3.30	(-3.38,-3.22)	**2.13E-276**	**1.03E-275**	2.76	(2.64,2.89)	**3.20E-113**	**1.65E-112**	0.68	(0.55,0.81)	**1.23E-20**	**6.48E-20**
331	PC(16:1_22:6)	-0.72	(-0.78,-0.67)	**6.63E-79**	**1.87E-78**	-1.72	(-1.77,-1.66)	**1.08E-218**	**2.47E-218**	0.50	(0.41,0.59)	**8.17E-24**	**1.08E-23**	0.47	(0.38,0.56)	**1.10E-20**	**5.94E-20**
332	PC(38:7)(c)	-0.85	(-0.9,-0.8)	**3.27E-115**	**2.09E-114**	-1.93	(-1.97,-1.88)	**1.24E-268**	**5.32E-268**	0.63	(0.55,0.7)	**6.13E-40**	**9.58E-40**	0.39	(0.31,0.46)	**4.00E-21**	**2.21E-20**
333	PC(39:5)(a)	-0.58	(-0.64,-0.51)	**1.03E-49**	**1.89E-49**	-2.08	(-2.15,-2.01)	**6.44E-216**	**1.41E-215**	1.16	(1.05,1.28)	**4.54E-51**	**8.01E-51**	0.28	(0.17,0.39)	**7.38E-07**	**1.28E-06**
334	PC(39:5)(b)	-0.70	(-0.77,-0.64)	**3.50E-67**	**8.08E-67**	-2.15	(-2.22,-2.09)	**8.22E-232**	**2.19E-231**	1.15	(1.05,1.26)	**2.53E-56**	**4.69E-56**	0.25	(0.14,0.35)	**3.67E-06**	**6.11E-06**
335	PC(15-MHDA_22:6)	-0.48	(-0.54,-0.42)	**7.01E-40**	**1.16E-39**	-2.08	(-2.15,-2.01)	**1.87E-211**	**3.81E-211**	1.35	(1.24,1.47)	**2.44E-62**	**4.80E-62**	0.20	(0.09,0.3)	**2.04E-04**	**2.93E-04**
336	PC(17:0_22:6)	-0.79	(-0.85,-0.73)	**1.37E-83**	**4.22E-83**	-2.13	(-2.19,-2.07)	**1.96E-247**	**6.48E-247**	0.88	(0.77,0.99)	**2.44E-38**	**3.73E-38**	0.38	(0.28,0.48)	**3.38E-12**	**9.75E-12**
337	PC(18:0_22:5) (n6)	-0.24	(-0.31,-0.17)	**5.42E-12**	**6.86E-12**	-1.83	(-1.92,-1.74)	**3.38E-147**	**4.91E-147**	1.42	(1.28,1.57)	**3.43E-50**	**5.99E-50**	0.04	(-0.05,0.13)	3.78E-01	4.02E-01
338	PC(18:0_22:6)	-0.55	(-0.61,-0.49)	**8.13E-48**	**1.46E-47**	-1.30	(-1.36,-1.24)	**5.90E-165**	**9.16E-165**	0.36	(0.26,0.46)	**1.02E-11**	**1.22E-11**	0.34	(0.25,0.43)	**2.19E-12**	**6.40E-12**
339	PC(40:7) (a)	-0.51	(-0.57,-0.45)	**1.38E-48**	**2.49E-48**	-1.30	(-1.37,-1.22)	**2.64E-125**	**3.59E-125**	0.44	(0.33,0.56)	**1.22E-12**	**1.47E-12**	0.31	(0.23,0.39)	**2.78E-13**	**8.61E-13**
340	PC(18:1_22:6) (a)	-0.86	(-0.91,-0.81)	**1.01E-114**	**6.40E-114**	-1.47	(-1.53,-1.42)	**8.38E-193**	**1.49E-192**	0.35	(0.27,0.43)	**1.72E-15**	**2.11E-15**	0.26	(0.19,0.33)	**3.62E-12**	**1.03E-11**
341	PC(40:8)	-0.31	(-0.36,-0.26)	**1.61E-32**	**2.54E-32**	-1.57	(-1.62,-1.51)	**3.15E-196**	**5.79E-196**	0.94	(0.86,1.02)	**3.73E-62**	**7.29E-62**	0.30	(0.23,0.37)	**3.04E-15**	**1.12E-14**
342	PC(44:12)	-0.71	(-0.81,-0.6)	**3.01E-33**	**4.79E-33**	-1.75	(-1.83,-1.67)	**4.22E-164**	**6.52E-164**	0.41	(0.27,0.54)	**2.86E-08**	**3.25E-08**	0.60	(0.45,0.74)	**1.80E-14**	**6.12E-14**
343	PC(O-16:0/16:0)	-0.24	(-0.27,-0.2)	**2.56E-31**	**4.00E-31**	-0.82	(-0.87,-0.77)	**1.39E-126**	**1.89E-126**	0.47	(0.41,0.54)	**1.17E-35**	**1.76E-35**	0.09	(0.04,0.14)	**6.63E-04**	**9.09E-04**
344	PC(O-32:1)	-0.46	(-0.51,-0.41)	**1.41E-59**	**2.90E-59**	-1.05	(-1.11,-1)	**5.94E-141**	**8.34E-141**	0.27	(0.2,0.35)	**1.23E-11**	**1.46E-11**	0.33	(0.27,0.39)	**4.27E-21**	**2.33E-20**
345	PC(O-34:1)	-0.25	(-0.29,-0.21)	**6.63E-34**	**1.06E-33**	-1.42	(-1.46,-1.37)	**4.72E-213**	**1.00E-212**	1.09	(1.03,1.15)	**2.23E-96**	**7.97E-96**	0.06	(0.01,0.11)	**1.67E-02**	**2.02E-02**
346	PC(O-34:2)	-0.31	(-0.37,-0.25)	**5.46E-23**	**7.83E-23**	-3.80	(-3.86,-3.75)	**0.00E+00**	**0.00E+00**	3.17	(3.09,3.26)	**3.21E-166**	**8.12E-165**	0.29	(0.21,0.38)	**5.38E-11**	**1.37E-10**
347	PC(O-34:4)	-0.03	(-0.12,0.07)	5.61E-01	5.78E-01	-2.39	(-2.47,-2.3)	**1.01E-200**	**1.90E-200**	1.34	(1.22,1.46)	**1.17E-58**	**2.20E-58**	1.01	(0.88,1.14)	**3.09E-38**	**1.48E-36**
348	PC(O-35:4)	-0.02	(-0.1,0.06)	6.60E-01	6.77E-01	-2.92	(-3,-2.85)	**2.96E-259**	**1.08E-258**	2.13	(2.01,2.25)	**1.12E-95**	**3.96E-95**	0.78	(0.66,0.9)	**8.39E-29**	**9.37E-28**
349	PC(O-36:0)	-0.27	(-0.31,-0.22)	**3.61E-27**	**5.43E-27**	-1.51	(-1.57,-1.45)	**3.94E-186**	**6.73E-186**	1.17	(1.09,1.25)	**8.09E-76**	**2.01E-75**	0.04	(-0.02,0.1)	1.87E-01	2.05E-01
350	PC(O-18:0/18:1)	-0.15	(-0.19,-0.11)	**2.13E-13**	**2.77E-13**	-1.51	(-1.56,-1.46)	**9.29E-221**	**2.17E-220**	1.32	(1.26,1.38)	**2.47E-111**	**1.16E-110**	0.00	(-0.05,0.06)	8.67E-01	8.80E-01
351	PC(O-18:1/18:1)	-0.12	(-0.15,-0.08)	**6.94E-09**	**8.68E-09**	-1.90	(-1.94,-1.86)	**1.58E-278**	**8.32E-278**	1.64	(1.58,1.7)	**1.61E-136**	**1.55E-135**	0.12	(0.07,0.18)	**8.93E-06**	**1.42E-05**
352	PC(O-18:0/18:2)	-0.19	(-0.24,-0.14)	**2.21E-12**	**2.82E-12**	-3.33	(-3.38,-3.27)	**0.00E+00**	**0.00E+00**	2.92	(2.84,2.99)	**4.24E-172**	**1.46E-170**	0.15	(0.08,0.23)	**2.70E-05**	**4.10E-05**

353	PC(O-18:1/18:2)	-0.24	(-0.29,-0.19)	1.86E-19	2.58E-19	-3.24	(-3.29,-3.19)	0.00E+00	0.00E+00	2.75	(2.67,2.82)	7.14E-164	1.49E-162	0.23	(0.16,0.31)	6.63E-10	1.50E-09
354	PC(O-16:0/20:3)	-0.30	(-0.35,-0.25)	1.47E-26	2.19E-26	-1.93	(-1.98,-1.88)	7.92E-252	2.75E-251	1.48	(1.41,1.55)	5.96E-110	2.67E-109	0.14	(0.07,0.2)	7.66E-05	1.13E-04
355	PC(O-16:0/20:4)	-0.17	(-0.22,-0.13)	3.79E-13	4.88E-13	-1.55	(-1.59,-1.5)	7.04E-225	1.74E-224	1.15	(1.08,1.22)	7.00E-91	2.30E-90	0.17	(0.1,0.23)	4.97E-07	8.84E-07
356	PC(O-36:5)	0.08	(-0.01,0.17)	7.49E-02	8.06E-02	-3.26	(-3.33,-3.2)	2.45E-295	1.93E-294	2.50	(2.39,2.62)	9.98E-113	4.99E-112	0.79	(0.67,0.92)	5.51E-29	6.45E-28
357	PC(O-18:0/20:4)	-0.04	(-0.08,0)	5.34E-02	5.77E-02	-1.12	(-1.17,-1.08)	2.80E-183	4.74E-183	1.05	(0.98,1.11)	4.20E-84	1.19E-83	-0.02	(-0.08,0.05)	6.09E-01	6.32E-01
358	PC(O-38:5)	-0.18	(-0.22,-0.13)	2.90E-15	3.85E-15	-2.15	(-2.2,-2.1)	3.96E-278	2.02E-277	1.86	(1.79,1.93)	3.19E-131	2.78E-130	0.08	(0.02,0.14)	9.44E-03	1.17E-02
359	PC(O-16:0/22:6)	-0.59	(-0.65,-0.53)	3.25E-62	6.99E-62	-2.02	(-2.07,-1.97)	3.43E-267	1.43E-266	0.97	(0.89,1.05)	4.82E-65	9.71E-65	0.36	(0.28,0.44)	1.01E-17	4.58E-17
360	PC(O-40:5)	-0.27	(-0.31,-0.23)	3.32E-36	5.40E-36	-1.92	(-1.96,-1.88)	7.51E-285	4.29E-284	1.56	(1.49,1.62)	3.40E-128	2.47E-127	0.05	(-0.01,0.11)	1.09E-01	1.22E-01
361	PC(O-18:0/22:6)	-0.25	(-0.29,-0.2)	4.21E-23	6.05E-23	-1.45	(-1.49,-1.41)	8.22E-234	2.28E-233	1.03	(0.96,1.1)	1.86E-81	5.07E-81	0.09	(0.03,0.16)	4.28E-03	5.45E-03
362	PC(O-40:7) (a)	-0.44	(-0.49,-0.38)	4.95E-44	8.52E-44	-2.25	(-2.3,-2.2)	6.99E-290	4.42E-289	1.46	(1.38,1.54)	2.18E-96	7.88E-96	0.29	(0.22,0.36)	1.90E-14	6.41E-14
363	PC(P-16:0/14:0)	-0.25	(-0.3,-0.2)	4.38E-20	6.11E-20	-0.96	(-1.02,-0.91)	2.65E-119	3.55E-119	0.45	(0.37,0.54)	3.20E-21	4.19E-21	0.23	(0.15,0.3)	1.37E-08	2.79E-08
364	PC(P-16:0/16:0)	-0.06	(-0.1,-0.01)	9.05E-03	1.01E-02	-0.84	(-0.89,-0.79)	2.17E-111	2.81E-111	0.54	(0.47,0.61)	3.82E-37	5.82E-37	0.22	(0.16,0.27)	6.10E-12	1.68E-11
365	PC(P-16:0/16:1)	-0.37	(-0.42,-0.32)	9.86E-45	1.71E-44	-0.91	(-0.96,-0.85)	2.01E-114	2.62E-114	0.03	(-0.05,0.1)	4.58E-01	4.63E-01	0.50	(0.43,0.57)	4.79E-36	1.92E-34
366	PC(P-16:0/18:0)	-0.02	(-0.06,0.01)	1.59E-01	1.68E-01	-0.89	(-0.94,-0.85)	5.20E-154	7.83E-154	0.77	(0.71,0.82)	1.44E-73	3.40E-73	0.08	(0.03,0.12)	1.75E-03	2.30E-03
367	PC(P-16:0/18:1)	-0.01	(-0.05,0.03)	7.15E-01	7.26E-01	-0.98	(-1.03,-0.93)	1.89E-144	2.71E-144	0.67	(0.61,0.74)	6.87E-56	1.26E-55	0.26	(0.2,0.33)	1.37E-15	5.25E-15
368	PC(P-16:0/18:2)	-0.11	(-0.15,-0.06)	2.72E-05	3.20E-05	-3.54	(-3.59,-3.49)	0.00E+00	0.00E+00	3.28	(3.2,3.35)	6.74E-181	2.94E-179	0.12	(0.04,0.19)	3.35E-03	4.30E-03
369	PC(P-16:0/18:3)	-0.01	(-0.07,0.04)	6.70E-01	6.86E-01	-3.05	(-3.1,-3)	0.00E+00	0.00E+00	2.82	(2.74,2.89)	4.45E-164	1.02E-162	0.20	(0.12,0.28)	1.11E-06	1.92E-06
370	PC(P-35:2) (a)	-0.22	(-0.3,-0.14)	4.55E-08	5.62E-08	-3.80	(-3.87,-3.72)	9.39E-306	9.39E-305	3.31	(3.21,3.42)	1.18E-147	1.45E-146	0.14	(0.03,0.25)	1.21E-02	1.49E-02
371	PC(P-35:2) (b)	-0.13	(-0.19,-0.07)	6.92E-05	8.12E-05	-4.02	(-4.09,-3.96)	0.00E+00	0.00E+00	3.64	(3.55,3.73)	1.88E-170	5.64E-169	0.22	(0.13,0.31)	2.72E-06	4.55E-06
372	PC(P-15:0/20:4) (b)	0.07	(0,0.13)	3.86E-02	4.20E-02	-1.56	(-1.63,-1.5)	1.03E-173	1.65E-173	1.44	(1.35,1.53)	6.14E-85	1.77E-84	0.15	(0.06,0.24)	8.97E-04	1.22E-03
373	PC(P-18:1/18:1)	-0.07	(-0.11,-0.03)	8.48E-04	9.66E-04	-1.27	(-1.33,-1.21)	9.41E-146	1.36E-145	0.87	(0.79,0.95)	2.91E-55	5.33E-55	0.25	(0.19,0.31)	1.63E-14	5.57E-14
374	PC(P-18:0/18:2)	-0.03	(-0.07,0.02)	2.90E-01	3.00E-01	-3.34	(-3.39,-3.29)	0.00E+00	0.00E+00	3.20	(3.13,3.28)	1.69E-181	8.13E-180	0.03	(-0.04,0.11)	3.80E-01	4.03E-01
375	PC(P-16:0/20:4)	-0.14	(-0.19,-0.09)	3.64E-09	4.56E-09	-1.28	(-1.33,-1.22)	5.77E-177	9.39E-177	0.98	(0.91,1.05)	4.77E-72	1.09E-71	0.07	(0,0.14)	3.61E-02	4.23E-02
376	PC(P-16:0/20:5)	0.17	(0.09,0.26)	9.27E-05	1.09E-04	-3.03	(-3.1,-2.96)	2.74E-285	1.58E-284	2.51	(2.39,2.63)	8.65E-111	3.96E-110	0.63	(0.5,0.75)	1.13E-19	5.41E-19
377	PC(P-17:0/20:4) (a)	-0.16	(-0.23,-0.1)	5.36E-07	6.52E-07	-2.00	(-2.06,-1.94)	4.59E-223	1.10E-222	1.68	(1.58,1.79)	9.79E-84	2.73E-83	0.06	(-0.04,0.17)	2.40E-01	2.59E-01
378	PC(P-17:0/20:4) (b)	0.05	(-0.01,0.11)	8.32E-02	8.89E-02	-1.76	(-1.82,-1.7)	2.76E-205	5.45E-205	1.54	(1.45,1.64)	7.51E-88	2.31E-87	0.18	(0.09,0.27)	9.83E-05	1.43E-04
379	PC(P-18:0/20:4)	0.17	(0.12,0.21)	5.66E-14	7.45E-14	-1.11	(-1.16,-1.06)	1.55E-165	2.41E-165	1.26	(1.18,1.33)	7.68E-93	2.58E-92	-0.05	(-0.12,0.01)	1.13E-01	1.27E-01
380	PC(P-38:5) (a)	-0.08	(-0.12,-0.03)	7.19E-04	8.22E-04	-2.06	(-2.11,-2.01)	3.29E-265	1.33E-264	1.67	(1.6,1.75)	2.68E-118	1.48E-117	0.23	(0.16,0.3)	8.00E-11	1.99E-10
381	PC(P-38:5) (b)	-0.22	(-0.27,-0.17)	1.31E-17	1.79E-17	-1.34	(-1.4,-1.28)	7.22E-167	1.14E-166	1.12	(1.03,1.2)	1.60E-71	3.63E-71	-0.02	(-0.09,0.05)	5.58E-01	5.85E-01
382	PC(P-16:0/22:6)	-0.45	(-0.5,-0.39)	2.39E-44	4.12E-44	-1.91	(-1.96,-1.86)	2.02E-244	6.61E-244	1.14	(1.05,1.22)	7.00E-73	1.63E-72	0.23	(0.16,0.31)	1.20E-08	2.46E-08
383	PC(P-20:0/20:4)	-0.20	(-0.24,-0.15)	7.06E-16	9.41E-16	-1.02	(-1.07,-0.97)	5.48E-145	7.90E-145	0.93	(0.86,1)	3.39E-69	7.29E-69	-0.18	(-0.25,-0.11)	3.68E-07	6.61E-07
384	PC(P-40:5) (b)	-0.05	(-0.09,-0.01)	1.49E-02	1.64E-02	-1.28	(-1.33,-1.24)	1.29E-211	2.65E-211	1.16	(1.09,1.22)	1.16E-97	4.37E-97	0.01	(-0.05,0.07)	7.27E-01	7.44E-01
385	PC(P-18:0/22:6)	-0.01	(-0.06,0.04)	6.95E-01	7.08E-01	-1.81	(-1.86,-1.76)	6.46E-246	2.12E-245	1.69	(1.6,1.77)	1.65E-105	6.75E-105	0.00	(-0.08,0.08)	9.42E-01	9.42E-01
386	PE(16:0_16:1)	-3.13	(-3.26,-3)	9.54E-161	1.70E-159	-3.76	(-3.86,-3.66)	1.33E-253	4.76E-253	0.11	(-0.04,0.27)	1.40E-01	1.44E-01	0.45	(0.31,0.6)	4.26E-09	9.08E-09
387	PE(16:0_18:1)	-2.76	(-2.85,-2.67)	1.40E-199	1.68E-197	-4.35	(-4.42,-4.29)	0.00E+00	0.00E+00	1.26	(1.15,1.36)	5.78E-65	1.16E-64	0.34	(0.23,0.45)	5.35E-09	1.12E-08
388	PE(16:0_18:2)	-2.97	(-3.06,-2.87)	3.88E-201	6.21E-199	-5.30	(-5.37,-5.23)	0.00E+00	0.00E+00	1.89	(1.79,1.99)	1.12E-101	4.37E-101	0.45	(0.33,0.56)	3.61E-13	1.10E-12
389	PE(16:1_18:2)	-2.38	(-2.48,-2.28)	1.45E-164	3.02E-163	-3.24	(-3.32,-3.16)	3.29E-261	1.23E-260	0.57	(0.47,0.67)	1.57E-24	2.11E-24	0.32	(0.21,0.43)	1.02E-08	2.10E-08
390	PE(16:0_18:3) (a)	-2.92	(-3.02,-2.82)	8.51E-189	5.84E-187	-4.60	(-4.68,-4.53)	0.00E+00	0.00E+00	1.51	(1.4,1.63)	1.59E-70	3.53E-70	0.25	(0.13,0.37)	6.25E-05	9.29E-05
391	PE(16:0_18:3) (b)	-1.96	(-2.07,-1.86)	1.57E-126	1.18E-125	-2.97	(-3.06,-2.89)	3.13E-244	1.02E-243	0.88	(0.75,1)	2.51E-32	3.69E-32	0.09	(-0.05,0.23)	2.00E-01	2.17E-01
392	PE(17:0_18:2)	-2.10	(-2.18,-2.02)	5.48E-178	2.02E-176	-3.72	(-3.77,-3.66)	0.00E+00	0.00E+00	1.37	(1.28,1.46)	7.14E-83	1.97E-82	0.29	(0.19,0.39)	3.01E-08	5.94E-08
393	PE(18:0_18:1)	-2.12	(-2.2,-2.04)	2.10E-175	6.73E-174	-4.60	(-4.67,-4.54)	0.00E+00	0.00E+00	2.23	(2.13,2.33)	1.61E-114	8.57E-114	0.31	(0.2,0.42)	4.20E-08	8.19E-08
394	PE(18:1_18:1)	-1.89	(-1.97,-1.82)	7.99E-167	1.83E-165	-3.30	(-3.38,-3.22)	5.72E-265	2.29E-264	1.26	(1.17,1.35)	1.79E-76	4.51E-76	0.16	(0.07,0.24)	2.42E-04	3.46E-04
395	PE(18:0_18:2)	-1.91	(-1.99,-1.84)	5.67E-168	1.36E-166	-5.16	(-5.22,-5.1)	0.00E+00	0.00E+00	2.89	(2.79,2.98)	1.04E-146	1.22E-145	0.42	(0.32,0.53)	7.56E-14	2.45E-13
396	PE(18:1_18:2)	-2.53	(-2.62,-2.44)	2.74E-183	1.46E-181	-5.12	(-5.19,-5.06)	0.00E+00	0.00E+00	2.37	(2.27,2.47)	1.20E-117	6.53E-117	0.30	(0.17,0.42)	2.53E-06	4.27E-06
397	PE(16:0_20:3)	-2.68	(-2.77,-2.58)	7.78E-187	4.67E-185	-3.67	(-3.74,-3.6)	6.22E-306	6.35E-305	0.68	(0.58,0.79)	2.07E-30	2.98E-30	0.30	(0.19,0.41)	4.39E-07	7.83E-07
398	PE(16:0_20:4)	-2.51	(-2.59,-2.43)	3.67E-203	8.80E-201	-2.91	(-2.97,-2.85)	8.26E-294	6.20E-293	-0.02	(-0.1,0.07)	7.07E-01	7.08E-01	0.41	(0.31,0.51)	2.77E-15	1.03E-14
399	PE(16:1_20:4)	-1.97	(-2.06,-1.89)	1.59E-158	2.73E-157	-1.90	(-1.97,-1.82)	8.06E-179	1.32E-178	-0.46	(-0.55,-0.37)	7.64E-19	9.80E-19	0.34	(0.25,0.42)	3.21E-13	9.89E-13
400	PE(16:0_20:5)	-1.95	(-2.04,-1.85)	4.44E-140	4.54E-139	-3.29	(-3.36,-3.21)	1.57E-273	7.25E-273	0.64	(0.52,0.75)	1.29E-22	1.71E-22	0.73	(0.59,0.86)	1.12E-22	6.92E-22
401	PE(17:0_20:4)	-1.82	(-1.89,-1.75)	6.12E-178	2.10E-176	-2.75	(-2.81,-2.69)	6.43E-291	4.17E-290	0.55	(0.46,0.65)	3.15E-24	4.21E-24	0.33	(0.24,0.43)	8.73E-11	2.15E-10
402	PE(18:0_20:3) (a)	-1.87	(-1.96,-1.79)	4.02E-152	5.52E-151	-2.50	(-2.57,-2.43)	1.49E-243	4.71E-243	0.25	(0.16,0.35)	5.53E-07	6.14E-07	0.35	(0.24,0.46)	3.38E-10	7.99E-10
403	PE(18:0_20:4)	-1.46	(-1.52,-1.4)	4.07E-163	8.15E-162	-2.30	(-2.36,-2.24)	7.46E-265	2.96E-264	0.42	(0.33,0.5)	1.71E-19	2.21E-19	0.47	(0.38,0.55)	3.06E-23	2.07E-22

404	PE(38:5) (a)	-2.34	(-2.41,-2.26)	8.54E-194	6.83E-192	-3.31	(-3.37,-3.25)	0.00E+00	0.00E+00	0.55	(0.45,0.64)	1.73E-24	2.32E-24	0.45	(0.35,0.55)	1.47E-16	6.07E-16
405	PE(38:5) (b)	-2.23	(-2.32,-2.14)	7.15E-169	1.81E-167	-3.76	(-3.84,-3.68)	2.82E-291	1.88E-290	1.10	(0.97,1.23)	8.24E-41	1.30E-40	0.44	(0.32,0.57)	9.51E-12	2.52E-11
406	PE(16:0_22:6)	-2.64	(-2.73,-2.55)	1.49E-195	1.43E-193	-3.80	(-3.86,-3.73)	0.00E+00	0.00E+00	0.31	(0.2,0.41)	6.36E-08	7.20E-08	0.82	(0.71,0.94)	1.26E-33	3.17E-32
407	PE(17:0_22:6)	-1.91	(-1.98,-1.83)	4.06E-173	1.22E-171	-2.79	(-2.86,-2.73)	1.99E-272	8.91E-272	0.36	(0.26,0.46)	4.75E-11	5.62E-11	0.59	(0.48,0.69)	5.19E-24	3.72E-23
408	PE(18:0_22:4)	-1.77	(-1.85,-1.69)	2.80E-147	3.44E-146	-2.46	(-2.53,-2.38)	2.09E-223	5.01E-223	0.61	(0.51,0.71)	4.81E-26	6.54E-26	0.09	(0,0.18)	4.11E-02	4.78E-02
409	PE(18:0_22:5) (n3)	-1.80	(-1.87,-1.73)	5.09E-165	1.11E-163	-3.48	(-3.55,-3.4)	9.37E-294	6.92E-293	1.18	(1.06,1.29)	4.13E-53	7.45E-53	0.57	(0.47,0.67)	9.59E-24	6.67E-23
410	PE(18:0_22:5) (n6)	-1.88	(-1.97,-1.79)	3.23E-148	4.19E-147	-3.06	(-3.14,-2.97)	5.50E-250	1.87E-249	0.95	(0.82,1.08)	4.18E-33	6.21E-33	0.23	(0.12,0.35)	5.22E-05	7.83E-05
411	PE(18:0_22:6)	-2.04	(-2.12,-1.96)	4.85E-172	1.37E-170	-3.61	(-3.68,-3.53)	2.56E-296	2.12E-295	0.67	(0.55,0.79)	2.30E-24	3.08E-24	0.90	(0.79,1.02)	8.68E-39	4.63E-37
412	PE(40:7)	-3.12	(-3.22,-3.03)	7.29E-208	3.50E-205	-4.30	(-4.38,-4.23)	0.00E+00	0.00E+00	0.40	(0.29,0.52)	8.48E-11	9.98E-11	0.79	(0.66,0.92)	2.34E-27	2.34E-26
413	PE(O-34:1)	-0.07	(-0.13,-0.01)	1.51E-02	1.66E-02	-3.50	(-3.58,-3.42)	5.78E-275	2.74E-274	3.56	(3.45,3.67)	2.26E-151	3.19E-150	-0.13	(-0.21,-0.04)	3.08E-03	3.97E-03
414	PE(O-16:0/18:2)	-0.14	(-0.21,-0.06)	5.15E-04	5.89E-04	-5.84	(-5.97,-5.7)	1.91E-276	9.45E-276	5.56	(5.39,5.73)	7.94E-150	1.09E-148	0.14	(0.03,0.25)	1.26E-02	1.54E-02
415	PE(O-18:1/18:2)	0.10	(0.03,0.16)	4.97E-03	5.56E-03	-2.91	(-2.99,-2.83)	2.74E-242	8.56E-242	2.89	(2.79,3)	4.28E-134	3.95E-133	0.06	(-0.03,0.16)	1.76E-01	1.94E-01
416	PE(O-16:0/20:4)	0.14	(0.07,0.21)	1.67E-04	1.92E-04	-3.05	(-3.13,-2.97)	1.36E-262	5.18E-262	2.99	(2.89,3.1)	7.52E-137	7.36E-136	0.10	(0,0.2)	4.61E-02	5.32E-02
417	PE(O-36:5)	-0.25	(-0.35,-0.14)	3.88E-06	4.65E-06	-3.35	(-3.44,-3.25)	3.52E-237	1.00E-236	2.47	(2.35,2.59)	1.47E-108	6.37E-108	0.63	(0.49,0.77)	8.35E-17	3.51E-16
418	PE(O-16:0/22:4)	-0.11	(-0.17,-0.05)	1.28E-04	1.48E-04	-1.25	(-1.3,-1.2)	1.57E-168	2.48E-168	1.18	(1.1,1.27)	2.67E-77	6.81E-77	-0.02	(-0.1,0.06)	5.68E-01	5.91E-01
419	PE(O-18:0/20:4)	0.22	(0.14,0.3)	7.50E-08	9.20E-08	-2.77	(-2.85,-2.69)	1.46E-233	4.02E-233	2.79	(2.67,2.9)	1.38E-121	8.06E-121	0.10	(-0.02,0.21)	9.90E-02	1.12E-01
420	PE(O-38:5) (a)	0.17	(0.11,0.24)	1.15E-06	1.39E-06	-3.66	(-3.73,-3.59)	0.00E+00	0.00E+00	3.64	(3.54,3.74)	9.74E-163	1.95E-161	0.11	(0,0.21)	4.46E-02	5.18E-02
421	PE(O-38:5) (b)	-0.06	(-0.13,0.01)	1.12E-01	1.19E-01	-2.26	(-2.34,-2.18)	6.76E-208	1.36E-207	1.99	(1.88,2.1)	5.22E-97	1.93E-96	0.14	(0.04,0.24)	4.61E-03	5.82E-03
422	PE(O-16:0/22:6)	-0.21	(-0.27,-0.15)	6.06E-12	7.65E-12	-3.01	(-3.07,-2.95)	5.37E-297	4.69E-296	2.46	(2.38,2.55)	2.49E-140	2.54E-139	0.25	(0.17,0.33)	3.18E-09	6.80E-09
423	PE(O-18:0/22:6)	0.09	(0.03,0.15)	2.53E-03	2.85E-03	-2.31	(-2.37,-2.25)	3.69E-257	1.34E-256	2.20	(2.12,2.29)	2.32E-130	1.99E-129	0.13	(0.04,0.21)	3.36E-03	4.30E-03
424	PE(O-18:1/22:6)	-0.09	(-0.15,-0.04)	1.41E-03	1.60E-03	-2.59	(-2.64,-2.53)	1.40E-293	1.02E-292	2.26	(2.17,2.35)	6.74E-130	5.58E-129	0.18	(0.11,0.26)	4.89E-06	8.06E-06
425	PE(P-16:0/18:1)	-0.04	(-0.09,0)	7.63E-02	8.19E-02	-1.37	(-1.42,-1.31)	4.16E-192	7.33E-192	1.65	(1.58,1.72)	3.05E-122	1.81E-121	-0.37	(-0.44,-0.31)	2.82E-23	1.93E-22
426	PE(P-16:0/18:2)	-0.10	(-0.17,-0.04)	1.95E-03	2.20E-03	-4.72	(-4.79,-4.65)	0.00E+00	0.00E+00	4.76	(4.67,4.86)	6.49E-196	1.04E-193	-0.24	(-0.34,-0.15)	7.07E-07	1.23E-06
427	PE(P-16:0/20:3)	-0.02	(-0.09,0.05)	5.72E-01	5.88E-01	-2.10	(-2.17,-2.02)	5.08E-208	1.02E-207	2.32	(2.23,2.41)	1.50E-128	1.16E-127	-0.37	(-0.45,-0.28)	8.18E-15	2.89E-14
428	PE(P-16:0/20:4)	0.03	(-0.03,0.09)	3.59E-01	3.71E-01	-2.86	(-2.93,-2.79)	8.17E-277	4.13E-276	2.92	(2.82,3.01)	7.70E-144	8.80E-143	-0.12	(-0.21,-0.03)	1.05E-02	1.29E-02
429	PE(P-16:0/20:5)	-0.02	(-0.13,0.09)	6.96E-01	7.08E-01	-6.11	(-6.25,-5.97)	1.23E-275	5.91E-275	5.48	(5.27,5.69)	9.08E-130	7.26E-129	0.48	(0.32,0.63)	5.04E-09	1.06E-08
430	PE(P-16:0/22:4)	-0.22	(-0.26,-0.17)	3.17E-16	4.26E-16	-0.04	(-0.09,0.01)	1.12E-01	1.14E-01	0.34	(0.27,0.42)	3.21E-18	4.10E-18	-0.59	(-0.65,-0.52)	2.95E-47	3.54E-45
431	PE(P-16:0/22:5) (n3)	-0.05	(-0.1,0.01)	7.73E-02	8.28E-02	-2.97	(-3.02,-2.91)	0.00E+00	0.00E+00	2.97	(2.89,3.06)	1.50E-159	2.87E-158	-0.09	(-0.17,-0.01)	2.13E-02	2.55E-02
432	PE(P-16:0/22:5) (n6)	-0.35	(-0.41,-0.29)	2.75E-27	4.15E-27	-1.29	(-1.35,-1.23)	1.50E-168	2.38E-168	1.33	(1.23,1.42)	8.39E-75	2.02E-74	-0.42	(-0.5,-0.33)	3.36E-20	1.66E-19
433	PE(P-16:0/22:6)	-0.41	(-0.46,-0.36)	6.84E-47	1.22E-46	-2.25	(-2.29,-2.2)	2.24E-287	1.33E-286	1.63	(1.55,1.7)	7.02E-111	3.24E-110	0.15	(0.09,0.22)	1.08E-05	1.70E-05
434	PE(P-17:0/20:4) (a)	0.14	(0.06,0.22)	1.06E-03	1.20E-03	-2.13	(-2.2,-2.06)	9.14E-212	1.88E-211	2.02	(1.92,2.12)	5.44E-104	2.20E-103	0.18	(0.07,0.29)	1.57E-03	2.07E-03
435	PE(P-17:0/20:4) (b)	0.16	(0.09,0.24)	1.11E-05	1.31E-05	-2.38	(-2.45,-2.31)	1.64E-227	4.18E-227	2.29	(2.19,2.39)	2.82E-114	1.49E-113	0.23	(0.13,0.33)	1.44E-05	2.22E-05
436	PE(P-17:0/22:6) (a)	-0.19	(-0.26,-0.12)	2.89E-07	3.53E-07	-1.94	(-2,-1.87)	8.50E-205	1.67E-204	1.46	(1.35,1.57)	3.16E-72	7.28E-72	0.28	(0.17,0.38)	5.36E-07	9.50E-07
437	PE(P-17:0/22:6) (b)	-0.15	(-0.22,-0.09)	6.64E-06	7.93E-06	-2.44	(-2.5,-2.38)	9.12E-267	3.77E-266	1.98	(1.88,2.08)	1.82E-106	7.61E-106	0.30	(0.21,0.4)	5.55E-10	1.26E-09
438	PE(P-18:0/18:1)	0.00	(-0.05,0.06)	9.11E-01	9.11E-01	-2.99	(-3.05,-2.93)	6.93E-301	6.52E-300	3.27	(3.19,3.34)	5.12E-183	2.73E-181	-0.35	(-0.43,-0.27)	2.44E-16	9.84E-16
439	PE(P-18:0/18:2)	-0.05	(-0.11,0.02)	1.44E-01	1.53E-01	-5.95	(-6.03,-5.88)	0.00E+00	0.00E+00	6.08	(5.98,6.18)	8.68E-217	4.17E-214	-0.22	(-0.31,-0.13)	3.82E-06	6.34E-06
440	PE(P-18:0/20:3)	0.00	(-0.06,0.06)	9.05E-01	9.06E-01	-2.32	(-2.38,-2.26)	1.55E-262	5.85E-262	2.55	(2.47,2.62)	2.66E-156	4.41E-155	-0.29	(-0.37,-0.22)	3.40E-13	1.04E-12
441	PE(P-18:0/20:4)	0.33	(0.27,0.39)	1.13E-23	1.63E-23	-3.00	(-3.07,-2.93)	3.70E-278	1.91E-277	3.28	(3.18,3.37)	7.52E-157	1.29E-155	-0.05	(-0.14,0.04)	3.17E-01	3.40E-01
442	PE(P-18:0/20:5)	0.15	(0.03,0.27)	1.11E-02	1.23E-02	-6.17	(-6.3,-6.03)	8.16E-284	4.61E-283	5.80	(5.6,6)	8.73E-141	9.11E-140	0.49	(0.34,0.65)	1.42E-09	3.12E-09
443	PE(P-18:0/22:4)	-0.06	(-0.11,0)	4.72E-02	5.13E-02	-0.16	(-0.22,-0.1)	6.56E-08	6.91E-08	0.84	(0.76,0.91)	2.28E-57	4.25E-57	-0.79	(-0.86,-0.72)	8.23E-62	3.95E-59
444	PE(P-18:0/22:5) (n3)	0.23	(0.18,0.29)	5.18E-16	6.93E-16	-2.45	(-2.5,-2.39)	9.29E-297	7.96E-296	2.79	(2.71,2.86)	3.12E-168	8.82E-167	-0.14	(-0.21,-0.06)	3.85E-04	5.40E-04
445	PE(P-18:0/22:5) (n6)	-0.11	(-0.17,-0.06)	1.27E-04	1.48E-04	-1.47	(-1.53,-1.41)	2.86E-190	4.99E-190	1.72	(1.63,1.82)	4.01E-96	1.42E-95	-0.39	(-0.48,-0.3)	1.86E-16	7.55E-16
446	PE(P-18:0/22:6)	0.03	(-0.02,0.08)	2.44E-01	2.55E-01	-2.55	(-2.6,-2.5)	6.87E-296	5.59E-295	2.51	(2.43,2.6)	9.32E-143	1.02E-141	-0.01	(-0.09,0.06)	7.13E-01	7.32E-01
447	PE(P-18:1/18:1)	-0.13	(-0.18,-0.08)	5.20E-07	6.33E-07	-2.42	(-2.47,-2.37)	4.07E-290	2.61E-289	2.65	(2.58,2.72)	8.11E-166	1.95E-164	-0.40	(-0.47,-0.32)	7.29E-23	4.60E-22
448	PE(P-18:1/18:2)	-0.17	(-0.23,-0.1)	7.14E-07	8.65E-07	-5.96	(-6.05,-5.87)	0.00E+00	0.00E+00	5.97	(5.84,6.09)	5.17E-189	4.13E-187	-0.25	(-0.35,-0.15)	1.04E-06	1.80E-06
449	PE(P-18:1/20:4)	0.16	(0.09,0.22)	1.17E-06	1.41E-06	-3.19	(-3.26,-3.12)	2.97E-289	1.83E-288	3.41	(3.32,3.51)	5.02E-158	9.27E-157	-0.14	(-0.23,-0.05)	3.38E-03	4.31E-03
450	PE(P-18:1/20:5)	-0.25	(-0.36,-0.14)	1.19E-05	1.41E-05	-6.96	(-7.13,-6.79)	2.99E-268	1.26E-267	6.18	(5.94,6.43)	5.22E-128	3.74E-127	0.43	(0.27,0.59)	2.16E-07	3.95E-07
451	PE(P-18:1/22:4)	-0.18	(-0.24,-0.12)	1.14E-08	1.41E-08	-0.23	(-0.3,-0.17)	6.11E-12	6.48E-12	0.70	(0.62,0.78)	1.94E-44	3.17E-44	-0.72	(-0.8,-0.65)	5.75E-53	9.19E-51
452	PE(P-18:1/22:5) (a)	0.08	(0.02,0.13)	5.28E-03	5.90E-03	-2.50	(-2.56,-2.45)	9.15E-292	6.46E-291	2.65	(2.57,2.73)	2.58E-152	3.86E-151	-0.07	(-0.15,0.01)	8.30E-02	9.42E-02
453	PE(P-18:1/22:6) (a)	-0.34	(-0.4,-0.29)	5.67E-30	8.75E-30	-3.02	(-3.07,-2.96)	0.00E+00	0.00E+00	2.55	(2.46,2.64)	2.08E-138	2.08E-137	0.06	(-0.02,0.14)	1.24E-01	1.38E-01
454	PE(P-20:0/18:1)	-0.10	(-0.15,-0.04)	1.17E-03	1.33E-03	-3.52	(-3.63,-3.4)	6.92E-212	1.43E-211	4.04	(3.89,4.2)	2.25E-128	1.69E-127	-0.65	(-0.73,-0.57)	6.49E-40	4.45E-38

455	PE(P-20:0/18:2)	-0.03	(-0.09,0.02)	1.89E-01	1.99E-01	-3.32	(-3.37,-3.27)	**0.00E+00**	**0.00E+00**	3.81	(3.75,3.88)	**1.39E-210**	**3.35E-208**	-0.56	(-0.64,-0.49)	**1.20E-34**	**3.40E-33**
456	PE(P-20:0/20:4)	0.42	(0.37,0.47)	**2.15E-47**	**3.85E-47**	-1.58	(-1.65,-1.51)	**4.76E-174**	**7.67E-174**	2.41	(2.32,2.49)	**4.61E-135**	**4.34E-134**	-0.48	(-0.55,-0.41)	**1.58E-30**	**2.30E-29**
457	PE(P-20:0/22:6)	-0.11	(-0.15,-0.06)	**2.84E-06**	**3.42E-06**	-1.92	(-1.97,-1.88)	**1.89E-273**	**8.64E-273**	1.96	(1.89,2.04)	**1.40E-129**	**1.10E-128**	-0.22	(-0.29,-0.16)	**1.93E-10**	**4.64E-10**
458	PE(P-20:1/20:4)	0.36	(0.27,0.44)	**2.73E-15**	**3.63E-15**	-2.81	(-2.9,-2.72)	**1.74E-218**	**3.96E-218**	3.02	(2.89,3.15)	**2.77E-119**	**1.58E-118**	0.08	(-0.04,0.2)	1.87E-01	2.05E-01
459	PG(34:2)	-1.24	(-1.33,-1.15)	**9.59E-91**	**3.44E-90**	-2.54	(-2.64,-2.44)	**1.70E-188**	**2.96E-188**	0.95	(0.79,1.11)	**7.98E-26**	**1.08E-25**	0.24	(0.13,0.36)	**5.65E-05**	**8.45E-05**
460	PG(36:1)	-0.89	(-0.98,-0.81)	**3.85E-69**	**9.07E-69**	-2.47	(-2.56,-2.38)	**4.18E-193**	**7.48E-193**	1.35	(1.2,1.5)	**3.14E-45**	**5.17E-45**	0.36	(0.24,0.48)	**6.49E-09**	**1.35E-08**
461	PI(16:0/16:0)	-3.06	(-3.17,-2.95)	**2.26E-178**	**9.04E-177**	-4.54	(-4.63,-4.45)	**1.75E-302**	**1.72E-301**	1.18	(1.06,1.3)	**1.35E-51**	**2.39E-51**	0.27	(0.15,0.39)	**1.33E-05**	**2.07E-05**
462	PI(16:0_16:1)	-1.93	(-2.05,-1.82)	**1.48E-110**	**8.35E-110**	-2.60	(-2.69,-2.51)	**3.80E-204**	**7.35E-204**	0.54	(0.43,0.66)	**5.15E-17**	**6.44E-17**	0.12	(-0.01,0.25)	6.18E-02	7.04E-02
463	PI(34:0)	-3.14	(-3.25,-3.02)	**8.72E-183**	**4.19E-181**	-5.20	(-5.31,-5.09)	**3.49E-294**	**2.66E-293**	1.64	(1.48,1.8)	**1.21E-54**	**2.20E-54**	0.46	(0.33,0.59)	**8.51E-12**	**2.28E-11**
464	PI(34:1)	-1.65	(-1.73,-1.58)	**4.07E-154**	**5.75E-153**	-4.29	(-4.35,-4.23)	**0.00E+00**	**0.00E+00**	2.58	(2.5,2.66)	**2.55E-148**	**3.23E-147**	0.08	(0,0.17)	5.67E-02	6.50E-02
465	PI(17:0_18:1)	-1.46	(-1.53,-1.39)	**4.85E-144**	**5.54E-143**	-4.11	(-4.18,-4.03)	**0.00E+00**	**0.00E+00**	2.63	(2.52,2.74)	**4.63E-123**	**2.92E-122**	0.10	(0.01,0.19)	**2.31E-02**	**2.75E-02**
466	PI(17:0_18:2)	-1.45	(-1.52,-1.38)	**3.58E-143**	**4.00E-142**	-4.27	(-4.36,-4.19)	**2.73E-300**	**2.52E-299**	2.65	(2.51,2.79)	**3.96E-101**	**1.53E-100**	0.23	(0.14,0.33)	**1.18E-06**	**2.04E-06**
467	PI(18:0_18:1)	-1.70	(-1.77,-1.62)	**3.29E-157**	**5.26E-156**	-5.98	(-6.04,-5.91)	**0.00E+00**	**0.00E+00**	4.17	(4.08,4.26)	**3.36E-187**	**2.02E-185**	0.16	(0.07,0.25)	**9.65E-04**	**1.30E-03**
468	PI(36:2)	-1.43	(-1.48,-1.37)	**3.39E-171**	**9.03E-170**	-5.15	(-5.21,-5.1)	**0.00E+00**	**0.00E+00**	3.61	(3.54,3.68)	**2.26E-193**	**2.72E-191**	0.13	(0.06,0.2)	**1.70E-04**	**2.45E-04**
469	PI(18:1_18:2)	-1.18	(-1.25,-1.12)	**3.68E-127**	**2.87E-126**	-2.96	(-3.01,-2.9)	**0.00E+00**	**0.00E+00**	1.74	(1.66,1.82)	**1.37E-111**	**6.52E-111**	0.03	(-0.06,0.12)	4.74E-01	5.01E-01
470	PI(16:0_20:3) (a)	-1.43	(-1.5,-1.36)	**1.50E-133**	**1.31E-132**	-1.96	(-2.01,-1.9)	**2.67E-242**	**8.39E-242**	0.70	(0.63,0.78)	**1.61E-49**	**2.80E-49**	-0.19	(-0.27,-0.11)	**8.10E-06**	**1.30E-05**
471	PI(16:0_20:3) (b)	-1.09	(-1.17,-1.01)	**7.08E-89**	**2.31E-88**	-0.75	(-0.84,-0.66)	**4.59E-50**	**5.27E-50**	-0.09	(-0.19,0.01)	7.36E-02	7.60E-02	-0.18	(-0.27,-0.09)	**6.33E-05**	**9.37E-05**
472	PI(16:0_20:4)	-1.06	(-1.12,-0.99)	**2.63E-110**	**1.47E-109**	-1.09	(-1.15,-1.04)	**5.57E-152**	**8.27E-152**	0.03	(-0.05,0.1)	5.05E-01	5.09E-01	-0.02	(-0.09,0.06)	6.35E-01	6.54E-01
473	PI(18:0_20:2)	-1.67	(-1.74,-1.61)	**1.81E-161**	**3.35E-160**	-3.33	(-3.42,-3.25)	**1.13E-261**	**4.23E-261**	1.53	(1.42,1.63)	**3.18E-79**	**8.26E-79**	0.14	(0.05,0.22)	**1.44E-03**	**1.90E-03**
474	PI(18:0_20:3) (a)	-0.52	(-0.56,-0.47)	**3.13E-75**	**8.17E-75**	-1.46	(-1.5,-1.42)	**7.88E-228**	**2.03E-227**	0.79	(0.73,0.85)	**1.81E-69**	**3.91E-69**	0.13	(0.07,0.19)	**4.46E-05**	**6.71E-05**
475	PI(18:0_20:4)	-0.48	(-0.53,-0.44)	**1.32E-69**	**3.12E-69**	-1.41	(-1.46,-1.37)	**5.39E-223**	**1.28E-222**	0.77	(0.7,0.83)	**4.73E-64**	**9.39E-64**	0.15	(0.09,0.21)	**1.86E-06**	**3.16E-06**
476	PI(38:5) (a)	-0.86	(-0.92,-0.8)	**2.64E-99**	**1.11E-98**	-0.66	(-0.72,-0.61)	**1.44E-77**	**1.70E-77**	-0.22	(-0.3,-0.14)	**2.67E-07**	**3.00E-07**	0.02	(-0.05,0.09)	5.65E-01	5.90E-01
477	PI(38:6)	-1.36	(-1.42,-1.29)	**4.66E-146**	**5.59E-145**	-1.79	(-1.84,-1.73)	**5.06E-235**	**1.41E-234**	0.47	(0.39,0.54)	**7.90E-29**	**1.12E-28**	-0.10	(-0.18,-0.02)	**1.04E-02**	**1.29E-02**
478	PI(18:0_22:4)	-1.24	(-1.3,-1.18)	**1.08E-133**	**9.62E-133**	-1.83	(-1.89,-1.77)	**6.13E-214**	**1.33E-213**	0.59	(0.5,0.67)	**1.12E-31**	**1.63E-31**	-0.02	(-0.09,0.05)	5.92E-01	6.15E-01
479	PI(18:0_22:5) (n3)	-0.49	(-0.55,-0.43)	**6.84E-42**	**1.15E-41**	-2.63	(-2.69,-2.57)	**1.69E-283**	**9.45E-283**	1.86	(1.77,1.95)	**3.94E-111**	**1.84E-110**	0.26	(0.18,0.34)	**4.18E-10**	**9.64E-10**
480	PI(18:0_22:6)	-1.31	(-1.37,-1.25)	**1.88E-147**	**2.37E-146**	-2.50	(-2.55,-2.45)	**0.00E+00**	**0.00E+00**	1.18	(1.1,1.25)	**1.45E-82**	**3.98E-82**	0.02	(-0.05,0.1)	5.51E-01	5.79E-01

Table S2A. Association of lipid classes with BMI or BW in four sample groups (antenatal, postnatal, cord blood and 6-year-old child) in terms of levels. Related to Figure 2.

	**ppBMI (antenatal)** adjusted for ethnicity, maternal age, maternal education level and gestational weight gain at 26-28 weeks of gestation				**Adult BMI (postnatal)** adjusted for ethnicity, maternal age and maternal education level				**Birth Weight (cord blood)** adjusted for sex, ethnicity, maternal age, maternal education level, pre-pregnancy BMI, total gestational weight gain, gestational age and parity				**Child BMI (6-year-old)** adjusted for sex, ethnicity and maternal education level			
Lipid Class	%change in lipid concentration per unit BMI	95%CI	p-value	p-value(BH)	%change in lipid concentration per unit BMI	95%CI	p-value	p-value(BH)	%change in lipid concentration per 100 grams	95%CI	p-value	p-value(BH)	%change in lipid concentration per unit BMI	95%CI	p-value	p-value(BH)
AC	0.81	(0.18-1.44)	**1.13E-02**	**1.76E-02**	-0.24	(-0.72-0.25)	3.38E-01	3.84E-01	-0.62	(-1.14--0.06)	**3.00E-02**	6.26E-02	-1.00	(-2.51-0.54)	2.00E-01	3.84E-01
CE	-0.93	(-1.26--0.6)	**4.94E-08**	**2.29E-07**	-0.43	(-0.71--0.14)	**3.26E-03**	**4.80E-03**	-0.30	(-0.74-0.17)	2.06E-01	2.86E-01	0.54	(-0.38-1.47)	2.50E-01	4.47E-01
DG	1.12	(0.48-1.76)	**5.79E-04**	**1.45E-03**	3.05	(2.26-3.85)	**8.01E-14**	**1.00E-12**	-2.21	(-2.77--1.61)	**9.98E-11**	**4.99E-10**	2.83	(0.69-5.03)	**9.66E-03**	6.04E-02
TG	0.59	(0.15-1.04)	**8.50E-03**	**1.63E-02**	2.95	(2.24-3.66)	**8.03E-16**	**2.01E-14**	-3.26	(-3.92--2.53)	**2.12E-13**	**1.76E-12**	3.62	(1.61-5.67)	**4.09E-04**	**1.02E-02**
TG(O)	0.44	(-0.14-1.02)	1.35E-01	1.53E-01	2.88	(2.09-3.67)	**1.48E-12**	**1.24E-11**	-2.74	(-3.37--2.05)	**9.36E-12**	**5.85E-11**	3.05	(0.82-5.32)	**7.22E-03**	6.01E-02
dhCer	0.99	(0.41- 1.57)	**7.81E-04**	**1.63E-03**	1.42	(0.85- 1.99)	**9.62E-07**	**2.67E-06**	-0.32	(-0.95- 0.35)	3.35E-01	4.22E-01	0.80	(-0.77- 2.39)	3.18E-01	5.29E-01
Cer	-0.56	(-0.99--0.13)	**1.10E-02**	**1.76E-02**	0.22	(-0.22- 0.66)	3.19E-01	3.80E-01	-0.41	(-0.95- 0.16)	1.54E-01	2.57E-01	0.41	(-0.83- 1.68)	5.16E-01	6.79E-01
DeoxyCer	0.31	(-0.43-1.05)	4.14E-01	4.50E-01	2.50	(1.79-3.21)	**5.39E-12**	**3.37E-11**	-2.12	(-2.66--1.54)	**1.22E-10**	**5.10E-10**	1.65	(-0.04-3.36)	5.57E-02	1.76E-01
GM3	-1.98	(-2.5--1.47)	**1.59E-13**	**1.98E-12**	-0.89	(-1.36--0.42)	**2.46E-04**	**4.38E-04**	-0.34	(-1.01-0.37)	3.37E-01	4.22E-01	-1.46	(-2.71--0.19)	**2.41E-02**	1.20E-01
HexCer	-1.86	(-2.32--1.4)	**1.61E-14**	**4.03E-13**	-0.90	(-1.35--0.45)	**9.66E-05**	**2.01E-04**	0.19	(-0.53-0.97)	6.16E-01	7.33E-01	-1.81	(-3.09--0.5)	**6.98E-03**	6.01E-02
Hex2Cer	-1.53	(-2.04--1.02)	**6.86E-09**	**4.29E-08**	-1.16	(-1.67--0.64)	**1.49E-05**	**3.72E-05**	-0.46	(-1.12-0.25)	1.95E-01	2.86E-01	-1.46	(-3.09-0.21)	8.61E-02	2.39E-01
Hex3Cer	-1.17	(-1.58--0.75)	**5.51E-08**	**2.29E-07**	-1.34	(-1.74--0.94)	**1.52E-10**	**7.60E-10**	-0.44	(-1.02-0.19)	1.65E-01	2.57E-01	-1.20	(-2.32--0.07)	**3.69E-02**	1.54E-01
SM	-1.04	(-1.49--0.58)	**1.18E-05**	**3.70E-05**	-0.32	(-0.75-0.13)	1.60E-01	2.00E-01	-0.10	(-0.63-0.46)	7.17E-01	7.79E-01	-0.58	(-1.78-0.65)	3.53E-01	5.51E-01
LPC	0.07	(-0.4-0.55)	7.62E-01	7.62E-01	-0.91	(-1.38--0.45)	**1.28E-04**	**2.45E-04**	2.25	(1.73-2.79)	**8.80E-19**	**1.10E-17**	0.24	(-1.48-1.98)	7.88E-01	8.56E-01
LPC(O)	-0.87	(-1.26--0.47)	**1.96E-05**	**5.44E-05**	-1.15	(-1.56--0.74)	**6.12E-08**	**2.55E-07**	1.31	(0.86-1.77)	**2.73E-09**	**8.53E-09**	-0.45	(-2.04-1.17)	5.83E-01	6.94E-01
LPC(P)	0.40	(-0.04-0.83)	7.32E-02	9.64E-02	-0.92	(-1.36--0.48)	**4.97E-05**	**1.13E-04**	1.47	(1-1.96)	**1.85E-10**	**6.62E-10**	-0.14	(-1.84-1.59)	8.71E-01	8.77E-01
LPE	-0.37	(-0.81-0.07)	1.01E-01	1.20E-01	-0.79	(-1.28--0.31)	**1.45E-03**	**2.26E-03**	2.55	(1.99-3.13)	**4.07E-21**	**1.02E-19**	-1.18	(-2.61-0.27)	1.09E-01	2.74E-01
PC	-0.62	(-0.98--0.26)	**7.21E-04**	**1.63E-03**	-0.08	(-0.49-0.33)	7.04E-01	7.33E-01	-0.53	(-1.03-0)	5.04E-02	9.01E-02	-0.34	(-1.52-0.85)	5.70E-01	6.94E-01
PC(O)	-0.61	(-1.09--0.14)	**1.13E-02**	**1.76E-02**	-0.71	(-1.18--0.23)	**3.52E-03**	**4.88E-03**	-0.11	(-0.68-0.49)	7.15E-01	7.79E-01	-0.23	(-1.57-1.12)	7.36E-01	8.36E-01
PC(P)	-0.49	(-1.03-0.05)	7.27E-02	9.64E-02	-1.25	(-1.74--0.76)	**6.73E-07**	**2.10E-06**	-0.92	(-1.45--0.35)	**1.83E-03**	**5.07E-03**	-0.61	(-2.02-0.81)	3.96E-01	5.82E-01
PE	0.15	(-0.5-0.8)	6.49E-01	6.76E-01	0.30	(-0.51-1.12)	4.64E-01	5.04E-01	-0.91	(-1.47--0.31)	**3.63E-03**	**9.09E-03**	0.18	(-2.07-2.48)	8.77E-01	8.77E-01
PE(O)	-1.47	(-2.09--0.85)	**4.80E-06**	**1.72E-05**	-1.22	(-1.87--0.57)	**2.76E-04**	**4.60E-04**	-0.06	(-0.65-0.58)	8.60E-01	8.96E-01	0.69	(-1.14-2.56)	4.60E-01	6.40E-01
PE(P)	-0.54	(-1.11-0.05)	7.07E-02	9.64E-02	-0.54	(-1.11-0.03)	6.24E-02	8.21E-02	-0.04	(-0.63-0.59)	9.00E-01	9.00E-01	1.24	(-0.35-2.85)	1.28E-01	2.90E-01
PG	-0.51	(-1.09-0.07)	8.44E-02	1.06E-01	2.08	(1.27-2.89)	**4.82E-07**	**1.72E-06**	-1.00	(-1.87--0.04)	**4.14E-02**	7.96E-02	2.23	(-0.06-4.58)	5.64E-02	1.76E-01
PI	-1.59	(-2.07--1.1)	**2.95E-10**	**2.46E-09**	-0.07	(-0.56-0.42)	7.73E-01	7.73E-01	-0.47	(-0.87--0.05)	**2.97E-02**	6.26E-02	-0.85	(-2.1-0.41)	1.85E-01	3.84E-01

Table S2B. Association of lipid species with BMI or BW in four sample groups (antenatal, postnatal, cord blood and 6-year-old child) in terms of levels. Related to Figure 2.

Studies		**ppBMI (antenatal)**				**Adult BMI (postnatal)**				**Birth Weight (cord blood)**				**Child BMI (6-year-old)**			
Covariates		adjusted for ethnicity, maternal age, maternal education level and gestational weight gain at 26-28 weeks of gestation				adjusted for ethnicity, maternal age and maternal education level				adjusted for sex, ethnicity, maternal age, maternal education level, pre-pregnancy BMI, total gestational weight gain, gestational age and parity				adjusted for sex, ethnicity and maternal education level			
No.	Lipid Species	%change in lipid concentration per unit BMI	95%CI	p-value	p-value(BH)	%change in lipid concentration per unit BMI	95%CI	p-value	p-value(BH)	%change in lipid concentration per 100 grams	95%CI	p-value	p-value(BH)	%change in lipid concentration per unit BMI	95%CI	p-value	p-value(BH)
1	AC(12:0)	0.31	(-0.75-1.38)	5.65E-01	6.18E-01	-0.66	(-1.46-0.15)	1.10E-01	1.47E-01	-1.55	(-2.14--0.93)	**4.02E-06**	**1.32E-05**	-1.49	(-3.93-1.02)	2.42E-01	3.94E-01
2	AC(13:0)	1.33	(-0.01-2.68)	5.12E-02	7.95E-02	-0.35	(-1.59-0.91)	5.84E-01	6.40E-01	-1.16	(-2.22-0.03)	5.65E-02	9.86E-02	0.87	(-2.62-4.49)	6.28E-01	7.41E-01
3	AC(14:0)	0.33	(-0.37-1.04)	3.52E-01	4.17E-01	0.01	(-0.56-0.59)	9.69E-01	9.73E-01	-1.03	(-1.59--0.43)	**1.07E-03**	**2.58E-03**	-1.31	(-3.15-0.58)	1.72E-01	3.06E-01
4	AC(14:1)	1.38	(0.22-2.56)	**1.98E-02**	**3.50E-02**	-0.11	(-0.96-0.75)	8.03E-01	8.39E-01	-1.08	(-1.73--0.37)	**3.63E-03**	**8.13E-03**	-2.04	(-4.66-0.65)	1.36E-01	2.51E-01
5	AC(14:2)	1.03	(-0.08-2.15)	6.79E-02	1.00E-01	-0.44	(-1.25-0.37)	2.84E-01	3.39E-01	-0.94	(-1.49--0.36)	**1.99E-03**	**4.67E-03**	-1.62	(-4.07-0.9)	2.05E-01	3.43E-01
6	AC(16:0)	0.33	(-0.19-0.85)	2.12E-01	2.71E-01	0.00	(-0.42-0.42)	9.90E-01	9.92E-01	-0.47	(-1-0.09)	9.81E-02	1.58E-01	-0.44	(-1.62-0.76)	4.71E-01	6.14E-01
7	AC(16:1)	1.19	(0.37-2.02)	**4.39E-03**	**9.66E-03**	0.35	(-0.27-0.98)	2.67E-01	3.22E-01	-1.00	(-1.64--0.31)	**5.17E-03**	**1.12E-02**	-0.23	(-2.24-1.82)	8.25E-01	8.94E-01
8	AC(18:0)	-0.08	(-0.6-0.45)	7.74E-01	8.06E-01	-0.68	(-1.13--0.23)	**3.16E-03**	**6.00E-03**	-0.78	(-1.3--0.24)	**5.68E-03**	**1.22E-02**	-1.98	(-3.33--0.62)	**4.60E-03**	**1.97E-02**
9	AC(18:1)	1.36	(0.66-2.06)	**1.34E-04**	**4.24E-04**	-0.12	(-0.62-0.37)	6.29E-01	6.83E-01	0.10	(-0.58-0.83)	7.86E-01	8.50E-01	-0.64	(-2.04-0.79)	3.80E-01	5.29E-01
10	AC(18:2)	0.99	(0.31-1.67)	**4.42E-03**	**9.69E-03**	-0.41	(-0.89-0.08)	9.84E-02	1.33E-01	-0.43	(-1.04-0.23)	1.98E-01	2.86E-01	-0.30	(-1.64-1.06)	6.67E-01	7.58E-01
11	CE(14:0)	-1.93	(-2.48--1.38)	**2.08E-11**	**3.99E-10**	0.21	(-0.36-0.8)	4.69E-01	5.28E-01	-0.50	(-1.02-0.05)	7.17E-02	1.22E-01	2.26	(0.55-3.99)	**9.50E-03**	**3.47E-02**
12	CE(16:1)	-1.06	(-1.82--0.29)	**7.23E-03**	**1.46E-02**	2.17	(1.53-2.82)	**6.89E-11**	**4.60E-10**	0.98	(0.31-1.69)	**3.55E-03**	**8.00E-03**	5.77	(4.04-7.53)	**9.25E-11**	**2.22E-08**
13	CE(18:0)	-1.13	(-1.64--0.61)	**2.13E-05**	**8.04E-05**	-0.05	(-0.57-0.47)	8.47E-01	8.74E-01	0.27	(-0.5-1.1)	5.02E-01	5.92E-01	0.82	(-0.9-2.56)	3.51E-01	5.06E-01
14	CE(18:1)	-0.92	(-1.23--0.62)	**7.33E-09**	**5.29E-08**	0.06	(-0.22-0.34)	6.75E-01	7.21E-01	0.15	(-0.28-0.6)	4.96E-01	5.86E-01	0.45	(-0.36-1.27)	2.78E-01	4.39E-01
15	CE(18:2)	-0.83	(-1.18--0.48)	**4.08E-06**	**1.78E-05**	-0.32	(-0.64-0)	**4.82E-02**	7.03E-02	-0.41	(-0.9-0.12)	1.27E-01	1.98E-01	0.36	(-0.64-1.38)	4.80E-01	6.23E-01
16	CE(18:3)	-1.93	(-2.54--1.32)	**1.28E-09**	**1.25E-08**	0.72	(0.11-1.32)	**2.04E-02**	**3.21E-02**	0.31	(-0.55-1.25)	4.89E-01	5.80E-01	2.78	(0.7-4.91)	**8.76E-03**	**3.24E-02**
17	CE(20:1)	-0.83	(-1.29--0.37)	**4.07E-04**	**1.15E-03**	-0.70	(-1.17--0.22)	**4.06E-03**	**7.58E-03**	0.56	(-0.26-1.44)	1.87E-01	2.71E-01	-1.24	(-2.51-0.06)	6.17E-02	1.45E-01
18	CE(20:2)	-1.33	(-1.74--0.91)	**7.59E-10**	**7.92E-09**	0.10	(-0.26-0.46)	5.84E-01	6.40E-01	0.79	(0.16-1.46)	**1.36E-02**	**2.71E-02**	0.46	(-0.64-1.56)	4.14E-01	5.58E-01
19	CE(20:4)	-0.66	(-1.3--0.02)	**4.41E-02**	7.05E-02	-1.54	(-2.06--1.02)	**1.28E-08**	**5.79E-08**	-0.17	(-0.73-0.43)	5.78E-01	6.63E-01	0.49	(-1.02-2.02)	5.25E-01	6.51E-01
20	CE(20:5)	-1.71	(-3.04--0.36)	**1.31E-02**	**2.41E-02**	-0.98	(-2.2-0.25)	1.18E-01	1.56E-01	-2.42	(-3.39--1.3)	**9.07E-05**	**2.59E-04**	3.76	(0.43-7.2)	**2.68E-02**	7.82E-02
21	CE(22:5) (n6)	-1.88	(-2.46--1.29)	**7.49E-10**	**7.92E-09**	-0.68	(-1.1--0.26)	**1.77E-03**	**3.56E-03**	0.43	(-0.33-1.25)	2.74E-01	3.66E-01	1.10	(-0.31-2.52)	1.25E-01	2.34E-01
22	CE(22:6)	-1.55	(-2.27--0.82)	**3.29E-05**	**1.18E-04**	-0.52	(-1.23-0.2)	1.56E-01	2.01E-01	-1.97	(-2.73--1.12)	**1.95E-05**	**6.12E-05**	2.24	(0.07-4.46)	**4.26E-02**	1.10E-01
23	CE(24:5)	-2.01	(-2.72--1.3)	**4.73E-08**	**3.03E-07**	-0.34	(-1-0.33)	3.22E-01	3.80E-01	-2.88	(-3.68--1.98)	**3.40E-08**	**1.41E-07**	-1.19	(-3.09-0.75)	2.27E-01	3.72E-01
24	CE(24:6)	-2.55	(-3.28--1.82)	**2.50E-11**	**4.44E-10**	-0.16	(-0.84-0.53)	6.43E-01	6.92E-01	-1.14	(-2.05--0.14)	**2.66E-02**	**4.98E-02**	0.84	(-1.15-2.87)	4.10E-01	5.58E-01
25	DG(16:0_16:1)	0.64	(-0.39-1.68)	2.23E-01	2.80E-01	4.43	(3.46-5.4)	**9.58E-19**	**4.12E-17**	-1.32	(-2.11--0.45)	**3.64E-03**	**8.13E-03**	6.21	(3.58-8.89)	**3.25E-06**	**8.67E-05**
26	DG(14:0_18:2)	-1.03	(-2.03--0.02)	**4.55E-02**	7.18E-02	4.19	(2.86-5.54)	**7.61E-10**	**4.11E-09**	-3.08	(-4.04--1.96)	**1.68E-06**	**5.70E-06**	6.70	(3.02-10.51)	**3.23E-04**	**2.28E-03**
27	DG(16:0_18:1)	1.33	(0.59-2.07)	**4.36E-04**	**1.22E-03**	4.26	(3.33-5.21)	**1.32E-18**	**4.86E-17**	-1.91	(-2.57--1.19)	**1.35E-06**	**4.62E-06**	3.90	(1.38-6.49)	**2.40E-03**	**1.18E-02**
28	DG(16:1_18:1)	0.78	(-0.08-1.65)	7.61E-02	1.10E-01	4.04	(3.07-5.03)	**1.05E-15**	**1.80E-14**	-2.08	(-2.77--1.32)	**7.91E-07**	**2.84E-06**	6.17	(3.26-9.17)	**2.92E-05**	**4.64E-04**
29	DG(16:0_18:2)	0.92	(0.21-1.65)	**1.15E-02**	**2.16E-02**	3.62	(2.72-4.52)	**5.53E-15**	**7.59E-14**	-3.07	(-3.76--2.31)	**1.09E-11**	**6.22E-11**	4.00	(1.57-6.48)	**1.20E-03**	**6.49E-03**
30	DG(18:0_18:1)	1.36	(0.69-2.04)	**7.91E-05**	**2.60E-04**	4.41	(3.51-5.31)	**4.70E-21**	**4.51E-19**	-1.29	(-1.99--0.53)	**1.23E-03**	**2.94E-03**	4.06	(1.71-6.46)	**6.87E-04**	**4.23E-03**
31	DG(18:1_18:1)	1.37	(0.64-2.1)	**2.23E-04**	**6.69E-04**	2.93	(2.08-3.77)	**1.44E-11**	**1.03E-10**	-1.85	(-2.5--1.16)	**1.20E-06**	**4.16E-06**	2.05	(-0.25-4.4)	8.10E-02	1.74E-01
32	DG(18:0_18:2)	0.99	(0.23-1.76)	**1.04E-02**	**1.97E-02**	4.40	(3.41-5.39)	**7.51E-18**	**2.57E-16**	-1.62	(-2.52--0.61)	**2.43E-03**	**5.62E-03**	3.76	(1.3-6.29)	**2.68E-03**	**1.27E-02**
33	DG(18:1_18:2)	0.95	(0.22-1.68)	**1.02E-02**	**1.97E-02**	2.26	(1.45-3.08)	**5.91E-08**	**2.42E-07**	-3.15	(-3.76--2.48)	**8.45E-15**	**7.12E-14**	1.89	(-0.33-4.15)	9.51E-02	1.93E-01
34	DG(18:2_18:2)	0.88	(-0.05-1.82)	6.31E-02	9.47E-02	1.84	(0.87-2.81)	**1.86E-04**	**4.53E-04**	-4.16	(-4.84--3.39)	**9.59E-17**	**1.10E-15**	2.33	(-0.23-4.96)	7.47E-02	1.63E-01
35	DG(18:1_18:3)	0.37	(-0.41-1.15)	3.56E-01	4.21E-01	2.29	(1.37-3.21)	**1.02E-06**	**3.33E-06**	-2.63	(-3.39--1.77)	**6.81E-08**	**2.73E-07**	3.15	(0.38-6.01)	**2.58E-02**	7.63E-02
36	DG(16:0_20:4)	1.19	(0.26-2.12)	**1.22E-02**	**2.27E-02**	4.27	(3.25-5.3)	**4.79E-16**	**9.59E-15**	-2.06	(-2.83--1.21)	**9.20E-06**	**2.95E-05**	4.97	(2.34-7.66)	**1.98E-04**	**1.61E-03**
37	DG(18:1_20:3)	0.43	(-0.33-1.19)	2.68E-01	3.33E-01	2.33	(1.5-3.17)	**4.24E-08**	**1.82E-07**	-0.30	(-1.09-0.55)	4.72E-01	5.69E-01	1.17	(-1.22-3.63)	3.39E-01	4.95E-01
38	DG(18:1_20:4)	1.14	(0.4-1.88)	**2.42E-03**	**5.53E-03**	2.47	(1.71-3.22)	**1.81E-10**	**1.10E-09**	-2.06	(-2.71--1.36)	**1.08E-07**	**4.14E-07**	3.90	(1.68-6.16)	**5.47E-04**	**3.60E-03**
39	DG(16:0_22:5)	0.58	(-0.3-1.46)	2.00E-01	2.56E-01	2.42	(1.46-3.38)	**7.01E-07**	**2.39E-06**	-1.95	(-2.72--1.09)	**2.99E-05**	**9.02E-05**	4.60	(2.03-7.23)	**4.22E-04**	**2.89E-03**
40	DG(18:2_20:4)	0.81	(-0.04-1.66)	6.22E-02	9.35E-02	2.42	(1.55-3.3)	**5.34E-08**	**2.23E-07**	-3.14	(-3.87--2.33)	**7.74E-11**	**4.08E-10**	4.23	(1.78-6.73)	**6.78E-04**	**4.23E-03**
41	DG(16:0_22:6)	1.16	(0.04-2.3)	**4.29E-02**	6.89E-02	4.15	(2.87-5.45)	**2.35E-10**	**1.41E-09**	-4.09	(-4.76--3.33)	**6.80E-17**	**8.37E-16**	5.50	(2.13-8.99)	**1.31E-03**	**6.84E-03**

42	DG(18:1_22:5)	0.51	(-0.32-1.35)	2.30E-01	2.89E-01	1.38	(0.54-2.23)	**1.34E-03**	**2.78E-03**	-1.90	(-2.66--1.06)	**2.96E-05**	**9.00E-05**	1.63	(-0.72-4.04)	1.75E-01	3.07E-01
43	DG(18:1_22:6)	1.53	(0.47-2.61)	**4.63E-03**	**9.97E-03**	3.18	(2.04-4.34)	**5.26E-08**	**2.22E-07**	-3.49	(-4.15--2.76)	**7.44E-15**	**6.49E-14**	4.14	(1.32-7.04)	**3.88E-03**	**1.73E-02**
44	DG(18:2_22:6)	1.52	(0.42-2.63)	**6.55E-03**	**1.35E-02**	3.05	(1.87-4.24)	**3.60E-07**	**1.28E-06**	-4.00	(-4.74--3.16)	**9.00E-14**	**6.75E-13**	4.79	(1.45-8.24)	**4.80E-03**	**2.04E-02**
45	TG(48:0) [NL-18:0]	-1.56	(-2.43--0.68)	**5.76E-04**	**1.53E-03**	4.34	(2.99-5.71)	**3.54E-10**	**2.07E-09**	-1.44	(-2.28--0.51)	**3.14E-03**	**7.14E-03**	7.45	(3.74-11.29)	**7.09E-05**	**8.54E-04**
46	TG(48:1) [NL-16:1]	0.51	(-0.62-1.65)	3.76E-01	4.39E-01	6.64	(5.32-7.97)	**1.51E-22**	**3.62E-20**	-2.43	(-3.32--1.41)	**1.61E-05**	**5.11E-05**	9.58	(5.88-13.41)	**2.99E-07**	**1.32E-05**
47	TG(48:1) [NL-18:1]	-0.61	(-1.49-0.27)	1.75E-01	2.30E-01	5.21	(3.76-6.69)	**2.44E-12**	**2.13E-11**	-3.15	(-3.94--2.26)	**2.16E-09**	**9.86E-09**	8.20	(3.97-12.59)	**1.20E-04**	**1.17E-03**
48	TG(48:2) [NL-14:0]	-0.94	(-1.97-0.1)	7.69E-02	1.11E-01	5.21	(3.73-6.72)	**5.81E-12**	**4.73E-11**	-3.76	(-4.46--2.97)	**4.13E-14**	**3.31E-13**	8.73	(4.41-13.22)	**6.10E-05**	**7.91E-04**
49	TG(48:2) [NL-14:1]	-1.20	(-2.25--0.13)	**2.78E-02**	**4.70E-02**	4.79	(3.42-6.18)	**7.51E-12**	**5.72E-11**	-3.06	(-3.81--2.23)	**4.50E-10**	**2.23E-09**	8.90	(4.81-13.16)	**1.61E-05**	**3.36E-04**
50	TG(48:2) [NL-16:1]	-0.17	(-1.47-1.15)	8.03E-01	8.32E-01	5.71	(4.32-7.12)	**1.20E-15**	**1.99E-14**	-2.85	(-3.65--1.96)	**3.48E-08**	**1.43E-07**	10.74	(6.57-15.07)	**3.03E-07**	**1.32E-05**
51	TG(48:2) [NL-18:2]	-1.16	(-2.2--0.12)	**2.91E-02**	**4.87E-02**	5.21	(3.68-6.77)	**2.87E-11**	**2.03E-10**	-4.14	(-4.86--3.32)	**5.42E-15**	**4.91E-14**	8.58	(4.3-13.04)	**7.11E-05**	**8.54E-04**
52	TG(48:3) [NL-14:0]	-1.51	(-2.64--0.37)	**9.55E-03**	**1.85E-02**	4.32	(2.9-5.76)	**2.31E-09**	**1.14E-08**	-4.04	(-4.73--3.26)	**1.02E-15**	**1.04E-14**	9.53	(5.17-14.08)	**1.43E-05**	**3.28E-04**
53	TG(48:3) [NL-16:1]	-1.14	(-2.29-0.02)	5.44E-02	8.37E-02	4.00	(2.79-5.24)	**1.30E-10**	**8.10E-10**	-2.89	(-3.59--2.12)	**1.90E-10**	**9.60E-10**	8.59	(4.99-12.31)	**2.28E-06**	**6.49E-05**
54	TG(48:3) [NL-18:3]	-1.81	(-3.07--0.53)	**5.76E-03**	**1.21E-02**	4.99	(3.41-6.59)	**5.23E-10**	**2.92E-09**	-3.23	(-3.91--2.48)	**8.75E-13**	**5.83E-12**	8.18	(3.69-12.86)	**3.09E-04**	**2.27E-03**
55	TG(49:1) [NL-17:1]	0.33	(-0.53-1.21)	4.51E-01	5.10E-01	4.04	(3.11-4.97)	**2.94E-17**	**8.82E-16**	-2.19	(-2.84--1.47)	**5.29E-08**	**2.14E-07**	4.94	(2.47-7.47)	**8.54E-05**	**9.54E-04**
56	TG(50:1) [NL-14:0]	-0.81	(-1.57--0.05)	**3.75E-02**	6.17E-02	4.78	(3.52-6.05)	**1.11E-13**	**1.24E-12**	-1.46	(-2.35--0.47)	**4.76E-03**	**1.03E-02**	6.89	(3.47-10.42)	**7.00E-05**	**8.54E-04**
57	TG(50:1) [NL-16:0]	0.83	(0.22-1.43)	**7.27E-03**	**1.46E-02**	4.34	(3.39-5.3)	**1.03E-18**	**4.12E-17**	-2.49	(-3.25--1.64)	**2.17E-07**	**8.15E-07**	4.63	(2.1-7.22)	**3.13E-04**	**2.27E-03**
58	TG(50:1) [NL-18:1]	0.76	(0.18-1.34)	**1.03E-02**	**1.97E-02**	4.10	(3.16-5.05)	**4.06E-17**	**1.08E-15**	-2.47	(-3.24--1.62)	**2.61E-07**	**9.63E-07**	4.17	(1.73-6.68)	**7.99E-04**	**4.74E-03**
59	TG(50:2) [NL-14:0]	-0.65	(-1.21--0.07)	**2.69E-02**	**4.57E-02**	2.80	(1.76-3.86)	**1.50E-07**	**5.54E-07**	-3.34	(-4.1--2.48)	**1.16E-10**	**6.01E-10**	4.36	(1.14-7.69)	**7.83E-03**	**3.01E-02**
60	TG(50:2) [NL-16:1]	0.65	(-0.07-1.38)	7.62E-02	1.10E-01	4.04	(3.07-5.02)	**6.67E-16**	**1.28E-14**	-3.01	(-3.81--2.11)	**1.08E-08**	**4.67E-08**	6.45	(3.42-9.58)	**2.79E-05**	**4.62E-04**
61	TG(50:2) [NL-18:1]	0.32	(-0.31-0.95)	3.19E-01	3.85E-01	3.59	(2.65-4.54)	**1.19E-13**	**1.30E-12**	-3.12	(-3.91--2.21)	**4.54E-09**	**2.00E-08**	6.24	(3.2-9.38)	**5.20E-05**	**7.33E-04**
62	TG(50:2) [NL-18:2]	0.66	(0.08-1.24)	**2.58E-02**	**4.41E-02**	3.72	(2.82-4.62)	**8.45E-16**	**1.53E-14**	-4.17	(-4.92--3.31)	**5.26E-14**	**4.14E-13**	4.53	(2.11-7)	**2.30E-04**	**1.78E-03**
63	TG(50:3) [NL-14:0]	-0.51	(-1.24-0.22)	1.70E-01	2.25E-01	3.13	(1.93-4.34)	**2.88E-07**	**1.04E-06**	-4.25	(-4.95--3.45)	**4.04E-16**	**4.31E-15**	5.47	(1.88-9.18)	**2.69E-03**	**1.27E-02**
64	TG(50:3) [NL-14:1]	-0.64	(-1.29-0.02)	5.67E-02	8.67E-02	2.66	(1.69-3.63)	**7.85E-08**	**3.14E-07**	-2.39	(-3.17--1.51)	**1.11E-06**	**3.90E-06**	4.17	(1.33-7.09)	**3.92E-03**	**1.73E-02**
65	TG(50:3) [NL-16:1]	0.58	(-0.25-1.43)	1.70E-01	2.25E-01	4.38	(3.28-5.5)	**1.32E-14**	**1.67E-13**	-3.88	(-4.58--3.09)	**8.11E-15**	**6.95E-14**	8.28	(4.83-11.85)	**2.15E-06**	**6.49E-05**
66	TG(50:3) [NL-18:2]	0.28	(-0.45-1.02)	4.51E-01	5.10E-01	3.72	(2.65-4.81)	**1.38E-11**	**1.00E-10**	-4.37	(-5.06--3.59)	**3.53E-17**	**4.70E-16**	7.14	(3.78-10.61)	**2.72E-05**	**4.62E-04**
67	TG(50:3) [NL-18:3]	0.14	(-0.78-1.07)	7.65E-01	8.02E-01	5.18	(3.92-6.44)	**8.59E-16**	**1.53E-14**	-3.81	(-4.54--2.98)	**2.31E-13**	**1.65E-12**	6.76	(3.2-10.44)	**1.78E-04**	**1.52E-03**
68	TG(50:4) [NL-14:0]	-0.87	(-1.89-0.15)	9.35E-02	1.31E-01	2.75	(1.47-4.04)	**2.32E-05**	**6.36E-05**	-4.31	(-4.97--3.57)	**2.52E-18**	**4.32E-17**	5.22	(1.61-8.96)	**4.43E-03**	**1.92E-02**
69	TG(50:4) [NL-18:3]	-0.72	(-1.76-0.33)	1.76E-01	2.31E-01	4.14	(2.91-5.38)	**5.20E-11**	**3.51E-10**	-3.93	(-4.56--3.22)	**9.72E-18**	**1.37E-16**	7.93	(4.25-11.74)	**2.01E-05**	**4.02E-04**
70	TG(50:4) [NL-20:4]	-0.81	(-2.19-0.58)	2.50E-01	3.11E-01	5.64	(4.02-7.29)	**8.80E-12**	**6.60E-11**	-3.52	(-4.34--2.57)	**6.23E-10**	**3.05E-09**	10.00	(5.69-14.48)	**3.96E-06**	**1.00E-04**
71	TG(51:1) [NL-17:0]	0.67	(-0.08-1.42)	7.92E-02	1.13E-01	4.54	(3.5-5.59)	**2.82E-17**	**8.82E-16**	-2.72	(-3.44--1.91)	**4.45E-09**	**1.98E-08**	4.90	(2-7.89)	**8.93E-04**	**5.23E-03**
72	TG(51:2) [NL-15:0]	-0.52	(-1.18-0.13)	1.19E-01	1.65E-01	2.32	(1.4-3.24)	**7.50E-07**	**2.50E-06**	-3.37	(-4.11--2.54)	**1.77E-11**	**9.75E-11**	2.95	(0.1-5.89)	**4.21E-02**	1.10E-01
73	TG(51:2) [NL-17:0]	0.37	(-0.32-1.06)	2.96E-01	3.59E-01	4.07	(3.06-5.09)	**6.13E-15**	**8.18E-14**	-3.40	(-4.11--2.6)	**2.54E-12**	**1.63E-11**	5.48	(2.52-8.52)	**2.67E-04**	**2.03E-03**
74	TG(51:2) [NL-17:1]	0.36	(-0.31-1.02)	2.93E-01	3.56E-01	3.80	(2.86-4.74)	**3.86E-15**	**5.98E-14**	-2.89	(-3.53--2.19)	**2.44E-12**	**1.58E-11**	4.90	(2.22-7.65)	**3.17E-04**	**2.27E-03**
75	TG(52:1) [NL-18:0]	1.38	(0.59-2.18)	**6.58E-04**	**1.73E-03**	5.69	(4.53-6.86)	**2.15E-21**	**2.58E-19**	-1.86	(-2.76--0.83)	**6.81E-04**	**1.69E-03**	5.79	(2.8-8.86)	**1.32E-04**	**1.22E-03**
76	TG(52:1) [NL-18:1]	1.45	(0.69-2.23)	**2.04E-04**	**6.17E-04**	5.86	(4.73-7)	**1.79E-23**	**8.60E-21**	-1.80	(-2.71--0.77)	**9.96E-04**	**2.41E-03**	5.71	(2.83-8.68)	**9.59E-05**	**1.00E-03**
77	TG(52:2) [NL-16:0]	0.80	(0.4-1.21)	**1.01E-04**	**3.29E-04**	2.18	(1.57-2.79)	**3.69E-12**	**3.11E-11**	-2.79	(-3.44--2.07)	**3.12E-11**	**1.68E-10**	1.93	(0.21-3.67)	**2.76E-02**	7.97E-02
78	TG(52:2) [NL-18:2]	0.46	(-0.15-1.07)	1.38E-01	1.88E-01	4.17	(3.27-5.07)	**4.36E-19**	**2.33E-17**	-2.75	(-3.58--1.81)	**3.20E-07**	**1.17E-06**	4.45	(2.1-6.85)	**1.98E-04**	**1.61E-03**
79	TG(52:3) [NL-16:1]	0.39	(-0.18-0.97)	1.79E-01	2.35E-01	1.94	(1.21-2.66)	**1.73E-07**	**6.33E-07**	-3.37	(-4.1--2.56)	**1.00E-11**	**5.79E-11**	3.39	(0.97-5.88)	**5.95E-03**	**2.36E-02**
80	TG(52:3) [NL-18:2]	0.76	(0.32-1.2)	**8.08E-04**	**2.08E-03**	1.84	(1.22-2.48)	**1.11E-08**	**5.14E-08**	-4.40	(-5.07--3.64)	**3.04E-18**	**4.87E-17**	1.85	(-0.08-3.81)	6.03E-02	1.43E-01
81	TG(52:4) [NL-16:1]	0.68	(-0.05-1.41)	6.77E-02	9.99E-02	2.22	(1.27-3.18)	**4.67E-06**	**1.43E-05**	-4.52	(-5.17--3.78)	**1.14E-19**	**2.48E-18**	4.16	(1.08-7.34)	**7.91E-03**	**3.01E-02**
82	TG(52:4) [NL-18:2]	1.20	(0.44-1.96)	**1.92E-03**	**4.52E-03**	2.29	(1.29-3.3)	**7.06E-06**	**2.12E-05**	-4.97	(-5.63--4.21)	**2.07E-20**	**5.23E-19**	3.62	(0.64-6.69)	**1.72E-02**	5.52E-02
83	TG(52:4) [NL-18:3]	0.47	(-0.24-1.18)	1.97E-01	2.54E-01	3.19	(2.17-4.21)	**8.03E-10**	**4.24E-09**	-4.11	(-4.78--3.35)	**9.43E-17**	**1.10E-15**	4.40	(1.43-7.46)	**3.60E-03**	**1.63E-02**
84	TG(52:5) [NL-18:3]	0.10	(-0.9-1.11)	8.49E-01	8.72E-01	2.89	(1.78-4.01)	**3.35E-07**	**1.20E-06**	-4.16	(-4.81--3.43)	**2.87E-18**	**4.75E-17**	5.19	(2-8.47)	**1.36E-03**	**6.99E-03**
85	TG(52:5) [NL-20:4]	0.48	(-0.71-1.69)	4.28E-01	4.86E-01	5.46	(4.2-6.74)	**3.97E-17**	**1.08E-15**	-3.63	(-4.4--2.74)	**2.19E-11**	**1.19E-10**	10.03	(6.39-13.8)	**4.75E-08**	**4.56E-06**
86	TG(52:5) [NL-20:5]	0.85	(-0.43-2.14)	1.95E-01	2.52E-01	5.06	(3.69-6.44)	**4.80E-13**	**4.70E-12**	-4.43	(-5.13--3.64)	**4.66E-17**	**5.88E-16**	7.78	(4.53-11.13)	**2.30E-06**	**6.49E-05**
87	TG(53:2) [NL-18:1]	0.88	(0.2-1.56)	**1.08E-02**	**2.03E-02**	2.66	(1.85-3.47)	**1.63E-10**	**1.00E-09**	-2.69	(-3.33--1.98)	**5.26E-11**	**2.81E-10**	1.90	(-0.42-4.27)	1.10E-01	2.14E-01
88	TG(54:1) [NL-18:1]	0.99	(0.32-1.66)	**3.57E-03**	**8.00E-03**	4.45	(3.57-5.34)	**4.54E-22**	**7.26E-20**	-1.64	(-2.56--0.59)	**2.89E-03**	**6.61E-03**	3.54	(1.13-6.02)	**3.96E-03**	**1.73E-02**
89	TG(54:2) [NL-18:0]	1.41	(0.72-2.1)	**6.55E-05**	**2.23E-04**	4.24	(3.35-5.13)	**2.72E-20**	**1.63E-18**	-1.85	(-2.69--0.91)	**2.40E-04**	**6.39E-04**	3.30	(0.94-5.71)	**6.02E-03**	**2.36E-02**

90	TG(54:2) [NL-20:1]	0.43	(-0.34-1.21)	2.74E-01	3.39E-01	2.74	(1.85-3.64)	**1.93E-09**	**9.85E-09**	-1.84	(-2.64--0.95)	**1.25E-04**	**3.49E-04**	1.66	(-0.73-4.12)	1.74E-01	3.07E-01
91	TG(54:3) [NL-18:1]	0.57	(0.01-1.13)	**4.50E-02**	7.13E-02	1.26	(0.64-1.88)	**7.52E-05**	**1.93E-04**	-2.18	(-2.89--1.41)	**4.18E-07**	**1.52E-06**	0.18	(-1.55-1.95)	8.36E-01	8.99E-01
92	TG(54:3) [NL-18:2]	0.72	(0.14-1.3)	**1.43E-02**	**2.59E-02**	2.23	(1.57-2.89)	**5.13E-11**	**3.51E-10**	-3.06	(-3.75--2.3)	**1.28E-11**	**7.23E-11**	2.32	(0.48-4.19)	**1.32E-02**	**4.65E-02**
93	TG(54:4) [NL-18:2]	0.35	(-0.28-0.98)	2.76E-01	3.40E-01	0.85	(0.16-1.55)	**1.61E-02**	**2.62E-02**	-4.03	(-4.74--3.23)	**5.43E-15**	**4.91E-14**	0.62	(-1.51-2.8)	5.68E-01	6.95E-01
94	TG(54:4) [NL-20:3]	0.64	(-0.05-1.33)	6.76E-02	9.99E-02	3.70	(2.83-4.58)	**2.18E-16**	**4.75E-15**	-2.73	(-3.52--1.84)	**8.04E-08**	**3.16E-07**	4.94	(2.41-7.54)	**1.24E-04**	**1.19E-03**
95	TG(54:5) [NL-18:3]	0.07	(-0.8-0.94)	8.78E-01	8.96E-01	1.87	(0.89-2.85)	**1.77E-04**	**4.33E-04**	-3.77	(-4.42--3.03)	**5.03E-16**	**5.25E-15**	2.95	(-0.03-6.02)	5.22E-02	1.29E-01
96	TG(54:5) [NL-20:4]	1.04	(0.38-1.71)	**1.91E-03**	**4.51E-03**	3.37	(2.55-4.2)	**2.12E-15**	**3.39E-14**	-3.73	(-4.49--2.86)	**4.40E-12**	**2.64E-11**	5.84	(3.43-8.3)	**1.90E-06**	**6.49E-05**
97	TG(54:6) [NL-18:3]	-0.40	(-1.56-0.76)	4.95E-01	5.50E-01	1.37	(0.22-2.54)	**1.91E-02**	**3.03E-02**	-3.86	(-4.52--3.12)	**3.29E-16**	**3.59E-15**	2.95	(-0.25-6.26)	7.12E-02	1.58E-01
98	TG(54:6) [NL-20:4]	1.16	(0.33-2)	**6.24E-03**	**1.29E-02**	3.69	(2.71-4.68)	**2.17E-13**	**2.27E-12**	-4.11	(-4.83--3.28)	**1.19E-14**	**9.81E-14**	7.52	(4.68-10.44)	**1.91E-07**	**1.15E-05**
99	TG(54:6) [NL-20:5]	0.85	(-0.29-1.99)	1.44E-01	1.96E-01	3.82	(2.52-5.13)	**7.53E-09**	**3.51E-08**	-5.04	(-5.69--4.28)	**7.74E-21**	**2.50E-19**	8.39	(5.04-11.85)	**7.54E-07**	**3.02E-05**
100	TG(54:6) [NL-22:6]	1.79	(0.57-3.02)	**4.10E-03**	**9.06E-03**	5.76	(4.33-7.22)	**4.96E-15**	**7.00E-14**	-5.16	(-5.83--4.38)	**1.98E-20**	**5.23E-19**	8.20	(4.48-12.06)	**1.29E-05**	**3.10E-04**
101	TG(54:7) [NL-20:5]	0.47	(-0.81-1.77)	4.74E-01	5.30E-01	3.45	(2.17-4.75)	**1.37E-07**	**5.08E-07**	-4.97	(-5.63--4.22)	**1.23E-20**	**3.49E-19**	8.46	(5.22-11.8)	**2.49E-07**	**1.32E-05**
102	TG(54:7) [NL-22:6]	0.98	(-0.34-2.32)	1.48E-01	1.99E-01	5.09	(3.68-6.52)	**1.79E-12**	**1.62E-11**	-4.92	(-5.56--4.18)	**1.93E-21**	**7.12E-20**	10.42	(6.51-14.47)	**1.23E-07**	**9.83E-06**
103	TG(56:6) [NL-20:4]	1.34	(0.81-1.87)	**7.12E-07**	**3.60E-06**	1.36	(0.85-1.88)	**2.38E-07**	**8.65E-07**	-3.00	(-3.77--2.15)	**1.89E-09**	**8.92E-09**	3.72	(1.91-5.57)	**5.78E-05**	**7.86E-04**
104	TG(56:6) [NL-22:5]	0.62	(-0.03-1.28)	6.20E-02	9.35E-02	1.99	(1.2-2.77)	**7.33E-07**	**2.46E-06**	-3.83	(-4.52--3.06)	**4.63E-15**	**4.36E-14**	3.96	(1.82-6.14)	**2.87E-04**	**2.15E-03**
105	TG(56:7) [NL-20:4]	1.02	(0.35-1.7)	**2.84E-03**	**6.40E-03**	1.88	(1.14-2.62)	**6.63E-07**	**2.29E-06**	-3.66	(-4.41--2.81)	**3.38E-12**	**2.08E-11**	4.16	(1.73-6.64)	**7.60E-04**	**4.56E-03**
106	TG(56:7) [NL-20:5]	0.39	(-0.72-1.5)	4.93E-01	5.50E-01	2.47	(1.35-3.61)	**1.56E-05**	**4.46E-05**	-3.79	(-4.51--2.97)	**1.22E-13**	**9.04E-13**	5.58	(2.64-8.6)	**1.82E-04**	**1.54E-03**
107	TG(56:7) [NL-22:5]	0.79	(0.02-1.56)	**4.44E-02**	7.06E-02	2.55	(1.61-3.5)	**1.14E-07**	**4.45E-07**	-4.29	(-4.93--3.57)	**2.43E-19**	**4.87E-18**	5.15	(2.46-7.91)	**1.64E-04**	**1.46E-03**
108	TG(56:7) [NL-22:6]	1.93	(0.84-3.04)	**4.97E-04**	**1.35E-03**	3.96	(2.65-5.28)	**3.33E-09**	**1.61E-08**	-5.07	(-5.72--4.33)	**1.85E-21**	**7.12E-20**	6.45	(3.01-10)	**2.16E-04**	**1.70E-03**
109	TG(56:8) [NL-20:4]	0.80	(-0.13-1.74)	9.22E-02	1.30E-01	1.88	(1-2.77)	**2.85E-05**	**7.72E-05**	-2.95	(-3.72--2.08)	**5.67E-09**	**2.47E-08**	5.12	(2.54-7.75)	**9.23E-05**	**1.00E-03**
110	TG(56:8) [NL-20:5]	0.18	(-1.06-1.45)	7.72E-01	8.06E-01	1.59	(0.43-2.77)	**7.20E-03**	**1.26E-02**	-4.13	(-4.79--3.37)	**3.84E-17**	**4.98E-16**	5.03	(2.01-8.13)	**1.03E-03**	**5.94E-03**
111	TG(56:8) [NL-22:6]	1.76	(0.54-2.99)	**4.49E-03**	**9.76E-03**	3.97	(2.64-5.31)	**4.96E-09**	**2.38E-08**	-5.36	(-5.97--4.65)	**1.69E-24**	**1.02E-22**	7.57	(4.02-11.24)	**2.51E-05**	**4.62E-04**
112	TG(56:9) [NL-22:6]	0.79	(-0.4-1.99)	1.94E-01	2.50E-01	3.07	(1.89-4.27)	**3.82E-07**	**1.35E-06**	-4.88	(-5.5--4.17)	**1.57E-22**	**7.54E-21**	6.66	(3.29-10.14)	**9.49E-05**	**1.00E-03**
113	TG(58:10) [NL-22:6]	0.95	(-0.23-2.15)	1.13E-01	1.58E-01	2.08	(0.92-3.25)	**4.34E-04**	**9.92E-04**	-4.48	(-5.11--3.76)	**3.35E-20**	**8.05E-19**	5.17	(1.89-8.56)	**1.90E-03**	**9.52E-03**
114	TG(58:8) [NL-22:6]	1.46	(0.38-2.56)	**7.98E-03**	**1.58E-02**	2.80	(1.58-4.04)	**7.21E-06**	**2.15E-05**	-4.50	(-5.16--3.76)	**3.67E-19**	**7.05E-18**	4.73	(1.39-8.18)	**5.34E-03**	**2.16E-02**
115	TG(58:9) [NL-22:6]	1.22	(0.07-2.38)	**3.70E-02**	6.10E-02	2.17	(0.97-3.38)	**3.75E-04**	**8.69E-04**	-4.76	(-5.39--4.06)	**4.43E-22**	**1.93E-20**	4.51	(1.17-7.97)	**7.98E-03**	**3.02E-02**
116	TG(O-50:1) [NL-16:0]	-0.20	(-0.8-0.39)	5.04E-01	5.56E-01	0.74	(-0.02-1.51)	5.73E-02	8.14E-02	-0.59	(-1.3-0.18)	1.29E-01	2.00E-01	1.88	(-0.28-4.09)	8.85E-02	1.85E-01
117	TG(O-50:1) [NL-17:1]	0.33	(-0.54-1.21)	4.53E-01	5.11E-01	4.08	(3.14-5.03)	**4.97E-17**	**1.26E-15**	-2.21	(-2.88--1.47)	**9.38E-08**	**3.66E-07**	4.99	(2.47-7.57)	**1.01E-04**	**1.03E-03**
118	TG(O-50:1) [NL-18:1]	-0.61	(-1.27-0.05)	6.87E-02	1.01E-01	0.57	(-0.34-1.49)	2.18E-01	2.74E-01	-0.98	(-1.68--0.22)	**1.24E-02**	**2.49E-02**	2.75	(0.37-5.19)	**2.36E-02**	7.16E-02
119	TG(O-50:2) [NL-18:2]	-0.07	(-0.64-0.51)	8.21E-01	8.50E-01	0.77	(0.05-1.5)	**3.73E-02**	5.56E-02	-0.45	(-1.21-0.36)	2.64E-01	3.60E-01	2.97	(0.89-5.09)	**5.11E-03**	**2.10E-02**
120	TG(O-52:2) [NL-16:0]	-0.21	(-1.02-0.6)	6.04E-01	6.52E-01	2.25	(1.31-3.2)	**2.59E-06**	**8.08E-06**	-1.15	(-2.06--0.15)	**2.56E-02**	**4.81E-02**	3.32	(0.48-6.25)	**2.20E-02**	6.79E-02
121	TG(O-52:2) [NL-17:1]	0.36	(-0.31-1.03)	2.93E-01	3.56E-01	3.82	(2.87-4.77)	**4.43E-15**	**6.45E-14**	-2.91	(-3.55--2.2)	**4.20E-12**	**2.55E-11**	4.89	(2.19-7.66)	**3.63E-04**	**2.52E-03**
122	TG(O-52:2) [NL-18:1]	-0.45	(-1.28-0.38)	2.85E-01	3.50E-01	2.26	(1.27-3.27)	**8.29E-06**	**2.44E-05**	-1.84	(-2.72--0.85)	**5.37E-04**	**1.36E-03**	5.04	(2-8.16)	**1.08E-03**	**6.15E-03**
123	TG(O-54:2) [NL-18:1]	0.85	(0.18-1.53)	**1.33E-02**	**2.43E-02**	2.63	(1.82-3.44)	**2.48E-10**	**1.47E-09**	-2.66	(-3.31--1.95)	**1.06E-10**	**5.53E-10**	1.86	(-0.46-4.23)	1.16E-01	2.20E-01
124	TG(O-54:4) [NL-17:1]	0.08	(-0.68-0.85)	8.36E-01	8.61E-01	1.61	(0.72-2.51)	**4.02E-04**	**9.24E-04**	-4.03	(-4.73--3.24)	**1.59E-15**	**1.56E-14**	2.88	(0.29-5.53)	**2.92E-02**	8.30E-02
125	TG(O-54:4) [NL-18:2]	0.07	(-0.73-0.88)	8.68E-01	8.88E-01	1.49	(0.53-2.46)	**2.38E-03**	**4.69E-03**	-4.57	(-5.24--3.8)	**1.07E-18**	**1.90E-17**	2.73	(-0.04-5.59)	5.37E-02	1.31E-01
126	dhCer(d18:0/22:0)	1.14	(0.5-1.79)	**4.77E-04**	**1.32E-03**	1.96	(1.35-2.58)	**4.98E-10**	**2.81E-09**	-0.06	(-0.73-0.66)	8.74E-01	9.06E-01	1.23	(-0.54-3.02)	1.74E-01	3.07E-01
127	dhCer(d18:0/24:0)	0.21	(-0.38-0.8)	4.92E-01	5.49E-01	0.98	(0.38-1.57)	**1.25E-03**	**2.63E-03**	-0.54	(-1.2-0.18)	1.36E-01	2.10E-01	-0.03	(-1.63-1.6)	9.71E-01	9.87E-01
128	dhCer(d18:0/24:1)	1.70	(0.99-2.41)	**3.01E-06**	**1.36E-05**	1.49	(0.78-2.2)	**4.22E-05**	**1.11E-04**	-0.27	(-1.09-0.63)	5.47E-01	6.37E-01	1.59	(-0.43-3.66)	1.24E-01	2.33E-01
129	Cer(d16:1/22:0)	-0.73	(-1.33--0.13)	**1.79E-02**	**3.18E-02**	1.20	(0.56-1.84)	**2.31E-04**	**5.51E-04**	-1.03	(-1.75--0.25)	**1.07E-02**	**2.19E-02**	2.30	(0.33-4.31)	**2.24E-02**	6.84E-02
130	Cer(d16:1/23:0)	-0.99	(-1.66--0.32)	**3.93E-03**	**8.74E-03**	0.76	(0.1-1.42)	**2.38E-02**	**3.69E-02**	-1.07	(-2.23-0.26)	1.10E-01	1.74E-01	2.15	(-0.09-4.44)	6.04E-02	1.43E-01
131	Cer(d16:1/24:0)	-2.03	(-2.65--1.4)	**5.17E-10**	**5.77E-09**	0.28	(-0.34-0.91)	3.74E-01	4.29E-01	-0.79	(-1.51--0.01)	**4.60E-02**	8.21E-02	1.80	(-0.29-3.93)	9.09E-02	1.87E-01
132	Cer(d16:1/24:1)	-0.40	(-1.03-0.23)	2.11E-01	2.70E-01	0.69	(0.07-1.32)	**2.90E-02**	**4.44E-02**	-0.52	(-1.39-0.44)	2.75E-01	3.66E-01	1.85	(0.02-3.71)	**4.75E-02**	1.20E-01
133	Cer(d17:1/22:0)	-1.27	(-1.92--0.61)	**1.62E-04**	**5.04E-04**	0.68	(0.02-1.35)	**4.48E-02**	6.57E-02	-0.82	(-1.72-0.18)	1.04E-01	1.66E-01	0.95	(-0.94-2.87)	3.27E-01	4.87E-01
134	Cer(d17:1/23:0)	-1.28	(-1.96--0.6)	**2.35E-04**	**6.97E-04**	0.02	(-0.67-0.72)	9.57E-01	9.69E-01	-1.29	(-2.61-0.26)	9.78E-02	1.58E-01	1.83	(-0.39-4.1)	1.06E-01	2.10E-01
135	Cer(d17:1/24:0)	-2.19	(-2.76--1.61)	**4.35E-13**	**1.39E-11**	-0.28	(-0.85-0.3)	3.45E-01	4.00E-01	-1.07	(-1.78--0.28)	**8.53E-03**	**1.79E-02**	0.10	(-1.77-2.02)	9.13E-01	9.57E-01
136	Cer(d17:1/24:1)	-1.05	(-1.67--0.43)	**8.90E-04**	**2.26E-03**	-0.29	(-0.87-0.29)	3.24E-01	3.81E-01	-0.91	(-1.72--0.03)	**4.31E-02**	7.72E-02	1.03	(-0.88-2.98)	2.92E-01	4.50E-01
137	Cer(d18:1/16:0)	-0.87	(-1.33--0.4)	**2.78E-04**	**8.08E-04**	-0.36	(-0.81-0.09)	1.17E-01	1.56E-01	-0.52	(-1.09-0.1)	9.85E-02	1.58E-01	-0.45	(-1.74-0.85)	4.93E-01	6.31E-01

138	Cer(d18:1/18:0)	1.97	(1.31-2.64)	**7.34E-09**	**5.29E-08**	2.07	(1.45-2.7)	**1.07E-10**	**6.87E-10**	-0.68	(-1.34-0.03)	6.05E-02	1.04E-01	1.92	(0.19-3.69)	**2.95E-02**	8.32E-02
139	Cer(d18:1/20:0)	0.23	(-0.34-0.8)	4.26E-01	4.86E-01	0.79	(0.25-1.33)	**3.92E-03**	**7.41E-03**	-0.73	(-1.36--0.05)	**3.51E-02**	6.41E-02	0.96	(-0.59-2.54)	2.26E-01	3.71E-01
140	Cer(d18:1/22:0)	-0.08	(-0.56-0.4)	7.34E-01	7.74E-01	0.97	(0.48-1.47)	**1.24E-04**	**3.06E-04**	-0.09	(-0.69-0.55)	7.78E-01	8.43E-01	0.93	(-0.49-2.36)	2.00E-01	3.37E-01
141	Cer(d18:1/23:0)	-0.40	(-0.88-0.09)	1.07E-01	1.50E-01	0.49	(0-0.99)	**4.97E-02**	7.21E-02	-0.41	(-1.17-0.41)	3.11E-01	4.09E-01	1.11	(-0.37-2.61)	1.43E-01	2.63E-01
142	Cer(d18:1/24:0)	-0.95	(-1.41--0.48)	**7.18E-05**	**2.39E-04**	0.09	(-0.39-0.56)	7.23E-01	7.62E-01	-0.39	(-1-0.25)	2.23E-01	3.11E-01	0.03	(-1.3-1.38)	9.63E-01	9.85E-01
143	Cer(d18:1/24:1)	0.09	(-0.44-0.62)	7.49E-01	7.88E-01	0.06	(-0.44-0.56)	8.23E-01	8.59E-01	-0.31	(-0.96-0.38)	3.67E-01	4.68E-01	0.62	(-0.76-2.03)	3.78E-01	5.29E-01
144	Cer(d18:2/16:0)	-0.40	(-1.02-0.23)	2.17E-01	2.76E-01	0.17	(-0.43-0.79)	5.75E-01	6.33E-01	0.15	(-0.78-1.18)	7.60E-01	8.29E-01	0.35	(-1.47-2.2)	7.08E-01	7.93E-01
145	Cer(d18:2/22:0)	-0.35	(-0.91-0.21)	2.22E-01	2.80E-01	0.39	(-0.18-0.97)	1.77E-01	2.27E-01	-0.26	(-0.97-0.5)	4.89E-01	5.80E-01	0.35	(-1.27-1.99)	6.76E-01	7.65E-01
146	Cer(d18:2/23:0)	-0.56	(-1.16-0.03)	6.45E-02	9.61E-02	-0.11	(-0.69-0.48)	7.09E-01	7.53E-01	-0.40	(-1.35-0.64)	4.35E-01	5.35E-01	1.17	(-0.56-2.92)	1.85E-01	3.18E-01
147	Cer(d18:2/24:0)	-1.25	(-1.79--0.69)	**1.15E-05**	**4.61E-05**	-0.68	(-1.22--0.14)	**1.42E-02**	**2.32E-02**	-0.83	(-1.51--0.08)	**3.00E-02**	5.52E-02	-0.65	(-2.18-0.91)	4.11E-01	5.58E-01
148	Cer(d18:2/24:1)	-0.04	(-0.61-0.54)	8.96E-01	9.10E-01	-0.32	(-0.88-0.24)	2.57E-01	3.10E-01	-0.02	(-0.75-0.78)	9.64E-01	9.75E-01	0.54	(-1.01-2.11)	4.96E-01	6.33E-01
149	Cer(d19:1/24:0)	-1.22	(-2.12--0.32)	**8.01E-03**	**1.58E-02**	1.08	(0.23-1.93)	**1.29E-02**	**2.13E-02**	-1.23	(-2.03--0.34)	**7.85E-03**	**1.67E-02**	0.08	(-2.67-2.91)	9.55E-01	9.81E-01
150	Cer(d19:1/24:1)	-0.31	(-1.35-0.74)	5.63E-01	6.17E-01	1.17	(0.21-2.14)	**1.64E-02**	**2.66E-02**	-1.22	(-2.19--0.13)	**2.94E-02**	5.42E-02	1.08	(-2.03-4.28)	5.00E-01	6.34E-01
151	Cer(m18:0/22:0)	0.25	(-0.53-1.04)	5.30E-01	5.82E-01	2.95	(2.15-3.76)	**8.19E-13**	**7.86E-12**	-1.25	(-2.16--0.23)	**1.75E-02**	**3.42E-02**	2.43	(0.5-4.39)	**1.34E-02**	**4.68E-02**
152	Cer(m18:0/23:0)	0.41	(-0.46-1.28)	3.58E-01	4.22E-01	2.81	(1.92-3.7)	**6.59E-10**	**3.60E-09**	-1.33	(-3.02-0.76)	1.95E-01	2.82E-01	2.72	(0.35-5.15)	**2.45E-02**	7.30E-02
153	Cer(m18:0/24:0)	-0.62	(-1.4-0.17)	1.22E-01	1.69E-01	1.77	(1.04-2.5)	**1.94E-06**	**6.16E-06**	-1.21	(-1.95--0.4)	**4.18E-03**	**9.24E-03**	2.12	(0.28-3.98)	**2.38E-02**	7.17E-02
154	Cer(m18:0/24:1)	1.15	(0.26-2.04)	**1.11E-02**	**2.08E-02**	2.84	(2-3.68)	**3.94E-11**	**2.74E-10**	-1.46	(-2.42--0.37)	**9.81E-03**	**2.02E-02**	4.35	(2.12-6.63)	**1.28E-04**	**1.20E-03**
155	Cer(m18:1/22:0)	0.63	(-0.22-1.49)	1.44E-01	1.96E-01	3.00	(2.15-3.86)	**6.87E-12**	**5.32E-11**	-2.51	(-3.17--1.79)	**9.94E-10**	**4.73E-09**	1.19	(-1.06-3.48)	3.01E-01	4.58E-01
156	Cer(m18:1/23:0)	0.17	(-0.73-1.08)	7.11E-01	7.52E-01	2.64	(1.71-3.57)	**2.81E-08**	**1.23E-07**	-3.37	(-4.59--1.88)	**7.84E-05**	**2.27E-04**	2.08	(-0.45-4.68)	1.07E-01	2.12E-01
157	Cer(m18:1/24:0)	-0.42	(-1.2-0.36)	2.91E-01	3.56E-01	2.12	(1.35-2.89)	**7.27E-08**	**2.96E-07**	-2.59	(-3.23--1.88)	**1.87E-10**	**9.55E-10**	-0.03	(-2.04-2.03)	9.80E-01	9.92E-01
158	Cer(m18:1/24:1)	1.37	(0.47-2.27)	**2.70E-03**	**6.11E-03**	2.73	(1.88-3.59)	**4.23E-10**	**2.42E-09**	-2.16	(-2.91--1.32)	**3.30E-06**	**1.10E-05**	1.88	(-0.3-4.1)	9.05E-02	1.87E-01
159	GM3(d18:1/18:0)	-1.16	(-1.7--0.62)	**2.65E-05**	**9.76E-05**	-0.70	(-1.2--0.2)	**5.94E-03**	**1.06E-02**	-0.78	(-1.37--0.16)	**1.53E-02**	**3.01E-02**	-1.22	(-2.62-0.19)	8.96E-02	1.86E-01
160	GM3(d18:1/22:0)	-2.16	(-2.77--1.54)	**2.44E-11**	**4.44E-10**	0.01	(-0.56-0.59)	9.60E-01	9.71E-01	0.09	(-0.88-1.16)	8.64E-01	9.01E-01	-0.55	(-2.13-1.07)	5.03E-01	6.36E-01
161	GM3(d18:1/24:0)	-3.17	(-3.78--2.56)	**5.91E-22**	**9.47E-20**	-1.92	(-2.46--1.39)	**6.41E-12**	**5.13E-11**	-0.48	(-1.25-0.35)	2.45E-01	3.39E-01	-2.41	(-3.83--0.96)	**1.23E-03**	**6.55E-03**
162	GM3(d18:1/24:1)	-1.77	(-2.36--1.17)	**1.05E-08**	**7.23E-08**	-1.28	(-1.87--0.68)	**2.89E-05**	**7.79E-05**	-0.08	(-0.88-0.79)	8.48E-01	8.99E-01	-1.68	(-3.15--0.18)	**2.86E-02**	8.18E-02
163	HexCer(d16:1/22:0)	-1.73	(-2.36--1.09)	**1.69E-07**	**9.90E-07**	0.01	(-0.64-0.67)	9.69E-01	9.73E-01	-0.73	(-1.99-0.73)	3.11E-01	4.09E-01	-0.95	(-2.95-1.08)	3.54E-01	5.07E-01
164	HexCer(d16:1/24:0)	-3.23	(-3.88--2.57)	**2.16E-20**	**2.07E-18**	-0.23	(-0.88-0.42)	4.87E-01	5.45E-01	-0.86	(-1.98-0.41)	1.73E-01	2.55E-01	0.12	(-1.92-2.2)	9.09E-01	9.57E-01
165	HexCer(d18:1/16:0)	-1.32	(-1.8--0.83)	**1.66E-07**	**9.85E-07**	-0.27	(-0.74-0.19)	2.49E-01	3.05E-01	-0.03	(-0.84-0.84)	9.41E-01	9.57E-01	-1.63	(-3.01--0.24)	**2.21E-02**	6.79E-02
166	HexCer(d18:1/18:0)	-1.56	(-2.15--0.97)	**3.24E-07**	**1.80E-06**	-1.40	(-2.04--0.75)	**2.70E-05**	**7.38E-05**	0.29	(-0.73-1.41)	5.96E-01	6.81E-01	-1.86	(-3.59--0.11)	**3.74E-02**	1.00E-01
167	HexCer(d18:1/20:0)	-1.84	(-2.41--1.27)	**4.40E-10**	**5.02E-09**	-1.29	(-1.81--0.76)	**2.18E-06**	**6.88E-06**	0.06	(-0.87-1.08)	9.06E-01	9.31E-01	-2.39	(-4.02--0.73)	**5.05E-03**	**2.10E-02**
168	HexCer(d18:1/22:0)	-1.93	(-2.43--1.44)	**1.05E-13**	**3.87E-12**	-0.92	(-1.41--0.43)	**2.61E-04**	**6.17E-04**	0.50	(-0.26-1.31)	2.01E-01	2.88E-01	-2.35	(-3.8--0.88)	**1.85E-03**	**9.34E-03**
169	HexCer(d18:1/24:0)	-2.60	(-3.1--2.09)	**5.92E-22**	**9.47E-20**	-1.34	(-1.83--0.85)	**1.19E-07**	**4.57E-07**	0.07	(-0.67-0.87)	8.63E-01	9.01E-01	-1.92	(-3.37--0.46)	**1.03E-02**	**3.73E-02**
170	HexCer(d18:1/24:1)	-1.68	(-2.24--1.13)	**5.30E-09**	**4.04E-08**	-1.23	(-1.76--0.69)	**8.47E-06**	**2.48E-05**	0.56	(-0.2-1.39)	1.50E-01	2.28E-01	-1.42	(-2.9-0.08)	6.31E-02	1.48E-01
171	Hex2Cer(d16:1/16:0)	-1.30	(-1.92--0.67)	**5.63E-05**	**1.93E-04**	0.64	(0.03-1.26)	**3.93E-02**	5.84E-02	-1.04	(-2.01-0.05)	6.05E-02	1.04E-01	2.20	(0.06-4.39)	**4.43E-02**	1.14E-01
172	Hex2Cer(d18:1/16:0)	-1.58	(-2.1--1.05)	**6.78E-09**	**5.09E-08**	-1.17	(-1.7--0.63)	**2.30E-05**	**6.34E-05**	-0.53	(-1.21-0.2)	1.48E-01	2.26E-01	-1.45	(-3.16-0.3)	1.03E-01	2.07E-01
173	Hex2Cer(d18:1/22:0)	-1.73	(-2.29--1.17)	**2.30E-09**	**2.05E-08**	-1.43	(-2--0.87)	**8.84E-07**	**2.91E-06**	-0.20	(-1.06-0.73)	6.55E-01	7.37E-01	-2.48	(-4.09--0.83)	**3.43E-03**	**1.57E-02**
174	Hex2Cer(d18:1/24:1)	-1.66	(-2.25--1.07)	**4.96E-08**	**3.13E-07**	-1.91	(-2.5--1.32)	**5.82E-10**	**3.21E-09**	-0.14	(-0.92-0.71)	7.40E-01	8.15E-01	-2.62	(-4.35--0.85)	**3.91E-03**	**1.73E-02**
175	Hex2Cer(d18:2/16:0)	-0.67	(-1.21--0.12)	**1.65E-02**	**2.97E-02**	-0.31	(-0.85-0.22)	2.50E-01	3.06E-01	-0.49	(-1.27-0.35)	2.44E-01	3.38E-01	0.71	(-0.97-2.41)	4.08E-01	5.58E-01
176	Hex3Cer(d18:1/16:0)	-1.04	(-1.45--0.62)	**1.58E-06**	**7.44E-06**	-1.03	(-1.43--0.63)	**6.36E-07**	**2.21E-06**	-0.49	(-1.07-0.13)	1.21E-01	1.90E-01	-0.80	(-1.93-0.33)	1.65E-01	2.97E-01
177	Hex3Cer(d18:1/22:0)	-1.54	(-2.14--0.93)	**9.28E-07**	**4.59E-06**	-2.07	(-2.63--1.51)	**2.59E-12**	**2.22E-11**	0.15	(-0.79-1.19)	7.65E-01	8.33E-01	-1.67	(-3.22--0.11)	**3.65E-02**	9.91E-02
178	Hex3Cer(d18:1/24:0)	-1.57	(-2.15--0.99)	**1.64E-07**	**9.85E-07**	-1.90	(-2.42--1.37)	**6.58E-12**	**5.18E-11**	-0.46	(-1.22-0.37)	2.68E-01	3.62E-01	-2.28	(-3.7--0.83)	**2.21E-03**	**1.09E-02**
179	Hex3Cer(d18:1/24:1)	-1.14	(-1.69--0.58)	**6.61E-05**	**2.23E-04**	-1.42	(-1.92--0.93)	**3.28E-08**	**1.42E-07**	-0.55	(-1.29-0.25)	1.73E-01	2.55E-01	-1.03	(-2.44-0.41)	1.59E-01	2.90E-01
180	SM(d17:1/14:0)	-1.88	(-2.45--1.3)	**3.06E-10**	**3.86E-09**	-0.04	(-0.58-0.51)	8.94E-01	9.17E-01	-0.98	(-1.49--0.44)	**5.42E-04**	**1.36E-03**	2.34	(0.42-4.3)	**1.70E-02**	5.51E-02
181	SM(d18:0/14:0)	-0.89	(-1.42--0.36)	**1.08E-03**	**2.70E-03**	0.73	(0.18-1.28)	**9.62E-03**	**1.64E-02**	-0.26	(-0.77-0.28)	3.36E-01	4.34E-01	1.63	(0.05-3.24)	**4.26E-02**	1.10E-01
182	SM(d18:1/14:0)/SM(d16:1/16:0)	-1.03	(-1.5--0.55)	**2.66E-05**	**9.76E-05**	0.72	(0.27-1.18)	**1.93E-03**	**3.86E-03**	-0.56	(-1.05--0.03)	**3.86E-02**	6.97E-02	2.11	(0.57-3.68)	**7.37E-03**	**2.85E-02**
183	SM(d18:2/14:0)	0.18	(-0.33-0.69)	4.96E-01	5.50E-01	1.49	(1.01-1.98)	**2.12E-09**	**1.07E-08**	0.07	(-0.46-0.63)	7.91E-01	8.51E-01	6.29	(4.83-7.77)	**2.07E-16**	**9.93E-14**
184	SM(d17:1/16:0)	-1.40	(-1.85--0.95)	**2.57E-09**	**2.25E-08**	-0.22	(-0.65-0.22)	3.29E-01	3.84E-01	-0.98	(-1.47--0.47)	**2.86E-04**	**7.59E-04**	-0.38	(-1.9-1.16)	6.27E-01	7.41E-01
185	SM(d18:1/16:0)	-1.46	(-1.99--0.92)	**1.89E-07**	**1.08E-06**	-0.89	(-1.41--0.37)	**9.07E-04**	**1.95E-03**	-0.34	(-0.91-0.28)	2.74E-01	3.66E-01	-1.60	(-3.02--0.16)	**3.01E-02**	8.45E-02

186	SM(d18:2/16:0)	0.08	(-0.32-0.48)	7.06E-01	7.48E-01	0.65	(0.28-1.02)	**5.74E-04**	**1.26E-03**	0.06	(-0.44-0.6)	8.06E-01	8.64E-01	1.21	(0.14-2.29)	**2.69E-02**	7.82E-02
187	SM(34:3)	0.20	(-0.28-0.68)	4.10E-01	4.70E-01	0.97	(0.49-1.46)	**8.92E-05**	**2.27E-04**	0.13	(-0.42-0.72)	6.47E-01	7.30E-01	2.92	(1.38-4.49)	**2.12E-04**	**1.70E-03**
188	SM(d18:2/17:0)	-0.37	(-0.83-0.1)	1.23E-01	1.70E-01	0.32	(-0.1-0.74)	1.30E-01	1.71E-01	-0.91	(-1.41--0.38)	**9.15E-04**	**2.25E-03**	1.15	(-0.24-2.56)	1.04E-01	2.08E-01
189	SM(35:2) (b)	-1.53	(-2.12--0.94)	**4.86E-07**	**2.56E-06**	-0.79	(-1.37--0.2)	**8.48E-03**	**1.46E-02**	-0.68	(-1.39-0.1)	8.62E-02	1.42E-01	-1.19	(-2.94-0.58)	1.85E-01	3.18E-01
190	SM(d18:1/18:0)/SM(d16:1/20:0)	0.25	(-0.26-0.76)	3.41E-01	4.06E-01	0.68	(0.2-1.16)	**5.56E-03**	**1.00E-02**	-0.05	(-0.63-0.56)	8.71E-01	9.06E-01	0.38	(-1.02-1.81)	5.93E-01	7.19E-01
191	SM(d18:2/18:1)	0.37	(-0.11-0.84)	1.30E-01	1.79E-01	0.35	(-0.07-0.77)	1.01E-01	1.36E-01	-0.01	(-0.59-0.61)	9.77E-01	9.79E-01	1.30	(0.08-2.53)	**3.65E-02**	9.91E-02
192	SM(37:1)	-0.75	(-1.3--0.2)	**7.20E-03**	**1.46E-02**	-0.09	(-0.6-0.41)	7.20E-01	7.61E-01	-0.82	(-1.45--0.15)	**1.76E-02**	**3.42E-02**	0.13	(-1.47-1.76)	8.71E-01	9.29E-01
193	SM(37:2)	-1.21	(-1.81--0.6)	**1.07E-04**	**3.46E-04**	-1.13	(-1.68--0.57)	**7.90E-05**	**2.02E-04**	-0.76	(-1.35--0.12)	**2.06E-02**	**3.93E-02**	-0.55	(-2.23-1.16)	5.25E-01	6.51E-01
194	SM(d18:1/20:0)/SM(d16:1/22:0)	-0.75	(-1.17--0.33)	**4.82E-04**	**1.32E-03**	0.43	(0.04-0.83)	**2.93E-02**	**4.47E-02**	0.28	(-0.27-0.85)	3.27E-01	4.25E-01	0.57	(-0.67-1.83)	3.67E-01	5.21E-01
195	SM(d18:2/20:0)	-0.36	(-0.86-0.14)	1.60E-01	2.13E-01	0.11	(-0.41-0.62)	6.89E-01	7.33E-01	0.31	(-0.42-1.1)	4.15E-01	5.15E-01	0.44	(-1.07-1.98)	5.66E-01	6.94E-01
196	SM(38:3) (a)	0.49	(0-0.98)	**4.88E-02**	7.63E-02	0.56	(0.14-0.98)	**8.55E-03**	**1.47E-02**	-0.52	(-1.05-0.06)	7.63E-02	1.28E-01	1.93	(0.68-3.2)	**2.56E-03**	**1.24E-02**
197	SM(38:3) (b)	-0.77	(-1.33--0.22)	**6.71E-03**	**1.37E-02**	-0.67	(-1.28--0.07)	**3.00E-02**	**4.55E-02**	-0.07	(-0.77-0.69)	8.53E-01	9.00E-01	0.47	(-1.3-2.27)	6.02E-01	7.24E-01
198	SM(d16:1/23:0)/SM(d17:1/22:0)	-1.08	(-1.55--0.62)	**6.16E-06**	**2.62E-05**	0.38	(-0.05-0.81)	8.35E-02	1.15E-01	-0.37	(-0.89-0.18)	1.79E-01	2.62E-01	1.38	(-0.17-2.96)	8.16E-02	1.74E-01
199	SM(d18:1/22:0)/SM(d16:1/24:0)	-0.59	(-1.06--0.13)	**1.28E-02**	**2.38E-02**	0.71	(0.26-1.16)	**2.08E-03**	**4.13E-03**	0.40	(-0.17-1)	1.76E-01	2.58E-01	0.43	(-0.84-1.71)	5.12E-01	6.45E-01
200	SM(d16:1/24:1)	-0.70	(-1.21--0.19)	**7.53E-03**	**1.51E-02**	0.55	(0.05-1.05)	**3.15E-02**	**4.75E-02**	-0.12	(-0.71-0.51)	7.00E-01	7.76E-01	2.62	(1.12-4.15)	**6.11E-04**	**3.91E-03**
201	SM(d18:2/22:0)	-0.79	(-1.25--0.33)	**7.92E-04**	**2.04E-03**	0.01	(-0.41-0.43)	9.67E-01	9.73E-01	0.38	(-0.22-1.02)	2.17E-01	3.06E-01	0.42	(-0.78-1.64)	4.91E-01	6.31E-01
202	SM(40:3) (a)	-0.13	(-0.62-0.36)	5.98E-01	6.48E-01	0.29	(-0.19-0.77)	2.39E-01	2.94E-01	-0.44	(-1.01-0.17)	1.54E-01	2.32E-01	1.68	(0.3-3.08)	**1.72E-02**	5.52E-02
203	SM(40:3) (b)	-0.91	(-1.46--0.36)	**1.23E-03**	**3.06E-03**	-0.26	(-0.79-0.27)	3.35E-01	3.91E-01	-0.89	(-1.5--0.24)	**8.17E-03**	**1.72E-02**	0.93	(-0.86-2.74)	3.11E-01	4.70E-01
204	SM(41:0)	-0.42	(-0.85-0.01)	5.33E-02	8.22E-02	0.74	(0.28-1.19)	**1.45E-03**	**2.96E-03**	-0.51	(-1.39-0.45)	2.86E-01	3.79E-01	0.37	(-0.9-1.65)	5.73E-01	7.00E-01
205	SM(41:1)	-0.70	(-1.12--0.28)	**1.15E-03**	**2.85E-03**	0.39	(-0.02-0.81)	6.15E-02	8.66E-02	-0.21	(-0.85-0.47)	5.36E-01	6.25E-01	0.49	(-0.79-1.78)	4.55E-01	6.00E-01
206	SM(d17:1/24:1)	-0.87	(-1.32--0.41)	**2.01E-04**	**6.11E-04**	-0.13	(-0.57-0.31)	5.57E-01	6.17E-01	-0.47	(-1.01-0.1)	1.02E-01	1.63E-01	0.60	(-0.74-1.96)	3.83E-01	5.29E-01
207	SM(d18:2/23:0)	-0.93	(-1.36--0.5)	**2.33E-05**	**8.74E-05**	-0.51	(-0.9--0.11)	**1.15E-02**	**1.91E-02**	-0.01	(-0.68-0.7)	9.74E-01	9.79E-01	0.72	(-0.51-1.96)	2.51E-01	4.04E-01
208	SM(d18:1/24:0)	-1.46	(-1.92--1.01)	**6.70E-10**	**7.31E-09**	0.04	(-0.38-0.47)	8.44E-01	8.73E-01	-0.25	(-0.81-0.35)	4.12E-01	5.14E-01	-0.50	(-1.63-0.63)	3.83E-01	5.29E-01
209	SM(d18:1/24:1)	-0.55	(-1.08--0.01)	**4.44E-02**	7.06E-02	-0.10	(-0.61-0.42)	7.12E-01	7.55E-01	0.21	(-0.37-0.84)	4.83E-01	5.80E-01	-0.12	(-1.44-1.22)	8.57E-01	9.18E-01
210	SM(d18:2/24:0)	-1.48	(-1.89--1.07)	**6.63E-12**	**1.45E-10**	-0.85	(-1.23--0.46)	**1.89E-05**	**5.27E-05**	-0.11	(-0.67-0.48)	7.00E-01	7.76E-01	-0.49	(-1.53-0.56)	3.54E-01	5.07E-01
211	SM(43:1)	-1.62	(-2.09--1.14)	**7.20E-11**	**1.19E-09**	-0.14	(-0.59-0.31)	5.37E-01	5.96E-01	-1.04	(-1.74--0.27)	**9.36E-03**	**1.94E-02**	-1.37	(-2.82-0.1)	6.74E-02	1.53E-01
212	SM(44:1)	-1.59	(-2.01--1.17)	**4.74E-13**	**1.42E-11**	-0.38	(-0.76-0.01)	5.52E-02	7.89E-02	-0.49	(-1-0.04)	7.11E-02	1.21E-01	-2.17	(-3.24--1.08)	**1.19E-04**	**1.17E-03**
213	SM(44:2)	-1.13	(-1.63--0.63)	**1.09E-05**	**4.45E-05**	-0.45	(-0.9-0.01)	5.39E-02	7.74E-02	-0.23	(-0.75-0.32)	4.07E-01	5.08E-01	-1.24	(-2.48-0.02)	5.39E-02	1.31E-01
214	SM(44:3) (a)	-1.42	(-1.88--0.95)	**4.02E-09**	**3.21E-08**	-1.26	(-1.69--0.84)	**1.15E-08**	**5.25E-08**	-0.41	(-0.9-0.11)	1.18E-01	1.85E-01	-0.55	(-1.66-0.56)	3.30E-01	4.88E-01
215	LPC(14:0) [sn2]	-0.72	(-1.5-0.06)	6.98E-02	1.02E-01	1.18	(0.41-1.96)	**2.81E-03**	**5.40E-03**	1.75	(1.25-2.26)	**6.35E-13**	**4.36E-12**	4.86	(2.45-7.33)	**7.65E-05**	**8.95E-04**
216	LPC(14:0) [sn1]	-0.42	(-1.17-0.34)	2.76E-01	3.40E-01	1.53	(0.78-2.3)	**7.13E-05**	**1.84E-04**	1.62	(1.14-2.11)	**5.17E-12**	**3.06E-11**	4.70	(2.35-7.09)	**8.28E-05**	**9.46E-04**
217	LPC(15:0) [sn2]	-1.50	(-2.11--0.88)	**2.82E-06**	**1.29E-05**	-0.45	(-1.04-0.14)	1.37E-01	1.79E-01	1.04	(0.46-1.66)	**3.62E-04**	**9.45E-04**	1.83	(-0.4-4.11)	1.09E-01	2.13E-01
218	LPC(15:0) [sn1]	-1.28	(-1.83--0.72)	**8.57E-06**	**3.58E-05**	-0.43	(-0.97-0.12)	1.27E-01	1.67E-01	0.76	(0.24-1.32)	**4.17E-03**	**9.24E-03**	1.40	(-0.68-3.52)	1.87E-01	3.18E-01
219	LPC(16:0) [sn2]	-0.01	(-0.46-0.43)	9.50E-01	9.56E-01	-0.61	(-1.03--0.19)	**4.76E-03**	**8.73E-03**	1.73	(1.26-2.21)	**7.55E-14**	**5.75E-13**	0.68	(-0.95-2.34)	4.13E-01	5.58E-01
220	LPC(16:0) [sn1]	0.26	(-0.27-0.8)	3.31E-01	3.96E-01	-0.69	(-1.19--0.19)	**7.24E-03**	**1.26E-02**	1.95	(1.38-2.55)	**2.32E-12**	**1.53E-11**	0.54	(-1.35-2.47)	5.78E-01	7.05E-01
221	LPC(16:1) [sn2]	0.17	(-0.55-0.9)	6.35E-01	6.82E-01	0.95	(0.37-1.53)	**1.29E-03**	**2.69E-03**	4.00	(3.27-4.78)	**3.37E-31**	**8.10E-29**	5.09	(3.19-7.03)	**1.66E-07**	**1.14E-05**
222	LPC(16:1) [sn1]	0.34	(-0.29-0.97)	2.93E-01	3.56E-01	0.87	(0.34-1.4)	**1.33E-03**	**2.76E-03**	3.89	(3.2-4.61)	**6.09E-33**	**2.92E-30**	3.93	(2.1-5.79)	**2.67E-05**	**4.62E-04**
223	LPC(15-MHDA) [sn2]	-1.34	(-2.09--0.59)	**5.17E-04**	**1.39E-03**	-1.80	(-2.53--1.05)	**2.97E-06**	**9.21E-06**	0.88	(0-1.85)	5.11E-02	9.02E-02	-0.29	(-2.99-2.49)	8.37E-01	8.99E-01
224	LPC(15-MHDA) [sn1] / LPC(17:0) [sn2]	-1.22	(-1.86--0.59)	**1.83E-04**	**5.61E-04**	-1.69	(-2.31--1.07)	**1.33E-07**	**5.00E-07**	0.53	(-0.15-1.25)	1.30E-01	2.01E-01	-0.87	(-3.13-1.44)	4.56E-01	6.00E-01
225	LPC(17:0) [sn1]	-0.89	(-1.39--0.39)	**5.04E-04**	**1.36E-03**	-1.40	(-1.9--0.89)	**8.98E-08**	**3.53E-07**	0.17	(-0.3-0.67)	4.88E-01	5.80E-01	-0.97	(-2.82-0.91)	3.09E-01	4.67E-01
226	LPC(17:1) [sn1] (a) / LPC(17:1) [sn2] (b)	-0.95	(-1.52--0.37)	**1.28E-03**	**3.14E-03**	-0.68	(-1.27--0.08)	**2.64E-02**	**4.06E-02**	1.72	(1.09-2.4)	**3.10E-08**	**1.29E-07**	0.85	(-1.18-2.93)	4.14E-01	5.58E-01
227	LPC(18:0) [sn2]	-0.05	(-0.52-0.42)	8.27E-01	8.53E-01	-0.52	(-1--0.04)	**3.29E-02**	**4.94E-02**	0.99	(0.53-1.46)	**1.70E-05**	**5.36E-05**	0.72	(-1.04-2.51)	4.23E-01	5.69E-01
228	LPC(18:0) [sn1]	-0.03	(-0.51-0.44)	8.92E-01	9.08E-01	-0.55	(-1.05--0.05)	**3.14E-02**	**4.75E-02**	0.99	(0.52-1.48)	**2.53E-05**	**7.78E-05**	0.77	(-1.17-2.74)	4.39E-01	5.82E-01
229	LPC(18:1) [sn2]	-0.59	(-1.11--0.07)	**2.69E-02**	**4.57E-02**	-1.43	(-2--0.86)	**1.28E-06**	**4.15E-06**	3.61	(2.91-4.34)	**4.61E-28**	**5.53E-26**	-0.89	(-2.69-0.94)	3.36E-01	4.95E-01
230	LPC(18:1) [sn1]	-0.50	(-0.97--0.04)	**3.47E-02**	5.77E-02	-1.38	(-1.88--0.87)	**1.29E-07**	**4.88E-07**	3.36	(2.74-4.01)	**4.86E-30**	**7.77E-28**	-1.16	(-2.75-0.46)	1.60E-01	2.91E-01
231	LPC(18:2) [sn2]	-0.64	(-1.13--0.15)	**1.04E-02**	**1.97E-02**	-2.17	(-2.7--1.63)	**2.57E-14**	**3.16E-13**	2.93	(2.22-3.69)	**4.36E-18**	**6.75E-17**	-1.10	(-2.7-0.54)	1.86E-01	3.18E-01
232	LPC(18:2) [sn1]	-0.41	(-0.96-0.14)	1.45E-01	1.96E-01	-2.32	(-2.91--1.74)	**5.71E-14**	**6.85E-13**	2.94	(2.22-3.7)	**9.01E-18**	**1.35E-16**	-1.59	(-3.34-0.19)	8.00E-02	1.73E-01
233	LPC(18:3) [sn2] (a)	-1.54	(-2.23--0.85)	**1.68E-05**	**6.51E-05**	-0.86	(-1.59--0.12)	**2.29E-02**	**3.56E-02**	2.31	(1.65-3.01)	**5.03E-13**	**3.55E-12**	0.51	(-1.64-2.71)	6.43E-01	7.48E-01

234	LPC(18:3) [sn1] (a)/LPC(18:3) [sn2] (b)	-0.95	(-1.67--0.23)	**9.74E-03**	**1.88E-02**	-0.22	(-0.97-0.54)	5.67E-01	6.27E-01	4.43	(3.51-5.42)	**1.76E-25**	**1.41E-23**	1.18	(-1.15-3.57)	3.23E-01	4.84E-01
235	LPC(18:3) (a) [sn1] [104_sn1]	-0.94	(-1.68--0.2)	**1.34E-02**	**2.43E-02**	-0.40	(-1.16-0.36)	3.02E-01	3.59E-01	4.09	(3.21-5.02)	**1.27E-23**	**6.77E-22**	1.30	(-1.04-3.69)	2.77E-01	4.39E-01
236	LPC(19:0) [sn1] (a) / LPC(19:0) [sn2] (b)	-1.56	(-2.02--1.09)	**1.61E-10**	**2.49E-09**	-2.28	(-2.79--1.76)	**6.69E-17**	**1.61E-15**	-0.24	(-0.87-0.42)	4.64E-01	5.64E-01	-2.26	(-3.96--0.53)	**1.08E-02**	**3.85E-02**
237	LPC(20:0) [sn1]	-1.59	(-2.03--1.14)	**1.26E-11**	**2.64E-10**	-2.10	(-2.58--1.62)	**1.05E-16**	**2.41E-15**	0.30	(-0.12-0.74)	1.68E-01	2.51E-01	-3.35	(-4.85--1.82)	**2.48E-05**	**4.62E-04**
238	LPC(20:1) [sn2]	-0.60	(-1.07--0.14)	**1.07E-02**	**2.02E-02**	-1.58	(-2.1--1.06)	**5.88E-09**	**2.79E-08**	1.44	(0.92-1.98)	**1.54E-08**	**6.60E-08**	-1.99	(-3.63--0.33)	**1.93E-02**	6.09E-02
239	LPC(20:1) [sn1]	-0.62	(-1.08--0.15)	**9.49E-03**	**1.84E-02**	-1.86	(-2.38--1.33)	**1.33E-11**	**9.82E-11**	1.75	(1.27-2.25)	**1.55E-13**	**1.12E-12**	-2.32	(-3.93--0.7)	**5.46E-03**	**2.19E-02**
240	LPC(20:2) [sn2]	-0.91	(-1.41--0.4)	**4.81E-04**	**1.32E-03**	-0.80	(-1.3--0.31)	**1.50E-03**	**3.03E-03**	3.83	(3.05-4.65)	**3.73E-26**	**3.58E-24**	-0.47	(-2.07-1.15)	5.65E-01	6.94E-01
241	LPC(20:2) [sn1]	-0.63	(-1.11--0.16)	**9.14E-03**	**1.78E-02**	-0.94	(-1.4--0.46)	**1.11E-04**	**2.77E-04**	3.35	(2.66-4.06)	**4.61E-25**	**3.16E-23**	-0.77	(-2.38-0.86)	3.50E-01	5.06E-01
242	LPC(20:3) [sn2]	1.05	(0.4-1.7)	**1.48E-03**	**3.59E-03**	1.19	(0.56-1.83)	**2.39E-04**	**5.67E-04**	2.58	(1.91-3.3)	**1.79E-15**	**1.72E-14**	2.63	(0.79-4.5)	**4.98E-03**	**2.10E-02**
243	LPC(20:3) [sn1]	1.28	(0.59-1.97)	**2.78E-04**	**8.08E-04**	0.91	(0.26-1.56)	**5.99E-03**	**1.06E-02**	3.27	(2.53-4.05)	**7.83E-21**	**2.50E-19**	2.43	(0.45-4.44)	**1.61E-02**	5.28E-02
244	LPC(20:3) [104_sn1]	1.42	(0.72-2.12)	**7.46E-05**	**2.47E-04**	0.93	(0.28-1.58)	**4.92E-03**	**8.95E-03**	3.22	(2.49-3.99)	**9.49E-21**	**2.85E-19**	2.53	(0.51-4.6)	**1.42E-02**	**4.91E-02**
245	LPC(20:4) [sn2]	0.85	(0.33-1.37)	**1.45E-03**	**3.53E-03**	-0.79	(-1.3--0.28)	**2.64E-03**	**5.13E-03**	2.39	(1.75-3.06)	**6.31E-15**	**5.61E-14**	1.51	(-0.14-3.19)	7.23E-02	1.59E-01
246	LPC(20:4) [sn1]	1.36	(0.79-1.93)	**3.16E-06**	**1.42E-05**	-1.16	(-1.71--0.61)	**4.15E-05**	**1.10E-04**	2.66	(1.98-3.38)	**2.33E-16**	**2.60E-15**	1.04	(-0.76-2.87)	2.58E-01	4.13E-01
247	LPC(20:5) [sn2]	-0.04	(-1.13-1.06)	9.43E-01	9.53E-01	-0.23	(-1.33-0.88)	6.78E-01	7.23E-01	0.45	(-0.63-1.66)	4.27E-01	5.27E-01	4.34	(1.51-7.25)	**2.58E-03**	**1.24E-02**
248	LPC(20:5) [sn1]	0.24	(-0.88-1.36)	6.78E-01	7.25E-01	-0.27	(-1.38-0.86)	6.41E-01	6.91E-01	0.62	(-0.47-1.83)	2.74E-01	3.66E-01	4.41	(1.48-7.43)	**3.09E-03**	**1.43E-02**
249	LPC(22:4) [sn2]	-0.46	(-1.09-0.17)	1.50E-01	2.02E-01	-0.27	(-0.86-0.32)	3.74E-01	4.29E-01	2.50	(1.81-3.24)	**7.33E-14**	**5.68E-13**	-0.12	(-1.97-1.77)	8.99E-01	9.51E-01
250	LPC(22:4) [sn1]	0.02	(-0.59-0.64)	9.45E-01	9.53E-01	-0.55	(-1.14-0.05)	7.14E-02	9.97E-02	2.70	(2.04-3.39)	**9.68E-18**	**1.37E-16**	0.72	(-1.19-2.67)	4.63E-01	6.06E-01
251	LPC(22:5) [sn2] (n3)	-0.98	(-1.6--0.35)	**2.21E-03**	**5.12E-03**	-0.74	(-1.34--0.14)	**1.67E-02**	**2.68E-02**	1.73	(0.89-2.63)	**2.63E-05**	**8.04E-05**	0.81	(-0.85-2.5)	3.39E-01	4.95E-01
252	LPC(22:5) [sn1] (n3)/LPC(22:5) [sn2] (n6)	-0.84	(-1.42--0.26)	**4.63E-03**	**9.97E-03**	-0.82	(-1.43--0.21)	**8.19E-03**	**1.42E-02**	2.12	(1.41-2.88)	**6.79E-10**	**3.29E-09**	0.43	(-1.27-2.16)	6.21E-01	7.41E-01
253	LPC(22:5) [sn1] (n6)	-0.53	(-1.3-0.25)	1.80E-01	2.35E-01	-1.14	(-1.82--0.47)	**9.90E-04**	**2.10E-03**	4.48	(3.33-5.74)	**1.81E-17**	**2.48E-16**	-0.41	(-2.62-1.85)	7.17E-01	7.98E-01
254	LPC(22:5) (n3) [sn1] [104_sn1]	-0.74	(-1.42--0.05)	**3.52E-02**	5.83E-02	-0.95	(-1.59--0.31)	**4.00E-03**	**7.50E-03**	1.03	(0.27-1.84)	**7.20E-03**	**1.54E-02**	0.80	(-0.97-2.6)	3.79E-01	5.29E-01
255	LPC(22:6) [sn2]	-0.66	(-1.28--0.03)	**3.88E-02**	6.33E-02	-1.09	(-1.7--0.47)	**5.66E-04**	**1.25E-03**	1.62	(0.94-2.33)	**1.13E-06**	**3.92E-06**	0.47	(-1.44-2.43)	6.30E-01	7.41E-01
256	LPC(22:6) [sn1]	-0.13	(-0.79-0.53)	6.95E-01	7.40E-01	-1.17	(-1.8--0.53)	**3.39E-04**	**7.95E-04**	2.08	(1.36-2.84)	**2.30E-09**	**1.04E-08**	0.38	(-1.62-2.43)	7.09E-01	7.93E-01
257	LPC(O-16:0)	-0.32	(-0.76-0.13)	1.63E-01	2.17E-01	-0.94	(-1.4--0.47)	**9.91E-05**	**2.49E-04**	1.73	(1.23-2.26)	**3.01E-12**	**1.87E-11**	0.40	(-1.39-2.22)	6.66E-01	7.58E-01
258	LPC(O-18:0)	-1.05	(-1.59--0.5)	**1.83E-04**	**5.61E-04**	-1.53	(-2.07--0.99)	**5.02E-08**	**2.13E-07**	0.28	(-0.28-0.88)	3.35E-01	4.33E-01	-1.09	(-3.06-0.93)	2.87E-01	4.45E-01
259	LPC(O-18:1)	0.52	(0.07-0.97)	**2.38E-02**	**4.13E-02**	-0.80	(-1.25--0.35)	**5.53E-04**	**1.23E-03**	1.82	(1.3-2.37)	**7.59E-13**	**5.13E-12**	-0.28	(-2.05-1.54)	7.63E-01	8.42E-01
260	LPC(O-20:0)	-1.70	(-2.21--1.19)	**1.86E-10**	**2.70E-09**	-2.23	(-2.77--1.69)	**4.19E-15**	**6.29E-14**	0.04	(-0.78-0.93)	9.25E-01	9.42E-01	-2.32	(-4.14--0.47)	**1.46E-02**	**4.96E-02**
261	LPC(O-20:1)	0.35	(-0.5-1.21)	4.19E-01	4.80E-01	-0.87	(-1.46--0.26)	**4.90E-03**	**8.94E-03**	2.68	(1.69-3.77)	**1.85E-08**	**7.79E-08**	0.12	(-2.19-2.48)	9.22E-01	9.64E-01
262	LPC(O-22:0)	-2.40	(-2.88--1.91)	**1.69E-20**	**2.02E-18**	-1.43	(-1.85--1)	**1.03E-10**	**6.79E-10**	0.22	(-0.26-0.72)	3.73E-01	4.73E-01	-1.92	(-3.1--0.72)	**1.79E-03**	**9.15E-03**
263	LPC(O-22:1)	-1.62	(-2.15--1.1)	**2.73E-09**	**2.33E-08**	-2.10	(-2.63--1.56)	**9.90E-14**	**1.16E-12**	1.29	(0.72-1.9)	**6.07E-06**	**1.97E-05**	-1.92	(-3.6--0.2)	**2.86E-02**	8.18E-02
264	LPC(O-24:0)	-3.04	(-3.62--2.45)	**1.56E-22**	**7.48E-20**	-1.17	(-1.62--0.71)	**7.24E-07**	**2.45E-06**	-0.48	(-0.98-0.06)	7.85E-02	1.31E-01	-1.49	(-2.7--0.26)	**1.81E-02**	5.76E-02
265	LPC(O-24:1)	-2.43	(-2.98--1.87)	**1.19E-16**	**8.16E-15**	-1.37	(-1.84--0.9)	**1.75E-08**	**7.86E-08**	0.47	(-0.04-1.01)	7.26E-02	1.23E-01	-0.57	(-1.98-0.86)	4.30E-01	5.74E-01
266	LPC(O-24:2)	-1.94	(-2.53--1.34)	**3.86E-10**	**4.64E-09**	-2.07	(-2.6--1.53)	**1.68E-13**	**1.79E-12**	0.20	(-0.32-0.75)	4.61E-01	5.61E-01	-1.62	(-3.28-0.07)	6.00E-02	1.43E-01
267	LPC(P-16:0)	0.43	(-0.05-0.9)	7.81E-02	1.12E-01	-1.04	(-1.51--0.56)	**2.15E-05**	**5.96E-05**	1.13	(0.65-1.63)	**2.63E-06**	**8.83E-06**	0.40	(-1.33-2.16)	6.53E-01	7.51E-01
268	LPC(P-18:0)	0.41	(-0.04-0.87)	7.48E-02	1.09E-01	-0.79	(-1.24--0.34)	**6.48E-04**	**1.41E-03**	1.76	(1.24-2.29)	**2.96E-12**	**1.87E-11**	-0.43	(-2.19-1.36)	6.34E-01	7.42E-01
269	LPC(P-18:1)	0.95	(0.36-1.54)	**1.57E-03**	**3.78E-03**	-0.88	(-1.4--0.35)	**1.14E-03**	**2.40E-03**	1.25	(0.59-1.95)	**1.32E-04**	**3.64E-04**	0.83	(-1.2-2.9)	4.25E-01	5.70E-01
270	LPC(P-20:0)	-0.73	(-1.3--0.15)	**1.34E-02**	**2.43E-02**	-1.56	(-2.13--0.99)	**1.23E-07**	**4.68E-07**	2.02	(1.36-2.71)	**3.08E-10**	**1.54E-09**	-1.83	(-3.76-0.15)	6.94E-02	1.56E-01
271	LPE(16:0) [sn2]	-0.58	(-1.09--0.08)	**2.45E-02**	**4.23E-02**	-1.40	(-1.94--0.85)	**8.49E-07**	**2.81E-06**	2.24	(1.6-2.92)	**6.15E-13**	**4.28E-12**	-1.78	(-3.44--0.09)	**3.95E-02**	1.05E-01
272	LPE(16:0) [sn1]	-0.47	(-0.95-0.01)	5.74E-02	8.74E-02	-1.36	(-1.89--0.84)	**4.93E-07**	**1.73E-06**	2.24	(1.64-2.88)	**1.97E-14**	**1.60E-13**	-1.41	(-2.97-0.19)	8.30E-02	1.76E-01
273	LPE(18:0) [sn2]	0.18	(-0.3-0.66)	4.70E-01	5.28E-01	-0.80	(-1.27--0.33)	**9.36E-04**	**2.01E-03**	1.40	(0.87-1.96)	**9.59E-08**	**3.71E-07**	-1.19	(-2.65-0.28)	1.12E-01	2.14E-01
274	LPE(18:0) [sn1]	0.40	(-0.05-0.85)	7.97E-02	1.14E-01	-0.85	(-1.33--0.37)	**5.55E-04**	**1.23E-03**	1.56	(1.03-2.12)	**2.13E-09**	**9.83E-09**	-0.83	(-2.34-0.7)	2.86E-01	4.45E-01
275	LPE(18:1) [sn2]	-0.78	(-1.33--0.24)	**5.15E-03**	**1.10E-02**	-0.57	(-1.23-0.09)	8.95E-02	1.22E-01	2.54	(1.88-3.24)	**1.28E-15**	**1.28E-14**	-1.28	(-3.21-0.69)	2.01E-01	3.37E-01
276	LPE(18:1) [sn1]	-0.74	(-1.28--0.2)	**7.22E-03**	**1.46E-02**	-0.63	(-1.3-0.04)	6.44E-02	9.03E-02	2.94	(2.26-3.65)	**1.02E-19**	**2.33E-18**	-0.98	(-2.95-1.04)	3.39E-01	4.95E-01
277	LPE(18:2) [sn2]	-0.93	(-1.59--0.27)	**6.21E-03**	**1.29E-02**	-0.58	(-1.27-0.12)	1.03E-01	1.38E-01	3.65	(2.71-4.66)	**9.44E-17**	**1.10E-15**	-1.63	(-3.55-0.33)	1.03E-01	2.07E-01
278	LPE(18:2) [sn1]	-0.96	(-1.63--0.29)	**5.28E-03**	**1.12E-02**	-0.59	(-1.27-0.09)	8.83E-02	1.21E-01	3.74	(2.85-4.69)	**1.99E-19**	**4.16E-18**	-1.59	(-3.53-0.38)	1.12E-01	2.14E-01
279	LPE(20:4) [sn1]	0.57	(0.03-1.11)	**3.84E-02**	6.29E-02	-0.63	(-1.14--0.11)	**1.73E-02**	**2.76E-02**	3.15	(2.41-3.95)	**4.99E-19**	**9.21E-18**	-1.23	(-2.61-0.18)	8.67E-02	1.82E-01
280	LPE(22:6) [sn2]	-0.37	(-0.96-0.22)	2.19E-01	2.77E-01	-0.37	(-0.97-0.22)	2.16E-01	2.73E-01	2.12	(1.4-2.88)	**9.96E-10**	**4.73E-09**	-0.60	(-2.39-1.23)	5.19E-01	6.50E-01
281	LPE(22:6) [sn1]	-0.32	(-0.95-0.31)	3.16E-01	3.83E-01	-0.51	(-1.08-0.06)	8.10E-02	1.12E-01	2.35	(1.63-3.11)	**9.70E-12**	**5.68E-11**	-0.45	(-2.17-1.29)	6.09E-01	7.29E-01

282	PC(28:0)	-2.72	(-3.8--1.63)	**1.51E-06**	**7.19E-06**	0.17	(-0.93-1.29)	7.62E-01	8.00E-01	-0.29	(-0.88-0.34)	3.63E-01	4.65E-01	2.70	(-0.18-5.66)	6.63E-02	1.52E-01
283	PC(14:0_16:0)	-2.18	(-2.89--1.47)	**4.19E-09**	**3.29E-08**	0.08	(-0.63-0.79)	8.31E-01	8.63E-01	-0.19	(-0.85-0.52)	5.89E-01	6.74E-01	0.35	(-1.54-2.28)	7.18E-01	7.98E-01
284	PC(31:0) (a)	-3.27	(-4.32--2.21)	**3.45E-09**	**2.85E-08**	0.93	(-0.13-2)	8.46E-02	1.16E-01	-0.55	(-1.4-0.4)	2.47E-01	3.40E-01	1.80	(-1.79-5.52)	3.29E-01	4.88E-01
285	PC(31:0) (b)	-2.25	(-2.86--1.64)	**2.46E-12**	**6.57E-11**	-0.26	(-0.91-0.39)	4.28E-01	4.87E-01	-1.00	(-1.85--0.06)	**3.79E-02**	6.87E-02	-0.24	(-2.42-1.99)	8.32E-01	8.97E-01
286	PC(31:1)	-2.56	(-3.45--1.67)	**3.05E-08**	**2.06E-07**	1.45	(0.56-2.34)	**1.35E-03**	**2.79E-03**	-0.29	(-1.06-0.54)	4.80E-01	5.78E-01	3.29	(0.61-6.04)	**1.58E-02**	5.23E-02
287	PC(16:0_16:0)	-0.68	(-1.18--0.17)	**8.41E-03**	**1.65E-02**	-0.23	(-0.72-0.26)	3.50E-01	4.04E-01	-0.57	(-1.19-0.09)	9.09E-02	1.49E-01	-1.29	(-2.54--0.03)	**4.48E-02**	1.14E-01
288	PC(32:1)	-0.87	(-1.69--0.04)	**4.11E-02**	6.65E-02	2.58	(1.77-3.39)	**4.13E-10**	**2.39E-09**	0.94	(0.11-1.83)	**2.49E-02**	**4.70E-02**	3.56	(1.41-5.75)	**1.12E-03**	**6.20E-03**
289	PC(32:2)	-1.59	(-2.26--0.92)	**4.67E-06**	**2.00E-05**	0.85	(0.03-1.67)	**4.18E-02**	6.19E-02	0.08	(-0.57-0.78)	8.09E-01	8.65E-01	2.57	(0.47-4.71)	**1.64E-02**	5.36E-02
290	PC(33:0) (a)	-2.28	(-3.03--1.52)	**7.38E-09**	**5.29E-08**	0.04	(-0.76-0.84)	9.31E-01	9.49E-01	-0.79	(-1.65-0.15)	9.71E-02	1.58E-01	0.06	(-2.65-2.83)	9.68E-01	9.86E-01
291	PC(33:0) (b)	-1.78	(-2.34--1.22)	**1.18E-09**	**1.18E-08**	-0.47	(-1.06-0.13)	1.25E-01	1.64E-01	-1.45	(-2.17--0.66)	**5.09E-04**	**1.30E-03**	-1.02	(-2.85-0.84)	2.79E-01	4.39E-01
292	PC(33:1)	-2.44	(-3.14--1.73)	**5.10E-11**	**8.75E-10**	0.47	(-0.25-1.2)	2.03E-01	2.57E-01	-0.58	(-1.18-0.07)	7.75E-02	1.30E-01	0.03	(-2.09-2.19)	9.82E-01	9.92E-01
293	PC(33:2)	-2.27	(-2.89--1.64)	**6.00E-12**	**1.37E-10**	-0.81	(-1.42--0.19)	**1.07E-02**	**1.78E-02**	-1.25	(-1.87--0.6)	**3.06E-04**	**8.08E-04**	-0.85	(-2.83-1.16)	4.02E-01	5.53E-01
294	PC(16:0_18:0)	-0.99	(-1.41--0.57)	**4.55E-06**	**1.97E-05**	-0.29	(-0.73-0.16)	2.08E-01	2.63E-01	-0.26	(-0.82-0.33)	3.83E-01	4.82E-01	-0.81	(-2.02-0.42)	1.95E-01	3.30E-01
295	PC(16:0_18:1)	-1.09	(-1.64--0.53)	**1.37E-04**	**4.33E-04**	0.44	(-0.22-1.09)	1.91E-01	2.42E-01	0.08	(-0.6-0.82)	8.21E-01	8.74E-01	-0.54	(-2.16-1.12)	5.22E-01	6.50E-01
296	PC(16:0_18:2)	-0.34	(-0.64--0.04)	**2.86E-02**	**4.82E-02**	-0.56	(-0.98--0.15)	**7.64E-03**	**1.33E-02**	-0.59	(-1.39-0.29)	1.81E-01	2.64E-01	-0.98	(-2.32-0.37)	1.54E-01	2.81E-01
297	PC(16:1_18:2)	-0.87	(-1.5--0.23)	**7.57E-03**	**1.51E-02**	-0.04	(-0.63-0.57)	9.08E-01	9.27E-01	-0.75	(-1.37--0.09)	**2.77E-02**	5.15E-02	1.83	(0.15-3.54)	**3.28E-02**	9.16E-02
298	PC(16:0_18:3) (a)	-1.78	(-2.43--1.13)	**1.51E-07**	**9.27E-07**	0.34	(-0.34-1.03)	3.27E-01	3.83E-01	0.05	(-0.73-0.9)	8.95E-01	9.24E-01	0.46	(-1.47-2.43)	6.39E-01	7.45E-01
299	PC(14:0_20:4)	-0.73	(-1.49-0.05)	6.64E-02	9.86E-02	1.14	(0.28-2.01)	**9.67E-03**	**1.64E-02**	-0.15	(-0.74-0.48)	6.36E-01	7.20E-01	4.75	(2.3-7.26)	**1.38E-04**	**1.25E-03**
300	PC(34:5)	-1.19	(-2.36-0)	**4.95E-02**	7.71E-02	1.64	(0.4-2.9)	**9.61E-03**	**1.64E-02**	-1.14	(-1.93--0.28)	**1.10E-02**	**2.23E-02**	7.01	(3.69-10.43)	**3.00E-05**	**4.64E-04**
301	PC(15-MHDA_18:1)	-2.51	(-3.31--1.72)	**1.39E-09**	**1.34E-08**	-0.81	(-1.58--0.03)	**4.30E-02**	6.35E-02	-1.26	(-1.99--0.45)	**2.77E-03**	**6.37E-03**	-1.45	(-4-1.16)	2.72E-01	4.33E-01
302	PC(17:0_18:1)	-1.99	(-2.49--1.47)	**1.15E-13**	**3.93E-12**	-0.24	(-0.76-0.29)	3.78E-01	4.33E-01	-1.06	(-1.56--0.54)	**1.14E-04**	**3.25E-04**	-1.23	(-2.85-0.43)	1.45E-01	2.67E-01
303	PC(15-MHDA_18:2)	-1.86	(-2.67--1.05)	**9.80E-06**	**4.05E-05**	-1.20	(-1.96--0.42)	**2.53E-03**	**4.94E-03**	-1.39	(-2.07--0.66)	**3.55E-04**	**9.32E-04**	-1.77	(-4.23-0.75)	1.66E-01	2.99E-01
304	PC(17:0_18:2)	-1.54	(-2.08--1)	**3.86E-08**	**2.57E-07**	-1.17	(-1.68--0.65)	**1.30E-05**	**3.74E-05**	-1.28	(-1.84--0.68)	**5.78E-05**	**1.69E-04**	-2.03	(-3.62--0.41)	**1.44E-02**	**4.92E-02**
305	PC(17:1_18:2)	-1.21	(-1.75--0.67)	**1.36E-05**	**5.30E-05**	0.46	(-0.14-1.06)	1.30E-01	1.70E-01	-0.72	(-1.27--0.14)	**1.54E-02**	**3.03E-02**	1.20	(-0.55-2.98)	1.80E-01	3.14E-01
306	PC(15:0_20:3)	-0.57	(-0.83--0.31)	**1.77E-05**	**6.79E-05**	-0.19	(-0.52-0.15)	2.73E-01	3.29E-01	-0.02	(-0.98-1.04)	9.65E-01	9.75E-01	-0.51	(-1.62-0.63)	3.79E-01	5.29E-01
307	PC(15:0_20:4)	-1.28	(-1.97--0.58)	**3.68E-04**	**1.06E-03**	-0.54	(-1.19-0.12)	1.08E-01	1.44E-01	-1.32	(-1.88--0.72)	**3.80E-05**	**1.13E-04**	1.28	(-0.87-3.47)	2.45E-01	3.97E-01
308	PC(35:5)	-1.57	(-2.8--0.32)	**1.38E-02**	**2.50E-02**	0.66	(-0.56-1.9)	2.89E-01	3.45E-01	-2.33	(-3.15--1.42)	**4.33E-06**	**1.41E-05**	4.17	(0.8-7.65)	**1.51E-02**	5.03E-02
309	PC(36:0)	0.97	(0.36-1.59)	**2.00E-03**	**4.66E-03**	3.16	(2.47-3.84)	**7.15E-19**	**3.43E-17**	0.25	(-0.29-0.82)	3.65E-01	4.66E-01	1.52	(-0.21-3.29)	8.57E-02	1.80E-01
310	PC(18:0_18:1)	-1.34	(-1.89--0.78)	**3.35E-06**	**1.49E-05**	0.98	(0.36-1.59)	**1.86E-03**	**3.74E-03**	0.47	(-0.1-1.07)	1.09E-01	1.73E-01	1.07	(-0.46-2.62)	1.71E-01	3.05E-01
311	PC(18:0_18:2)	-0.61	(-1.15--0.08)	**2.54E-02**	**4.35E-02**	-0.06	(-0.62-0.5)	8.30E-01	8.63E-01	-0.46	(-1.09-0.21)	1.76E-01	2.58E-01	0.10	(-1.42-1.65)	8.94E-01	9.48E-01
312	PC(16:0_20:3) (a)	-1.23	(-1.82--0.64)	**5.06E-05**	**1.75E-04**	-1.61	(-2.18--1.04)	**5.63E-08**	**2.33E-07**	-0.39	(-1.07-0.34)	2.85E-01	3.78E-01	-2.86	(-4.42--1.29)	**4.47E-04**	**3.02E-03**
313	PC(16:0_20:3) (b)	0.67	(-0.08-1.43)	8.02E-02	1.14E-01	1.95	(1.24-2.66)	**7.67E-08**	**3.09E-07**	0.32	(-0.31-0.98)	3.33E-01	4.32E-01	1.74	(0.01-3.51)	**4.89E-02**	1.23E-01
314	PC(18:2_18:2)	-2.57	(-3.28--1.87)	**3.69E-12**	**9.32E-11**	-2.49	(-3.22--1.75)	**1.24E-10**	**7.80E-10**	0.36	(-0.55-1.37)	4.49E-01	5.50E-01	-3.04	(-5.24--0.78)	**8.73E-03**	**3.24E-02**
315	PC(16:1_20:4)	-0.42	(-1.09-0.27)	2.30E-01	2.89E-01	0.56	(-0.07-1.2)	8.11E-02	1.12E-01	0.04	(-0.59-0.7)	9.13E-01	9.34E-01	3.69	(1.89-5.53)	**5.90E-05**	**7.86E-04**
316	PC(16:0_20:5)	-0.26	(-1.46-0.96)	6.74E-01	7.22E-01	0.55	(-0.59-1.69)	3.45E-01	4.00E-01	-1.86	(-2.69--0.94)	**1.83E-04**	**4.92E-04**	3.86	(0.8-7.01)	**1.32E-02**	**4.65E-02**
317	PC(36:6)	-2.22	(-3.03--1.4)	**1.62E-07**	**9.83E-07**	0.53	(-0.37-1.43)	2.52E-01	3.06E-01	-0.83	(-1.42--0.2)	**1.07E-02**	**2.19E-02**	4.41	(1.91-6.97)	**5.17E-04**	**3.45E-03**
318	PC(15-MHDA_20:4)	-0.68	(-1.46-0.11)	9.03E-02	1.28E-01	-0.47	(-1.22-0.29)	2.23E-01	2.78E-01	-1.62	(-2.28--0.91)	**2.18E-05**	**6.78E-05**	0.08	(-2.33-2.55)	9.47E-01	9.76E-01
319	PC(17:0_20:4)	-0.33	(-0.97-0.32)	3.20E-01	3.85E-01	-0.68	(-1.27--0.09)	**2.42E-02**	**3.73E-02**	-1.70	(-2.2--1.18)	**4.15E-09**	**1.86E-08**	-0.10	(-1.92-1.75)	9.13E-01	9.57E-01
320	PC(15:0_22:6)	-2.68	(-3.42--1.94)	**3.93E-12**	**9.43E-11**	-0.93	(-1.62--0.24)	**8.87E-03**	**1.52E-02**	-1.88	(-2.45--1.28)	**1.58E-08**	**6.70E-08**	0.59	(-1.64-2.87)	6.05E-01	7.26E-01
321	PC(38:2)	-1.13	(-1.58--0.67)	**1.45E-06**	**6.94E-06**	0.67	(0.19-1.14)	**5.76E-03**	**1.03E-02**	0.92	(0.32-1.55)	**2.19E-03**	**5.11E-03**	1.05	(-0.09-2.21)	7.19E-02	1.59E-01
322	PC(18:0_20:3)	1.23	(0.54-1.93)	**5.29E-04**	**1.41E-03**	3.69	(2.93-4.46)	**1.54E-20**	**1.06E-18**	0.12	(-0.44-0.71)	6.79E-01	7.58E-01	5.35	(3.52-7.21)	**1.22E-08**	**1.46E-06**
323	PC(18:1_20:3)	-0.59	(-1.2-0.03)	6.21E-02	9.35E-02	0.96	(0.37-1.55)	**1.39E-03**	**2.85E-03**	0.23	(-0.38-0.88)	4.60E-01	5.61E-01	0.85	(-0.73-2.46)	2.91E-01	4.50E-01
324	PC(38:4) (b)	-0.97	(-1.66--0.28)	**5.93E-03**	**1.24E-02**	1.29	(0.57-2.02)	**4.90E-04**	**1.11E-03**	-0.11	(-0.83-0.67)	7.73E-01	8.40E-01	1.67	(-0.2-3.57)	8.07E-02	1.74E-01
325	PC(18:0_20:4)	0.98	(0.35-1.61)	**2.29E-03**	**5.29E-03**	0.43	(-0.17-1.03)	1.56E-01	2.01E-01	-1.07	(-1.65--0.46)	**8.92E-04**	**2.21E-03**	1.69	(-0.04-3.44)	5.52E-02	1.34E-01
326	PC(38:5) (a)	-1.09	(-1.59--0.58)	**2.73E-05**	**9.86E-05**	-0.22	(-0.71-0.28)	3.89E-01	4.45E-01	-1.17	(-1.68--0.63)	**3.91E-05**	**1.15E-04**	0.06	(-1.45-1.59)	9.39E-01	9.74E-01
327	PC(38:5) (b)	-1.24	(-1.97--0.51)	**9.15E-04**	**2.31E-03**	0.54	(-0.23-1.31)	1.72E-01	2.20E-01	0.11	(-0.54-0.82)	7.40E-01	8.15E-01	1.61	(-0.44-3.69)	1.23E-01	2.33E-01
328	PC(38:6) (a)	-1.01	(-1.61--0.42)	**9.39E-04**	**2.36E-03**	-1.09	(-1.67--0.5)	**2.90E-04**	**6.81E-04**	0.02	(-0.69-0.79)	9.50E-01	9.64E-01	0.16	(-1.65-1.99)	8.64E-01	9.24E-01
329	PC(16:0_22:6)	-1.01	(-1.64--0.37)	**1.94E-03**	**4.55E-03**	-0.37	(-1.07-0.34)	3.10E-01	3.67E-01	-1.25	(-1.93--0.5)	**1.40E-03**	**3.31E-03**	0.13	(-1.97-2.26)	9.07E-01	9.57E-01

330	PC(18:2_20:5)	-1.76	(-2.73--0.78)	**4.83E-04**	**1.32E-03**	-0.89	(-1.88-0.12)	8.29E-02	1.15E-01	-0.64	(-1.41-0.21)	1.36E-01	2.09E-01	2.51	(-0.16-5.25)	6.60E-02	1.52E-01
331	PC(16:1_22:6)	-1.64	(-2.33--0.96)	**3.49E-06**	**1.54E-05**	-0.66	(-1.32-0.01)	5.51E-02	7.89E-02	-1.04	(-1.56--0.48)	**4.09E-04**	**1.06E-03**	1.62	(-0.35-3.62)	1.08E-01	2.12E-01
332	PC(38:7)(c)	-1.58	(-2.17--0.99)	**2.34E-07**	**1.32E-06**	-0.64	(-1.21--0.06)	**2.93E-02**	**4.47E-02**	-0.88	(-1.37--0.37)	**1.02E-03**	**2.45E-03**	-0.03	(-1.69-1.66)	9.73E-01	9.88E-01
333	PC(39:5)(a)	-2.53	(-3.35--1.7)	**4.56E-09**	**3.53E-08**	-1.82	(-2.59--1.05)	**4.96E-06**	**1.51E-05**	-1.98	(-2.62--1.29)	**2.49E-07**	**9.28E-07**	-0.54	(-2.96-1.95)	6.68E-01	7.58E-01
334	PC(39:5)(b)	-2.31	(-3.04--1.58)	**1.68E-09**	**1.56E-08**	-0.86	(-1.58--0.13)	**2.03E-02**	**3.21E-02**	-1.95	(-2.55--1.29)	**7.31E-08**	**2.90E-07**	-0.94	(-3.24-1.42)	4.31E-01	5.75E-01
335	PC(15-MHDA_22:6)	-2.51	(-3.31--1.7)	**2.77E-09**	**2.33E-08**	-1.62	(-2.33--0.91)	**1.10E-05**	**3.17E-05**	-2.07	(-2.73--1.36)	**1.67E-07**	**6.31E-07**	-1.16	(-3.44-1.17)	3.25E-01	4.86E-01
336	PC(17:0_22:6)	-2.00	(-2.74--1.26)	**1.87E-07**	**1.08E-06**	-1.05	(-1.79--0.31)	**5.44E-03**	**9.82E-03**	-2.24	(-2.78--1.65)	**1.65E-11**	**9.21E-11**	-0.48	(-2.73-1.83)	6.82E-01	7.69E-01
337	PC(18:0_22:5) (n6)	-1.32	(-2.07--0.57)	**6.32E-04**	**1.67E-03**	1.17	(0.37-1.97)	**3.94E-03**	**7.42E-03**	-1.24	(-2.36-0.06)	6.01E-02	1.04E-01	2.25	(0.3-4.25)	**2.39E-02**	7.17E-02
338	PC(18:0_22:6)	-0.61	(-1.32-0.1)	9.21E-02	1.30E-01	1.19	(0.44-1.95)	**1.98E-03**	**3.94E-03**	-1.44	(-2.09--0.74)	**1.21E-04**	**3.40E-04**	3.01	(0.9-5.18)	**5.22E-03**	**2.12E-02**
339	PC(40:7) (a)	-1.36	(-1.99--0.73)	**2.64E-05**	**9.76E-05**	0.02	(-0.65-0.69)	9.56E-01	9.69E-01	0.94	(0-1.97)	**4.91E-02**	8.72E-02	1.35	(-0.64-3.39)	1.84E-01	3.18E-01
340	PC(18:1_22:6) (a)	-1.70	(-2.24--1.16)	**1.69E-09**	**1.56E-08**	-0.86	(-1.44--0.27)	**4.10E-03**	**7.62E-03**	-0.45	(-1.13-0.27)	2.15E-01	3.04E-01	-1.64	(-3.29-0.03)	5.37E-02	1.31E-01
341	PC(40:8)	-1.77	(-2.3--1.24)	**1.86E-10**	**2.70E-09**	-1.88	(-2.39--1.37)	**2.15E-12**	**1.91E-11**	0.83	(0.2-1.5)	**9.19E-03**	**1.92E-02**	-0.97	(-2.47-0.55)	2.09E-01	3.48E-01
342	PC(44:12)	-2.40	(-3.44--1.34)	**1.14E-05**	**4.60E-05**	-2.61	(-3.74--1.46)	**1.06E-05**	**3.09E-05**	-1.02	(-1.76--0.22)	**1.39E-02**	**2.75E-02**	-0.64	(-3.66-2.47)	6.82E-01	7.69E-01
343	PC(O-16:0/16:0)	-0.78	(-1.21--0.35)	**3.82E-04**	**1.09E-03**	-0.75	(-1.18--0.33)	**5.02E-04**	**1.13E-03**	0.45	(-0.19-1.14)	1.70E-01	2.52E-01	-1.08	(-2.23-0.07)	6.58E-02	1.52E-01
344	PC(O-32:1)	-1.01	(-1.56--0.45)	**4.20E-04**	**1.18E-03**	-0.53	(-1.07-0.02)	5.89E-02	8.34E-02	0.73	(0-1.52)	5.05E-02	8.95E-02	-0.34	(-1.82-1.16)	6.50E-01	7.50E-01
345	PC(O-34:1)	-0.84	(-1.27--0.41)	**1.54E-04**	**4.84E-04**	-1.21	(-1.62--0.79)	**2.44E-08**	**1.08E-07**	0.68	(0.02-1.38)	**4.18E-02**	7.52E-02	-2.24	(-3.3--1.18)	**4.84E-05**	**7.04E-04**
346	PC(O-34:2)	-2.02	(-2.62--1.41)	**2.10E-10**	**2.80E-09**	-1.75	(-2.35--1.15)	**2.36E-08**	**1.05E-07**	-0.06	(-0.77-0.69)	8.63E-01	9.01E-01	-1.81	(-3.58-0)	**4.98E-02**	1.24E-01
347	PC(O-34:4)	-0.88	(-1.84-0.1)	7.86E-02	1.13E-01	-0.61	(-1.64-0.42)	2.46E-01	3.02E-01	-0.01	(-0.7-0.73)	9.69E-01	9.77E-01	1.29	(-1.45-4.11)	3.58E-01	5.10E-01
348	PC(O-35:4)	-0.89	(-1.73--0.04)	**4.09E-02**	6.65E-02	-0.70	(-1.63-0.24)	1.44E-01	1.86E-01	-0.49	(-1.13-0.19)	1.52E-01	2.29E-01	0.45	(-2.21-3.19)	7.41E-01	8.21E-01
349	PC(O-36:0)	-1.30	(-1.8--0.81)	**3.88E-07**	**2.12E-06**	-0.84	(-1.33--0.35)	**9.07E-04**	**1.95E-03**	0.14	(-0.67-1.02)	7.52E-01	8.24E-01	-2.41	(-3.76--1.03)	**6.74E-04**	**4.23E-03**
350	PC(O-18:0/18:1)	-1.51	(-1.98--1.03)	**1.00E-09**	**1.02E-08**	-1.48	(-1.92--1.04)	**1.07E-10**	**6.87E-10**	1.31	(0.62-2.05)	**1.35E-04**	**3.71E-04**	-3.04	(-4.22--1.85)	**1.05E-06**	**3.88E-05**
351	PC(O-18:1/18:1)	-1.20	(-1.66--0.74)	**4.16E-07**	**2.24E-06**	-1.71	(-2.13--1.29)	**1.10E-14**	**1.42E-13**	0.71	(0.11-1.35)	**2.01E-02**	**3.86E-02**	-1.86	(-2.95--0.75)	**1.12E-03**	**6.20E-03**
352	PC(O-18:0/18:2)	-2.36	(-2.92--1.79)	**3.60E-15**	**1.73E-13**	-1.89	(-2.38--1.39)	**4.43E-13**	**4.43E-12**	0.44	(-0.18-1.11)	1.67E-01	2.51E-01	-2.63	(-4.12--1.12)	**7.48E-04**	**4.54E-03**
353	PC(O-18:1/18:2)	-1.41	(-1.96--0.86)	**7.43E-07**	**3.71E-06**	-1.34	(-1.85--0.82)	**6.84E-07**	**2.35E-06**	-0.06	(-0.7-0.63)	8.59E-01	9.01E-01	-1.45	(-2.94-0.06)	5.92E-02	1.43E-01
354	PC(O-16:0/20:3)	-0.51	(-1.03-0.02)	6.07E-02	9.23E-02	0.21	(-0.32-0.76)	4.34E-01	4.92E-01	0.39	(-0.29-1.12)	2.68E-01	3.62E-01	0.94	(-0.55-2.45)	2.17E-01	3.61E-01
355	PC(O-16:0/20:4)	-0.15	(-0.68-0.38)	5.74E-01	6.26E-01	-0.43	(-0.95-0.09)	1.04E-01	1.39E-01	-0.15	(-0.73-0.46)	6.14E-01	6.98E-01	0.53	(-0.97-2.05)	4.89E-01	6.31E-01
356	PC(O-36:5)	-1.11	(-2.09--0.12)	**2.87E-02**	**4.82E-02**	-0.38	(-1.4-0.66)	4.72E-01	5.31E-01	-1.06	(-1.73--0.34)	**4.52E-03**	**9.91E-03**	2.54	(-0.25-5.41)	7.43E-02	1.63E-01
357	PC(O-18:0/20:4)	-0.40	(-0.91-0.12)	1.28E-01	1.76E-01	-0.72	(-1.18--0.24)	**3.06E-03**	**5.83E-03**	-0.31	(-0.85-0.26)	2.75E-01	3.66E-01	-1.33	(-2.77-0.14)	7.48E-02	1.63E-01
358	PC(O-38:5)	-0.01	(-0.47-0.46)	9.81E-01	9.83E-01	-0.63	(-1.12--0.13)	**1.31E-02**	**2.16E-02**	-0.16	(-0.83-0.55)	6.44E-01	7.28E-01	0.05	(-1.34-1.45)	9.46E-01	9.76E-01
359	PC(O-16:0/22:6)	-0.23	(-0.91-0.45)	5.00E-01	5.53E-01	-0.30	(-1-0.4)	3.99E-01	4.55E-01	-0.81	(-1.4--0.17)	**1.38E-02**	**2.75E-02**	1.12	(-0.78-3.07)	2.49E-01	4.02E-01
360	PC(O-40:5)	-0.80	(-1.3--0.3)	**1.67E-03**	**3.98E-03**	-0.88	(-1.34--0.42)	**1.90E-04**	**4.60E-04**	-0.16	(-0.68-0.39)	5.65E-01	6.53E-01	-0.79	(-2.12-0.56)	2.48E-01	4.01E-01
361	PC(O-18:0/22:6)	-0.69	(-1.25--0.12)	**1.77E-02**	**3.15E-02**	-1.09	(-1.63--0.55)	**9.15E-05**	**2.31E-04**	-0.93	(-1.43--0.39)	**9.93E-04**	**2.41E-03**	-1.23	(-2.72-0.29)	1.11E-01	2.14E-01
362	PC(O-40:7) (a)	-0.06	(-0.71-0.59)	8.54E-01	8.76E-01	-0.80	(-1.41--0.18)	**1.10E-02**	**1.83E-02**	-0.71	(-1.28--0.11)	**2.17E-02**	**4.11E-02**	-0.07	(-1.89-1.79)	9.42E-01	9.74E-01
363	PC(P-16:0/14:0)	-1.27	(-1.86--0.68)	**2.71E-05**	**9.86E-05**	-1.18	(-1.79--0.56)	**1.98E-04**	**4.77E-04**	-0.35	(-1.06-0.41)	3.52E-01	4.52E-01	-0.01	(-1.86-1.89)	9.95E-01	9.99E-01
364	PC(P-16:0/16:0)	-0.71	(-1.21--0.2)	**6.69E-03**	**1.37E-02**	-1.03	(-1.54--0.53)	**6.60E-05**	**1.72E-04**	-0.52	(-1.15-0.16)	1.33E-01	2.06E-01	-1.25	(-2.56-0.08)	6.48E-02	1.50E-01
365	PC(P-16:0/16:1)	-0.71	(-1.31--0.11)	**2.01E-02**	**3.52E-02**	-0.83	(-1.39--0.26)	**4.66E-03**	**8.63E-03**	-0.04	(-0.71-0.69)	9.17E-01	9.36E-01	0.80	(-0.71-2.32)	3.00E-01	4.58E-01
366	PC(P-16:0/18:0)	-1.20	(-1.59--0.81)	**3.58E-09**	**2.91E-08**	-1.47	(-1.84--1.09)	**1.05E-13**	**1.20E-12**	-0.24	(-0.76-0.32)	4.00E-01	5.02E-01	-2.11	(-3.09--1.13)	**3.73E-05**	**5.60E-04**
367	PC(P-16:0/18:1)	-1.21	(-1.75--0.67)	**1.36E-05**	**5.30E-05**	-2.19	(-2.7--1.69)	**2.94E-16**	**6.14E-15**	-0.28	(-0.86-0.34)	3.73E-01	4.73E-01	-1.99	(-3.17--0.79)	**1.27E-03**	**6.69E-03**
368	PC(P-16:0/18:2)	-1.10	(-1.68--0.5)	**3.02E-04**	**8.73E-04**	-2.03	(-2.57--1.48)	**1.39E-12**	**1.28E-11**	-1.30	(-1.9--0.66)	**1.43E-04**	**3.91E-04**	-1.51	(-3.09-0.09)	6.46E-02	1.50E-01
369	PC(P-16:0/18:3)	-1.64	(-2.27--1.01)	**5.09E-07**	**2.66E-06**	-1.42	(-1.99--0.84)	**2.28E-06**	**7.17E-06**	-0.48	(-1.01-0.08)	9.39E-02	1.53E-01	-0.44	(-2.21-1.35)	6.24E-01	7.41E-01
370	PC(P-35:2) (a)	-2.26	(-3.13--1.39)	**5.70E-07**	**2.91E-06**	-1.87	(-2.71--1.02)	**1.87E-05**	**5.25E-05**	-0.45	(-1.13-0.28)	2.20E-01	3.08E-01	-2.39	(-4.91-0.2)	7.04E-02	1.58E-01
371	PC(P-35:2) (b)	-1.83	(-2.55--1.1)	**1.07E-06**	**5.20E-06**	-2.14	(-2.83--1.45)	**2.27E-09**	**1.13E-08**	-1.45	(-2.06--0.79)	**3.89E-05**	**1.15E-04**	-2.72	(-4.69--0.7)	**8.59E-03**	**3.22E-02**
372	PC(P-15:0/20:4) (b)	-0.66	(-1.34-0.01)	5.51E-02	8.45E-02	-0.44	(-1.19-0.32)	2.53E-01	3.07E-01	-0.76	(-1.47-0.01)	5.19E-02	9.09E-02	0.40	(-1.76-2.61)	7.16E-01	7.98E-01
373	PC(P-18:1/18:1)	-1.20	(-1.72--0.68)	**7.54E-06**	**3.17E-05**	-1.58	(-2.08--1.09)	**7.85E-10**	**4.18E-09**	1.22	(0.39-2.12)	**3.50E-03**	**7.92E-03**	-0.48	(-1.71-0.76)	4.45E-01	5.88E-01
374	PC(P-18:0/18:2)	-1.93	(-2.51--1.36)	**1.35E-10**	**2.16E-09**	-2.58	(-3.1--2.06)	**1.54E-20**	**1.06E-18**	-0.65	(-1.19--0.07)	**2.78E-02**	5.15E-02	-2.97	(-4.48--1.45)	**1.69E-04**	**1.48E-03**
375	PC(P-16:0/20:4)	0.43	(-0.18-1.05)	1.67E-01	2.22E-01	-0.65	(-1.18--0.11)	**1.81E-02**	**2.88E-02**	-1.10	(-1.65--0.5)	**4.44E-04**	**1.14E-03**	0.30	(-1.26-1.89)	7.03E-01	7.91E-01
376	PC(P-16:0/20:5)	-0.52	(-1.56-0.53)	3.29E-01	3.95E-01	-0.31	(-1.37-0.77)	5.75E-01	6.33E-01	-1.95	(-2.64--1.2)	**2.21E-06**	**7.46E-06**	3.00	(0.12-5.97)	**4.13E-02**	1.10E-01
377	PC(P-17:0/20:4) (a)	-1.01	(-1.8--0.22)	**1.29E-02**	**2.38E-02**	-0.63	(-1.41-0.16)	1.19E-01	1.57E-01	-1.57	(-2.22--0.86)	**3.59E-05**	**1.08E-04**	-0.85	(-3.43-1.79)	5.22E-01	6.50E-01

378	PC(P-17:0/20:4) (b)	-0.32	(-1.05-0.42)	3.96E-01	4.59E-01	-1.00	(-1.68--0.31)	**4.69E-03**	**8.66E-03**	-1.89	(-2.47--1.27)	**4.13E-08**	**1.68E-07**	-1.03	(-3.11-1.1)	3.39E-01	4.95E-01
379	PC(P-18:0/20:4)	-0.09	(-0.67-0.5)	7.64E-01	8.02E-01	-1.20	(-1.71--0.68)	**7.83E-06**	**2.32E-05**	-1.09	(-1.6--0.56)	**1.16E-04**	**3.27E-04**	-0.77	(-2.4-0.88)	3.56E-01	5.09E-01
380	PC(P-38:5) (a)	0.24	(-0.36-0.84)	4.31E-01	4.89E-01	-0.55	(-1.09--0.01)	**4.46E-02**	6.57E-02	-1.21	(-1.75--0.63)	**8.09E-05**	**2.33E-04**	0.72	(-0.88-2.35)	3.77E-01	5.29E-01
381	PC(P-38:5) (b)	-0.85	(-1.43--0.27)	**4.44E-03**	**9.70E-03**	-1.10	(-1.67--0.53)	**1.76E-04**	**4.33E-04**	-0.87	(-1.5--0.2)	**1.16E-02**	**2.34E-02**	-0.41	(-2.09-1.31)	6.40E-01	7.45E-01
382	PC(P-16:0/22:6)	-0.29	(-1.01-0.43)	4.26E-01	4.86E-01	-0.95	(-1.62--0.28)	**5.79E-03**	**1.03E-02**	-1.52	(-2.09--0.91)	**3.95E-06**	**1.31E-05**	0.28	(-1.67-2.27)	7.79E-01	8.55E-01
383	PC(P-20:0/20:4)	-0.43	(-1.04-0.17)	1.58E-01	2.12E-01	-1.30	(-1.83--0.77)	**1.74E-06**	**5.57E-06**	-0.86	(-1.45--0.23)	**7.93E-03**	**1.68E-02**	-1.69	(-3.16--0.2)	**2.69E-02**	7.82E-02
384	PC(P-40:5) (b)	-0.80	(-1.37--0.24)	**5.56E-03**	**1.18E-02**	-1.41	(-1.93--0.9)	**1.16E-07**	**4.47E-07**	-0.80	(-1.31--0.26)	**4.26E-03**	**9.37E-03**	-1.36	(-2.75-0.05)	5.94E-02	1.43E-01
385	PC(P-18:0/22:6)	-0.68	(-1.37-0)	5.13E-02	7.95E-02	-2.07	(-2.72--1.42)	**9.79E-10**	**5.11E-09**	-1.57	(-2.08--1.02)	**1.26E-07**	**4.78E-07**	-1.63	(-3.5-0.27)	9.23E-02	1.88E-01
386	PE(16:0_16:1)	-0.54	(-1.69-0.63)	3.62E-01	4.26E-01	3.03	(1.74-4.33)	**3.84E-06**	**1.18E-05**	0.44	(-0.57-1.57)	4.07E-01	5.08E-01	4.35	(0.88-7.95)	**1.38E-02**	**4.78E-02**
387	PE(16:0_18:1)	-0.10	(-0.87-0.67)	7.94E-01	8.25E-01	1.21	(0.29-2.14)	**1.01E-02**	**1.71E-02**	-0.60	(-1.29-0.14)	1.11E-01	1.76E-01	0.32	(-2.24-2.94)	8.11E-01	8.80E-01
388	PE(16:0_18:2)	-0.32	(-1.18-0.55)	4.71E-01	5.29E-01	0.55	(-0.44-1.56)	2.76E-01	3.31E-01	-0.68	(-1.39-0.1)	8.58E-02	1.42E-01	-0.95	(-3.68-1.86)	5.03E-01	6.36E-01
389	PE(16:1_18:2)	-0.95	(-1.96-0.08)	6.93E-02	1.01E-01	0.53	(-0.35-1.43)	2.39E-01	2.94E-01	0.38	(-0.4-1.22)	3.50E-01	4.50E-01	0.00	(-2.28-2.33)	1.00E+00	1.00E+00
390	PE(16:0_18:3) (a)	-1.62	(-2.53--0.69)	**6.80E-04**	**1.77E-03**	0.67	(-0.33-1.68)	1.90E-01	2.42E-01	0.07	(-0.71-0.9)	8.73E-01	9.06E-01	-0.67	(-3.48-2.22)	6.45E-01	7.48E-01
391	PE(16:0_18:3) (b)	-0.42	(-1.33-0.49)	3.65E-01	4.27E-01	1.99	(0.89-3.11)	**3.82E-04**	**8.81E-04**	0.56	(-0.31-1.5)	2.15E-01	3.04E-01	1.71	(-1.39-4.9)	2.82E-01	4.41E-01
392	PE(17:0_18:2)	-1.21	(-1.9--0.52)	**6.65E-04**	**1.74E-03**	0.07	(-0.73-0.89)	8.59E-01	8.83E-01	-0.85	(-1.35--0.31)	**2.33E-03**	**5.41E-03**	-1.18	(-3.43-1.13)	3.14E-01	4.72E-01
393	PE(18:0_18:1)	0.43	(-0.21-1.08)	1.90E-01	2.46E-01	1.56	(0.67-2.45)	**5.73E-04**	**1.26E-03**	-0.09	(-0.87-0.75)	8.21E-01	8.74E-01	2.66	(0.19-5.19)	**3.45E-02**	9.47E-02
394	PE(18:1_18:1)	-0.40	(-1.06-0.27)	2.42E-01	3.03E-01	0.28	(-0.49-1.05)	4.78E-01	5.36E-01	-0.01	(-1.07-1.17)	9.83E-01	9.83E-01	0.26	(-2.06-2.63)	8.29E-01	8.96E-01
395	PE(18:0_18:2)	0.50	(-0.21-1.22)	1.70E-01	2.25E-01	1.26	(0.38-2.15)	**5.10E-03**	**9.24E-03**	-0.50	(-1.17-0.22)	1.68E-01	2.51E-01	1.32	(-1.13-3.82)	2.93E-01	4.50E-01
396	PE(18:1_18:2)	-0.77	(-1.58-0.04)	6.38E-02	9.54E-02	-0.81	(-1.77-0.15)	9.83E-02	1.33E-01	-0.26	(-1.01-0.56)	5.29E-01	6.21E-01	-2.14	(-4.71-0.49)	1.10E-01	2.14E-01
397	PE(16:0_20:3)	-0.37	(-1.21-0.47)	3.85E-01	4.48E-01	0.97	(-0.01-1.97)	5.21E-02	7.54E-02	-0.31	(-1.06-0.5)	4.42E-01	5.42E-01	-0.12	(-2.72-2.55)	9.29E-01	9.67E-01
398	PE(16:0_20:4)	0.75	(0.01-1.5)	**4.65E-02**	7.31E-02	-0.44	(-1.27-0.4)	3.04E-01	3.60E-01	-0.34	(-0.96-0.33)	3.14E-01	4.11E-01	-1.51	(-3.89-0.92)	2.19E-01	3.63E-01
399	PE(16:1_20:4)	-0.18	(-1.08-0.73)	6.98E-01	7.41E-01	-0.08	(-0.84-0.69)	8.40E-01	8.71E-01	0.73	(-0.09-1.62)	8.04E-02	1.34E-01	1.01	(-1.06-3.12)	3.42E-01	4.98E-01
400	PE(16:0_20:5)	-0.47	(-1.52-0.58)	3.80E-01	4.43E-01	0.27	(-0.78-1.33)	6.16E-01	6.70E-01	-0.82	(-1.58-0.01)	5.18E-02	9.09E-02	1.62	(-1.04-4.36)	2.34E-01	3.82E-01
401	PE(17:0_20:4)	-0.03	(-0.7-0.65)	9.34E-01	9.46E-01	-0.05	(-0.82-0.72)	8.96E-01	9.17E-01	-1.71	(-2.3--1.07)	**8.04E-07**	**2.86E-06**	-1.45	(-3.59-0.74)	1.91E-01	3.24E-01
402	PE(18:0_20:3) (a)	1.68	(0.87-2.5)	**4.94E-05**	**1.72E-04**	2.90	(1.96-3.84)	**1.44E-09**	**7.41E-09**	-0.61	(-1.25-0.09)	8.50E-02	1.41E-01	3.60	(1.22-6.04)	**3.02E-03**	**1.41E-02**
403	PE(18:0_20:4)	2.04	(1.41-2.67)	**2.05E-10**	**2.80E-09**	0.25	(-0.47-0.96)	5.01E-01	5.59E-01	-0.70	(-1.29--0.07)	**3.03E-02**	5.55E-02	0.55	(-1.48-2.63)	5.96E-01	7.19E-01
404	PE(38:5) (a)	0.01	(-0.72-0.75)	9.72E-01	9.76E-01	-0.72	(-1.56-0.13)	9.55E-02	1.30E-01	-0.63	(-1.26-0.04)	6.53E-02	1.12E-01	-1.61	(-3.89-0.73)	1.76E-01	3.07E-01
405	PE(38:5) (b)	-0.87	(-1.73--0.01)	**4.69E-02**	7.36E-02	0.00	(-0.94-0.95)	9.99E-01	9.99E-01	0.39	(-0.63-1.53)	4.68E-01	5.68E-01	0.76	(-2.02-3.62)	5.95E-01	7.19E-01
406	PE(16:0_22:6)	0.00	(-0.73-0.74)	9.96E-01	9.96E-01	-0.56	(-1.51-0.4)	2.52E-01	3.06E-01	-1.58	(-2.23--0.88)	**2.50E-05**	**7.75E-05**	-0.46	(-3.22-2.37)	7.47E-01	8.26E-01
407	PE(17:0_22:6)	-0.21	(-0.95-0.55)	5.87E-01	6.38E-01	-0.18	(-0.98-0.62)	6.55E-01	7.03E-01	-1.52	(-2.1--0.89)	**7.66E-06**	**2.47E-05**	-0.10	(-2.25-2.09)	9.25E-01	9.66E-01
408	PE(18:0_22:4)	0.52	(-0.35-1.4)	2.43E-01	3.03E-01	1.66	(0.84-2.48)	**6.84E-05**	**1.77E-04**	-0.22	(-1.01-0.65)	6.13E-01	6.98E-01	2.27	(0.13-4.45)	**3.74E-02**	1.00E-01
409	PE(18:0_22:5) (n3)	0.45	(-0.26-1.16)	2.14E-01	2.72E-01	1.79	(0.94-2.64)	**3.74E-05**	**9.97E-05**	-1.09	(-1.86--0.24)	**1.27E-02**	**2.54E-02**	2.54	(0.19-4.95)	**3.42E-02**	9.47E-02
410	PE(18:0_22:5) (n6)	0.40	(-0.53-1.33)	4.00E-01	4.62E-01	0.72	(-0.23-1.68)	1.39E-01	1.80E-01	-0.46	(-1.41-0.59)	3.78E-01	4.77E-01	2.62	(0.04-5.27)	**4.66E-02**	1.18E-01
411	PE(18:0_22:6)	1.34	(0.55-2.14)	**8.34E-04**	**2.13E-03**	1.67	(0.73-2.63)	**5.37E-04**	**1.20E-03**	-1.61	(-2.34--0.8)	**2.02E-04**	**5.43E-04**	3.81	(1.08-6.61)	**6.06E-03**	**2.36E-02**
412	PE(40:7)	-0.40	(-1.25-0.44)	3.48E-01	4.14E-01	-1.43	(-2.41--0.44)	**4.72E-03**	**8.68E-03**	-1.29	(-1.94--0.58)	**5.38E-04**	**1.36E-03**	-1.14	(-3.97-1.76)	4.36E-01	5.79E-01
413	PE(O-34:1)	-1.42	(-2--0.83)	**2.58E-06**	**1.19E-05**	-1.43	(-2.04--0.82)	**6.33E-06**	**1.91E-05**	0.33	(-0.78-1.57)	5.73E-01	6.59E-01	-1.48	(-3.2-0.26)	9.51E-02	1.93E-01
414	PE(O-16:0/18:2)	-2.74	(-3.5--1.96)	**1.39E-11**	**2.79E-10**	-2.48	(-3.27--1.68)	**2.70E-09**	**1.32E-08**	-0.23	(-2-1.95)	8.24E-01	8.75E-01	-0.34	(-2.54-1.91)	7.64E-01	8.42E-01
415	PE(O-18:1/18:2)	-1.56	(-2.3--0.82)	**4.20E-05**	**1.47E-04**	-1.10	(-1.81--0.38)	**2.71E-03**	**5.25E-03**	0.06	(-0.88-1.09)	9.08E-01	9.31E-01	-0.25	(-2.27-1.81)	8.10E-01	8.80E-01
416	PE(O-16:0/20:4)	-1.39	(-2.12--0.65)	**2.38E-04**	**7.02E-04**	-1.38	(-2.13--0.61)	**4.51E-04**	**1.03E-03**	-0.81	(-1.62-0.07)	6.94E-02	1.18E-01	1.19	(-0.97-3.4)	2.82E-01	4.41E-01
417	PE(O-36:5)	-2.34	(-3.42--1.25)	**3.37E-05**	**1.20E-04**	-1.17	(-2.3--0.03)	**4.50E-02**	6.59E-02	-0.42	(-1.08-0.29)	2.38E-01	3.31E-01	2.28	(-0.48-5.12)	1.06E-01	2.10E-01
418	PE(O-16:0/22:4)	-0.63	(-1.21--0.05)	**3.37E-02**	5.62E-02	-0.79	(-1.37--0.22)	**7.21E-03**	**1.26E-02**	0.56	(-0.18-1.36)	1.43E-01	2.19E-01	0.37	(-1.29-2.06)	6.60E-01	7.54E-01
419	PE(O-18:0/20:4)	-1.81	(-2.63--0.99)	**2.02E-05**	**7.69E-05**	-1.76	(-2.55--0.97)	**1.81E-05**	**5.11E-05**	-0.17	(-0.95-0.69)	6.94E-01	7.73E-01	0.60	(-1.82-3.08)	6.30E-01	7.41E-01
420	PE(O-38:5) (a)	-1.44	(-2.16--0.71)	**1.14E-04**	**3.65E-04**	-0.89	(-1.6--0.18)	**1.37E-02**	**2.25E-02**	-0.10	(-0.78-0.64)	7.90E-01	8.51E-01	1.52	(-0.73-3.83)	1.86E-01	3.18E-01
421	PE(O-38:5) (b)	-1.98	(-2.64--1.31)	**9.88E-09**	**6.87E-08**	-1.06	(-1.81--0.29)	**6.73E-03**	**1.19E-02**	0.16	(-0.75-1.17)	7.38E-01	8.15E-01	0.24	(-1.68-2.19)	8.07E-01	8.80E-01
422	PE(O-16:0/22:6)	-1.03	(-1.66--0.4)	**1.52E-03**	**3.67E-03**	-0.83	(-1.53--0.13)	**2.05E-02**	**3.21E-02**	0.55	(-0.23-1.38)	1.70E-01	2.52E-01	0.77	(-1.03-2.6)	4.02E-01	5.53E-01
423	PE(O-18:0/22:6)	-1.54	(-2.14--0.93)	**1.02E-06**	**5.01E-06**	-1.41	(-2.04--0.77)	**1.78E-05**	**5.04E-05**	0.01	(-0.62-0.68)	9.77E-01	9.79E-01	-0.02	(-1.8-1.8)	9.85E-01	9.93E-01
424	PE(O-18:1/22:6)	-1.16	(-1.76--0.56)	**1.73E-04**	**5.35E-04**	-1.29	(-1.9--0.67)	**5.13E-05**	**1.35E-04**	0.08	(-0.49-0.68)	7.96E-01	8.54E-01	0.41	(-1.42-2.28)	6.60E-01	7.54E-01
425	PE(P-16:0/18:1)	-1.04	(-1.56--0.52)	**1.03E-04**	**3.35E-04**	-0.62	(-1.13--0.11)	**1.76E-02**	**2.81E-02**	1.69	(0.98-2.44)	**1.11E-06**	**3.90E-06**	-0.19	(-1.6-1.25)	7.98E-01	8.73E-01

426	PE(P-16:0/18:2)	-1.53	(-2.2--0.85)	**1.07E-05**	**4.41E-05**	-0.73	(-1.4--0.05)	**3.44E-02**	5.14E-02	0.14	(-0.73-1.1)	7.59E-01	8.29E-01	0.55	(-1.4-2.55)	5.80E-01	7.05E-01
427	PE(P-16:0/20:3)	-0.36	(-1.08-0.37)	3.33E-01	3.97E-01	1.10	(0.38-1.82)	**2.78E-03**	**5.35E-03**	0.18	(-0.65-1.09)	6.78E-01	7.58E-01	4.14	(2.26-6.06)	**1.60E-05**	**3.36E-04**
428	PE(P-16:0/20:4)	0.15	(-0.58-0.88)	6.90E-01	7.36E-01	0.18	(-0.55-0.9)	6.32E-01	6.83E-01	-0.23	(-0.99-0.59)	5.66E-01	6.53E-01	3.45	(1.36-5.57)	**1.16E-03**	**6.34E-03**
429	PE(P-16:0/20:5)	-1.00	(-2.32-0.34)	1.42E-01	1.93E-01	0.17	(-1.12-1.48)	7.96E-01	8.34E-01	-0.81	(-2.51-1.27)	4.15E-01	5.15E-01	4.78	(1.15-8.54)	**9.55E-03**	**3.47E-02**
430	PE(P-16:0/22:4)	0.38	(-0.18-0.94)	1.87E-01	2.43E-01	-0.41	(-0.96-0.13)	1.38E-01	1.79E-01	0.23	(-0.47-0.98)	5.32E-01	6.23E-01	-0.44	(-1.83-0.97)	5.37E-01	6.65E-01
431	PE(P-16:0/22:5) (n3)	-0.62	(-1.21--0.02)	**4.12E-02**	6.65E-02	0.30	(-0.26-0.87)	2.97E-01	3.54E-01	-0.48	(-1.19-0.28)	2.09E-01	2.98E-01	1.24	(-0.52-3.03)	1.69E-01	3.02E-01
432	PE(P-16:0/22:5) (n6)	-0.76	(-1.4--0.12)	**2.07E-02**	**3.60E-02**	-0.37	(-0.95-0.22)	2.21E-01	2.77E-01	0.91	(0.1-1.79)	**2.66E-02**	**4.98E-02**	0.45	(-1.46-2.39)	6.48E-01	7.49E-01
433	PE(P-16:0/22:6)	0.09	(-0.49-0.67)	7.67E-01	8.02E-01	-0.02	(-0.62-0.58)	9.45E-01	9.61E-01	0.17	(-0.5-0.88)	6.33E-01	7.19E-01	1.74	(0.13-3.38)	**3.45E-02**	9.47E-02
434	PE(P-17:0/20:4) (a)	-0.28	(-1.14-0.59)	5.22E-01	5.75E-01	-0.20	(-1.09-0.7)	6.62E-01	7.10E-01	-0.39	(-0.98-0.23)	2.11E-01	2.99E-01	1.34	(-1-3.73)	2.64E-01	4.21E-01
435	PE(P-17:0/20:4) (b)	-0.21	(-1.01-0.6)	6.15E-01	6.63E-01	-0.27	(-1.05-0.52)	5.04E-01	5.62E-01	-0.96	(-1.57--0.3)	**4.73E-03**	**1.03E-02**	1.16	(-0.96-3.33)	2.86E-01	4.45E-01
436	PE(P-17:0/22:6) (a)	-1.24	(-2.03--0.44)	**2.33E-03**	**5.35E-03**	-0.51	(-1.32-0.31)	2.22E-01	2.77E-01	-0.27	(-1.02-0.55)	5.11E-01	6.02E-01	0.05	(-2.29-2.45)	9.65E-01	9.85E-01
437	PE(P-17:0/22:6) (b)	-0.75	(-1.46--0.03)	**4.23E-02**	6.81E-02	-0.90	(-1.67--0.13)	**2.28E-02**	**3.55E-02**	-0.44	(-1.11-0.27)	2.18E-01	3.06E-01	-0.50	(-2.48-1.53)	6.27E-01	7.41E-01
438	PE(P-18:0/18:1)	-1.25	(-1.84--0.66)	**3.98E-05**	**1.40E-04**	-1.01	(-1.6--0.41)	**9.72E-04**	**2.07E-03**	1.01	(0.24-1.85)	**9.34E-03**	**1.94E-02**	-0.71	(-2.29-0.88)	3.78E-01	5.29E-01
439	PE(P-18:0/18:2)	-1.83	(-2.48--1.19)	**4.04E-08**	**2.66E-07**	-1.53	(-2.15--0.91)	**1.62E-06**	**5.22E-06**	-0.15	(-1-0.78)	7.43E-01	8.16E-01	-0.69	(-2.59-1.24)	4.78E-01	6.22E-01
440	PE(P-18:0/20:3)	-0.92	(-1.55--0.28)	**4.71E-03**	**1.01E-02**	-0.15	(-0.76-0.46)	6.31E-01	6.83E-01	-0.34	(-0.91-0.26)	2.56E-01	3.51E-01	2.28	(0.68-3.89)	**5.08E-03**	**2.10E-02**
441	PE(P-18:0/20:4)	-0.19	(-0.95-0.58)	6.33E-01	6.81E-01	-0.83	(-1.51--0.15)	**1.66E-02**	**2.68E-02**	-0.65	(-1.33-0.08)	7.77E-02	1.30E-01	1.63	(-0.45-3.75)	1.24E-01	2.33E-01
442	PE(P-18:0/20:5)	-1.63	(-2.99--0.26)	**2.00E-02**	**3.52E-02**	-0.80	(-2.1-0.52)	2.34E-01	2.91E-01	-1.97	(-3.3--0.38)	**1.77E-02**	**3.42E-02**	3.62	(0.14-7.22)	**4.16E-02**	1.10E-01
443	PE(P-18:0/22:4)	-0.72	(-1.31--0.13)	**1.74E-02**	**3.11E-02**	-0.98	(-1.57--0.38)	**1.48E-03**	**3.02E-03**	-0.28	(-1.04-0.54)	4.88E-01	5.80E-01	-1.74	(-3.12--0.34)	**1.51E-02**	5.03E-02
444	PE(P-18:0/22:5) (n3)	-0.81	(-1.4--0.21)	**8.18E-03**	**1.61E-02**	-0.47	(-1-0.07)	8.56E-02	1.17E-01	-0.41	(-1.01-0.23)	2.02E-01	2.89E-01	0.50	(-1.13-2.16)	5.49E-01	6.78E-01
445	PE(P-18:0/22:5) (n6)	-1.29	(-1.87--0.72)	**1.35E-05**	**5.30E-05**	-1.25	(-1.83--0.67)	**3.16E-05**	**8.48E-05**	0.36	(-0.27-1.04)	2.67E-01	3.62E-01	-0.44	(-2.36-1.51)	6.54E-01	7.51E-01
446	PE(P-18:0/22:6)	-0.79	(-1.43--0.14)	**1.80E-02**	**3.18E-02**	-1.21	(-1.87--0.55)	**3.71E-04**	**8.65E-04**	-0.06	(-0.69-0.62)	8.57E-01	9.01E-01	0.00	(-1.8-1.82)	9.98E-01	1.00E+00
447	PE(P-18:1/18:1)	-0.90	(-1.45--0.35)	**1.43E-03**	**3.50E-03**	-0.83	(-1.38--0.28)	**3.06E-03**	**5.83E-03**	0.36	(-0.26-1.02)	2.58E-01	3.53E-01	-0.47	(-1.9-0.98)	5.21E-01	6.50E-01
448	PE(P-18:1/18:2)	-1.86	(-2.56--1.16)	**3.27E-07**	**1.80E-06**	-1.22	(-1.9--0.53)	**5.96E-04**	**1.30E-03**	-0.63	(-1.73-0.61)	3.01E-01	3.97E-01	-0.76	(-2.77-1.29)	4.62E-01	6.05E-01
449	PE(P-18:1/20:4)	-0.06	(-0.81-0.71)	8.85E-01	9.02E-01	-0.43	(-1.13-0.27)	2.28E-01	2.83E-01	-0.55	(-1.23-0.19)	1.40E-01	2.14E-01	1.88	(-0.24-4.04)	8.28E-02	1.76E-01
450	PE(P-18:1/20:5)	-1.57	(-2.88--0.25)	**2.01E-02**	**3.52E-02**	-0.22	(-1.51-1.08)	7.37E-01	7.75E-01	-0.82	(-2.78-1.69)	4.88E-01	5.80E-01	3.76	(0.1-7.55)	**4.42E-02**	1.14E-01
451	PE(P-18:1/22:4)	-0.28	(-0.92-0.37)	4.02E-01	4.62E-01	-0.99	(-1.63--0.35)	**2.44E-03**	**4.77E-03**	0.33	(-0.55-1.3)	4.70E-01	5.68E-01	-1.15	(-2.57-0.28)	1.14E-01	2.16E-01
452	PE(P-18:1/22:5) (a)	-0.71	(-1.33--0.09)	**2.54E-02**	**4.35E-02**	-0.39	(-0.96-0.18)	1.83E-01	2.33E-01	-0.38	(-1-0.29)	2.55E-01	3.51E-01	0.91	(-0.84-2.69)	3.07E-01	4.66E-01
453	PE(P-18:1/22:6) (a)	-0.50	(-1.16-0.15)	1.32E-01	1.81E-01	-0.66	(-1.33-0.02)	5.55E-02	7.90E-02	-0.05	(-0.73-0.67)	8.79E-01	9.09E-01	0.85	(-1.04-2.77)	3.80E-01	5.29E-01
454	PE(P-20:0/18:1)	-1.27	(-1.89--0.65)	**7.01E-05**	**2.35E-04**	-1.14	(-1.71--0.57)	**1.01E-04**	**2.52E-04**	0.52	(-1.11-2.44)	5.56E-01	6.45E-01	-1.09	(-2.82-0.67)	2.22E-01	3.67E-01
455	PE(P-20:0/18:2)	-1.81	(-2.36--1.25)	**3.40E-10**	**4.18E-09**	-1.98	(-2.5--1.45)	**1.04E-12**	**9.79E-12**	-0.03	(-0.46-0.42)	9.01E-01	9.28E-01	-2.72	(-4.31--1.11)	**1.09E-03**	**6.15E-03**
456	PE(P-20:0/20:4)	-0.17	(-0.76-0.43)	5.85E-01	6.36E-01	-1.62	(-2.15--1.08)	**6.16E-09**	**2.90E-08**	-0.61	(-1.46-0.34)	2.00E-01	2.87E-01	-0.21	(-1.81-1.41)	7.97E-01	8.73E-01
457	PE(P-20:0/22:6)	-0.78	(-1.31--0.26)	**3.71E-03**	**8.27E-03**	-1.89	(-2.39--1.4)	**3.83E-13**	**3.92E-12**	-0.70	(-1.25--0.11)	**2.13E-02**	**4.05E-02**	-1.79	(-3.28--0.27)	**2.13E-02**	6.63E-02
458	PE(P-20:1/20:4)	-0.41	(-1.28-0.47)	3.64E-01	4.27E-01	-1.06	(-2.02--0.09)	**3.22E-02**	**4.84E-02**	-0.82	(-1.84-0.32)	1.51E-01	2.29E-01	0.93	(-1.28-3.19)	4.10E-01	5.58E-01
459	PG(34:2)	-1.07	(-1.82--0.31)	**6.14E-03**	**1.28E-02**	1.17	(0.21-2.14)	**1.72E-02**	**2.76E-02**	-0.67	(-1.86-0.7)	3.23E-01	4.21E-01	2.03	(-0.75-4.88)	1.53E-01	2.80E-01
460	PG(36:1)	-0.28	(-0.94-0.38)	4.00E-01	4.62E-01	2.52	(1.6-3.45)	**8.86E-08**	**3.52E-07**	-1.38	(-2.5--0.09)	**3.65E-02**	6.63E-02	2.63	(-0.01-5.33)	5.10E-02	1.27E-01
461	PI(16:0/16:0)	-2.44	(-3.37--1.51)	**4.46E-07**	**2.38E-06**	1.75	(0.7-2.81)	**1.12E-03**	**2.36E-03**	-0.09	(-1.03-0.93)	8.50E-01	8.99E-01	3.09	(0.46-5.79)	**2.10E-02**	6.58E-02
462	PI(16:0_16:1)	-1.73	(-2.79--0.66)	**1.69E-03**	**4.01E-03**	3.85	(2.77-4.94)	**3.76E-12**	**3.11E-11**	0.72	(-0.12-1.62)	9.40E-02	1.53E-01	8.42	(5.56-11.34)	**6.42E-09**	**1.03E-06**
463	PI(34:0)	-2.66	(-3.58--1.72)	**4.56E-08**	**2.96E-07**	1.08	(0-2.17)	**4.90E-02**	7.13E-02	-1.86	(-2.99--0.54)	**7.41E-03**	**1.58E-02**	-0.02	(-3.05-3.11)	9.90E-01	9.96E-01
464	PI(34:1)	-2.01	(-2.68--1.34)	**8.14E-09**	**5.75E-08**	0.20	(-0.53-0.93)	5.92E-01	6.47E-01	-0.19	(-0.81-0.47)	5.56E-01	6.45E-01	0.45	(-1.4-2.34)	6.33E-01	7.42E-01
465	PI(17:0_18:1)	-2.37	(-2.95--1.79)	**6.39E-15**	**2.79E-13**	0.44	(-0.29-1.18)	2.37E-01	2.93E-01	-1.30	(-2.26--0.22)	**1.94E-02**	**3.75E-02**	-0.77	(-2.94-1.45)	4.91E-01	6.31E-01
466	PI(17:0_18:2)	-2.70	(-3.33--2.07)	**4.72E-16**	**2.83E-14**	0.69	(-0.03-1.41)	6.05E-02	8.54E-02	-1.91	(-2.87--0.83)	**9.67E-04**	**2.37E-03**	0.15	(-1.85-2.18)	8.85E-01	9.42E-01
467	PI(18:0_18:1)	-1.94	(-2.65--1.23)	**1.16E-07**	**7.23E-07**	-0.74	(-1.49-0.01)	5.26E-02	7.58E-02	-1.18	(-1.84--0.46)	**1.71E-03**	**4.02E-03**	-2.01	(-4.12-0.14)	6.70E-02	1.52E-01
468	PI(36:2)	-1.93	(-2.51--1.34)	**2.53E-10**	**3.28E-09**	-0.30	(-0.88-0.3)	3.26E-01	3.83E-01	-1.22	(-1.78--0.62)	**1.28E-04**	**3.56E-04**	-1.49	(-3.08-0.13)	7.12E-02	1.58E-01
469	PI(18:1_18:2)	-2.66	(-3.29--2.03)	**1.11E-15**	**5.91E-14**	-1.29	(-1.97--0.61)	**2.16E-04**	**5.17E-04**	-1.47	(-1.98--0.93)	**4.93E-07**	**1.78E-06**	-3.22	(-4.99--1.41)	**5.62E-04**	**3.65E-03**
470	PI(16:0_20:3) (a)	-2.16	(-2.82--1.5)	**3.96E-10**	**4.64E-09**	0.93	(0.22-1.65)	**1.07E-02**	**1.78E-02**	0.22	(-0.31-0.79)	4.19E-01	5.18E-01	-0.04	(-1.72-1.66)	9.62E-01	9.85E-01
471	PI(16:0_20:3) (b)	-2.46	(-3.2--1.72)	**1.99E-10**	**2.80E-09**	0.86	(0.13-1.6)	**2.06E-02**	**3.21E-02**	2.99	(1.94-4.14)	**2.09E-09**	**9.74E-09**	0.67	(-1.27-2.64)	5.00E-01	6.34E-01
472	PI(16:0_20:4)	-1.45	(-2.03--0.86)	**1.88E-06**	**8.78E-06**	0.99	(0.36-1.62)	**2.13E-03**	**4.20E-03**	0.12	(-0.42-0.7)	6.69E-01	7.50E-01	1.36	(-0.32-3.07)	1.12E-01	2.14E-01
473	PI(18:0_20:2)	-2.66	(-3.36--1.95)	**6.56E-13**	**1.85E-11**	-0.07	(-0.8-0.66)	8.50E-01	8.75E-01	2.45	(1.2-3.85)	**5.83E-05**	**1.70E-04**	-2.45	(-4.37--0.49)	**1.48E-02**	5.00E-02

474	PI(18:0_20:3) (a)	-0.92	(-1.42--0.41)	**4.19E-04**	**1.18E-03**	-0.19	(-0.7-0.31)	4.57E-01	5.16E-01	-0.28	(-0.75-0.22)	2.65E-01	3.60E-01	-1.02	(-2.35-0.32)	1.33E-01	2.48E-01
475	PI(18:0_20:4)	-0.79	(-1.31--0.28)	**2.68E-03**	**6.09E-03**	-0.13	(-0.63-0.37)	6.01E-01	6.56E-01	-0.59	(-1.01--0.14)	**1.08E-02**	**2.19E-02**	-1.18	(-2.55-0.2)	9.21E-02	1.88E-01
476	PI(38:5) (a)	-2.43	(-2.97--1.88)	**5.10E-17**	**4.08E-15**	-0.27	(-0.84-0.3)	3.46E-01	4.00E-01	-0.59	(-1.16-0.02)	5.72E-02	9.95E-02	-1.02	(-2.47-0.45)	1.73E-01	3.06E-01
477	PI(38:6)	-2.63	(-3.27--1.98)	**1.30E-14**	**5.19E-13**	-0.89	(-1.56--0.21)	**1.01E-02**	**1.70E-02**	-0.92	(-1.44--0.37)	**1.31E-03**	**3.11E-03**	-0.06	(-1.76-1.66)	9.42E-01	9.74E-01
478	PI(18:0_22:4)	-1.24	(-1.89--0.58)	**2.27E-04**	**6.78E-04**	0.25	(-0.38-0.9)	4.35E-01	4.92E-01	1.20	(0.52-1.92)	**4.03E-04**	**1.05E-03**	-0.81	(-2.49-0.89)	3.47E-01	5.03E-01
479	PI(18:0_22:5) (n3)	-1.73	(-2.4--1.06)	**5.69E-07**	**2.91E-06**	0.86	(0.2-1.52)	**1.06E-02**	**1.78E-02**	-1.24	(-1.88--0.56)	**5.87E-04**	**1.47E-03**	0.63	(-1.16-2.45)	4.93E-01	6.31E-01
480	PI(18:0_22:6)	-2.03	(-2.68--1.38)	**2.31E-09**	**2.05E-08**	-0.63	(-1.3-0.05)	6.94E-02	9.71E-02	-1.11	(-1.63--0.55)	**1.59E-04**	**4.30E-04**	-0.13	(-1.98-1.75)	8.89E-01	9.44E-01

Table S2C. Association of antenatal lipidomic profiles with antenatal BMI

Studies		Antenatal BMI			
Covariates		adjusted for ethnicity, maternal age and maternal education level			
No.	Lipid Species	%change in lipid concentration per unit BMI	95%CI	p-value	p-value(BH)
1	AC(12:0)	0.49	(-0.50- 1.50)	3.30E-01	3.94E-01
2	AC(13:0)	1.28	(0.01- 2.56)	**4.79E-02**	8.05E-02
3	AC(14:0)	0.45	(-0.20- 1.12)	1.76E-01	2.33E-01
4	AC(14:1)	1.43	(0.34- 2.54)	**1.04E-02**	**2.12E-02**
5	AC(14:2)	1.09	(0.05- 2.14)	**4.02E-02**	6.96E-02
6	AC(16:0)	0.37	(-0.11- 0.86)	1.33E-01	1.86E-01
7	AC(16:1)	1.22	(0.44- 2.01)	**2.06E-03**	**5.13E-03**
8	AC(18:0)	-0.09	(-0.59- 0.40)	7.15E-01	7.73E-01
9	AC(18:1)	1.25	(0.59- 1.92)	**2.15E-04**	**6.73E-04**
10	AC(18:2)	0.86	(0.22- 1.51)	**8.72E-03**	**1.81E-02**
11	CE(14:0)	-1.76	(-2.29--1.23)	**1.93E-10**	**2.65E-09**
12	CE(16:1)	-0.73	(-1.46- 0.00)	5.07E-02	8.44E-02
13	CE(18:0)	-1.04	(-1.53--0.55)	**3.22E-05**	**1.24E-04**
14	CE(18:1)	-0.86	(-1.15--0.57)	**1.10E-08**	**8.35E-08**
15	CE(18:2)	-0.79	(-1.12--0.45)	**4.07E-06**	**1.95E-05**
16	CE(18:3)	-1.70	(-2.28--1.10)	**3.30E-08**	**2.20E-07**
17	CE(20:1)	-0.83	(-1.26--0.40)	**1.92E-04**	**6.13E-04**
18	CE(20:2)	-1.21	(-1.60--0.81)	**3.72E-09**	**3.19E-08**
19	CE(20:4)	-0.63	(-1.23--0.02)	**4.21E-02**	7.20E-02
20	CE(20:5)	-1.44	(-2.70--0.16)	**2.74E-02**	**4.96E-02**
21	CE(22:5) (n6)	-1.82	(-2.37--1.26)	**4.43E-10**	**5.24E-09**
22	CE(22:6)	-1.50	(-2.17--0.81)	**2.05E-05**	**8.21E-05**
23	CE(24:5)	-1.96	(-2.63--1.29)	**1.94E-08**	**1.35E-07**
24	CE(24:6)	-2.36	(-3.05--1.67)	**6.50E-11**	**1.04E-09**
25	DG(16:0_16:1)	0.72	(-0.25- 1.69)	1.45E-01	1.99E-01
26	DG(14:0_18:2)	-0.96	(-1.90--0.02)	**4.62E-02**	7.81E-02

27	DG(16:0_18:1)	1.22	(0.52- 1.92)	**6.17E-04**	**1.76E-03**
28	DG(16:1_18:1)	0.64	(-0.17- 1.46)	1.21E-01	1.73E-01
29	DG(16:0_18:2)	0.83	(0.16- 1.51)	**1.59E-02**	**3.06E-02**
30	DG(18:0_18:1)	1.22	(0.58- 1.86)	**1.73E-04**	**5.66E-04**
31	DG(18:1_18:1)	1.06	(0.37- 1.76)	**2.51E-03**	**6.17E-03**
32	DG(18:0_18:2)	0.92	(0.21- 1.64)	**1.10E-02**	**2.23E-02**
33	DG(18:1_18:2)	0.64	(-0.05- 1.34)	6.83E-02	1.08E-01
34	DG(18:2_18:2)	0.57	(-0.32- 1.46)	2.10E-01	2.74E-01
35	DG(18:1_18:3)	0.17	(-0.57- 0.91)	6.60E-01	7.21E-01
36	DG(16:0_20:4)	1.22	(0.34- 2.11)	**6.58E-03**	**1.45E-02**
37	DG(18:1_20:3)	0.45	(-0.27- 1.17)	2.22E-01	2.86E-01
38	DG(18:1_20:4)	1.01	(0.30- 1.72)	**5.34E-03**	**1.22E-02**
39	DG(16:0_22:5)	0.54	(-0.29- 1.38)	2.00E-01	2.63E-01
40	DG(18:2_20:4)	0.65	(-0.16- 1.47)	1.17E-01	1.68E-01
41	DG(16:0_22:6)	1.28	(0.22- 2.36)	**1.82E-02**	**3.42E-02**
42	DG(18:1_22:5)	0.25	(-0.54- 1.04)	5.41E-01	6.11E-01
43	DG(18:1_22:6)	1.42	(0.41- 2.44)	**5.72E-03**	**1.29E-02**
44	DG(18:2_22:6)	1.39	(0.34- 2.45)	**9.25E-03**	**1.91E-02**
45	TG(48:0) [NL-18:0]	-1.36	(-2.19--0.52)	**1.56E-03**	**3.97E-03**
46	TG(48:1) [NL-16:1]	0.79	(-0.28- 1.86)	1.49E-01	2.02E-01
47	TG(48:1) [NL-18:1]	-0.47	(-1.30- 0.36)	2.65E-01	3.28E-01
48	TG(48:2) [NL-14:0]	-0.80	(-1.77- 0.19)	1.12E-01	1.62E-01
49	TG(48:2) [NL-14:1]	-0.98	(-1.98- 0.03)	5.60E-02	9.20E-02
50	TG(48:2) [NL-16:1]	0.02	(-1.21- 1.26)	9.81E-01	9.87E-01
51	TG(48:2) [NL-18:2]	-1.03	(-2.00--0.04)	**4.10E-02**	7.02E-02
52	TG(48:3) [NL-14:0]	-1.30	(-2.36--0.22)	**1.81E-02**	**3.42E-02**
53	TG(48:3) [NL-16:1]	-1.01	(-2.08- 0.08)	7.04E-02	1.10E-01
54	TG(48:3) [NL-18:3]	-1.57	(-2.77--0.35)	**1.18E-02**	**2.35E-02**
55	TG(49:1) [NL-17:1]	0.51	(-0.31- 1.34)	2.24E-01	2.86E-01
56	TG(50:1) [NL-14:0]	-0.63	(-1.35- 0.09)	8.73E-02	1.31E-01
57	TG(50:1) [NL-16:0]	0.91	(0.34- 1.48)	**1.82E-03**	**4.57E-03**
58	TG(50:1) [NL-18:1]	0.79	(0.25- 1.34)	**4.35E-03**	**1.00E-02**
59	TG(50:2) [NL-14:0]	-0.67	(-1.20--0.13)	**1.48E-02**	**2.87E-02**

60	TG(50:2) [NL-16:1]	0.62	(-0.05- 1.30)	6.93E-02	1.09E-01
61	TG(50:2) [NL-18:1]	0.30	(-0.29- 0.89)	3.19E-01	3.82E-01
62	TG(50:2) [NL-18:2]	0.70	(0.15- 1.25)	**1.24E-02**	**2.47E-02**
63	TG(50:3) [NL-14:0]	-0.59	(-1.27- 0.10)	9.24E-02	1.36E-01
64	TG(50:3) [NL-14:1]	-0.52	(-1.13- 0.10)	1.03E-01	1.50E-01
65	TG(50:3) [NL-16:1]	0.56	(-0.22- 1.34)	1.60E-01	2.16E-01
66	TG(50:3) [NL-18:2]	0.19	(-0.50- 0.89)	5.87E-01	6.54E-01
67	TG(50:3) [NL-18:3]	0.33	(-0.54- 1.21)	4.63E-01	5.28E-01
68	TG(50:4) [NL-14:0]	-0.87	(-1.83- 0.09)	7.51E-02	1.16E-01
69	TG(50:4) [NL-18:3]	-0.63	(-1.61- 0.36)	2.10E-01	2.74E-01
70	TG(50:4) [NL-20:4]	-0.65	(-1.95- 0.67)	3.32E-01	3.95E-01
71	TG(51:1) [NL-17:0]	0.63	(-0.07- 1.35)	7.91E-02	1.20E-01
72	TG(51:2) [NL-15:0]	-0.67	(-1.30--0.05)	**3.57E-02**	6.26E-02
73	TG(51:2) [NL-17:0]	0.36	(-0.28- 1.02)	2.71E-01	3.31E-01
74	TG(51:2) [NL-17:1]	0.36	(-0.27- 0.99)	2.66E-01	3.28E-01
75	TG(52:1) [NL-18:0]	1.42	(0.67- 2.17)	**1.98E-04**	**6.24E-04**
76	TG(52:1) [NL-18:1]	1.47	(0.75- 2.19)	**6.59E-05**	**2.40E-04**
77	TG(52:2) [NL-16:0]	0.63	(0.25- 1.02)	**1.26E-03**	**3.25E-03**
78	TG(52:2) [NL-18:2]	0.56	(-0.02- 1.14)	5.71E-02	9.29E-02
79	TG(52:3) [NL-16:1]	0.24	(-0.30- 0.78)	3.93E-01	4.54E-01
80	TG(52:3) [NL-18:2]	0.57	(0.15- 1.00)	**8.11E-03**	**1.70E-02**
81	TG(52:4) [NL-16:1]	0.48	(-0.21- 1.17)	1.71E-01	2.28E-01
82	TG(52:4) [NL-18:2]	0.96	(0.24- 1.69)	**9.34E-03**	**1.92E-02**
83	TG(52:4) [NL-18:3]	0.37	(-0.30- 1.04)	2.77E-01	3.37E-01
84	TG(52:5) [NL-18:3]	0.02	(-0.92- 0.97)	9.60E-01	9.72E-01
85	TG(52:5) [NL-20:4]	0.56	(-0.56- 1.70)	3.30E-01	3.94E-01
86	TG(52:5) [NL-20:5]	1.08	(-0.13- 2.31)	8.11E-02	1.22E-01
87	TG(53:2) [NL-18:1]	0.68	(0.04- 1.33)	**3.72E-02**	6.49E-02
88	TG(54:1) [NL-18:1]	0.87	(0.24- 1.51)	**6.65E-03**	**1.46E-02**
89	TG(54:2) [NL-18:0]	1.30	(0.64- 1.95)	**1.01E-04**	**3.50E-04**
90	TG(54:2) [NL-20:1]	0.28	(-0.46- 1.02)	4.57E-01	5.22E-01
91	TG(54:3) [NL-18:1]	0.29	(-0.24- 0.83)	2.83E-01	3.43E-01
92	TG(54:3) [NL-18:2]	0.60	(0.05- 1.14)	**3.19E-02**	5.63E-02

93	TG(54:4) [NL-18:2]	0.11	(-0.49- 0.71)	7.27E-01	7.82E-01
94	TG(54:4) [NL-20:3]	0.66	(0.01- 1.31)	**4.66E-02**	7.84E-02
95	TG(54:5) [NL-18:3]	-0.12	(-0.94- 0.70)	7.70E-01	8.21E-01
96	TG(54:5) [NL-20:4]	0.96	(0.33- 1.59)	**2.65E-03**	**6.45E-03**
97	TG(54:6) [NL-18:3]	-0.60	(-1.69- 0.49)	2.80E-01	3.39E-01
98	TG(54:6) [NL-20:4]	1.11	(0.32- 1.91)	**5.77E-03**	**1.30E-02**
99	TG(54:6) [NL-20:5]	1.00	(-0.09- 2.09)	7.14E-02	1.11E-01
100	TG(54:6) [NL-22:6]	1.98	(0.83- 3.15)	**7.51E-04**	**2.08E-03**
101	TG(54:7) [NL-20:5]	0.65	(-0.57- 1.89)	2.96E-01	3.57E-01
102	TG(54:7) [NL-22:6]	1.13	(-0.13- 2.39)	7.81E-02	1.19E-01
103	TG(56:6) [NL-20:4]	1.20	(0.69- 1.71)	**3.67E-06**	**1.82E-05**
104	TG(56:6) [NL-22:5]	0.56	(-0.07- 1.18)	8.11E-02	1.22E-01
105	TG(56:7) [NL-20:4]	0.87	(0.23- 1.52)	**7.69E-03**	**1.63E-02**
106	TG(56:7) [NL-20:5]	0.46	(-0.59- 1.52)	3.89E-01	4.51E-01
107	TG(56:7) [NL-22:5]	0.71	(-0.02- 1.45)	5.82E-02	9.34E-02
108	TG(56:7) [NL-22:6]	1.96	(0.93- 3.00)	**1.94E-04**	**6.16E-04**
109	TG(56:8) [NL-20:4]	0.74	(-0.14- 1.64)	9.96E-02	1.46E-01
110	TG(56:8) [NL-20:5]	0.28	(-0.90- 1.47)	6.47E-01	7.09E-01
111	TG(56:8) [NL-22:6]	1.74	(0.59- 2.91)	**3.07E-03**	**7.27E-03**
112	TG(56:9) [NL-22:6]	0.92	(-0.21- 2.05)	1.10E-01	1.59E-01
113	TG(58:10) [NL-22:6]	0.88	(-0.23- 2.01)	1.21E-01	1.73E-01
114	TG(58:8) [NL-22:6]	1.42	(0.39- 2.45)	**6.55E-03**	**1.45E-02**
115	TG(58:9) [NL-22:6]	1.22	(0.13- 2.32)	**2.87E-02**	5.13E-02
116	TG(O-50:1) [NL-16:0]	-0.05	(-0.61- 0.52)	8.74E-01	9.08E-01
117	TG(O-50:1) [NL-17:1]	0.51	(-0.31- 1.35)	2.24E-01	2.86E-01
118	TG(O-50:1) [NL-18:1]	-0.48	(-1.10- 0.16)	1.40E-01	1.94E-01
119	TG(O-50:2) [NL-18:2]	-0.03	(-0.58- 0.53)	9.24E-01	9.44E-01
120	TG(O-52:2) [NL-16:0]	-0.09	(-0.85- 0.67)	8.11E-01	8.54E-01
121	TG(O-52:2) [NL-17:1]	0.36	(-0.27- 0.99)	2.68E-01	3.30E-01
122	TG(O-52:2) [NL-18:1]	-0.35	(-1.13- 0.44)	3.83E-01	4.45E-01
123	TG(O-54:2) [NL-18:1]	0.66	(0.02- 1.30)	**4.41E-02**	7.51E-02
124	TG(O-54:4) [NL-17:1]	-0.07	(-0.80- 0.66)	8.43E-01	8.83E-01
125	TG(O-54:4) [NL-18:2]	-0.11	(-0.87- 0.66)	7.82E-01	8.32E-01

126	dhCer(d18:0/22:0)	1.17	(0.57- 1.78)	**1.51E-04**	**5.00E-04**
127	dhCer(d18:0/24:0)	0.32	(-0.24- 0.89)	2.66E-01	3.28E-01
128	dhCer(d18:0/24:1)	1.68	(1.01- 2.36)	**9.12E-07**	**4.92E-06**
129	Cer(d16:1/22:0)	-0.55	(-1.12- 0.02)	6.04E-02	9.67E-02
130	Cer(d16:1/23:0)	-0.82	(-1.46--0.19)	**1.15E-02**	**2.31E-02**
131	Cer(d16:1/24:0)	-1.86	(-2.45--1.26)	**2.60E-09**	**2.35E-08**
132	Cer(d16:1/24:1)	-0.17	(-0.76- 0.43)	5.86E-01	6.54E-01
133	Cer(d17:1/22:0)	-1.12	(-1.73--0.50)	**3.95E-04**	**1.18E-03**
134	Cer(d17:1/23:0)	-1.10	(-1.74--0.45)	**9.38E-04**	**2.53E-03**
135	Cer(d17:1/24:0)	-2.00	(-2.55--1.45)	**3.43E-12**	**8.23E-11**
136	Cer(d17:1/24:1)	-1.06	(-1.65--0.47)	**4.22E-04**	**1.24E-03**
137	Cer(d18:1/16:0)	-0.73	(-1.16--0.29)	**1.20E-03**	**3.14E-03**
138	Cer(d18:1/18:0)	2.06	(1.42- 2.69)	**2.38E-10**	**3.14E-09**
139	Cer(d18:1/20:0)	0.35	(-0.18- 0.89)	1.95E-01	2.57E-01
140	Cer(d18:1/22:0)	0.03	(-0.42- 0.48)	9.07E-01	9.30E-01
141	Cer(d18:1/23:0)	-0.31	(-0.76- 0.15)	1.91E-01	2.52E-01
142	Cer(d18:1/24:0)	-0.90	(-1.34--0.46)	**7.06E-05**	**2.55E-04**
143	Cer(d18:1/24:1)	0.13	(-0.38- 0.64)	6.12E-01	6.77E-01
144	Cer(d18:2/16:0)	-0.19	(-0.78- 0.41)	5.31E-01	6.01E-01
145	Cer(d18:2/22:0)	-0.24	(-0.78- 0.29)	3.75E-01	4.37E-01
146	Cer(d18:2/23:0)	-0.42	(-0.98- 0.14)	1.44E-01	1.98E-01
147	Cer(d18:2/24:0)	-1.18	(-1.70--0.66)	**1.13E-05**	**4.82E-05**
148	Cer(d18:2/24:1)	0.02	(-0.53- 0.57)	9.54E-01	9.70E-01
149	Cer(d19:1/24:0)	-1.18	(-2.03--0.33)	**6.84E-03**	**1.49E-02**
150	Cer(d19:1/24:1)	-0.24	(-1.23- 0.75)	6.30E-01	6.95E-01
151	Cer(m18:0/22:0)	0.36	(-0.40- 1.12)	3.51E-01	4.14E-01
152	Cer(m18:0/23:0)	0.52	(-0.31- 1.36)	2.19E-01	2.84E-01
153	Cer(m18:0/24:0)	-0.42	(-1.17- 0.34)	2.77E-01	3.37E-01
154	Cer(m18:0/24:1)	1.23	(0.39- 2.08)	**4.05E-03**	**9.38E-03**
155	Cer(m18:1/22:0)	0.56	(-0.25- 1.37)	1.73E-01	2.30E-01
156	Cer(m18:1/23:0)	0.17	(-0.69- 1.03)	7.02E-01	7.62E-01
157	Cer(m18:1/24:0)	-0.58	(-1.32- 0.17)	1.31E-01	1.84E-01
158	Cer(m18:1/24:1)	1.15	(0.31- 2.00)	**7.18E-03**	**1.54E-02**

159	GM3(d18:1/18:0)	-1.01	(-1.51--0.50)	**1.09E-04**	**3.69E-04**
160	GM3(d18:1/22:0)	-2.05	(-2.63--1.47)	**1.54E-11**	**2.96E-10**
161	GM3(d18:1/24:0)	-3.09	(-3.67--2.51)	**3.54E-23**	**8.49E-21**
162	GM3(d18:1/24:1)	-1.70	(-2.26--1.14)	**4.39E-09**	**3.70E-08**
163	HexCer(d16:1/22:0)	-1.66	(-2.26--1.05)	**1.12E-07**	**6.87E-07**
164	HexCer(d16:1/24:0)	-3.08	(-3.70--2.46)	**9.70E-21**	**9.31E-19**
165	HexCer(d18:1/16:0)	-1.29	(-1.75--0.83)	**6.17E-08**	**3.97E-07**
166	HexCer(d18:1/18:0)	-1.47	(-2.03--0.91)	**3.56E-07**	**2.06E-06**
167	HexCer(d18:1/20:0)	-1.84	(-2.37--1.31)	**3.17E-11**	**5.85E-10**
168	HexCer(d18:1/22:0)	-1.91	(-2.37--1.44)	**6.16E-15**	**2.69E-13**
169	HexCer(d18:1/24:0)	-2.58	(-3.05--2.11)	**1.77E-24**	**8.48E-22**
170	HexCer(d18:1/24:1)	-1.70	(-2.22--1.17)	**5.53E-10**	**6.33E-09**
171	Hex2Cer(d16:1/16:0)	-1.19	(-1.78--0.60)	**8.56E-05**	**3.07E-04**
172	Hex2Cer(d18:1/16:0)	-1.53	(-2.03--1.04)	**2.47E-09**	**2.28E-08**
173	Hex2Cer(d18:1/22:0)	-1.69	(-2.21--1.17)	**4.47E-10**	**5.24E-09**
174	Hex2Cer(d18:1/24:1)	-1.63	(-2.19--1.08)	**1.53E-08**	**1.10E-07**
175	Hex2Cer(d18:2/16:0)	-0.74	(-1.26--0.22)	**5.12E-03**	**1.17E-02**
176	Hex3Cer(d18:1/16:0)	-1.08	(-1.47--0.68)	**1.34E-07**	**8.16E-07**
177	Hex3Cer(d18:1/22:0)	-1.49	(-2.06--0.92)	**4.93E-07**	**2.75E-06**
178	Hex3Cer(d18:1/24:0)	-1.62	(-2.16--1.07)	**1.04E-08**	**8.05E-08**
179	Hex3Cer(d18:1/24:1)	-1.20	(-1.72--0.68)	**8.14E-06**	**3.55E-05**
180	SM(d17:1/14:0)	-1.71	(-2.25--1.17)	**1.07E-09**	**1.11E-08**
181	SM(d18:0/14:0)	-0.71	(-1.21--0.21)	**5.93E-03**	**1.33E-02**
182	SM(d18:1/14:0)/SM(d16:1/16:0)	-0.82	(-1.27--0.37)	**3.86E-04**	**1.16E-03**
183	SM(d18:2/14:0)	0.35	(-0.14- 0.84)	1.62E-01	2.18E-01
184	SM(d17:1/16:0)	-1.29	(-1.72--0.86)	**5.52E-09**	**4.57E-08**
185	SM(d18:1/16:0)	-1.33	(-1.84--0.82)	**4.31E-07**	**2.44E-06**
186	SM(d18:2/16:0)	0.16	(-0.22- 0.54)	4.09E-01	4.71E-01
187	SM(34:3)	0.37	(-0.08- 0.82)	1.09E-01	1.59E-01
188	SM(d18:2/17:0)	-0.25	(-0.69- 0.18)	2.53E-01	3.17E-01
189	SM(35:2) (b)	-1.39	(-1.94--0.83)	**1.32E-06**	**7.02E-06**
190	SM(d18:1/18:0)/SM(d16:1/20:0)	0.38	(-0.10- 0.87)	1.22E-01	1.73E-01
191	SM(d18:2/18:1)	0.44	(-0.01- 0.89)	5.67E-02	9.29E-02

192	SM(37:1)	-0.65	(-1.17--0.13)	**1.39E-02**	**2.74E-02**
193	SM(37:2)	-1.00	(-1.58--0.43)	**6.68E-04**	**1.90E-03**
194	SM(d18:1/20:0)/SM(d16:1/22:0)	-0.67	(-1.06--0.27)	**1.15E-03**	**3.03E-03**
195	SM(d18:2/20:0)	-0.28	(-0.76- 0.20)	2.47E-01	3.11E-01
196	SM(38:3) (a)	0.56	(0.09- 1.03)	**2.01E-02**	**3.73E-02**
197	SM(38:3) (b)	-0.63	(-1.15--0.10)	**1.98E-02**	**3.70E-02**
198	SM(d16:1/23:0)/SM(d17:1/22:0)	-0.96	(-1.40--0.52)	**2.37E-05**	**9.27E-05**
199	SM(d18:1/22:0)/SM(d16:1/24:0)	-0.54	(-0.99--0.10)	**1.59E-02**	**3.06E-02**
200	SM(d16:1/24:1)	-0.58	(-1.06--0.10)	**1.72E-02**	**3.26E-02**
201	SM(d18:2/22:0)	-0.70	(-1.13--0.26)	**1.83E-03**	**4.57E-03**
202	SM(40:3) (a)	-0.01	(-0.47- 0.46)	9.76E-01	9.84E-01
203	SM(40:3) (b)	-0.77	(-1.29--0.25)	**3.79E-03**	**8.88E-03**
204	SM(41:0)	-0.37	(-0.77- 0.04)	7.80E-02	1.19E-01
205	SM(41:1)	-0.63	(-1.03--0.22)	**2.50E-03**	**6.17E-03**
206	SM(d17:1/24:1)	-0.84	(-1.27--0.41)	**1.47E-04**	**4.89E-04**
207	SM(d18:2/23:0)	-0.86	(-1.27--0.45)	**4.75E-05**	**1.78E-04**
208	SM(d18:1/24:0)	-1.44	(-1.87--1.00)	**1.67E-10**	**2.39E-09**
209	SM(d18:1/24:1)	-0.55	(-1.06--0.04)	**3.34E-02**	5.87E-02
210	SM(d18:2/24:0)	-1.43	(-1.83--1.04)	**2.62E-12**	**6.63E-11**
211	SM(43:1)	-1.60	(-2.05--1.15)	**6.52E-12**	**1.42E-10**
212	SM(44:1)	-1.57	(-1.97--1.18)	**2.98E-14**	**1.19E-12**
213	SM(44:2)	-1.12	(-1.58--0.65)	**3.68E-06**	**1.82E-05**
214	SM(44:3) (a)	-1.38	(-1.82--0.94)	**1.42E-09**	**1.42E-08**
215	LPC(14:0) [sn2]	-0.46	(-1.20- 0.28)	2.22E-01	2.86E-01
216	LPC(14:0) [sn1]	-0.19	(-0.91- 0.52)	5.95E-01	6.62E-01
217	LPC(15:0) [sn2]	-1.36	(-1.94--0.77)	**7.01E-06**	**3.11E-05**
218	LPC(15:0) [sn1]	-1.15	(-1.68--0.62)	**2.41E-05**	**9.34E-05**
219	LPC(16:0) [sn2]	0.07	(-0.36- 0.49)	7.57E-01	8.11E-01
220	LPC(16:0) [sn1]	0.31	(-0.19- 0.81)	2.25E-01	2.86E-01
221	LPC(16:1) [sn2]	0.39	(-0.30- 1.08)	2.70E-01	3.30E-01
222	LPC(16:1) [sn1]	0.52	(-0.08- 1.12)	9.12E-02	1.36E-01
223	LPC(15-MHDA) [sn2]	-1.37	(-2.07--0.66)	**1.82E-04**	**5.86E-04**
224	LPC(15-MHDA) [sn1] / LPC(17:0) [sn2]	-1.20	(-1.79--0.60)	**1.06E-04**	**3.64E-04**

225	LPC(17:0) [sn1]	-0.87	(-1.35--0.40)	**3.09E-04**	**9.45E-04**
226	LPC(17:1) [sn1] (a) / LPC(17:1) [sn2] (b)	-0.82	(-1.37--0.27)	**3.48E-03**	**8.20E-03**
227	LPC(18:0) [sn2]	0.00	(-0.44- 0.44)	9.99E-01	9.99E-01
228	LPC(18:0) [sn1]	0.03	(-0.42- 0.48)	8.87E-01	9.16E-01
229	LPC(18:1) [sn2]	-0.48	(-0.98- 0.01)	5.70E-02	9.29E-02
230	LPC(18:1) [sn1]	-0.38	(-0.82- 0.06)	9.10E-02	1.36E-01
231	LPC(18:2) [sn2]	-0.47	(-0.93--0.00)	**4.83E-02**	8.09E-02
232	LPC(18:2) [sn1]	-0.25	(-0.77- 0.27)	3.45E-01	4.09E-01
233	LPC(18:3) [sn2] (a)	-1.27	(-1.94--0.60)	**2.17E-04**	**6.76E-04**
234	LPC(18:3) [sn1] (a)/LPC(18:3) [sn2] (b)	-0.71	(-1.39--0.01)	**4.57E-02**	7.75E-02
235	LPC(18:3) (a) [sn1] [104_sn1]	-0.71	(-1.42- 0.01)	5.20E-02	8.64E-02
236	LPC(19:0) [sn1] (a) / LPC(19:0) [sn2] (b)	-1.53	(-1.97--1.08)	**3.81E-11**	**6.53E-10**
237	LPC(20:0) [sn1]	-1.62	(-2.04--1.19)	**3.01E-13**	**9.64E-12**
238	LPC(20:1) [sn2]	-0.59	(-1.03--0.16)	**7.98E-03**	**1.69E-02**
239	LPC(20:1) [sn1]	-0.61	(-1.05--0.17)	**6.96E-03**	**1.50E-02**
240	LPC(20:2) [sn2]	-0.81	(-1.29--0.33)	**1.00E-03**	**2.67E-03**
241	LPC(20:2) [sn1]	-0.52	(-0.97--0.06)	**2.62E-02**	**4.76E-02**
242	LPC(20:3) [sn2]	1.22	(0.60- 1.85)	**1.08E-04**	**3.67E-04**
243	LPC(20:3) [sn1]	1.45	(0.79- 2.11)	**1.76E-05**	**7.12E-05**
244	LPC(20:3) [104_sn1]	1.61	(0.94- 2.28)	**2.82E-06**	**1.47E-05**
245	LPC(20:4) [sn2]	0.85	(0.36- 1.35)	**7.28E-04**	**2.04E-03**
246	LPC(20:4) [sn1]	1.32	(0.78- 1.87)	**1.66E-06**	**8.77E-06**
247	LPC(20:5) [sn2]	0.19	(-0.84- 1.24)	7.14E-01	7.73E-01
248	LPC(20:5) [sn1]	0.44	(-0.62- 1.51)	4.18E-01	4.82E-01
249	LPC(22:4) [sn2]	-0.37	(-0.97- 0.23)	2.25E-01	2.86E-01
250	LPC(22:4) [sn1]	0.13	(-0.45- 0.72)	6.61E-01	7.21E-01
251	LPC(22:5) [sn2] (n3)	-0.82	(-1.41--0.23)	**6.89E-03**	**1.50E-02**
252	LPC(22:5) [sn1] (n3)/LPC(22:5) [sn2] (n6)	-0.64	(-1.20--0.08)	**2.59E-02**	**4.72E-02**
253	LPC(22:5) [sn1] (n6)	-0.40	(-1.13- 0.34)	2.89E-01	3.49E-01
254	LPC(22:5) (n3) [sn1] [104_sn1]	-0.51	(-1.17- 0.14)	1.26E-01	1.78E-01
255	LPC(22:6) [sn2]	-0.57	(-1.16- 0.02)	5.76E-02	9.31E-02
256	LPC(22:6) [sn1]	-0.08	(-0.70- 0.54)	7.99E-01	8.45E-01
257	LPC(O-16:0)	-0.26	(-0.68- 0.16)	2.31E-01	2.92E-01

258	LPC(O-18:0)	-1.04	(-1.55--0.52)	**8.66E-05**	**3.08E-04**
259	LPC(O-18:1)	0.51	(0.08- 0.94)	**2.06E-02**	**3.82E-02**
260	LPC(O-20:0)	-1.71	(-2.19--1.22)	**1.22E-11**	**2.55E-10**
261	LPC(O-20:1)	0.46	(-0.35- 1.27)	2.64E-01	3.28E-01
262	LPC(O-22:0)	-2.36	(-2.82--1.89)	**9.14E-22**	**1.10E-19**
263	LPC(O-22:1)	-1.65	(-2.14--1.15)	**1.69E-10**	**2.39E-09**
264	LPC(O-24:0)	-2.90	(-3.46--2.35)	**1.39E-22**	**2.23E-20**
265	LPC(O-24:1)	-2.37	(-2.90--1.84)	**1.45E-17**	**1.16E-15**
266	LPC(O-24:2)	-1.90	(-2.45--1.33)	**8.94E-11**	**1.39E-09**
267	LPC(P-16:0)	0.42	(-0.03- 0.88)	6.77E-02	1.07E-01
268	LPC(P-18:0)	0.35	(-0.09- 0.79)	1.15E-01	1.65E-01
269	LPC(P-18:1)	0.85	(0.30- 1.42)	**2.74E-03**	**6.59E-03**
270	LPC(P-20:0)	-0.75	(-1.29--0.20)	**7.43E-03**	**1.59E-02**
271	LPE(16:0) [sn2]	-0.54	(-1.02--0.06)	**2.86E-02**	5.13E-02
272	LPE(16:0) [sn1]	-0.39	(-0.85- 0.06)	9.23E-02	1.36E-01
273	LPE(18:0) [sn2]	0.26	(-0.19- 0.71)	2.50E-01	3.14E-01
274	LPE(18:0) [sn1]	0.44	(0.02- 0.87)	**4.07E-02**	7.00E-02
275	LPE(18:1) [sn2]	-0.73	(-1.25--0.21)	**6.51E-03**	**1.45E-02**
276	LPE(18:1) [sn1]	-0.69	(-1.20--0.18)	**8.03E-03**	**1.69E-02**
277	LPE(18:2) [sn2]	-0.77	(-1.40--0.14)	**1.63E-02**	**3.14E-02**
278	LPE(18:2) [sn1]	-0.81	(-1.45--0.16)	**1.44E-02**	**2.82E-02**
279	LPE(20:4) [sn1]	0.58	(0.07- 1.10)	**2.48E-02**	**4.54E-02**
280	LPE(22:6) [sn2]	-0.22	(-0.77- 0.34)	4.48E-01	5.14E-01
281	LPE(22:6) [sn1]	-0.23	(-0.83- 0.36)	4.42E-01	5.07E-01
282	PC(28:0)	-2.43	(-3.46--1.39)	**6.79E-06**	**3.08E-05**
283	PC(14:0_16:0)	-1.92	(-2.60--1.24)	**5.84E-08**	**3.84E-07**
284	PC(31:0) (a)	-2.98	(-3.98--1.97)	**1.59E-08**	**1.12E-07**
285	PC(31:0) (b)	-2.16	(-2.75--1.58)	**1.60E-12**	**4.27E-11**
286	PC(31:1)	-2.30	(-3.15--1.45)	**1.79E-07**	**1.06E-06**
287	PC(16:0_16:0)	-0.54	(-1.01--0.06)	**2.77E-02**	5.01E-02
288	PC(32:1)	-0.58	(-1.37- 0.21)	1.51E-01	2.04E-01
289	PC(32:2)	-1.33	(-1.97--0.69)	**5.63E-05**	**2.06E-04**
290	PC(33:0) (a)	-2.12	(-2.83--1.40)	**1.22E-08**	**9.14E-08**

291	PC(33:0) (b)	-1.66	(-2.19--1.12)	**2.20E-09**	**2.07E-08**
292	PC(33:1)	-2.23	(-2.90--1.55)	**2.77E-10**	**3.50E-09**
293	PC(33:2)	-2.10	(-2.70--1.51)	**1.37E-11**	**2.74E-10**
294	PC(16:0_18:0)	-0.91	(-1.30--0.51)	**7.79E-06**	**3.43E-05**
295	PC(16:0_18:1)	-0.96	(-1.48--0.43)	**3.81E-04**	**1.15E-03**
296	PC(16:0_18:2)	-0.27	(-0.56- 0.01)	6.26E-02	9.95E-02
297	PC(16:1_18:2)	-0.70	(-1.29--0.10)	**2.18E-02**	**4.03E-02**
298	PC(16:0_18:3) (a)	-1.50	(-2.13--0.87)	**3.82E-06**	**1.87E-05**
299	PC(14:0_20:4)	-0.59	(-1.32- 0.14)	1.15E-01	1.65E-01
300	PC(34:5)	-0.85	(-1.97- 0.29)	1.45E-01	1.99E-01
301	PC(15-MHDA_18:1)	-2.41	(-3.16--1.66)	**7.36E-10**	**8.21E-09**
302	PC(17:0_18:1)	-1.88	(-2.36--1.39)	**1.04E-13**	**3.57E-12**
303	PC(15-MHDA_18:2)	-1.74	(-2.51--0.97)	**1.13E-05**	**4.82E-05**
304	PC(17:0_18:2)	-1.47	(-1.98--0.96)	**2.43E-08**	**1.67E-07**
305	PC(17:1_18:2)	-1.07	(-1.58--0.55)	**5.63E-05**	**2.06E-04**
306	PC(15:0_20:3)	-0.55	(-0.79--0.30)	**1.27E-05**	**5.33E-05**
307	PC(15:0_20:4)	-1.25	(-1.91--0.59)	**2.44E-04**	**7.52E-04**
308	PC(35:5)	-1.32	(-2.49--0.13)	**3.04E-02**	5.41E-02
309	PC(36:0)	0.99	(0.41- 1.58)	**8.01E-04**	**2.20E-03**
310	PC(18:0_18:1)	-1.19	(-1.72--0.65)	**1.53E-05**	**6.23E-05**
311	PC(18:0_18:2)	-0.49	(-0.99- 0.02)	6.14E-02	9.79E-02
312	PC(16:0_20:3) (a)	-1.12	(-1.68--0.56)	**9.06E-05**	**3.20E-04**
313	PC(16:0_20:3) (b)	0.86	(0.14- 1.59)	**1.96E-02**	**3.67E-02**
314	PC(18:2_18:2)	-2.25	(-2.92--1.58)	**1.52E-10**	**2.28E-09**
315	PC(16:1_20:4)	-0.31	(-0.95- 0.33)	3.45E-01	4.09E-01
316	PC(16:0_20:5)	-0.01	(-1.15- 1.15)	9.87E-01	9.92E-01
317	PC(36:6)	-2.02	(-2.79--1.24)	**5.96E-07**	**3.29E-06**
318	PC(15-MHDA_20:4)	-0.65	(-1.39- 0.09)	8.52E-02	1.28E-01
319	PC(17:0_20:4)	-0.37	(-0.98- 0.24)	2.35E-01	2.97E-01
320	PC(15:0_22:6)	-2.61	(-3.31--1.91)	**9.22E-13**	**2.60E-11**
321	PC(38:2)	-0.99	(-1.42--0.56)	**8.38E-06**	**3.62E-05**
322	PC(18:0_20:3)	1.40	(0.74- 2.08)	**3.78E-05**	**1.43E-04**
323	PC(18:1_20:3)	-0.41	(-1.00- 0.18)	1.69E-01	2.26E-01

324	PC(38:4) (b)	-0.80	(-1.46--0.15)	**1.65E-02**	**3.15E-02**
325	PC(18:0_20:4)	0.92	(0.32- 1.52)	**2.72E-03**	**6.56E-03**
326	PC(38:5) (a)	-0.99	(-1.47--0.52)	**4.99E-05**	**1.86E-04**
327	PC(38:5) (b)	-1.08	(-1.77--0.38)	**2.72E-03**	**6.56E-03**
328	PC(38:6) (a)	-0.84	(-1.41--0.27)	**3.92E-03**	**9.14E-03**
329	PC(16:0_22:6)	-1.00	(-1.59--0.40)	**1.21E-03**	**3.15E-03**
330	PC(18:2_20:5)	-1.44	(-2.37--0.51)	**2.64E-03**	**6.45E-03**
331	PC(16:1_22:6)	-1.51	(-2.15--0.86)	**6.89E-06**	**3.09E-05**
332	PC(38:7)(c)	-1.55	(-2.11--0.99)	**8.20E-08**	**5.11E-07**
333	PC(39:5)(a)	-2.54	(-3.32--1.76)	**3.76E-10**	**4.63E-09**
334	PC(39:5)(b)	-2.23	(-2.92--1.53)	**8.17E-10**	**8.92E-09**
335	PC(15-MHDA_22:6)	-2.51	(-3.27--1.75)	**2.42E-10**	**3.14E-09**
336	PC(17:0_22:6)	-1.97	(-2.66--1.26)	**6.49E-08**	**4.10E-07**
337	PC(18:0_22:5) (n6)	-1.18	(-1.89--0.47)	**1.26E-03**	**3.25E-03**
338	PC(18:0_22:6)	-0.57	(-1.24- 0.11)	9.85E-02	1.45E-01
339	PC(40:7) (a)	-1.17	(-1.77--0.56)	**1.68E-04**	**5.51E-04**
340	PC(18:1_22:6) (a)	-1.58	(-2.09--1.06)	**3.73E-09**	**3.19E-08**
341	PC(40:8)	-1.59	(-2.09--1.08)	**1.66E-09**	**1.62E-08**
342	PC(44:12)	-2.16	(-3.16--1.15)	**3.40E-05**	**1.29E-04**
343	PC(O-16:0/16:0)	-0.70	(-1.11--0.29)	**7.75E-04**	**2.14E-03**
344	PC(O-32:1)	-0.77	(-1.29--0.24)	**4.50E-03**	**1.03E-02**
345	PC(O-34:1)	-0.75	(-1.15--0.34)	**3.53E-04**	**1.07E-03**
346	PC(O-34:2)	-1.75	(-2.33--1.17)	**6.44E-09**	**5.19E-08**
347	PC(O-34:4)	-0.55	(-1.47- 0.39)	2.49E-01	3.12E-01
348	PC(O-35:4)	-0.61	(-1.42- 0.20)	1.40E-01	1.94E-01
349	PC(O-36:0)	-1.21	(-1.68--0.74)	**6.03E-07**	**3.29E-06**
350	PC(O-18:0/18:1)	-1.41	(-1.86--0.96)	**1.69E-09**	**1.62E-08**
351	PC(O-18:1/18:1)	-1.13	(-1.56--0.70)	**3.79E-07**	**2.16E-06**
352	PC(O-18:0/18:2)	-2.08	(-2.63--1.54)	**3.31E-13**	**9.92E-12**
353	PC(O-18:1/18:2)	-1.25	(-1.77--0.73)	**3.42E-06**	**1.75E-05**
354	PC(O-16:0/20:3)	-0.28	(-0.79- 0.22)	2.69E-01	3.30E-01
355	PC(O-16:0/20:4)	-0.12	(-0.62- 0.39)	6.47E-01	7.09E-01
356	PC(O-36:5)	-0.85	(-1.79- 0.09)	7.70E-02	1.18E-01

357	PC(O-18:0/20:4)	-0.45	(-0.93- 0.04)	7.33E-02	1.14E-01
358	PC(O-38:5)	-0.05	(-0.49- 0.40)	8.37E-01	8.79E-01
359	PC(O-16:0/22:6)	-0.05	(-0.69- 0.61)	8.91E-01	9.18E-01
360	PC(O-40:5)	-0.82	(-1.29--0.35)	**6.74E-04**	**1.90E-03**
361	PC(O-18:0/22:6)	-0.57	(-1.10--0.02)	**4.07E-02**	7.00E-02
362	PC(O-40:7) (a)	0.03	(-0.58- 0.65)	9.22E-01	9.44E-01
363	PC(P-16:0/14:0)	-1.12	(-1.68--0.56)	**1.06E-04**	**3.64E-04**
364	PC(P-16:0/16:0)	-0.62	(-1.10--0.14)	**1.16E-02**	**2.32E-02**
365	PC(P-16:0/16:1)	-0.51	(-1.07- 0.06)	8.08E-02	1.22E-01
366	PC(P-16:0/18:0)	-1.09	(-1.46--0.72)	**1.37E-08**	**1.01E-07**
367	PC(P-16:0/18:1)	-1.11	(-1.62--0.60)	**2.15E-05**	**8.53E-05**
368	PC(P-16:0/18:2)	-0.93	(-1.49--0.37)	**1.10E-03**	**2.91E-03**
369	PC(P-16:0/18:3)	-1.43	(-2.04--0.83)	**4.49E-06**	**2.11E-05**
370	PC(P-35:2) (a)	-1.94	(-2.77--1.11)	**6.24E-06**	**2.85E-05**
371	PC(P-35:2) (b)	-1.55	(-2.24--0.86)	**1.30E-05**	**5.41E-05**
372	PC(P-15:0/20:4) (b)	-0.41	(-1.06- 0.23)	2.09E-01	2.74E-01
373	PC(P-18:1/18:1)	-1.10	(-1.59--0.61)	**1.35E-05**	**5.58E-05**
374	PC(P-18:0/18:2)	-1.74	(-2.29--1.19)	**9.23E-10**	**9.84E-09**
375	PC(P-16:0/20:4)	0.45	(-0.13- 1.03)	1.25E-01	1.77E-01
376	PC(P-16:0/20:5)	-0.26	(-1.26- 0.74)	6.04E-01	6.70E-01
377	PC(P-17:0/20:4) (a)	-0.92	(-1.67--0.17)	**1.68E-02**	**3.19E-02**
378	PC(P-17:0/20:4) (b)	-0.21	(-0.91- 0.49)	5.49E-01	6.18E-01
379	PC(P-18:0/20:4)	-0.04	(-0.59- 0.51)	8.87E-01	9.16E-01
380	PC(P-38:5) (a)	0.26	(-0.31- 0.83)	3.69E-01	4.31E-01
381	PC(P-38:5) (b)	-0.72	(-1.27--0.17)	**1.08E-02**	**2.21E-02**
382	PC(P-16:0/22:6)	-0.11	(-0.79- 0.58)	7.57E-01	8.11E-01
383	PC(P-20:0/20:4)	-0.42	(-0.99- 0.15)	1.48E-01	2.01E-01
384	PC(P-40:5) (b)	-0.69	(-1.23--0.16)	**1.13E-02**	**2.28E-02**
385	PC(P-18:0/22:6)	-0.48	(-1.13- 0.18)	1.53E-01	2.06E-01
386	PE(16:0_16:1)	-0.27	(-1.36- 0.84)	6.33E-01	6.97E-01
387	PE(16:0_18:1)	-0.02	(-0.76- 0.71)	9.47E-01	9.65E-01
388	PE(16:0_18:2)	-0.07	(-0.90- 0.76)	8.63E-01	9.01E-01
389	PE(16:1_18:2)	-0.65	(-1.62- 0.33)	1.91E-01	2.52E-01

390	PE(16:0_18:3) (a)	-1.35	(-2.23--0.46)	**2.93E-03**	**6.99E-03**
391	PE(16:0_18:3) (b)	-0.24	(-1.10- 0.63)	5.84E-01	6.54E-01
392	PE(17:0_18:2)	-1.10	(-1.76--0.43)	**1.28E-03**	**3.29E-03**
393	PE(18:0_18:1)	0.47	(-0.14- 1.08)	1.34E-01	1.86E-01
394	PE(18:1_18:1)	-0.30	(-0.93- 0.33)	3.50E-01	4.14E-01
395	PE(18:0_18:2)	0.67	(-0.01- 1.35)	5.23E-02	8.65E-02
396	PE(18:1_18:2)	-0.61	(-1.39- 0.17)	1.27E-01	1.79E-01
397	PE(16:0_20:3)	-0.14	(-0.95- 0.66)	7.25E-01	7.82E-01
398	PE(16:0_20:4)	0.82	(0.11- 1.52)	**2.29E-02**	**4.20E-02**
399	PE(16:1_20:4)	-0.02	(-0.87- 0.83)	9.56E-01	9.71E-01
400	PE(16:0_20:5)	-0.12	(-1.12- 0.89)	8.11E-01	8.54E-01
401	PE(17:0_20:4)	0.00	(-0.64- 0.63)	9.92E-01	9.94E-01
402	PE(18:0_20:3) (a)	1.82	(1.06- 2.59)	**3.08E-06**	**1.59E-05**
403	PE(18:0_20:4)	1.97	(1.39- 2.56)	**5.87E-11**	**9.72E-10**
404	PE(38:5) (a)	0.10	(-0.60- 0.79)	7.86E-01	8.33E-01
405	PE(38:5) (b)	-0.75	(-1.56- 0.07)	7.23E-02	1.12E-01
406	PE(16:0_22:6)	0.07	(-0.63- 0.77)	8.52E-01	8.91E-01
407	PE(17:0_22:6)	-0.16	(-0.86- 0.56)	6.69E-01	7.28E-01
408	PE(18:0_22:4)	0.64	(-0.18- 1.47)	1.26E-01	1.78E-01
409	PE(18:0_22:5) (n3)	0.66	(-0.01- 1.33)	5.49E-02	9.06E-02
410	PE(18:0_22:5) (n6)	0.40	(-0.47- 1.29)	3.67E-01	4.30E-01
411	PE(18:0_22:6)	1.33	(0.59- 2.08)	**4.39E-04**	**1.28E-03**
412	PE(40:7)	-0.30	(-1.10- 0.51)	4.65E-01	5.29E-01
413	PE(O-34:1)	-1.21	(-1.76--0.65)	**2.30E-05**	**9.04E-05**
414	PE(O-16:0/18:2)	-2.30	(-3.04--1.55)	**2.74E-09**	**2.44E-08**
415	PE(O-18:1/18:2)	-1.33	(-2.03--0.63)	**2.38E-04**	**7.36E-04**
416	PE(O-16:0/20:4)	-1.07	(-1.77--0.37)	**2.99E-03**	**7.11E-03**
417	PE(O-36:5)	-1.89	(-2.94--0.84)	**4.62E-04**	**1.33E-03**
418	PE(O-16:0/22:4)	-0.54	(-1.08- 0.02)	5.74E-02	9.31E-02
419	PE(O-18:0/20:4)	-1.43	(-2.21--0.64)	**4.06E-04**	**1.20E-03**
420	PE(O-38:5) (a)	-1.14	(-1.83--0.45)	**1.32E-03**	**3.38E-03**
421	PE(O-38:5) (b)	-1.72	(-2.35--1.08)	**1.73E-07**	**1.04E-06**
422	PE(O-16:0/22:6)	-0.84	(-1.44--0.23)	**7.04E-03**	**1.52E-02**

423	PE(O-18:0/22:6)	-1.30	(-1.88--0.72)	**1.52E-05**	**6.23E-05**
424	PE(O-18:1/22:6)	-0.97	(-1.54--0.39)	**9.92E-04**	**2.66E-03**
425	PE(P-16:0/18:1)	-0.86	(-1.36--0.36)	**7.40E-04**	**2.06E-03**
426	PE(P-16:0/18:2)	-1.15	(-1.80--0.50)	**5.72E-04**	**1.65E-03**
427	PE(P-16:0/20:3)	0.04	(-0.65- 0.75)	9.04E-01	9.29E-01
428	PE(P-16:0/20:4)	0.40	(-0.29- 1.10)	2.58E-01	3.21E-01
429	PE(P-16:0/20:5)	-0.61	(-1.87- 0.68)	3.53E-01	4.14E-01
430	PE(P-16:0/22:4)	0.33	(-0.19- 0.86)	2.16E-01	2.81E-01
431	PE(P-16:0/22:5) (n3)	-0.43	(-0.99- 0.14)	1.38E-01	1.91E-01
432	PE(P-16:0/22:5) (n6)	-0.67	(-1.28--0.06)	**3.09E-02**	5.48E-02
433	PE(P-16:0/22:6)	0.18	(-0.37- 0.73)	5.27E-01	5.97E-01
434	PE(P-17:0/20:4) (a)	-0.07	(-0.89- 0.76)	8.69E-01	9.05E-01
435	PE(P-17:0/20:4) (b)	0.12	(-0.65- 0.90)	7.61E-01	8.14E-01
436	PE(P-17:0/22:6) (a)	-1.02	(-1.77--0.27)	**8.14E-03**	**1.70E-02**
437	PE(P-17:0/22:6) (b)	-0.51	(-1.20- 0.18)	1.46E-01	1.99E-01
438	PE(P-18:0/18:1)	-0.97	(-1.54--0.40)	**8.42E-04**	**2.30E-03**
439	PE(P-18:0/18:2)	-1.48	(-2.10--0.86)	**4.03E-06**	**1.95E-05**
440	PE(P-18:0/20:3)	-0.53	(-1.14- 0.09)	9.38E-02	1.38E-01
441	PE(P-18:0/20:4)	0.05	(-0.67- 0.78)	8.83E-01	9.15E-01
442	PE(P-18:0/20:5)	-1.20	(-2.50- 0.12)	7.52E-02	1.16E-01
443	PE(P-18:0/22:4)	-0.73	(-1.29--0.18)	**1.01E-02**	**2.07E-02**
444	PE(P-18:0/22:5) (n3)	-0.63	(-1.20--0.07)	**2.88E-02**	5.13E-02
445	PE(P-18:0/22:5) (n6)	-1.11	(-1.66--0.56)	**9.76E-05**	**3.42E-04**
446	PE(P-18:0/22:6)	-0.60	(-1.22- 0.02)	5.80E-02	9.34E-02
447	PE(P-18:1/18:1)	-0.84	(-1.36--0.32)	**1.60E-03**	**4.03E-03**
448	PE(P-18:1/18:2)	-1.57	(-2.23--0.89)	**6.16E-06**	**2.84E-05**
449	PE(P-18:1/20:4)	0.10	(-0.61- 0.82)	7.85E-01	8.33E-01
450	PE(P-18:1/20:5)	-1.17	(-2.42- 0.09)	6.87E-02	1.08E-01
451	PE(P-18:1/22:4)	-0.18	(-0.79- 0.43)	5.58E-01	6.26E-01
452	PE(P-18:1/22:5) (a)	-0.62	(-1.20--0.03)	**3.86E-02**	6.71E-02
453	PE(P-18:1/22:6) (a)	-0.38	(-1.00- 0.24)	2.26E-01	2.87E-01
454	PE(P-20:0/18:1)	-1.16	(-1.74--0.56)	**1.46E-04**	**4.89E-04**
455	PE(P-20:0/18:2)	-1.56	(-2.09--1.03)	**1.52E-08**	**1.10E-07**

456	PE(P-20:0/20:4)	0.01	(-0.56- 0.58)	9.71E-01	9.82E-01
457	PE(P-20:0/22:6)	-0.63	(-1.12--0.13)	**1.35E-02**	**2.67E-02**
458	PE(P-20:1/20:4)	-0.26	(-1.08- 0.58)	5.43E-01	6.11E-01
459	PG(34:2)	-0.90	(-1.61--0.18)	**1.45E-02**	**2.82E-02**
460	PG(36:1)	-0.29	(-0.91- 0.33)	3.53E-01	4.14E-01
461	PI(16:0/16:0)	-2.14	(-3.02--1.24)	**3.56E-06**	**1.80E-05**
462	PI(16:0_16:1)	-1.42	(-2.44--0.40)	**6.52E-03**	**1.45E-02**
463	PI(34:0)	-2.48	(-3.36--1.60)	**6.20E-08**	**3.97E-07**
464	PI(34:1)	-1.84	(-2.48--1.20)	**2.61E-08**	**1.76E-07**
465	PI(17:0_18:1)	-2.27	(-2.82--1.72)	**3.26E-15**	**1.57E-13**
466	PI(17:0_18:2)	-2.60	(-3.19--2.00)	**1.23E-16**	**7.39E-15**
467	PI(18:0_18:1)	-1.80	(-2.47--1.13)	**1.90E-07**	**1.11E-06**
468	PI(36:2)	-1.90	(-2.45--1.35)	**3.65E-11**	**6.49E-10**
469	PI(18:1_18:2)	-2.55	(-3.14--1.95)	**3.92E-16**	**2.09E-14**
470	PI(16:0_20:3) (a)	-1.92	(-2.55--1.28)	**6.49E-09**	**5.19E-08**
471	PI(16:0_20:3) (b)	-2.27	(-2.99--1.55)	**1.20E-09**	**1.23E-08**
472	PI(16:0_20:4)	-1.33	(-1.88--0.77)	**4.23E-06**	**2.01E-05**
473	PI(18:0_20:2)	-2.42	(-3.08--1.75)	**5.08E-12**	**1.16E-10**
474	PI(18:0_20:3) (a)	-0.92	(-1.39--0.44)	**1.79E-04**	**5.82E-04**
475	PI(18:0_20:4)	-0.83	(-1.31--0.34)	**9.23E-04**	**2.50E-03**
476	PI(38:5) (a)	-2.30	(-2.82--1.78)	**5.13E-17**	**3.52E-15**
477	PI(38:6)	-2.45	(-3.06--1.83)	**3.89E-14**	**1.44E-12**
478	PI(18:0_22:4)	-1.12	(-1.73--0.50)	**4.46E-04**	**1.30E-03**
479	PI(18:0_22:5) (n3)	-1.50	(-2.14--0.87)	**4.94E-06**	**2.30E-05**
480	PI(18:0_22:6)	-1.86	(-2.48--1.24)	**6.95E-09**	**5.47E-08**

Table S3A. The overlapping lipid species between the ppBMI and postnatal BMI studies. Related to Figure 3.

	ppBMI (antenatal)				Adult BMI (postnatal)				
Lipid Species	%change in lipid concentration per unit BMI	95%CI	p-value	p-value(BH)	%change in lipid concentration per unit BMI	95%CI	p-value	p-value(BH)	Trend
CE(16:1)	-1.06	(-1.82--0.29)	7.23E-03	1.46E-02	2.17	(1.53-2.82)	6.89E-11	4.60E-10	opposite
CE(18:3)	-1.93	(-2.54--1.32)	1.28E-09	1.25E-08	0.72	(0.11-1.32)	2.04E-02	3.21E-02	opposite
CE(20:1)	-0.83	(-1.29--0.37)	4.07E-04	1.15E-03	-0.70	(-1.17--0.22)	4.06E-03	7.58E-03	same
CE(22:5) (n6)	-1.88	(-2.46--1.29)	7.49E-10	7.92E-09	-0.68	(-1.1--0.26)	1.77E-03	3.56E-03	same
DG(16:0_18:1)	1.33	(0.59-2.07)	4.36E-04	1.22E-03	4.26	(3.33-5.21)	1.32E-18	4.86E-17	same
DG(16:0_18:2)	0.92	(0.21-1.65)	1.15E-02	2.16E-02	3.62	(2.72-4.52)	5.53E-15	7.59E-14	same
DG(18:0_18:1)	1.36	(0.69-2.04)	7.91E-05	2.60E-04	4.41	(3.51-5.31)	4.70E-21	4.51E-19	same
DG(18:1_18:1)	1.37	(0.64-2.1)	2.23E-04	6.69E-04	2.93	(2.08-3.77)	1.44E-11	1.03E-10	same
DG(18:0_18:2)	0.99	(0.23-1.76)	1.04E-02	1.97E-02	4.40	(3.41-5.39)	7.51E-18	2.57E-16	same
DG(18:1_18:2)	0.95	(0.22-1.68)	1.02E-02	1.97E-02	2.26	(1.45-3.08)	5.91E-08	2.42E-07	same
DG(16:0_20:4)	1.19	(0.26-2.12)	1.22E-02	2.27E-02	4.27	(3.25-5.3)	4.79E-16	9.59E-15	same
DG(18:1_20:4)	1.14	(0.4-1.88)	2.42E-03	5.53E-03	2.47	(1.71-3.22)	1.81E-10	1.10E-09	same
DG(18:1_22:6)	1.53	(0.47-2.61)	4.63E-03	9.97E-03	3.18	(2.04-4.34)	5.26E-08	2.22E-07	same
DG(18:2_22:6)	1.52	(0.42-2.63)	6.55E-03	1.35E-02	3.05	(1.87-4.24)	3.60E-07	1.28E-06	same
TG(48:0) [NL-18:0]	-1.56	(-2.43--0.68)	5.76E-04	1.53E-03	4.34	(2.99-5.71)	3.54E-10	2.07E-09	opposite
TG(48:2) [NL-14:1]	-1.20	(-2.25--0.13)	2.78E-02	4.70E-02	4.79	(3.42-6.18)	7.51E-12	5.72E-11	opposite
TG(48:2) [NL-18:2]	-1.16	(-2.2--0.12)	2.91E-02	4.87E-02	5.21	(3.68-6.77)	2.87E-11	2.03E-10	opposite
TG(48:3) [NL-14:0]	-1.51	(-2.64--0.37)	9.55E-03	1.85E-02	4.32	(2.9-5.76)	2.31E-09	1.14E-08	opposite
TG(48:3) [NL-18:3]	-1.81	(-3.07--0.53)	5.76E-03	1.21E-02	4.99	(3.41-6.59)	5.23E-10	2.92E-09	opposite
TG(50:1) [NL-16:0]	0.83	(0.22-1.43)	7.27E-03	1.46E-02	4.34	(3.39-5.3)	1.03E-18	4.12E-17	same
TG(50:1) [NL-18:1]	0.76	(0.18-1.34)	1.03E-02	1.97E-02	4.10	(3.16-5.05)	4.06E-17	1.08E-15	same
TG(50:2) [NL-14:0]	-0.65	(-1.21--0.07)	2.69E-02	4.57E-02	2.80	(1.76-3.86)	1.50E-07	5.54E-07	opposite
TG(50:2) [NL-18:2]	0.66	(0.08-1.24)	2.58E-02	4.41E-02	3.72	(2.82-4.62)	8.45E-16	1.53E-14	same
TG(52:1) [NL-18:0]	1.38	(0.59-2.18)	6.58E-04	1.73E-03	5.69	(4.53-6.86)	2.15E-21	2.58E-19	same
TG(52:1) [NL-18:1]	1.45	(0.69-2.23)	2.04E-04	6.17E-04	5.86	(4.73-7)	1.79E-23	8.60E-21	same
TG(52:2) [NL-16:0]	0.80	(0.4-1.21)	1.01E-04	3.29E-04	2.18	(1.57-2.79)	3.69E-12	3.11E-11	same
TG(52:3) [NL-18:2]	0.76	(0.32-1.2)	8.08E-04	2.08E-03	1.84	(1.22-2.48)	1.11E-08	5.14E-08	same
TG(52:4) [NL-18:2]	1.20	(0.44-1.96)	1.92E-03	4.52E-03	2.29	(1.29-3.3)	7.06E-06	2.12E-05	same
TG(53:2) [NL-18:1]	0.88	(0.2-1.56)	1.08E-02	2.03E-02	2.66	(1.85-3.47)	1.63E-10	1.00E-09	same
TG(54:1) [NL-18:1]	0.99	(0.32-1.66)	3.57E-03	8.00E-03	4.45	(3.57-5.34)	4.54E-22	7.26E-20	same
TG(54:2) [NL-18:0]	1.41	(0.72-2.1)	6.55E-05	2.23E-04	4.24	(3.35-5.13)	2.72E-20	1.63E-18	same
TG(54:3) [NL-18:2]	0.72	(0.14-1.3)	1.43E-02	2.59E-02	2.23	(1.57-2.89)	5.13E-11	3.51E-10	same
TG(54:5) [NL-20:4]	1.04	(0.38-1.71)	1.91E-03	4.51E-03	3.37	(2.55-4.2)	2.12E-15	3.39E-14	same
TG(54:6) [NL-20:4]	1.16	(0.33-2)	6.24E-03	1.29E-02	3.69	(2.71-4.68)	2.17E-13	2.27E-12	same
TG(54:6) [NL-22:6]	1.79	(0.57-3.02)	4.10E-03	9.06E-03	5.76	(4.33-7.22)	4.96E-15	7.00E-14	same
TG(56:6) [NL-20:4]	1.34	(0.81-1.87)	7.12E-07	3.60E-06	1.36	(0.85-1.88)	2.38E-07	8.65E-07	same
TG(56:7) [NL-20:4]	1.02	(0.35-1.7)	2.84E-03	6.40E-03	1.88	(1.14-2.62)	6.63E-07	2.29E-06	same
TG(56:7) [NL-22:6]	1.93	(0.84-3.04)	4.97E-04	1.35E-03	3.96	(2.65-5.28)	3.33E-09	1.61E-08	same
TG(56:8) [NL-22:6]	1.76	(0.54-2.99)	4.49E-03	9.76E-03	3.97	(2.64-5.31)	4.96E-09	2.38E-08	same
TG(58:8) [NL-22:6]	1.46	(0.38-2.56)	7.98E-03	1.58E-02	2.80	(1.58-4.04)	7.21E-06	2.15E-05	same
TG(O-54:2) [NL-18:1]	0.85	(0.18-1.53)	1.33E-02	2.43E-02	2.63	(1.82-3.44)	2.48E-10	1.47E-09	same
dhCer(d18:0/22:0)	1.14	(0.5-1.79)	4.77E-04	1.32E-03	1.96	(1.35-2.58)	4.98E-10	2.81E-09	same
dhCer(d18:0/24:1)	1.70	(0.99-2.41)	3.01E-06	1.36E-05	1.49	(0.78-2.2)	4.22E-05	1.11E-04	same
Cer(d16:1/22:0)	-0.73	(-1.33--0.13)	1.79E-02	3.18E-02	1.20	(0.56-1.84)	2.31E-04	5.51E-04	opposite
Cer(d16:1/23:0)	-0.99	(-1.66--0.32)	3.93E-03	8.74E-03	0.76	(0.1-1.42)	2.38E-02	3.69E-02	opposite
Cer(d18:1/18:0)	1.97	(1.31-2.64)	7.34E-09	5.29E-08	2.07	(1.45-2.7)	1.07E-10	6.87E-10	same
Cer(d18:2/24:0)	-1.25	(-1.79--0.69)	1.15E-05	4.61E-05	-0.68	(-1.22--0.14)	1.42E-02	2.32E-02	same
Cer(d19:1/24:0)	-1.22	(-2.12--0.32)	8.01E-03	1.58E-02	1.08	(0.23-1.93)	1.29E-02	2.13E-02	opposite
Cer(m18:0/24:1)	1.15	(0.26-2.04)	1.11E-02	2.08E-02	2.84	(2-3.68)	3.94E-11	2.74E-10	same
Cer(m18:1/24:1)	1.37	(0.47-2.27)	2.70E-03	6.11E-03	2.73	(1.88-3.59)	4.23E-10	2.42E-09	same
GM3(d18:1/18:0)	-1.16	(-1.7--0.62)	2.65E-05	9.76E-05	-0.70	(-1.2--0.2)	5.94E-03	1.06E-02	same
GM3(d18:1/24:0)	-3.17	(-3.78--2.56)	5.91E-22	9.47E-20	-1.92	(-2.46--1.39)	6.41E-12	5.13E-11	same
GM3(d18:1/24:1)	-1.77	(-2.36--1.17)	1.05E-08	7.23E-08	-1.28	(-1.87--0.68)	2.89E-05	7.79E-05	same
HexCer(d18:1/18:0)	-1.56	(-2.15--0.97)	3.24E-07	1.80E-06	-1.40	(-2.04--0.75)	2.70E-05	7.38E-05	same
HexCer(d18:1/20:0)	-1.84	(-2.41--1.27)	4.40E-10	5.02E-09	-1.29	(-1.81--0.76)	2.18E-06	6.88E-06	same
HexCer(d18:1/22:0)	-1.93	(-2.43--1.44)	1.05E-13	3.87E-12	-0.92	(-1.41--0.43)	2.61E-04	6.17E-04	same
HexCer(d18:1/24:0)	-2.60	(-3.1--2.09)	5.92E-22	9.47E-20	-1.34	(-1.83--0.85)	1.19E-07	4.57E-07	same
HexCer(d18:1/24:1)	-1.68	(-2.24--1.13)	5.30E-09	4.04E-08	-1.23	(-1.76--0.69)	8.47E-06	2.48E-05	same
Hex2Cer(d18:1/16:0)	-1.58	(-2.1--1.05)	6.78E-09	5.09E-08	-1.17	(-1.7--0.63)	2.30E-05	6.34E-05	same
Hex2Cer(d18:1/22:0)	-1.73	(-2.29--1.17)	2.30E-09	2.05E-08	-1.43	(-2--0.87)	8.84E-07	2.91E-06	same
Hex2Cer(d18:1/24:1)	-1.66	(-2.25--1.07)	4.96E-08	3.13E-07	-1.91	(-2.5--1.32)	5.82E-10	3.21E-09	same
Hex3Cer(d18:1/16:0)	-1.04	(-1.45--0.62)	1.58E-06	7.44E-06	-1.03	(-1.43--0.63)	6.36E-07	2.21E-06	same
Hex3Cer(d18:1/22:0)	-1.54	(-2.14--0.93)	9.28E-07	4.59E-06	-2.07	(-2.63--1.51)	2.59E-12	2.22E-11	same
Hex3Cer(d18:1/24:0)	-1.57	(-2.15--0.99)	1.64E-07	9.85E-07	-1.90	(-2.42--1.37)	6.58E-12	5.18E-11	same

Hex3Cer(d18:1/24:1)	-1.14	(-1.69--0.58)	**6.61E-05**	**2.23E-04**	-1.42	(-1.92--0.93)	**3.28E-08**	**1.42E-07**	same
SM(d18:0/14:0)	-0.89	(-1.42--0.36)	**1.08E-03**	**2.70E-03**	0.73	(0.18-1.28)	**9.62E-03**	**1.64E-02**	opposite
SM(d18:1/14:0)/SM(d16:1/16:0)	-1.03	(-1.5--0.55)	**2.66E-05**	**9.76E-05**	0.72	(0.27-1.18)	**1.93E-03**	**3.86E-03**	opposite
SM(d18:1/16:0)	-1.46	(-1.99--0.92)	**1.89E-07**	**1.08E-06**	-0.89	(-1.41--0.37)	**9.07E-04**	**1.95E-03**	same
SM(35:2) (b)	-1.53	(-2.12--0.94)	**4.86E-07**	**2.56E-06**	-0.79	(-1.37--0.2)	**8.48E-03**	**1.46E-02**	same
SM(37:2)	-1.21	(-1.81--0.6)	**1.07E-04**	**3.46E-04**	-1.13	(-1.68--0.57)	**7.90E-05**	**2.02E-04**	same
SM(d18:1/20:0)/SM(d16:1/22:0)	-0.75	(-1.17--0.33)	**4.82E-04**	**1.32E-03**	0.43	(0.04-0.83)	**2.93E-02**	**4.47E-02**	opposite
SM(38:3) (b)	-0.77	(-1.33--0.22)	**6.71E-03**	**1.37E-02**	-0.67	(-1.28--0.07)	**3.00E-02**	**4.55E-02**	same
SM(d18:1/22:0)/SM(d16:1/24:0)	-0.59	(-1.06--0.13)	**1.28E-02**	**2.38E-02**	0.71	(0.26-1.16)	**2.08E-03**	**4.13E-03**	opposite
SM(d16:1/24:1)	-0.70	(-1.21--0.19)	**7.53E-03**	**1.51E-02**	0.55	(0.05-1.05)	**3.15E-02**	**4.75E-02**	opposite
SM(d18:2/23:0)	-0.93	(-1.36--0.5)	**2.33E-05**	**8.74E-05**	-0.51	(-0.9--0.11)	**1.15E-02**	**1.91E-02**	same
SM(d18:2/24:0)	-1.48	(-1.89--1.07)	**6.63E-12**	**1.45E-10**	-0.85	(-1.23--0.46)	**1.89E-05**	**5.27E-05**	same
SM(44:3) (a)	-1.42	(-1.88--0.95)	**4.02E-09**	**3.21E-08**	-1.26	(-1.69--0.84)	**1.15E-08**	**5.25E-08**	same
LPC(15-MHDA) [sn2]	-1.34	(-2.09--0.59)	**5.17E-04**	**1.39E-03**	-1.80	(-2.53--1.05)	**2.97E-06**	**9.21E-06**	same
LPC(15-MHDA) [sn1] / LPC(17:0) [sn2]	-1.22	(-1.86--0.59)	**1.83E-04**	**5.61E-04**	-1.69	(-2.31--1.07)	**1.33E-07**	**5.00E-07**	same
LPC(17:0) [sn1]	-0.89	(-1.39--0.39)	**5.04E-04**	**1.36E-03**	-1.40	(-1.9--0.89)	**8.98E-08**	**3.53E-07**	same
LPC(17:1) [sn1] (a) / LPC(17:1) [sn2] (b)	-0.95	(-1.52--0.37)	**1.28E-03**	**3.14E-03**	-0.68	(-1.27--0.08)	**2.64E-02**	**4.06E-02**	same
LPC(18:1) [sn2]	-0.59	(-1.11--0.07)	**2.69E-02**	**4.57E-02**	-1.43	(-2--0.86)	**1.28E-06**	**4.15E-06**	same
LPC(18:2) [sn2]	-0.64	(-1.13--0.15)	**1.04E-02**	**1.97E-02**	-2.17	(-2.7--1.63)	**2.57E-14**	**3.16E-13**	same
LPC(18:3) [sn2] (a)	-1.54	(-2.23--0.85)	**1.68E-05**	**6.51E-05**	-0.86	(-1.59--0.12)	**2.29E-02**	**3.56E-02**	same
LPC(19:0) [sn1] (a) / LPC(19:0) [sn2] (b)	-1.56	(-2.02--1.09)	**1.61E-10**	**2.49E-09**	-2.28	(-2.79--1.76)	**6.69E-17**	**1.61E-15**	same
LPC(20:0) [sn1]	-1.59	(-2.03--1.14)	**1.26E-11**	**2.64E-10**	-2.10	(-2.58--1.62)	**1.05E-16**	**2.41E-15**	same
LPC(20:1) [sn2]	-0.60	(-1.07--0.14)	**1.07E-02**	**2.02E-02**	-1.58	(-2.1--1.06)	**5.88E-09**	**2.79E-08**	same
LPC(20:1) [sn1]	-0.62	(-1.08--0.15)	**9.49E-03**	**1.84E-02**	-1.86	(-2.38--1.33)	**1.33E-11**	**9.82E-11**	same
LPC(20:2) [sn2]	-0.91	(-1.41--0.4)	**4.81E-04**	**1.32E-03**	-0.80	(-1.3--0.31)	**1.50E-03**	**3.03E-03**	same
LPC(20:2) [sn1]	-0.63	(-1.11--0.16)	**9.14E-03**	**1.78E-02**	-0.94	(-1.4--0.46)	**1.11E-04**	**2.77E-04**	same
LPC(20:3) [sn2]	1.05	(0.4-1.7)	**1.48E-03**	**3.59E-03**	1.19	(0.56-1.83)	**2.39E-04**	**5.67E-04**	same
LPC(20:3) [sn1]	1.28	(0.59-1.97)	**2.78E-04**	**8.08E-04**	0.91	(0.26-1.56)	**5.99E-03**	**1.06E-02**	same
LPC(20:3) [104_sn1]	1.42	(0.72-2.12)	**7.46E-05**	**2.47E-04**	0.93	(0.28-1.58)	**4.92E-03**	**8.95E-03**	same
LPC(20:4) [sn2]	0.85	(0.33-1.37)	**1.45E-03**	**3.53E-03**	-0.79	(-1.3--0.28)	**2.64E-03**	**5.13E-03**	opposite
LPC(20:4) [sn1]	1.36	(0.79-1.93)	**3.16E-06**	**1.42E-05**	-1.16	(-1.71--0.61)	**4.15E-05**	**1.10E-04**	opposite
LPC(22:5) [sn2] (n3)	-0.98	(-1.6--0.35)	**2.21E-03**	**5.12E-03**	-0.74	(-1.34--0.14)	**1.67E-02**	**2.68E-02**	same
LPC(22:5) [sn1] (n3)/LPC(22:5) [sn2] (n6)	-0.84	(-1.42--0.26)	**4.63E-03**	**9.97E-03**	-0.82	(-1.43--0.21)	**8.19E-03**	**1.42E-02**	same
LPC(O-18:0)	-1.05	(-1.59--0.5)	**1.83E-04**	**5.61E-04**	-1.53	(-2.07--0.99)	**5.02E-08**	**2.13E-07**	same
LPC(O-18:1)	0.52	(0.07-0.97)	**2.38E-02**	**4.13E-02**	-0.80	(-1.25--0.35)	**5.53E-04**	**1.23E-03**	opposite
LPC(O-20:0)	-1.70	(-2.21--1.19)	**1.86E-10**	**2.70E-09**	-2.23	(-2.77--1.69)	**4.19E-15**	**6.29E-14**	same
LPC(O-22:0)	-2.40	(-2.88--1.91)	**1.69E-20**	**2.02E-18**	-1.43	(-1.85--1)	**1.03E-10**	**6.79E-10**	same
LPC(O-22:1)	-1.62	(-2.15--1.1)	**2.73E-09**	**2.33E-08**	-2.10	(-2.63--1.56)	**9.90E-14**	**1.16E-12**	same
LPC(O-24:0)	-3.04	(-3.62--2.45)	**1.56E-22**	**7.48E-20**	-1.17	(-1.62--0.71)	**7.24E-07**	**2.45E-06**	same
LPC(O-24:1)	-2.43	(-2.98--1.87)	**1.19E-16**	**8.16E-15**	-1.37	(-1.84--0.9)	**1.75E-08**	**7.86E-08**	same
LPC(O-24:2)	-1.94	(-2.53--1.34)	**3.86E-10**	**4.64E-09**	-2.07	(-2.6--1.53)	**1.68E-13**	**1.79E-12**	same
LPC(P-18:1)	0.95	(0.36-1.54)	**1.57E-03**	**3.78E-03**	-0.88	(-1.4--0.35)	**1.14E-03**	**2.40E-03**	opposite
LPC(P-20:0)	-0.73	(-1.3--0.15)	**1.34E-02**	**2.43E-02**	-1.56	(-2.13--0.99)	**1.23E-07**	**4.68E-07**	same
LPE(16:0) [sn2]	-0.58	(-1.09--0.08)	**2.45E-02**	**4.23E-02**	-1.40	(-1.94--0.85)	**8.49E-07**	**2.81E-06**	same
PC(31:1)	-2.56	(-3.45--1.67)	**3.05E-08**	**2.06E-07**	1.45	(0.56-2.34)	**1.35E-03**	**2.79E-03**	opposite
PC(33:2)	-2.27	(-2.89--1.64)	**6.00E-12**	**1.37E-10**	-0.81	(-1.42--0.19)	**1.07E-02**	**1.78E-02**	same
PC(16:0_18:2)	-0.34	(-0.64--0.04)	**2.86E-02**	**4.82E-02**	-0.56	(-0.98--0.15)	**7.64E-03**	**1.33E-02**	same
PC(15-MHDA_18:2)	-1.86	(-2.67--1.05)	**9.80E-06**	**4.05E-05**	-1.20	(-1.96--0.42)	**2.53E-03**	**4.94E-03**	same
PC(17:0_18:2)	-1.54	(-2.08--1)	**3.86E-08**	**2.57E-07**	-1.17	(-1.68--0.65)	**1.30E-05**	**3.74E-05**	same
PC(36:0)	0.97	(0.36-1.59)	**2.00E-03**	**4.66E-03**	3.16	(2.47-3.84)	**7.15E-19**	**3.43E-17**	same
PC(18:0_18:1)	-1.34	(-1.89--0.78)	**3.35E-06**	**1.49E-05**	0.98	(0.36-1.59)	**1.86E-03**	**3.74E-03**	opposite
PC(16:0_20:3) (a)	-1.23	(-1.82--0.64)	**5.06E-05**	**1.75E-04**	-1.61	(-2.18--1.04)	**5.63E-08**	**2.33E-07**	same
PC(18:2_18:2)	-2.57	(-3.28--1.87)	**3.69E-12**	**9.32E-11**	-2.49	(-3.22--1.75)	**1.24E-10**	**7.80E-10**	same
PC(15:0_22:6)	-2.68	(-3.42--1.94)	**3.93E-12**	**9.43E-11**	-0.93	(-1.62--0.24)	**8.87E-03**	**1.52E-02**	same
PC(38:2)	-1.13	(-1.58--0.67)	**1.45E-06**	**6.94E-06**	0.67	(0.19-1.14)	**5.76E-03**	**1.03E-02**	opposite
PC(18:0_20:3)	1.23	(0.54-1.93)	**5.29E-04**	**1.41E-03**	3.69	(2.93-4.46)	**1.54E-20**	**1.06E-18**	same
PC(38:4) (b)	-0.97	(-1.66--0.28)	**5.93E-03**	**1.24E-02**	1.29	(0.57-2.02)	**4.90E-04**	**1.11E-03**	opposite
PC(38:6) (a)	-1.01	(-1.61--0.42)	**9.39E-04**	**2.36E-03**	-1.09	(-1.67--0.5)	**2.90E-04**	**6.81E-04**	same
PC(38:7)(c)	-1.58	(-2.17--0.99)	**2.34E-07**	**1.32E-06**	-0.64	(-1.21--0.06)	**2.93E-02**	**4.47E-02**	same
PC(39:5)(a)	-2.53	(-3.35--1.7)	**4.56E-09**	**3.53E-08**	-1.82	(-2.59--1.05)	**4.96E-06**	**1.51E-05**	same
PC(39:5)(b)	-2.31	(-3.04--1.58)	**1.68E-09**	**1.56E-08**	-0.86	(-1.58--0.13)	**2.03E-02**	**3.21E-02**	same
PC(15-MHDA_22:6)	-2.51	(-3.31--1.7)	**2.77E-09**	**2.33E-08**	-1.62	(-2.33--0.91)	**1.10E-05**	**3.17E-05**	same
PC(17:0_22:6)	-2.00	(-2.74--1.26)	**1.87E-07**	**1.08E-06**	-1.05	(-1.79--0.31)	**5.44E-03**	**9.82E-03**	same
PC(18:0_22:5) (n6)	-1.32	(-2.07--0.57)	**6.32E-04**	**1.67E-03**	1.17	(0.37-1.97)	**3.94E-03**	**7.42E-03**	opposite
PC(18:1_22:6) (a)	-1.70	(-2.24--1.16)	**1.69E-09**	**1.56E-08**	-0.86	(-1.44--0.27)	**4.10E-03**	**7.62E-03**	same
PC(40:8)	-1.77	(-2.3--1.24)	**1.86E-10**	**2.70E-09**	-1.88	(-2.39--1.37)	**2.15E-12**	**1.91E-11**	same
PC(44:12)	-2.40	(-3.44--1.34)	**1.14E-05**	**4.60E-05**	-2.61	(-3.74--1.46)	**1.06E-05**	**3.09E-05**	same
PC(O-16:0/16:0)	-0.78	(-1.21--0.35)	**3.82E-04**	**1.09E-03**	-0.75	(-1.18--0.33)	**5.02E-04**	**1.13E-03**	same
PC(O-34:1)	-0.84	(-1.27--0.41)	**1.54E-04**	**4.84E-04**	-1.21	(-1.62--0.79)	**2.44E-08**	**1.08E-07**	same

PC(O-34:2)	-2.02	(-2.62--1.41)	2.10E-10	2.80E-09	-1.75	(-2.35--1.15)	2.36E-08	1.05E-07	same
PC(O-36:0)	-1.30	(-1.8--0.81)	3.88E-07	2.12E-06	-0.84	(-1.33--0.35)	9.07E-04	1.95E-03	same
PC(O-18:0/18:1)	-1.51	(-1.98--1.03)	1.00E-09	1.02E-08	-1.48	(-1.92--1.04)	1.07E-10	6.87E-10	same
PC(O-18:1/18:1)	-1.20	(-1.66--0.74)	4.16E-07	2.24E-06	-1.71	(-2.13--1.29)	1.10E-14	1.42E-13	same
PC(O-18:0/18:2)	-2.36	(-2.92--1.79)	3.60E-15	1.73E-13	-1.89	(-2.38--1.39)	4.43E-13	4.43E-12	same
PC(O-18:1/18:2)	-1.41	(-1.96--0.86)	7.43E-07	3.71E-06	-1.34	(-1.85--0.82)	6.84E-07	2.35E-06	same
PC(O-40:5)	-0.80	(-1.3--0.3)	1.67E-03	3.98E-03	-0.88	(-1.34--0.42)	1.90E-04	4.60E-04	same
PC(O-18:0/22:6)	-0.69	(-1.25--0.12)	1.77E-02	3.15E-02	-1.09	(-1.63--0.55)	9.15E-05	2.31E-04	same
PC(P-16:0/14:0)	-1.27	(-1.86--0.68)	2.71E-05	9.86E-05	-1.18	(-1.79--0.56)	1.98E-04	4.77E-04	same
PC(P-16:0/16:0)	-0.71	(-1.21--0.2)	6.69E-03	1.37E-02	-1.03	(-1.54--0.53)	6.60E-05	1.72E-04	same
PC(P-16:0/16:1)	-0.71	(-1.31--0.11)	2.01E-02	3.52E-02	-0.83	(-1.39--0.26)	4.66E-03	8.63E-03	same
PC(P-16:0/18:0)	-1.20	(-1.59--0.81)	3.58E-09	2.91E-08	-1.47	(-1.84--1.09)	1.05E-13	1.20E-12	same
PC(P-16:0/18:1)	-1.21	(-1.75--0.67)	1.36E-05	5.30E-05	-2.19	(-2.7--1.69)	2.94E-16	6.14E-15	same
PC(P-16:0/18:2)	-1.10	(-1.68--0.5)	3.02E-04	8.73E-04	-2.03	(-2.57--1.48)	1.39E-12	1.28E-11	same
PC(P-16:0/18:3)	-1.64	(-2.27--1.01)	5.09E-07	2.66E-06	-1.42	(-1.99--0.84)	2.28E-06	7.17E-06	same
PC(P-35:2) (a)	-2.26	(-3.13--1.39)	5.70E-07	2.91E-06	-1.87	(-2.71--1.02)	1.87E-05	5.25E-05	same
PC(P-35:2) (b)	-1.83	(-2.55--1.1)	1.07E-06	5.20E-06	-2.14	(-2.83--1.45)	2.27E-09	1.13E-08	same
PC(P-18:1/18:1)	-1.20	(-1.72--0.68)	7.54E-06	3.17E-05	-1.58	(-2.08--1.09)	7.85E-10	4.18E-09	same
PC(P-18:0/18:2)	-1.93	(-2.51--1.36)	1.35E-10	2.16E-09	-2.58	(-3.1--2.06)	1.54E-20	1.06E-18	same
PC(P-38:5) (b)	-0.85	(-1.43--0.27)	4.44E-03	9.70E-03	-1.10	(-1.67--0.53)	1.76E-04	4.33E-04	same
PC(P-40:5) (b)	-0.80	(-1.37--0.24)	5.56E-03	1.18E-02	-1.41	(-1.93--0.9)	1.16E-07	4.47E-07	same
PE(18:0_20:3) (a)	1.68	(0.87-2.5)	4.94E-05	1.72E-04	2.90	(1.96-3.84)	1.44E-09	7.41E-09	same
PE(18:0_22:6)	1.34	(0.55-2.14)	8.34E-04	2.13E-03	1.67	(0.73-2.63)	5.37E-04	1.20E-03	same
PE(O-34:1)	-1.42	(-2--0.83)	2.58E-06	1.19E-05	-1.43	(-2.04--0.82)	6.33E-06	1.91E-05	same
PE(O-16:0/18:2)	-2.74	(-3.5--1.96)	1.39E-11	2.79E-10	-2.48	(-3.27--1.68)	2.70E-09	1.32E-08	same
PE(O-18:1/18:2)	-1.56	(-2.3--0.82)	4.20E-05	1.47E-04	-1.10	(-1.81--0.38)	2.71E-03	5.25E-03	same
PE(O-16:0/20:4)	-1.39	(-2.12--0.65)	2.38E-04	7.02E-04	-1.38	(-2.13--0.61)	4.51E-04	1.03E-03	same
PE(O-18:0/20:4)	-1.81	(-2.63--0.99)	2.02E-05	7.69E-05	-1.76	(-2.55--0.97)	1.81E-05	5.11E-05	same
PE(O-38:5) (a)	-1.44	(-2.16--0.71)	1.14E-04	3.65E-04	-0.89	(-1.6--0.18)	1.37E-02	2.25E-02	same
PE(O-38:5) (b)	-1.98	(-2.64--1.31)	9.88E-09	6.87E-08	-1.06	(-1.81--0.29)	6.73E-03	1.19E-02	same
PE(O-16:0/22:6)	-1.03	(-1.66--0.4)	1.52E-03	3.67E-03	-0.83	(-1.53--0.13)	2.05E-02	3.21E-02	same
PE(O-18:0/22:6)	-1.54	(-2.14--0.93)	1.02E-06	5.01E-06	-1.41	(-2.04--0.77)	1.78E-05	5.04E-05	same
PE(O-18:1/22:6)	-1.16	(-1.76--0.56)	1.73E-04	5.35E-04	-1.29	(-1.9--0.67)	5.13E-05	1.35E-04	same
PE(P-16:0/18:1)	-1.04	(-1.56--0.52)	1.03E-04	3.35E-04	-0.62	(-1.13--0.11)	1.76E-02	2.81E-02	same
PE(P-18:0/18:1)	-1.25	(-1.84--0.66)	3.98E-05	1.40E-04	-1.01	(-1.6--0.41)	9.72E-04	2.07E-03	same
PE(P-18:0/18:2)	-1.83	(-2.48--1.19)	4.04E-08	2.66E-07	-1.53	(-2.15--0.91)	1.62E-06	5.22E-06	same
PE(P-18:0/22:4)	-0.72	(-1.31--0.13)	1.74E-02	3.11E-02	-0.98	(-1.57--0.38)	1.48E-03	3.02E-03	same
PE(P-18:0/22:5) (n6)	-1.29	(-1.87--0.72)	1.35E-05	5.30E-05	-1.25	(-1.83--0.67)	3.16E-05	8.48E-05	same
PE(P-18:0/22:6)	-0.79	(-1.43--0.14)	1.80E-02	3.18E-02	-1.21	(-1.87--0.55)	3.71E-04	8.65E-04	same
PE(P-18:1/18:1)	-0.90	(-1.45--0.35)	1.43E-03	3.50E-03	-0.83	(-1.38--0.28)	3.06E-03	5.83E-03	same
PE(P-18:1/18:2)	-1.86	(-2.56--1.16)	3.27E-07	1.80E-06	-1.22	(-1.9--0.53)	5.96E-04	1.30E-03	same
PE(P-20:0/18:1)	-1.27	(-1.89--0.65)	7.01E-05	2.35E-04	-1.14	(-1.71--0.57)	1.01E-04	2.52E-04	same
PE(P-20:0/18:2)	-1.81	(-2.36--1.25)	3.40E-10	4.18E-09	-1.98	(-2.5--1.45)	1.04E-12	9.79E-12	same
PE(P-20:0/22:6)	-0.78	(-1.31--0.26)	3.71E-03	8.27E-03	-1.89	(-2.39--1.4)	3.83E-13	3.92E-12	same
PG(34:2)	-1.07	(-1.82--0.31)	6.14E-03	1.28E-02	1.17	(0.21-2.14)	1.72E-02	2.76E-02	opposite
PI(16:0/16:0)	-2.44	(-3.37--1.51)	4.46E-07	2.38E-06	1.75	(0.7-2.81)	1.12E-03	2.36E-03	opposite
PI(16:0_16:1)	-1.73	(-2.79--0.66)	1.69E-03	4.01E-03	3.85	(2.77-4.94)	3.76E-12	3.11E-11	opposite
PI(18:1_18:2)	-2.66	(-3.29--2.03)	1.11E-15	5.91E-14	-1.29	(-1.97--0.61)	2.16E-04	5.17E-04	same
PI(16:0_20:3) (a)	-2.16	(-2.82--1.5)	3.96E-10	4.64E-09	0.93	(0.22-1.65)	1.07E-02	1.78E-02	opposite
PI(16:0_20:3) (b)	-2.46	(-3.2--1.72)	1.99E-10	2.80E-09	0.86	(0.13-1.6)	2.06E-02	3.21E-02	opposite
PI(16:0_20:4)	-1.45	(-2.03--0.86)	1.88E-06	8.78E-06	0.99	(0.36-1.62)	2.13E-03	4.20E-03	opposite
PI(38:6)	-2.63	(-3.27--1.98)	1.30E-14	5.19E-13	-0.89	(-1.56--0.21)	1.01E-02	1.70E-02	same
PI(18:0_22:5) (n3)	-1.73	(-2.4--1.06)	5.69E-07	2.91E-06	0.86	(0.2-1.52)	1.06E-02	1.78E-02	opposite

Table S3B. The overlapping lipid species between the ppBMI and birth weight studies. Related to Figure 3.

	ppBMI (antenatal)				Birth Weight (cord blood)				
Lipid Sepcies	%change in lipid concentration per unit BMI	95%CI	p-value	p-value(BH)	%change in lipid concentration per 100 grams	95%CI	p-value	p-value(BH)	Trend
AC(14:1)	1.38	(0.22-2.56)	1.98E-02	3.50E-02	-1.08	(-1.73--0.37)	3.63E-03	8.13E-03	opposite
AC(16:1)	1.19	(0.37-2.02)	4.39E-03	9.66E-03	-1.00	(-1.64--0.31)	5.17E-03	1.12E-02	opposite
CE(16:1)	-1.06	(-1.82--0.29)	7.23E-03	1.46E-02	0.98	(0.31-1.69)	3.55E-03	8.00E-03	opposite
CE(20:2)	-1.33	(-1.74--0.91)	7.59E-10	7.92E-09	0.79	(0.16-1.46)	1.36E-02	2.71E-02	opposite
CE(20:5)	-1.71	(-3.04--0.36)	1.31E-02	2.41E-02	-2.42	(-3.39--1.3)	9.07E-05	2.59E-04	same
CE(22:6)	-1.55	(-2.27--0.82)	3.29E-05	1.18E-04	-1.97	(-2.73--1.12)	1.95E-05	6.12E-05	same
CE(24:5)	-2.01	(-2.72--1.3)	4.73E-08	3.03E-07	-2.88	(-3.68--1.98)	3.40E-08	1.41E-07	same
CE(24:6)	-2.55	(-3.28--1.82)	2.50E-11	4.44E-10	-1.14	(-2.05--0.14)	2.66E-02	4.98E-02	same
DG(16:0_18:1)	1.33	(0.59-2.07)	4.36E-04	1.22E-03	-1.91	(-2.57--1.19)	1.35E-06	4.62E-06	opposite
DG(16:0_18:2)	0.92	(0.21-1.65)	1.15E-02	2.16E-02	-3.07	(-3.76--2.31)	1.09E-11	6.22E-11	opposite
DG(18:0_18:1)	1.36	(0.69-2.04)	7.91E-05	2.60E-04	-1.29	(-1.99--0.53)	1.23E-03	2.94E-03	opposite
DG(18:1_18:1)	1.37	(0.64-2.1)	2.23E-04	6.69E-04	-1.85	(-2.5--1.16)	1.20E-06	4.16E-06	opposite
DG(18:0_18:2)	0.99	(0.23-1.76)	1.04E-02	1.97E-02	-1.62	(-2.52--0.61)	2.43E-03	5.62E-03	opposite
DG(18:1_18:2)	0.95	(0.22-1.68)	1.02E-02	1.97E-02	-3.15	(-3.76--2.48)	8.45E-15	7.12E-14	opposite
DG(16:0_20:4)	1.19	(0.26-2.12)	1.22E-02	2.27E-02	-2.06	(-2.83--1.21)	9.20E-06	2.95E-05	opposite
DG(18:1_20:4)	1.14	(0.4-1.88)	2.42E-03	5.53E-03	-2.06	(-2.71--1.36)	1.08E-07	4.14E-07	opposite
DG(18:1_22:6)	1.53	(0.47-2.61)	4.63E-03	9.97E-03	-3.49	(-4.15--2.76)	7.44E-15	6.49E-14	opposite
DG(18:2_22:6)	1.52	(0.42-2.63)	6.55E-03	1.35E-02	-4.00	(-4.74--3.16)	9.00E-14	6.75E-13	opposite
TG(48:0) [NL-18:0]	-1.56	(-2.43--0.68)	5.76E-04	1.53E-03	-1.44	(-2.28--0.51)	3.14E-03	7.14E-03	same
TG(48:2) [NL-14:1]	-1.20	(-2.25--0.13)	2.78E-02	4.70E-02	-3.06	(-3.81--2.23)	4.50E-10	2.23E-09	same
TG(48:2) [NL-18:2]	-1.16	(-2.2--0.12)	2.91E-02	4.87E-02	-4.14	(-4.86--3.32)	5.42E-15	4.91E-14	same
TG(48:3) [NL-14:0]	-1.51	(-2.64--0.37)	9.55E-03	1.85E-02	-4.04	(-4.73--3.26)	1.02E-15	1.04E-14	same
TG(48:3) [NL-18:3]	-1.81	(-3.07--0.53)	5.76E-03	1.21E-02	-3.23	(-3.91--2.48)	8.75E-13	5.83E-12	same
TG(50:1) [NL-16:0]	0.83	(0.22-1.43)	7.27E-03	1.46E-02	-2.49	(-3.25--1.64)	2.17E-07	8.15E-07	opposite
TG(50:1) [NL-18:1]	0.76	(0.18-1.34)	1.03E-02	1.97E-02	-2.47	(-3.24--1.62)	2.61E-07	9.63E-07	opposite
TG(50:2) [NL-14:0]	-0.65	(-1.21--0.07)	2.69E-02	4.57E-02	-3.34	(-4.1--2.48)	1.16E-10	6.01E-10	same
TG(50:2) [NL-18:2]	0.66	(0.08-1.24)	2.58E-02	4.41E-02	-4.17	(-4.92--3.31)	5.26E-14	4.14E-13	opposite
TG(52:1) [NL-18:0]	1.38	(0.59-2.18)	6.58E-04	1.73E-03	-1.86	(-2.76--0.83)	6.81E-04	1.69E-03	opposite
TG(52:1) [NL-18:1]	1.45	(0.69-2.23)	2.04E-04	6.17E-04	-1.80	(-2.71--0.77)	9.96E-04	2.41E-03	opposite
TG(52:2) [NL-16:0]	0.80	(0.4-1.21)	1.01E-04	3.29E-04	-2.79	(-3.44--2.07)	3.12E-11	1.68E-10	opposite
TG(52:3) [NL-18:2]	0.76	(0.32-1.2)	8.08E-04	2.08E-03	-4.40	(-5.07--3.64)	3.04E-18	4.87E-17	opposite
TG(52:4) [NL-18:2]	1.20	(0.44-1.96)	1.92E-03	4.52E-03	-4.97	(-5.63--4.21)	2.07E-20	5.23E-19	opposite
TG(53:2) [NL-18:1]	0.88	(0.2-1.56)	1.08E-02	2.03E-02	-2.69	(-3.33--1.98)	5.26E-11	2.81E-10	opposite
TG(54:1) [NL-18:1]	0.99	(0.32-1.66)	3.57E-03	8.00E-03	-1.64	(-2.56--0.59)	2.89E-03	6.61E-03	opposite
TG(54:2) [NL-18:0]	1.41	(0.72-2.1)	6.55E-05	2.23E-04	-1.85	(-2.69--0.91)	2.40E-04	6.39E-04	opposite
TG(54:3) [NL-18:2]	0.72	(0.14-1.3)	1.43E-02	2.59E-02	-3.06	(-3.75--2.3)	1.28E-11	7.23E-11	opposite
TG(54:5) [NL-20:4]	1.04	(0.38-1.71)	1.91E-03	4.51E-03	-3.73	(-4.49--2.86)	4.40E-12	2.64E-11	opposite
TG(54:6) [NL-20:4]	1.16	(0.33-2)	6.24E-03	1.29E-02	-4.11	(-4.83--3.28)	1.19E-14	9.81E-14	opposite
TG(54:6) [NL-22:6]	1.79	(0.57-3.02)	4.10E-03	9.06E-03	-5.16	(-5.83--4.38)	1.98E-20	5.23E-19	opposite
TG(56:6) [NL-20:4]	1.34	(0.81-1.87)	7.12E-07	3.60E-06	-3.00	(-3.77--2.15)	1.89E-09	8.92E-09	opposite
TG(56:7) [NL-20:4]	1.02	(0.35-1.7)	2.84E-03	6.40E-03	-3.66	(-4.41--2.81)	3.38E-12	2.08E-11	opposite
TG(56:7) [NL-22:6]	1.93	(0.84-3.04)	4.97E-04	1.35E-03	-5.07	(-5.72--4.33)	1.85E-21	7.12E-20	opposite
TG(56:8) [NL-22:6]	1.76	(0.54-2.99)	4.49E-03	9.76E-03	-5.36	(-5.97--4.65)	1.69E-24	1.02E-22	opposite
TG(58:8) [NL-22:6]	1.46	(0.38-2.56)	7.98E-03	1.58E-02	-4.50	(-5.16--3.76)	3.67E-19	7.05E-18	opposite
TG(O-54:2) [NL-18:1]	0.85	(0.18-1.53)	1.33E-02	2.43E-02	-2.66	(-3.31--1.95)	1.06E-10	5.53E-10	opposite
Cer(d16:1/22:0)	-0.73	(-1.33--0.13)	1.79E-02	3.18E-02	-1.03	(-1.75--0.25)	1.07E-02	2.19E-02	same
Cer(d17:1/24:0)	-2.19	(-2.76--1.61)	4.35E-13	1.39E-11	-1.07	(-1.78--0.28)	8.53E-03	1.79E-02	same
Cer(d19:1/24:0)	-1.22	(-2.12--0.32)	8.01E-03	1.58E-02	-1.23	(-2.03--0.34)	7.85E-03	1.67E-02	same
Cer(m18:0/24:1)	1.15	(0.26-2.04)	1.11E-02	2.08E-02	-1.46	(-2.42--0.37)	9.81E-03	2.02E-02	opposite
Cer(m18:1/24:1)	1.37	(0.47-2.27)	2.70E-03	6.11E-03	-2.16	(-2.91--1.32)	3.30E-06	1.10E-05	opposite
GM3(d18:1/18:0)	-1.16	(-1.7--0.62)	2.65E-05	9.76E-05	-0.78	(-1.37--0.16)	1.53E-02	3.01E-02	same
SM(d17:1/14:0)	-1.88	(-2.45--1.3)	3.06E-10	3.86E-09	-0.98	(-1.49--0.44)	5.42E-04	1.36E-03	same
SM(d17:1/16:0)	-1.40	(-1.85--0.95)	2.57E-09	2.25E-08	-0.98	(-1.47--0.47)	2.86E-04	7.59E-04	same
SM(37:1)	-0.75	(-1.3--0.2)	7.20E-03	1.46E-02	-0.82	(-1.45--0.15)	1.76E-02	3.42E-02	same
SM(37:2)	-1.21	(-1.81--0.6)	1.07E-04	3.46E-04	-0.76	(-1.35--0.12)	2.06E-02	3.93E-02	same
SM(40:3) (b)	-0.91	(-1.46--0.36)	1.23E-03	3.06E-03	-0.89	(-1.5--0.24)	8.17E-03	1.72E-02	same
SM(43:1)	-1.62	(-2.09--1.14)	7.20E-11	1.19E-09	-1.04	(-1.74--0.27)	9.36E-03	1.94E-02	same
LPC(15:0) [sn2]	-1.50	(-2.11--0.88)	2.82E-06	1.29E-05	1.04	(0.46-1.66)	3.62E-04	9.45E-04	opposite
LPC(15:0) [sn1]	-1.28	(-1.83--0.72)	8.57E-06	3.58E-05	0.76	(0.24-1.32)	4.17E-03	9.24E-03	opposite
LPC(17:1) [sn1] (a) / LPC(17:1) [sn2] (b)	-0.95	(-1.52--0.37)	1.28E-03	3.14E-03	1.72	(1.09-2.4)	3.10E-08	1.29E-07	opposite
LPC(18:1) [sn2]	-0.59	(-1.11--0.07)	2.69E-02	4.57E-02	3.61	(2.91-4.34)	4.61E-28	5.53E-26	opposite
LPC(18:2) [sn2]	-0.64	(-1.13--0.15)	1.04E-02	1.97E-02	2.93	(2.22-3.69)	4.36E-18	6.75E-17	opposite
LPC(18:3) [sn2] (a)	-1.54	(-2.23--0.85)	1.68E-05	6.51E-05	2.31	(1.65-3.01)	5.03E-13	3.55E-12	opposite

LPC(18:3) [sn1] (a)/LPC(18:3) [sn2] (b)	-0.95	(-1.67--0.23)	**9.74E-03**	**1.88E-02**	4.43	(3.51-5.42)	**1.76E-25**	**1.41E-23**	opposite
LPC(18:3) (a) [sn1] [104_sn1]	-0.94	(-1.68--0.2)	**1.34E-02**	**2.43E-02**	4.09	(3.21-5.02)	**1.27E-23**	**6.77E-22**	opposite
LPC(20:1) [sn2]	-0.60	(-1.07--0.14)	**1.07E-02**	**2.02E-02**	1.44	(0.92-1.98)	**1.54E-08**	**6.60E-08**	opposite
LPC(20:1) [sn1]	-0.62	(-1.08--0.15)	**9.49E-03**	**1.84E-02**	1.75	(1.27-2.25)	**1.55E-13**	**1.12E-12**	opposite
LPC(20:2) [sn2]	-0.91	(-1.41--0.4)	**4.81E-04**	**1.32E-03**	3.83	(3.05-4.65)	**3.73E-26**	**3.58E-24**	opposite
LPC(20:2) [sn1]	-0.63	(-1.11--0.16)	**9.14E-03**	**1.78E-02**	3.35	(2.66-4.06)	**4.61E-25**	**3.16E-23**	opposite
LPC(20:3) [sn2]	1.05	(0.4-1.7)	**1.48E-03**	**3.59E-03**	2.58	(1.91-3.3)	**1.79E-15**	**1.72E-14**	same
LPC(20:3) [sn1]	1.28	(0.59-1.97)	**2.78E-04**	**8.08E-04**	3.27	(2.53-4.05)	**7.83E-21**	**2.50E-19**	same
LPC(20:3) [104_sn1]	1.42	(0.72-2.12)	**7.46E-05**	**2.47E-04**	3.22	(2.49-3.99)	**9.49E-21**	**2.85E-19**	same
LPC(20:4) [sn2]	0.85	(0.33-1.37)	**1.45E-03**	**3.53E-03**	2.39	(1.75-3.06)	**6.31E-15**	**5.61E-14**	same
LPC(20:4) [sn1]	1.36	(0.79-1.93)	**3.16E-06**	**1.42E-05**	2.66	(1.98-3.38)	**2.33E-16**	**2.60E-15**	same
LPC(22:5) [sn2] (n3)	-0.98	(-1.6--0.35)	**2.21E-03**	**5.12E-03**	1.73	(0.89-2.63)	**2.63E-05**	**8.04E-05**	opposite
LPC(22:5) [sn1] (n3)/LPC(22:5) [sn2] (n6)	-0.84	(-1.42--0.26)	**4.63E-03**	**9.97E-03**	2.12	(1.41-2.88)	**6.79E-10**	**3.29E-09**	opposite
LPC(O-18:1)	0.52	(0.07-0.97)	**2.38E-02**	**4.13E-02**	1.82	(1.3-2.37)	**7.59E-13**	**5.13E-12**	same
LPC(O-22:1)	-1.62	(-2.15--1.1)	**2.73E-09**	**2.33E-08**	1.29	(0.72-1.9)	**6.07E-06**	**1.97E-05**	opposite
LPC(P-18:1)	0.95	(0.36-1.54)	**1.57E-03**	**3.78E-03**	1.25	(0.59-1.95)	**1.32E-04**	**3.64E-04**	same
LPC(P-20:0)	-0.73	(-1.3--0.15)	**1.34E-02**	**2.43E-02**	2.02	(1.36-2.71)	**3.08E-10**	**1.54E-09**	opposite
LPE(16:0) [sn2]	-0.58	(-1.09--0.08)	**2.45E-02**	**4.23E-02**	2.24	(1.6-2.92)	**6.15E-13**	**4.28E-12**	opposite
LPE(18:1) [sn2]	-0.78	(-1.33--0.24)	**5.15E-03**	**1.10E-02**	2.54	(1.88-3.24)	**1.28E-15**	**1.28E-14**	opposite
LPE(18:1) [sn1]	-0.74	(-1.28--0.2)	**7.22E-03**	**1.46E-02**	2.94	(2.26-3.65)	**1.02E-19**	**2.33E-18**	opposite
LPE(18:2) [sn2]	-0.93	(-1.59--0.27)	**6.21E-03**	**1.29E-02**	3.65	(2.71-4.66)	**9.44E-17**	**1.10E-15**	opposite
LPE(18:2) [sn1]	-0.96	(-1.63--0.29)	**5.28E-03**	**1.12E-02**	3.74	(2.85-4.69)	**1.99E-19**	**4.16E-18**	opposite
PC(33:0) (b)	-1.78	(-2.34--1.22)	**1.18E-09**	**1.18E-08**	-1.45	(-2.17--0.66)	**5.09E-04**	**1.30E-03**	same
PC(33:2)	-2.27	(-2.89--1.64)	**6.00E-12**	**1.37E-10**	-1.25	(-1.87--0.6)	**3.06E-04**	**8.08E-04**	same
PC(15-MHDA_18:1)	-2.51	(-3.31--1.72)	**1.39E-09**	**1.34E-08**	-1.26	(-1.99--0.45)	**2.77E-03**	**6.37E-03**	same
PC(17:0_18:1)	-1.99	(-2.49--1.47)	**1.15E-13**	**3.93E-12**	-1.06	(-1.56--0.54)	**1.14E-04**	**3.25E-04**	same
PC(15-MHDA_18:2)	-1.86	(-2.67--1.05)	**9.80E-06**	**4.05E-05**	-1.39	(-2.07--0.66)	**3.55E-04**	**9.32E-04**	same
PC(17:0_18:2)	-1.54	(-2.08--1)	**3.86E-08**	**2.57E-07**	-1.28	(-1.84--0.68)	**5.78E-05**	**1.69E-04**	same
PC(17:1_18:2)	-1.21	(-1.75--0.67)	**1.36E-05**	**5.30E-05**	-0.72	(-1.27--0.14)	**1.54E-02**	**3.03E-02**	same
PC(15:0_20:4)	-1.28	(-1.97--0.58)	**3.68E-04**	**1.06E-03**	-1.32	(-1.88--0.72)	**3.80E-05**	**1.13E-04**	same
PC(35:5)	-1.57	(-2.8--0.32)	**1.38E-02**	**2.50E-02**	-2.33	(-3.15--1.42)	**4.33E-06**	**1.41E-05**	same
PC(36:6)	-2.22	(-3.03--1.4)	**1.62E-07**	**9.83E-07**	-0.83	(-1.42--0.2)	**1.07E-02**	**2.19E-02**	same
PC(15:0_22:6)	-2.68	(-3.42--1.94)	**3.93E-12**	**9.43E-11**	-1.88	(-2.45--1.28)	**1.58E-08**	**6.70E-08**	same
PC(38:2)	-1.13	(-1.58--0.67)	**1.45E-06**	**6.94E-06**	0.92	(0.32-1.55)	**2.19E-03**	**5.11E-03**	opposite
PC(18:0_20:4)	0.98	(0.35-1.61)	**2.29E-03**	**5.29E-03**	-1.07	(-1.65--0.46)	**8.92E-04**	**2.21E-03**	opposite
PC(38:5) (a)	-1.09	(-1.59--0.58)	**2.73E-05**	**9.86E-05**	-1.17	(-1.68--0.63)	**3.91E-05**	**1.15E-04**	same
PC(16:0_22:6)	-1.01	(-1.64--0.37)	**1.94E-03**	**4.55E-03**	-1.25	(-1.93--0.5)	**1.40E-03**	**3.31E-03**	same
PC(16:1_22:6)	-1.64	(-2.33--0.96)	**3.49E-06**	**1.54E-05**	-1.04	(-1.56--0.48)	**4.09E-04**	**1.06E-03**	same
PC(38:7)(c)	-1.58	(-2.17--0.99)	**2.34E-07**	**1.32E-06**	-0.88	(-1.37--0.37)	**1.02E-03**	**2.45E-03**	same
PC(39:5)(a)	-2.53	(-3.35--1.7)	**4.56E-09**	**3.53E-08**	-1.98	(-2.62--1.29)	**2.49E-07**	**9.28E-07**	same
PC(39:5)(b)	-2.31	(-3.04--1.58)	**1.68E-09**	**1.56E-08**	-1.95	(-2.55--1.29)	**7.31E-08**	**2.90E-07**	same
PC(15-MHDA_22:6)	-2.51	(-3.31--1.7)	**2.77E-09**	**2.33E-08**	-2.07	(-2.73--1.36)	**1.67E-07**	**6.31E-07**	same
PC(17:0_22:6)	-2.00	(-2.74--1.26)	**1.87E-07**	**1.08E-06**	-2.24	(-2.78--1.65)	**1.65E-11**	**9.21E-11**	same
PC(40:8)	-1.77	(-2.3--1.24)	**1.86E-10**	**2.70E-09**	0.83	(0.2-1.5)	**9.19E-03**	**1.92E-02**	opposite
PC(44:12)	-2.40	(-3.44--1.34)	**1.14E-05**	**4.60E-05**	-1.02	(-1.76--0.22)	**1.39E-02**	**2.75E-02**	same
PC(O-18:0/18:1)	-1.51	(-1.98--1.03)	**1.00E-09**	**1.02E-08**	1.31	(0.62-2.05)	**1.35E-04**	**3.71E-04**	opposite
PC(O-18:1/18:1)	-1.20	(-1.66--0.74)	**4.16E-07**	**2.24E-06**	0.71	(0.11-1.35)	**2.01E-02**	**3.86E-02**	opposite
PC(O-36:5)	-1.11	(-2.09--0.12)	**2.87E-02**	**4.82E-02**	-1.06	(-1.73--0.34)	**4.52E-03**	**9.91E-03**	same
PC(O-18:0/22:6)	-0.69	(-1.25--0.12)	**1.77E-02**	**3.15E-02**	-0.93	(-1.43--0.39)	**9.93E-04**	**2.41E-03**	same
PC(P-16:0/18:2)	-1.10	(-1.68--0.5)	**3.02E-04**	**8.73E-04**	-1.30	(-1.9--0.66)	**1.43E-04**	**3.91E-04**	same
PC(P-35:2) (b)	-1.83	(-2.55--1.1)	**1.07E-06**	**5.20E-06**	-1.45	(-2.06--0.79)	**3.89E-05**	**1.15E-04**	same
PC(P-18:1/18:1)	-1.20	(-1.72--0.68)	**7.54E-06**	**3.17E-05**	1.22	(0.39-2.12)	**3.50E-03**	**7.92E-03**	opposite
PC(P-17:0/20:4) (a)	-1.01	(-1.8--0.22)	**1.29E-02**	**2.38E-02**	-1.57	(-2.22--0.86)	**3.59E-05**	**1.08E-04**	same
PC(P-38:5) (b)	-0.85	(-1.43--0.27)	**4.44E-03**	**9.70E-03**	-0.87	(-1.5--0.2)	**1.16E-02**	**2.34E-02**	same
PC(P-40:5) (b)	-0.80	(-1.37--0.24)	**5.56E-03**	**1.18E-02**	-0.80	(-1.31--0.26)	**4.26E-03**	**9.37E-03**	same
PE(17:0_18:2)	-1.21	(-1.9--0.52)	**6.65E-04**	**1.74E-03**	-0.85	(-1.35--0.31)	**2.33E-03**	**5.41E-03**	same
PE(18:0_22:6)	1.34	(0.55-2.14)	**8.34E-04**	**2.13E-03**	-1.61	(-2.34--0.8)	**2.02E-04**	**5.43E-04**	opposite
PE(P-16:0/18:1)	-1.04	(-1.56--0.52)	**1.03E-04**	**3.35E-04**	1.69	(0.98-2.44)	**1.11E-06**	**3.90E-06**	opposite
PE(P-16:0/22:5) (n6)	-0.76	(-1.4--0.12)	**2.07E-02**	**3.60E-02**	0.91	(0.1-1.79)	**2.66E-02**	**4.98E-02**	opposite
PE(P-18:0/18:1)	-1.25	(-1.84--0.66)	**3.98E-05**	**1.40E-04**	1.01	(0.24-1.85)	**9.34E-03**	**1.94E-02**	opposite
PE(P-18:0/20:5)	-1.63	(-2.99--0.26)	**2.00E-02**	**3.52E-02**	-1.97	(-3.3--0.38)	**1.77E-02**	**3.42E-02**	same
PE(P-20:0/22:6)	-0.78	(-1.31--0.26)	**3.71E-03**	**8.27E-03**	-0.70	(-1.25--0.11)	**2.13E-02**	**4.05E-02**	same
PI(34:0)	-2.66	(-3.58--1.72)	**4.56E-08**	**2.96E-07**	-1.86	(-2.99--0.54)	**7.41E-03**	**1.58E-02**	same
PI(17:0_18:1)	-2.37	(-2.95--1.79)	**6.39E-15**	**2.79E-13**	-1.30	(-2.26--0.22)	**1.94E-02**	**3.75E-02**	same
PI(17:0_18:2)	-2.70	(-3.33--2.07)	**4.72E-16**	**2.83E-14**	-1.91	(-2.87--0.83)	**9.67E-04**	**2.37E-03**	same
PI(18:0_18:1)	-1.94	(-2.65--1.23)	**1.16E-07**	**7.23E-07**	-1.18	(-1.84--0.46)	**1.71E-03**	**4.02E-03**	same
PI(36:2)	-1.93	(-2.51--1.34)	**2.53E-10**	**3.28E-09**	-1.22	(-1.78--0.62)	**1.28E-04**	**3.56E-04**	same
PI(18:1_18:2)	-2.66	(-3.29--2.03)	**1.11E-15**	**5.91E-14**	-1.47	(-1.98--0.93)	**4.93E-07**	**1.78E-06**	same

PI(16:0_20:3) (b)	-2.46	(-3.2--1.72)	**1.99E-10**	**2.80E-09**	2.99	(1.94-4.14)	**2.09E-09**	**9.74E-09**	opposite
PI(18:0_20:2)	-2.66	(-3.36--1.95)	**6.56E-13**	**1.85E-11**	2.45	(1.2-3.85)	**5.83E-05**	**1.70E-04**	opposite
PI(18:0_20:4)	-0.79	(-1.31--0.28)	**2.68E-03**	**6.09E-03**	-0.59	(-1.01--0.14)	**1.08E-02**	**2.19E-02**	same
PI(38:6)	-2.63	(-3.27--1.98)	**1.30E-14**	**5.19E-13**	-0.92	(-1.44--0.37)	**1.31E-03**	**3.11E-03**	same
PI(18:0_22:4)	-1.24	(-1.89--0.58)	**2.27E-04**	**6.78E-04**	1.20	(0.52-1.92)	**4.03E-04**	**1.05E-03**	opposite
PI(18:0_22:5) (n3)	-1.73	(-2.4--1.06)	**5.69E-07**	**2.91E-06**	-1.24	(-1.88--0.56)	**5.87E-04**	**1.47E-03**	same
PI(18:0_22:6)	-2.03	(-2.68--1.38)	**2.31E-09**	**2.05E-08**	-1.11	(-1.63--0.55)	**1.59E-04**	**4.30E-04**	same

Table S3C. The overlapping lipid species between the birth weight and 6-year-old child BMI studies. Related to Figure 3.

	Birth Weight (cord blood)				Child BMI (6-year-old)				
Lipid Species	%change in lipid concentration per 100 grams	95%CI	p-value	p-value(BH)	%change in lipid concentration per unit BMI	95%CI	p-value	p-value(BH)	Trend
AC(18:0)	-0.78	(-1.3--0.24)	**5.68E-03**	**1.22E-02**	-1.98	(-3.33--0.62)	**4.60E-03**	**1.97E-02**	same
CE(16:1)	0.98	(0.31-1.69)	**3.55E-03**	**8.00E-03**	5.77	(4.04-7.53)	**9.25E-11**	**2.22E-08**	same
DG(16:0_16:1)	-1.32	(-2.11--0.45)	**3.64E-03**	**8.13E-03**	6.21	(3.58-8.89)	**3.25E-06**	**8.67E-05**	opposite
DG(14:0_18:2)	-3.08	(-4.04--1.96)	**1.68E-06**	**5.70E-06**	6.70	(3.02-10.51)	**3.23E-04**	**2.28E-03**	opposite
DG(16:0_18:1)	-1.91	(-2.57--1.19)	**1.35E-06**	**4.62E-06**	3.90	(1.38-6.49)	**2.40E-03**	**1.18E-02**	opposite
DG(16:1_18:1)	-2.08	(-2.77--1.32)	**7.91E-07**	**2.84E-06**	6.17	(3.26-9.17)	**2.92E-05**	**4.64E-04**	opposite
DG(16:0_18:2)	-3.07	(-3.76--2.31)	**1.09E-11**	**6.22E-11**	4.00	(1.57-6.48)	**1.20E-03**	**6.49E-03**	opposite
DG(18:0_18:1)	-1.29	(-1.99--0.53)	**1.23E-03**	**2.94E-03**	4.06	(1.71-6.46)	**6.87E-04**	**4.23E-03**	opposite
DG(18:0_18:2)	-1.62	(-2.52--0.61)	**2.43E-03**	**5.62E-03**	3.76	(1.3-6.29)	**2.68E-03**	**1.27E-02**	opposite
DG(16:0_20:4)	-2.06	(-2.83--1.21)	**9.20E-06**	**2.95E-05**	4.97	(2.34-7.66)	**1.98E-04**	**1.61E-03**	opposite
DG(18:1_20:4)	-2.06	(-2.71--1.36)	**1.08E-07**	**4.14E-07**	3.90	(1.68-6.16)	**5.47E-04**	**3.60E-03**	opposite
DG(16:0_22:5)	-1.95	(-2.72--1.09)	**2.99E-05**	**9.02E-05**	4.60	(2.03-7.23)	**4.22E-04**	**2.89E-03**	opposite
DG(18:2_20:4)	-3.14	(-3.87--2.33)	**7.74E-11**	**4.08E-10**	4.23	(1.78-6.73)	**6.78E-04**	**4.23E-03**	opposite
DG(16:0_22:6)	-4.09	(-4.76--3.33)	**6.80E-17**	**8.37E-16**	5.50	(2.13-8.99)	**1.31E-03**	**6.84E-03**	opposite
DG(18:1_22:6)	-3.49	(-4.15--2.76)	**7.44E-15**	**6.49E-14**	4.14	(1.32-7.04)	**3.88E-03**	**1.73E-02**	opposite
DG(18:2_22:6)	-4.00	(-4.74--3.16)	**9.00E-14**	**6.75E-13**	4.79	(1.45-8.24)	**4.80E-03**	**2.04E-02**	opposite
TG(48:0) [NL-18:0]	-1.44	(-2.28--0.51)	**3.14E-03**	**7.14E-03**	7.45	(3.74-11.29)	**7.09E-05**	**8.54E-04**	opposite
TG(48:1) [NL-16:1]	-2.43	(-3.32--1.41)	**1.61E-05**	**5.11E-05**	9.58	(5.88-13.41)	**2.99E-07**	**1.32E-05**	opposite
TG(48:1) [NL-18:1]	-3.15	(-3.94--2.26)	**2.16E-09**	**9.86E-09**	8.20	(3.97-12.59)	**1.20E-04**	**1.17E-03**	opposite
TG(48:2) [NL-14:0]	-3.76	(-4.46--2.97)	**4.13E-14**	**3.31E-13**	8.73	(4.41-13.22)	**6.10E-05**	**7.91E-04**	opposite
TG(48:2) [NL-14:1]	-3.06	(-3.81--2.23)	**4.50E-10**	**2.23E-09**	8.90	(4.81-13.16)	**1.61E-05**	**3.36E-04**	opposite
TG(48:2) [NL-16:1]	-2.85	(-3.65--1.96)	**3.48E-08**	**1.43E-07**	10.74	(6.57-15.07)	**3.03E-07**	**1.32E-05**	opposite
TG(48:2) [NL-18:2]	-4.14	(-4.86--3.32)	**5.42E-15**	**4.91E-14**	8.58	(4.3-13.04)	**7.11E-05**	**8.54E-04**	opposite
TG(48:3) [NL-14:0]	-4.04	(-4.73--3.26)	**1.02E-15**	**1.04E-14**	9.53	(5.17-14.08)	**1.43E-05**	**3.28E-04**	opposite
TG(48:3) [NL-16:1]	-2.89	(-3.59--2.12)	**1.90E-10**	**9.60E-10**	8.59	(4.99-12.31)	**2.28E-06**	**6.49E-05**	opposite
TG(48:3) [NL-18:3]	-3.23	(-3.91--2.48)	**8.75E-13**	**5.83E-12**	8.18	(3.69-12.86)	**3.09E-04**	**2.27E-03**	opposite
TG(49:1) [NL-17:1]	-2.19	(-2.84--1.47)	**5.29E-08**	**2.14E-07**	4.94	(2.47-7.47)	**8.54E-05**	**9.54E-04**	opposite
TG(50:1) [NL-14:0]	-1.46	(-2.35--0.47)	**4.76E-03**	**1.03E-02**	6.89	(3.47-10.42)	**7.00E-05**	**8.54E-04**	opposite
TG(50:1) [NL-16:0]	-2.49	(-3.25--1.64)	**2.17E-07**	**8.15E-07**	4.63	(2.1-7.22)	**3.13E-04**	**2.27E-03**	opposite
TG(50:1) [NL-18:1]	-2.47	(-3.24--1.62)	**2.61E-07**	**9.63E-07**	4.17	(1.73-6.68)	**7.99E-04**	**4.74E-03**	opposite
TG(50:2) [NL-14:0]	-3.34	(-4.1--2.48)	**1.16E-10**	**6.01E-10**	4.36	(1.14-7.69)	**7.83E-03**	**3.01E-02**	opposite
TG(50:2) [NL-16:1]	-3.01	(-3.81--2.11)	**1.08E-08**	**4.67E-08**	6.45	(3.42-9.58)	**2.79E-05**	**4.62E-04**	opposite
TG(50:2) [NL-18:1]	-3.12	(-3.91--2.21)	**4.54E-09**	**2.00E-08**	6.24	(3.2-9.38)	**5.20E-05**	**7.33E-04**	opposite
TG(50:2) [NL-18:2]	-4.17	(-4.92--3.31)	**5.26E-14**	**4.14E-13**	4.53	(2.11-7)	**2.30E-04**	**1.78E-03**	opposite
TG(50:3) [NL-14:0]	-4.25	(-4.95--3.45)	**4.04E-16**	**4.31E-15**	5.47	(1.88-9.18)	**2.69E-03**	**1.27E-02**	opposite
TG(50:3) [NL-14:1]	-2.39	(-3.17--1.51)	**1.11E-06**	**3.90E-06**	4.17	(1.33-7.09)	**3.92E-03**	**1.73E-02**	opposite
TG(50:3) [NL-16:1]	-3.88	(-4.58--3.09)	**8.11E-15**	**6.95E-14**	8.28	(4.83-11.85)	**2.15E-06**	**6.49E-05**	opposite
TG(50:3) [NL-18:2]	-4.37	(-5.06--3.59)	**3.53E-17**	**4.70E-16**	7.14	(3.78-10.61)	**2.72E-05**	**4.62E-04**	opposite
TG(50:3) [NL-18:3]	-3.81	(-4.54--2.98)	**2.31E-13**	**1.65E-12**	6.76	(3.2-10.44)	**1.78E-04**	**1.52E-03**	opposite
TG(50:4) [NL-14:0]	-4.31	(-4.97--3.57)	**2.52E-18**	**4.32E-17**	5.22	(1.61-8.96)	**4.43E-03**	**1.92E-02**	opposite
TG(50:4) [NL-18:3]	-3.93	(-4.56--3.22)	**9.72E-18**	**1.37E-16**	7.93	(4.25-11.74)	**2.01E-05**	**4.02E-04**	opposite
TG(50:4) [NL-20:4]	-3.52	(-4.34--2.57)	**6.23E-10**	**3.05E-09**	10.00	(5.69-14.48)	**3.96E-06**	**1.00E-04**	opposite
TG(51:1) [NL-17:0]	-2.72	(-3.44--1.91)	**4.45E-09**	**1.98E-08**	4.90	(2-7.89)	**8.93E-04**	**5.23E-03**	opposite
TG(51:2) [NL-17:0]	-3.40	(-4.11--2.6)	**2.54E-12**	**1.63E-11**	5.48	(2.52-8.52)	**2.67E-04**	**2.03E-03**	opposite
TG(51:2) [NL-17:1]	-2.89	(-3.53--2.19)	**2.44E-12**	**1.58E-11**	4.90	(2.22-7.65)	**3.17E-04**	**2.27E-03**	opposite
TG(52:1) [NL-18:0]	-1.86	(-2.76--0.83)	**6.81E-04**	**1.69E-03**	5.79	(2.8-8.86)	**1.32E-04**	**1.22E-03**	opposite
TG(52:1) [NL-18:1]	-1.80	(-2.71--0.77)	**9.96E-04**	**2.41E-03**	5.71	(2.83-8.68)	**9.59E-05**	**1.00E-03**	opposite
TG(52:2) [NL-18:2]	-2.75	(-3.58--1.81)	**3.20E-07**	**1.17E-06**	4.45	(2.1-6.85)	**1.98E-04**	**1.61E-03**	opposite
TG(52:3) [NL-16:1]	-3.37	(-4.1--2.56)	**1.00E-11**	**5.79E-11**	3.39	(0.97-5.88)	**5.95E-03**	**2.36E-02**	opposite
TG(52:4) [NL-16:1]	-4.52	(-5.17--3.78)	**1.14E-19**	**2.48E-18**	4.16	(1.08-7.34)	**7.91E-03**	**3.01E-02**	opposite
TG(52:4) [NL-18:3]	-4.11	(-4.78--3.35)	**9.43E-17**	**1.10E-15**	4.40	(1.43-7.46)	**3.60E-03**	**1.63E-02**	opposite
TG(52:5) [NL-18:3]	-4.16	(-4.81--3.43)	**2.87E-18**	**4.75E-17**	5.19	(2-8.47)	**1.36E-03**	**6.99E-03**	opposite
TG(52:5) [NL-20:4]	-3.63	(-4.4--2.74)	**2.19E-11**	**1.19E-10**	10.03	(6.39-13.8)	**4.75E-08**	**4.56E-06**	opposite
TG(52:5) [NL-20:5]	-4.43	(-5.13--3.64)	**4.66E-17**	**5.88E-16**	7.78	(4.53-11.13)	**2.30E-06**	**6.49E-05**	opposite
TG(54:1) [NL-18:1]	-1.64	(-2.56--0.59)	**2.89E-03**	**6.61E-03**	3.54	(1.13-6.02)	**3.96E-03**	**1.73E-02**	opposite
TG(54:2) [NL-18:0]	-1.85	(-2.69--0.91)	**2.40E-04**	**6.39E-04**	3.30	(0.94-5.71)	**6.02E-03**	**2.36E-02**	opposite

TG(54:3) [NL-18:2]	-3.06	(-3.75--2.3)	**1.28E-11**	**7.23E-11**	2.32	(0.48-4.19)	**1.32E-02**	**4.65E-02**	opposite
TG(54:4) [NL-20:3]	-2.73	(-3.52--1.84)	**8.04E-08**	**3.16E-07**	4.94	(2.41-7.54)	**1.24E-04**	**1.19E-03**	opposite
TG(54:5) [NL-20:4]	-3.73	(-4.49--2.86)	**4.40E-12**	**2.64E-11**	5.84	(3.43-8.3)	**1.90E-06**	**6.49E-05**	opposite
TG(54:6) [NL-20:4]	-4.11	(-4.83--3.28)	**1.19E-14**	**9.81E-14**	7.52	(4.68-10.44)	**1.91E-07**	**1.15E-05**	opposite
TG(54:6) [NL-20:5]	-5.04	(-5.69--4.28)	**7.74E-21**	**2.50E-19**	8.39	(5.04-11.85)	**7.54E-07**	**3.02E-05**	opposite
TG(54:6) [NL-22:6]	-5.16	(-5.83--4.38)	**1.98E-20**	**5.23E-19**	8.20	(4.48-12.06)	**1.29E-05**	**3.10E-04**	opposite
TG(54:7) [NL-20:5]	-4.97	(-5.63--4.22)	**1.23E-20**	**3.49E-19**	8.46	(5.22-11.8)	**2.49E-07**	**1.32E-05**	opposite
TG(54:7) [NL-22:6]	-4.92	(-5.56--4.18)	**1.93E-21**	**7.12E-20**	10.42	(6.51-14.47)	**1.23E-07**	**9.83E-06**	opposite
TG(56:6) [NL-20:4]	-3.00	(-3.77--2.15)	**1.89E-09**	**8.92E-09**	3.72	(1.91-5.57)	**5.78E-05**	**7.86E-04**	opposite
TG(56:6) [NL-22:5]	-3.83	(-4.52--3.06)	**4.63E-15**	**4.36E-14**	3.96	(1.82-6.14)	**2.87E-04**	**2.15E-03**	opposite
TG(56:7) [NL-20:4]	-3.66	(-4.41--2.81)	**3.38E-12**	**2.08E-11**	4.16	(1.73-6.64)	**7.60E-04**	**4.56E-03**	opposite
TG(56:7) [NL-20:5]	-3.79	(-4.51--2.97)	**1.22E-13**	**9.04E-13**	5.58	(2.64-8.6)	**1.82E-04**	**1.54E-03**	opposite
TG(56:7) [NL-22:5]	-4.29	(-4.93--3.57)	**2.43E-19**	**4.87E-18**	5.15	(2.46-7.91)	**1.64E-04**	**1.46E-03**	opposite
TG(56:7) [NL-22:6]	-5.07	(-5.72--4.33)	**1.85E-21**	**7.12E-20**	6.45	(3.01-10)	**2.16E-04**	**1.70E-03**	opposite
TG(56:8) [NL-20:4]	-2.95	(-3.72--2.08)	**5.67E-09**	**2.47E-08**	5.12	(2.54-7.75)	**9.23E-05**	**1.00E-03**	opposite
TG(56:8) [NL-20:5]	-4.13	(-4.79--3.37)	**3.84E-17**	**4.98E-16**	5.03	(2.01-8.13)	**1.03E-03**	**5.94E-03**	opposite
TG(56:8) [NL-22:6]	-5.36	(-5.97--4.65)	**1.69E-24**	**1.02E-22**	7.57	(4.02-11.24)	**2.51E-05**	**4.62E-04**	opposite
TG(56:9) [NL-22:6]	-4.88	(-5.5--4.17)	**1.57E-22**	**7.54E-21**	6.66	(3.29-10.14)	**9.49E-05**	**1.00E-03**	opposite
TG(58:10) [NL-22:6]	-4.48	(-5.11--3.76)	**3.35E-20**	**8.05E-19**	5.17	(1.89-8.56)	**1.90E-03**	**9.52E-03**	opposite
TG(58:8) [NL-22:6]	-4.50	(-5.16--3.76)	**3.67E-19**	**7.05E-18**	4.73	(1.39-8.18)	**5.34E-03**	**2.16E-02**	opposite
TG(58:9) [NL-22:6]	-4.76	(-5.39--4.06)	**4.43E-22**	**1.93E-20**	4.51	(1.17-7.97)	**7.98E-03**	**3.02E-02**	opposite
TG(O-50:1) [NL-17:1]	-2.21	(-2.88--1.47)	**9.38E-08**	**3.66E-07**	4.99	(2.47-7.57)	**1.01E-04**	**1.03E-03**	opposite
TG(O-52:2) [NL-17:1]	-2.91	(-3.55--2.2)	**4.20E-12**	**2.55E-11**	4.89	(2.19-7.66)	**3.63E-04**	**2.52E-03**	opposite
TG(O-52:2) [NL-18:1]	-1.84	(-2.72--0.85)	**5.37E-04**	**1.36E-03**	5.04	(2-8.16)	**1.08E-03**	**6.15E-03**	opposite
Cer(m18:0/22:0)	-1.25	(-2.16--0.23)	**1.75E-02**	**3.42E-02**	2.43	(0.5-4.39)	**1.34E-02**	**4.68E-02**	opposite
Cer(m18:0/24:1)	-1.46	(-2.42--0.37)	**9.81E-03**	**2.02E-02**	4.35	(2.12-6.63)	**1.28E-04**	**1.20E-03**	opposite
LPC(14:0) [sn2]	1.75	(1.25-2.26)	**6.35E-13**	**4.36E-12**	4.86	(2.45-7.33)	**7.65E-05**	**8.95E-04**	same
LPC(14:0) [sn1]	1.62	(1.14-2.11)	**5.17E-12**	**3.06E-11**	4.70	(2.35-7.09)	**8.28E-05**	**9.46E-04**	same
LPC(16:1) [sn2]	4.00	(3.27-4.78)	**3.37E-31**	**8.10E-29**	5.09	(3.19-7.03)	**1.66E-07**	**1.14E-05**	same
LPC(16:1) [sn1]	3.89	(3.2-4.61)	**6.09E-33**	**2.92E-30**	3.93	(2.1-5.79)	**2.67E-05**	**4.62E-04**	same
LPC(20:1) [sn1]	1.75	(1.27-2.25)	**1.55E-13**	**1.12E-12**	-2.32	(-3.93--0.7)	**5.46E-03**	**2.19E-02**	opposite
LPC(20:3) [sn2]	2.58	(1.91-3.3)	**1.79E-15**	**1.72E-14**	2.63	(0.79-4.5)	**4.98E-03**	**2.10E-02**	same
LPC(20:3) [104_sn1]	3.22	(2.49-3.99)	**9.49E-21**	**2.85E-19**	2.53	(0.51-4.6)	**1.42E-02**	**4.91E-02**	same
PC(32:1)	0.94	(0.11-1.83)	**2.49E-02**	**4.70E-02**	3.56	(1.41-5.75)	**1.12E-03**	**6.20E-03**	same
PC(34:5)	-1.14	(-1.93--0.28)	**1.10E-02**	**2.23E-02**	7.01	(3.69-10.43)	**3.00E-05**	**4.64E-04**	opposite
PC(17:0_18:2)	-1.28	(-1.84--0.68)	**5.78E-05**	**1.69E-04**	-2.03	(-3.62--0.41)	**1.44E-02**	**4.92E-02**	same
PC(16:0_20:5)	-1.86	(-2.69--0.94)	**1.83E-04**	**4.92E-04**	3.86	(0.8-7.01)	**1.32E-02**	**4.65E-02**	opposite
PC(36:6)	-0.83	(-1.42--0.2)	**1.07E-02**	**2.19E-02**	4.41	(1.91-6.97)	**5.17E-04**	**3.45E-03**	opposite
PC(18:0_22:6)	-1.44	(-2.09--0.74)	**1.21E-04**	**3.40E-04**	3.01	(0.9-5.18)	**5.22E-03**	**2.12E-02**	opposite
PC(O-18:0/18:1)	1.31	(0.62-2.05)	**1.35E-04**	**3.71E-04**	-3.04	(-4.22--1.85)	**1.05E-06**	**3.88E-05**	opposite
PC(O-18:1/18:1)	0.71	(0.11-1.35)	**2.01E-02**	**3.86E-02**	-1.86	(-2.95--0.75)	**1.12E-03**	**6.20E-03**	opposite
PC(P-35:2) (b)	-1.45	(-2.06--0.79)	**3.89E-05**	**1.15E-04**	-2.72	(-4.69--0.7)	**8.59E-03**	**3.22E-02**	same
PE(18:0_22:6)	-1.61	(-2.34--0.8)	**2.02E-04**	**5.43E-04**	3.81	(1.08-6.61)	**6.06E-03**	**2.36E-02**	opposite
PI(18:1_18:2)	-1.47	(-1.98--0.93)	**4.93E-07**	**1.78E-06**	-3.22	(-4.99--1.41)	**5.62E-04**	**3.65E-03**	same

Table S3D. The overlapping lipid species between the 6-year-old child BMI and adult (postnatal mother) BMI studies. Related to Figure 3.

	Child BMI (6-year-old)				Adult BMI (postnatal)				
Lipid Species	%change in lipid concentration per unit BMI	95%CI	p-value	p-value(BH)	%change in lipid concentration per unit BMI	95%CI	p-value	p-value(BH)	Trend
AC(18:0)	-1.98	(-3.33--0.62)	**4.60E-03**	**1.97E-02**	-0.68	(-1.13--0.23)	**3.16E-03**	**6.00E-03**	same
CE(16:1)	5.77	(4.04-7.53)	**9.25E-11**	**2.22E-08**	2.17	(1.53-2.82)	**6.89E-11**	**4.60E-10**	same
CE(18:3)	2.78	(0.7-4.91)	**8.76E-03**	**3.24E-02**	0.72	(0.11-1.32)	**2.04E-02**	**3.21E-02**	same
DG(16:0_16:1)	6.21	(3.58-8.89)	**3.25E-06**	**8.67E-05**	4.43	(3.46-5.4)	**9.58E-19**	**4.12E-17**	same
DG(14:0_18:2)	6.70	(3.02-10.51)	**3.23E-04**	**2.28E-03**	4.19	(2.86-5.54)	**7.61E-10**	**4.11E-09**	same
DG(16:0_18:1)	3.90	(1.38-6.49)	**2.40E-03**	**1.18E-02**	4.26	(3.33-5.21)	**1.32E-18**	**4.86E-17**	same
DG(16:1_18:1)	6.17	(3.26-9.17)	**2.92E-05**	**4.64E-04**	4.04	(3.07-5.03)	**1.05E-15**	**1.80E-14**	same
DG(16:0_18:2)	4.00	(1.57-6.48)	**1.20E-03**	**6.49E-03**	3.62	(2.72-4.52)	**5.53E-15**	**7.59E-14**	same
DG(18:0_18:1)	4.06	(1.71-6.46)	**6.87E-04**	**4.23E-03**	4.41	(3.51-5.31)	**4.70E-21**	**4.51E-19**	same
DG(18:0_18:2)	3.76	(1.3-6.29)	**2.68E-03**	**1.27E-02**	4.40	(3.41-5.39)	**7.51E-18**	**2.57E-16**	same
DG(16:0_20:4)	4.97	(2.34-7.66)	**1.98E-04**	**1.61E-03**	4.27	(3.25-5.3)	**4.79E-16**	**9.59E-15**	same
DG(18:1_20:4)	3.90	(1.68-6.16)	**5.47E-04**	**3.60E-03**	2.47	(1.71-3.22)	**1.81E-10**	**1.10E-09**	same
DG(16:0_22:5)	4.60	(2.03-7.23)	**4.22E-04**	**2.89E-03**	2.42	(1.46-3.38)	**7.01E-07**	**2.39E-06**	same
DG(18:2_20:4)	4.23	(1.78-6.73)	**6.78E-04**	**4.23E-03**	2.42	(1.55-3.3)	**5.34E-08**	**2.23E-07**	same
DG(16:0_22:6)	5.50	(2.13-8.99)	**1.31E-03**	**6.84E-03**	4.15	(2.87-5.45)	**2.35E-10**	**1.41E-09**	same
DG(18:1_22:6)	4.14	(1.32-7.04)	**3.88E-03**	**1.73E-02**	3.18	(2.04-4.34)	**5.26E-08**	**2.22E-07**	same
DG(18:2_22:6)	4.79	(1.45-8.24)	**4.80E-03**	**2.04E-02**	3.05	(1.87-4.24)	**3.60E-07**	**1.28E-06**	same
TG(48:0) [NL-18:0]	7.45	(3.74-11.29)	**7.09E-05**	**8.54E-04**	4.34	(2.99-5.71)	**3.54E-10**	**2.07E-09**	same
TG(48:1) [NL-16:1]	9.58	(5.88-13.41)	**2.99E-07**	**1.32E-05**	6.64	(5.32-7.97)	**1.51E-22**	**3.62E-20**	same
TG(48:1) [NL-18:1]	8.20	(3.97-12.59)	**1.20E-04**	**1.17E-03**	5.21	(3.76-6.69)	**2.44E-12**	**2.13E-11**	same
TG(48:2) [NL-14:0]	8.73	(4.41-13.22)	**6.10E-05**	**7.91E-04**	5.21	(3.73-6.72)	**5.81E-12**	**4.73E-11**	same
TG(48:2) [NL-14:1]	8.90	(4.81-13.16)	**1.61E-05**	**3.36E-04**	4.79	(3.42-6.18)	**7.51E-12**	**5.72E-11**	same
TG(48:2) [NL-16:1]	10.74	(6.57-15.07)	**3.03E-07**	**1.32E-05**	5.71	(4.32-7.12)	**1.20E-15**	**1.99E-14**	same
TG(48:2) [NL-18:2]	8.58	(4.3-13.04)	**7.11E-05**	**8.54E-04**	5.21	(3.68-6.77)	**2.87E-11**	**2.03E-10**	same
TG(48:3) [NL-14:0]	9.53	(5.17-14.08)	**1.43E-05**	**3.28E-04**	4.32	(2.9-5.76)	**2.31E-09**	**1.14E-08**	same
TG(48:3) [NL-16:1]	8.59	(4.99-12.31)	**2.28E-06**	**6.49E-05**	4.00	(2.79-5.24)	**1.30E-10**	**8.10E-10**	same
TG(48:3) [NL-18:3]	8.18	(3.69-12.86)	**3.09E-04**	**2.27E-03**	4.99	(3.41-6.59)	**5.23E-10**	**2.92E-09**	same
TG(49:1) [NL-17:1]	4.94	(2.47-7.47)	**8.54E-05**	**9.54E-04**	4.04	(3.11-4.97)	**2.94E-17**	**8.82E-16**	same
TG(50:1) [NL-14:0]	6.89	(3.47-10.42)	**7.00E-05**	**8.54E-04**	4.78	(3.52-6.05)	**1.11E-13**	**1.24E-12**	same
TG(50:1) [NL-16:0]	4.63	(2.1-7.22)	**3.13E-04**	**2.27E-03**	4.34	(3.39-5.3)	**1.03E-18**	**4.12E-17**	same
TG(50:1) [NL-18:1]	4.17	(1.73-6.68)	**7.99E-04**	**4.74E-03**	4.10	(3.16-5.05)	**4.06E-17**	**1.08E-15**	same
TG(50:2) [NL-14:0]	4.36	(1.14-7.69)	**7.83E-03**	**3.01E-02**	2.80	(1.76-3.86)	**1.50E-07**	**5.54E-07**	same
TG(50:2) [NL-16:1]	6.45	(3.42-9.58)	**2.79E-05**	**4.62E-04**	4.04	(3.07-5.02)	**6.67E-16**	**1.28E-14**	same
TG(50:2) [NL-18:1]	6.24	(3.2-9.38)	**5.20E-05**	**7.33E-04**	3.59	(2.65-4.54)	**1.19E-13**	**1.30E-12**	same
TG(50:2) [NL-18:2]	4.53	(2.11-7)	**2.30E-04**	**1.78E-03**	3.72	(2.82-4.62)	**8.45E-16**	**1.53E-14**	same
TG(50:3) [NL-14:0]	5.47	(1.88-9.18)	**2.69E-03**	**1.27E-02**	3.13	(1.93-4.34)	**2.88E-07**	**1.04E-06**	same
TG(50:3) [NL-14:1]	4.17	(1.33-7.09)	**3.92E-03**	**1.73E-02**	2.66	(1.69-3.63)	**7.85E-08**	**3.14E-07**	same
TG(50:3) [NL-16:1]	8.28	(4.83-11.85)	**2.15E-06**	**6.49E-05**	4.38	(3.28-5.5)	**1.32E-14**	**1.67E-13**	same
TG(50:3) [NL-18:2]	7.14	(3.78-10.61)	**2.72E-05**	**4.62E-04**	3.72	(2.65-4.81)	**1.38E-11**	**1.00E-10**	same
TG(50:3) [NL-18:3]	6.76	(3.2-10.44)	**1.78E-04**	**1.52E-03**	5.18	(3.92-6.44)	**8.59E-16**	**1.53E-14**	same
TG(50:4) [NL-14:0]	5.22	(1.61-8.96)	**4.43E-03**	**1.92E-02**	2.75	(1.47-4.04)	**2.32E-05**	**6.36E-05**	same
TG(50:4) [NL-18:3]	7.93	(4.25-11.74)	**2.01E-05**	**4.02E-04**	4.14	(2.91-5.38)	**5.20E-11**	**3.51E-10**	same
TG(50:4) [NL-20:4]	10.00	(5.69-14.48)	**3.96E-06**	**1.00E-04**	5.64	(4.02-7.29)	**8.80E-12**	**6.60E-11**	same
TG(51:1) [NL-17:0]	4.90	(2-7.89)	**8.93E-04**	**5.23E-03**	4.54	(3.5-5.59)	**2.82E-17**	**8.82E-16**	same
TG(51:2) [NL-17:0]	5.48	(2.52-8.52)	**2.67E-04**	**2.03E-03**	4.07	(3.06-5.09)	**6.13E-15**	**8.18E-14**	same
TG(51:2) [NL-17:1]	4.90	(2.22-7.65)	**3.17E-04**	**2.27E-03**	3.80	(2.86-4.74)	**3.86E-15**	**5.98E-14**	same
TG(52:1) [NL-18:0]	5.79	(2.8-8.86)	**1.32E-04**	**1.22E-03**	5.69	(4.53-6.86)	**2.15E-21**	**2.58E-19**	same
TG(52:1) [NL-18:1]	5.71	(2.83-8.68)	**9.59E-05**	**1.00E-03**	5.86	(4.73-7)	**1.79E-23**	**8.60E-21**	same
TG(52:2) [NL-18:2]	4.45	(2.1-6.85)	**1.98E-04**	**1.61E-03**	4.17	(3.27-5.07)	**4.36E-19**	**2.33E-17**	same
TG(52:3) [NL-16:1]	3.39	(0.97-5.88)	**5.95E-03**	**2.36E-02**	1.94	(1.21-2.66)	**1.73E-07**	**6.33E-07**	same
TG(52:4) [NL-16:1]	4.16	(1.08-7.34)	**7.91E-03**	**3.01E-02**	2.22	(1.27-3.18)	**4.67E-06**	**1.43E-05**	same
TG(52:4) [NL-18:3]	4.40	(1.43-7.46)	**3.60E-03**	**1.63E-02**	3.19	(2.17-4.21)	**8.03E-10**	**4.24E-09**	same
TG(52:5) [NL-18:3]	5.19	(2-8.47)	**1.36E-03**	**6.99E-03**	2.89	(1.78-4.01)	**3.35E-07**	**1.20E-06**	same
TG(52:5) [NL-20:4]	10.03	(6.39-13.8)	**4.75E-08**	**4.56E-06**	5.46	(4.2-6.74)	**3.97E-17**	**1.08E-15**	same

TG(52:5) [NL-20:5]	7.78	(4.53-11.13)	**2.30E-06**	**6.49E-05**	5.06	(3.69-6.44)	**4.80E-13**	**4.70E-12**	same
TG(54:1) [NL-18:1]	3.54	(1.13-6.02)	**3.96E-03**	**1.73E-02**	4.45	(3.57-5.34)	**4.54E-22**	**7.26E-20**	same
TG(54:2) [NL-18:0]	3.30	(0.94-5.71)	**6.02E-03**	**2.36E-02**	4.24	(3.35-5.13)	**2.72E-20**	**1.63E-18**	same
TG(54:3) [NL-18:2]	2.32	(0.48-4.19)	**1.32E-02**	**4.65E-02**	2.23	(1.57-2.89)	**5.13E-11**	**3.51E-10**	same
TG(54:4) [NL-20:3]	4.94	(2.41-7.54)	**1.24E-04**	**1.19E-03**	3.70	(2.83-4.58)	**2.18E-16**	**4.75E-15**	same
TG(54:5) [NL-20:4]	5.84	(3.43-8.3)	**1.90E-06**	**6.49E-05**	3.37	(2.55-4.2)	**2.12E-15**	**3.39E-14**	same
TG(54:6) [NL-20:4]	7.52	(4.68-10.44)	**1.91E-07**	**1.15E-05**	3.69	(2.71-4.68)	**2.17E-13**	**2.27E-12**	same
TG(54:6) [NL-20:5]	8.39	(5.04-11.85)	**7.54E-07**	**3.02E-05**	3.82	(2.52-5.13)	**7.53E-09**	**3.51E-08**	same
TG(54:6) [NL-22:6]	8.20	(4.48-12.06)	**1.29E-05**	**3.10E-04**	5.76	(4.33-7.22)	**4.96E-15**	**7.00E-14**	same
TG(54:7) [NL-20:5]	8.46	(5.22-11.8)	**2.49E-07**	**1.32E-05**	3.45	(2.17-4.75)	**1.37E-07**	**5.08E-07**	same
TG(54:7) [NL-22:6]	10.42	(6.51-14.47)	**1.23E-07**	**9.83E-06**	5.09	(3.68-6.52)	**1.79E-12**	**1.62E-11**	same
TG(56:6) [NL-20:4]	3.72	(1.91-5.57)	**5.78E-05**	**7.86E-04**	1.36	(0.85-1.88)	**2.38E-07**	**8.65E-07**	same
TG(56:6) [NL-22:5]	3.96	(1.82-6.14)	**2.87E-04**	**2.15E-03**	1.99	(1.2-2.77)	**7.33E-07**	**2.46E-06**	same
TG(56:7) [NL-20:4]	4.16	(1.73-6.64)	**7.60E-04**	**4.56E-03**	1.88	(1.14-2.62)	**6.63E-07**	**2.29E-06**	same
TG(56:7) [NL-20:5]	5.58	(2.64-8.6)	**1.82E-04**	**1.54E-03**	2.47	(1.35-3.61)	**1.56E-05**	**4.46E-05**	same
TG(56:7) [NL-22:5]	5.15	(2.46-7.91)	**1.64E-04**	**1.46E-03**	2.55	(1.61-3.5)	**1.14E-07**	**4.45E-07**	same
TG(56:7) [NL-22:6]	6.45	(3.01-10)	**2.16E-04**	**1.70E-03**	3.96	(2.65-5.28)	**3.33E-09**	**1.61E-08**	same
TG(56:8) [NL-20:4]	5.12	(2.54-7.75)	**9.23E-05**	**1.00E-03**	1.88	(1-2.77)	**2.85E-05**	**7.72E-05**	same
TG(56:8) [NL-20:5]	5.03	(2.01-8.13)	**1.03E-03**	**5.94E-03**	1.59	(0.43-2.77)	**7.20E-03**	**1.26E-02**	same
TG(56:8) [NL-22:6]	7.57	(4.02-11.24)	**2.51E-05**	**4.62E-04**	3.97	(2.64-5.31)	**4.96E-09**	**2.38E-08**	same
TG(56:9) [NL-22:6]	6.66	(3.29-10.14)	**9.49E-05**	**1.00E-03**	3.07	(1.89-4.27)	**3.82E-07**	**1.35E-06**	same
TG(58:10) [NL-22:6]	5.17	(1.89-8.56)	**1.90E-03**	**9.52E-03**	2.08	(0.92-3.25)	**4.34E-04**	**9.92E-04**	same
TG(58:8) [NL-22:6]	4.73	(1.39-8.18)	**5.34E-03**	**2.16E-02**	2.80	(1.58-4.04)	**7.21E-06**	**2.15E-05**	same
TG(58:9) [NL-22:6]	4.51	(1.17-7.97)	**7.98E-03**	**3.02E-02**	2.17	(0.97-3.38)	**3.75E-04**	**8.69E-04**	same
TG(O-50:1) [NL-17:1]	4.99	(2.47-7.57)	**1.01E-04**	**1.03E-03**	4.08	(3.14-5.03)	**4.97E-17**	**1.26E-15**	same
TG(O-52:2) [NL-17:1]	4.89	(2.19-7.66)	**3.63E-04**	**2.52E-03**	3.82	(2.87-4.77)	**4.43E-15**	**6.45E-14**	same
TG(O-52:2) [NL-18:1]	5.04	(2-8.16)	**1.08E-03**	**6.15E-03**	2.26	(1.27-3.27)	**8.29E-06**	**2.44E-05**	same
Cer(m18:0/22:0)	2.43	(0.5-4.39)	**1.34E-02**	**4.68E-02**	2.95	(2.15-3.76)	**8.19E-13**	**7.86E-12**	same
Cer(m18:0/24:1)	4.35	(2.12-6.63)	**1.28E-04**	**1.20E-03**	2.84	(2-3.68)	**3.94E-11**	**2.74E-10**	same
GM3(d18:1/24:0)	-2.41	(-3.83--0.96)	**1.23E-03**	**6.55E-03**	-1.92	(-2.46--1.39)	**6.41E-12**	**5.13E-11**	same
HexCer(d18:1/20:0)	-2.39	(-4.02--0.73)	**5.05E-03**	**2.10E-02**	-1.29	(-1.81--0.76)	**2.18E-06**	**6.88E-06**	same
HexCer(d18:1/22:0)	-2.35	(-3.8--0.88)	**1.85E-03**	**9.34E-03**	-0.92	(-1.41--0.43)	**2.61E-04**	**6.17E-04**	same
HexCer(d18:1/24:0)	-1.92	(-3.37--0.46)	**1.03E-02**	**3.73E-02**	-1.34	(-1.83--0.85)	**1.19E-07**	**4.57E-07**	same
Hex2Cer(d18:1/22:0)	-2.48	(-4.09--0.83)	**3.43E-03**	**1.57E-02**	-1.43	(-2--0.87)	**8.84E-07**	**2.91E-06**	same
Hex2Cer(d18:1/24:1)	-2.62	(-4.35--0.85)	**3.91E-03**	**1.73E-02**	-1.91	(-2.5--1.32)	**5.82E-10**	**3.21E-09**	same
Hex3Cer(d18:1/24:0)	-2.28	(-3.7--0.83)	**2.21E-03**	**1.09E-02**	-1.90	(-2.42--1.37)	**6.58E-12**	**5.18E-11**	same
SM(d18:1/14:0)/SM(d16:1/16:0)	2.11	(0.57-3.68)	**7.37E-03**	**2.85E-02**	0.72	(0.27-1.18)	**1.93E-03**	**3.86E-03**	same
SM(d18:2/14:0)	6.29	(4.83-7.77)	**2.07E-16**	**9.93E-14**	1.49	(1.01-1.98)	**2.12E-09**	**1.07E-08**	same
SM(34:3)	2.92	(1.38-4.49)	**2.12E-04**	**1.70E-03**	0.97	(0.49-1.46)	**8.92E-05**	**2.27E-04**	same
SM(38:3) (a)	1.93	(0.68-3.2)	**2.56E-03**	**1.24E-02**	0.56	(0.14-0.98)	**8.55E-03**	**1.47E-02**	same
SM(d16:1/24:1)	2.62	(1.12-4.15)	**6.11E-04**	**3.91E-03**	0.55	(0.05-1.05)	**3.15E-02**	**4.75E-02**	same
LPC(14:0) [sn2]	4.86	(2.45-7.33)	**7.65E-05**	**8.95E-04**	1.18	(0.41-1.96)	**2.81E-03**	**5.40E-03**	same
LPC(14:0) [sn1]	4.70	(2.35-7.09)	**8.28E-05**	**9.46E-04**	1.53	(0.78-2.3)	**7.13E-05**	**1.84E-04**	same
LPC(16:1) [sn2]	5.09	(3.19-7.03)	**1.66E-07**	**1.14E-05**	0.95	(0.37-1.53)	**1.29E-03**	**2.69E-03**	same
LPC(16:1) [sn1]	3.93	(2.1-5.79)	**2.67E-05**	**4.62E-04**	0.87	(0.34-1.4)	**1.33E-03**	**2.76E-03**	same
LPC(19:0) [sn1] (a) / LPC(19:0) [sn2] (b)	-2.26	(-3.96--0.53)	**1.08E-02**	**3.85E-02**	-2.28	(-2.79--1.76)	**6.69E-17**	**1.61E-15**	same
LPC(20:0) [sn1]	-3.35	(-4.85--1.82)	**2.48E-05**	**4.62E-04**	-2.10	(-2.58--1.62)	**1.05E-16**	**2.41E-15**	same
LPC(20:1) [sn1]	-2.32	(-3.93--0.7)	**5.46E-03**	**2.19E-02**	-1.86	(-2.38--1.33)	**1.33E-11**	**9.82E-11**	same
LPC(20:3) [sn2]	2.63	(0.79-4.5)	**4.98E-03**	**2.10E-02**	1.19	(0.56-1.83)	**2.39E-04**	**5.67E-04**	same
LPC(20:3) [104_sn1]	2.53	(0.51-4.6)	**1.42E-02**	**4.91E-02**	0.93	(0.28-1.58)	**4.92E-03**	**8.95E-03**	same
LPC(O-20:0)	-2.32	(-4.14--0.47)	**1.46E-02**	**4.96E-02**	-2.23	(-2.77--1.69)	**4.19E-15**	**6.29E-14**	same
LPC(O-22:0)	-1.92	(-3.1--0.72)	**1.79E-03**	**9.15E-03**	-1.43	(-1.85--1)	**1.03E-10**	**6.79E-10**	same
PC(32:1)	3.56	(1.41-5.75)	**1.12E-03**	**6.20E-03**	2.58	(1.77-3.39)	**4.13E-10**	**2.39E-09**	same
PC(14:0_20:4)	4.75	(2.3-7.26)	**1.38E-04**	**1.25E-03**	1.14	(0.28-2.01)	**9.67E-03**	**1.64E-02**	same
PC(34:5)	7.01	(3.69-10.43)	**3.00E-05**	**4.64E-04**	1.64	(0.4-2.9)	**9.61E-03**	**1.64E-02**	same
PC(17:0_18:2)	-2.03	(-3.62--0.41)	**1.44E-02**	**4.92E-02**	-1.17	(-1.68--0.65)	**1.30E-05**	**3.74E-05**	same
PC(16:0_20:3) (a)	-2.86	(-4.42--1.29)	**4.47E-04**	**3.02E-03**	-1.61	(-2.18--1.04)	**5.63E-08**	**2.33E-07**	same
PC(18:2_18:2)	-3.04	(-5.24--0.78)	**8.73E-03**	**3.24E-02**	-2.49	(-3.22--1.75)	**1.24E-10**	**7.80E-10**	same
PC(18:0_20:3)	5.35	(3.52-7.21)	**1.22E-08**	**1.46E-06**	3.69	(2.93-4.46)	**1.54E-20**	**1.06E-18**	same

PC(18:0_22:6)	3.01	(0.9-5.18)	**5.22E-03**	**2.12E-02**	1.19	(0.44-1.95)	**1.98E-03**	**3.94E-03**	same
PC(O-34:1)	-2.24	(-3.3--1.18)	**4.84E-05**	**7.04E-04**	-1.21	(-1.62--0.79)	**2.44E-08**	**1.08E-07**	same
PC(O-36:0)	-2.41	(-3.76--1.03)	**6.74E-04**	**4.23E-03**	-0.84	(-1.33--0.35)	**9.07E-04**	**1.95E-03**	same
PC(O-18:0/18:1)	-3.04	(-4.22--1.85)	**1.05E-06**	**3.88E-05**	-1.48	(-1.92--1.04)	**1.07E-10**	**6.87E-10**	same
PC(O-18:1/18:1)	-1.86	(-2.95--0.75)	**1.12E-03**	**6.20E-03**	-1.71	(-2.13--1.29)	**1.10E-14**	**1.42E-13**	same
PC(O-18:0/18:2)	-2.63	(-4.12--1.12)	**7.48E-04**	**4.54E-03**	-1.89	(-2.38--1.39)	**4.43E-13**	**4.43E-12**	same
PC(P-16:0/18:0)	-2.11	(-3.09--1.13)	**3.73E-05**	**5.60E-04**	-1.47	(-1.84--1.09)	**1.05E-13**	**1.20E-12**	same
PC(P-16:0/18:1)	-1.99	(-3.17--0.79)	**1.27E-03**	**6.69E-03**	-2.19	(-2.7--1.69)	**2.94E-16**	**6.14E-15**	same
PC(P-35:2) (b)	-2.72	(-4.69--0.7)	**8.59E-03**	**3.22E-02**	-2.14	(-2.83--1.45)	**2.27E-09**	**1.13E-08**	same
PC(P-18:0/18:2)	-2.97	(-4.48--1.45)	**1.69E-04**	**1.48E-03**	-2.58	(-3.1--2.06)	**1.54E-20**	**1.06E-18**	same
PE(16:0_16:1)	4.35	(0.88-7.95)	**1.38E-02**	**4.78E-02**	3.03	(1.74-4.33)	**3.84E-06**	**1.18E-05**	same
PE(18:0_20:3) (a)	3.60	(1.22-6.04)	**3.02E-03**	**1.41E-02**	2.90	(1.96-3.84)	**1.44E-09**	**7.41E-09**	same
PE(18:0_22:6)	3.81	(1.08-6.61)	**6.06E-03**	**2.36E-02**	1.67	(0.73-2.63)	**5.37E-04**	**1.20E-03**	same
PE(P-16:0/20:3)	4.14	(2.26-6.06)	**1.60E-05**	**3.36E-04**	1.10	(0.38-1.82)	**2.78E-03**	**5.35E-03**	same
PE(P-20:0/18:2)	-2.72	(-4.31--1.11)	**1.09E-03**	**6.15E-03**	-1.98	(-2.5--1.45)	**1.04E-12**	**9.79E-12**	same
PI(16:0_16:1)	8.42	(5.56-11.34)	**6.42E-09**	**1.03E-06**	3.85	(2.77-4.94)	**3.76E-12**	**3.11E-11**	same
PI(18:1_18:2)	-3.22	(-4.99--1.41)	**5.62E-04**	**3.65E-03**	-1.29	(-1.97--0.61)	**2.16E-04**	**5.17E-04**	same

Table S3E. The overlapping lipid species in the four lipid-adiposity association studies. Related to Figure 3.

	ppBMI (antenatal)				Adult BMI (postnatal)				Birth Weight (cord blood)				Child BMI (6-year-old)				
Lipid Species	%change in lipid concentration per unit BMI	95%CI	p-value	p-value(BH)	%change in lipid concentration per unit BMI	95%CI	p-value	p-value(BH)	%change in lipid concentration per 100 grams	95%CI	p-value	p-value(BH)	%change in lipid concentration per unit BMI	95%CI	p-value	p-value(BH)	Trend
CE(16:1)	-1.06	(-1.82--0.29)	**7.23E-03**	**1.46E-02**	2.17	(1.53-2.82)	**6.89E-11**	**4.60E-10**	0.98	(0.31-1.69)	**3.55E-03**	**8.00E-03**	5.77	(4.04-7.53)	**9.25E-11**	**2.22E-08**	different
DG(16:0_18:1)	1.33	(0.59-2.07)	**4.36E-04**	**1.22E-03**	4.26	(3.33-5.21)	**1.32E-18**	**4.86E-17**	-1.91	(-2.57--1.19)	**1.35E-06**	**4.62E-06**	3.90	(1.38-6.49)	**2.40E-03**	**1.18E-02**	different
DG(16:0_18:2)	0.92	(0.21-1.65)	**1.15E-02**	**2.16E-02**	3.62	(2.72-4.52)	**5.53E-15**	**7.59E-14**	-3.07	(-3.76--2.31)	**1.09E-11**	**6.22E-11**	4.00	(1.57-6.48)	**1.20E-03**	**6.49E-03**	different
DG(18:0_18:1)	1.36	(0.69-2.04)	**7.91E-05**	**2.60E-04**	4.41	(3.51-5.31)	**4.70E-21**	**4.51E-19**	-1.29	(-1.99--0.53)	**1.23E-03**	**2.94E-03**	4.06	(1.71-6.46)	**6.87E-04**	**4.23E-03**	different
DG(18:0_18:2)	0.99	(0.23-1.76)	**1.04E-02**	**1.97E-02**	4.40	(3.41-5.39)	**7.51E-18**	**2.57E-16**	-1.62	(-2.52--0.61)	**2.43E-03**	**5.62E-03**	3.76	(1.3-6.29)	**2.68E-03**	**1.27E-02**	different
DG(16:0_20:4)	1.19	(0.26-2.12)	**1.22E-02**	**2.27E-02**	4.27	(3.25-5.3)	**4.79E-16**	**9.59E-15**	-2.06	(-2.83--1.21)	**9.20E-06**	**2.95E-05**	4.97	(2.34-7.66)	**1.98E-04**	**1.61E-03**	different
DG(18:1_20:4)	1.14	(0.4-1.88)	**2.42E-03**	**5.53E-03**	2.47	(1.71-3.22)	**1.81E-10**	**1.10E-09**	-2.06	(-2.71--1.36)	**1.08E-07**	**4.14E-07**	3.90	(1.68-6.16)	**5.47E-04**	**3.60E-03**	different
DG(18:1_22:6)	1.53	(0.47-2.61)	**4.63E-03**	**9.97E-03**	3.18	(2.04-4.34)	**5.26E-08**	**2.22E-07**	-3.49	(-4.15--2.76)	**7.44E-15**	**6.49E-14**	4.14	(1.32-7.04)	**3.88E-03**	**1.73E-02**	different
DG(18:2_22:6)	1.52	(0.42-2.63)	**6.55E-03**	**1.35E-02**	3.05	(1.87-4.24)	**3.60E-07**	**1.28E-06**	-4.00	(-4.74--3.16)	**9.00E-14**	**6.75E-13**	4.79	(1.45-8.24)	**4.80E-03**	**2.04E-02**	different
TG(48:0) [NL-18:0]	-1.56	(-2.43--0.68)	**5.76E-04**	**1.53E-03**	4.34	(2.99-5.71)	**3.54E-10**	**2.07E-09**	-1.44	(-2.28--0.51)	**3.14E-03**	**7.14E-03**	7.45	(3.74-11.29)	**7.09E-05**	**8.54E-04**	different
TG(48:2) [NL-14:1]	-1.20	(-2.25--0.13)	**2.78E-02**	**4.70E-02**	4.79	(3.42-6.18)	**7.51E-12**	**5.72E-11**	-3.06	(-3.81--2.23)	**4.50E-10**	**2.23E-09**	8.90	(4.81-13.16)	**1.61E-05**	**3.36E-04**	different
TG(48:2) [NL-18:2]	-1.16	(-2.2--0.12)	**2.91E-02**	**4.87E-02**	5.21	(3.68-6.77)	**2.87E-11**	**2.03E-10**	-4.14	(-4.86--3.32)	**5.42E-15**	**4.91E-14**	8.58	(4.3-13.04)	**7.11E-05**	**8.54E-04**	different
TG(48:3) [NL-14:0]	-1.51	(-2.64--0.37)	**9.55E-03**	**1.85E-02**	4.32	(2.9-5.76)	**2.31E-09**	**1.14E-08**	-4.04	(-4.73--3.26)	**1.02E-15**	**1.04E-14**	9.53	(5.17-14.08)	**1.43E-05**	**3.28E-04**	different
TG(48:3) [NL-18:3]	-1.81	(-3.07--0.53)	**5.76E-03**	**1.21E-02**	4.99	(3.41-6.59)	**5.23E-10**	**2.92E-09**	-3.23	(-3.91--2.48)	**8.75E-13**	**5.83E-12**	8.18	(3.69-12.86)	**3.09E-04**	**2.27E-03**	different
TG(50:1) [NL-16:0]	0.83	(0.22-1.43)	**7.27E-03**	**1.46E-02**	4.34	(3.39-5.3)	**1.03E-18**	**4.12E-17**	-2.49	(-3.25--1.64)	**2.17E-07**	**8.15E-07**	4.63	(2.1-7.22)	**3.13E-04**	**2.27E-03**	different
TG(50:1) [NL-18:1]	0.76	(0.18-1.34)	**1.03E-02**	**1.97E-02**	4.10	(3.16-5.05)	**4.06E-17**	**1.08E-15**	-2.47	(-3.24--1.62)	**2.61E-07**	**9.63E-07**	4.17	(1.73-6.68)	**7.99E-04**	**4.74E-03**	different
TG(50:2) [NL-14:0]	-0.65	(-1.21--0.07)	**2.69E-02**	**4.57E-02**	2.80	(1.76-3.86)	**1.50E-07**	**5.54E-07**	-3.34	(-4.1--2.48)	**1.16E-10**	**6.01E-10**	4.36	(1.14-7.69)	**7.83E-03**	**3.01E-02**	different
TG(50:2) [NL-18:2]	0.66	(0.08-1.24)	**2.58E-02**	**4.41E-02**	3.72	(2.82-4.62)	**8.45E-16**	**1.53E-14**	-4.17	(-4.92--3.31)	**5.26E-14**	**4.14E-13**	4.53	(2.11-7)	**2.30E-04**	**1.78E-03**	different
TG(52:1) [NL-18:0]	1.38	(0.59-2.18)	**6.58E-04**	**1.73E-03**	5.69	(4.53-6.86)	**2.15E-21**	**2.58E-19**	-1.86	(-2.76--0.83)	**6.81E-04**	**1.69E-03**	5.79	(2.8-8.86)	**1.32E-04**	**1.22E-03**	different
TG(52:1) [NL-18:1]	1.45	(0.69-2.23)	**2.04E-04**	**6.17E-04**	5.86	(4.73-7)	**1.79E-23**	**8.60E-21**	-1.80	(-2.71--0.77)	**9.96E-04**	**2.41E-03**	5.71	(2.83-8.68)	**9.59E-05**	**1.00E-03**	different
TG(54:1) [NL-18:1]	0.99	(0.32-1.66)	**3.57E-03**	**8.00E-03**	4.45	(3.57-5.34)	**4.54E-22**	**7.26E-20**	-1.64	(-2.56--0.59)	**2.89E-03**	**6.61E-03**	3.54	(1.13-6.02)	**3.96E-03**	**1.73E-02**	different
TG(54:2) [NL-18:0]	1.41	(0.72-2.1)	**6.55E-05**	**2.23E-04**	4.24	(3.35-5.13)	**2.72E-20**	**1.63E-18**	-1.85	(-2.69--0.91)	**2.40E-04**	**6.39E-04**	3.30	(0.94-5.71)	**6.02E-03**	**2.36E-02**	different
TG(54:3) [NL-18:2]	0.72	(0.14-1.3)	**1.43E-02**	**2.59E-02**	2.23	(1.57-2.89)	**5.13E-11**	**3.51E-10**	-3.06	(-3.75--2.3)	**1.28E-11**	**7.23E-11**	2.32	(0.48-4.19)	**1.32E-02**	**4.65E-02**	different
TG(54:5) [NL-20:4]	1.04	(0.38-1.71)	**1.91E-03**	**4.51E-03**	3.37	(2.55-4.2)	**2.12E-15**	**3.39E-14**	-3.73	(-4.49--2.86)	**4.40E-12**	**2.64E-11**	5.84	(3.43-8.3)	**1.90E-06**	**6.49E-05**	different
TG(54:6) [NL-20:4]	1.16	(0.33-2)	**6.24E-03**	**1.29E-02**	3.69	(2.71-4.68)	**2.17E-13**	**2.27E-12**	-4.11	(-4.83--3.28)	**1.19E-14**	**9.81E-14**	7.52	(4.68-10.44)	**1.91E-07**	**1.15E-05**	different
TG(54:6) [NL-22:6]	1.79	(0.57-3.02)	**4.10E-03**	**9.06E-03**	5.76	(4.33-7.22)	**4.96E-15**	**7.00E-14**	-5.16	(-5.83--4.38)	**1.98E-20**	**5.23E-19**	8.20	(4.48-12.06)	**1.29E-05**	**3.10E-04**	different
TG(56:6) [NL-20:4]	1.34	(0.81-1.87)	**7.12E-07**	**3.60E-06**	1.36	(0.85-1.88)	**2.38E-07**	**8.65E-07**	-3.00	(-3.77--2.15)	**1.89E-09**	**8.92E-09**	3.72	(1.91-5.57)	**5.78E-05**	**7.86E-04**	different
TG(56:7) [NL-20:4]	1.02	(0.35-1.7)	**2.84E-03**	**6.40E-03**	1.88	(1.14-2.62)	**6.63E-07**	**2.29E-06**	-3.66	(-4.41--2.81)	**3.38E-12**	**2.08E-11**	4.16	(1.73-6.64)	**7.60E-04**	**4.56E-03**	different
TG(56:7) [NL-22:6]	1.93	(0.84-3.04)	**4.97E-04**	**1.35E-03**	3.96	(2.65-5.28)	**3.33E-09**	**1.61E-08**	-5.07	(-5.72--4.33)	**1.85E-21**	**7.12E-20**	6.45	(3.01-10)	**2.16E-04**	**1.70E-03**	different
TG(56:8) [NL-22:6]	1.76	(0.54-2.99)	**4.49E-03**	**9.76E-03**	3.97	(2.64-5.31)	**4.96E-09**	**2.38E-08**	-5.36	(-5.97--4.65)	**1.69E-24**	**1.02E-22**	7.57	(4.02-11.24)	**2.51E-05**	**4.62E-04**	different
TG(58:8) [NL-22:6]	1.46	(0.38-2.56)	**7.98E-03**	**1.58E-02**	2.80	(1.58-4.04)	**7.21E-06**	**2.15E-05**	-4.50	(-5.16--3.76)	**3.67E-19**	**7.05E-18**	4.73	(1.39-8.18)	**5.34E-03**	**2.16E-02**	different
Cer(m18:0/24:1)	1.15	(0.26-2.04)	**1.11E-02**	**2.08E-02**	2.84	(2-3.68)	**3.94E-11**	**2.74E-10**	-1.46	(-2.42--0.37)	**9.81E-03**	**2.02E-02**	4.35	(2.12-6.63)	**1.28E-04**	**1.20E-03**	different
LPC(20:1) [sn1]	-0.62	(-1.08--0.15)	**9.49E-03**	**1.84E-02**	-1.86	(-2.38--1.33)	**1.33E-11**	**9.82E-11**	1.75	(1.27-2.25)	**1.55E-13**	**1.12E-12**	-2.32	(-3.93--0.7)	**5.46E-03**	**2.19E-02**	different
LPC(20:3) [sn2]	1.05	(0.4-1.7)	**1.48E-03**	**3.59E-03**	1.19	(0.56-1.83)	**2.39E-04**	**5.67E-04**	2.58	(1.91-3.3)	**1.79E-15**	**1.72E-14**	2.63	(0.79-4.5)	**4.98E-03**	**2.10E-02**	same
LPC(20:3) [104_sn1]	1.42	(0.72-2.12)	**7.46E-05**	**2.47E-04**	0.93	(0.28-1.58)	**4.92E-03**	**8.95E-03**	3.22	(2.49-3.99)	**9.49E-21**	**2.85E-19**	2.53	(0.51-4.6)	**1.42E-02**	**4.91E-02**	same
PC(17:0_18:2)	-1.54	(-2.08--1)	**3.86E-08**	**2.57E-07**	-1.17	(-1.68--0.65)	**1.30E-05**	**3.74E-05**	-1.28	(-1.84--0.68)	**5.78E-05**	**1.69E-04**	-2.03	(-3.62--0.41)	**1.44E-02**	**4.92E-02**	same
PC(O-18:0/18:1)	-1.51	(-1.98--1.03)	**1.00E-09**	**1.02E-08**	-1.48	(-1.92--1.04)	**1.07E-10**	**6.87E-10**	1.31	(0.62-2.05)	**1.35E-04**	**3.71E-04**	-3.04	(-4.22--1.85)	**1.05E-06**	**3.88E-05**	different
PC(O-18:1/18:1)	-1.20	(-1.66--0.74)	**4.16E-07**	**2.24E-06**	-1.71	(-2.13--1.29)	**1.10E-14**	**1.42E-13**	0.71	(0.11-1.35)	**2.01E-02**	**3.86E-02**	-1.86	(-2.95--0.75)	**1.12E-03**	**6.20E-03**	different
PC(P-35:2) (b)	-1.83	(-2.55--1.1)	**1.07E-06**	**5.20E-06**	-2.14	(-2.83--1.45)	**2.27E-09**	**1.13E-08**	-1.45	(-2.06--0.79)	**3.89E-05**	**1.15E-04**	-2.72	(-4.69--0.7)	**8.59E-03**	**3.22E-02**	same
PE(18:0_22:6)	1.34	(0.55-2.14)	**8.34E-04**	**2.13E-03**	1.67	(0.73-2.63)	**5.37E-04**	**1.20E-03**	-1.61	(-2.34--0.8)	**2.02E-04**	**5.43E-04**	3.81	(1.08-6.61)	**6.06E-03**	**2.36E-02**	different
PI(18:1_18:2)	-2.66	(-3.29--2.03)	**1.11E-15**	**5.91E-14**	-1.29	(-1.97--0.61)	**2.16E-04**	**5.17E-04**	-1.47	(-1.98--0.93)	**4.93E-07**	**1.78E-06**	-3.22	(-4.99--1.41)	**5.62E-04**	**3.65E-03**	same

Table S4A. Sex difference in association studies with birth weight and child BMI in terms of class levels.

	**Birth Weight (cord blood) - Male** adjusted for ethnicity, maternal age, maternal education level, pre-pregnancy BMI, total gestational weight gain, gestational age and parity				**Birth Weight (cord blood) -Female** adjusted for ethnicity, maternal age, maternal education level, pre-pregnancy BMI, total gestational weight gain, gestational age and parity				**Child BMI (6-year-old) - Male** adjusted for ethnicity and maternal education level				**Child BMI (6-year-old) -Female** adjusted for ethnicity and maternal education level			
Lipid Class	%change in lipid concentration per 100 grams	95%CI	p-value	p-value(BH)	%change in lipid concentration per 100 grams	95%CI	p-value	p-value(BH)	%change in lipid concentration per unit BMI	95%CI	p-value	p-value(BH)	%change in lipid concentration per unit BMI	95%CI	p-value	p-value(BH)
AC	-0.87	(-1.54--0.15)	**1.90E-02**	**3.96E-02**	-0.06	(-0.91- 0.87)	8.95E-01	9.66E-01	-0.13	(-2.08-1.86)	8.98E-01	9.35E-01	-1.79	(-4.19- 0.68)	1.53E-01	4.77E-01
CE	-0.44	(-0.99- 0.14)	1.36E-01	2.12E-01	-0.06	(-0.79- 0.74)	8.84E-01	9.66E-01	0.36	(-0.87-1.61)	5.63E-01	7.19E-01	0.71	(-0.70- 2.13)	3.22E-01	5.75E-01
DG	-2.70	(-3.37--1.97)	**3.67E-10**	**2.29E-09**	-1.42	(-2.40--0.32)	**1.34E-02**	**4.17E-02**	1.75	(-1.03-4.60)	2.18E-01	6.07E-01	4.47	(1.03- 8.02)	**1.08E-02**	9.02E-02
TG	-3.85	(-4.60--3.01)	**9.57E-13**	**1.20E-11**	-2.25	(-3.47--0.80)	**3.73E-03**	**1.33E-02**	3.11	(0.51-5.78)	**1.89E-02**	4.73E-01	4.46	(1.23- 7.80)	**6.78E-03**	8.48E-02
TG(O)	-3.00	(-3.77--2.14)	**4.15E-09**	**2.08E-08**	-2.25	(-3.29--1.04)	**6.13E-04**	**2.55E-03**	2.70	(-0.12-5.59)	6.05E-02	6.07E-01	3.59	(-0.10- 7.40)	5.63E-02	2.81E-01
dhCer	-0.17	(-1.00- 0.74)	7.03E-01	8.36E-01	-0.41	(-1.38- 0.67)	4.37E-01	8.87E-01	0.94	(-1.19-3.12)	3.88E-01	6.47E-01	0.67	(-1.69- 3.08)	5.80E-01	7.94E-01
Cer	-0.63	(-1.30- 0.08)	8.20E-02	1.46E-01	-0.01	(-0.90- 0.97)	9.81E-01	9.81E-01	0.60	(-1.14-2.36)	5.02E-01	6.97E-01	0.48	(-1.32- 2.33)	6.00E-01	7.94E-01
DeoxyCer	-2.13	(-2.82--1.37)	**5.38E-07**	**2.24E-06**	-1.97	(-2.84--0.99)	**2.03E-04**	**1.02E-03**	1.15	(-0.99-3.33)	2.95E-01	6.20E-01	2.73	(-0.07- 5.61)	5.59E-02	2.81E-01
GM3	-0.41	(-1.25- 0.50)	3.60E-01	4.91E-01	-0.17	(-1.25- 1.05)	7.78E-01	9.66E-01	-1.01	(-2.59-0.59)	2.14E-01	6.07E-01	-1.94	(-4.02- 0.18)	7.18E-02	2.96E-01
HexCer	0.33	(-0.63- 1.39)	5.16E-01	6.44E-01	0.06	(-1.03- 1.29)	9.15E-01	9.66E-01	-0.89	(-2.66-0.91)	3.29E-01	6.28E-01	-2.84	(-4.69--0.95)	**3.67E-03**	8.48E-02
Hex2Cer	-0.41	(-1.26- 0.52)	3.74E-01	4.91E-01	-0.41	(-1.46- 0.77)	4.82E-01	8.87E-01	-1.03	(-3.16-1.16)	3.52E-01	6.28E-01	-1.47	(-4.02- 1.15)	2.68E-01	5.15E-01
Hex3Cer	-0.39	(-1.12- 0.40)	3.27E-01	4.81E-01	-0.35	(-1.29- 0.70)	4.99E-01	8.87E-01	-1.00	(-2.45-0.47)	1.78E-01	6.07E-01	-1.19	(-2.97- 0.63)	1.98E-01	4.78E-01
SM	-0.09	(-0.77- 0.64)	8.03E-01	8.36E-01	-0.05	(-0.90- 0.88)	9.10E-01	9.66E-01	-0.57	(-2.14-1.03)	4.81E-01	6.97E-01	-0.38	(-2.32- 1.59)	6.99E-01	7.94E-01
LPC	2.24	(1.55- 2.97)	**2.84E-11**	**2.37E-10**	2.30	(1.49- 3.17)	**5.89E-09**	**7.37E-08**	-0.86	(-3.09-1.41)	4.52E-01	6.97E-01	1.77	(-0.96- 4.58)	2.05E-01	4.78E-01
LPC(O)	1.34	(0.75- 1.96)	**5.46E-06**	**1.71E-05**	1.33	(0.66- 2.05)	**7.05E-05**	**4.41E-04**	-1.44	(-3.53-0.70)	1.84E-01	6.07E-01	1.07	(-1.41- 3.61)	3.99E-01	6.66E-01
LPC(P)	1.51	(0.88- 2.17)	**1.27E-06**	**4.54E-06**	1.44	(0.73- 2.19)	**4.20E-05**	**3.50E-04**	-1.31	(-3.56-0.99)	2.60E-01	6.20E-01	1.59	(-1.04- 4.29)	2.36E-01	4.91E-01
LPE	2.54	(1.82- 3.30)	**3.66E-13**	**9.16E-12**	2.67	(1.78- 3.62)	**5.31E-10**	**1.33E-08**	-1.66	(-3.45-0.17)	7.42E-02	6.07E-01	-0.61	(-2.99- 1.82)	6.18E-01	7.94E-01
PC	-0.56	(-1.19- 0.12)	1.04E-01	1.74E-01	-0.42	(-1.24- 0.47)	3.44E-01	8.60E-01	-0.21	(-1.73-1.33)	7.82E-01	8.51E-01	-0.20	(-2.08- 1.73)	8.39E-01	9.03E-01
PC(O)	-0.10	(-0.84- 0.69)	7.92E-01	8.36E-01	-0.04	(-0.94- 0.94)	9.28E-01	9.66E-01	0.02	(-1.71-1.78)	9.82E-01	9.82E-01	-0.05	(-2.12- 2.06)	9.61E-01	9.61E-01
PC(P)	-1.00	(-1.68--0.27)	**8.01E-03**	**2.00E-02**	-0.71	(-1.57- 0.24)	1.35E-01	3.76E-01	-0.32	(-2.14-1.53)	7.30E-01	8.29E-01	-0.50	(-2.70- 1.75)	6.59E-01	7.94E-01
PE	-1.12	(-1.79--0.39)	**3.12E-03**	**8.68E-03**	-0.45	(-1.43- 0.63)	3.94E-01	8.87E-01	0.74	(-2.19-3.76)	6.24E-01	7.42E-01	-0.76	(-4.33- 2.94)	6.81E-01	7.94E-01
PE(O)	-0.08	(-0.84- 0.74)	8.36E-01	8.36E-01	0.06	(-0.88- 1.10)	9.06E-01	9.66E-01	0.68	(-1.70-3.13)	5.75E-01	7.19E-01	1.01	(-1.85- 3.96)	4.91E-01	7.67E-01
PE(P)	-0.11	(-0.83- 0.66)	7.63E-01	8.36E-01	0.20	(-0.78- 1.29)	7.03E-01	9.66E-01	1.42	(-0.67-3.54)	1.83E-01	6.07E-01	1.57	(-0.89- 4.09)	2.11E-01	4.78E-01
PG	-1.47	(-2.50--0.31)	**1.49E-02**	**3.39E-02**	-0.23	(-1.74- 1.56)	7.86E-01	9.66E-01	1.59	(-1.39-4.66)	2.98E-01	6.20E-01	3.25	(-0.42- 7.06)	8.29E-02	2.96E-01
PI	-0.58	(-1.07--0.06)	**2.80E-02**	5.38E-02	-0.23	(-0.90- 0.50)	5.32E-01	8.87E-01	-1.23	(-2.80-0.37)	1.30E-01	6.07E-01	-0.18	(-2.27- 1.95)	8.67E-01	9.03E-01

Table S4B. Sex difference in association studies with birth weight and child BMI in terms of species levels.

Studies		**Birth Weight (cord blood) - Male**				**Birth Weight (cord blood) - Female**				**Child BMI (6-year-old) - Male**				**Child BMI (6-year-old) - Female**			
Covariates		adjusted for ethnicity, maternal age, maternal education level, pre-pregnancy BMI, total gestational weight gain, gestational age and parity				adjusted for ethnicity, maternal age, maternal education level, pre-pregnancy BMI, total gestational weight gain, gestational age and parity				adjusted for ethnicity and maternal education level				adjusted for ethnicity and maternal education level			
No.	Lipid Species	%change in lipid concentration per 100 grams	95%CI	p-value	p-value(BH)	%change in lipid concentration per 100 grams	95%CI	p-value	p-value(BH)	%change in lipid concentration per unit BMI	95%CI	p-value	p-value(BH)	%change in lipid concentration per unit BMI	95%CI	p-value	p-value(BH)
1	AC(12:0)	-1.55	(-2.29--0.75)	**2.80E-04**	**8.56E-04**	-1.39	(-2.33--0.33)	**1.19E-02**	**4.10E-02**	-0.10	(-3.26- 3.15)	9.49E-01	9.83E-01	-2.70	(-6.60- 1.35)	1.87E-01	3.70E-01
2	AC(13:0)	-1.05	(-2.40- 0.54)	1.83E-01	2.90E-01	-1.14	(-2.80- 0.89)	2.49E-01	4.38E-01	3.09	(-1.44- 7.82)	1.83E-01	4.24E-01	-1.31	(-6.81- 4.51)	6.50E-01	8.08E-01
3	AC(14:0)	-1.18	(-1.87--0.42)	**3.07E-03**	**8.02E-03**	-0.61	(-1.54- 0.43)	2.43E-01	4.31E-01	0.19	(-2.16- 2.60)	8.75E-01	9.47E-01	-2.93	(-5.92- 0.14)	6.12E-02	1.88E-01
4	AC(14:1)	-0.99	(-1.84--0.05)	**3.96E-02**	7.73E-02	-1.10	(-2.13- 0.06)	6.17E-02	1.56E-01	-0.67	(-4.11- 2.88)	7.04E-01	8.83E-01	-3.22	(-7.33- 1.06)	1.37E-01	3.14E-01
5	AC(14:2)	-1.15	(-1.83--0.41)	**2.88E-03**	**7.61E-03**	-0.59	(-1.49- 0.41)	2.40E-01	4.30E-01	-0.20	(-3.28- 2.98)	9.02E-01	9.64E-01	-2.92	(-6.94- 1.28)	1.69E-01	3.54E-01
6	AC(16:0)	-0.83	(-1.49--0.11)	**2.41E-02**	5.01E-02	0.25	(-0.63- 1.20)	5.89E-01	7.63E-01	0.21	(-1.33- 1.76)	7.92E-01	9.16E-01	-1.11	(-3.00- 0.81)	2.53E-01	4.44E-01
7	AC(16:1)	-1.15	(-1.98--0.25)	**1.40E-02**	**3.08E-02**	-0.62	(-1.64- 0.54)	2.82E-01	4.75E-01	0.97	(-1.63- 3.64)	4.67E-01	7.19E-01	-1.48	(-4.69- 1.83)	3.73E-01	5.66E-01
8	AC(18:0)	-1.07	(-1.70--0.38)	**2.94E-03**	**7.72E-03**	-0.25	(-1.12- 0.69)	5.85E-01	7.61E-01	-1.51	(-3.37- 0.38)	1.16E-01	3.23E-01	-2.29	(-4.23--0.31)	**2.42E-02**	1.13E-01
9	AC(18:1)	-0.19	(-1.08- 0.78)	6.86E-01	7.66E-01	0.76	(-0.31- 1.94)	1.69E-01	3.37E-01	-0.29	(-2.16- 1.62)	7.66E-01	9.12E-01	-0.68	(-2.84- 1.53)	5.43E-01	7.15E-01
10	AC(18:2)	-1.15	(-1.90--0.32)	**7.70E-03**	**1.82E-02**	0.80	(-0.23- 1.94)	1.33E-01	2.80E-01	0.07	(-1.58- 1.75)	9.34E-01	9.76E-01	-0.58	(-2.82- 1.71)	6.12E-01	7.74E-01
11	CE(14:0)	-0.36	(-1.04- 0.36)	3.17E-01	4.49E-01	-0.68	(-1.50- 0.21)	1.30E-01	2.75E-01	2.68	(0.50- 4.90)	**1.59E-02**	9.00E-02	1.44	(-1.34- 4.29)	3.10E-01	5.08E-01
12	CE(16:1)	1.66	(0.73- 2.66)	**3.09E-04**	**9.28E-04**	0.19	(-0.76- 1.24)	7.07E-01	8.47E-01	5.39	(3.07- 7.76)	**6.25E-06**	**1.50E-03**	6.14	(3.51- 8.85)	**6.52E-06**	**1.04E-03**
13	CE(18:0)	0.37	(-0.68- 1.54)	5.01E-01	6.25E-01	0.32	(-0.81- 1.60)	5.90E-01	7.63E-01	-0.70	(-2.78- 1.44)	5.18E-01	7.51E-01	2.88	(-0.05- 5.89)	5.41E-02	1.76E-01
14	CE(18:1)	0.38	(-0.21- 1.02)	2.13E-01	3.28E-01	-0.15	(-0.78- 0.51)	6.38E-01	8.04E-01	0.10	(-0.92- 1.13)	8.48E-01	9.40E-01	0.71	(-0.63- 2.07)	2.96E-01	4.94E-01
15	CE(18:2)	-0.77	(-1.37--0.12)	**2.02E-02**	**4.22E-02**	0.13	(-0.70- 1.04)	7.60E-01	8.85E-01	0.13	(-1.29- 1.57)	8.59E-01	9.40E-01	0.65	(-0.78- 2.09)	3.74E-01	5.66E-01
16	CE(18:3)	0.10	(-0.98- 1.31)	8.60E-01	9.13E-01	0.68	(-0.71- 2.27)	3.52E-01	5.54E-01	2.38	(-0.29- 5.11)	8.07E-02	2.53E-01	3.38	(-0.03- 6.90)	5.17E-02	1.71E-01
17	CE(20:1)	1.28	(0.11- 2.58)	**3.06E-02**	6.24E-02	-0.37	(-1.47- 0.88)	5.44E-01	7.32E-01	-1.79	(-3.52--0.02)	**4.70E-02**	1.97E-01	-0.44	(-2.40- 1.55)	6.58E-01	8.10E-01
18	CE(20:2)	1.17	(0.30- 2.12)	**7.56E-03**	**1.80E-02**	0.23	(-0.68- 1.22)	6.38E-01	8.04E-01	0.00	(-1.42- 1.44)	9.99E-01	9.99E-01	0.94	(-0.79- 2.70)	2.86E-01	4.81E-01
19	CE(20:4)	-0.38	(-1.07- 0.37)	3.11E-01	4.44E-01	0.18	(-0.76- 1.21)	7.14E-01	8.47E-01	0.77	(-1.10- 2.67)	4.20E-01	6.78E-01	-0.10	(-2.65- 2.52)	9.42E-01	9.75E-01
20	CE(20:5)	-2.79	(-4.02--1.30)	**6.99E-04**	**2.00E-03**	-1.80	(-3.35- 0.12)	6.47E-02	1.61E-01	4.14	(0.08- 8.37)	**4.58E-02**	1.95E-01	3.58	(-2.12- 9.61)	2.21E-01	4.16E-01
21	CE(22:5) (n6)	0.42	(-0.56- 1.51)	4.11E-01	5.44E-01	0.44	(-0.74- 1.76)	4.80E-01	6.66E-01	0.91	(-0.98- 2.84)	3.44E-01	6.22E-01	1.14	(-0.95- 3.27)	2.87E-01	4.81E-01
22	CE(22:6)	-2.22	(-3.18--1.13)	**1.99E-04**	**6.27E-04**	-1.50	(-2.75--0.05)	**4.32E-02**	1.18E-01	2.64	(-0.21- 5.56)	6.93E-02	2.38E-01	1.44	(-1.89- 4.88)	3.98E-01	5.90E-01
23	CE(24:5)	-2.66	(-3.77--1.34)	**2.75E-04**	**8.45E-04**	-3.18	(-4.29--1.85)	**3.17E-05**	**3.05E-04**	-2.06	(-4.70- 0.66)	1.35E-01	3.48E-01	-0.11	(-2.85- 2.70)	9.37E-01	9.75E-01
24	CE(24:6)	-0.72	(-1.96- 0.72)	3.11E-01	4.44E-01	-1.59	(-2.88--0.06)	**4.25E-02**	1.16E-01	-0.14	(-2.51- 2.29)	9.08E-01	9.66E-01	2.09	(-1.39- 5.69)	2.41E-01	4.32E-01
25	DG(16:0_16:1)	-1.88	(-2.82--0.81)	**9.94E-04**	**2.76E-03**	-0.43	(-1.79- 1.15)	5.71E-01	7.51E-01	5.60	(2.49- 8.79)	**4.07E-04**	**1.50E-02**	7.08	(2.48-11.88)	**2.47E-03**	**2.97E-02**
26	DG(14:0_18:2)	-3.22	(-4.38--1.82)	**5.89E-05**	**2.02E-04**	-2.69	(-4.28--0.66)	**1.23E-02**	**4.20E-02**	4.96	(0.30- 9.84)	**3.66E-02**	1.64E-01	9.36	(3.33-15.75)	**2.21E-03**	**2.72E-02**
27	DG(16:0_18:1)	-2.54	(-3.32--1.67)	**3.15E-07**	**1.41E-06**	-0.88	(-2.05- 0.45)	1.84E-01	3.57E-01	2.76	(-0.43- 6.05)	9.02E-02	2.70E-01	5.44	(1.29- 9.76)	**1.01E-02**	6.90E-02
28	DG(16:1_18:1)	-2.51	(-3.34--1.57)	**2.13E-06**	**8.66E-06**	-1.33	(-2.53- 0.06)	5.94E-02	1.53E-01	5.07	(1.31- 8.98)	**8.12E-03**	6.04E-02	7.86	(3.21-12.72)	**8.87E-04**	**1.90E-02**
29	DG(16:0_18:2)	-3.79	(-4.59--2.88)	**3.80E-11**	**3.04E-10**	-1.96	(-3.17--0.53)	**9.02E-03**	**3.16E-02**	2.63	(-0.54- 5.90)	1.04E-01	2.94E-01	5.97	(2.18- 9.90)	**2.02E-03**	**2.63E-02**
30	DG(18:0_18:1)	-1.73	(-2.56--0.81)	**4.38E-04**	**1.29E-03**	-0.61	(-1.82- 0.78)	3.72E-01	5.75E-01	2.91	(0.07- 5.84)	**4.47E-02**	1.93E-01	5.77	(1.69-10.00)	**5.45E-03**	**4.59E-02**
31	DG(18:1_18:1)	-2.22	(-3.01--1.33)	**6.57E-06**	**2.50E-05**	-1.24	(-2.31--0.02)	**4.73E-02**	1.27E-01	1.16	(-1.81- 4.23)	4.45E-01	6.97E-01	3.38	(-0.31- 7.20)	7.26E-02	2.10E-01
32	DG(18:0_18:2)	-2.55	(-3.58--1.36)	**1.20E-04**	**3.97E-04**	-0.19	(-1.81- 1.76)	8.38E-01	9.33E-01	2.56	(-0.52- 5.74)	1.03E-01	2.93E-01	5.71	(1.57-10.02)	**6.78E-03**	5.51E-02
33	DG(18:1_18:2)	-3.75	(-4.45--2.96)	**9.13E-14**	**1.14E-12**	-2.19	(-3.30--0.90)	**1.57E-03**	**6.92E-03**	0.82	(-2.10- 3.83)	5.85E-01	8.05E-01	3.57	(0.14- 7.13)	**4.14E-02**	1.54E-01
34	DG(18:2_18:2)	-4.90	(-5.67--3.99)	**1.12E-14**	**1.85E-13**	-2.89	(-4.13--1.39)	**5.37E-04**	**2.93E-03**	1.30	(-2.03- 4.75)	4.46E-01	6.97E-01	4.10	(0.00- 8.37)	**4.99E-02**	1.71E-01
35	DG(18:1_18:3)	-3.19	(-4.08--2.15)	**1.73E-07**	**8.05E-07**	-1.52	(-2.89- 0.12)	6.76E-02	1.67E-01	1.95	(-1.75- 5.79)	3.04E-01	5.75E-01	5.41	(1.17- 9.83)	**1.23E-02**	7.46E-02
36	DG(16:0_20:4)	-2.73	(-3.63--1.69)	**3.52E-06**	**1.39E-05**	-0.95	(-2.29- 0.63)	2.23E-01	4.08E-01	4.44	(1.01- 7.98)	**1.10E-02**	6.76E-02	6.02	(1.85-10.35)	**4.58E-03**	**4.18E-02**
37	DG(18:1_20:3)	-0.41	(-1.44- 0.75)	4.76E-01	6.03E-01	0.00	(-1.21- 1.38)	9.99E-01	9.99E-01	-0.05	(-3.17- 3.18)	9.77E-01	9.94E-01	3.24	(-0.57- 7.20)	9.57E-02	2.50E-01
38	DG(18:1_20:4)	-2.44	(-3.23--1.56)	**9.71E-07**	**4.02E-06**	-1.43	(-2.51--0.20)	**2.48E-02**	7.31E-02	3.22	(0.37- 6.14)	**2.67E-02**	1.29E-01	5.18	(1.62- 8.87)	**4.35E-03**	**4.17E-02**
39	DG(16:0_22:5)	-2.24	(-3.18--1.17)	**1.36E-04**	**4.40E-04**	-1.33	(-2.64- 0.22)	8.92E-02	2.04E-01	2.97	(-0.34- 6.39)	7.91E-02	2.50E-01	7.25	(3.11-11.56)	**5.90E-04**	**1.57E-02**

40	DG(18:2_20:4)	-3.51	(-4.37--2.51)	**7.48E-09**	**4.23E-08**	-2.60	(-3.82--1.13)	**1.21E-03**	**5.67E-03**	3.79	(0.56- 7.13)	**2.13E-02**	1.10E-01	5.07	(1.23- 9.06)	**9.64E-03**	6.72E-02
41	DG(16:0_22:6)	-4.54	(-5.36--3.59)	**1.43E-12**	**1.43E-11**	-3.35	(-4.46--2.00)	**1.79E-05**	**1.87E-04**	5.24	(0.67-10.01)	**2.42E-02**	1.21E-01	5.67	(0.73-10.85)	**2.43E-02**	1.13E-01
42	DG(18:1_22:5)	-1.93	(-2.92--0.80)	**1.41E-03**	**3.82E-03**	-1.94	(-3.11--0.58)	**6.78E-03**	**2.58E-02**	0.81	(-2.44- 4.17)	6.28E-01	8.41E-01	2.39	(-0.96- 5.86)	1.63E-01	3.44E-01
43	DG(18:1_22:6)	-3.76	(-4.58--2.81)	**1.91E-10**	**1.37E-09**	-3.07	(-4.12--1.81)	**1.92E-05**	**1.92E-04**	3.21	(-0.40- 6.95)	8.20E-02	2.56E-01	5.85	(1.28-10.63)	**1.20E-02**	7.38E-02
44	DG(18:2_22:6)	-4.58	(-5.42--3.59)	**5.13E-12**	**4.56E-11**	-3.08	(-4.42--1.43)	**7.99E-04**	**4.00E-03**	3.42	(-0.90- 7.93)	1.21E-01	3.31E-01	7.28	(1.88-12.98)	**8.03E-03**	6.02E-02
45	TG(48:0) [NL-18:0]	-2.22	(-3.17--1.13)	**1.87E-04**	**5.94E-04**	-0.01	(-1.56- 1.82)	9.88E-01	9.94E-01	7.53	(2.92-12.35)	**1.30E-03**	**2.49E-02**	7.33	(1.10-13.95)	**2.08E-02**	1.05E-01
46	TG(48:1) [NL-16:1]	-3.12	(-4.11--1.98)	**2.70E-06**	**1.09E-05**	-1.26	(-2.92- 0.79)	2.11E-01	3.96E-01	9.50	(4.81-14.39)	**6.51E-05**	**4.46E-03**	9.88	(3.77-16.34)	**1.40E-03**	**2.49E-02**
47	TG(48:1) [NL-18:1]	-3.72	(-4.59--2.71)	**2.17E-09**	**1.37E-08**	-2.13	(-3.61--0.31)	**2.43E-02**	7.22E-02	7.66	(2.32-13.28)	**4.71E-03**	**4.82E-02**	9.11	(2.17-16.51)	**9.66E-03**	6.72E-02
48	TG(48:2) [NL-14:0]	-4.21	(-5.01--3.29)	**2.40E-12**	**2.31E-11**	-2.93	(-4.22--1.36)	**7.95E-04**	**4.00E-03**	7.99	(2.52-13.76)	**3.97E-03**	**4.82E-02**	10.05	(3.00-17.58)	**4.88E-03**	**4.18E-02**
49	TG(48:2) [NL-14:1]	-3.30	(-4.17--2.29)	**4.21E-08**	**2.06E-07**	-2.60	(-3.88--1.04)	**2.05E-03**	**8.87E-03**	9.57	(4.45-14.93)	**2.21E-04**	**9.89E-03**	7.84	(1.05-15.09)	**2.33E-02**	1.11E-01
50	TG(48:2) [NL-16:1]	-3.18	(-4.11--2.10)	**5.04E-07**	**2.18E-06**	-2.21	(-3.60--0.51)	**1.32E-02**	**4.40E-02**	10.34	(5.02-15.92)	**1.21E-04**	**6.46E-03**	11.55	(4.79-18.75)	**7.22E-04**	**1.76E-02**
51	TG(48:2) [NL-18:2]	-4.78	(-5.58--3.84)	**1.42E-13**	**1.66E-12**	-2.99	(-4.34--1.31)	**1.26E-03**	**5.81E-03**	7.95	(2.40-13.79)	**4.73E-03**	**4.82E-02**	9.77	(2.92-17.08)	**4.87E-03**	**4.18E-02**
52	TG(48:3) [NL-14:0]	-4.43	(-5.23--3.51)	**5.69E-13**	**6.35E-12**	-3.39	(-4.62--1.87)	**1.03E-04**	**8.22E-04**	8.83	(3.18-14.78)	**2.02E-03**	**3.23E-02**	11.17	(4.21-18.60)	**1.49E-03**	**2.56E-02**
53	TG(48:3) [NL-16:1]	-3.11	(-3.96--2.15)	**4.59E-08**	**2.22E-07**	-2.48	(-3.66--1.08)	**1.12E-03**	**5.34E-03**	8.05	(3.43-12.88)	**5.94E-04**	**1.59E-02**	9.57	(3.77-15.69)	**1.13E-03**	**2.08E-02**
54	TG(48:3) [NL-18:3]	-3.79	(-4.56--2.91)	**8.55E-12**	**7.33E-11**	-2.31	(-3.52--0.87)	**2.79E-03**	**1.16E-02**	8.11	(2.36-14.17)	**5.40E-03**	**4.82E-02**	8.74	(1.44-16.57)	**1.85E-02**	9.75E-02
55	TG(49:1) [NL-17:1]	-2.57	(-3.37--1.67)	**5.47E-07**	**2.34E-06**	-1.52	(-2.61--0.27)	**1.89E-02**	5.89E-02	4.87	(1.86- 7.96)	**1.50E-03**	**2.77E-02**	5.00	(0.73- 9.45)	**2.16E-02**	1.06E-01
56	TG(50:1) [NL-14:0]	-2.17	(-3.16--1.03)	**4.53E-04**	**1.32E-03**	-0.23	(-1.87- 1.74)	8.05E-01	9.14E-01	6.29	(2.13-10.62)	**2.96E-03**	**3.98E-02**	7.98	(2.08-14.21)	**7.72E-03**	5.98E-02
57	TG(50:1) [NL-16:0]	-3.30	(-4.13--2.35)	**6.72E-09**	**3.84E-08**	-1.09	(-2.55- 0.65)	2.04E-01	3.85E-01	4.46	(1.31- 7.70)	**5.42E-03**	**4.82E-02**	4.85	(0.60- 9.29)	**2.53E-02**	1.16E-01
58	TG(50:1) [NL-18:1]	-3.08	(-3.94--2.09)	**1.04E-07**	**4.95E-07**	-1.46	(-2.85- 0.21)	8.37E-02	1.93E-01	4.11	(1.07- 7.24)	**7.95E-03**	6.04E-02	4.47	(0.34- 8.77)	**3.39E-02**	1.35E-01
59	TG(50:2) [NL-14:0]	-3.73	(-4.62--2.69)	**5.05E-09**	**2.99E-08**	-2.59	(-3.95--0.93)	**3.78E-03**	**1.54E-02**	3.78	(-0.34- 8.07)	7.26E-02	2.45E-01	5.30	(0.07-10.80)	**4.68E-02**	1.67E-01
60	TG(50:2) [NL-16:1]	-3.52	(-4.43--2.47)	**3.08E-08**	**1.62E-07**	-2.09	(-3.54--0.30)	**2.41E-02**	7.22E-02	5.88	(1.92-10.00)	**3.51E-03**	**4.51E-02**	7.56	(2.75-12.60)	**1.98E-03**	**2.63E-02**
61	TG(50:2) [NL-18:1]	-3.55	(-4.47--2.48)	**3.91E-08**	**1.95E-07**	-2.31	(-3.74--0.56)	**1.23E-02**	**4.20E-02**	5.45	(1.48- 9.58)	**7.00E-03**	5.60E-02	7.61	(2.81-12.63)	**1.82E-03**	**2.63E-02**
62	TG(50:2) [NL-18:2]	-5.03	(-5.82--4.09)	**2.94E-14**	**3.92E-13**	-2.65	(-4.14--0.79)	**7.57E-03**	**2.82E-02**	4.85	(1.65- 8.16)	**2.98E-03**	**3.98E-02**	4.31	(0.54- 8.22)	**2.48E-02**	1.14E-01
63	TG(50:3) [NL-14:0]	-4.63	(-5.44--3.68)	**6.63E-13**	**7.24E-12**	-3.57	(-4.82--2.03)	**6.73E-05**	**5.57E-04**	4.38	(-0.26- 9.22)	6.42E-02	2.28E-01	7.21	(1.46-13.28)	**1.37E-02**	7.82E-02
64	TG(50:3) [NL-14:1]	-2.50	(-3.48--1.38)	**6.15E-05**	**2.09E-04**	-2.01	(-3.30--0.46)	**1.32E-02**	**4.40E-02**	4.51	(0.99- 8.16)	**1.20E-02**	7.12E-02	3.82	(-0.97- 8.83)	1.19E-01	2.83E-01
65	TG(50:3) [NL-16:1]	-4.37	(-5.18--3.44)	**1.66E-12**	**1.63E-11**	-3.01	(-4.27--1.47)	**4.48E-04**	**2.57E-03**	7.59	(3.01-12.37)	**1.10E-03**	**2.30E-02**	9.80	(4.47-15.41)	**2.95E-04**	**1.09E-02**
66	TG(50:3) [NL-18:2]	-5.01	(-5.76--4.13)	**1.19E-15**	**2.49E-14**	-3.22	(-4.54--1.59)	**4.43E-04**	**2.57E-03**	6.26	(1.84-10.89)	**5.41E-03**	**4.82E-02**	8.76	(3.53-14.26)	**9.67E-04**	**1.93E-02**
67	TG(50:3) [NL-18:3]	-4.66	(-5.43--3.77)	**2.57E-14**	**3.53E-13**	-2.28	(-3.75--0.47)	**1.62E-02**	5.20E-02	5.99	(1.39-10.81)	**1.05E-02**	6.73E-02	8.51	(2.79-14.55)	**3.40E-03**	**3.55E-02**
68	TG(50:4) [NL-14:0]	-4.84	(-5.57--3.97)	**1.09E-15**	**2.48E-14**	-3.48	(-4.68--2.00)	**4.78E-05**	**4.17E-04**	4.58	(-0.16- 9.55)	5.83E-02	2.17E-01	6.38	(0.70-12.37)	**2.74E-02**	1.19E-01
69	TG(50:4) [NL-18:3]	-4.57	(-5.27--3.76)	**1.60E-16**	**7.66E-15**	-2.79	(-4.01--1.33)	**5.52E-04**	**2.97E-03**	6.89	(2.13-11.88)	**4.40E-03**	**4.82E-02**	10.01	(4.14-16.21)	**7.70E-04**	**1.76E-02**
70	TG(50:4) [NL-20:4]	-4.27	(-5.17--3.19)	**7.40E-10**	**5.00E-09**	-2.20	(-3.75--0.28)	**2.72E-02**	7.78E-02	9.73	(4.17-15.58)	**5.40E-04**	**1.59E-02**	10.87	(3.92-18.28)	**1.97E-03**	**2.63E-02**
71	TG(51:1) [NL-17:0]	-3.15	(-4.00--2.18)	**3.94E-08**	**1.95E-07**	-2.01	(-3.26--0.52)	**1.01E-02**	**3.51E-02**	4.79	(1.36- 8.34)	**6.09E-03**	5.13E-02	4.99	(-0.16-10.40)	5.75E-02	1.83E-01
72	TG(51:2) [NL-15:0]	-3.51	(-4.41--2.46)	**3.27E-08**	**1.70E-07**	-2.96	(-4.18--1.48)	**3.55E-04**	**2.21E-03**	2.33	(-1.42- 6.22)	2.26E-01	4.75E-01	3.68	(-0.75- 8.31)	1.04E-01	2.65E-01
73	TG(51:2) [NL-17:0]	-3.68	(-4.53--2.70)	**1.23E-09**	**8.23E-09**	-2.87	(-4.09--1.40)	**4.50E-04**	**2.57E-03**	4.92	(1.21- 8.76)	**9.19E-03**	6.39E-02	6.43	(1.49-11.61)	**1.05E-02**	6.90E-02
74	TG(51:2) [NL-17:1]	-3.12	(-3.90--2.23)	**3.49E-09**	**2.15E-08**	-2.44	(-3.49--1.22)	**2.81E-04**	**1.88E-03**	4.39	(1.04- 7.85)	**1.01E-02**	6.71E-02	5.72	(1.25-10.39)	**1.20E-02**	7.38E-02
75	TG(52:1) [NL-18:0]	-2.68	(-3.68--1.52)	**3.70E-05**	**1.31E-04**	-0.47	(-2.16- 1.59)	6.29E-01	7.96E-01	5.25	(1.63- 8.99)	**4.38E-03**	**4.82E-02**	6.56	(1.39-12.00)	**1.26E-02**	7.47E-02
76	TG(52:1) [NL-18:1]	-2.69	(-3.69--1.55)	**3.02E-05**	**1.08E-04**	-0.30	(-2.03- 1.79)	7.57E-01	8.85E-01	4.81	(1.41- 8.33)	**5.55E-03**	**4.85E-02**	7.03	(1.90-12.41)	**7.00E-03**	5.51E-02
77	TG(52:2) [NL-16:0]	-3.28	(-4.05--2.41)	**5.03E-10**	**3.45E-09**	-1.92	(-3.07--0.58)	**6.63E-03**	**2.55E-02**	1.44	(-0.82- 3.75)	2.13E-01	4.62E-01	2.66	(-0.05- 5.43)	5.40E-02	1.76E-01
78	TG(52:2) [NL-18:2]	-3.74	(-4.64--2.69)	**7.94E-09**	**4.43E-08**	-1.05	(-2.65- 0.89)	2.67E-01	4.62E-01	3.99	(0.99- 7.08)	**9.19E-03**	6.39E-02	5.33	(1.49- 9.32)	**6.52E-03**	5.40E-02
79	TG(52:3) [NL-16:1]	-3.72	(-4.60--2.69)	**4.42E-09**	**2.65E-08**	-2.79	(-4.02--1.31)	**6.50E-04**	**3.46E-03**	3.16	(-0.06- 6.48)	5.47E-02	2.12E-01	3.77	(0.03- 7.65)	**4.81E-02**	1.69E-01
80	TG(52:3) [NL-18:2]	-5.00	(-5.73--4.14)	**2.68E-16**	**1.07E-14**	-3.38	(-4.64--1.82)	**1.47E-04**	**1.14E-03**	1.36	(-1.28- 4.06)	3.14E-01	5.90E-01	2.73	(-0.16- 5.70)	6.39E-02	1.90E-01
81	TG(52:4) [NL-16:1]	-4.96	(-5.71--4.08)	**1.14E-15**	**2.48E-14**	-3.77	(-4.93--2.34)	**9.19E-06**	**1.05E-04**	3.59	(-0.53- 7.88)	8.81E-02	2.66E-01	5.26	(0.57-10.17)	**2.77E-02**	1.19E-01
82	TG(52:4) [NL-18:2]	-5.66	(-6.38--4.80)	**9.57E-18**	**7.65E-16**	-3.76	(-5.02--2.17)	**5.65E-05**	**4.76E-04**	3.00	(-0.87- 7.02)	1.29E-01	3.47E-01	4.86	(0.11- 9.85)	**4.50E-02**	1.62E-01
83	TG(52:4) [NL-18:3]	-4.75	(-5.48--3.90)	**1.12E-15**	**2.48E-14**	-2.97	(-4.26--1.38)	**7.42E-04**	**3.87E-03**	3.67	(-0.16- 7.65)	6.01E-02	2.20E-01	5.99	(1.18-11.03)	**1.44E-02**	8.16E-02
84	TG(52:5) [NL-18:3]	-4.81	(-5.54--3.97)	**3.12E-16**	**1.07E-14**	-3.09	(-4.29--1.63)	**1.81E-04**	**1.32E-03**	3.83	(-0.26- 8.10)	6.66E-02	2.33E-01	7.81	(2.65-13.23)	**2.90E-03**	**3.19E-02**
85	TG(52:5) [NL-20:4]	-4.28	(-5.14--3.27)	**7.13E-11**	**5.43E-10**	-2.50	(-3.94--0.70)	**8.74E-03**	**3.13E-02**	9.64	(4.84-14.66)	**7.49E-05**	**4.49E-03**	11.13	(5.45-17.11)	**1.09E-04**	**5.25E-03**

86	TG(52:5) [NL-20:5]	-5.06	(-5.85--4.13)	**2.07E-14**	**3.01E-13**	-3.43	(-4.69--1.88)	**1.15E-04**	**9.08E-04**	7.27	(2.99-11.74)	**8.36E-04**	**2.04E-02**	9.30	(4.19-14.65)	**3.35E-04**	**1.14E-02**
87	TG(53:2) [NL-18:1]	-2.88	(-3.68--1.99)	**2.98E-08**	**1.60E-07**	-2.34	(-3.40--1.11)	**4.81E-04**	**2.71E-03**	1.38	(-1.60- 4.45)	3.67E-01	6.37E-01	2.62	(-1.14- 6.53)	1.73E-01	3.57E-01
88	TG(54:1) [NL-18:1]	-1.99	(-3.09--0.72)	**3.33E-03**	**8.56E-03**	-1.10	(-2.67- 0.82)	2.41E-01	4.30E-01	2.86	(-0.08- 5.88)	5.64E-02	2.17E-01	4.57	(0.39- 8.92)	**3.20E-02**	1.31E-01
89	TG(54:2) [NL-18:0]	-2.33	(-3.31--1.21)	**1.61E-04**	**5.18E-04**	-1.11	(-2.57- 0.64)	1.98E-01	3.76E-01	2.69	(-0.17- 5.64)	6.56E-02	2.32E-01	4.23	(0.14- 8.48)	**4.26E-02**	1.57E-01
90	TG(54:2) [NL-20:1]	-2.03	(-3.00--0.91)	**7.29E-04**	**2.06E-03**	-1.43	(-2.77- 0.16)	7.48E-02	1.78E-01	0.69	(-2.44- 3.92)	6.69E-01	8.54E-01	3.09	(-0.72- 7.05)	1.12E-01	2.75E-01
91	TG(54:3) [NL-18:1]	-2.50	(-3.37--1.52)	**5.92E-06**	**2.29E-05**	-1.73	(-2.89--0.38)	**1.40E-02**	**4.62E-02**	-0.38	(-2.60- 1.88)	7.35E-01	8.86E-01	1.10	(-1.75- 4.04)	4.51E-01	6.33E-01
92	TG(54:3) [NL-18:2]	-3.84	(-4.60--2.97)	**3.74E-12**	**3.39E-11**	-1.87	(-3.14--0.36)	**1.73E-02**	5.50E-02	2.13	(-0.23- 4.54)	7.65E-02	2.45E-01	2.55	(-0.44- 5.63)	9.51E-02	2.50E-01
93	TG(54:4) [NL-18:2]	-4.54	(-5.34--3.59)	**7.27E-13**	**7.59E-12**	-3.20	(-4.49--1.62)	**3.40E-04**	**2.19E-03**	0.22	(-2.47- 2.98)	8.76E-01	9.47E-01	1.43	(-2.12- 5.10)	4.33E-01	6.15E-01
94	TG(54:4) [NL-20:3]	-3.15	(-4.06--2.09)	**3.70E-07**	**1.63E-06**	-1.95	(-3.36--0.24)	**2.72E-02**	7.78E-02	4.06	(0.73- 7.50)	**1.67E-02**	9.22E-02	6.70	(2.74-10.82)	**9.11E-04**	**1.90E-02**
95	TG(54:5) [NL-18:3]	-4.38	(-5.12--3.54)	**1.18E-14**	**1.85E-13**	-2.71	(-3.93--1.24)	**8.01E-04**	**4.00E-03**	1.63	(-2.12- 5.52)	3.98E-01	6.68E-01	5.33	(0.39-10.50)	**3.41E-02**	1.35E-01
96	TG(54:5) [NL-20:4]	-4.47	(-5.29--3.51)	**2.78E-12**	**2.56E-11**	-2.41	(-3.89--0.57)	**1.28E-02**	**4.34E-02**	5.48	(2.17- 8.90)	**1.17E-03**	**2.34E-02**	6.77	(3.23-10.43)	**1.81E-04**	**7.88E-03**
97	TG(54:6) [NL-18:3]	-4.46	(-5.21--3.59)	**2.51E-14**	**3.53E-13**	-2.83	(-4.04--1.36)	**5.10E-04**	**2.82E-03**	1.25	(-2.82- 5.50)	5.50E-01	7.82E-01	6.09	(0.82-11.62)	**2.31E-02**	1.11E-01
98	TG(54:6) [NL-20:4]	-4.82	(-5.61--3.88)	**9.29E-14**	**1.14E-12**	-2.87	(-4.25--1.16)	**2.15E-03**	**9.22E-03**	6.68	(2.90-10.60)	**5.14E-04**	**1.59E-02**	9.25	(4.84-13.84)	**3.85E-05**	**3.08E-03**
99	TG(54:6) [NL-20:5]	-5.63	(-6.35--4.75)	**2.45E-17**	**1.47E-15**	-3.99	(-5.22--2.43)	**1.89E-05**	**1.92E-04**	7.67	(3.27-12.26)	**5.97E-04**	**1.59E-02**	10.66	(5.44-16.14)	**5.87E-05**	**4.03E-03**
100	TG(54:6) [NL-22:6]	-5.67	(-6.44--4.73)	**1.49E-15**	**2.97E-14**	-4.31	(-5.49--2.82)	**2.99E-06**	**4.09E-05**	8.61	(3.84-13.59)	**3.69E-04**	**1.47E-02**	7.77	(1.81-14.07)	**1.02E-02**	6.90E-02
101	TG(54:7) [NL-20:5]	-5.55	(-6.29--4.66)	**9.60E-17**	**5.12E-15**	-4.07	(-5.26--2.58)	**6.79E-06**	**8.14E-05**	7.69	(3.39-12.17)	**4.37E-04**	**1.50E-02**	10.93	(5.96-16.14)	**1.59E-05**	**1.53E-03**
102	TG(54:7) [NL-22:6]	-5.27	(-6.06--4.34)	**7.77E-15**	**1.33E-13**	-4.34	(-5.40--3.02)	**1.65E-07**	**3.61E-06**	10.45	(5.29-15.86)	**6.36E-05**	**4.46E-03**	10.52	(4.54-16.84)	**5.16E-04**	**1.46E-02**
103	TG(56:6) [NL-20:4]	-3.58	(-4.43--2.61)	**1.98E-09**	**1.28E-08**	-2.08	(-3.48--0.39)	**1.84E-02**	5.77E-02	3.64	(1.29- 6.06)	**2.50E-03**	**3.63E-02**	4.30	(1.39- 7.28)	**3.75E-03**	**3.83E-02**
104	TG(56:6) [NL-22:5]	-4.23	(-5.02--3.33)	**1.07E-12**	**1.09E-11**	-3.05	(-4.30--1.53)	**3.52E-04**	**2.21E-03**	3.90	(0.93- 6.97)	**1.01E-02**	6.71E-02	4.13	(1.07- 7.29)	**8.24E-03**	6.02E-02
105	TG(56:7) [NL-20:4]	-4.36	(-5.20--3.38)	**1.38E-11**	**1.17E-10**	-2.47	(-3.86--0.76)	**6.63E-03**	**2.55E-02**	3.96	(0.81- 7.21)	**1.35E-02**	7.74E-02	4.93	(1.03- 8.99)	**1.32E-02**	7.63E-02
106	TG(56:7) [NL-20:5]	-4.42	(-5.25--3.44)	**7.75E-12**	**6.76E-11**	-2.75	(-4.04--1.18)	**1.37E-03**	**6.19E-03**	5.08	(1.17- 9.15)	**1.08E-02**	6.76E-02	7.20	(2.68-11.92)	**1.74E-03**	**2.63E-02**
107	TG(56:7) [NL-22:5]	-4.79	(-5.52--3.93)	**8.16E-16**	**2.06E-14**	-3.45	(-4.60--2.05)	**2.32E-05**	**2.27E-04**	4.85	(1.24- 8.58)	**8.32E-03**	6.05E-02	5.93	(1.83-10.19)	**4.51E-03**	**4.18E-02**
108	TG(56:7) [NL-22:6]	-5.45	(-6.23--4.51)	**3.63E-15**	**6.46E-14**	-4.45	(-5.55--3.08)	**2.88E-07**	**5.53E-06**	6.41	(1.90-11.11)	**5.16E-03**	**4.82E-02**	6.57	(1.26-12.16)	**1.50E-02**	8.40E-02
109	TG(56:8) [NL-20:4]	-3.75	(-4.62--2.74)	**2.02E-09**	**1.29E-08**	-1.57	(-3.00- 0.14)	7.05E-02	1.72E-01	4.51	(1.22- 7.90)	**7.15E-03**	5.62E-02	6.47	(2.25-10.87)	**2.61E-03**	**3.06E-02**
110	TG(56:8) [NL-20:5]	-4.81	(-5.55--3.94)	**2.16E-15**	**4.14E-14**	-3.07	(-4.30--1.59)	**2.42E-04**	**1.69E-03**	4.10	(0.03- 8.35)	**4.84E-02**	1.98E-01	7.48	(2.98-12.19)	**1.11E-03**	**2.08E-02**
111	TG(56:8) [NL-22:6]	-5.84	(-6.57--4.96)	**2.22E-17**	**1.47E-15**	-4.61	(-5.66--3.32)	**3.86E-08**	**1.42E-06**	7.11	(2.31-12.15)	**3.57E-03**	**4.51E-02**	8.30	(3.04-13.83)	**1.89E-03**	**2.63E-02**
112	TG(56:9) [NL-22:6]	-5.30	(-6.04--4.42)	**2.03E-16**	**8.86E-15**	-4.24	(-5.30--2.94)	**1.95E-07**	**3.90E-06**	5.85	(1.37-10.53)	**1.02E-02**	6.71E-02	8.01	(2.89-13.38)	**2.08E-03**	**2.63E-02**
113	TG(58:10) [NL-22:6]	-5.02	(-5.75--4.16)	**2.94E-16**	**1.07E-14**	-3.63	(-4.76--2.26)	**7.78E-06**	**9.11E-05**	5.10	(0.69- 9.69)	**2.31E-02**	1.17E-01	5.55	(0.57-10.77)	**2.86E-02**	1.21E-01
114	TG(58:8) [NL-22:6]	-4.95	(-5.76--4.00)	**1.12E-13**	**1.34E-12**	-3.76	(-4.86--2.43)	**2.50E-06**	**3.64E-05**	4.39	(-0.08- 9.06)	5.42E-02	2.11E-01	5.12	(0.12-10.38)	**4.49E-02**	1.62E-01
115	TG(58:9) [NL-22:6]	-5.22	(-5.96--4.33)	**4.35E-16**	**1.33E-14**	-4.05	(-5.11--2.76)	**3.75E-07**	**6.67E-06**	3.42	(-0.99- 8.03)	1.29E-01	3.47E-01	6.25	(1.09-11.68)	**1.73E-02**	9.33E-02
116	TG(O-50:1) [NL-16:0]	-0.91	(-1.82- 0.11)	7.75E-02	1.42E-01	-0.15	(-1.24- 1.08)	8.04E-01	9.14E-01	1.93	(-0.91- 4.85)	1.83E-01	4.24E-01	2.23	(-1.09- 5.66)	1.89E-01	3.72E-01
117	TG(O-50:1) [NL-17:1]	-2.62	(-3.44--1.70)	**5.75E-07**	**2.44E-06**	-1.48	(-2.61--0.18)	**2.71E-02**	7.78E-02	4.94	(1.86- 8.11)	**1.64E-03**	**2.81E-02**	5.04	(0.70- 9.57)	**2.28E-02**	1.11E-01
118	TG(O-50:1) [NL-18:1]	-1.31	(-2.20--0.31)	**1.16E-02**	**2.57E-02**	-0.47	(-1.58- 0.78)	4.41E-01	6.29E-01	3.27	(0.25- 6.37)	**3.36E-02**	1.52E-01	2.73	(-1.04- 6.65)	1.57E-01	3.40E-01
119	TG(O-50:2) [NL-18:2]	-0.54	(-1.53- 0.56)	3.20E-01	4.52E-01	-0.24	(-1.39- 1.07)	7.04E-01	8.47E-01	3.60	(0.90- 6.36)	**8.82E-03**	6.32E-02	2.19	(-1.06- 5.55)	1.86E-01	3.70E-01
120	TG(O-52:2) [NL-16:0]	-1.09	(-2.30- 0.31)	1.20E-01	2.02E-01	-1.08	(-2.40- 0.48)	1.64E-01	3.31E-01	4.14	(0.42- 8.00)	**2.91E-02**	1.40E-01	1.81	(-2.69- 6.51)	4.35E-01	6.16E-01
121	TG(O-52:2) [NL-17:1]	-3.15	(-3.95--2.26)	**3.90E-09**	**2.37E-08**	-2.42	(-3.49--1.17)	**4.01E-04**	**2.41E-03**	4.41	(1.03- 7.90)	**1.05E-02**	6.73E-02	5.69	(1.18-10.39)	**1.32E-02**	7.63E-02
122	TG(O-52:2) [NL-18:1]	-1.39	(-2.63- 0.06)	5.90E-02	1.12E-01	-2.16	(-3.40--0.70)	**5.47E-03**	**2.17E-02**	5.31	(1.22- 9.58)	**1.09E-02**	6.76E-02	4.33	(-0.27- 9.15)	6.54E-02	1.93E-01
123	TG(O-54:2) [NL-18:1]	-2.88	(-3.68--1.99)	**3.31E-08**	**1.71E-07**	-2.28	(-3.35--1.03)	**7.73E-04**	**3.99E-03**	1.34	(-1.63- 4.40)	3.79E-01	6.53E-01	2.58	(-1.17- 6.48)	1.79E-01	3.65E-01
124	TG(O-54:4) [NL-17:1]	-4.46	(-5.28--3.50)	**2.66E-12**	**2.51E-11**	-3.21	(-4.43--1.71)	**1.55E-04**	**1.18E-03**	2.45	(-1.04- 6.06)	1.69E-01	4.07E-01	3.54	(-0.34- 7.58)	7.38E-02	2.11E-01
125	TG(O-54:4) [NL-18:2]	-5.23	(-5.97--4.34)	**4.44E-16**	**1.33E-14**	-3.45	(-4.69--1.92)	**9.27E-05**	**7.54E-04**	2.63	(-1.04- 6.44)	1.61E-01	3.95E-01	2.86	(-1.47- 7.39)	1.97E-01	3.82E-01
126	dhCer(d18:0/22:0)	0.02	(-0.87- 1.00)	9.65E-01	9.88E-01	-0.15	(-1.17- 0.99)	7.89E-01	9.01E-01	1.33	(-1.12- 3.85)	2.87E-01	5.55E-01	1.21	(-1.38- 3.86)	3.60E-01	5.57E-01
127	dhCer(d18:0/24:0)	-0.64	(-1.46- 0.27)	1.63E-01	2.64E-01	-0.26	(-1.36- 0.98)	6.69E-01	8.33E-01	0.07	(-2.14- 2.33)	9.50E-01	9.83E-01	-0.13	(-2.50- 2.29)	9.13E-01	9.74E-01
128	dhCer(d18:0/24:1)	0.39	(-0.80- 1.73)	5.33E-01	6.57E-01	-0.93	(-2.05- 0.35)	1.48E-01	3.06E-01	1.80	(-0.84- 4.51)	1.82E-01	4.24E-01	1.38	(-1.82- 4.67)	4.00E-01	5.90E-01
129	Cer(d16:1/22:0)	-1.09	(-1.99--0.09)	**3.43E-02**	6.86E-02	-0.93	(-2.09- 0.40)	1.61E-01	3.27E-01	1.94	(-0.68- 4.63)	1.46E-01	3.64E-01	3.14	(0.04- 6.34)	**4.68E-02**	1.67E-01
130	Cer(d16:1/23:0)	-0.97	(-2.54- 0.93)	2.96E-01	4.29E-01	-1.00	(-2.68- 1.06)	3.14E-01	5.08E-01	1.84	(-1.15- 4.91)	2.29E-01	4.78E-01	3.13	(-0.35- 6.73)	7.82E-02	2.18E-01
131	Cer(d16:1/24:0)	-0.99	(-1.94- 0.07)	6.68E-02	1.23E-01	-0.55	(-1.64- 0.67)	3.60E-01	5.61E-01	1.20	(-1.61- 4.08)	4.04E-01	6.75E-01	3.27	(0.08- 6.55)	**4.43E-02**	1.62E-01

132	Cer(d16:1/24:1)	-0.69	(-1.81- 0.58)	2.72E-01	4.00E-01	-0.12	(-1.50- 1.48)	8.74E-01	9.49E-01	1.16	(-1.12- 3.50)	3.18E-01	5.94E-01	3.31	(0.23- 6.48)	**3.49E-02**	1.37E-01
133	Cer(d17:1/22:0)	-0.81	(-2.00- 0.56)	2.33E-01	3.51E-01	-0.84	(-2.19- 0.74)	2.77E-01	4.73E-01	0.81	(-1.73- 3.42)	5.32E-01	7.65E-01	1.34	(-1.57- 4.33)	3.68E-01	5.64E-01
134	Cer(d17:1/23:0)	-1.93	(-3.39--0.16)	**3.39E-02**	6.81E-02	-0.26	(-2.64- 2.89)	8.52E-01	9.38E-01	1.79	(-1.17- 4.84)	2.37E-01	4.91E-01	2.09	(-1.37- 5.66)	2.37E-01	4.31E-01
135	Cer(d17:1/24:0)	-1.25	(-2.17--0.23)	**1.80E-02**	**3.85E-02**	-0.76	(-1.90- 0.54)	2.40E-01	4.30E-01	0.35	(-2.27- 3.03)	7.96E-01	9.16E-01	-0.11	(-2.85- 2.70)	9.35E-01	9.75E-01
136	Cer(d17:1/24:1)	-1.39	(-2.39--0.25)	**1.80E-02**	**3.85E-02**	-0.09	(-1.43- 1.46)	9.03E-01	9.63E-01	1.34	(-1.18- 3.92)	3.00E-01	5.71E-01	0.84	(-2.17- 3.95)	5.84E-01	7.50E-01
137	Cer(d18:1/16:0)	-0.44	(-1.18- 0.36)	2.71E-01	3.99E-01	-0.54	(-1.45- 0.47)	2.81E-01	4.75E-01	-0.57	(-2.30- 1.18)	5.17E-01	7.51E-01	-0.07	(-2.07- 1.97)	9.45E-01	9.75E-01
138	Cer(d18:1/18:0)	-0.77	(-1.59- 0.14)	9.51E-02	1.69E-01	-0.64	(-1.70- 0.54)	2.73E-01	4.70E-01	1.53	(-0.75- 3.85)	1.88E-01	4.29E-01	2.70	(-0.06- 5.53)	5.50E-02	1.77E-01
139	Cer(d18:1/20:0)	-0.75	(-1.57- 0.14)	9.73E-02	1.72E-01	-0.73	(-1.72- 0.37)	1.83E-01	3.55E-01	1.44	(-0.66- 3.58)	1.79E-01	4.20E-01	0.36	(-2.00- 2.78)	7.67E-01	8.89E-01
140	Cer(d18:1/22:0)	-0.33	(-1.07- 0.49)	4.19E-01	5.53E-01	0.31	(-0.67- 1.40)	5.47E-01	7.34E-01	1.19	(-0.79- 3.21)	2.38E-01	4.91E-01	0.69	(-1.37- 2.80)	5.11E-01	6.95E-01
141	Cer(d18:1/23:0)	-0.69	(-1.59- 0.30)	1.63E-01	2.64E-01	0.13	(-1.16- 1.61)	8.51E-01	9.38E-01	0.86	(-1.19- 2.95)	4.11E-01	6.75E-01	1.89	(-0.27- 4.09)	8.62E-02	2.35E-01
142	Cer(d18:1/24:0)	-0.72	(-1.46- 0.09)	7.91E-02	1.43E-01	0.16	(-0.84- 1.26)	7.67E-01	8.85E-01	0.25	(-1.65- 2.18)	7.97E-01	9.16E-01	0.12	(-1.72- 2.00)	8.98E-01	9.70E-01
143	Cer(d18:1/24:1)	-0.42	(-1.22- 0.46)	3.41E-01	4.71E-01	-0.10	(-1.17- 1.09)	8.57E-01	9.41E-01	1.32	(-0.57- 3.25)	1.72E-01	4.11E-01	-0.14	(-2.18- 1.95)	8.97E-01	9.70E-01
144	Cer(d18:2/16:0)	-0.65	(-1.77- 0.63)	3.06E-01	4.40E-01	1.39	(-0.18- 3.22)	8.54E-02	1.96E-01	0.26	(-2.08- 2.66)	8.28E-01	9.33E-01	1.02	(-1.93- 4.07)	4.98E-01	6.83E-01
145	Cer(d18:2/22:0)	-0.81	(-1.69- 0.17)	1.01E-01	1.75E-01	0.56	(-0.60- 1.86)	3.58E-01	5.60E-01	0.29	(-1.78- 2.39)	7.86E-01	9.16E-01	0.85	(-1.81- 3.58)	5.33E-01	7.11E-01
146	Cer(d18:2/23:0)	-0.88	(-2.01- 0.41)	1.70E-01	2.72E-01	0.56	(-1.06- 2.47)	5.20E-01	7.08E-01	0.99	(-1.30- 3.32)	3.98E-01	6.68E-01	1.80	(-0.89- 4.57)	1.90E-01	3.72E-01
147	Cer(d18:2/24:0)	-0.98	(-1.86--0.00)	**4.96E-02**	9.48E-02	-0.45	(-1.55- 0.79)	4.58E-01	6.47E-01	-0.88	(-2.89- 1.17)	3.97E-01	6.68E-01	0.09	(-2.34- 2.58)	9.40E-01	9.75E-01
148	Cer(d18:2/24:1)	0.03	(-0.91- 1.07)	9.55E-01	9.79E-01	0.03	(-1.14- 1.35)	9.64E-01	9.86E-01	0.50	(-1.53- 2.57)	6.31E-01	8.42E-01	0.92	(-1.53- 3.43)	4.63E-01	6.46E-01
149	Cer(d19:1/24:0)	-0.93	(-2.01- 0.30)	1.33E-01	2.22E-01	-1.46	(-2.65--0.06)	**4.13E-02**	1.14E-01	0.49	(-3.24- 4.36)	7.99E-01	9.16E-01	-0.26	(-4.45- 4.12)	9.05E-01	9.71E-01
150	Cer(d19:1/24:1)	-0.55	(-1.88- 1.01)	4.67E-01	5.93E-01	-2.07	(-3.42--0.45)	**1.49E-02**	**4.87E-02**	1.52	(-2.52- 5.72)	4.65E-01	7.18E-01	0.78	(-4.13- 5.94)	7.59E-01	8.85E-01
151	Cer(m18:0/22:0)	-0.67	(-1.89- 0.74)	3.33E-01	4.65E-01	-1.77	(-3.11--0.16)	**3.30E-02**	9.22E-02	1.99	(-0.60- 4.65)	1.32E-01	3.48E-01	3.74	(0.81- 6.75)	**1.24E-02**	7.46E-02
152	Cer(m18:0/23:0)	-3.06	(-4.80--0.73)	**1.34E-02**	**2.97E-02**	1.54	(-1.71- 6.07)	3.94E-01	5.91E-01	2.29	(-0.70- 5.38)	1.34E-01	3.48E-01	3.57	(-0.39- 7.68)	7.77E-02	2.18E-01
153	Cer(m18:0/24:0)	-1.48	(-2.39--0.46)	**5.63E-03**	**1.38E-02**	-0.56	(-1.79- 0.87)	4.22E-01	6.18E-01	2.03	(-0.41- 4.53)	1.03E-01	2.93E-01	2.54	(-0.33- 5.50)	8.29E-02	2.28E-01
154	Cer(m18:0/24:1)	-1.01	(-2.26- 0.45)	1.64E-01	2.64E-01	-2.11	(-3.51--0.40)	**1.82E-02**	5.75E-02	3.88	(0.85- 7.01)	**1.20E-02**	7.12E-02	5.40	(2.05- 8.86)	**1.59E-03**	**2.62E-02**
155	Cer(m18:1/22:0)	-2.74	(-3.55--1.84)	**1.44E-07**	**6.76E-07**	-2.05	(-3.15--0.77)	**2.79E-03**	**1.16E-02**	0.15	(-2.59- 2.96)	9.16E-01	9.69E-01	3.27	(-0.61- 7.30)	9.93E-02	2.58E-01
156	Cer(m18:1/23:0)	-3.02	(-4.50--1.15)	**3.15E-03**	**8.17E-03**	-3.90	(-5.70--1.34)	**5.88E-03**	**2.30E-02**	2.36	(-0.98- 5.81)	1.67E-01	4.06E-01	1.68	(-2.29- 5.82)	4.09E-01	5.97E-01
157	Cer(m18:1/24:0)	-2.64	(-3.45--1.74)	**3.32E-07**	**1.48E-06**	-2.36	(-3.41--1.14)	**4.08E-04**	**2.42E-03**	-0.49	(-3.07- 2.17)	7.14E-01	8.85E-01	1.01	(-2.29- 4.43)	5.50E-01	7.24E-01
158	Cer(m18:1/24:1)	-1.76	(-2.83--0.52)	**6.74E-03**	**1.62E-02**	-2.53	(-3.57--1.31)	**1.76E-04**	**1.31E-03**	0.60	(-2.10- 3.38)	6.63E-01	8.54E-01	3.93	(0.25- 7.74)	**3.63E-02**	1.40E-01
159	GM3(d18:1/18:0)	-0.87	(-1.62--0.06)	**3.50E-02**	6.96E-02	-0.55	(-1.50- 0.50)	2.92E-01	4.89E-01	-0.52	(-2.29- 1.28)	5.66E-01	7.93E-01	-2.21	(-4.46- 0.10)	6.06E-02	1.88E-01
160	GM3(d18:1/22:0)	-0.04	(-1.21- 1.29)	9.51E-01	9.77E-01	0.35	(-1.27- 2.26)	6.93E-01	8.47E-01	-0.44	(-2.44- 1.60)	6.69E-01	8.54E-01	-0.29	(-2.92- 2.41)	8.30E-01	9.23E-01
161	GM3(d18:1/24:0)	-0.66	(-1.64- 0.44)	2.28E-01	3.47E-01	-0.16	(-1.35- 1.20)	8.09E-01	9.16E-01	-2.09	(-3.98--0.17)	**3.36E-02**	1.52E-01	-2.80	(-5.04--0.50)	**1.76E-02**	9.38E-02
162	GM3(d18:1/24:1)	-0.02	(-1.04- 1.13)	9.75E-01	9.89E-01	-0.14	(-1.40- 1.30)	8.41E-01	9.33E-01	-1.02	(-2.87- 0.87)	2.87E-01	5.55E-01	-2.51	(-4.97- 0.01)	5.06E-02	1.71E-01
163	HexCer(d16:1/22:0)	-0.85	(-2.39- 0.99)	3.41E-01	4.71E-01	-0.92	(-2.88- 1.59)	4.37E-01	6.27E-01	-0.57	(-3.29- 2.23)	6.85E-01	8.62E-01	-0.95	(-3.91- 2.11)	5.37E-01	7.12E-01
164	HexCer(d16:1/24:0)	-0.91	(-2.25- 0.66)	2.40E-01	3.60E-01	-0.76	(-2.59- 1.53)	4.84E-01	6.67E-01	0.97	(-1.90- 3.91)	5.11E-01	7.47E-01	-0.57	(-3.45- 2.41)	7.04E-01	8.48E-01
165	HexCer(d18:1/16:0)	0.24	(-0.82- 1.43)	6.66E-01	7.48E-01	-0.25	(-1.48- 1.15)	7.06E-01	8.47E-01	-0.79	(-2.70- 1.17)	4.26E-01	6.81E-01	-2.68	(-4.63--0.68)	**9.26E-03**	6.64E-02
166	HexCer(d18:1/18:0)	-0.03	(-1.30- 1.43)	9.71E-01	9.89E-01	0.61	(-1.01- 2.53)	4.82E-01	6.66E-01	-1.68	(-3.97- 0.67)	1.58E-01	3.89E-01	-1.86	(-4.53- 0.89)	1.83E-01	3.70E-01
167	HexCer(d18:1/20:0)	-0.11	(-1.28- 1.23)	8.67E-01	9.19E-01	0.21	(-1.24- 1.90)	7.93E-01	9.04E-01	-1.76	(-4.03- 0.56)	1.34E-01	3.48E-01	-3.20	(-5.58--0.76)	**1.08E-02**	6.90E-02
168	HexCer(d18:1/22:0)	0.30	(-0.68- 1.38)	5.64E-01	6.80E-01	0.84	(-0.34- 2.16)	1.71E-01	3.39E-01	-1.61	(-3.63- 0.44)	1.23E-01	3.33E-01	-3.03	(-5.05--0.96)	**4.64E-03**	**4.18E-02**
169	HexCer(d18:1/24:0)	0.22	(-0.78- 1.32)	6.80E-01	7.61E-01	-0.07	(-1.18- 1.18)	9.11E-01	9.69E-01	-0.95	(-2.87- 1.00)	3.34E-01	6.14E-01	-2.91	(-5.07--0.70)	**1.06E-02**	6.90E-02
170	HexCer(d18:1/24:1)	0.81	(-0.21- 1.94)	1.22E-01	2.06E-01	0.21	(-0.92- 1.48)	7.26E-01	8.57E-01	-0.23	(-2.22- 1.80)	8.23E-01	9.32E-01	-2.92	(-5.12--0.66)	**1.18E-02**	7.38E-02
171	Hex2Cer(d16:1/16:0)	-1.29	(-2.52- 0.13)	7.36E-02	1.35E-01	-0.64	(-2.19- 1.22)	4.72E-01	6.59E-01	2.53	(-0.25- 5.39)	7.44E-02	2.45E-01	2.38	(-1.06- 5.93)	1.75E-01	3.60E-01
172	Hex2Cer(d18:1/16:0)	-0.46	(-1.32- 0.49)	3.33E-01	4.65E-01	-0.50	(-1.58- 0.71)	3.99E-01	5.96E-01	-0.92	(-3.16- 1.37)	4.25E-01	6.81E-01	-1.58	(-4.25- 1.16)	2.54E-01	4.44E-01
173	Hex2Cer(d18:1/22:0)	-0.10	(-1.22- 1.18)	8.76E-01	9.20E-01	-0.27	(-1.55- 1.20)	7.03E-01	8.47E-01	-2.30	(-4.36--0.19)	**3.26E-02**	1.52E-01	-2.58	(-5.23- 0.15)	6.38E-02	1.90E-01
174	Hex2Cer(d18:1/24:1)	-0.09	(-1.11- 1.05)	8.75E-01	9.20E-01	-0.15	(-1.36- 1.22)	8.19E-01	9.20E-01	-2.61	(-4.86--0.32)	**2.59E-02**	1.27E-01	-1.91	(-4.63- 0.88)	1.76E-01	3.60E-01
175	Hex2Cer(d18:2/16:0)	-0.69	(-1.70- 0.44)	2.18E-01	3.34E-01	0.03	(-1.19- 1.43)	9.59E-01	9.85E-01	1.38	(-0.81- 3.62)	2.18E-01	4.66E-01	0.15	(-2.47- 2.85)	9.10E-01	9.73E-01
176	Hex3Cer(d18:1/16:0)	-0.37	(-1.11- 0.44)	3.60E-01	4.87E-01	-0.54	(-1.47- 0.49)	2.94E-01	4.89E-01	-0.68	(-2.09- 0.76)	3.51E-01	6.24E-01	-0.66	(-2.52- 1.24)	4.90E-01	6.78E-01
177	Hex3Cer(d18:1/22:0)	0.32	(-0.86- 1.65)	6.14E-01	7.17E-01	0.05	(-1.47- 1.84)	9.52E-01	9.82E-01	-1.58	(-3.65- 0.54)	1.43E-01	3.60E-01	-1.47	(-3.84- 0.95)	2.30E-01	4.27E-01

178	Hex3Cer(d18:1/24:0)	-0.65	(-1.60- 0.41)	2.20E-01	3.36E-01	0.07	(-1.17- 1.48)	9.21E-01	9.70E-01	-2.36	(-4.26--0.41)	**1.79E-02**	9.75E-02	-1.74	(-3.95- 0.52)	1.29E-01	3.01E-01
179	Hex3Cer(d18:1/24:1)	-0.48	(-1.41- 0.55)	3.47E-01	4.75E-01	-0.50	(-1.68- 0.86)	4.50E-01	6.38E-01	-0.34	(-2.19- 1.54)	7.17E-01	8.85E-01	-1.89	(-4.13- 0.39)	1.03E-01	2.64E-01
180	SM(d17:1/14:0)	-0.65	(-1.33- 0.08)	7.79E-02	1.42E-01	-1.33	(-2.10--0.48)	**2.83E-03**	**1.17E-02**	1.99	(-0.50- 4.54)	1.17E-01	3.25E-01	3.07	(-0.05- 6.29)	5.35E-02	1.76E-01
181	SM(d18:0/14:0)	0.28	(-0.43- 1.04)	4.48E-01	5.75E-01	-0.91	(-1.64--0.11)	**2.70E-02**	7.78E-02	1.29	(-0.76- 3.38)	2.18E-01	4.66E-01	2.32	(-0.21- 4.91)	7.26E-02	2.10E-01
182	SM(d18:1/14:0)/SM(d16:1/16:0)	-0.27	(-0.92- 0.43)	4.36E-01	5.66E-01	-0.84	(-1.61- 0.00)	5.12E-02	1.36E-01	1.74	(-0.25- 3.78)	8.64E-02	2.66E-01	2.96	(0.46- 5.53)	**2.05E-02**	1.05E-01
183	SM(d18:2/14:0)	0.33	(-0.35- 1.06)	3.50E-01	4.79E-01	-0.17	(-1.01- 0.75)	7.02E-01	8.47E-01	5.30	(3.41- 7.21)	**6.74E-08**	**3.24E-05**	8.07	(5.72-10.47)	**1.01E-10**	**4.83E-08**
184	SM(d17:1/16:0)	-0.92	(-1.54--0.24)	**8.73E-03**	**2.03E-02**	-1.01	(-1.78--0.17)	**2.03E-02**	6.25E-02	-0.43	(-2.40- 1.58)	6.71E-01	8.54E-01	-0.16	(-2.61- 2.36)	9.01E-01	9.70E-01
185	SM(d18:1/16:0)	-0.23	(-0.98- 0.58)	5.66E-01	6.80E-01	-0.43	(-1.33- 0.56)	3.78E-01	5.75E-01	-1.51	(-3.36- 0.38)	1.15E-01	3.23E-01	-1.47	(-3.73- 0.85)	2.12E-01	4.01E-01
186	SM(d18:2/16:0)	0.19	(-0.46- 0.89)	5.74E-01	6.85E-01	0.00	(-0.81- 0.87)	9.97E-01	9.99E-01	0.75	(-0.62- 2.14)	2.84E-01	5.53E-01	2.14	(0.41- 3.90)	**1.57E-02**	8.55E-02
187	SM(34:3)	0.48	(-0.25- 1.26)	1.99E-01	3.12E-01	-0.26	(-1.10- 0.67)	5.76E-01	7.52E-01	1.94	(-0.06- 3.98)	5.77E-02	2.17E-01	4.73	(2.25- 7.28)	**2.10E-04**	**8.39E-03**
188	SM(d18:2/17:0)	-0.74	(-1.40--0.04)	**3.75E-02**	7.37E-02	-1.05	(-1.83--0.21)	**1.60E-02**	5.15E-02	0.98	(-0.78- 2.78)	2.76E-01	5.46E-01	1.61	(-0.69- 3.97)	1.69E-01	3.54E-01
189	SM(35:2) (b)	-0.24	(-1.22- 0.85)	6.50E-01	7.36E-01	-1.13	(-2.18- 0.06)	6.18E-02	1.56E-01	-1.15	(-3.40- 1.15)	3.21E-01	5.98E-01	-0.73	(-3.52- 2.14)	6.13E-01	7.74E-01
190	SM(d18:1/18:0)/SM(d16:1/20:0)	-0.21	(-0.94- 0.58)	5.87E-01	6.98E-01	0.20	(-0.73- 1.23)	6.83E-01	8.39E-01	0.13	(-1.70- 1.99)	8.93E-01	9.59E-01	0.94	(-1.33- 3.26)	4.19E-01	6.04E-01
191	SM(d18:2/18:1)	0.37	(-0.40- 1.19)	3.56E-01	4.85E-01	-0.44	(-1.33- 0.54)	3.66E-01	5.68E-01	0.92	(-0.67- 2.54)	2.55E-01	5.14E-01	2.18	(0.24- 4.16)	**2.81E-02**	1.19E-01
192	SM(37:1)	-0.91	(-1.71--0.04)	**4.10E-02**	7.91E-02	-0.63	(-1.64- 0.49)	2.56E-01	4.48E-01	0.13	(-1.90- 2.20)	9.02E-01	9.64E-01	0.37	(-2.29- 3.10)	7.87E-01	8.98E-01
193	SM(37:2)	-0.70	(-1.47- 0.14)	9.82E-02	1.73E-01	-0.74	(-1.67- 0.30)	1.58E-01	3.22E-01	-0.55	(-2.77- 1.72)	6.29E-01	8.41E-01	-0.33	(-2.97- 2.39)	8.10E-01	9.17E-01
194	SM(d18:1/20:0)/SM(d16:1/22:0)	0.06	(-0.64- 0.80)	8.74E-01	9.20E-01	0.62	(-0.26- 1.58)	1.71E-01	3.39E-01	0.37	(-1.21- 1.98)	6.44E-01	8.52E-01	0.99	(-1.07- 3.09)	3.45E-01	5.41E-01
195	SM(d18:2/20:0)	0.26	(-0.65- 1.26)	5.87E-01	6.98E-01	0.45	(-0.74- 1.80)	4.74E-01	6.60E-01	0.22	(-1.74- 2.22)	8.26E-01	9.33E-01	1.04	(-1.41- 3.55)	4.06E-01	5.96E-01
196	SM(38:3) (a)	-0.18	(-0.91- 0.60)	6.35E-01	7.30E-01	-0.96	(-1.76--0.08)	**3.34E-02**	9.26E-02	1.86	(0.24- 3.52)	**2.49E-02**	1.23E-01	2.01	(0.01- 4.05)	**4.88E-02**	1.69E-01
197	SM(38:3) (b)	0.11	(-0.83- 1.14)	8.31E-01	8.90E-01	-0.21	(-1.28- 0.99)	7.14E-01	8.47E-01	-0.30	(-2.65- 2.11)	8.03E-01	9.16E-01	1.95	(-0.75- 4.73)	1.57E-01	3.40E-01
198	SM(d16:1/23:0)/SM(d17:1/22:0)	-0.46	(-1.12- 0.26)	2.03E-01	3.16E-01	-0.19	(-1.02- 0.71)	6.62E-01	8.28E-01	1.00	(-1.03- 3.07)	3.36E-01	6.14E-01	2.18	(-0.31- 4.73)	8.66E-02	2.35E-01
199	SM(d18:1/22:0)/SM(d16:1/24:0)	0.22	(-0.51- 1.00)	5.64E-01	6.80E-01	0.71	(-0.20- 1.70)	1.32E-01	2.79E-01	0.47	(-1.22- 2.20)	5.84E-01	8.05E-01	0.50	(-1.47- 2.52)	6.18E-01	7.76E-01
200	SM(d16:1/24:1)	0.13	(-0.66- 0.98)	7.60E-01	8.22E-01	-0.42	(-1.32- 0.59)	4.01E-01	5.96E-01	2.65	(0.81- 4.53)	**4.85E-03**	**4.82E-02**	2.77	(0.21- 5.39)	**3.39E-02**	1.35E-01
201	SM(d18:2/22:0)	0.08	(-0.66- 0.87)	8.37E-01	8.90E-01	0.91	(-0.11- 2.04)	8.05E-02	1.87E-01	-0.02	(-1.56- 1.54)	9.78E-01	9.94E-01	1.34	(-0.63- 3.35)	1.83E-01	3.70E-01
202	SM(40:3) (a)	-0.26	(-1.01- 0.56)	5.27E-01	6.50E-01	-0.61	(-1.49- 0.36)	2.06E-01	3.88E-01	1.80	(0.02- 3.60)	**4.73E-02**	1.97E-01	1.75	(-0.50- 4.05)	1.26E-01	2.98E-01
203	SM(40:3) (b)	-0.72	(-1.50- 0.12)	9.23E-02	1.65E-01	-0.99	(-1.97- 0.10)	7.25E-02	1.74E-01	0.58	(-1.73- 2.95)	6.23E-01	8.38E-01	1.69	(-1.18- 4.63)	2.49E-01	4.40E-01
204	SM(41:0)	-0.29	(-1.44- 1.02)	6.51E-01	7.36E-01	-0.66	(-1.99- 0.90)	3.87E-01	5.82E-01	0.08	(-1.58- 1.76)	9.27E-01	9.71E-01	1.03	(-0.99- 3.08)	3.18E-01	5.17E-01
205	SM(41:1)	-0.33	(-1.14- 0.56)	4.52E-01	5.78E-01	0.08	(-0.95- 1.23)	8.82E-01	9.56E-01	0.36	(-1.33- 2.08)	6.74E-01	8.56E-01	0.83	(-1.16- 2.85)	4.15E-01	6.02E-01
206	SM(d17:1/24:1)	-0.21	(-0.92- 0.54)	5.67E-01	6.80E-01	-0.74	(-1.58- 0.19)	1.14E-01	2.46E-01	0.68	(-1.01- 2.40)	4.31E-01	6.87E-01	0.56	(-1.67- 2.84)	6.24E-01	7.82E-01
207	SM(d18:2/23:0)	-0.18	(-1.01- 0.73)	6.93E-01	7.70E-01	0.34	(-0.76- 1.58)	5.55E-01	7.37E-01	0.38	(-1.20- 1.97)	6.40E-01	8.51E-01	1.43	(-0.56- 3.45)	1.59E-01	3.41E-01
208	SM(d18:1/24:0)	-0.43	(-1.12- 0.32)	2.55E-01	3.81E-01	0.15	(-0.79- 1.19)	7.63E-01	8.85E-01	-0.52	(-2.00- 0.99)	4.96E-01	7.35E-01	-0.33	(-2.11- 1.48)	7.15E-01	8.49E-01
209	SM(d18:1/24:1)	0.49	(-0.30- 1.35)	2.29E-01	3.48E-01	-0.14	(-1.03- 0.82)	7.61E-01	8.85E-01	0.19	(-1.56- 1.97)	8.32E-01	9.35E-01	-0.55	(-2.58- 1.52)	5.97E-01	7.62E-01
210	SM(d18:2/24:0)	-0.39	(-1.07- 0.34)	2.84E-01	4.15E-01	0.39	(-0.55- 1.42)	4.26E-01	6.21E-01	-0.61	(-1.92- 0.72)	3.68E-01	6.37E-01	-0.11	(-1.83- 1.64)	9.00E-01	9.70E-01
211	SM(43:1)	-0.63	(-1.58- 0.43)	2.32E-01	3.51E-01	-1.42	(-2.48--0.21)	**2.33E-02**	7.02E-02	-1.48	(-3.37- 0.45)	1.31E-01	3.48E-01	-1.13	(-3.45- 1.25)	3.48E-01	5.43E-01
212	SM(44:1)	-0.46	(-1.10- 0.22)	1.78E-01	2.83E-01	-0.37	(-1.19- 0.53)	4.11E-01	6.07E-01	-2.46	(-3.86--1.04)	**8.52E-04**	**2.04E-02**	-1.52	(-3.23- 0.23)	8.83E-02	2.37E-01
213	SM(44:2)	-0.01	(-0.68- 0.72)	9.84E-01	9.92E-01	-0.42	(-1.25- 0.48)	3.42E-01	5.43E-01	-1.16	(-2.80- 0.50)	1.69E-01	4.07E-01	-1.20	(-3.16- 0.80)	2.36E-01	4.31E-01
214	SM(44:3) (a)	-0.37	(-1.00- 0.30)	2.67E-01	3.95E-01	-0.38	(-1.16- 0.48)	3.75E-01	5.75E-01	-0.41	(-1.83- 1.02)	5.69E-01	7.94E-01	-0.71	(-2.52- 1.13)	4.46E-01	6.28E-01
215	LPC(14:0) [sn2]	2.00	(1.33- 2.71)	**1.28E-09**	**8.39E-09**	1.49	(0.75- 2.28)	**5.65E-05**	**4.76E-04**	4.13	(1.16- 7.19)	**6.34E-03**	5.25E-02	6.01	(1.90-10.30)	**4.15E-03**	**4.07E-02**
216	LPC(14:0) [sn1]	1.76	(1.12- 2.43)	**2.26E-08**	**1.23E-07**	1.49	(0.76- 2.27)	**4.09E-05**	**3.65E-04**	3.98	(1.07- 6.96)	**7.29E-03**	5.64E-02	5.83	(1.87- 9.93)	**3.84E-03**	**3.84E-02**
217	LPC(15:0) [sn2]	1.45	(0.66- 2.30)	**2.29E-04**	**7.18E-04**	0.60	(-0.26- 1.53)	1.75E-01	3.44E-01	0.77	(-2.04- 3.65)	5.95E-01	8.10E-01	3.15	(-0.54- 6.98)	9.44E-02	2.49E-01
218	LPC(15:0) [sn1]	0.93	(0.23- 1.69)	**8.96E-03**	**2.07E-02**	0.67	(-0.13- 1.53)	1.01E-01	2.26E-01	0.40	(-2.22- 3.08)	7.69E-01	9.12E-01	2.66	(-0.77- 6.21)	1.29E-01	3.01E-01
219	LPC(16:0) [sn2]	1.54	(0.94- 2.17)	**1.98E-07**	**9.14E-07**	2.07	(1.31- 2.88)	**2.86E-08**	**1.15E-06**	-0.59	(-2.69- 1.54)	5.82E-01	8.05E-01	2.50	(-0.13- 5.20)	6.20E-02	1.88E-01
220	LPC(16:0) [sn1]	1.85	(1.12- 2.64)	**3.00E-07**	**1.36E-06**	2.15	(1.25- 3.13)	**1.15E-06**	**1.79E-05**	-0.69	(-3.16- 1.83)	5.85E-01	8.05E-01	2.18	(-0.81- 5.25)	1.53E-01	3.40E-01
221	LPC(16:1) [sn2]	4.38	(3.37- 5.47)	**4.90E-20**	**1.18E-17**	3.52	(2.46- 4.66)	**2.65E-12**	**1.27E-09**	4.20	(1.71- 6.76)	**9.57E-04**	**2.14E-02**	6.65	(3.67- 9.72)	**1.49E-05**	**1.53E-03**
222	LPC(16:1) [sn1]	4.38	(3.43- 5.40)	**2.15E-22**	**1.03E-19**	3.29	(2.29- 4.37)	**6.50E-12**	**1.56E-09**	3.09	(0.68- 5.56)	**1.19E-02**	7.12E-02	5.26	(2.38- 8.22)	**3.56E-04**	**1.14E-02**
223	LPC(15-MHDA) [sn2]	1.64	(0.37- 3.07)	**1.03E-02**	**2.35E-02**	-0.03	(-1.21- 1.32)	9.65E-01	9.86E-01	-1.63	(-5.04- 1.91)	3.61E-01	6.36E-01	1.64	(-2.82- 6.29)	4.75E-01	6.59E-01

224	LPC(15-MHDA) [sn1] / LPC(17:0) [sn2]	0.99	(0.04- 2.03)	**4.04E-02**	7.83E-02	0.02	(-0.93- 1.06)	9.72E-01	9.87E-01	-2.02	(-4.94- 0.99)	1.85E-01	4.25E-01	0.77	(-2.85- 4.52)	6.80E-01	8.31E-01
225	LPC(17:0) [sn1]	0.13	(-0.49- 0.79)	6.88E-01	7.66E-01	0.34	(-0.40- 1.14)	3.77E-01	5.75E-01	-2.13	(-4.56- 0.35)	9.18E-02	2.70E-01	0.61	(-2.30- 3.60)	6.84E-01	8.33E-01
226	LPC(17:1) [sn1] (a) / LPC(17:1) [sn2] (b)	2.44	(1.55- 3.41)	**2.06E-08**	**1.14E-07**	0.87	(-0.01- 1.82)	5.23E-02	1.38E-01	0.00	(-2.59- 2.66)	9.99E-01	9.99E-01	2.13	(-1.21- 5.59)	2.12E-01	4.01E-01
227	LPC(18:0) [sn2]	0.72	(0.16- 1.31)	**1.09E-02**	**2.43E-02**	1.42	(0.64- 2.25)	**2.43E-04**	**1.69E-03**	-0.75	(-3.04- 1.59)	5.25E-01	7.57E-01	2.97	(0.20- 5.81)	**3.56E-02**	1.38E-01
228	LPC(18:0) [sn1]	0.81	(0.23- 1.43)	**5.76E-03**	**1.40E-02**	1.29	(0.50- 2.13)	**1.03E-03**	**4.99E-03**	-0.72	(-3.29- 1.92)	5.87E-01	8.05E-01	2.97	(-0.01- 6.04)	5.08E-02	1.71E-01
229	LPC(18:1) [sn2]	3.96	(3.00- 4.99)	**4.21E-18**	**4.04E-16**	3.16	(2.16- 4.24)	**4.94E-11**	**5.23E-09**	-1.92	(-4.17- 0.37)	9.93E-02	2.86E-01	0.79	(-2.24- 3.90)	6.13E-01	7.74E-01
230	LPC(18:1) [sn1]	3.71	(2.86- 4.62)	**1.22E-19**	**1.96E-17**	2.91	(2.01- 3.87)	**2.21E-11**	**3.54E-09**	-1.93	(-4.00- 0.19)	7.43E-02	2.45E-01	0.02	(-2.53- 2.65)	9.86E-01	9.91E-01
231	LPC(18:2) [sn2]	2.88	(1.95- 3.88)	**1.19E-10**	**8.82E-10**	2.95	(1.85- 4.14)	**2.38E-08**	**1.04E-06**	-1.36	(-3.44- 0.77)	2.09E-01	4.58E-01	-0.57	(-3.16- 2.10)	6.71E-01	8.24E-01
232	LPC(18:2) [sn1]	2.93	(1.99- 3.95)	**9.62E-11**	**7.21E-10**	2.87	(1.77- 4.07)	**6.68E-08**	**2.14E-06**	-1.55	(-3.85- 0.81)	1.95E-01	4.38E-01	-1.69	(-4.46- 1.16)	2.41E-01	4.32E-01
233	LPC(18:3) [sn2] (a)	2.28	(1.42- 3.20)	**5.55E-08**	**2.67E-07**	2.29	(1.27- 3.40)	**4.21E-06**	**5.32E-05**	0.04	(-2.69- 2.85)	9.76E-01	9.94E-01	1.46	(-2.11- 5.16)	4.26E-01	6.08E-01
234	LPC(18:3) [sn1] (a)/LPC(18:3) [sn2] (b)	4.69	(3.44- 6.07)	**7.42E-16**	**2.04E-14**	4.00	(2.66- 5.48)	**2.33E-10**	**1.87E-08**	0.54	(-2.41- 3.59)	7.20E-01	8.86E-01	2.51	(-1.38- 6.54)	2.07E-01	3.98E-01
235	LPC(18:3) (a) [sn1] [104_sn1]	4.40	(3.21- 5.70)	**2.66E-15**	**4.91E-14**	3.62	(2.33- 5.04)	**2.99E-09**	**1.60E-07**	0.80	(-2.16- 3.84)	5.99E-01	8.11E-01	2.36	(-1.54- 6.40)	2.37E-01	4.31E-01
236	LPC(19:0) [sn1] (a) / LPC(19:0) [sn2] (b)	0.02	(-0.83- 0.94)	9.73E-01	9.89E-01	-0.49	(-1.41- 0.52)	3.25E-01	5.20E-01	-3.27	(-5.49--0.99)	**5.33E-03**	**4.82E-02**	-0.95	(-3.63- 1.80)	4.92E-01	6.78E-01
237	LPC(20:0) [sn1]	0.27	(-0.27- 0.83)	3.36E-01	4.67E-01	0.39	(-0.28- 1.12)	2.61E-01	4.51E-01	-4.17	(-6.10--2.20)	**5.50E-05**	**4.46E-03**	-2.07	(-4.53- 0.45)	1.06E-01	2.68E-01
238	LPC(20:1) [sn2]	1.66	(0.97- 2.38)	**8.89E-07**	**3.74E-06**	1.10	(0.31- 1.95)	**5.90E-03**	**2.30E-02**	-3.18	(-5.31--1.00)	**4.74E-03**	**4.82E-02**	-0.02	(-2.60- 2.63)	9.88E-01	9.91E-01
239	LPC(20:1) [sn1]	2.06	(1.41- 2.75)	**1.46E-10**	**1.06E-09**	1.32	(0.60- 2.09)	**2.38E-04**	**1.69E-03**	-3.45	(-5.56--1.28)	**2.11E-03**	**3.27E-02**	-0.70	(-3.17- 1.83)	5.79E-01	7.46E-01
240	LPC(20:2) [sn2]	3.91	(2.89- 5.02)	**7.66E-16**	**2.04E-14**	3.66	(2.49- 4.95)	**5.44E-11**	**5.23E-09**	-1.54	(-3.61- 0.58)	1.52E-01	3.75E-01	1.28	(-1.25- 3.88)	3.22E-01	5.19E-01
241	LPC(20:2) [sn1]	3.72	(2.82- 4.68)	**3.71E-18**	**4.04E-16**	2.88	(1.83- 4.03)	**1.33E-08**	**6.37E-07**	-1.87	(-3.97- 0.28)	8.69E-02	2.66E-01	0.84	(-1.68- 3.42)	5.16E-01	6.98E-01
242	LPC(20:3) [sn2]	2.83	(1.94- 3.80)	**5.17E-11**	**4.07E-10**	2.33	(1.30- 3.47)	**4.18E-06**	**5.32E-05**	1.02	(-1.28- 3.37)	3.84E-01	6.55E-01	5.44	(2.42- 8.55)	**4.27E-04**	**1.28E-02**
243	LPC(20:3) [sn1]	3.53	(2.56- 4.59)	**3.33E-14**	**4.32E-13**	2.97	(1.84- 4.21)	**4.25E-08**	**1.46E-06**	0.88	(-1.71- 3.53)	5.07E-01	7.44E-01	4.99	(1.89- 8.19)	**1.64E-03**	**2.62E-02**
244	LPC(20:3) [104_sn1]	3.58	(2.60- 4.65)	**2.02E-14**	**3.01E-13**	2.80	(1.72- 3.99)	**9.31E-08**	**2.74E-06**	0.99	(-1.63- 3.68)	4.62E-01	7.17E-01	5.08	(1.85- 8.41)	**2.07E-03**	**2.63E-02**
245	LPC(20:4) [sn2]	2.46	(1.61- 3.37)	**2.40E-09**	**1.50E-08**	2.31	(1.35- 3.35)	**8.21E-07**	**1.36E-05**	0.06	(-2.09- 2.24)	9.59E-01	9.88E-01	4.00	(1.39- 6.67)	**2.70E-03**	**3.09E-02**
246	LPC(20:4) [sn1]	2.74	(1.83- 3.72)	**4.87E-10**	**3.39E-09**	2.59	(1.58- 3.70)	**1.46E-07**	**3.50E-06**	0.00	(-2.41- 2.46)	9.97E-01	9.99E-01	2.65	(-0.11- 5.48)	5.97E-02	1.87E-01
247	LPC(20:5) [sn2]	0.31	(-1.09- 1.93)	6.80E-01	7.61E-01	0.70	(-0.99- 2.71)	4.37E-01	6.27E-01	3.35	(-0.26- 7.09)	6.93E-02	2.38E-01	6.65	(2.03-11.49)	**4.73E-03**	**4.18E-02**
248	LPC(20:5) [sn1]	0.69	(-0.74- 2.35)	3.63E-01	4.90E-01	0.57	(-1.07- 2.51)	5.17E-01	7.05E-01	3.54	(-0.28- 7.50)	6.95E-02	2.38E-01	6.45	(1.75-11.36)	**6.99E-03**	5.51E-02
249	LPC(22:4) [sn2]	2.45	(1.53- 3.43)	**3.64E-08**	**1.86E-07**	2.66	(1.58- 3.85)	**4.14E-07**	**7.09E-06**	-1.43	(-3.81- 1.01)	2.46E-01	5.03E-01	2.15	(-0.87- 5.25)	1.63E-01	3.44E-01
250	LPC(22:4) [sn1]	2.78	(1.91- 3.70)	**3.03E-11**	**2.47E-10**	2.61	(1.60- 3.72)	**1.03E-07**	**2.74E-06**	-0.19	(-2.72- 2.40)	8.82E-01	9.49E-01	2.15	(-0.85- 5.23)	1.60E-01	3.41E-01
251	LPC(22:5) [sn2] (n3)	1.51	(0.45- 2.67)	**4.32E-03**	**1.09E-02**	2.18	(0.81- 3.71)	**1.22E-03**	**5.68E-03**	-0.01	(-2.16- 2.18)	9.91E-01	9.99E-01	2.49	(-0.14- 5.18)	6.37E-02	1.90E-01
252	LPC(22:5) [sn1] (n3)/LPC(22:5) [sn2] (n6)	1.94	(1.04- 2.92)	**1.19E-05**	**4.49E-05**	2.46	(1.30- 3.73)	**1.26E-05**	**1.37E-04**	-0.32	(-2.56- 1.98)	7.84E-01	9.16E-01	2.00	(-0.65- 4.72)	1.39E-01	3.14E-01
253	LPC(22:5) [sn1] (n6)	4.71	(3.19- 6.41)	**2.08E-11**	**1.72E-10**	4.30	(2.54- 6.32)	**1.85E-07**	**3.86E-06**	-1.41	(-4.32- 1.58)	3.50E-01	6.24E-01	1.02	(-2.44- 4.60)	5.67E-01	7.36E-01
254	LPC(22:5) (n3) [sn1] [104_sn1]	0.84	(-0.13- 1.89)	9.17E-02	1.64E-01	1.38	(0.17- 2.74)	**2.44E-02**	7.22E-02	0.33	(-1.98- 2.68)	7.83E-01	9.16E-01	1.91	(-0.91- 4.81)	1.85E-01	3.70E-01
255	LPC(22:6) [sn2]	1.49	(0.60- 2.45)	**7.81E-04**	**2.19E-03**	1.86	(0.82- 3.00)	**3.09E-04**	**2.03E-03**	-0.09	(-2.54- 2.41)	9.42E-01	9.79E-01	1.58	(-1.47- 4.73)	3.10E-01	5.08E-01
256	LPC(22:6) [sn1]	2.03	(1.07- 3.07)	**1.50E-05**	**5.60E-05**	2.19	(1.10- 3.38)	**4.10E-05**	**3.65E-04**	0.27	(-2.39- 3.01)	8.41E-01	9.40E-01	0.63	(-2.40- 3.75)	6.86E-01	8.34E-01
257	LPC(O-16:0)	1.64	(0.97- 2.36)	**8.96E-07**	**3.74E-06**	1.94	(1.17- 2.76)	**3.11E-07**	**5.74E-06**	-0.82	(-3.14- 1.56)	4.94E-01	7.35E-01	2.27	(-0.57- 5.20)	1.18E-01	2.83E-01
258	LPC(O-18:0)	0.55	(-0.23- 1.38)	1.71E-01	2.74E-01	0.05	(-0.77- 0.94)	9.15E-01	9.70E-01	-2.38	(-4.94- 0.25)	7.56E-02	2.45E-01	0.76	(-2.38- 3.99)	6.38E-01	7.96E-01
259	LPC(O-18:1)	1.90	(1.20- 2.64)	**3.74E-08**	**1.89E-07**	1.74	(0.96- 2.57)	**6.71E-06**	**8.14E-05**	-1.36	(-3.72- 1.05)	2.64E-01	5.28E-01	1.34	(-1.42- 4.18)	3.41E-01	5.39E-01
260	LPC(O-20:0)	0.44	(-0.66- 1.67)	4.43E-01	5.70E-01	-0.41	(-1.60- 0.96)	5.39E-01	7.27E-01	-3.78	(-6.12--1.38)	**2.35E-03**	**3.53E-02**	-0.31	(-3.22- 2.69)	8.39E-01	9.30E-01
261	LPC(O-20:1)	2.44	(1.22- 3.79)	**4.14E-05**	**1.45E-04**	3.29	(1.62- 5.21)	**3.99E-05**	**3.65E-04**	-0.97	(-3.77- 1.91)	5.01E-01	7.38E-01	1.82	(-2.15- 5.96)	3.71E-01	5.66E-01
262	LPC(O-22:0)	0.23	(-0.41- 0.91)	4.88E-01	6.13E-01	0.22	(-0.50- 1.00)	5.57E-01	7.37E-01	-1.81	(-3.29--0.31)	**1.87E-02**	1.01E-01	-1.92	(-3.89- 0.09)	6.11E-02	1.88E-01
263	LPC(O-22:1)	1.62	(0.86- 2.44)	**1.60E-05**	**5.90E-05**	0.80	(-0.07- 1.73)	7.14E-02	1.73E-01	-2.10	(-4.24- 0.10)	6.10E-02	2.20E-01	-1.54	(-4.28- 1.28)	2.80E-01	4.80E-01
264	LPC(O-24:0)	-0.48	(-1.17- 0.26)	1.94E-01	3.06E-01	-0.38	(-1.13- 0.45)	3.58E-01	5.60E-01	-1.06	(-2.58- 0.49)	1.77E-01	4.20E-01	-1.85	(-3.86- 0.21)	7.72E-02	2.18E-01
265	LPC(O-24:1)	0.86	(0.15- 1.61)	**1.61E-02**	**3.54E-02**	-0.04	(-0.79- 0.78)	9.26E-01	9.70E-01	-0.68	(-2.41- 1.09)	4.48E-01	6.98E-01	-0.18	(-2.56- 2.26)	8.84E-01	9.67E-01
266	LPC(O-24:2)	0.25	(-0.43- 0.97)	4.82E-01	6.07E-01	0.13	(-0.69- 1.03)	7.58E-01	8.85E-01	-1.61	(-3.70- 0.53)	1.38E-01	3.52E-01	-1.36	(-4.11- 1.47)	3.41E-01	5.39E-01
267	LPC(P-16:0)	1.02	(0.39- 1.69)	**1.27E-03**	**3.51E-03**	1.30	(0.55- 2.10)	**5.10E-04**	**2.82E-03**	-0.77	(-3.08- 1.60)	5.20E-01	7.52E-01	2.16	(-0.52- 4.90)	1.14E-01	2.80E-01
268	LPC(P-18:0)	1.89	(1.20- 2.63)	**3.00E-08**	**1.60E-07**	1.58	(0.81- 2.39)	**3.27E-05**	**3.08E-04**	-1.58	(-3.89- 0.78)	1.86E-01	4.25E-01	1.26	(-1.51- 4.10)	3.75E-01	5.66E-01
269	LPC(P-18:1)	1.24	(0.40- 2.14)	**3.19E-03**	**8.23E-03**	1.39	(0.34- 2.56)	**8.86E-03**	**3.15E-02**	-0.72	(-3.39- 2.02)	6.00E-01	8.11E-01	3.19	(-0.01- 6.49)	5.06E-02	1.71E-01

270	LPC(P-20:0)	2.59	(1.67- 3.57)	**5.48E-09**	**3.21E-08**	1.26	(0.33- 2.29)	**7.45E-03**	**2.80E-02**	-2.47	(-4.95- 0.07)	5.69E-02	2.17E-01	-0.91	(-4.05- 2.34)	5.76E-01	7.45E-01
271	LPE(16:0) [sn2]	1.63	(0.86- 2.47)	**2.17E-05**	**7.90E-05**	3.34	(2.23- 4.55)	**2.77E-10**	**1.90E-08**	-2.11	(-4.27- 0.11)	6.24E-02	2.23E-01	-1.46	(-4.09- 1.23)	2.83E-01	4.80E-01
272	LPE(16:0) [sn1]	1.81	(1.08- 2.59)	**4.95E-07**	**2.16E-06**	3.02	(1.99- 4.14)	**1.07E-09**	**6.42E-08**	-1.76	(-3.77- 0.28)	9.04E-02	2.70E-01	-1.01	(-3.55- 1.61)	4.44E-01	6.26E-01
273	LPE(18:0) [sn2]	1.12	(0.47- 1.82)	**6.66E-04**	**1.93E-03**	1.94	(1.04- 2.90)	**1.05E-05**	**1.17E-04**	-1.99	(-3.76--0.19)	**3.03E-02**	1.43E-01	0.07	(-2.43- 2.63)	9.57E-01	9.75E-01
274	LPE(18:0) [sn1]	1.17	(0.55- 1.83)	**1.80E-04**	**5.75E-04**	2.23	(1.30- 3.23)	**9.65E-07**	**1.54E-05**	-1.75	(-3.65- 0.18)	7.51E-02	2.45E-01	0.39	(-2.11- 2.96)	7.61E-01	8.85E-01
275	LPE(18:1) [sn2]	2.78	(1.90- 3.72)	**5.35E-11**	**4.14E-10**	2.29	(1.28- 3.39)	**3.51E-06**	**4.67E-05**	-2.39	(-4.81- 0.08)	5.79E-02	2.17E-01	0.11	(-3.12- 3.44)	9.48E-01	9.75E-01
276	LPE(18:1) [sn1]	3.45	(2.51- 4.46)	**1.19E-14**	**1.85E-13**	2.27	(1.30- 3.34)	**1.89E-06**	**2.84E-05**	-1.74	(-4.22- 0.81)	1.77E-01	4.20E-01	-0.13	(-3.42- 3.27)	9.40E-01	9.75E-01
277	LPE(18:2) [sn2]	3.67	(2.45- 5.01)	**1.97E-10**	**1.39E-09**	3.61	(2.16- 5.24)	**1.53E-07**	**3.51E-06**	-1.63	(-4.13- 0.93)	2.08E-01	4.58E-01	-1.73	(-4.83- 1.46)	2.82E-01	4.80E-01
278	LPE(18:2) [sn1]	3.94	(2.79- 5.19)	**3.20E-13**	**3.65E-12**	3.44	(2.08- 4.96)	**1.00E-07**	**2.74E-06**	-1.56	(-4.10- 1.05)	2.37E-01	4.91E-01	-1.71	(-4.76- 1.45)	2.83E-01	4.80E-01
279	LPE(20:4) [sn1]	3.50	(2.47- 4.61)	**6.96E-13**	**7.43E-12**	2.76	(1.68- 3.95)	**1.42E-07**	**3.50E-06**	-1.81	(-3.60- 0.02)	5.20E-02	2.08E-01	-0.42	(-2.66- 1.88)	7.16E-01	8.49E-01
280	LPE(22:6) [sn2]	2.13	(1.18- 3.15)	**4.65E-06**	**1.82E-05**	2.27	(1.19- 3.46)	**1.76E-05**	**1.87E-04**	-0.99	(-3.33- 1.40)	4.11E-01	6.75E-01	0.12	(-2.65- 2.98)	9.32E-01	9.75E-01
281	LPE(22:6) [sn1]	2.40	(1.44- 3.44)	**2.49E-07**	**1.14E-06**	2.46	(1.38- 3.64)	**2.68E-06**	**3.78E-05**	-0.35	(-2.65- 2.00)	7.66E-01	9.12E-01	-0.55	(-3.14- 2.11)	6.79E-01	8.31E-01
282	PC(28:0)	0.04	(-0.73- 0.87)	9.20E-01	9.58E-01	-0.63	(-1.55- 0.40)	2.22E-01	4.08E-01	3.28	(-0.56- 7.26)	9.45E-02	2.76E-01	2.22	(-2.23- 6.88)	3.32E-01	5.31E-01
283	PC(14:0_16:0)	0.24	(-0.64- 1.20)	6.04E-01	7.09E-01	-0.67	(-1.66- 0.44)	2.28E-01	4.13E-01	0.90	(-1.55- 3.41)	4.73E-01	7.26E-01	-0.10	(-3.15- 3.05)	9.52E-01	9.75E-01
284	PC(31:0) (a)	0.40	(-0.78- 1.72)	5.25E-01	6.50E-01	-1.56	(-2.77--0.15)	**3.13E-02**	8.83E-02	2.46	(-2.13- 7.26)	2.97E-01	5.68E-01	0.72	(-5.13- 6.93)	8.13E-01	9.17E-01
285	PC(31:0) (b)	-0.86	(-1.93- 0.36)	1.58E-01	2.57E-01	-1.02	(-2.37- 0.58)	1.97E-01	3.76E-01	0.17	(-2.57- 2.99)	9.05E-01	9.65E-01	-0.52	(-4.15- 3.26)	7.84E-01	8.98E-01
286	PC(31:1)	0.49	(-0.57- 1.68)	3.78E-01	5.07E-01	-1.21	(-2.28- 0.02)	5.28E-02	1.38E-01	4.35	(0.93- 7.88)	**1.26E-02**	7.37E-02	2.19	(-2.18- 6.76)	3.28E-01	5.27E-01
287	PC(16:0_16:0)	-0.25	(-1.08- 0.66)	5.73E-01	6.85E-01	-0.92	(-1.86- 0.12)	7.96E-02	1.86E-01	-0.81	(-2.48- 0.89)	3.45E-01	6.22E-01	-1.74	(-3.64- 0.21)	7.91E-02	2.19E-01
288	PC(32:1)	1.65	(0.51- 2.90)	**3.70E-03**	**9.44E-03**	0.08	(-1.10- 1.42)	8.96E-01	9.63E-01	4.07	(1.14- 7.08)	**6.52E-03**	5.30E-02	3.13	(-0.08- 6.44)	5.60E-02	1.79E-01
289	PC(32:2)	0.46	(-0.41- 1.41)	3.04E-01	4.38E-01	-0.31	(-1.30- 0.78)	5.58E-01	7.37E-01	3.33	(0.60- 6.13)	**1.67E-02**	9.22E-02	1.85	(-1.51- 5.33)	2.82E-01	4.80E-01
290	PC(33:0) (a)	0.02	(-1.20- 1.41)	9.76E-01	9.89E-01	-1.71	(-2.86--0.37)	**1.43E-02**	**4.71E-02**	0.13	(-3.32- 3.70)	9.41E-01	9.79E-01	0.08	(-4.35- 4.73)	9.71E-01	9.85E-01
291	PC(33:0) (b)	-1.04	(-1.98- 0.01)	5.31E-02	1.01E-01	-1.91	(-2.99--0.66)	**4.05E-03**	**1.62E-02**	-0.53	(-2.90- 1.90)	6.64E-01	8.54E-01	-1.35	(-4.28- 1.67)	3.76E-01	5.66E-01
292	PC(33:1)	-0.17	(-0.98- 0.72)	7.03E-01	7.75E-01	-1.00	(-1.89--0.02)	**4.68E-02**	1.26E-01	0.38	(-2.33- 3.18)	7.83E-01	9.16E-01	-0.22	(-3.69- 3.37)	9.01E-01	9.70E-01
293	PC(33:2)	-1.09	(-1.89--0.20)	**1.71E-02**	**3.71E-02**	-1.43	(-2.35--0.38)	**8.68E-03**	**3.13E-02**	-0.35	(-2.89- 2.27)	7.91E-01	9.16E-01	-1.50	(-4.70- 1.81)	3.68E-01	5.64E-01
294	PC(16:0_18:0)	-0.19	(-0.92- 0.60)	6.24E-01	7.22E-01	-0.32	(-1.20- 0.64)	4.99E-01	6.87E-01	-1.01	(-2.60- 0.62)	2.22E-01	4.72E-01	-0.37	(-2.29- 1.58)	7.05E-01	8.48E-01
295	PC(16:0_18:1)	0.36	(-0.56- 1.37)	4.58E-01	5.83E-01	-0.22	(-1.25- 0.93)	6.92E-01	8.47E-01	-0.48	(-2.56- 1.65)	6.56E-01	8.54E-01	-0.18	(-2.81- 2.53)	8.96E-01	9.70E-01
296	PC(16:0_18:2)	-0.89	(-1.86- 0.20)	1.05E-01	1.82E-01	-0.07	(-1.40- 1.47)	9.22E-01	9.70E-01	-0.76	(-2.59- 1.11)	4.23E-01	6.81E-01	-1.00	(-2.95- 1.00)	3.22E-01	5.19E-01
297	PC(16:1_18:2)	-0.55	(-1.35- 0.33)	2.14E-01	3.29E-01	-0.99	(-1.94- 0.08)	6.84E-02	1.68E-01	2.00	(-0.20- 4.25)	7.55E-02	2.45E-01	2.02	(-0.64- 4.74)	1.36E-01	3.13E-01
298	PC(16:0_18:3) (a)	0.01	(-1.00- 1.15)	9.78E-01	9.89E-01	0.12	(-1.08- 1.49)	8.48E-01	9.37E-01	0.53	(-1.95- 3.07)	6.78E-01	8.57E-01	1.02	(-2.12- 4.25)	5.26E-01	7.05E-01
299	PC(14:0_20:4)	0.19	(-0.59- 1.04)	6.39E-01	7.32E-01	-0.43	(-1.33- 0.57)	3.85E-01	5.82E-01	4.71	(1.62- 7.89)	**2.76E-03**	**3.89E-02**	5.46	(1.40- 9.68)	**8.28E-03**	6.02E-02
300	PC(34:5)	-0.95	(-2.00- 0.25)	1.16E-01	1.97E-01	-1.30	(-2.48- 0.07)	6.15E-02	1.56E-01	7.08	(2.85-11.48)	**9.79E-04**	**2.14E-02**	8.14	(2.75-13.81)	**2.92E-03**	**3.19E-02**
301	PC(15-MHDA_18:1)	-0.60	(-1.64- 0.56)	2.93E-01	4.26E-01	-1.99	(-3.01--0.83)	**1.43E-03**	**6.39E-03**	-1.31	(-4.61- 2.10)	4.45E-01	6.97E-01	-1.46	(-5.59- 2.85)	4.97E-01	6.83E-01
302	PC(17:0_18:1)	-0.78	(-1.44--0.07)	**3.11E-02**	6.30E-02	-1.36	(-2.10--0.54)	**1.62E-03**	**7.06E-03**	-1.11	(-3.18- 1.00)	2.97E-01	5.68E-01	-1.12	(-3.82- 1.65)	4.21E-01	6.04E-01
303	PC(15-MHDA_18:2)	-0.90	(-1.82- 0.13)	8.58E-02	1.55E-01	-1.96	(-2.93--0.84)	**1.05E-03**	**5.02E-03**	-1.52	(-4.66- 1.73)	3.53E-01	6.25E-01	-1.83	(-5.87- 2.38)	3.86E-01	5.75E-01
304	PC(17:0_18:2)	-1.11	(-1.84--0.32)	**6.91E-03**	**1.65E-02**	-1.46	(-2.33--0.50)	**3.88E-03**	**1.57E-02**	-1.88	(-3.90- 0.18)	7.26E-02	2.45E-01	-1.95	(-4.60- 0.77)	1.56E-01	3.40E-01
305	PC(17:1_18:2)	-0.36	(-1.09- 0.43)	3.57E-01	4.85E-01	-1.03	(-1.85--0.13)	**2.64E-02**	7.72E-02	0.98	(-1.24- 3.25)	3.86E-01	6.55E-01	1.74	(-1.16- 4.73)	2.40E-01	4.32E-01
306	PC(15:0_20:3)	0.05	(-1.17- 1.44)	9.41E-01	9.73E-01	0.07	(-1.47- 1.87)	9.37E-01	9.74E-01	-0.26	(-1.73- 1.23)	7.25E-01	8.86E-01	-0.56	(-2.32- 1.24)	5.38E-01	7.12E-01
307	PC(15:0_20:4)	-0.90	(-1.67--0.06)	**3.60E-02**	7.11E-02	-1.77	(-2.58--0.87)	**2.74E-04**	**1.88E-03**	0.98	(-1.73- 3.77)	4.80E-01	7.29E-01	1.92	(-1.65- 5.63)	2.93E-01	4.90E-01
308	PC(35:5)	-1.91	(-3.02--0.63)	**4.88E-03**	**1.21E-02**	-2.80	(-3.98--1.39)	**3.66E-04**	**2.23E-03**	4.57	(0.35- 8.97)	**3.35E-02**	1.52E-01	4.26	(-1.36-10.19)	1.39E-01	3.14E-01
309	PC(36:0)	0.35	(-0.33- 1.08)	3.17E-01	4.49E-01	0.18	(-0.69- 1.12)	7.01E-01	8.47E-01	0.86	(-1.56- 3.34)	4.88E-01	7.35E-01	2.54	(0.02- 5.12)	**4.82E-02**	1.69E-01
310	PC(18:0_18:1)	0.64	(-0.11- 1.46)	9.55E-02	1.69E-01	0.21	(-0.66- 1.15)	6.48E-01	8.15E-01	1.00	(-0.81- 2.85)	2.78E-01	5.47E-01	1.51	(-1.20- 4.30)	2.75E-01	4.77E-01
311	PC(18:0_18:2)	-0.59	(-1.39- 0.29)	1.81E-01	2.88E-01	-0.31	(-1.31- 0.82)	5.76E-01	7.52E-01	0.44	(-1.52- 2.44)	6.60E-01	8.54E-01	-0.02	(-2.47- 2.49)	9.85E-01	9.91E-01
312	PC(16:0_20:3) (a)	-0.19	(-1.10- 0.81)	7.00E-01	7.74E-01	-0.66	(-1.68- 0.49)	2.49E-01	4.38E-01	-2.82	(-4.70--0.90)	**4.32E-03**	**4.82E-02**	-2.58	(-5.25- 0.16)	6.50E-02	1.92E-01
313	PC(16:0_20:3) (b)	0.42	(-0.38- 1.29)	3.15E-01	4.49E-01	0.31	(-0.70- 1.43)	5.58E-01	7.37E-01	0.96	(-1.17- 3.13)	3.79E-01	6.53E-01	3.32	(0.37- 6.35)	**2.75E-02**	1.19E-01
314	PC(18:2_18:2)	0.50	(-0.69- 1.84)	4.28E-01	5.57E-01	0.15	(-1.24- 1.77)	8.39E-01	9.33E-01	-2.39	(-5.31- 0.62)	1.18E-01	3.26E-01	-3.83	(-7.27--0.26)	**3.57E-02**	1.38E-01
315	PC(16:1_20:4)	0.45	(-0.39- 1.36)	3.02E-01	4.37E-01	-0.40	(-1.33- 0.64)	4.35E-01	6.27E-01	3.02	(0.78- 5.31)	**8.18E-03**	6.04E-02	5.24	(2.20- 8.37)	**7.43E-04**	**1.76E-02**

316	PC(16:0_20:5)	-1.88	(-2.96--0.63)	**4.43E-03**	**1.10E-02**	-1.79	(-3.07--0.27)	**2.27E-02**	6.89E-02	3.68	(-0.38- 7.91)	7.61E-02	2.45E-01	5.20	(0.48-10.14)	**3.08E-02**	1.29E-01
317	PC(36:6)	-0.64	(-1.42- 0.21)	1.35E-01	2.25E-01	-0.93	(-1.85- 0.09)	7.21E-02	1.74E-01	5.11	(1.94- 8.37)	**1.56E-03**	**2.77E-02**	3.99	(-0.02- 8.16)	5.13E-02	1.71E-01
318	PC(15-MHDA_20:4)	-1.12	(-2.00--0.14)	**2.66E-02**	5.51E-02	-2.12	(-3.08--1.03)	**3.59E-04**	**2.21E-03**	-0.66	(-3.70- 2.48)	6.76E-01	8.56E-01	1.62	(-2.39- 5.79)	4.32E-01	6.15E-01
319	PC(17:0_20:4)	-1.63	(-2.29--0.92)	**2.58E-05**	**9.30E-05**	-1.66	(-2.41--0.84)	**1.77E-04**	**1.31E-03**	-0.55	(-2.87- 1.83)	6.45E-01	8.52E-01	0.96	(-2.03- 4.04)	5.30E-01	7.09E-01
320	PC(15:0_22:6)	-1.99	(-2.70--1.21)	**3.93E-06**	**1.55E-05**	-1.58	(-2.48--0.57)	**3.04E-03**	**1.25E-02**	0.74	(-2.10- 3.66)	6.11E-01	8.24E-01	0.52	(-3.08- 4.26)	7.80E-01	8.97E-01
321	PC(38:2)	0.98	(0.23- 1.78)	**9.39E-03**	**2.16E-02**	0.87	(-0.10- 1.93)	7.94E-02	1.86E-01	0.64	(-0.76- 2.06)	3.67E-01	6.37E-01	1.98	(0.01- 3.98)	**4.89E-02**	1.69E-01
322	PC(18:0_20:3)	0.19	(-0.51- 0.93)	6.09E-01	7.13E-01	0.11	(-0.80- 1.11)	8.24E-01	9.22E-01	4.07	(1.92- 6.27)	**2.27E-04**	**9.89E-03**	7.65	(4.41-11.00)	**4.56E-06**	**1.04E-03**
323	PC(18:1_20:3)	0.62	(-0.22- 1.52)	1.51E-01	2.47E-01	-0.17	(-1.07- 0.82)	7.27E-01	8.57E-01	0.17	(-1.75- 2.14)	8.59E-01	9.40E-01	2.37	(-0.35- 5.16)	8.77E-02	2.37E-01
324	PC(38:4) (b)	-0.05	(-0.99- 0.99)	9.22E-01	9.58E-01	-0.06	(-1.18- 1.21)	9.26E-01	9.70E-01	0.85	(-1.51- 3.27)	4.82E-01	7.29E-01	3.49	(0.40- 6.67)	**2.66E-02**	1.19E-01
325	PC(18:0_20:4)	-1.14	(-1.87--0.34)	**5.99E-03**	**1.45E-02**	-0.86	(-1.79- 0.17)	9.85E-02	2.23E-01	1.09	(-1.09- 3.30)	3.28E-01	6.08E-01	3.01	(0.14- 5.97)	**3.98E-02**	1.49E-01
326	PC(38:5) (a)	-1.04	(-1.68--0.35)	**3.90E-03**	**9.89E-03**	-1.25	(-2.06--0.35)	**7.37E-03**	**2.79E-02**	-0.36	(-2.25- 1.57)	7.14E-01	8.85E-01	1.30	(-1.17- 3.83)	3.02E-01	5.02E-01
327	PC(38:5) (b)	0.14	(-0.70- 1.05)	7.52E-01	8.15E-01	0.22	(-0.84- 1.40)	6.95E-01	8.47E-01	1.13	(-1.57- 3.91)	4.14E-01	6.76E-01	2.83	(-0.37- 6.14)	8.28E-02	2.28E-01
328	PC(38:6) (a)	0.03	(-0.91- 1.07)	9.51E-01	9.77E-01	-0.01	(-1.09- 1.19)	9.80E-01	9.90E-01	-0.19	(-2.41- 2.08)	8.66E-01	9.44E-01	1.00	(-2.07- 4.17)	5.25E-01	7.05E-01
329	PC(16:0_22:6)	-1.37	(-2.26--0.37)	**8.36E-03**	**1.96E-02**	-0.95	(-2.03- 0.27)	1.20E-01	2.58E-01	0.50	(-2.15- 3.22)	7.15E-01	8.85E-01	-0.43	(-3.81- 3.07)	8.07E-01	9.17E-01
330	PC(18:2_20:5)	-0.59	(-1.59- 0.53)	2.87E-01	4.18E-01	-0.70	(-1.90- 0.68)	3.05E-01	4.98E-01	2.05	(-1.40- 5.62)	2.46E-01	5.03E-01	3.99	(-0.32- 8.48)	6.95E-02	2.03E-01
331	PC(16:1_22:6)	-0.81	(-1.50--0.06)	**3.54E-02**	7.02E-02	-1.22	(-2.03--0.33)	**8.62E-03**	**3.13E-02**	1.62	(-0.94- 4.23)	2.15E-01	4.66E-01	2.07	(-1.01- 5.25)	1.89E-01	3.72E-01
332	PC(38:7)(c)	-0.92	(-1.54--0.25)	**7.98E-03**	**1.88E-02**	-0.71	(-1.49- 0.14)	1.00E-01	2.25E-01	0.28	(-1.86- 2.47)	7.99E-01	9.16E-01	0.02	(-2.61- 2.71)	9.91E-01	9.91E-01
333	PC(39:5)(a)	-1.75	(-2.57--0.83)	**3.70E-04**	**1.10E-03**	-2.10	(-3.10--0.95)	**7.13E-04**	**3.76E-03**	-0.44	(-3.46- 2.68)	7.80E-01	9.16E-01	-0.32	(-4.36- 3.90)	8.80E-01	9.66E-01
334	PC(39:5)(b)	-1.89	(-2.68--1.01)	**8.04E-05**	**2.72E-04**	-1.82	(-2.76--0.76)	**1.36E-03**	**6.19E-03**	-1.18	(-4.17- 1.89)	4.44E-01	6.97E-01	-0.17	(-3.85- 3.64)	9.28E-01	9.75E-01
335	PC(15-MHDA_22:6)	-1.84	(-2.69--0.89)	**3.31E-04**	**9.86E-04**	-2.26	(-3.27--1.11)	**3.42E-04**	**2.19E-03**	-1.04	(-3.88- 1.88)	4.77E-01	7.28E-01	-1.22	(-5.04- 2.75)	5.38E-01	7.12E-01
336	PC(17:0_22:6)	-2.46	(-3.13--1.72)	**6.30E-09**	**3.64E-08**	-1.74	(-2.64--0.71)	**1.44E-03**	**6.39E-03**	-0.28	(-3.17- 2.70)	8.50E-01	9.40E-01	-0.45	(-4.08- 3.32)	8.12E-01	9.17E-01
337	PC(18:0_22:5) (n6)	-1.73	(-3.03--0.17)	**3.09E-02**	6.28E-02	-0.35	(-2.32- 2.14)	7.63E-01	8.85E-01	1.64	(-0.82- 4.17)	1.92E-01	4.33E-01	3.63	(0.39- 6.98)	**2.81E-02**	1.19E-01
338	PC(18:0_22:6)	-1.78	(-2.56--0.92)	**1.28E-04**	**4.22E-04**	-0.83	(-1.94- 0.44)	1.91E-01	3.68E-01	3.12	(0.46- 5.86)	**2.16E-02**	1.10E-01	3.17	(-0.31- 6.78)	7.42E-02	2.11E-01
339	PC(40:7) (a)	1.11	(-0.16- 2.53)	8.81E-02	1.58E-01	0.82	(-0.56- 2.41)	2.57E-01	4.48E-01	0.56	(-1.86- 3.03)	6.53E-01	8.54E-01	2.78	(-0.68- 6.36)	1.16E-01	2.80E-01
340	PC(18:1_22:6) (a)	-0.29	(-1.18- 0.70)	5.54E-01	6.72E-01	-0.54	(-1.59- 0.63)	3.50E-01	5.53E-01	-1.88	(-4.02- 0.31)	9.10E-02	2.70E-01	-0.98	(-3.53- 1.65)	4.59E-01	6.42E-01
341	PC(40:8)	1.00	(0.16- 1.92)	**1.88E-02**	**3.97E-02**	0.64	(-0.31- 1.69)	1.96E-01	3.76E-01	-1.34	(-3.23- 0.58)	1.69E-01	4.07E-01	-0.28	(-2.77- 2.28)	8.27E-01	9.21E-01
342	PC(44:12)	-1.20	(-2.14--0.13)	**2.87E-02**	5.89E-02	-0.64	(-1.81- 0.70)	3.31E-01	5.28E-01	-0.51	(-4.52- 3.66)	8.05E-01	9.16E-01	-0.87	(-5.51- 4.00)	7.20E-01	8.50E-01
343	PC(O-16:0/16:0)	0.50	(-0.35- 1.42)	2.60E-01	3.86E-01	0.45	(-0.53- 1.54)	3.79E-01	5.75E-01	-0.62	(-2.10- 0.89)	4.16E-01	6.76E-01	-1.46	(-3.27- 0.38)	1.18E-01	2.83E-01
344	PC(O-32:1)	1.20	(0.21- 2.28)	**1.64E-02**	**3.57E-02**	0.22	(-0.88- 1.45)	7.10E-01	8.47E-01	0.19	(-1.85- 2.27)	8.56E-01	9.40E-01	-0.57	(-2.70- 1.59)	5.99E-01	7.62E-01
345	PC(O-34:1)	1.05	(0.18- 2.01)	**1.75E-02**	**3.77E-02**	0.22	(-0.78- 1.33)	6.75E-01	8.33E-01	-1.96	(-3.30--0.60)	**5.04E-03**	**4.82E-02**	-2.29	(-4.00--0.54)	**1.07E-02**	6.90E-02
346	PC(O-34:2)	-0.17	(-1.05- 0.80)	7.20E-01	7.86E-01	0.13	(-1.02- 1.42)	8.35E-01	9.32E-01	-1.11	(-3.41- 1.23)	3.47E-01	6.22E-01	-2.03	(-4.76- 0.79)	1.55E-01	3.40E-01
347	PC(O-34:4)	0.43	(-0.54- 1.50)	3.97E-01	5.27E-01	-0.50	(-1.45- 0.56)	3.42E-01	5.43E-01	0.50	(-3.15- 4.29)	7.91E-01	9.16E-01	3.00	(-1.17- 7.35)	1.60E-01	3.41E-01
348	PC(O-35:4)	-0.23	(-1.06- 0.67)	6.00E-01	7.06E-01	-0.74	(-1.72- 0.35)	1.74E-01	3.43E-01	0.10	(-3.29- 3.62)	9.52E-01	9.83E-01	1.61	(-2.69- 6.09)	4.67E-01	6.50E-01
349	PC(O-36:0)	0.42	(-0.64- 1.60)	4.53E-01	5.79E-01	-0.03	(-1.28- 1.41)	9.70E-01	9.87E-01	-2.10	(-3.83--0.34)	**1.97E-02**	1.05E-01	-2.53	(-4.73--0.28)	**2.79E-02**	1.19E-01
350	PC(O-18:0/18:1)	1.55	(0.63- 2.54)	**7.13E-04**	**2.02E-03**	1.06	(0.01- 2.22)	**4.81E-02**	1.28E-01	-3.34	(-4.86--1.79)	**3.82E-05**	**4.46E-03**	-2.26	(-4.14--0.36)	**2.06E-02**	1.05E-01
351	PC(O-18:1/18:1)	1.04	(0.25- 1.90)	**9.51E-03**	**2.17E-02**	0.26	(-0.66- 1.27)	5.93E-01	7.64E-01	-1.48	(-2.89--0.04)	**4.38E-02**	1.91E-01	-1.92	(-3.64--0.17)	**3.20E-02**	1.31E-01
352	PC(O-18:0/18:2)	0.61	(-0.21- 1.50)	1.51E-01	2.47E-01	0.26	(-0.70- 1.32)	6.13E-01	7.83E-01	-1.98	(-3.91--0.02)	**4.82E-02**	1.98E-01	-2.99	(-5.34--0.59)	**1.54E-02**	8.50E-02
353	PC(O-18:1/18:2)	-0.04	(-0.87- 0.86)	9.25E-01	9.59E-01	-0.07	(-1.09- 1.07)	9.02E-01	9.63E-01	-0.65	(-2.52- 1.25)	4.99E-01	7.37E-01	-1.92	(-4.31- 0.53)	1.23E-01	2.91E-01
354	PC(O-16:0/20:3)	0.28	(-0.58- 1.21)	5.36E-01	6.58E-01	0.66	(-0.46- 1.90)	2.59E-01	4.50E-01	0.68	(-1.19- 2.59)	4.76E-01	7.28E-01	1.95	(-0.44- 4.40)	1.10E-01	2.72E-01
355	PC(O-16:0/20:4)	-0.18	(-0.93- 0.63)	6.49E-01	7.36E-01	-0.04	(-0.94- 0.96)	9.37E-01	9.74E-01	0.58	(-1.38- 2.58)	5.63E-01	7.90E-01	0.96	(-1.34- 3.32)	4.15E-01	6.02E-01
356	PC(O-36:5)	-1.35	(-2.17--0.45)	**4.42E-03**	**1.10E-02**	-0.51	(-1.62- 0.74)	4.05E-01	6.00E-01	2.46	(-1.21- 6.27)	1.90E-01	4.31E-01	3.61	(-0.71- 8.11)	1.02E-01	2.63E-01
357	PC(O-18:0/20:4)	-0.31	(-1.01- 0.46)	4.20E-01	5.53E-01	-0.28	(-1.10- 0.63)	5.35E-01	7.24E-01	-1.24	(-3.20- 0.75)	2.18E-01	4.66E-01	-1.13	(-3.29- 1.07)	3.09E-01	5.08E-01
358	PC(O-38:5)	-0.03	(-0.89- 0.90)	9.43E-01	9.74E-01	-0.24	(-1.30- 0.94)	6.73E-01	8.33E-01	0.43	(-1.39- 2.28)	6.46E-01	8.52E-01	0.07	(-2.04- 2.23)	9.46E-01	9.75E-01
359	PC(O-16:0/22:6)	-1.00	(-1.77--0.16)	**2.02E-02**	**4.22E-02**	-0.42	(-1.37- 0.63)	4.15E-01	6.09E-01	1.60	(-0.89- 4.16)	2.08E-01	4.58E-01	0.86	(-2.08- 3.89)	5.67E-01	7.36E-01
360	PC(O-40:5)	0.01	(-0.67- 0.74)	9.79E-01	9.89E-01	-0.37	(-1.18- 0.51)	4.00E-01	5.96E-01	-0.29	(-2.02- 1.48)	7.47E-01	8.99E-01	-1.20	(-3.28- 0.92)	2.62E-01	4.57E-01
361	PC(O-18:0/22:6)	-1.03	(-1.69--0.32)	**5.47E-03**	**1.35E-02**	-0.70	(-1.51- 0.18)	1.13E-01	2.46E-01	-0.55	(-2.51- 1.44)	5.83E-01	8.05E-01	-2.02	(-4.34- 0.35)	9.42E-02	2.49E-01

362	PC(O-40:7) (a)	-0.64	(-1.39- 0.17)	1.19E-01	2.01E-01	-0.78	(-1.65- 0.19)	1.10E-01	2.42E-01	0.74	(-1.65- 3.18)	5.47E-01	7.80E-01	-0.84	(-3.61- 2.01)	5.55E-01	7.26E-01
363	PC(P-16:0/14:0)	-0.22	(-1.11- 0.76)	6.52E-01	7.36E-01	-0.42	(-1.55- 0.86)	5.01E-01	6.87E-01	0.13	(-2.37- 2.70)	9.18E-01	9.69E-01	0.13	(-2.71- 3.06)	9.28E-01	9.75E-01
364	PC(P-16:0/16:0)	-0.52	(-1.34- 0.38)	2.45E-01	3.66E-01	-0.43	(-1.43- 0.69)	4.40E-01	6.28E-01	-0.86	(-2.63- 0.95)	3.47E-01	6.22E-01	-1.52	(-3.47- 0.47)	1.32E-01	3.06E-01
365	PC(P-16:0/16:1)	0.15	(-0.73- 1.13)	7.42E-01	8.06E-01	-0.21	(-1.26- 0.97)	7.15E-01	8.47E-01	1.14	(-0.90- 3.22)	2.73E-01	5.44E-01	0.76	(-1.48- 3.04)	5.08E-01	6.94E-01
366	PC(P-16:0/18:0)	-0.30	(-0.98- 0.44)	4.20E-01	5.53E-01	-0.05	(-0.89- 0.85)	9.03E-01	9.63E-01	-2.07	(-3.33--0.79)	**1.71E-03**	**2.82E-02**	-1.93	(-3.50--0.32)	**1.93E-02**	1.01E-01
367	PC(P-16:0/18:1)	-0.19	(-0.93- 0.62)	6.41E-01	7.32E-01	-0.32	(-1.24- 0.70)	5.22E-01	7.08E-01	-1.85	(-3.38--0.29)	**2.02E-02**	1.07E-01	-1.85	(-3.75- 0.09)	6.13E-02	1.88E-01
368	PC(P-16:0/18:2)	-1.57	(-2.30--0.77)	**2.57E-04**	**7.96E-04**	-0.83	(-1.84- 0.31)	1.45E-01	3.00E-01	-0.81	(-2.86- 1.28)	4.43E-01	6.97E-01	-1.85	(-4.29- 0.66)	1.47E-01	3.29E-01
369	PC(P-16:0/18:3)	-0.54	(-1.23- 0.21)	1.55E-01	2.53E-01	-0.35	(-1.17- 0.55)	4.32E-01	6.27E-01	0.37	(-1.97- 2.75)	7.60E-01	9.12E-01	-0.76	(-3.42- 1.97)	5.78E-01	7.46E-01
370	PC(P-35:2) (a)	-0.18	(-1.06- 0.78)	6.99E-01	7.74E-01	-0.79	(-1.84- 0.39)	1.81E-01	3.53E-01	-1.40	(-4.65- 1.97)	4.09E-01	6.75E-01	-3.01	(-7.03- 1.18)	1.55E-01	3.40E-01
371	PC(P-35:2) (b)	-1.75	(-2.52--0.90)	**1.33E-04**	**4.34E-04**	-1.04	(-2.03- 0.07)	6.41E-02	1.60E-01	-2.17	(-4.70- 0.42)	9.96E-02	2.86E-01	-2.64	(-5.78- 0.61)	1.10E-01	2.72E-01
372	PC(P-15:0/20:4) (b)	-0.96	(-1.85- 0.03)	5.62E-02	1.07E-01	-0.31	(-1.45- 0.99)	6.27E-01	7.96E-01	0.16	(-2.68- 3.08)	9.11E-01	9.68E-01	1.40	(-1.99- 4.90)	4.21E-01	6.04E-01
373	PC(P-18:1/18:1)	1.43	(0.35- 2.63)	**8.79E-03**	**2.04E-02**	0.97	(-0.31- 2.42)	1.45E-01	3.00E-01	-0.57	(-2.19- 1.08)	4.92E-01	7.35E-01	0.21	(-1.67- 2.13)	8.24E-01	9.21E-01
374	PC(P-18:0/18:2)	-0.60	(-1.28- 0.14)	1.07E-01	1.84E-01	-0.67	(-1.55- 0.30)	1.67E-01	3.35E-01	-2.43	(-4.45--0.37)	**2.12E-02**	1.10E-01	-3.16	(-5.41--0.85)	**7.88E-03**	6.01E-02
375	PC(P-16:0/20:4)	-1.23	(-1.93--0.46)	**2.23E-03**	**5.95E-03**	-0.83	(-1.73- 0.17)	9.99E-02	2.25E-01	0.38	(-1.65- 2.45)	7.13E-01	8.85E-01	0.56	(-1.91- 3.10)	6.57E-01	8.10E-01
376	PC(P-16:0/20:5)	-2.29	(-3.14--1.33)	**1.72E-05**	**6.30E-05**	-1.43	(-2.56--0.13)	**3.29E-02**	9.22E-02	3.50	(-0.16- 7.30)	6.11E-02	2.20E-01	3.19	(-1.51- 8.11)	1.85E-01	3.70E-01
377	PC(P-17:0/20:4) (a)	-1.29	(-2.15--0.32)	**1.04E-02**	**2.36E-02**	-1.79	(-2.78--0.66)	**2.76E-03**	**1.16E-02**	-0.74	(-3.98- 2.60)	6.57E-01	8.54E-01	-0.35	(-4.60- 4.09)	8.75E-01	9.63E-01
378	PC(P-17:0/20:4) (b)	-2.12	(-2.86--1.31)	**2.76E-06**	**1.10E-05**	-1.46	(-2.40--0.41)	**7.72E-03**	**2.83E-02**	-1.20	(-3.85- 1.54)	3.85E-01	6.55E-01	-0.10	(-3.46- 3.38)	9.55E-01	9.75E-01
379	PC(P-18:0/20:4)	-1.18	(-1.84--0.48)	**1.45E-03**	**3.91E-03**	-0.88	(-1.68- 0.01)	5.25E-02	1.38E-01	-0.99	(-3.10- 1.16)	3.62E-01	6.36E-01	-0.03	(-2.62- 2.64)	9.84E-01	9.91E-01
380	PC(P-38:5) (a)	-1.28	(-1.98--0.52)	**1.39E-03**	**3.78E-03**	-1.06	(-1.92--0.10)	**3.11E-02**	8.83E-02	0.64	(-1.40- 2.73)	5.38E-01	7.71E-01	1.49	(-1.04- 4.08)	2.49E-01	4.40E-01
381	PC(P-38:5) (b)	-0.85	(-1.67- 0.04)	6.12E-02	1.15E-01	-0.77	(-1.74- 0.31)	1.54E-01	3.17E-01	-0.39	(-2.60- 1.87)	7.31E-01	8.86E-01	-0.02	(-2.65- 2.68)	9.89E-01	9.91E-01
382	PC(P-16:0/22:6)	-1.66	(-2.39--0.87)	**9.95E-05**	**3.34E-04**	-1.21	(-2.13--0.19)	**2.17E-02**	6.63E-02	0.84	(-1.69- 3.44)	5.18E-01	7.51E-01	-0.10	(-3.19- 3.08)	9.47E-01	9.75E-01
383	PC(P-20:0/20:4)	-1.01	(-1.76--0.19)	**1.72E-02**	**3.71E-02**	-0.60	(-1.54- 0.45)	2.49E-01	4.38E-01	-1.46	(-3.34- 0.46)	1.35E-01	3.48E-01	-2.02	(-4.42- 0.44)	1.06E-01	2.68E-01
384	PC(P-40:5) (b)	-0.93	(-1.59--0.23)	**1.07E-02**	**2.42E-02**	-0.55	(-1.36- 0.35)	2.24E-01	4.09E-01	-1.16	(-3.01- 0.73)	2.27E-01	4.75E-01	-1.23	(-3.35- 0.93)	2.59E-01	4.51E-01
385	PC(P-18:0/22:6)	-1.73	(-2.38--1.03)	**6.54E-06**	**2.50E-05**	-1.24	(-2.06--0.33)	**8.94E-03**	**3.15E-02**	-1.02	(-3.45- 1.47)	4.16E-01	6.76E-01	-2.19	(-5.18- 0.88)	1.59E-01	3.41E-01
386	PE(16:0_16:1)	0.79	(-0.57- 2.35)	2.67E-01	3.95E-01	0.10	(-1.39- 1.84)	9.03E-01	9.63E-01	4.60	(-0.05- 9.48)	5.25E-02	2.08E-01	4.25	(-1.08- 9.86)	1.19E-01	2.83E-01
387	PE(16:0_18:1)	-0.45	(-1.29- 0.47)	3.22E-01	4.53E-01	-0.64	(-1.79- 0.67)	3.23E-01	5.18E-01	0.33	(-2.93- 3.71)	8.43E-01	9.40E-01	0.13	(-4.04- 4.47)	9.54E-01	9.75E-01
388	PE(16:0_18:2)	-1.02	(-1.89--0.05)	**3.98E-02**	7.73E-02	-0.08	(-1.29- 1.29)	8.97E-01	9.63E-01	-0.28	(-3.93- 3.51)	8.82E-01	9.49E-01	-2.10	(-6.28- 2.26)	3.37E-01	5.37E-01
389	PE(16:1_18:2)	0.47	(-0.56- 1.61)	3.84E-01	5.13E-01	0.32	(-0.87- 1.66)	6.12E-01	7.83E-01	0.31	(-2.73- 3.43)	8.45E-01	9.40E-01	-0.52	(-4.03- 3.13)	7.76E-01	8.97E-01
390	PE(16:0_18:3) (a)	0.26	(-0.79- 1.42)	6.46E-01	7.36E-01	-0.14	(-1.30- 1.16)	8.18E-01	9.20E-01	-0.70	(-4.39- 3.13)	7.15E-01	8.85E-01	-0.85	(-5.27- 3.77)	7.11E-01	8.49E-01
391	PE(16:0_18:3) (b)	0.43	(-0.64- 1.64)	4.43E-01	5.70E-01	0.90	(-0.50- 2.52)	2.20E-01	4.08E-01	1.62	(-2.42- 5.83)	4.36E-01	6.92E-01	2.25	(-2.68- 7.43)	3.75E-01	5.66E-01
392	PE(17:0_18:2)	-0.71	(-1.41- 0.04)	6.27E-02	1.17E-01	-1.00	(-1.74--0.20)	**1.57E-02**	5.09E-02	-1.54	(-4.48- 1.50)	3.15E-01	5.90E-01	-0.65	(-4.27- 3.11)	7.31E-01	8.58E-01
393	PE(18:0_18:1)	-0.15	(-1.11- 0.91)	7.66E-01	8.26E-01	0.04	(-1.23- 1.48)	9.57E-01	9.85E-01	2.46	(-0.64- 5.67)	1.21E-01	3.31E-01	2.72	(-1.38- 6.99)	1.95E-01	3.79E-01
394	PE(18:1_18:1)	0.51	(-0.94- 2.19)	5.12E-01	6.35E-01	-0.76	(-2.24- 1.00)	3.73E-01	5.75E-01	0.25	(-2.69- 3.28)	8.68E-01	9.44E-01	-0.29	(-4.08- 3.66)	8.84E-01	9.67E-01
395	PE(18:0_18:2)	-0.99	(-1.81--0.09)	**3.15E-02**	6.35E-02	0.27	(-0.86- 1.55)	6.50E-01	8.15E-01	1.99	(-1.21- 5.30)	2.25E-01	4.75E-01	0.21	(-3.66- 4.24)	9.16E-01	9.75E-01
396	PE(18:1_18:2)	-0.38	(-1.38- 0.74)	4.92E-01	6.17E-01	-0.10	(-1.25- 1.20)	8.73E-01	9.49E-01	-1.86	(-5.14- 1.53)	2.76E-01	5.46E-01	-2.88	(-7.07- 1.50)	1.93E-01	3.76E-01
397	PE(16:0_20:3)	-0.36	(-1.29- 0.67)	4.82E-01	6.07E-01	-0.07	(-1.29- 1.32)	9.17E-01	9.70E-01	0.39	(-2.93- 3.82)	8.20E-01	9.30E-01	-0.99	(-5.22- 3.42)	6.51E-01	8.08E-01
398	PE(16:0_20:4)	-0.38	(-1.16- 0.46)	3.60E-01	4.87E-01	-0.10	(-1.13- 1.06)	8.64E-01	9.47E-01	-0.96	(-4.10- 2.27)	5.53E-01	7.83E-01	-2.43	(-6.13- 1.42)	2.11E-01	4.01E-01
399	PE(16:1_20:4)	0.97	(-0.10- 2.15)	7.71E-02	1.41E-01	0.33	(-0.91- 1.74)	6.20E-01	7.90E-01	1.50	(-1.26- 4.33)	2.88E-01	5.55E-01	0.27	(-2.93- 3.58)	8.69E-01	9.59E-01
400	PE(16:0_20:5)	-0.90	(-1.87- 0.18)	9.99E-02	1.74E-01	-0.71	(-1.91- 0.68)	3.00E-01	4.96E-01	2.40	(-1.13- 6.06)	1.84E-01	4.24E-01	0.83	(-3.33- 5.17)	6.99E-01	8.47E-01
401	PE(17:0_20:4)	-1.83	(-2.56--1.02)	**3.39E-05**	**1.20E-04**	-1.32	(-2.30--0.23)	**1.97E-02**	6.11E-02	-1.06	(-3.85- 1.81)	4.64E-01	7.18E-01	-1.91	(-5.34- 1.64)	2.84E-01	4.81E-01
402	PE(18:0_20:3) (a)	-0.67	(-1.50- 0.24)	1.45E-01	2.40E-01	-0.43	(-1.44- 0.71)	4.44E-01	6.30E-01	3.27	(0.31- 6.33)	**3.03E-02**	1.43E-01	4.21	(0.20- 8.38)	**3.97E-02**	1.49E-01
403	PE(18:0_20:4)	-0.88	(-1.60--0.10)	**2.71E-02**	5.58E-02	-0.29	(-1.29- 0.83)	5.94E-01	7.64E-01	0.78	(-1.83- 3.47)	5.60E-01	7.88E-01	0.12	(-3.17- 3.52)	9.45E-01	9.75E-01
404	PE(38:5) (a)	-0.56	(-1.35- 0.31)	1.98E-01	3.10E-01	-0.58	(-1.59- 0.56)	3.03E-01	4.98E-01	-1.44	(-4.36- 1.57)	3.43E-01	6.22E-01	-1.69	(-5.42- 2.19)	3.87E-01	5.75E-01
405	PE(38:5) (b)	0.37	(-0.91- 1.83)	5.91E-01	7.01E-01	0.64	(-1.02- 2.60)	4.71E-01	6.59E-01	1.89	(-1.70- 5.62)	3.04E-01	5.75E-01	-0.63	(-5.08- 4.03)	7.87E-01	8.98E-01
406	PE(16:0_22:6)	-1.91	(-2.69--1.05)	**4.47E-05**	**1.55E-04**	-0.94	(-2.05- 0.32)	1.36E-01	2.85E-01	0.66	(-3.01- 4.47)	7.26E-01	8.86E-01	-2.20	(-6.35- 2.14)	3.13E-01	5.12E-01
407	PE(17:0_22:6)	-1.98	(-2.73--1.15)	**1.42E-05**	**5.32E-05**	-0.85	(-1.77- 0.18)	1.03E-01	2.28E-01	0.36	(-2.40- 3.19)	8.02E-01	9.16E-01	-0.67	(-4.13- 2.91)	7.08E-01	8.49E-01

408	PE(18:0_22:4)	-0.28	(-1.26- 0.80)	5.95E-01	7.04E-01	0.09	(-1.24- 1.62)	8.99E-01	9.63E-01	2.66	(-0.04- 5.42)	5.34E-02	2.10E-01	1.85	(-1.70- 5.52)	3.08E-01	5.08E-01
409	PE(18:0_22:5) (n3)	-1.83	(-2.72--0.83)	**6.62E-04**	**1.93E-03**	0.21	(-1.17- 1.82)	7.76E-01	8.91E-01	2.74	(-0.41- 5.99)	8.81E-02	2.66E-01	2.13	(-1.43- 5.82)	2.42E-01	4.32E-01
410	PE(18:0_22:5) (n6)	-0.53	(-1.72- 0.84)	4.28E-01	5.57E-01	-0.19	(-1.72- 1.62)	8.24E-01	9.22E-01	2.97	(-0.29- 6.33)	7.46E-02	2.45E-01	2.09	(-2.17- 6.54)	3.39E-01	5.39E-01
411	PE(18:0_22:6)	-2.15	(-3.01--1.18)	**5.23E-05**	**1.81E-04**	-0.65	(-1.96- 0.87)	3.78E-01	5.75E-01	5.09	(1.50- 8.81)	**5.41E-03**	**4.82E-02**	1.93	(-2.24- 6.29)	3.66E-01	5.64E-01
412	PE(40:7)	-1.27	(-2.08--0.38)	**6.54E-03**	**1.58E-02**	-1.18	(-2.25- 0.04)	5.83E-02	1.51E-01	-0.15	(-3.95- 3.80)	9.40E-01	9.79E-01	-2.69	(-6.91- 1.73)	2.28E-01	4.25E-01
413	PE(O-34:1)	0.79	(-0.74- 2.57)	3.30E-01	4.63E-01	-0.03	(-1.61- 1.85)	9.72E-01	9.87E-01	-1.06	(-3.39- 1.34)	3.81E-01	6.53E-01	-1.66	(-4.16- 0.90)	2.00E-01	3.86E-01
414	PE(O-16:0/18:2)	0.71	(-1.89- 4.14)	6.29E-01	7.25E-01	-1.25	(-3.52- 1.82)	3.82E-01	5.79E-01	0.01	(-2.86- 2.98)	9.92E-01	9.99E-01	-0.32	(-3.72- 3.21)	8.56E-01	9.46E-01
415	PE(O-18:1/18:2)	0.01	(-1.19- 1.38)	9.88E-01	9.94E-01	0.40	(-1.06- 2.09)	6.10E-01	7.83E-01	-0.25	(-2.78- 2.35)	8.50E-01	9.40E-01	-0.08	(-3.36- 3.31)	9.61E-01	9.77E-01
416	PE(O-16:0/20:4)	-0.90	(-1.91- 0.22)	1.11E-01	1.90E-01	-0.59	(-1.88- 0.90)	4.14E-01	6.09E-01	0.96	(-1.80- 3.80)	4.96E-01	7.35E-01	1.90	(-1.53- 5.44)	2.79E-01	4.80E-01
417	PE(O-36:5)	0.01	(-0.91- 1.01)	9.92E-01	9.94E-01	-0.85	(-1.81- 0.21)	1.12E-01	2.46E-01	2.66	(-0.91- 6.35)	1.45E-01	3.62E-01	2.08	(-2.32- 6.67)	3.58E-01	5.55E-01
418	PE(O-16:0/22:4)	0.38	(-0.55- 1.39)	4.38E-01	5.67E-01	0.93	(-0.28- 2.29)	1.38E-01	2.88E-01	0.12	(-2.09- 2.37)	9.17E-01	9.69E-01	1.03	(-1.48- 3.61)	4.21E-01	6.04E-01
419	PE(O-18:0/20:4)	-0.07	(-1.13- 1.12)	9.01E-01	9.42E-01	-0.18	(-1.33- 1.12)	7.72E-01	8.89E-01	0.55	(-2.54- 3.75)	7.29E-01	8.86E-01	0.47	(-3.42- 4.52)	8.14E-01	9.17E-01
420	PE(O-38:5) (a)	-0.22	(-1.04- 0.68)	6.21E-01	7.22E-01	0.18	(-0.97- 1.48)	7.67E-01	8.85E-01	1.87	(-1.06- 4.88)	2.11E-01	4.61E-01	1.50	(-2.01- 5.14)	4.05E-01	5.96E-01
421	PE(O-38:5) (b)	0.13	(-1.02- 1.42)	8.35E-01	8.90E-01	0.33	(-1.14- 2.03)	6.80E-01	8.36E-01	0.01	(-2.45- 2.53)	9.96E-01	9.99E-01	1.01	(-2.04- 4.14)	5.18E-01	6.99E-01
422	PE(O-16:0/22:6)	0.42	(-0.58- 1.53)	4.23E-01	5.55E-01	0.75	(-0.45- 2.09)	2.29E-01	4.13E-01	0.63	(-1.70- 3.02)	5.96E-01	8.10E-01	1.25	(-1.57- 4.14)	3.87E-01	5.75E-01
423	PE(O-18:0/22:6)	0.07	(-0.76- 0.97)	8.76E-01	9.20E-01	-0.08	(-1.02- 0.96)	8.72E-01	9.49E-01	-0.06	(-2.41- 2.36)	9.63E-01	9.89E-01	0.11	(-2.69- 2.99)	9.37E-01	9.75E-01
424	PE(O-18:1/22:6)	0.14	(-0.60- 0.95)	7.11E-01	7.81E-01	-0.02	(-0.89- 0.93)	9.60E-01	9.85E-01	0.35	(-2.00- 2.75)	7.74E-01	9.15E-01	0.88	(-2.07- 3.92)	5.59E-01	7.30E-01
425	PE(P-16:0/18:1)	1.66	(0.80- 2.59)	**1.05E-04**	**3.49E-04**	1.93	(0.75- 3.24)	**9.42E-04**	**4.66E-03**	0.01	(-1.84- 1.90)	9.89E-01	9.99E-01	0.14	(-2.05- 2.39)	8.99E-01	9.70E-01
426	PE(P-16:0/18:2)	-0.25	(-1.28- 0.91)	6.61E-01	7.44E-01	1.02	(-0.50- 2.78)	1.98E-01	3.76E-01	0.74	(-1.96- 3.52)	5.93E-01	8.10E-01	0.95	(-1.86- 3.85)	5.09E-01	6.94E-01
427	PE(P-16:0/20:3)	0.50	(-0.57- 1.70)	3.73E-01	5.01E-01	-0.19	(-1.48- 1.30)	7.89E-01	9.01E-01	3.59	(1.12- 6.12)	**4.43E-03**	**4.82E-02**	5.78	(2.90- 8.75)	**9.29E-05**	**5.07E-03**
428	PE(P-16:0/20:4)	-0.26	(-1.20- 0.79)	6.16E-01	7.18E-01	0.05	(-1.18- 1.44)	9.44E-01	9.77E-01	3.59	(0.88- 6.37)	**9.38E-03**	6.43E-02	3.85	(0.58- 7.23)	**2.10E-02**	1.05E-01
429	PE(P-16:0/20:5)	-1.60	(-3.57- 0.99)	2.03E-01	3.16E-01	0.39	(-2.49- 4.38)	8.17E-01	9.20E-01	4.73	(0.09- 9.58)	**4.55E-02**	1.95E-01	6.37	(0.53-12.55)	**3.23E-02**	1.31E-01
430	PE(P-16:0/22:4)	0.00	(-0.85- 0.92)	9.94E-01	9.94E-01	0.64	(-0.55- 1.99)	3.04E-01	4.98E-01	-0.42	(-2.25- 1.44)	6.51E-01	8.54E-01	-0.13	(-2.28- 2.06)	9.06E-01	9.71E-01
431	PE(P-16:0/22:5) (n3)	-0.80	(-1.66- 0.16)	1.00E-01	1.74E-01	0.02	(-1.17- 1.37)	9.77E-01	9.89E-01	1.33	(-1.08- 3.79)	2.80E-01	5.49E-01	1.88	(-0.66- 4.48)	1.48E-01	3.30E-01
432	PE(P-16:0/22:5) (n6)	0.83	(-0.20- 1.97)	1.17E-01	1.98E-01	1.20	(-0.11- 2.67)	7.31E-02	1.75E-01	0.48	(-1.92- 2.93)	6.99E-01	8.78E-01	0.38	(-2.79- 3.64)	8.17E-01	9.18E-01
433	PE(P-16:0/22:6)	0.10	(-0.76- 1.03)	8.33E-01	8.90E-01	0.33	(-0.73- 1.51)	5.59E-01	7.37E-01	1.78	(-0.26- 3.87)	8.76E-02	2.66E-01	2.09	(-0.51- 4.76)	1.16E-01	2.80E-01
434	PE(P-17:0/20:4) (a)	0.00	(-0.79- 0.85)	9.93E-01	9.94E-01	-0.81	(-1.69- 0.16)	9.79E-02	2.23E-01	1.04	(-1.91- 4.07)	4.92E-01	7.35E-01	2.47	(-1.39- 6.47)	2.11E-01	4.01E-01
435	PE(P-17:0/20:4) (b)	-0.88	(-1.67--0.01)	**4.77E-02**	9.16E-02	-0.93	(-1.87- 0.13)	8.27E-02	1.92E-01	0.60	(-2.14- 3.41)	6.71E-01	8.54E-01	2.56	(-0.82- 6.05)	1.38E-01	3.14E-01
436	PE(P-17:0/22:6) (a)	0.21	(-0.82- 1.35)	7.04E-01	7.75E-01	-0.67	(-1.78- 0.59)	2.81E-01	4.75E-01	-0.06	(-2.93- 2.91)	9.70E-01	9.93E-01	0.58	(-3.42- 4.74)	7.79E-01	8.97E-01
437	PE(P-17:0/22:6) (b)	-0.45	(-1.31- 0.49)	3.35E-01	4.66E-01	-0.44	(-1.45- 0.70)	4.35E-01	6.27E-01	-0.78	(-3.27- 1.78)	5.45E-01	7.79E-01	0.75	(-2.50- 4.11)	6.53E-01	8.08E-01
438	PE(P-18:0/18:1)	1.12	(0.18- 2.15)	**1.87E-02**	**3.97E-02**	0.90	(-0.38- 2.35)	1.74E-01	3.43E-01	-0.49	(-2.61- 1.68)	6.54E-01	8.54E-01	-0.45	(-2.80- 1.96)	7.13E-01	8.49E-01
439	PE(P-18:0/18:2)	-0.21	(-1.33- 1.07)	7.39E-01	8.04E-01	0.01	(-1.27- 1.48)	9.87E-01	9.94E-01	0.06	(-2.65- 2.85)	9.65E-01	9.90E-01	-1.23	(-3.78- 1.38)	3.49E-01	5.44E-01
440	PE(P-18:0/20:3)	-0.23	(-0.93- 0.53)	5.41E-01	6.61E-01	-0.36	(-1.26- 0.65)	4.71E-01	6.59E-01	2.17	(0.07- 4.32)	**4.30E-02**	1.90E-01	2.87	(0.42- 5.38)	**2.15E-02**	1.06E-01
441	PE(P-18:0/20:4)	-0.94	(-1.74--0.05)	**3.86E-02**	7.57E-02	-0.04	(-1.20- 1.26)	9.44E-01	9.77E-01	1.77	(-0.98- 4.60)	2.08E-01	4.58E-01	1.93	(-1.25- 5.22)	2.34E-01	4.31E-01
442	PE(P-18:0/20:5)	-1.78	(-3.49- 0.38)	9.86E-02	1.73E-01	-2.61	(-4.50--0.08)	**4.40E-02**	1.19E-01	3.74	(-0.66- 8.34)	9.63E-02	2.80E-01	4.66	(-1.03-10.67)	1.10E-01	2.72E-01
443	PE(P-18:0/22:4)	-0.27	(-1.20- 0.77)	5.97E-01	7.05E-01	-0.11	(-1.36- 1.32)	8.71E-01	9.49E-01	-2.03	(-3.90--0.13)	**3.69E-02**	1.64E-01	-1.02	(-3.10- 1.11)	3.44E-01	5.41E-01
444	PE(P-18:0/22:5) (n3)	-0.49	(-1.25- 0.33)	2.35E-01	3.53E-01	-0.16	(-1.14- 0.93)	7.63E-01	8.85E-01	0.33	(-1.82- 2.53)	7.64E-01	9.12E-01	1.53	(-0.94- 4.05)	2.25E-01	4.23E-01
445	PE(P-18:0/22:5) (n6)	0.64	(-0.18- 1.54)	1.30E-01	2.18E-01	0.04	(-0.93- 1.12)	9.35E-01	9.74E-01	-0.23	(-2.75- 2.35)	8.59E-01	9.40E-01	-0.79	(-3.81- 2.33)	6.15E-01	7.74E-01
446	PE(P-18:0/22:6)	-0.15	(-0.92- 0.69)	7.20E-01	7.86E-01	0.22	(-0.84- 1.40)	7.02E-01	8.47E-01	0.34	(-1.95- 2.69)	7.70E-01	9.12E-01	-0.12	(-3.02- 2.87)	9.37E-01	9.75E-01
447	PE(P-18:1/18:1)	0.19	(-0.59- 1.03)	6.40E-01	7.32E-01	0.64	(-0.37- 1.75)	2.22E-01	4.08E-01	-0.33	(-2.23- 1.61)	7.35E-01	8.86E-01	-0.08	(-2.25- 2.14)	9.45E-01	9.75E-01
448	PE(P-18:1/18:2)	-0.56	(-2.03- 1.17)	4.98E-01	6.23E-01	-0.93	(-2.49- 0.94)	3.05E-01	4.98E-01	0.13	(-2.62- 2.96)	9.25E-01	9.71E-01	-1.32	(-4.23- 1.68)	3.83E-01	5.75E-01
449	PE(P-18:1/20:4)	-0.39	(-1.23- 0.53)	3.93E-01	5.24E-01	-0.65	(-1.76- 0.61)	2.95E-01	4.89E-01	2.07	(-0.63- 4.85)	1.33E-01	3.48E-01	2.43	(-0.92- 5.90)	1.56E-01	3.40E-01
450	PE(P-18:1/20:5)	-0.76	(-3.25- 2.65)	6.22E-01	7.22E-01	-0.79	(-3.72- 3.50)	6.71E-01	8.33E-01	3.61	(-1.12- 8.58)	1.36E-01	3.49E-01	5.56	(-0.22-11.68)	5.93E-02	1.87E-01
451	PE(P-18:1/22:4)	0.36	(-0.76- 1.62)	5.42E-01	6.61E-01	0.50	(-0.91- 2.14)	5.05E-01	6.90E-01	-1.12	(-3.01- 0.81)	2.51E-01	5.11E-01	-0.94	(-3.14- 1.31)	4.08E-01	5.97E-01
452	PE(P-18:1/22:5) (a)	-0.78	(-1.53- 0.04)	6.26E-02	1.17E-01	0.34	(-0.73- 1.54)	5.51E-01	7.36E-01	0.91	(-1.26- 3.13)	4.11E-01	6.75E-01	1.77	(-1.11- 4.73)	2.30E-01	4.27E-01
453	PE(P-18:1/22:6) (a)	-0.06	(-0.93- 0.90)	9.03E-01	9.42E-01	-0.06	(-1.11- 1.12)	9.21E-01	9.70E-01	1.17	(-1.22- 3.62)	3.36E-01	6.14E-01	0.93	(-2.13- 4.09)	5.52E-01	7.24E-01

454	PE(P-20:0/18:1)	0.93	(-1.24- 3.64)	4.28E-01	5.57E-01	0.11	(-2.22- 3.14)	9.36E-01	9.74E-01	-0.45	(-2.94- 2.10)	7.24E-01	8.86E-01	-2.01	(-4.37- 0.42)	1.03E-01	2.64E-01
455	PE(P-20:0/18:2)	0.18	(-0.40- 0.80)	5.41E-01	6.61E-01	-0.35	(-0.99- 0.34)	3.14E-01	5.08E-01	-2.04	(-4.27- 0.24)	7.90E-02	2.50E-01	-3.60	(-5.89--1.25)	**3.06E-03**	**3.26E-02**
456	PE(P-20:0/20:4)	-0.37	(-1.48- 0.88)	5.46E-01	6.63E-01	-0.76	(-2.09- 0.78)	3.14E-01	5.08E-01	0.02	(-2.11- 2.19)	9.85E-01	9.99E-01	-0.42	(-2.89- 2.11)	7.43E-01	8.69E-01
457	PE(P-20:0/22:6)	-0.58	(-1.31- 0.21)	1.46E-01	2.41E-01	-0.75	(-1.60- 0.19)	1.14E-01	2.46E-01	-0.94	(-2.82- 0.98)	3.33E-01	6.14E-01	-2.82	(-5.23--0.34)	**2.62E-02**	1.18E-01
458	PE(P-20:1/20:4)	-0.65	(-1.86- 0.75)	3.44E-01	4.74E-01	-0.72	(-2.42- 1.35)	4.65E-01	6.55E-01	1.61	(-1.14- 4.43)	2.53E-01	5.13E-01	0.42	(-3.19- 4.17)	8.21E-01	9.21E-01
459	PG(34:2)	-1.94	(-3.27--0.34)	**2.00E-02**	**4.21E-02**	1.64	(-0.60- 4.42)	1.64E-01	3.31E-01	1.09	(-2.53- 4.83)	5.59E-01	7.88E-01	3.09	(-1.33- 7.70)	1.72E-01	3.57E-01
460	PG(36:1)	-1.48	(-2.82- 0.11)	6.66E-02	1.23E-01	-1.21	(-3.06- 1.12)	2.82E-01	4.75E-01	1.93	(-1.44- 5.41)	2.63E-01	5.28E-01	3.92	(-0.38- 8.40)	7.44E-02	2.11E-01
461	PI(16:0/16:0)	-0.39	(-1.45- 0.82)	5.12E-01	6.35E-01	0.49	(-1.14- 2.43)	5.76E-01	7.52E-01	3.35	(-0.03- 6.83)	5.17E-02	2.08E-01	2.54	(-1.69- 6.94)	2.42E-01	4.32E-01
462	PI(16:0_16:1)	0.67	(-0.36- 1.82)	2.09E-01	3.23E-01	0.86	(-0.50- 2.41)	2.25E-01	4.09E-01	8.10	(4.43-11.90)	**1.52E-05**	**2.43E-03**	9.16	(4.55-13.96)	**9.51E-05**	**5.07E-03**
463	PI(34:0)	-1.69	(-3.19- 0.12)	6.60E-02	1.23E-01	-2.02	(-3.70- 0.11)	6.18E-02	1.56E-01	1.71	(-2.41- 5.99)	4.19E-01	6.78E-01	-2.77	(-7.20- 1.87)	2.36E-01	4.31E-01
464	PI(34:1)	-0.15	(-0.92- 0.69)	7.20E-01	7.86E-01	-0.23	(-1.24- 0.89)	6.73E-01	8.33E-01	0.34	(-2.02- 2.76)	7.78E-01	9.16E-01	0.56	(-2.48- 3.69)	7.21E-01	8.50E-01
465	PI(17:0_18:1)	-1.02	(-2.19- 0.33)	1.31E-01	2.18E-01	-1.70	(-3.22- 0.15)	6.97E-02	1.71E-01	-1.17	(-3.90- 1.63)	4.06E-01	6.75E-01	-0.41	(-4.02- 3.34)	8.27E-01	9.21E-01
466	PI(17:0_18:2)	-2.29	(-3.38--1.03)	**8.51E-04**	**2.37E-03**	-1.03	(-2.76- 1.10)	3.16E-01	5.08E-01	0.58	(-2.09- 3.34)	6.71E-01	8.54E-01	-0.59	(-3.65- 2.57)	7.09E-01	8.49E-01
467	PI(18:0_18:1)	-1.36	(-2.17--0.46)	**4.04E-03**	**1.02E-02**	-0.99	(-2.08- 0.24)	1.10E-01	2.42E-01	-2.06	(-4.76- 0.71)	1.43E-01	3.60E-01	-1.79	(-5.23- 1.78)	3.19E-01	5.17E-01
468	PI(36:2)	-1.48	(-2.19--0.71)	**3.09E-04**	**9.28E-04**	-0.79	(-1.70- 0.23)	1.26E-01	2.69E-01	-1.43	(-3.58- 0.77)	2.01E-01	4.48E-01	-1.68	(-4.10- 0.80)	1.81E-01	3.68E-01
469	PI(18:1_18:2)	-1.40	(-2.07--0.67)	**2.93E-04**	**8.89E-04**	-1.51	(-2.29--0.65)	**9.89E-04**	**4.85E-03**	-3.25	(-5.48--0.96)	**5.81E-03**	**4.98E-02**	-3.22	(-6.17--0.17)	**3.86E-02**	1.47E-01
470	PI(16:0_20:3) (a)	0.08	(-0.59- 0.78)	8.27E-01	8.88E-01	0.57	(-0.33- 1.56)	2.20E-01	4.08E-01	-0.18	(-2.18- 1.86)	8.60E-01	9.40E-01	0.42	(-2.53- 3.46)	7.81E-01	8.97E-01
471	PI(16:0_20:3) (b)	3.31	(1.88- 4.92)	**1.16E-06**	**4.74E-06**	2.73	(1.18- 4.48)	**2.82E-04**	**1.88E-03**	-0.43	(-2.81- 2.02)	7.29E-01	8.86E-01	2.82	(-0.48- 6.22)	9.39E-02	2.49E-01
472	PI(16:0_20:4)	-0.09	(-0.75- 0.63)	8.04E-01	8.65E-01	0.52	(-0.40- 1.51)	2.77E-01	4.73E-01	0.95	(-1.10- 3.03)	3.65E-01	6.37E-01	2.24	(-0.68- 5.23)	1.32E-01	3.06E-01
473	PI(18:0_20:2)	3.06	(1.34- 5.04)	**2.33E-04**	**7.25E-04**	1.63	(-0.16- 3.76)	7.72E-02	1.82E-01	-3.21	(-5.66--0.70)	**1.28E-02**	7.41E-02	-1.07	(-4.23- 2.20)	5.15E-01	6.98E-01
474	PI(18:0_20:3) (a)	-0.40	(-0.97- 0.20)	1.87E-01	2.95E-01	0.00	(-0.81- 0.87)	9.93E-01	9.97E-01	-1.35	(-3.12- 0.45)	1.39E-01	3.52E-01	-0.37	(-2.42- 1.73)	7.28E-01	8.57E-01
475	PI(18:0_20:4)	-0.73	(-1.25--0.17)	**1.08E-02**	**2.42E-02**	-0.31	(-1.02- 0.47)	4.28E-01	6.22E-01	-1.81	(-3.59- 0.00)	5.03E-02	2.05E-01	0.04	(-2.13- 2.25)	9.73E-01	9.85E-01
476	PI(38:5) (a)	-0.06	(-0.87- 0.82)	8.84E-01	9.27E-01	-1.22	(-2.02--0.34)	**7.69E-03**	**2.83E-02**	-1.78	(-3.60- 0.08)	6.07E-02	2.20E-01	0.38	(-2.04- 2.86)	7.58E-01	8.85E-01
477	PI(38:6)	-1.14	(-1.80--0.42)	**2.42E-03**	**6.43E-03**	-0.49	(-1.33- 0.44)	2.92E-01	4.89E-01	0.21	(-1.99- 2.46)	8.54E-01	9.40E-01	-0.66	(-3.33- 2.08)	6.31E-01	7.89E-01
478	PI(18:0_22:4)	1.38	(0.50- 2.34)	**1.81E-03**	**4.87E-03**	1.01	(-0.05- 2.18)	6.34E-02	1.59E-01	-0.76	(-2.93- 1.45)	4.94E-01	7.35E-01	-0.75	(-3.46- 2.04)	5.93E-01	7.59E-01
479	PI(18:0_22:5) (n3)	-1.48	(-2.27--0.61)	**1.28E-03**	**3.52E-03**	-0.73	(-1.79- 0.47)	2.22E-01	4.08E-01	0.11	(-2.14- 2.41)	9.25E-01	9.71E-01	1.75	(-1.21- 4.81)	2.48E-01	4.40E-01
480	PI(18:0_22:6)	-1.27	(-1.93--0.56)	**6.94E-04**	**2.00E-03**	-0.74	(-1.60- 0.21)	1.22E-01	2.61E-01	0.19	(-2.12- 2.56)	8.70E-01	9.45E-01	-0.59	(-3.61- 2.52)	7.04E-01	8.48E-01

Table S5. Association studies with pre-pregnancy BMI and birth weight in Barwon Infant Study (BIS) and the meta-analysis results (GUSTO and BIS). Related to Figure 4.

	**BIS - ppBMI (antenatal)** adjusted for ethnicity, maternal age, maternal education level and gestational weight gain					**Meta Analysis of ppBMI study (GUSTO and BIS)**				**BIS - Birth Weight (cord blood)** adjusted for sex, ethnicity, maternal age, maternal education level, pre-pregnancy BMI, gestational age and parity					**Meta Analysis of BW study (GUSTO and BIS)**			
Lipid Species	%change in lipid concentration per unit BMI	95%CI	p-value	p-value(BH)	Comparison between GUSTO and BIS	Overall effect size (%change)	Overall 95%CI	Overall p-value	Overall p-value(BH)	%change in lipid concentration per 100 grams	95%CI	p-value	p-value(BH)	Comparison between GUSTO and BIS	Overall Effect Size (%change)	Overall 95%CI	Overall p-value	Overall p-value(BH)
AC(12:0)	3.36	(1.75-4.99)	**4.02E-05**	**2.06E-04**	BIS only	1.23	(0.34-2.11)	**6.89E-03**	**1.11E-02**	-1.93	(-2.73--1.13)	**2.89E-06**	**1.63E-05**	GUSTO and BIS	-1.69	(-2.17--1.21)	**6.51E-12**	**2.70E-11**
AC(13:0)	1.81	(0.09-3.56)	**3.97E-02**	7.21E-02	notSig	1.51	(0.45-2.57)	**5.44E-03**	**9.06E-03**	-1.04	(-1.89--0.2)	**1.59E-02**	**3.46E-02**	BIS only	-1.09	(-1.76--0.41)	**1.66E-03**	**3.23E-03**
AC(14:0)	1.10	(0.07-2.14)	**3.63E-02**	6.69E-02	notSig	0.57	(-0.01-1.15)	5.28E-02	7.22E-02	-1.47	(-2.33--0.61)	**8.38E-04**	**2.64E-03**	GUSTO and BIS	-1.17	(-1.65--0.68)	**2.12E-06**	**5.68E-06**
AC(14:1)	5.56	(3.78-7.37)	**7.46E-10**	**2.16E-08**	GUSTO and BIS	2.62	(1.64-3.6)	**1.57E-07**	**6.30E-07**	-1.48	(-2.34--0.63)	**7.02E-04**	**2.25E-03**	GUSTO and BIS	-1.23	(-1.77--0.7)	**5.89E-06**	**1.52E-05**
AC(14:2)	4.48	(2.74-6.25)	**3.73E-07**	**3.93E-06**	BIS only	2.02	(1.08-2.96)	**2.51E-05**	**6.69E-05**	-1.38	(-2.05--0.71)	**5.56E-05**	**2.26E-04**	GUSTO and BIS	-1.13	(-1.56--0.69)	**3.41E-07**	**9.70E-07**
AC(16:0)	1.08	(0.31-1.85)	**6.12E-03**	**1.49E-02**	BIS only	0.56	(0.13-0.99)	**1.07E-02**	**1.66E-02**	-0.36	(-0.98-0.27)	2.63E-01	3.63E-01	notSig	-0.42	(-0.83--0.01)	**4.43E-02**	6.64E-02
AC(16:1)	3.86	(2.73-5)	**2.01E-11**	**1.87E-09**	GUSTO and BIS	2.12	(1.45-2.78)	**5.13E-10**	**2.94E-09**	-1.29	(-2.29--0.3)	**1.08E-02**	**2.50E-02**	GUSTO and BIS	-1.09	(-1.65--0.54)	**1.08E-04**	**2.49E-04**
AC(18:0)	-0.02	(-0.89-0.85)	9.57E-01	9.66E-01	notSig	-0.06	(-0.51-0.39)	7.85E-01	8.17E-01	-0.32	(-1.03-0.39)	3.76E-01	4.82E-01	GUSTO only	-0.62	(-1.04--0.19)	**4.54E-03**	**8.40E-03**
AC(18:1)	2.21	(1.23-3.21)	**1.07E-05**	**6.72E-05**	GUSTO and BIS	1.64	(1.07-2.22)	**1.74E-08**	**7.98E-08**	-0.02	(-0.96-0.92)	9.62E-01	9.66E-01	notSig	0.05	(-0.51-0.62)	8.52E-01	8.86E-01
AC(18:2)	-0.10	(-0.96-0.78)	8.30E-01	8.71E-01	GUSTO only	0.58	(0.04-1.11)	**3.55E-02**	**4.97E-02**	-0.86	(-1.83-0.12)	8.58E-02	1.42E-01	notSig	-0.55	(-1.09--0.02)	**4.16E-02**	6.31E-02
CE(14:0)	-3.91	(-4.95--2.86)	**1.52E-12**	**3.69E-10**	GUSTO and BIS	-2.36	(-2.84--1.87)	**2.35E-21**	**9.90E-20**	0.46	(-0.09-1.02)	9.98E-02	1.62E-01	notSig	-0.04	(-0.42-0.35)	8.54E-01	8.86E-01
CE(16:1)	-1.32	(-2.52--0.11)	**3.33E-02**	6.31E-02	GUSTO only	-1.13	(-1.78--0.49)	**6.00E-04**	**1.16E-03**	2.15	(1.5-2.81)	**2.31E-10**	**2.75E-09**	GUSTO and BIS	1.59	(1.12-2.07)	**5.07E-11**	**1.88E-10**
CE(18:0)	-0.68	(-1.39-0.05)	6.63E-02	1.13E-01	GUSTO only	-0.97	(-1.39--0.56)	**4.91E-06**	**1.44E-05**	1.13	(0.4-1.85)	**2.42E-03**	**6.75E-03**	BIS only	0.74	(0.2-1.28)	**7.07E-03**	**1.25E-02**
CE(18:1)	-0.17	(-0.61-0.28)	4.52E-01	5.34E-01	GUSTO only	-0.68	(-0.93--0.43)	**1.48E-07**	**6.13E-07**	0.94	(0.41-1.46)	**4.81E-04**	**1.60E-03**	BIS only	0.48	(0.14-0.81)	**5.70E-03**	**1.03E-02**
CE(18:2)	-0.53	(-0.92--0.15)	**6.72E-03**	**1.62E-02**	GUSTO and BIS	-0.70	(-0.95--0.44)	**1.33E-07**	**5.65E-07**	0.14	(-0.48-0.76)	6.58E-01	7.31E-01	notSig	-0.18	(-0.58-0.21)	3.58E-01	4.15E-01
CE(18:3)	-4.43	(-5.6--3.24)	**1.59E-12**	**3.69E-10**	GUSTO and BIS	-2.46	(-3--1.91)	**6.77E-19**	**1.85E-17**	2.20	(1.43-2.97)	**2.92E-08**	**2.38E-07**	BIS only	1.40	(0.81-1.98)	**2.73E-06**	**7.24E-06**
CE(20:1)	-0.30	(-0.98-0.38)	3.84E-01	4.73E-01	GUSTO only	-0.67	(-1.05--0.29)	**5.79E-04**	**1.13E-03**	1.11	(0.33-1.9)	**5.51E-03**	**1.42E-02**	BIS only	0.86	(0.28-1.43)	**3.60E-03**	**6.75E-03**
CE(20:2)	-1.31	(-1.91--0.72)	**1.87E-05**	**1.08E-04**	GUSTO and BIS	-1.32	(-1.66--0.98)	**2.52E-14**	**2.93E-13**	1.17	(0.57-1.78)	**1.47E-04**	**5.52E-04**	GUSTO and BIS	1.00	(0.55-1.44)	**1.04E-05**	**2.60E-05**
CE(20:4)	-1.38	(-2.49--0.27)	**1.51E-02**	**3.24E-02**	BIS only	-0.84	(-1.39--0.29)	**2.95E-03**	**5.14E-03**	1.21	(0.43-1.98)	**2.38E-03**	**6.68E-03**	BIS only	0.32	(-0.14-0.79)	1.72E-01	2.22E-01
CE(20:5)	-2.86	(-4.45--1.24)	**6.17E-04**	**2.19E-03**	GUSTO and BIS	-2.18	(-3.21--1.15)	**3.28E-05**	**8.36E-05**	1.01	(-0.11-2.13)	7.69E-02	1.31E-01	GUSTO only	-0.82	(-1.58--0.05)	**3.66E-02**	5.67E-02
CE(22:5) (n6)	-2.13	(-3.07--1.17)	**1.65E-05**	**9.70E-05**	GUSTO and BIS	-1.95	(-2.44--1.45)	**1.99E-14**	**2.44E-13**	1.56	(0.94-2.17)	**8.10E-07**	**5.01E-06**	BIS only	1.13	(0.65-1.61)	**4.76E-06**	**1.24E-05**
CE(22:6)	-1.77	(-2.92--0.61)	**2.93E-03**	**8.08E-03**	GUSTO and BIS	-1.61	(-2.22--1)	**2.48E-07**	**9.75E-07**	0.14	(-0.48-0.76)	6.58E-01	7.31E-01	GUSTO only	-0.65	(-1.14--0.15)	**9.99E-03**	**1.71E-02**
CE(24:5)	0.29	(-0.71-1.3)	5.69E-01	6.48E-01	GUSTO only	-1.24	(-1.82--0.67)	**2.48E-05**	**6.69E-05**	-1.43	(-2.54--0.31)	**1.21E-02**	**2.75E-02**	GUSTO and BIS	-2.34	(-3.02--1.67)	**1.14E-11**	**4.55E-11**
CE(24:6)	-3.03	(-4.14--1.91)	**1.92E-07**	**2.41E-06**	GUSTO and BIS	-2.69	(-3.3--2.08)	**4.80E-18**	**1.24E-16**	-0.37	(-1.22-0.47)	3.85E-01	4.90E-01	GUSTO only	-0.71	(-1.34--0.08)	**2.72E-02**	**4.35E-02**
DG(16:0_16:1)	1.88	(0.13-3.66)	**3.49E-02**	6.55E-02	notSig	0.96	(0.07-1.85)	**3.54E-02**	**4.97E-02**	-1.05	(-1.86--0.24)	**1.15E-02**	**2.65E-02**	GUSTO and BIS	-1.18	(-1.76--0.6)	**6.52E-05**	**1.54E-04**
DG(14:0_18:2)	-1.79	(-3.27--0.29)	**1.99E-02**	**4.02E-02**	BIS only	-1.27	(-2.1--0.43)	**2.89E-03**	**5.05E-03**	-2.05	(-3.02--1.08)	**3.47E-05**	**1.49E-04**	GUSTO and BIS	-2.53	(-3.23--1.82)	**2.49E-12**	**1.06E-11**
DG(16:0_18:1)	2.82	(1.49-4.16)	**2.90E-05**	**1.57E-04**	GUSTO and BIS	1.68	(1.03-2.33)	**3.83E-07**	**1.42E-06**	-1.34	(-2.33--0.36)	**7.64E-03**	**1.88E-02**	GUSTO and BIS	-1.72	(-2.29--1.16)	**2.38E-09**	**8.12E-09**
DG(16:1_18:1)	3.07	(1.7-4.47)	**1.09E-05**	**6.72E-05**	BIS only	1.43	(0.69-2.17)	**1.38E-04**	**3.16E-04**	-0.86	(-1.72--0.01)	**4.75E-02**	9.00E-02	GUSTO only	-1.57	(-2.12--1.01)	**2.91E-08**	**8.99E-08**
DG(16:0_18:2)	1.91	(0.58-3.25)	**4.66E-03**	**1.21E-02**	GUSTO and BIS	1.15	(0.51-1.78)	**3.88E-04**	**7.93E-04**	-2.81	(-3.89--1.73)	**4.47E-07**	**3.01E-06**	GUSTO and BIS	-2.99	(-3.59--2.39)	**1.76E-22**	**1.36E-21**
DG(18:0_18:1)	2.39	(1.17-3.63)	**1.27E-04**	**5.45E-04**	GUSTO and BIS	1.60	(1.01-2.19)	**1.29E-07**	**5.53E-07**	-0.66	(-1.5-0.18)	1.25E-01	1.95E-01	GUSTO only	-1.02	(-1.57--0.47)	**2.89E-04**	**6.35E-04**
DG(18:1_18:1)	3.44	(2.11-4.79)	**3.65E-07**	**3.93E-06**	GUSTO and BIS	1.84	(1.2-2.48)	**1.70E-08**	**7.89E-08**	-0.91	(-1.93-0.11)	8.07E-02	1.35E-01	GUSTO only	-1.57	(-2.13--1.01)	**3.94E-08**	**1.20E-07**
DG(18:0_18:2)	1.65	(0.26-3.06)	**1.98E-02**	**4.01E-02**	GUSTO and BIS	1.14	(0.47-1.81)	**8.15E-04**	**1.54E-03**	-1.86	(-2.84--0.87)	**2.22E-04**	**8.05E-04**	GUSTO and BIS	-1.74	(-2.42--1.05)	**7.02E-07**	**1.93E-06**
DG(18:1_18:2)	2.55	(1.26-3.86)	**1.00E-04**	**4.47E-04**	GUSTO and BIS	1.33	(0.7-1.97)	**3.83E-05**	**9.51E-05**	-2.31	(-3.35--1.27)	**1.45E-05**	**6.95E-05**	GUSTO and BIS	-2.92	(-3.47--2.37)	**9.92E-26**	**9.21E-25**
DG(18:2_18:2)	1.55	(-0.09-3.22)	6.41E-02	1.11E-01	notSig	1.04	(0.23-1.86)	**1.19E-02**	**1.84E-02**	-3.01	(-4.18--1.84)	**5.35E-07**	**3.54E-06**	GUSTO and BIS	-3.84	(-4.46--3.23)	**1.99E-34**	**2.98E-33**
DG(18:1_18:3)	0.76	(-0.53-2.06)	2.50E-01	3.37E-01	notSig	0.47	(-0.2-1.14)	1.67E-01	2.06E-01	-1.91	(-2.84--0.98)	**6.56E-05**	**2.65E-04**	GUSTO and BIS	-2.32	(-2.93--1.71)	**1.08E-13**	**5.07E-13**
DG(16:0_20:4)	2.13	(0.73-3.55)	**2.73E-03**	**7.58E-03**	GUSTO and BIS	1.47	(0.7-2.25)	**2.00E-04**	**4.39E-04**	-2.23	(-3.31--1.16)	**5.00E-05**	**2.07E-04**	GUSTO and BIS	-2.12	(-2.77--1.48)	**1.10E-10**	**3.94E-10**
DG(18:1_20:3)	1.68	(0.53-2.84)	**4.11E-03**	**1.09E-02**	BIS only	0.80	(0.17-1.44)	**1.29E-02**	**1.96E-02**	0.37	(-0.43-1.17)	3.59E-01	4.65E-01	notSig	0.04	(-0.53-0.61)	8.84E-01	9.07E-01
DG(18:1_20:4)	3.07	(1.99-4.16)	**2.60E-08**	**4.17E-07**	GUSTO and BIS	1.75	(1.14-2.36)	**2.01E-08**	**9.07E-08**	-1.70	(-2.54--0.86)	**7.91E-05**	**3.06E-04**	GUSTO and BIS	-1.92	(-2.45--1.4)	**6.80E-13**	**2.98E-12**
DG(16:0_22:5)	2.32	(0.94-3.72)	**9.73E-04**	**3.07E-03**	BIS only	1.08	(0.33-1.82)	**4.65E-03**	**7.82E-03**	-4.31	(-5.44--3.18)	**2.22E-13**	**4.12E-12**	GUSTO and BIS	-2.76	(-3.42--2.09)	**3.35E-16**	**1.85E-15**
DG(18:2_20:4)	2.45	(1.24-3.67)	**6.69E-05**	**3.11E-04**	BIS only	1.35	(0.65-2.04)	**1.50E-04**	**3.41E-04**	-3.12	(-4.09--2.15)	**4.51E-10**	**4.77E-09**	GUSTO and BIS	-3.13	(-3.74--2.53)	**2.07E-24**	**1.78E-23**
DG(16:0_22:6)	3.43	(1.88-5.01)	**1.30E-05**	**7.85E-05**	BIS only	1.94	(1.03-2.86)	**3.23E-05**	**8.28E-05**	-4.45	(-5.52--3.39)	**1.00E-15**	**2.45E-14**	GUSTO and BIS	-4.20	(-4.79--3.61)	**5.66E-44**	**1.75E-42**
DG(18:1_22:5)	3.06	(1.95-4.17)	**5.46E-08**	**7.45E-07**	BIS only	1.43	(0.76-2.09)	**2.68E-05**	**7.06E-05**	-1.58	(-2.32--0.84)	**3.41E-05**	**1.48E-04**	GUSTO and BIS	-1.73	(-2.27--1.19)	**4.74E-10**	**1.68E-09**
DG(18:1_22:6)	4.34	(2.91-5.79)	**2.49E-09**	**5.50E-08**	GUSTO and BIS	2.53	(1.67-3.39)	**7.83E-09**	**3.79E-08**	-3.89	(-4.84--2.93)	**4.72E-15**	**1.04E-13**	GUSTO and BIS	-3.63	(-4.19--3.07)	**4.57E-37**	**9.64E-36**
DG(18:2_22:6)	3.34	(1.77-4.94)	**2.92E-05**	**1.57E-04**	GUSTO and BIS	2.12	(1.21-3.02)	**4.73E-06**	**1.41E-05**	-5.08	(-6.21--3.96)	**4.34E-18**	**2.01E-16**	GUSTO and BIS	-4.36	(-5.01--3.71)	**7.07E-40**	**1.64E-38**

TG(48:0) [NL-18:0]	-3.52	(-5.19--1.83)	**5.76E-05**	**2.73E-04**	GUSTO and BIS	-1.98	(-2.76--1.2)	**6.07E-07**	**2.13E-06**	0.07	(-0.69-0.84)	8.51E-01	8.91E-01	GUSTO only	-0.58	(-1.16-0)	5.07E-02	7.47E-02
TG(48:1) [NL-16:1]	-0.31	(-1.92-1.33)	7.10E-01	7.74E-01	notSig	0.24	(-0.69-1.17)	6.09E-01	6.62E-01	-0.31	(-1.24-0.62)	5.11E-01	6.06E-01	GUSTO only	-1.34	(-2.01--0.68)	**7.78E-05**	**1.82E-04**
TG(48:1) [NL-18:1]	-2.33	(-3.29--1.37)	**3.02E-06**	**2.46E-05**	BIS only	-1.40	(-2.05--0.75)	**2.50E-05**	**6.69E-05**	-0.80	(-1.74-0.14)	9.49E-02	1.54E-01	GUSTO only	-2.11	(-2.73--1.48)	**4.31E-11**	**1.61E-10**
TG(48:2) [NL-14:0]	-1.38	(-2.47--0.28)	**1.44E-02**	**3.12E-02**	BIS only	-1.15	(-1.9--0.39)	**2.83E-03**	**4.98E-03**	-2.11	(-3.22--1)	**1.96E-04**	**7.20E-04**	GUSTO and BIS	-3.24	(-3.86--2.62)	**1.29E-24**	**1.13E-23**
TG(48:2) [NL-14:1]	-1.37	(-2.85-0.13)	7.37E-02	1.22E-01	GUSTO only	-1.26	(-2.12--0.39)	**4.40E-03**	**7.46E-03**	-1.65	(-2.91--0.39)	**1.01E-02**	**2.37E-02**	GUSTO and BIS	-2.67	(-3.33--2)	**5.20E-15**	**2.65E-14**
TG(48:2) [NL-16:1]	0.72	(-0.78-2.25)	3.48E-01	4.37E-01	notSig	0.21	(-0.78-1.2)	6.73E-01	7.19E-01	-0.87	(-1.76-0.03)	5.73E-02	1.05E-01	GUSTO only	-1.92	(-2.53--1.3)	**9.98E-10**	**3.43E-09**
TG(48:2) [NL-18:2]	-2.43	(-3.5--1.36)	**1.21E-05**	**7.41E-05**	GUSTO and BIS	-1.78	(-2.52--1.03)	**2.94E-06**	**9.16E-06**	-2.40	(-3.4--1.39)	**3.60E-06**	**1.99E-05**	GUSTO and BIS	-3.50	(-4.11--2.89)	**3.79E-29**	**4.19E-28**
TG(48:3) [NL-14:0]	-1.75	(-3.43--0.04)	**4.49E-02**	8.05E-02	GUSTO only	-1.58	(-2.53--0.64)	**9.75E-04**	**1.81E-03**	-3.53	(-4.8--2.25)	**7.61E-08**	**5.88E-07**	GUSTO and BIS	-3.91	(-4.55--3.27)	**3.03E-33**	**4.26E-32**
TG(48:3) [NL-16:1]	0.38	(-1.25-2.05)	6.47E-01	7.13E-01	notSig	-0.64	(-1.59-0.31)	1.86E-01	2.27E-01	-2.17	(-3.11--1.23)	**6.87E-06**	**3.58E-05**	GUSTO and BIS	-2.62	(-3.2--2.04)	**9.32E-19**	**5.61E-18**
TG(48:3) [NL-18:3]	-3.12	(-4.76--1.46)	**2.71E-04**	**1.06E-03**	GUSTO and BIS	-2.30	(-3.3--1.29)	**7.64E-06**	**2.19E-05**	-2.68	(-3.82--1.55)	**4.18E-06**	**2.23E-05**	GUSTO and BIS	-3.08	(-3.68--2.47)	**1.61E-23**	**1.31E-22**
TG(49:1) [NL-17:1]	0.59	(-0.99-2.2)	4.67E-01	5.50E-01	notSig	0.39	(-0.37-1.16)	3.14E-01	3.62E-01	-1.18	(-2.06--0.3)	**8.39E-03**	**2.04E-02**	GUSTO and BIS	-1.80	(-2.34--1.26)	**6.71E-11**	**2.43E-10**
TG(50:1) [NL-14:0]	-2.79	(-3.99--1.57)	**9.51E-06**	**6.23E-05**	BIS only	-1.37	(-2.01--0.73)	**3.05E-05**	**7.90E-05**	-0.33	(-1.16-0.51)	4.43E-01	5.41E-01	GUSTO only	-0.83	(-1.46--0.21)	**9.17E-03**	**1.59E-02**
TG(50:1) [NL-16:0]	0.08	(-0.72-0.89)	8.39E-01	8.75E-01	GUSTO only	0.56	(0.07-1.04)	**2.39E-02**	**3.43E-02**	-0.91	(-1.67--0.15)	**1.93E-02**	**4.12E-02**	GUSTO and BIS	-1.65	(-2.2--1.1)	**4.95E-09**	**1.64E-08**
TG(50:1) [NL-18:1]	0.05	(-0.66-0.76)	8.98E-01	9.20E-01	GUSTO only	0.47	(0.02-0.92)	**3.88E-02**	5.39E-02	-0.86	(-1.52--0.21)	**1.01E-02**	**2.37E-02**	GUSTO and BIS	-1.50	(-2.01--0.99)	**7.44E-09**	**2.40E-08**
TG(50:2) [NL-14:0]	-0.97	(-1.73--0.2)	**1.41E-02**	**3.07E-02**	GUSTO and BIS	-0.76	(-1.22--0.3)	**1.14E-03**	**2.08E-03**	-0.48	(-1.4-0.45)	3.11E-01	4.14E-01	GUSTO only	-2.09	(-2.7--1.48)	**1.95E-11**	**7.56E-11**
TG(50:2) [NL-16:1]	1.29	(0.46-2.13)	**2.41E-03**	**6.77E-03**	BIS only	0.92	(0.38-1.47)	**9.27E-04**	**1.73E-03**	-0.44	(-1.23-0.34)	2.68E-01	3.67E-01	GUSTO only	-1.62	(-2.2--1.05)	**3.43E-08**	**1.05E-07**
TG(50:2) [NL-18:1]	0.16	(-0.42-0.75)	5.85E-01	6.61E-01	notSig	0.23	(-0.19-0.66)	2.82E-01	3.33E-01	-0.42	(-1.11-0.27)	2.33E-01	3.33E-01	GUSTO only	-1.50	(-2.03--0.96)	**4.88E-08**	**1.45E-07**
TG(50:2) [NL-18:2]	-0.43	(-1.17-0.32)	2.61E-01	3.50E-01	GUSTO only	0.25	(-0.21-0.7)	2.91E-01	3.40E-01	-2.06	(-2.84--1.27)	**3.16E-07**	**2.19E-06**	GUSTO and BIS	-3.08	(-3.64--2.52)	**4.68E-27**	**4.72E-26**
TG(50:3) [NL-14:0]	-1.31	(-2.05--0.58)	**5.22E-04**	**1.92E-03**	BIS only	-0.91	(-1.43--0.39)	**5.80E-04**	**1.13E-03**	-2.92	(-4.09--1.74)	**1.49E-06**	**8.86E-06**	GUSTO and BIS	-3.87	(-4.5--3.23)	**4.82E-33**	**6.39E-32**
TG(50:3) [NL-14:1]	-0.54	(-1.57-0.5)	3.05E-01	3.97E-01	notSig	-0.61	(-1.17--0.06)	**3.04E-02**	**4.30E-02**	-1.34	(-2.49--0.18)	**2.31E-02**	**4.77E-02**	GUSTO and BIS	-2.03	(-2.7--1.35)	**3.68E-09**	**1.23E-08**
TG(50:3) [NL-16:1]	1.82	(0.94-2.72)	**5.63E-05**	**2.69E-04**	BIS only	1.17	(0.55-1.78)	**1.83E-04**	**4.07E-04**	-1.95	(-2.85--1.05)	**2.21E-05**	**1.01E-04**	GUSTO and BIS	-3.10	(-3.67--2.52)	**3.03E-26**	**2.99E-25**
TG(50:3) [NL-18:2]	0.36	(-0.27-0.99)	2.69E-01	3.60E-01	notSig	0.32	(-0.16-0.81)	1.85E-01	2.26E-01	-2.52	(-3.45--1.6)	**1.12E-07**	**8.36E-07**	GUSTO and BIS	-3.65	(-4.23--3.08)	**1.24E-35**	**2.13E-34**
TG(50:3) [NL-18:3]	-1.20	(-2.3--0.08)	**3.50E-02**	6.56E-02	notSig	-0.41	(-1.12-0.3)	2.57E-01	3.06E-01	-2.39	(-3.36--1.42)	**1.62E-06**	**9.51E-06**	GUSTO and BIS	-3.25	(-3.86--2.64)	**1.05E-25**	**9.52E-25**
TG(50:4) [NL-14:0]	-1.26	(-2.67-0.17)	8.43E-02	1.37E-01	notSig	-1.00	(-1.83--0.18)	**1.73E-02**	**2.55E-02**	-4.67	(-6.01--3.34)	**1.27E-11**	**1.73E-10**	GUSTO and BIS	-4.39	(-5.01--3.77)	**1.13E-43**	**3.28E-42**
TG(50:4) [NL-18:3]	-0.36	(-1.65-0.94)	5.84E-01	6.61E-01	notSig	-0.58	(-1.39-0.23)	1.62E-01	2.01E-01	-3.25	(-4.4--2.11)	**3.61E-08**	**2.84E-07**	GUSTO and BIS	-3.75	(-4.33--3.17)	**1.06E-36**	**2.05E-35**
TG(50:4) [NL-20:4]	-1.30	(-2.92-0.35)	1.22E-01	1.84E-01	notSig	-1.02	(-2.07-0.04)	5.92E-02	8.06E-02	-2.53	(-3.68--1.38)	**1.81E-05**	**8.42E-05**	GUSTO and BIS	-3.15	(-3.85--2.45)	**1.04E-18**	**6.18E-18**
TG(51:1) [NL-17:0]	1.56	(0.29-2.83)	**1.55E-02**	**3.29E-02**	BIS only	0.90	(0.25-1.54)	**6.39E-03**	**1.04E-02**	-1.72	(-2.74--0.71)	**8.91E-04**	**2.77E-03**	GUSTO and BIS	-2.36	(-2.97--1.75)	**3.56E-14**	**1.72E-13**
TG(51:2) [NL-15:0]	1.27	(0.22-2.32)	**1.79E-02**	**3.72E-02**	BIS only	-0.02	(-0.58-0.54)	9.37E-01	9.47E-01	-2.20	(-3.35--1.06)	**1.71E-04**	**6.36E-04**	GUSTO and BIS	-3.00	(-3.64--2.35)	**9.85E-20**	**6.38E-19**
TG(51:2) [NL-17:0]	2.24	(1.27-3.23)	**6.14E-06**	**4.43E-05**	BIS only	0.99	(0.42-1.55)	**5.85E-04**	**1.14E-03**	-1.83	(-2.83--0.84)	**3.12E-04**	**1.11E-03**	GUSTO and BIS	-2.83	(-3.43--2.23)	**3.31E-20**	**2.19E-19**
TG(51:2) [NL-17:1]	2.25	(1.27-3.24)	**6.21E-06**	**4.43E-05**	BIS only	0.95	(0.4-1.5)	**6.95E-04**	**1.33E-03**	-1.84	(-2.84--0.84)	**3.17E-04**	**1.11E-03**	GUSTO and BIS	-2.57	(-3.12--2.01)	**1.23E-19**	**7.83E-19**
TG(52:1) [NL-18:0]	1.13	(0.07-2.21)	**3.75E-02**	6.89E-02	GUSTO only	1.29	(0.65-1.93)	**7.59E-05**	**1.81E-04**	-0.34	(-1.34-0.67)	5.14E-01	6.06E-01	GUSTO only	-1.13	(-1.83--0.43)	**1.48E-03**	**2.90E-03**
TG(52:1) [NL-18:1]	1.62	(0.43-2.82)	**7.55E-03**	**1.79E-02**	GUSTO and BIS	1.50	(0.85-2.15)	**5.43E-06**	**1.57E-05**	-0.56	(-1.66-0.55)	3.24E-01	4.28E-01	GUSTO only	-1.26	(-1.99--0.53)	**6.97E-04**	**1.45E-03**
TG(52:2) [NL-16:0]	1.13	(0.49-1.77)	**5.41E-04**	**1.97E-03**	GUSTO and BIS	0.90	(0.55-1.24)	**2.89E-07**	**1.12E-06**	-0.99	(-1.78--0.2)	**1.40E-02**	**3.09E-02**	GUSTO and BIS	-2.02	(-2.53--1.5)	**2.34E-14**	**1.14E-13**
TG(52:2) [NL-18:2]	-0.46	(-1.3-0.38)	2.83E-01	3.74E-01	notSig	0.14	(-0.35-0.64)	5.74E-01	6.31E-01	-1.63	(-2.53--0.73)	**3.91E-04**	**1.33E-03**	GUSTO and BIS	-2.20	(-2.83--1.56)	**9.40E-12**	**3.86E-11**
TG(52:3) [NL-16:1]	1.90	(1.05-2.75)	**1.03E-05**	**6.59E-05**	BIS only	0.86	(0.39-1.34)	**3.56E-04**	**7.37E-04**	-0.57	(-1.42-0.28)	1.90E-01	2.80E-01	GUSTO only	-2.11	(-2.68--1.54)	**5.56E-13**	**2.45E-12**
TG(52:3) [NL-18:2]	0.51	(-0.04-1.07)	7.11E-02	1.19E-01	GUSTO only	0.66	(0.32-1.01)	**1.84E-04**	**4.07E-04**	-2.39	(-3.27--1.51)	**1.37E-07**	**9.90E-07**	GUSTO and BIS	-3.61	(-4.16--3.05)	**2.57E-37**	**5.69E-36**
TG(52:4) [NL-16:1]	2.54	(1.64-3.44)	**2.78E-08**	**4.30E-07**	BIS only	1.42	(0.85-1.98)	**9.55E-07**	**3.10E-06**	-3.04	(-4.19--1.89)	**2.88E-07**	**2.02E-06**	GUSTO and BIS	-4.12	(-4.72--3.53)	**2.39E-42**	**6.16E-41**
TG(52:4) [NL-18:2]	1.00	(0.17-1.84)	**1.88E-02**	**3.85E-02**	GUSTO and BIS	1.11	(0.55-1.67)	**1.13E-04**	**2.62E-04**	-3.62	(-4.64--2.59)	**1.11E-11**	**1.61E-10**	GUSTO and BIS	-4.53	(-5.12--3.95)	**3.66E-52**	**1.70E-50**
TG(52:4) [NL-18:3]	-0.10	(-0.85-0.65)	7.92E-01	8.41E-01	notSig	0.20	(-0.32-0.71)	4.51E-01	5.09E-01	-2.88	(-4--1.75)	**6.99E-07**	**4.44E-06**	GUSTO and BIS	-3.75	(-4.36--3.15)	**6.14E-34**	**8.90E-33**
TG(52:5) [NL-18:3]	0.20	(-1.12-1.53)	7.71E-01	8.21E-01	notSig	0.13	(-0.67-0.93)	7.44E-01	7.84E-01	-4.40	(-5.64--3.16)	**8.60E-12**	**1.33E-10**	GUSTO and BIS	-4.22	(-4.82--3.62)	**6.84E-43**	**1.87E-41**
TG(52:5) [NL-20:4]	1.26	(-0.1-2.63)	6.85E-02	1.16E-01	notSig	0.82	(-0.08-1.72)	7.36E-02	9.81E-02	-2.37	(-3.41--1.33)	**8.78E-06**	**4.35E-05**	GUSTO and BIS	-3.14	(-3.78--2.49)	**2.43E-21**	**1.73E-20**
TG(52:5) [NL-20:5]	-0.16	(-1.67-1.38)	8.41E-01	8.75E-01	notSig	0.43	(-0.55-1.41)	3.92E-01	4.47E-01	-4.88	(-6.11--3.66)	**1.65E-14**	**3.33E-13**	GUSTO and BIS	-4.56	(-5.19--3.92)	**7.69E-45**	**2.74E-43**
TG(53:2) [NL-18:1]	2.37	(1.44-3.31)	**5.97E-07**	**5.90E-06**	GUSTO and BIS	1.39	(0.84-1.94)	**6.65E-07**	**2.26E-06**	-1.82	(-2.78--0.86)	**2.02E-04**	**7.36E-04**	GUSTO and BIS	-2.40	(-2.95--1.85)	**1.36E-17**	**7.80E-17**
TG(54:1) [NL-18:1]	0.32	(-1.39-2.06)	7.16E-01	7.77E-01	GUSTO only	0.90	(0.28-1.53)	**4.52E-03**	**7.62E-03**	0.19	(-0.84-1.21)	7.22E-01	7.92E-01	GUSTO only	-0.76	(-1.47--0.05)	**3.56E-02**	5.52E-02
TG(54:2) [NL-18:0]	0.95	(-0.15-2.06)	9.07E-02	1.46E-01	GUSTO only	1.28	(0.69-1.87)	**1.97E-05**	**5.44E-05**	-0.31	(-1.36-0.73)	5.58E-01	6.40E-01	GUSTO only	-1.21	(-1.88--0.53)	**4.79E-04**	**1.03E-03**
TG(54:2) [NL-20:1]	2.17	(1.15-3.19)	**2.60E-05**	**1.44E-04**	BIS only	1.07	(0.46-1.69)	**6.58E-04**	**1.27E-03**	-0.36	(-1.53-0.81)	5.45E-01	6.32E-01	GUSTO only	-1.33	(-2.01--0.64)	**1.40E-04**	**3.20E-04**
TG(54:3) [NL-18:1]	0.68	(-0.2-1.58)	1.31E-01	1.96E-01	notSig	0.60	(0.13-1.08)	**1.27E-02**	**1.94E-02**	-0.07	(-0.75-0.61)	8.34E-01	8.82E-01	GUSTO only	-1.04	(-1.54--0.54)	**4.79E-05**	**1.15E-04**
TG(54:3) [NL-18:2]	0.43	(-0.44-1.31)	3.33E-01	4.21E-01	GUSTO only	0.63	(0.15-1.11)	**1.02E-02**	**1.59E-02**	-1.84	(-2.74--0.93)	**7.18E-05**	**2.85E-04**	GUSTO and BIS	-2.58	(-3.15--2.02)	**3.03E-19**	**1.88E-18**
TG(54:4) [NL-18:2]	0.45	(-0.55-1.45)	3.81E-01	4.70E-01	notSig	0.38	(-0.16-0.91)	1.66E-01	2.05E-01	-2.10	(-3.02--1.18)	**8.74E-06**	**4.35E-05**	GUSTO and BIS	-3.25	(-3.84--2.67)	**1.13E-27**	**1.19E-26**
TG(54:4) [NL-20:3]	0.90	(0.07-1.73)	**3.33E-02**	6.31E-02	notSig	0.74	(0.21-1.27)	**5.86E-03**	**9.61E-03**	-1.21	(-2.13--0.3)	**9.51E-03**	**2.26E-02**	GUSTO and BIS	-2.04	(-2.65--1.42)	**1.13E-10**	**4.04E-10**
TG(54:5) [NL-18:3]	0.00	(-1.21-1.22)	9.98E-01	9.98E-01	notSig	0.04	(-0.66-0.75)	9.02E-01	9.21E-01	-2.32	(-3.36--1.27)	**1.47E-05**	**6.95E-05**	GUSTO and BIS	-3.32	(-3.9--2.74)	**2.65E-29**	**3.00E-28**
TG(54:5) [NL-20:4]	1.44	(0.64-2.25)	**4.00E-04**	**1.51E-03**	GUSTO and BIS	1.21	(0.7-1.72)	**3.66E-06**	**1.11E-05**	-2.32	(-3.25--1.38)	**1.46E-06**	**8.79E-06**	GUSTO and BIS	-3.12	(-3.73--2.5)	**2.94E-23**	**2.35E-22**

TG(54:6) [NL-18:3]	0.13	(-1.5-1.79)	8.78E-01	9.01E-01	notSig	-0.23	(-1.18-0.72)	6.39E-01	6.89E-01	-3.59	(-4.73--2.45)	**1.06E-09**	**1.03E-08**	GUSTO and BIS	-3.78	(-4.38--3.19)	**2.63E-35**	**4.21E-34**
TG(54:6) [NL-20:4]	1.79	(0.88-2.71)	**1.16E-04**	**5.05E-04**	GUSTO and BIS	1.45	(0.83-2.06)	**4.34E-06**	**1.30E-05**	-3.12	(-4.1--2.14)	**7.87E-10**	**7.77E-09**	GUSTO and BIS	-3.73	(-4.34--3.12)	**3.88E-33**	**5.29E-32**
TG(54:6) [NL-20:5]	1.12	(-0.09-2.35)	6.96E-02	1.17E-01	notSig	0.97	(0.14-1.81)	**2.18E-02**	**3.15E-02**	-4.78	(-5.95--3.6)	**6.83E-15**	**1.44E-13**	GUSTO and BIS	-4.97	(-5.57--4.36)	**3.94E-58**	**2.28E-56**
TG(54:6) [NL-22:6]	1.92	(0.5-3.36)	**7.92E-03**	**1.85E-02**	GUSTO and BIS	1.84	(0.91-2.78)	**1.06E-04**	**2.47E-04**	-4.45	(-5.51--3.38)	**1.07E-15**	**2.49E-14**	GUSTO and BIS	-4.93	(-5.53--4.34)	**6.08E-59**	**4.03E-57**
TG(54:7) [NL-20:5]	2.11	(0.44-3.82)	**1.34E-02**	**2.95E-02**	BIS only	1.07	(0.05-2.1)	**4.02E-02**	5.57E-02	-6.33	(-7.66--5)	**1.20E-19**	**7.13E-18**	GUSTO and BIS	-5.27	(-5.89--4.65)	**1.35E-61**	**1.57E-59**
TG(54:7) [NL-22:6]	3.03	(1.47-4.61)	**1.30E-04**	**5.49E-04**	BIS only	1.84	(0.82-2.85)	**3.90E-04**	**7.93E-04**	-5.23	(-6.42--4.05)	**3.09E-17**	**1.10E-15**	GUSTO and BIS	-5.00	(-5.59--4.4)	**5.92E-61**	**4.58E-59**
TG(56:6) [NL-20:4]	1.52	(0.58-2.46)	**1.53E-03**	**4.58E-03**	GUSTO and BIS	1.38	(0.92-1.84)	**4.37E-09**	**2.23E-08**	-1.04	(-1.92--0.15)	**2.18E-02**	**4.56E-02**	GUSTO and BIS	-2.11	(-2.71--1.52)	**3.91E-12**	**1.64E-11**
TG(56:7) [NL-20:4]	1.01	(0.1-1.92)	**2.88E-02**	5.56E-02	GUSTO only	1.02	(0.48-1.56)	**2.28E-04**	**4.92E-04**	-2.52	(-3.61--1.44)	**6.15E-06**	**3.24E-05**	GUSTO and BIS	-3.26	(-3.91--2.62)	**3.97E-23**	**3.12E-22**
TG(56:7) [NL-20:5]	0.93	(-0.31-2.19)	1.42E-01	2.11E-01	notSig	0.63	(-0.2-1.46)	1.39E-01	1.74E-01	-3.67	(-4.79--2.56)	**2.02E-10**	**2.47E-09**	GUSTO and BIS	-3.75	(-4.38--3.12)	**3.53E-31**	**4.31E-30**
TG(56:7) [NL-22:6]	3.23	(2.1-4.37)	**2.13E-08**	**3.81E-07**	GUSTO and BIS	2.56	(1.77-3.35)	**2.14E-10**	**1.36E-09**	-4.32	(-5.32--3.31)	**1.93E-16**	**5.98E-15**	GUSTO and BIS	-4.83	(-5.4--4.25)	**2.44E-61**	**2.27E-59**
TG(56:8) [NL-20:4]	0.85	(-0.38-2.09)	1.78E-01	2.55E-01	notSig	0.82	(0.07-1.56)	**3.19E-02**	**4.49E-02**	-3.04	(-4.15--1.94)	**8.83E-08**	**6.72E-07**	GUSTO and BIS	-2.98	(-3.64--2.32)	**7.27E-19**	**4.44E-18**
TG(56:8) [NL-20:5]	1.07	(-0.46-2.61)	1.71E-01	2.47E-01	notSig	0.54	(-0.43-1.51)	2.78E-01	3.29E-01	-5.25	(-6.49--4.01)	**5.15E-16**	**1.41E-14**	GUSTO and BIS	-4.40	(-5.02--3.79)	**1.62E-44**	**5.38E-43**
TG(56:8) [NL-22:6]	3.61	(2.28-4.97)	**1.02E-07**	**1.31E-06**	GUSTO and BIS	2.60	(1.7-3.51)	**1.75E-08**	**7.98E-08**	-5.76	(-6.86--4.65)	**6.44E-23**	**1.19E-20**	GUSTO and BIS	-5.46	(-6.03--4.9)	**9.51E-80**	**4.41E-77**
TG(56:9) [NL-22:6]	2.32	(0.63-4.04)	**7.16E-03**	**1.70E-02**	BIS only	1.29	(0.31-2.27)	**9.72E-03**	**1.52E-02**	-6.30	(-7.52--5.09)	**7.68E-23**	**1.19E-20**	GUSTO and BIS	-5.21	(-5.79--4.62)	**1.65E-68**	**3.83E-66**
TG(58:10) [NL-22:6]	1.99	(0.3-3.71)	**2.10E-02**	**4.18E-02**	BIS only	1.29	(0.32-2.26)	**9.36E-03**	**1.47E-02**	-5.80	(-6.99--4.61)	**1.65E-20**	**1.27E-18**	GUSTO and BIS	-4.80	(-5.39--4.21)	**1.63E-57**	**8.42E-56**
TG(58:8) [NL-22:6]	2.68	(1.4-3.98)	**4.04E-05**	**2.06E-04**	GUSTO and BIS	1.97	(1.14-2.8)	**3.53E-06**	**1.08E-05**	-4.00	(-5.01--2.99)	**2.81E-14**	**5.43E-13**	GUSTO and BIS	-4.34	(-4.92--3.76)	**2.48E-49**	**1.04E-47**
TG(58:9) [NL-22:6]	2.27	(0.81-3.74)	**2.19E-03**	**6.26E-03**	BIS only	1.62	(0.72-2.53)	**4.53E-04**	**9.06E-04**	-5.43	(-6.62--4.24)	**2.33E-18**	**1.20E-16**	GUSTO and BIS	-4.92	(-5.5--4.34)	**4.05E-62**	**6.26E-60**
TG(O-50:1) [NL-16:0]	-0.17	(-1.21-0.88)	7.51E-01	8.08E-01	notSig	-0.19	(-0.71-0.32)	4.60E-01	5.19E-01	-0.74	(-1.49-0.02)	5.49E-02	1.01E-01	notSig	-0.66	(-1.19--0.13)	**1.39E-02**	**2.33E-02**
TG(O-50:1) [NL-17:1]	-0.65	(-1.62-0.34)	1.97E-01	2.76E-01	notSig	-0.10	(-0.75-0.55)	7.61E-01	7.99E-01	-0.61	(-1.19--0.03)	**3.78E-02**	7.30E-02	GUSTO only	-1.25	(-1.69--0.8)	**4.46E-08**	**1.33E-07**
TG(O-50:1) [NL-18:1]	-0.83	(-1.86-0.21)	1.16E-01	1.79E-01	notSig	-0.68	(-1.23--0.12)	**1.70E-02**	**2.53E-02**	-0.86	(-1.65--0.07)	**3.23E-02**	6.40E-02	GUSTO only	-0.93	(-1.46--0.39)	**7.04E-04**	**1.46E-03**
TG(O-50:2) [NL-18:2]	-0.25	(-1.58-1.1)	7.14E-01	7.76E-01	notSig	-0.10	(-0.63-0.43)	7.25E-01	7.70E-01	-0.57	(-1.52-0.38)	2.42E-01	3.43E-01	notSig	-0.50	(-1.1-0.1)	1.05E-01	1.44E-01
TG(O-52:2) [NL-16:0]	0.73	(-0.37-1.85)	1.91E-01	2.71E-01	notSig	0.12	(-0.54-0.77)	7.27E-01	7.70E-01	-0.04	(-1.03-0.96)	9.42E-01	9.54E-01	GUSTO only	-0.62	(-1.31-0.07)	7.77E-02	1.09E-01
TG(O-52:2) [NL-17:1]	0.64	(-0.33-1.62)	1.99E-01	2.78E-01	notSig	0.45	(-0.1-1)	1.12E-01	1.43E-01	-0.56	(-1.27-0.14)	1.18E-01	1.86E-01	GUSTO only	-1.78	(-2.27--1.29)	**9.10E-13**	**3.95E-12**
TG(O-52:2) [NL-18:1]	0.72	(-0.36-1.81)	1.93E-01	2.72E-01	notSig	-0.02	(-0.68-0.64)	9.47E-01	9.55E-01	-0.58	(-1.36-0.19)	1.38E-01	2.12E-01	GUSTO only	-1.09	(-1.69--0.49)	**3.34E-04**	**7.27E-04**
TG(O-54:2) [NL-18:1]	-0.29	(-1.33-0.77)	5.90E-01	6.64E-01	GUSTO only	0.52	(-0.05-1.08)	7.46E-02	9.89E-02	-0.40	(-1.24-0.45)	3.57E-01	4.63E-01	GUSTO only	-1.77	(-2.29--1.24)	**5.95E-11**	**2.17E-10**
TG(O-54:4) [NL-17:1]	-0.64	(-1.98-0.72)	3.54E-01	4.42E-01	notSig	-0.09	(-0.76-0.57)	7.81E-01	8.15E-01	-1.75	(-3.65-0.14)	7.01E-02	1.23E-01	GUSTO only	-3.73	(-4.42--3.04)	**3.57E-26**	**3.45E-25**
TG(O-54:4) [NL-18:2]	1.17	(-0.64-3.02)	2.07E-01	2.89E-01	notSig	0.25	(-0.49-0.98)	5.12E-01	5.72E-01	-1.24	(-2.74-0.27)	1.07E-01	1.70E-01	GUSTO only	-3.95	(-4.6--3.31)	**4.99E-33**	**6.43E-32**
dhCer(d18:0/22:0)	0.06	(-0.7-0.82)	8.75E-01	9.01E-01	GUSTO only	0.69	(0.2-1.18)	**5.76E-03**	**9.49E-03**	1.24	(0.7-1.79)	**9.64E-06**	**4.66E-05**	BIS only	0.74	(0.31-1.17)	**6.87E-04**	**1.44E-03**
dhCer(d18:0/24:0)	-0.60	(-1.34-0.15)	1.18E-01	1.81E-01	notSig	-0.10	(-0.57-0.36)	6.64E-01	7.13E-01	0.80	(0.17-1.43)	**1.31E-02**	**2.90E-02**	BIS only	0.19	(-0.27-0.66)	4.21E-01	4.79E-01
dhCer(d18:0/24:1)	2.18	(1.31-3.04)	**7.33E-07**	**6.77E-06**	GUSTO and BIS	1.89	(1.34-2.44)	**1.64E-11**	**1.28E-10**	1.21	(0.52-1.9)	**5.91E-04**	**1.93E-03**	BIS only	0.63	(0.1-1.17)	**2.11E-02**	**3.44E-02**
Cer(d16:1/22:0)	-0.82	(-1.81-0.17)	1.04E-01	1.63E-01	GUSTO only	-0.75	(-1.27--0.24)	**3.98E-03**	**6.81E-03**	-0.13	(-0.89-0.63)	7.36E-01	8.01E-01	GUSTO only	-0.59	(-1.12--0.05)	**3.11E-02**	**4.91E-02**
Cer(d16:1/23:0)	-1.81	(-2.87--0.73)	**1.07E-03**	**3.34E-03**	GUSTO and BIS	-1.22	(-1.79--0.65)	**2.51E-05**	**6.69E-05**	-0.46	(-1.25-0.33)	2.49E-01	3.49E-01	notSig	-0.64	(-1.31-0.03)	6.07E-02	8.70E-02
Cer(d16:1/24:0)	-2.27	(-3.24--1.28)	**8.83E-06**	**5.94E-05**	GUSTO and BIS	-2.10	(-2.63--1.57)	**6.69E-15**	**8.63E-14**	-0.23	(-0.91-0.45)	5.09E-01	6.06E-01	notSig	-0.48	(-0.98-0.02)	5.95E-02	8.54E-02
Cer(d16:1/24:1)	0.99	(0.06-1.93)	**3.63E-02**	6.69E-02	notSig	0.03	(-0.49-0.55)	9.04E-01	9.21E-01	0.32	(-0.44-1.09)	4.05E-01	5.07E-01	notSig	-0.02	(-0.61-0.56)	9.37E-01	9.45E-01
Cer(d17:1/22:0)	0.01	(-0.89-0.93)	9.77E-01	9.82E-01	GUSTO only	-0.83	(-1.36--0.3)	**2.04E-03**	**3.64E-03**	-0.16	(-0.8-0.49)	6.35E-01	7.17E-01	notSig	-0.37	(-0.9-0.17)	1.79E-01	2.29E-01
Cer(d17:1/23:0)	-0.62	(-1.66-0.42)	2.39E-01	3.26E-01	GUSTO only	-1.09	(-1.65--0.52)	**1.72E-04**	**3.87E-04**	-0.57	(-1.37-0.23)	1.63E-01	2.46E-01	notSig	-0.74	(-1.44--0.04)	**3.75E-02**	5.75E-02
Cer(d17:1/24:0)	-0.87	(-1.76-0.03)	5.68E-02	1.00E-01	GUSTO only	-1.80	(-2.28--1.32)	**2.78E-13**	**2.67E-12**	-0.51	(-1.11-0.08)	8.98E-02	1.47E-01	GUSTO only	-0.73	(-1.19--0.26)	**2.23E-03**	**4.27E-03**
Cer(d17:1/24:1)	1.23	(0.34-2.12)	**6.90E-03**	**1.66E-02**	GUSTO and BIS	-0.32	(-0.83-0.19)	2.18E-01	2.64E-01	-0.05	(-0.77-0.66)	8.80E-01	9.07E-01	notSig	-0.41	(-0.96-0.13)	1.38E-01	1.83E-01
Cer(d18:1/16:0)	-0.12	(-0.75-0.5)	7.00E-01	7.64E-01	GUSTO only	-0.60	(-0.97--0.23)	**1.51E-03**	**2.73E-03**	-0.25	(-0.99-0.49)	5.04E-01	6.06E-01	notSig	-0.41	(-0.87-0.05)	8.23E-02	1.15E-01
Cer(d18:1/18:0)	2.86	(1.89-3.83)	**6.98E-09**	**1.35E-07**	GUSTO and BIS	2.26	(1.71-2.81)	**9.19E-16**	**1.47E-14**	-0.23	(-0.99-0.53)	5.53E-01	6.37E-01	notSig	-0.48	(-0.99-0.03)	6.59E-02	9.35E-02
Cer(d18:1/20:0)	1.49	(0.58-2.41)	**1.28E-03**	**3.87E-03**	BIS only	0.58	(0.1-1.06)	**1.82E-02**	**2.66E-02**	0.09	(-0.64-0.82)	8.06E-01	8.64E-01	notSig	-0.37	(-0.85-0.12)	1.42E-01	1.88E-01
Cer(d18:1/22:0)	0.32	(-0.42-1.08)	3.94E-01	4.79E-01	notSig	0.04	(-0.37-0.44)	8.64E-01	8.85E-01	0.80	(0.08-1.51)	**2.95E-02**	5.92E-02	notSig	0.29	(-0.18-0.76)	2.26E-01	2.80E-01
Cer(d18:1/23:0)	-0.19	(-0.98-0.6)	6.37E-01	7.06E-01	notSig	-0.34	(-0.75-0.07)	1.04E-01	1.35E-01	-0.29	(-1.19-0.61)	5.26E-01	6.17E-01	notSig	-0.36	(-0.95-0.23)	2.33E-01	2.87E-01
Cer(d18:1/24:0)	-0.56	(-1.26-0.15)	1.25E-01	1.88E-01	GUSTO only	-0.83	(-1.22--0.44)	**2.71E-05**	**7.11E-05**	0.52	(-0.1-1.15)	1.02E-01	1.65E-01	notSig	0.06	(-0.38-0.5)	7.89E-01	8.30E-01
Cer(d18:1/24:1)	2.15	(1.4-2.9)	**2.24E-08**	**3.83E-07**	BIS only	0.77	(0.33-1.2)	**5.12E-04**	**1.01E-03**	0.71	(-0.04-1.47)	6.48E-02	1.15E-01	notSig	0.14	(-0.36-0.64)	5.90E-01	6.48E-01
Cer(d18:2/16:0)	0.45	(-0.15-1.05)	1.39E-01	2.08E-01	notSig	0.05	(-0.39-0.48)	8.32E-01	8.60E-01	0.42	(-0.32-1.15)	2.63E-01	3.63E-01	notSig	0.32	(-0.26-0.91)	2.82E-01	3.40E-01
Cer(d18:2/22:0)	0.75	(-0.07-1.58)	7.26E-02	1.21E-01	notSig	0.00	(-0.47-0.47)	1.00E+00	1.00E+00	0.74	(0.08-1.4)	**2.84E-02**	5.73E-02	notSig	0.29	(-0.2-0.78)	2.46E-01	3.00E-01
Cer(d18:2/23:0)	0.31	(-0.57-1.19)	4.93E-01	5.72E-01	notSig	-0.29	(-0.78-0.2)	2.51E-01	3.00E-01	-0.14	(-0.98-0.69)	7.37E-01	8.01E-01	notSig	-0.25	(-0.89-0.39)	4.43E-01	4.99E-01
Cer(d18:2/24:0)	-0.71	(-1.51-0.1)	8.56E-02	1.39E-01	GUSTO only	-1.08	(-1.53--0.62)	**3.50E-06**	**1.08E-05**	0.40	(-0.28-1.09)	2.49E-01	3.49E-01	notSig	-0.18	(-0.68-0.31)	4.63E-01	5.15E-01
Cer(d18:2/24:1)	2.56	(1.72-3.4)	**2.06E-09**	**4.77E-08**	BIS only	0.79	(0.32-1.27)	**1.05E-03**	**1.94E-03**	0.98	(0.28-1.68)	**6.30E-03**	**1.57E-02**	BIS only	0.52	(0.01-1.04)	**4.71E-02**	7.03E-02
Cer(d19:1/24:0)	0.85	(-0.11-1.81)	8.16E-02	1.33E-01	GUSTO only	-0.26	(-0.91-0.4)	4.43E-01	5.03E-01	-0.06	(-0.7-0.58)	8.49E-01	8.91E-01	GUSTO only	-0.48	(-0.99-0.03)	6.26E-02	8.94E-02
Cer(d19:1/24:1)	3.55	(2.42-4.7)	**8.62E-10**	**2.34E-08**	BIS only	1.45	(0.68-2.22)	**2.30E-04**	**4.93E-04**	0.19	(-0.62-0.99)	6.53E-01	7.31E-01	notSig	-0.35	(-0.99-0.29)	2.80E-01	3.38E-01

Cer(m18:0/22:0)	-0.83	(-2.06-0.41)	1.89E-01	2.68E-01	notSig	-0.06	(-0.72-0.6)	8.57E-01	8.82E-01	-0.33	(-1.13-0.48)	4.25E-01	5.26E-01	GUSTO only	-0.70	(-1.32--0.09)	**2.55E-02**	**4.10E-02**
Cer(m18:0/23:0)	-1.40	(-2.61--0.17)	**2.60E-02**	5.09E-02	notSig	-0.20	(-0.91-0.51)	5.80E-01	6.36E-01	-1.06	(-1.98--0.13)	**2.54E-02**	5.17E-02	notSig	-1.11	(-1.94--0.28)	**8.89E-03**	**1.55E-02**
Cer(m18:0/24:0)	-2.13	(-3.31--0.92)	**5.83E-04**	**2.10E-03**	BIS only	-1.07	(-1.73--0.42)	**1.33E-03**	**2.43E-03**	-0.62	(-1.4-0.16)	1.19E-01	1.86E-01	GUSTO only	-0.91	(-1.46--0.37)	**1.08E-03**	**2.20E-03**
Cer(m18:0/24:1)	0.66	(-0.57-1.9)	2.96E-01	3.87E-01	GUSTO only	0.98	(0.26-1.7)	**7.82E-03**	**1.25E-02**	-0.53	(-1.45-0.39)	2.60E-01	3.63E-01	GUSTO only	-0.95	(-1.63--0.26)	**6.81E-03**	**1.21E-02**
Cer(m18:1/22:0)	0.04	(-1.12-1.21)	9.50E-01	9.64E-01	notSig	0.43	(-0.26-1.11)	2.26E-01	2.72E-01	-0.75	(-1.54-0.05)	6.51E-02	1.15E-01	GUSTO only	-1.76	(-2.28--1.24)	**3.29E-11**	**1.24E-10**
Cer(m18:1/23:0)	-0.58	(-1.76-0.62)	3.41E-01	4.30E-01	notSig	-0.10	(-0.83-0.62)	7.75E-01	8.10E-01	-1.59	(-2.57--0.61)	**1.46E-03**	**4.34E-03**	GUSTO and BIS	-2.20	(-3--1.41)	**5.32E-08**	**1.57E-07**
Cer(m18:1/24:0)	-1.07	(-2.13-0.01)	5.17E-02	9.19E-02	notSig	-0.65	(-1.28--0.02)	**4.47E-02**	6.18E-02	-0.93	(-1.66--0.2)	**1.21E-02**	**2.75E-02**	GUSTO and BIS	-1.82	(-2.32--1.33)	**4.92E-13**	**2.24E-12**
Cer(m18:1/24:1)	1.69	(0.52-2.88)	**4.75E-03**	**1.22E-02**	GUSTO and BIS	1.49	(0.77-2.2)	**4.63E-05**	**1.14E-04**	-0.84	(-1.68--0.01)	**4.75E-02**	9.00E-02	GUSTO only	-1.53	(-2.1--0.95)	**2.07E-07**	**5.95E-07**
GM3(d18:1/18:0)	-0.02	(-0.71-0.69)	9.64E-01	9.70E-01	GUSTO only	-0.74	(-1.16--0.31)	**6.75E-04**	**1.29E-03**	-0.21	(-0.72-0.3)	4.26E-01	5.26E-01	GUSTO only	-0.44	(-0.83--0.05)	**2.56E-02**	**4.11E-02**
GM3(d18:1/22:0)	-0.33	(-1.15-0.5)	4.39E-01	5.25E-01	GUSTO only	-1.50	(-2--1.01)	**2.53E-09**	**1.37E-08**	0.64	(0.01-1.28)	**4.78E-02**	9.02E-02	notSig	0.49	(-0.05-1.03)	7.66E-02	1.07E-01
GM3(d18:1/24:0)	-1.84	(-2.77--0.89)	**1.55E-04**	**6.37E-04**	GUSTO and BIS	-2.77	(-3.29--2.26)	**4.58E-26**	**4.25E-24**	0.66	(-0.03-1.34)	6.13E-02	1.11E-01	notSig	0.17	(-0.35-0.69)	5.23E-01	5.78E-01
GM3(d18:1/24:1)	0.52	(-0.34-1.39)	2.33E-01	3.19E-01	GUSTO only	-1.03	(-1.52--0.54)	**3.56E-05**	**8.91E-05**	0.63	(-0.08-1.33)	8.00E-02	1.35E-01	notSig	0.33	(-0.2-0.87)	2.25E-01	2.80E-01
HexCer(d16:1/22:0)	-1.53	(-2.4--0.64)	**7.70E-04**	**2.57E-03**	GUSTO and BIS	-1.66	(-2.18--1.14)	**2.92E-10**	**1.78E-09**	0.04	(-0.72-0.8)	9.15E-01	9.33E-01	notSig	-0.14	(-0.8-0.52)	6.77E-01	7.26E-01
HexCer(d16:1/24:0)	-2.20	(-3.04--1.34)	**6.57E-07**	**6.22E-06**	GUSTO and BIS	-2.85	(-3.36--2.33)	**4.63E-27**	**5.37E-25**	-0.22	(-0.88-0.43)	5.02E-01	6.05E-01	notSig	-0.37	(-0.94-0.2)	2.04E-01	2.58E-01
HexCer(d18:1/16:0)	-0.69	(-1.48-0.12)	9.39E-02	1.50E-01	GUSTO only	-1.15	(-1.56--0.73)	**6.18E-08**	**2.68E-07**	0.77	(-0.04-1.59)	6.35E-02	1.14E-01	notSig	0.38	(-0.2-0.97)	2.00E-01	2.54E-01
HexCer(d18:1/18:0)	-0.24	(-1.08-0.6)	5.71E-01	6.49E-01	GUSTO only	-1.12	(-1.61--0.64)	**4.85E-06**	**1.43E-05**	0.52	(-0.31-1.36)	2.17E-01	3.13E-01	notSig	0.43	(-0.22-1.09)	1.95E-01	2.48E-01
HexCer(d18:1/20:0)	-0.19	(-1-0.62)	6.41E-01	7.08E-01	GUSTO only	-1.30	(-1.77--0.84)	**3.73E-08**	**1.65E-07**	0.82	(0.13-1.52)	**2.02E-02**	**4.27E-02**	BIS only	0.56	(0-1.13)	5.02E-02	7.41E-02
HexCer(d18:1/22:0)	-0.93	(-1.75--0.11)	**2.73E-02**	5.29E-02	GUSTO only	-1.67	(-2.09--1.24)	**1.38E-14**	**1.73E-13**	0.58	(-0.08-1.25)	8.55E-02	1.42E-01	notSig	0.55	(0.04-1.05)	**3.43E-02**	5.36E-02
HexCer(d18:1/24:0)	-1.88	(-2.65--1.1)	**3.16E-06**	**2.48E-05**	GUSTO and BIS	-2.38	(-2.81--1.96)	**2.66E-28**	**4.11E-26**	0.43	(-0.2-1.06)	1.81E-01	2.70E-01	notSig	0.28	(-0.2-0.77)	2.53E-01	3.08E-01
HexCer(d18:1/24:1)	0.03	(-0.79-0.85)	9.46E-01	9.64E-01	GUSTO only	-1.14	(-1.6--0.69)	**1.02E-06**	**3.25E-06**	0.95	(0.32-1.58)	**3.02E-03**	**8.10E-03**	BIS only	0.80	(0.31-1.3)	**1.40E-03**	**2.76E-03**
Hex2Cer(d16:1/16:0)	-1.54	(-2.44--0.63)	**9.65E-04**	**3.07E-03**	GUSTO and BIS	-1.38	(-1.89--0.86)	**1.56E-07**	**6.30E-07**	-0.60	(-1.45-0.24)	1.62E-01	2.46E-01	notSig	-0.78	(-1.43--0.13)	**1.95E-02**	**3.22E-02**
Hex2Cer(d18:1/16:0)	-1.32	(-2.07--0.57)	**6.43E-04**	**2.23E-03**	GUSTO and BIS	-1.49	(-1.92--1.06)	**9.38E-12**	**7.64E-11**	0.04	(-0.72-0.81)	9.10E-01	9.30E-01	notSig	-0.27	(-0.79-0.25)	3.08E-01	3.66E-01
Hex2Cer(d18:1/22:0)	-1.01	(-1.72--0.3)	**5.74E-03**	**1.42E-02**	GUSTO and BIS	-1.46	(-1.9--1.02)	**7.22E-11**	**4.93E-10**	0.31	(-0.35-0.98)	3.56E-01	4.63E-01	notSig	0.13	(-0.41-0.66)	6.38E-01	6.88E-01
Hex2Cer(d18:1/24:1)	-0.33	(-1.2-0.54)	4.53E-01	5.34E-01	GUSTO only	-1.25	(-1.74--0.76)	**5.00E-07**	**1.79E-06**	0.64	(-0.13-1.41)	1.05E-01	1.69E-01	notSig	0.27	(-0.29-0.83)	3.45E-01	4.03E-01
Hex2Cer(d18:2/16:0)	-0.40	(-1.18-0.39)	3.22E-01	4.14E-01	GUSTO only	-0.58	(-1.03--0.13)	**1.10E-02**	**1.70E-02**	0.11	(-0.58-0.79)	7.57E-01	8.19E-01	notSig	-0.14	(-0.66-0.38)	5.94E-01	6.52E-01
Hex3Cer(d18:1/16:0)	-0.40	(-0.95-0.16)	1.58E-01	2.31E-01	GUSTO only	-0.81	(-1.14--0.47)	**2.22E-06**	**6.97E-06**	0.01	(-0.48-0.49)	9.79E-01	9.79E-01	notSig	-0.19	(-0.56-0.19)	3.31E-01	3.90E-01
Hex3Cer(d18:1/22:0)	-1.08	(-1.75--0.4)	**1.90E-03**	**5.59E-03**	GUSTO and BIS	-1.33	(-1.78--0.88)	**6.73E-09**	**3.32E-08**	0.01	(-0.65-0.67)	9.71E-01	9.73E-01	notSig	0.05	(-0.5-0.6)	8.47E-01	8.83E-01
Hex3Cer(d18:1/24:0)	-1.93	(-2.71--1.15)	**1.79E-06**	**1.49E-05**	GUSTO and BIS	-1.70	(-2.16--1.23)	**7.77E-13**	**7.07E-12**	-0.22	(-0.88-0.44)	5.11E-01	6.06E-01	notSig	-0.32	(-0.82-0.19)	2.19E-01	2.73E-01
Hex3Cer(d18:1/24:1)	-0.24	(-0.86-0.38)	4.48E-01	5.34E-01	GUSTO only	-0.74	(-1.15--0.33)	**4.43E-04**	**8.90E-04**	0.24	(-0.35-0.84)	4.19E-01	5.20E-01	notSig	-0.05	(-0.52-0.42)	8.32E-01	8.71E-01
SM(d17:1/14:0)	-1.42	(-2.13--0.7)	**1.29E-04**	**5.48E-04**	GUSTO and BIS	-1.70	(-2.15--1.25)	**9.43E-14**	**9.72E-13**	-0.42	(-1.05-0.22)	1.98E-01	2.88E-01	GUSTO only	-0.75	(-1.15--0.35)	**2.76E-04**	**6.11E-04**
SM(d18:0/14:0)	-1.55	(-2.31--0.78)	**8.10E-05**	**3.72E-04**	GUSTO and BIS	-1.11	(-1.54--0.67)	**6.32E-07**	**2.19E-06**	0.89	(0.31-1.46)	**2.43E-03**	**6.76E-03**	BIS only	0.26	(-0.12-0.65)	1.80E-01	2.30E-01
SM(d18:1/14:0/SM(d16:1/16:0)	-1.10	(-1.73--0.47)	**6.97E-04**	**2.36E-03**	GUSTO and BIS	-1.05	(-1.43--0.67)	**5.15E-08**	**2.26E-07**	0.08	(-0.57-0.72)	8.17E-01	8.70E-01	notSig	-0.31	(-0.71-0.09)	1.27E-01	1.72E-01
SM(d18:2/14:0)	-0.28	(-0.89-0.34)	3.76E-01	4.67E-01	notSig	-0.01	(-0.4-0.38)	9.66E-01	9.72E-01	0.75	(0.16-1.34)	**1.23E-02**	**2.76E-02**	BIS only	0.39	(-0.01-0.79)	5.77E-02	8.32E-02
SM(d17:1/16:0)	-0.17	(-0.81-0.47)	5.97E-01	6.69E-01	GUSTO only	-0.99	(-1.37--0.62)	**1.39E-07**	**5.86E-07**	-0.43	(-1.11-0.25)	2.17E-01	3.13E-01	GUSTO only	-0.79	(-1.19--0.38)	**1.33E-04**	**3.06E-04**
SM(d18:1/16:0)	-0.07	(-0.49-0.35)	7.40E-01	8.01E-01	GUSTO only	-0.60	(-0.93--0.26)	**4.33E-04**	**8.74E-04**	0.33	(-0.35-1.01)	3.45E-01	4.51E-01	notSig	-0.05	(-0.5-0.4)	8.28E-01	8.69E-01
SM(d18:2/16:0)	0.87	(0.23-1.52)	**7.72E-03**	**1.82E-02**	BIS only	0.30	(-0.04-0.64)	8.61E-02	1.13E-01	0.84	(0.21-1.47)	**9.23E-03**	**2.21E-02**	BIS only	0.38	(-0.02-0.78)	6.45E-02	9.18E-02
SM(34:3)	0.77	(0.21-1.33)	**7.00E-03**	**1.67E-02**	BIS only	0.44	(0.08-0.8)	**1.79E-02**	**2.64E-02**	0.62	(0.04-1.2)	**3.50E-02**	6.90E-02	notSig	0.37	(-0.03-0.78)	7.18E-02	1.01E-01
SM(d18:2/17:0)	1.10	(0.37-1.83)	**2.94E-03**	**8.08E-03**	BIS only	0.06	(-0.33-0.45)	7.71E-01	8.08E-01	-0.28	(-1-0.44)	4.40E-01	5.40E-01	GUSTO only	-0.70	(-1.12--0.28)	**1.01E-03**	**2.05E-03**
SM(35:2) (b)	-0.08	(-0.77-0.61)	8.19E-01	8.62E-01	GUSTO only	-0.92	(-1.37--0.47)	**5.41E-05**	**1.31E-04**	0.45	(-0.67-1.57)	4.34E-01	5.34E-01	notSig	-0.33	(-0.95-0.29)	2.97E-01	3.57E-01
SM(d18:1/18:0/SM(d16:1/20:0)	1.36	(0.58-2.14)	**6.33E-04**	**2.21E-03**	BIS only	0.58	(0.15-1)	**7.96E-03**	**1.27E-02**	0.86	(0.16-1.57)	**1.66E-02**	**3.61E-02**	BIS only	0.33	(-0.13-0.78)	1.56E-01	2.03E-01
SM(d18:2/18:1)	1.51	(0.81-2.22)	**2.36E-05**	**1.33E-04**	BIS only	0.73	(0.33-1.12)	**2.99E-04**	**6.28E-04**	0.51	(-0.23-1.24)	1.77E-01	2.65E-01	notSig	0.20	(-0.27-0.66)	4.07E-01	4.65E-01
SM(37:1)	0.72	(0.06-1.39)	**3.23E-02**	6.17E-02	GUSTO only	-0.16	(-0.58-0.26)	4.63E-01	5.19E-01	-0.24	(-0.98-0.51)	5.31E-01	6.18E-01	GUSTO only	-0.57	(-1.06--0.08)	**2.27E-02**	**3.69E-02**
SM(37:2)	1.13	(0.41-1.86)	**2.12E-03**	**6.10E-03**	GUSTO and BIS	-0.25	(-0.71-0.22)	2.99E-01	3.49E-01	-0.03	(-0.78-0.72)	9.37E-01	9.51E-01	GUSTO only	-0.46	(-0.94-0.01)	5.63E-02	8.17E-02
SM(d18:1/20:0)/SM(d16:1/22:0)	-0.52	(-1.21-0.19)	1.49E-01	2.20E-01	GUSTO only	-0.69	(-1.05--0.33)	**1.72E-04**	**3.87E-04**	0.95	(0.29-1.6)	**4.51E-03**	**1.18E-02**	BIS only	0.56	(0.14-0.98)	**9.74E-03**	**1.67E-02**
SM(d18:2/20:0)	0.62	(0.02-1.23)	**4.38E-02**	7.91E-02	notSig	0.04	(-0.35-0.43)	8.44E-01	8.70E-01	1.12	(0.5-1.74)	**3.98E-04**	**1.35E-03**	BIS only	0.80	(0.32-1.28)	**1.13E-03**	**2.26E-03**
SM(38:3) (a)	0.95	(0.22-1.68)	**1.03E-02**	**2.35E-02**	BIS only	0.63	(0.23-1.04)	**2.25E-03**	**4.00E-03**	0.02	(-0.59-0.63)	9.52E-01	9.60E-01	notSig	-0.27	(-0.69-0.14)	1.91E-01	2.44E-01
SM(38:3) (b)	0.82	(0.14-1.51)	**1.79E-02**	**3.72E-02**	GUSTO and BIS	-0.14	(-0.57-0.29)	5.33E-01	5.95E-01	0.49	(-0.19-1.16)	1.57E-01	2.39E-01	notSig	0.23	(-0.27-0.73)	3.61E-01	4.17E-01
SM(d16:1/23:0)/SM(d17:1/22:0)	-0.81	(-1.51--0.11)	**2.40E-02**	**4.72E-02**	GUSTO and BIS	-1.00	(-1.39--0.61)	**4.10E-07**	**1.50E-06**	-0.17	(-0.93-0.59)	6.58E-01	7.31E-01	notSig	-0.30	(-0.74-0.13)	1.70E-01	2.20E-01
SM(d18:1/22:0)/SM(d16:1/24:0)	-0.35	(-0.91-0.21)	2.15E-01	2.98E-01	GUSTO only	-0.50	(-0.85--0.14)	**6.67E-03**	**1.08E-02**	0.93	(0.32-1.54)	**2.75E-03**	**7.50E-03**	BIS only	0.65	(0.23-1.07)	**2.40E-03**	**4.59E-03**
SM(d16:1/24:1)	0.46	(-0.31-1.23)	2.43E-01	3.30E-01	GUSTO only	-0.35	(-0.77-0.08)	1.10E-01	1.40E-01	0.95	(0.18-1.72)	**1.57E-02**	**3.43E-02**	BIS only	0.29	(-0.19-0.77)	2.30E-01	2.84E-01
SM(d18:2/22:0)	-0.11	(-0.77-0.56)	7.48E-01	8.08E-01	GUSTO only	-0.57	(-0.95--0.19)	**3.04E-03**	**5.26E-03**	1.12	(0.41-1.82)	**2.03E-03**	**5.84E-03**	BIS only	0.70	(0.24-1.17)	**3.20E-03**	**6.06E-03**
SM(40:3) (a)	0.84	(0.17-1.52)	**1.46E-02**	**3.14E-02**	BIS only	0.20	(-0.19-0.6)	3.15E-01	3.62E-01	0.55	(-0.11-1.21)	1.03E-01	1.66E-01	notSig	0.00	(-0.44-0.44)	9.98E-01	9.99E-01

SM(40:3) (b)	0.28	(-0.35-0.91)	3.88E-01	4.75E-01	GUSTO only	-0.40	(-0.81-0.02)	5.95E-02	8.08E-02	-0.34	(-1.01-0.33)	3.14E-01	4.17E-01	GUSTO only	-0.64	(-1.1--0.18)	**6.75E-03**	**1.20E-02**
SM(41:0)	-0.44	(-1.14-0.28)	2.30E-01	3.17E-01	notSig	-0.42	(-0.79--0.06)	**2.26E-02**	**3.26E-02**	0.07	(-0.52-0.66)	8.09E-01	8.65E-01	notSig	-0.10	(-0.59-0.4)	6.99E-01	7.46E-01
SM(d17:1/24:1)	1.08	(0.45-1.71)	**7.28E-04**	**2.45E-03**	GUSTO and BIS	-0.20	(-0.57-0.17)	2.90E-01	3.40E-01	-0.05	(-0.72-0.61)	8.76E-01	9.06E-01	notSig	-0.30	(-0.73-0.13)	1.66E-01	2.16E-01
SM(d18:2/23:0)	-0.44	(-1.06-0.18)	1.65E-01	2.40E-01	GUSTO only	-0.77	(-1.13--0.42)	**1.63E-05**	**4.54E-05**	0.12	(-0.67-0.9)	7.74E-01	8.31E-01	notSig	0.04	(-0.48-0.56)	8.69E-01	8.96E-01
SM(d18:1/24:0)	-0.89	(-1.51--0.26)	**5.55E-03**	**1.38E-02**	GUSTO and BIS	-1.26	(-1.63--0.9)	**1.68E-11**	**1.28E-10**	0.41	(-0.15-0.98)	1.51E-01	2.31E-01	notSig	0.09	(-0.31-0.5)	6.52E-01	7.01E-01
SM(d18:1/24:1)	1.63	(0.99-2.28)	**7.44E-07**	**6.77E-06**	BIS only	0.34	(-0.08-0.75)	1.10E-01	1.40E-01	1.08	(0.42-1.74)	**1.41E-03**	**4.28E-03**	BIS only	0.61	(0.16-1.05)	**7.53E-03**	**1.32E-02**
SM(d18:2/24:0)	-0.90	(-1.47--0.32)	**2.29E-03**	**6.51E-03**	GUSTO and BIS	-1.28	(-1.61--0.95)	**6.62E-14**	**7.15E-13**	0.33	(-0.26-0.91)	2.72E-01	3.72E-01	notSig	0.10	(-0.31-0.51)	6.21E-01	6.75E-01
SM(43:1)	-0.30	(-0.92-0.33)	3.52E-01	4.40E-01	GUSTO only	-1.13	(-1.51--0.75)	**4.63E-09**	**2.33E-08**	-0.82	(-1.44--0.2)	**9.72E-03**	**2.30E-02**	GUSTO and BIS	-0.91	(-1.39--0.43)	**1.78E-04**	**4.01E-04**
SM(44:1)	-0.70	(-1.29--0.11)	**2.04E-02**	**4.08E-02**	GUSTO and BIS	-1.29	(-1.63--0.95)	**1.41E-13**	**1.39E-12**	-0.28	(-0.86-0.29)	3.34E-01	4.37E-01	notSig	-0.40	(-0.78--0.01)	**4.33E-02**	6.53E-02
SM(44:2)	1.12	(0.47-1.76)	**6.60E-04**	**2.25E-03**	GUSTO and BIS	-0.29	(-0.68-0.1)	1.49E-01	1.86E-01	0.42	(-0.11-0.94)	1.20E-01	1.86E-01	notSig	0.10	(-0.27-0.47)	6.00E-01	6.55E-01
LPC(14:0) [sn2]	-2.10	(-3.31--0.87)	**8.49E-04**	**2.79E-03**	BIS only	-1.12	(-1.78--0.47)	**7.98E-04**	**1.51E-03**	1.68	(0.89-2.47)	**3.30E-05**	**1.46E-04**	GUSTO and BIS	1.73	(1.3-2.15)	**1.87E-15**	**9.76E-15**
LPC(14:0) [sn1]	-1.99	(-3.28--0.68)	**3.01E-03**	**8.16E-03**	BIS only	-0.81	(-1.46--0.16)	**1.44E-02**	**2.17E-02**	1.55	(0.71-2.39)	**2.92E-04**	**1.04E-03**	GUSTO and BIS	1.60	(1.18-2.02)	**8.50E-14**	**4.07E-13**
LPC(15:0) [sn2]	-0.51	(-1.43-0.42)	2.83E-01	3.74E-01	GUSTO only	-1.19	(-1.71--0.68)	**5.38E-06**	**1.57E-05**	0.55	(-0.08-1.19)	8.88E-02	1.46E-01	GUSTO only	0.81	(0.37-1.25)	**2.70E-04**	**5.99E-04**
LPC(15:0) [sn1]	-0.33	(-1.25-0.6)	4.82E-01	5.65E-01	GUSTO only	-1.03	(-1.5--0.55)	**2.44E-05**	**6.69E-05**	0.56	(-0.05-1.17)	7.21E-02	1.25E-01	GUSTO only	0.67	(0.27-1.08)	**1.11E-03**	**2.24E-03**
LPC(16:0) [sn2]	0.65	(-0.51-1.83)	2.72E-01	3.62E-01	notSig	0.07	(-0.35-0.49)	7.40E-01	7.82E-01	1.29	(0.69-1.9)	**3.05E-05**	**1.36E-04**	GUSTO and BIS	1.56	(1.18-1.93)	**4.22E-16**	**2.30E-15**
LPC(16:0) [sn1]	0.53	(-0.46-1.54)	2.93E-01	3.85E-01	notSig	0.32	(-0.15-0.79)	1.77E-01	2.18E-01	1.48	(0.9-2.07)	**6.48E-07**	**4.18E-06**	GUSTO and BIS	1.71	(1.3-2.13)	**3.21E-16**	**1.80E-15**
LPC(16:1) [sn2]	0.61	(-0.5-1.73)	2.85E-01	3.76E-01	notSig	0.30	(-0.3-0.91)	3.28E-01	3.76E-01	2.89	(1.95-3.83)	**2.73E-09**	**2.53E-08**	GUSTO and BIS	3.57	(2.98-4.16)	**1.72E-32**	**2.16E-31**
LPC(16:1) [sn1]	0.73	(-0.29-1.76)	1.59E-01	2.32E-01	notSig	0.45	(-0.09-0.98)	1.03E-01	1.34E-01	2.96	(2.03-3.89)	**7.73E-10**	**7.77E-09**	GUSTO and BIS	3.55	(2.98-4.11)	**5.45E-35**	**8.44E-34**
LPC(15-MHDA) [sn2]	0.45	(-0.61-1.51)	4.06E-01	4.92E-01	GUSTO only	-0.74	(-1.35--0.13)	**1.72E-02**	**2.55E-02**	-0.05	(-0.69-0.59)	8.75E-01	9.06E-01	notSig	0.25	(-0.28-0.78)	3.50E-01	4.08E-01
LPC(15-MHDA) [sn1] / LPC(17:0) [sn2]	0.45	(-0.55-1.46)	3.78E-01	4.68E-01	GUSTO only	-0.75	(-1.29--0.21)	**6.36E-03**	**1.04E-02**	-0.22	(-0.8-0.37)	4.69E-01	5.67E-01	notSig	0.09	(-0.36-0.54)	6.96E-01	7.44E-01
LPC(17:0) [sn1]	0.29	(-0.69-1.29)	5.63E-01	6.43E-01	GUSTO only	-0.65	(-1.1--0.21)	**4.11E-03**	**7.01E-03**	-0.29	(-0.84-0.26)	2.96E-01	3.98E-01	notSig	-0.03	(-0.4-0.33)	8.60E-01	8.91E-01
LPC(17:1) [sn1] (a) / LPC(17:1) [sn2] (b)	0.15	(-0.79-1.09)	7.59E-01	8.13E-01	GUSTO only	-0.65	(-1.14--0.16)	**8.94E-03**	**1.41E-02**	1.44	(0.79-2.09)	**1.69E-05**	**7.90E-05**	GUSTO and BIS	1.58	(1.12-2.04)	**1.91E-11**	**7.44E-11**
LPC(18:0) [sn2]	-0.33	(-1.33-0.68)	5.17E-01	5.94E-01	notSig	-0.10	(-0.53-0.32)	6.37E-01	6.89E-01	0.89	(0.35-1.44)	**1.42E-03**	**4.29E-03**	GUSTO and BIS	0.95	(0.59-1.3)	**1.84E-07**	**5.35E-07**
LPC(18:0) [sn1]	-0.35	(-1.32-0.63)	4.84E-01	5.66E-01	notSig	-0.09	(-0.52-0.33)	6.69E-01	7.16E-01	0.85	(0.33-1.38)	**1.46E-03**	**4.34E-03**	GUSTO and BIS	0.93	(0.57-1.28)	**2.85E-07**	**8.18E-07**
LPC(18:1) [sn2]	-0.39	(-1.26-0.5)	3.91E-01	4.77E-01	GUSTO only	-0.54	(-0.99--0.09)	**1.89E-02**	**2.75E-02**	2.84	(2.25-3.44)	**1.23E-19**	**7.13E-18**	GUSTO and BIS	3.16	(2.7-3.62)	**1.57E-41**	**3.82E-40**
LPC(18:1) [sn1]	-0.58	(-1.49-0.33)	2.12E-01	2.94E-01	notSig	-0.52	(-0.93--0.1)	**1.41E-02**	**2.14E-02**	2.62	(2.09-3.14)	**3.10E-21**	**3.59E-19**	GUSTO and BIS	2.92	(2.52-3.33)	**2.29E-45**	**8.85E-44**
LPC(18:2) [sn2]	-1.39	(-2.25--0.52)	**1.86E-03**	**5.49E-03**	GUSTO and BIS	-0.82	(-1.25--0.4)	**1.53E-04**	**3.47E-04**	1.91	(1.24-2.58)	**3.10E-08**	**2.48E-07**	GUSTO and BIS	2.38	(1.88-2.87)	**4.09E-21**	**2.88E-20**
LPC(18:2) [sn1]	-2.03	(-3.05--1.01)	**1.15E-04**	**5.02E-04**	BIS only	-0.77	(-1.26--0.29)	**1.68E-03**	**3.01E-03**	2.34	(1.63-3.05)	**1.96E-10**	**2.46E-09**	GUSTO and BIS	2.63	(2.11-3.14)	**1.05E-23**	**8.71E-23**
LPC(18:3) [sn2] (a)	-2.89	(-3.97--1.8)	**3.23E-07**	**3.56E-06**	GUSTO and BIS	-1.93	(-2.52--1.35)	**8.77E-11**	**5.81E-10**	1.44	(0.78-2.1)	**1.95E-05**	**8.96E-05**	GUSTO and BIS	1.86	(1.39-2.33)	**1.30E-14**	**6.55E-14**
LPC(18:3) [sn1] (a)/LPC(18:3) [sn2] (b)	-3.07	(-4.21--1.91)	**3.18E-07**	**3.56E-06**	GUSTO and BIS	-1.54	(-2.15--0.94)	**6.54E-07**	**2.25E-06**	2.68	(1.97-3.39)	**2.83E-13**	**4.86E-12**	GUSTO and BIS	3.30	(2.73-3.87)	**4.86E-30**	**5.64E-29**
LPC(18:3) (a) [sn1] [104_sn1]	-3.24	(-4.4--2.07)	**1.01E-07**	**1.31E-06**	GUSTO and BIS	-1.61	(-2.24--0.98)	**4.79E-07**	**1.74E-06**	2.20	(1.47-2.94)	**5.05E-09**	**4.51E-08**	GUSTO and BIS	2.94	(2.37-3.51)	**4.30E-24**	**3.63E-23**
LPC(19:0) [sn1] (a) / LPC(19:0) [sn2] (b)	-0.92	(-1.88-0.05)	6.18E-02	1.08E-01	GUSTO only	-1.44	(-1.86--1.02)	**2.14E-11**	**1.58E-10**	-0.48	(-1.35-0.39)	2.78E-01	3.80E-01	notSig	-0.33	(-0.84-0.19)	2.15E-01	2.69E-01
LPC(20:0) [sn1]	-1.33	(-2.16--0.49)	**1.99E-03**	**5.76E-03**	GUSTO and BIS	-1.53	(-1.92--1.13)	**3.37E-14**	**3.72E-13**	0.46	(-0.04-0.95)	7.18E-02	1.25E-01	notSig	0.36	(0.04-0.69)	**2.75E-02**	**4.39E-02**
LPC(20:1) [sn2]	-0.35	(-1.31-0.61)	4.75E-01	5.57E-01	GUSTO only	-0.56	(-0.97--0.14)	**8.81E-03**	**1.40E-02**	1.81	(1.3-2.32)	**9.50E-12**	**1.42E-10**	GUSTO and BIS	1.63	(1.26-2)	**3.73E-18**	**2.19E-17**
LPC(20:1) [sn1]	-0.33	(-1.29-0.64)	5.00E-01	5.78E-01	GUSTO only	-0.56	(-0.98--0.15)	**8.30E-03**	**1.32E-02**	1.58	(1.09-2.08)	**5.70E-10**	**5.87E-09**	GUSTO and BIS	1.67	(1.32-2.02)	**8.39E-21**	**5.73E-20**
LPC(20:2) [sn2]	-1.74	(-2.63--0.84)	**1.77E-04**	**7.21E-04**	GUSTO and BIS	-1.11	(-1.55--0.67)	**8.29E-07**	**2.77E-06**	2.44	(1.88-2.99)	**5.34E-17**	**1.77E-15**	GUSTO and BIS	2.89	(2.44-3.35)	**1.77E-35**	**2.94E-34**
LPC(20:2) [sn1]	-1.58	(-2.61--0.53)	**3.24E-03**	**8.75E-03**	GUSTO and BIS	-0.80	(-1.23--0.36)	**3.01E-04**	**6.28E-04**	2.42	(1.89-2.96)	**8.39E-18**	**3.54E-16**	GUSTO and BIS	2.77	(2.34-3.2)	**7.23E-37**	**1.46E-35**
LPC(20:3) [sn2]	-0.47	(-1.38-0.45)	3.11E-01	4.03E-01	GUSTO only	0.54	(0.01-1.07)	**4.59E-02**	6.33E-02	2.08	(1.43-2.72)	**4.52E-10**	**4.77E-09**	GUSTO and BIS	2.31	(1.84-2.78)	**1.04E-21**	**7.53E-21**
LPC(20:3) [sn1]	-0.76	(-1.88-0.37)	1.87E-01	2.68E-01	GUSTO only	0.72	(0.13-1.31)	**1.68E-02**	**2.50E-02**	2.31	(1.62-3)	**1.12E-10**	**1.44E-09**	GUSTO and BIS	2.74	(2.23-3.26)	**8.02E-26**	**7.59E-25**
LPC(20:4) [sn2]	1.07	(0.19-1.95)	**1.72E-02**	**3.61E-02**	GUSTO and BIS	0.91	(0.46-1.36)	**8.01E-05**	**1.90E-04**	1.48	(0.77-2.19)	**4.40E-05**	**1.84E-04**	GUSTO and BIS	1.97	(1.49-2.45)	**7.76E-16**	**4.09E-15**
LPC(20:4) [sn1]	1.02	(-0.05-2.11)	6.15E-02	1.08E-01	GUSTO only	1.29	(0.78-1.79)	**6.11E-07**	**2.13E-06**	1.68	(0.89-2.48)	**3.65E-05**	**1.55E-04**	GUSTO and BIS	2.24	(1.71-2.76)	**5.90E-17**	**3.34E-16**
LPC(20:5) [sn2]	-0.56	(-1.89-0.78)	4.12E-01	4.96E-01	notSig	-0.25	(-1.09-0.6)	5.65E-01	6.24E-01	0.81	(-0.06-1.68)	6.85E-02	1.20E-01	notSig	0.68	(-0.01-1.37)	5.49E-02	7.99E-02
LPC(20:5) [sn1]	-0.74	(-2.22-0.76)	3.33E-01	4.21E-01	notSig	-0.12	(-1.01-0.78)	8.01E-01	8.31E-01	1.19	(0.3-2.09)	**8.96E-03**	**2.16E-02**	BIS only	0.98	(0.27-1.68)	**6.65E-03**	**1.19E-02**
LPC(22:4) [sn2]	-1.14	(-2.14--0.13)	**2.65E-02**	5.17E-02	notSig	-0.66	(-1.19--0.12)	**1.61E-02**	**2.41E-02**	1.74	(1.19-2.3)	**1.31E-09**	**1.25E-08**	GUSTO and BIS	2.03	(1.59-2.47)	**1.55E-19**	**9.70E-19**
LPC(22:4) [sn1]	-1.20	(-2.44-0.06)	6.15E-02	1.08E-01	notSig	-0.22	(-0.77-0.34)	4.42E-01	5.03E-01	1.82	(1.21-2.43)	**7.00E-09**	**6.01E-08**	GUSTO and BIS	2.22	(1.76-2.67)	**8.20E-22**	**6.04E-21**
LPC(22:5) [sn2] (n3)	-1.45	(-2.43--0.45)	**4.51E-03**	**1.18E-02**	GUSTO and BIS	-1.11	(-1.64--0.58)	**3.57E-05**	**8.91E-05**	0.85	(0.22-1.49)	**8.51E-03**	**2.06E-02**	GUSTO and BIS	1.16	(0.65-1.67)	**9.48E-06**	**2.40E-05**
LPC(22:5) [sn1] (n3)/LPC(22:5) [sn2] (n6)	-1.50	(-2.61--0.38)	**8.68E-03**	**2.00E-02**	GUSTO and BIS	-0.98	(-1.5--0.47)	**1.77E-04**	**3.96E-04**	1.76	(1.17-2.35)	**6.68E-09**	**5.84E-08**	GUSTO and BIS	1.90	(1.45-2.36)	**4.33E-16**	**2.34E-15**
LPC(22:5) [sn1] (n6)	-1.52	(-2.78--0.24)	**2.03E-02**	**4.08E-02**	BIS only	-0.80	(-1.46--0.14)	**1.81E-02**	**2.65E-02**	3.06	(2.26-3.87)	**2.41E-13**	**4.31E-12**	GUSTO and BIS	3.50	(2.83-4.17)	**1.21E-24**	**1.08E-23**
LPC(22:5) (n3) [sn1] [104_sn1]	-1.52	(-2.73--0.29)	**1.54E-02**	**3.28E-02**	BIS only	-0.92	(-1.52--0.33)	**2.38E-03**	**4.21E-03**	1.05	(0.38-1.71)	**2.10E-03**	**6.02E-03**	GUSTO and BIS	1.04	(0.53-1.54)	**6.15E-05**	**1.46E-04**
LPC(22:6) [sn2]	-0.08	(-0.94-0.8)	8.63E-01	8.94E-01	notSig	-0.46	(-0.97-0.05)	7.43E-02	9.88E-02	1.13	(0.57-1.69)	**7.67E-05**	**2.99E-04**	GUSTO and BIS	1.32	(0.89-1.76)	**2.75E-09**	**9.31E-09**
LPC(22:6) [sn1]	-0.27	(-1.38-0.85)	6.34E-01	7.04E-01	notSig	-0.17	(-0.73-0.4)	5.62E-01	6.23E-01	1.43	(0.8-2.06)	**9.31E-06**	**4.55E-05**	GUSTO and BIS	1.70	(1.22-2.18)	**3.29E-12**	**1.39E-11**
LPC(O-16:0)	-0.71	(-1.72-0.31)	1.72E-01	2.47E-01	notSig	-0.38	(-0.78-0.03)	6.76E-02	9.07E-02	1.22	(0.7-1.73)	**3.81E-06**	**2.05E-05**	GUSTO and BIS	1.47	(1.11-1.84)	**2.33E-15**	**1.20E-14**

LPC(O-18:0)	-1.41	(-2.38--0.42)	**5.26E-03**	**1.33E-02**	GUSTO and BIS	-1.13	(-1.61--0.66)	**3.08E-06**	**9.53E-06**	0.93	(0.4-1.46)	**6.44E-04**	**2.08E-03**	BIS only	0.63	(0.24-1.02)	**1.54E-03**	**3.02E-03**
LPC(O-18:1)	0.37	(-0.67-1.41)	4.87E-01	5.68E-01	GUSTO only	0.49	(0.08-0.91)	**1.91E-02**	**2.76E-02**	1.64	(1.13-2.15)	**4.02E-10**	**4.44E-09**	GUSTO and BIS	1.73	(1.36-2.1)	**2.94E-20**	**1.98E-19**
LPC(O-20:0)	-2.20	(-3.04--1.36)	**4.96E-07**	**5.01E-06**	GUSTO and BIS	-1.84	(-2.27--1.4)	**1.96E-16**	**3.63E-15**	0.94	(0.46-1.42)	**1.27E-04**	**4.82E-04**	BIS only	0.73	(0.31-1.14)	**6.82E-04**	**1.43E-03**
LPC(O-20:1)	-0.83	(-1.85-0.21)	1.16E-01	1.79E-01	notSig	-0.13	(-0.79-0.53)	6.96E-01	7.43E-01	1.51	(1-2.02)	**8.84E-09**	**7.46E-08**	GUSTO and BIS	1.74	(1.28-2.19)	**1.00E-13**	**4.74E-13**
LPC(O-22:0)	-2.49	(-3.23--1.75)	**1.17E-10**	**5.76E-09**	GUSTO and BIS	-2.43	(-2.83--2.02)	**1.03E-31**	**2.39E-29**	0.42	(0-0.85)	5.17E-02	9.67E-02	notSig	0.34	(0.01-0.66)	**4.09E-02**	6.23E-02
LPC(O-22:1)	-1.58	(-2.47--0.68)	**6.18E-04**	**2.19E-03**	GUSTO and BIS	-1.61	(-2.06--1.16)	**2.91E-12**	**2.50E-11**	1.50	(1.01-1.99)	**2.80E-09**	**2.55E-08**	GUSTO and BIS	1.41	(1.04-1.79)	**1.82E-13**	**8.37E-13**
LPC(O-24:0)	-3.03	(-3.89--2.17)	**1.85E-11**	**1.87E-09**	GUSTO and BIS	-3.04	(-3.52--2.55)	**3.77E-35**	**1.75E-32**	0.04	(-0.5-0.57)	8.87E-01	9.11E-01	notSig	-0.23	(-0.6-0.15)	2.33E-01	2.87E-01
LPC(O-24:1)	-1.89	(-2.69--1.08)	**6.47E-06**	**4.55E-05**	GUSTO and BIS	-2.25	(-2.71--1.8)	**3.96E-22**	**2.04E-20**	1.07	(0.61-1.54)	**7.35E-06**	**3.79E-05**	BIS only	0.81	(0.46-1.16)	**5.66E-06**	**1.47E-05**
LPC(O-24:2)	-2.24	(-3.13--1.33)	**1.70E-06**	**1.43E-05**	GUSTO and BIS	-2.03	(-2.53--1.53)	**9.99E-16**	**1.55E-14**	0.15	(-0.33-0.62)	5.47E-01	6.32E-01	notSig	0.17	(-0.19-0.53)	3.51E-01	4.08E-01
LPC(P-16:0)	-0.11	(-1.16-0.96)	8.40E-01	8.75E-01	notSig	0.34	(-0.1-0.77)	1.28E-01	1.62E-01	0.86	(0.38-1.34)	**4.97E-04**	**1.65E-03**	GUSTO and BIS	0.99	(0.64-1.33)	**1.78E-08**	**5.55E-08**
LPC(P-18:0)	-0.84	(-1.88-0.22)	1.20E-01	1.83E-01	notSig	0.22	(-0.2-0.63)	3.14E-01	3.62E-01	0.72	(0.2-1.24)	**6.31E-03**	**1.57E-02**	GUSTO and BIS	1.23	(0.86-1.6)	**5.74E-11**	**2.11E-10**
LPC(P-18:1)	0.22	(-0.87-1.33)	6.94E-01	7.59E-01	GUSTO only	0.79	(0.27-1.31)	**3.03E-03**	**5.26E-03**	1.12	(0.59-1.64)	**3.33E-05**	**1.46E-04**	GUSTO and BIS	1.17	(0.75-1.58)	**3.54E-08**	**1.08E-07**
LPC(P-20:0)	-1.26	(-1.97--0.56)	**5.13E-04**	**1.91E-03**	GUSTO and BIS	-0.94	(-1.39--0.5)	**3.48E-05**	**8.84E-05**	0.62	(0.18-1.06)	**6.04E-03**	**1.53E-02**	GUSTO and BIS	1.03	(0.66-1.4)	**4.35E-08**	**1.31E-07**
LPE(16:0) [sn2]	0.33	(-0.67-1.35)	5.16E-01	5.94E-01	GUSTO only	-0.40	(-0.85-0.05)	8.42E-02	1.11E-01	1.64	(1.17-2.12)	**2.77E-11**	**3.67E-10**	GUSTO and BIS	1.85	(1.46-2.23)	**8.01E-21**	**5.55E-20**
LPE(16:0) [sn1]	0.24	(-0.73-1.23)	6.26E-01	6.98E-01	notSig	-0.33	(-0.77-0.1)	1.35E-01	1.70E-01	1.61	(1.15-2.07)	**1.20E-11**	**1.69E-10**	GUSTO and BIS	1.83	(1.46-2.2)	**2.07E-22**	**1.57E-21**
LPE(18:0) [sn2]	0.52	(-0.52-1.56)	3.27E-01	4.19E-01	notSig	0.24	(-0.2-0.67)	2.87E-01	3.38E-01	1.26	(0.78-1.74)	**2.72E-07**	**1.94E-06**	GUSTO and BIS	1.32	(0.96-1.68)	**5.11E-13**	**2.30E-12**
LPE(18:0) [sn1]	0.74	(-0.28-1.76)	1.55E-01	2.27E-01	notSig	0.46	(0.04-0.87)	**2.99E-02**	**4.24E-02**	1.21	(0.72-1.69)	**1.18E-06**	**7.21E-06**	GUSTO and BIS	1.36	(1-1.72)	**1.32E-13**	**6.11E-13**
LPE(18:1) [sn2]	-1.14	(-2.04--0.24)	**1.36E-02**	**2.98E-02**	GUSTO and BIS	-0.88	(-1.35--0.41)	**2.22E-04**	**4.84E-04**	2.73	(2.2-3.25)	**5.46E-23**	**1.19E-20**	GUSTO and BIS	2.66	(2.24-3.07)	**4.00E-36**	**7.14E-35**
LPE(18:1) [sn1]	-1.23	(-2.15--0.3)	**9.49E-03**	**2.17E-02**	GUSTO and BIS	-0.87	(-1.33--0.4)	**2.63E-04**	**5.57E-04**	2.60	(2.07-3.13)	**1.36E-20**	**1.27E-18**	GUSTO and BIS	2.72	(2.3-3.15)	**1.57E-36**	**2.91E-35**
LPE(18:2) [sn2]	-2.31	(-3.32--1.3)	**1.04E-05**	**6.59E-05**	GUSTO and BIS	-1.35	(-1.9--0.79)	**1.93E-06**	**6.11E-06**	3.21	(2.46-3.97)	**3.01E-16**	**8.74E-15**	GUSTO and BIS	3.37	(2.78-3.97)	**1.44E-28**	**1.55E-27**
LPE(18:2) [sn1]	-2.50	(-3.58--1.41)	**9.53E-06**	**6.23E-05**	GUSTO and BIS	-1.39	(-1.96--0.82)	**1.94E-06**	**6.11E-06**	3.28	(2.49-4.06)	**9.07E-16**	**2.34E-14**	GUSTO and BIS	3.47	(2.87-4.07)	**3.74E-30**	**4.45E-29**
LPE(20:4) [sn1]	0.57	(-0.4-1.55)	2.47E-01	3.35E-01	notSig	0.57	(0.1-1.04)	**1.80E-02**	**2.65E-02**	2.92	(2.13-3.71)	**8.91E-13**	**1.42E-11**	GUSTO and BIS	3.04	(2.49-3.59)	**2.81E-27**	**2.90E-26**
LPE(22:6) [sn2]	1.32	(0.22-2.44)	**1.86E-02**	**3.84E-02**	BIS only	0.00	(-0.52-0.52)	9.92E-01	9.94E-01	1.76	(0.86-2.66)	**1.31E-04**	**4.94E-04**	GUSTO and BIS	1.97	(1.4-2.54)	**1.14E-11**	**4.55E-11**
LPE(22:6) [sn1]	0.85	(-0.22-1.93)	1.21E-01	1.83E-01	notSig	-0.02	(-0.57-0.52)	9.31E-01	9.44E-01	1.91	(1.07-2.75)	**8.81E-06**	**4.35E-05**	GUSTO and BIS	2.16	(1.6-2.71)	**2.23E-14**	**1.10E-13**
PC(28:0)	-4.68	(-6.07--3.26)	**3.29E-10**	**1.22E-08**	GUSTO and BIS	-3.45	(-4.31--2.59)	**3.66E-15**	**5.14E-14**	0.39	(-0.36-1.14)	3.06E-01	4.09E-01	notSig	-0.02	(-0.49-0.46)	9.45E-01	9.49E-01
PC(14:0_16:0)	-2.51	(-3.49--1.53)	**8.07E-07**	**7.20E-06**	GUSTO and BIS	-2.30	(-2.87--1.72)	**5.36E-15**	**7.31E-14**	0.19	(-0.59-0.98)	6.28E-01	7.10E-01	notSig	-0.03	(-0.54-0.49)	9.24E-01	9.36E-01
PC(31:0) (a)	-2.80	(-3.99--1.6)	**6.84E-06**	**4.67E-05**	GUSTO and BIS	-3.07	(-3.86--2.27)	**3.15E-14**	**3.57E-13**	-0.64	(-1.49-0.2)	1.35E-01	2.09E-01	notSig	-0.60	(-1.21-0.02)	5.72E-02	8.27E-02
PC(31:0) (b)	-1.34	(-2.09--0.59)	**5.43E-04**	**1.97E-03**	GUSTO and BIS	-1.89	(-2.37--1.41)	**6.56E-15**	**8.63E-14**	-0.67	(-1.4-0.06)	7.16E-02	1.25E-01	notSig	-0.80	(-1.37--0.24)	**5.49E-03**	**1.01E-02**
PC(16:0_16:0)	-0.40	(-1.07-0.28)	2.51E-01	3.37E-01	GUSTO only	-0.58	(-0.98--0.18)	**4.92E-03**	**8.24E-03**	-0.14	(-0.9-0.62)	7.13E-01	7.84E-01	notSig	-0.39	(-0.88-0.1)	1.18E-01	1.60E-01
PC(32:1)	-0.42	(-1.51-0.68)	4.52E-01	5.34E-01	notSig	-0.71	(-1.37--0.04)	**3.65E-02**	5.10E-02	1.46	(0.74-2.19)	**7.35E-05**	**2.89E-04**	GUSTO and BIS	1.25	(0.7-1.8)	**9.54E-06**	**2.41E-05**
PC(32:2)	-2.17	(-3.15--1.17)	**2.37E-05**	**1.33E-04**	GUSTO and BIS	-1.77	(-2.33--1.22)	**4.02E-10**	**2.39E-09**	0.62	(-0.29-1.54)	1.82E-01	2.71E-01	notSig	0.27	(-0.27-0.82)	3.24E-01	3.84E-01
PC(33:0) (a)	-0.43	(-1.29-0.43)	3.28E-01	4.19E-01	GUSTO only	-1.47	(-2.04--0.91)	**3.59E-07**	**1.37E-06**	-0.80	(-1.6-0.01)	5.16E-02	9.67E-02	notSig	-0.79	(-1.39--0.2)	**9.27E-03**	**1.61E-02**
PC(33:0) (b)	-0.60	(-1.25-0.05)	6.96E-02	1.17E-01	GUSTO only	-1.28	(-1.7--0.85)	**3.92E-09**	**2.04E-08**	-0.88	(-1.6--0.16)	**1.73E-02**	**3.74E-02**	GUSTO and BIS	-1.15	(-1.67--0.63)	**1.49E-05**	**3.72E-05**
PC(33:1)	-0.46	(-1.33-0.43)	3.10E-01	4.02E-01	GUSTO only	-1.66	(-2.21--1.11)	**3.79E-09**	**2.00E-08**	0.09	(-0.77-0.95)	8.36E-01	8.82E-01	notSig	-0.35	(-0.85-0.16)	1.78E-01	2.28E-01
PC(33:2)	-0.83	(-1.53--0.12)	**2.21E-02**	**4.38E-02**	GUSTO and BIS	-1.63	(-2.1--1.16)	**9.66E-12**	**7.73E-11**	-0.84	(-1.79-0.1)	8.06E-02	1.35E-01	GUSTO only	-1.13	(-1.65--0.6)	**2.78E-05**	**6.79E-05**
PC(16:0_18:0)	-1.04	(-1.6--0.48)	**3.21E-04**	**1.22E-03**	GUSTO and BIS	-1.01	(-1.34--0.67)	**3.98E-09**	**2.05E-08**	0.07	(-0.52-0.66)	8.24E-01	8.75E-01	notSig	-0.10	(-0.51-0.31)	6.35E-01	6.86E-01
PC(16:0_18:1)	0.19	(-0.19-0.57)	3.31E-01	4.21E-01	GUSTO only	-0.22	(-0.54-0.09)	1.65E-01	2.05E-01	0.90	(0.36-1.44)	**1.07E-03**	**3.29E-03**	BIS only	0.60	(0.17-1.03)	**6.00E-03**	**1.08E-02**
PC(16:0_18:2)	-0.04	(-0.34-0.25)	7.70E-01	8.21E-01	GUSTO only	-0.19	(-0.4-0.02)	8.34E-02	1.10E-01	0.20	(-0.4-0.8)	5.12E-01	6.06E-01	notSig	-0.07	(-0.56-0.42)	7.88E-01	8.30E-01
PC(16:1_18:2)	-0.45	(-1.31-0.41)	2.99E-01	3.90E-01	GUSTO only	-0.72	(-1.23--0.21)	**5.46E-03**	**9.06E-03**	0.21	(-0.68-1.1)	6.43E-01	7.22E-01	notSig	-0.42	(-0.94-0.1)	1.12E-01	1.53E-01
PC(16:0_18:3) (a)	-1.99	(-2.94--1.04)	**5.17E-05**	**2.50E-04**	GUSTO and BIS	-1.85	(-2.39--1.31)	**1.69E-11**	**1.28E-10**	-0.34	(-1.4-0.72)	5.30E-01	6.18E-01	notSig	-0.09	(-0.74-0.56)	7.81E-01	8.25E-01
PC(14:0_20:4)	-1.32	(-2.33--0.29)	**1.21E-02**	**2.69E-02**	BIS only	-0.94	(-1.56--0.32)	**2.75E-03**	**4.84E-03**	0.74	(-0.04-1.53)	6.46E-02	1.15E-01	notSig	0.18	(-0.3-0.66)	4.51E-01	5.05E-01
PC(34:5)	-2.45	(-3.87--1.01)	**9.36E-04**	**3.00E-03**	BIS only	-1.70	(-2.61--0.79)	**2.50E-04**	**5.34E-04**	-0.53	(-1.48-0.41)	2.66E-01	3.66E-01	GUSTO only	-0.88	(-1.5--0.26)	**5.70E-03**	**1.03E-02**
PC(15-MHDA_18:1)	0.22	(-0.59-1.04)	5.93E-01	6.67E-01	GUSTO only	-1.18	(-1.75--0.62)	**4.51E-05**	**1.11E-04**	-0.73	(-1.55-0.1)	8.35E-02	1.39E-01	GUSTO only	-1.01	(-1.57--0.45)	**4.28E-04**	**9.24E-04**
PC(17:0_18:1)	0.02	(-0.75-0.8)	9.58E-01	9.66E-01	GUSTO only	-1.38	(-1.8--0.95)	**2.17E-10**	**1.36E-09**	-0.48	(-1.2-0.25)	1.96E-01	2.87E-01	GUSTO only	-0.87	(-1.28--0.45)	**4.19E-05**	**1.01E-04**
PC(15-MHDA_18:2)	0.01	(-0.9-0.92)	9.86E-01	9.88E-01	GUSTO only	-1.03	(-1.64--0.43)	**8.42E-04**	**1.58E-03**	-1.27	(-2.34--0.2)	**2.01E-02**	**4.26E-02**	GUSTO and BIS	-1.35	(-1.94--0.76)	**6.66E-06**	**1.71E-05**
PC(17:0_18:2)	-0.35	(-1.19-0.49)	4.12E-01	4.96E-01	GUSTO only	-1.20	(-1.65--0.74)	**2.56E-07**	**9.97E-07**	-0.94	(-1.91-0.04)	5.99E-02	1.09E-01	GUSTO only	-1.19	(-1.69--0.69)	**2.85E-06**	**7.51E-06**
PC(17:1_18:2)	-0.82	(-1.51--0.14)	**1.89E-02**	**3.86E-02**	GUSTO and BIS	-1.06	(-1.49--0.64)	**8.76E-07**	**2.88E-06**	-0.75	(-1.59-0.08)	7.59E-02	1.31E-01	GUSTO only	-0.73	(-1.2--0.27)	**2.08E-03**	**4.03E-03**
PC(15:0_20:3)	-0.99	(-1.64--0.34)	**3.00E-03**	**8.16E-03**	GUSTO and BIS	-0.63	(-0.87--0.39)	**2.98E-07**	**1.14E-06**	-0.32	(-1.04-0.4)	3.82E-01	4.87E-01	notSig	-0.22	(-0.81-0.37)	4.61E-01	5.14E-01
PC(15:0_20:4)	0.76	(-0.05-1.58)	6.46E-02	1.11E-01	GUSTO only	-0.41	(-0.94-0.12)	1.26E-01	1.59E-01	-0.49	(-1.2-0.22)	1.72E-01	2.59E-01	GUSTO only	-0.99	(-1.44--0.54)	**1.70E-05**	**4.19E-05**
PC(35:5)	-0.98	(-2.36-0.41)	1.66E-01	2.40E-01	GUSTO only	-1.31	(-2.23--0.39)	**5.47E-03**	**9.06E-03**	-1.77	(-2.84--0.71)	**1.13E-03**	**3.45E-03**	GUSTO and BIS	-2.11	(-2.78--1.44)	**7.15E-10**	**2.50E-09**
PC(18:0_18:1)	-1.19	(-1.88--0.5)	**8.08E-04**	**2.68E-03**	GUSTO and BIS	-1.28	(-1.71--0.85)	**7.15E-09**	**3.49E-08**	1.22	(0.63-1.81)	**5.51E-05**	**2.26E-04**	BIS only	0.84	(0.42-1.26)	**7.52E-05**	**1.77E-04**
PC(18:0_18:2)	-0.64	(-1.03--0.25)	**1.46E-03**	**4.41E-03**	GUSTO and BIS	-0.63	(-0.95--0.31)	**9.38E-05**	**2.21E-04**	0.28	(-0.39-0.95)	4.10E-01	5.10E-01	notSig	-0.10	(-0.57-0.37)	6.78E-01	7.26E-01

PC(16:0_20:3) (a)	-0.46	(-1.03-0.11)	1.14E-01	1.76E-01	GUSTO only	-0.83	(-1.24--0.42)	**6.66E-05**	**1.59E-04**	0.95	(0.33-1.57)	**2.84E-03**	**7.66E-03**	BIS only	0.36	(-0.1-0.83)	1.27E-01	1.71E-01
PC(16:0_20:3) (b)	-1.26	(-2.19--0.32)	**8.79E-03**	**2.02E-02**	BIS only	-0.09	(-0.68-0.5)	7.59E-01	7.99E-01	3.62	(2.8-4.44)	**2.32E-17**	**8.96E-16**	BIS only	1.59	(1.08-2.1)	**8.24E-10**	**2.85E-09**
PC(18:2_18:2)	-2.91	(-3.7--2.11)	**3.37E-12**	**5.22E-10**	GUSTO and BIS	-2.72	(-3.25--2.19)	**5.05E-24**	**2.93E-22**	0.49	(-0.43-1.4)	2.97E-01	3.99E-01	notSig	0.43	(-0.23-1.09)	2.06E-01	2.59E-01
PC(16:1_20:4)	0.28	(-0.51-1.08)	4.90E-01	5.70E-01	notSig	-0.12	(-0.64-0.39)	6.39E-01	6.89E-01	0.98	(0.35-1.61)	**2.30E-03**	**6.52E-03**	BIS only	0.52	(0.07-0.97)	**2.38E-02**	**3.85E-02**
PC(16:0_20:5)	0.04	(-1.11-1.2)	9.49E-01	9.64E-01	notSig	-0.10	(-0.94-0.73)	8.07E-01	8.36E-01	-0.81	(-1.71-0.09)	7.88E-02	1.34E-01	GUSTO only	-1.35	(-1.98--0.72)	**2.54E-05**	**6.23E-05**
PC(15-MHDA_20:4)	1.05	(0.36-1.74)	**2.70E-03**	**7.53E-03**	BIS only	0.30	(-0.22-0.81)	2.60E-01	3.08E-01	-1.15	(-1.81--0.5)	**5.34E-04**	**1.76E-03**	GUSTO and BIS	-1.38	(-1.85--0.91)	**9.74E-09**	**3.05E-08**
PC(17:0_20:4)	1.30	(0.57-2.04)	**5.05E-04**	**1.89E-03**	BIS only	0.38	(-0.1-0.86)	1.25E-01	1.59E-01	-1.35	(-1.9--0.8)	**1.68E-06**	**9.75E-06**	GUSTO and BIS	-1.54	(-1.92--1.17)	**6.96E-16**	**3.71E-15**
PC(15:0_22:6)	-0.74	(-1.59-0.12)	9.04E-02	1.46E-01	GUSTO only	-1.86	(-2.41--1.3)	**6.72E-11**	**4.65E-10**	-1.40	(-2.16--0.63)	**3.66E-04**	**1.26E-03**	GUSTO and BIS	-1.71	(-2.17--1.24)	**5.20E-13**	**2.32E-12**
PC(38:2)	-1.40	(-1.99--0.81)	**4.63E-06**	**3.52E-05**	GUSTO and BIS	-1.23	(-1.59--0.87)	**2.15E-11**	**1.58E-10**	1.28	(0.78-1.78)	**5.73E-07**	**3.75E-06**	GUSTO and BIS	1.14	(0.75-1.52)	**8.45E-09**	**2.69E-08**
PC(18:0_20:3)	-0.59	(-1.34-0.17)	1.26E-01	1.89E-01	GUSTO only	0.39	(-0.12-0.9)	1.37E-01	1.73E-01	0.56	(0.08-1.05)	**2.18E-02**	**4.56E-02**	BIS only	0.38	(0.01-0.75)	**4.33E-02**	6.53E-02
PC(18:1_20:3)	-0.77	(-1.5--0.04)	**3.98E-02**	7.21E-02	notSig	-0.66	(-1.13--0.19)	**5.77E-03**	**9.49E-03**	1.35	(0.71-2)	**4.08E-05**	**1.72E-04**	BIS only	0.78	(0.33-1.23)	**6.66E-04**	**1.40E-03**
PC(38:4) (b)	-1.31	(-2.05--0.56)	**6.54E-04**	**2.25E-03**	GUSTO and BIS	-1.13	(-1.63--0.62)	**1.26E-05**	**3.52E-05**	0.55	(0.05-1.06)	**3.07E-02**	6.14E-02	notSig	0.35	(-0.07-0.77)	1.02E-01	1.40E-01
PC(18:0_20:4)	1.26	(0.63-1.91)	**1.01E-04**	**4.47E-04**	GUSTO and BIS	1.12	(0.67-1.57)	**1.02E-06**	**3.25E-06**	-0.34	(-0.8-0.12)	1.49E-01	2.28E-01	GUSTO only	-0.61	(-0.98--0.25)	**9.84E-04**	**2.02E-03**
PC(38:5) (a)	0.26	(-0.28-0.8)	3.46E-01	4.35E-01	GUSTO only	-0.47	(-0.83--0.1)	**1.34E-02**	**2.04E-02**	-0.03	(-0.53-0.47)	9.05E-01	9.27E-01	GUSTO only	-0.58	(-0.94--0.21)	**1.76E-03**	**3.41E-03**
PC(38:5) (b)	-0.57	(-1.24-0.1)	9.65E-02	1.53E-01	GUSTO only	-0.88	(-1.37--0.39)	**4.74E-04**	**9.37E-04**	0.88	(0.33-1.44)	**1.68E-03**	**4.95E-03**	BIS only	0.58	(0.15-1.01)	**7.95E-03**	**1.39E-02**
PC(38:6) (a)	-0.86	(-1.53--0.19)	**1.19E-02**	**2.67E-02**	GUSTO and BIS	-0.95	(-1.39--0.5)	**3.04E-05**	**7.90E-05**	0.44	(-0.31-1.19)	2.48E-01	3.49E-01	notSig	0.23	(-0.3-0.75)	3.93E-01	4.51E-01
PC(16:0_22:6)	0.05	(-0.47-0.59)	8.42E-01	8.75E-01	GUSTO only	-0.38	(-0.79-0.02)	6.43E-02	8.68E-02	-0.50	(-0.95--0.04)	**3.14E-02**	6.25E-02	GUSTO only	-0.71	(-1.09--0.33)	**2.65E-04**	**5.90E-04**
PC(18:2_20:5)	-1.97	(-3.01--0.91)	**2.88E-04**	**1.11E-03**	GUSTO and BIS	-1.86	(-2.57--1.14)	**3.62E-07**	**1.37E-06**	0.23	(-0.63-1.08)	6.01E-01	6.83E-01	notSig	-0.23	(-0.82-0.36)	4.48E-01	5.03E-01
PC(16:1_22:6)	-0.18	(-0.96-0.61)	6.57E-01	7.22E-01	GUSTO only	-1.01	(-1.52--0.49)	**1.26E-04**	**2.90E-04**	-0.19	(-0.8-0.41)	5.28E-01	6.17E-01	GUSTO only	-0.66	(-1.07--0.26)	**1.33E-03**	**2.63E-03**
PC(38:7)(c)	0.06	(-0.68-0.81)	8.75E-01	9.01E-01	GUSTO only	-0.95	(-1.41--0.49)	**5.83E-05**	**1.40E-04**	-0.34	(-0.94-0.26)	2.62E-01	3.63E-01	GUSTO only	-0.66	(-1.04--0.27)	**7.68E-04**	**1.58E-03**
PC(39:5)(a)	-0.60	(-1.43-0.24)	1.62E-01	2.36E-01	GUSTO only	-1.57	(-2.16--0.99)	**1.49E-07**	**6.13E-07**	-1.86	(-2.59--1.13)	**8.03E-07**	**5.01E-06**	GUSTO and BIS	-1.93	(-2.42--1.43)	**2.05E-14**	**1.02E-13**
PC(39:5)(b)	-0.42	(-1.1-0.27)	2.33E-01	3.19E-01	GUSTO only	-1.30	(-1.8--0.8)	**3.85E-07**	**1.42E-06**	-1.07	(-1.72--0.43)	**1.03E-03**	**3.18E-03**	GUSTO and BIS	-1.52	(-1.97--1.07)	**3.30E-11**	**1.24E-10**
PC(15-MHDA_22:6)	0.12	(-0.79-1.04)	7.99E-01	8.45E-01	GUSTO only	-1.36	(-1.97--0.76)	**1.06E-05**	**2.99E-05**	-2.37	(-3.11--1.64)	**3.98E-10**	**4.44E-09**	GUSTO and BIS	-2.21	(-2.71--1.71)	**4.68E-18**	**2.72E-17**
PC(17:0_22:6)	-0.17	(-0.99-0.65)	6.75E-01	7.41E-01	GUSTO only	-1.18	(-1.73--0.63)	**2.49E-05**	**6.69E-05**	-1.94	(-2.61--1.27)	**1.81E-08**	**1.50E-07**	GUSTO and BIS	-2.12	(-2.55--1.68)	**6.87E-22**	**5.14E-21**
PC(18:0_22:5) (n6)	-1.13	(-2.15--0.11)	**3.01E-02**	5.80E-02	GUSTO only	-1.25	(-1.86--0.65)	**4.66E-05**	**1.14E-04**	1.74	(1.01-2.47)	**3.76E-06**	**2.05E-05**	BIS only	0.94	(0.31-1.57)	**3.28E-03**	**6.18E-03**
PC(18:0_22:6)	-0.20	(-0.92-0.52)	5.77E-01	6.54E-01	notSig	-0.41	(-0.92-0.09)	1.11E-01	1.42E-01	-0.82	(-1.39--0.25)	**4.70E-03**	**1.23E-02**	GUSTO and BIS	-1.08	(-1.51--0.64)	**1.23E-06**	**3.32E-06**
PC(40:7) (a)	-1.26	(-2.07--0.45)	**2.30E-03**	**6.51E-03**	GUSTO and BIS	-1.33	(-1.82--0.83)	**1.57E-07**	**6.30E-07**	1.75	(1.08-2.42)	**3.58E-07**	**2.44E-06**	BIS only	1.49	(0.94-2.05)	**1.16E-07**	**3.41E-07**
PC(18:1_22:6) (a)	-0.62	(-1.26-0.03)	6.33E-02	1.10E-01	GUSTO only	-1.25	(-1.67--0.84)	**3.36E-09**	**1.79E-08**	-0.08	(-0.58-0.42)	7.45E-01	8.07E-01	notSig	-0.21	(-0.61-0.2)	3.18E-01	3.77E-01
PC(40:8)	-1.39	(-2.06--0.72)	**5.91E-05**	**2.77E-04**	GUSTO and BIS	-1.62	(-2.04--1.2)	**2.37E-14**	**2.82E-13**	1.18	(0.54-1.83)	**3.61E-04**	**1.25E-03**	GUSTO and BIS	1.01	(0.55-1.47)	**1.69E-05**	**4.19E-05**
PC(44:12)	-1.58	(-2.86--0.27)	**1.81E-02**	**3.76E-02**	GUSTO and BIS	-2.07	(-2.89--1.25)	**6.67E-07**	**2.26E-06**	-0.84	(-1.54--0.14)	**1.90E-02**	**4.07E-02**	GUSTO and BIS	-0.92	(-1.44--0.4)	**4.94E-04**	**1.06E-03**
PC(O-16:0/16:0)	-1.28	(-1.95--0.61)	**2.19E-04**	**8.67E-04**	GUSTO and BIS	-0.93	(-1.29--0.57)	**5.07E-07**	**1.80E-06**	0.75	(0.05-1.44)	**3.63E-02**	7.11E-02	notSig	0.59	(0.11-1.07)	**1.58E-02**	**2.64E-02**
PC(O-32:1)	-1.65	(-2.49--0.8)	**1.47E-04**	**6.07E-04**	GUSTO and BIS	-1.20	(-1.66--0.74)	**3.71E-07**	**1.39E-06**	0.48	(-0.17-1.14)	1.49E-01	2.29E-01	notSig	0.59	(0.09-1.09)	**2.01E-02**	**3.30E-02**
PC(O-34:1)	-1.01	(-1.65--0.37)	**1.99E-03**	**5.76E-03**	GUSTO and BIS	-0.89	(-1.25--0.54)	**9.38E-07**	**3.06E-06**	1.00	(0.26-1.73)	**7.80E-03**	**1.90E-02**	BIS only	0.83	(0.33-1.33)	**1.13E-03**	**2.27E-03**
PC(O-34:2)	-2.65	(-3.43--1.86)	**1.09E-10**	**5.76E-09**	GUSTO and BIS	-2.25	(-2.73--1.77)	**3.67E-20**	**1.22E-18**	-0.02	(-0.77-0.73)	9.57E-01	9.63E-01	notSig	-0.04	(-0.57-0.48)	8.71E-01	8.97E-01
PC(O-34:4)	-1.10	(-1.88--0.31)	**6.50E-03**	**1.58E-02**	BIS only	-1.01	(-1.62--0.4)	**1.19E-03**	**2.18E-03**	0.08	(-0.67-0.84)	8.29E-01	8.78E-01	notSig	0.03	(-0.49-0.55)	9.05E-01	9.25E-01
PC(O-35:4)	-0.77	(-1.6-0.07)	7.30E-02	1.21E-01	notSig	-0.83	(-1.42--0.23)	**6.46E-03**	**1.05E-02**	-0.21	(-0.87-0.44)	5.24E-01	6.16E-01	notSig	-0.35	(-0.82-0.11)	1.37E-01	1.82E-01
PC(O-36:0)	-2.06	(-2.7--1.41)	**9.59E-10**	**2.34E-08**	GUSTO and BIS	-1.58	(-1.97--1.19)	**3.04E-15**	**4.41E-14**	0.34	(-0.29-0.98)	2.88E-01	3.89E-01	notSig	0.27	(-0.24-0.78)	3.00E-01	3.59E-01
PC(O-18:0/18:1)	-2.11	(-2.85--1.36)	**4.47E-08**	**6.48E-07**	GUSTO and BIS	-1.68	(-2.08--1.28)	**1.52E-16**	**2.94E-15**	1.03	(0.38-1.67)	**1.87E-03**	**5.46E-03**	GUSTO and BIS	1.16	(0.68-1.63)	**2.26E-06**	**6.02E-06**
PC(O-18:1/18:1)	-1.68	(-2.58--0.77)	**3.04E-04**	**1.16E-03**	GUSTO and BIS	-1.30	(-1.71--0.89)	**4.60E-10**	**2.70E-09**	1.46	(0.46-2.45)	**4.13E-03**	**1.09E-02**	GUSTO and BIS	0.92	(0.39-1.45)	**6.12E-04**	**1.30E-03**
PC(O-18:0/18:2)	-3.85	(-4.96--2.73)	**5.08E-11**	**3.37E-09**	GUSTO and BIS	-2.66	(-3.17--2.16)	**5.23E-25**	**3.47E-23**	0.44	(-0.59-1.47)	4.01E-01	5.07E-01	notSig	0.44	(-0.1-0.99)	1.12E-01	1.53E-01
PC(O-18:1/18:2)	-1.90	(-2.61--1.19)	**2.28E-07**	**2.75E-06**	GUSTO and BIS	-1.60	(-2.03--1.16)	**6.19E-13**	**5.74E-12**	0.47	(-0.24-1.17)	1.96E-01	2.87E-01	notSig	0.19	(-0.3-0.67)	4.49E-01	5.03E-01
PC(O-16:0/20:3)	-1.54	(-2.15--0.92)	**1.44E-06**	**1.24E-05**	BIS only	-0.94	(-1.34--0.54)	**4.09E-06**	**1.23E-05**	0.89	(0.31-1.47)	**2.49E-03**	**6.88E-03**	BIS only	0.69	(0.24-1.14)	**2.42E-03**	**4.60E-03**
PC(O-16:0/20:4)	-0.16	(-0.81-0.5)	6.34E-01	7.04E-01	notSig	-0.15	(-0.57-0.26)	4.62E-01	5.19E-01	0.73	(0.04-1.42)	**3.74E-02**	7.25E-02	notSig	0.23	(-0.23-0.68)	3.28E-01	3.87E-01
PC(O-36:5)	-1.22	(-2.18--0.26)	**1.31E-02**	**2.89E-02**	GUSTO and BIS	-1.17	(-1.86--0.48)	**8.81E-04**	**1.65E-03**	-0.32	(-1.06-0.43)	4.03E-01	5.07E-01	GUSTO only	-0.71	(-1.22--0.21)	**5.66E-03**	**1.03E-02**
PC(O-18:0/20:4)	-1.23	(-1.95--0.51)	**8.74E-04**	**2.86E-03**	BIS only	-0.68	(-1.1--0.26)	**1.41E-03**	**2.55E-03**	0.20	(-0.4-0.8)	5.12E-01	6.06E-01	notSig	-0.08	(-0.48-0.33)	7.13E-01	7.58E-01
PC(O-38:5)	0.23	(-0.31-0.77)	4.13E-01	4.97E-01	notSig	0.09	(-0.26-0.45)	6.06E-01	6.60E-01	0.70	(0.09-1.32)	**2.50E-02**	5.11E-02	notSig	0.32	(-0.14-0.78)	1.71E-01	2.21E-01
PC(O-16:0/22:6)	-0.65	(-1.35-0.05)	6.81E-02	1.15E-01	notSig	-0.44	(-0.93-0.05)	7.84E-02	1.04E-01	-0.15	(-0.74-0.44)	6.22E-01	7.06E-01	GUSTO only	-0.46	(-0.89--0.04)	**3.33E-02**	5.22E-02
PC(O-40:5)	-0.68	(-1.28--0.06)	**3.03E-02**	5.81E-02	GUSTO only	-0.75	(-1.14--0.37)	**1.31E-04**	**3.00E-04**	0.36	(-0.24-0.95)	2.38E-01	3.39E-01	notSig	0.07	(-0.32-0.47)	7.16E-01	7.60E-01
PC(O-18:0/22:6)	-1.39	(-2.1--0.66)	**1.87E-04**	**7.56E-04**	GUSTO and BIS	-0.95	(-1.4--0.51)	**2.61E-05**	**6.91E-05**	-0.49	(-1.03-0.05)	7.69E-02	1.31E-01	GUSTO only	-0.71	(-1.09--0.34)	**1.98E-04**	**4.45E-04**
PC(O-40:7) (a)	-0.02	(-0.71-0.67)	9.52E-01	9.65E-01	notSig	-0.04	(-0.52-0.43)	8.61E-01	8.84E-01	0.26	(-0.34-0.86)	3.91E-01	4.95E-01	GUSTO only	-0.24	(-0.66-0.18)	2.69E-01	3.26E-01
PC(P-16:0/14:0)	-2.41	(-3.13--1.69)	**1.37E-10**	**5.76E-09**	GUSTO and BIS	-1.73	(-2.19--1.28)	**9.24E-14**	**9.72E-13**	0.37	(-0.46-1.21)	3.82E-01	4.87E-01	notSig	-0.04	(-0.59-0.51)	8.92E-01	9.13E-01
PC(P-16:0/16:0)	-1.04	(-1.67--0.41)	**1.26E-03**	**3.83E-03**	GUSTO and BIS	-0.84	(-1.23--0.44)	**3.14E-05**	**8.08E-05**	0.28	(-0.48-1.04)	4.67E-01	5.66E-01	notSig	-0.17	(-0.67-0.32)	4.91E-01	5.45E-01

PC(P-16:0/16:1)	-0.98	(-1.64--0.32)	**3.77E-03**	**1.01E-02**	GUSTO and BIS	-0.83	(-1.28--0.39)	**2.26E-04**	**4.90E-04**	0.87	(0.13-1.61)	**2.21E-02**	**4.59E-02**	BIS only	0.39	(-0.12-0.9)	1.34E-01	1.80E-01
PC(P-16:0/18:0)	-2.11	(-3.14--1.07)	**8.57E-05**	**3.90E-04**	GUSTO and BIS	-1.32	(-1.68--0.95)	**2.10E-12**	**1.84E-11**	-0.19	(-1.26-0.88)	7.28E-01	7.97E-01	notSig	-0.23	(-0.71-0.26)	3.60E-01	4.16E-01
PC(P-16:0/18:1)	-1.46	(-2.14--0.77)	**3.88E-05**	**2.02E-04**	GUSTO and BIS	-1.31	(-1.73--0.88)	**1.61E-09**	**8.77E-09**	0.43	(-0.26-1.12)	2.24E-01	3.23E-01	notSig	0.03	(-0.43-0.48)	9.09E-01	9.25E-01
PC(P-16:0/18:2)	-1.90	(-2.69--1.11)	**3.14E-06**	**2.48E-05**	GUSTO and BIS	-1.38	(-1.86--0.91)	**8.71E-09**	**4.17E-08**	-0.93	(-1.87-0.02)	5.42E-02	1.00E-01	GUSTO only	-1.19	(-1.71--0.67)	**7.54E-06**	**1.92E-05**
PC(P-16:0/18:3)	-3.16	(-4.1--2.22)	**1.35E-10**	**5.76E-09**	GUSTO and BIS	-2.12	(-2.64--1.59)	**2.34E-15**	**3.50E-14**	-0.88	(-1.69--0.06)	**3.51E-02**	6.90E-02	notSig	-0.60	(-1.05--0.15)	**9.39E-03**	**1.62E-02**
PC(P-35:2)(a)	-2.69	(-3.8--1.57)	**3.32E-06**	**2.57E-05**	GUSTO and BIS	-2.43	(-3.11--1.74)	**4.02E-12**	**3.39E-11**	-1.45	(-2.56--0.33)	**1.10E-02**	**2.53E-02**	BIS only	-0.73	(-1.33--0.14)	**1.56E-02**	**2.61E-02**
PC(P-35:2)(b)	-2.44	(-3.55--1.32)	**2.37E-05**	**1.33E-04**	GUSTO and BIS	-2.01	(-2.62--1.4)	**8.01E-11**	**5.39E-10**	-1.49	(-2.74--0.24)	**1.94E-02**	**4.12E-02**	GUSTO and BIS	-1.46	(-2.03--0.89)	**4.47E-07**	**1.25E-06**
PC(P-15:0/20:4) (b)	-1.21	(-2.04--0.37)	**4.84E-03**	**1.24E-02**	BIS only	-0.88	(-1.41--0.35)	**1.03E-03**	**1.91E-03**	-0.56	(-1.09--0.02)	**4.06E-02**	7.82E-02	notSig	-0.63	(-1.06--0.2)	**4.46E-03**	**8.28E-03**
PC(P-18:1/18:1)	-1.46	(-2.15--0.76)	**4.81E-05**	**2.39E-04**	GUSTO and BIS	-1.29	(-1.71--0.88)	**1.12E-09**	**6.25E-09**	2.18	(1.51-2.84)	**2.66E-10**	**3.09E-09**	GUSTO and BIS	1.82	(1.29-2.35)	**1.46E-11**	**5.76E-11**
PC(P-18:0/18:2)	-2.51	(-3.46--1.55)	**4.05E-07**	**4.18E-06**	GUSTO and BIS	-2.09	(-2.58--1.6)	**1.04E-16**	**2.19E-15**	-0.52	(-1.46-0.42)	2.82E-01	3.84E-01	notSig	-0.61	(-1.09--0.13)	**1.23E-02**	**2.08E-02**
PC(P-16:0/20:4)	0.51	(-0.25-1.29)	1.89E-01	2.68E-01	notSig	0.47	(-0.02-0.95)	5.81E-02	7.93E-02	0.06	(-0.7-0.82)	8.76E-01	9.06E-01	GUSTO only	-0.68	(-1.14--0.22)	**3.87E-03**	**7.23E-03**
PC(P-16:0/20:5)	-0.88	(-1.95-0.2)	1.10E-01	1.71E-01	notSig	-0.69	(-1.44-0.05)	6.92E-02	9.26E-02	-1.12	(-1.92--0.33)	**5.76E-03**	**1.48E-02**	GUSTO and BIS	-1.58	(-2.11--1.04)	**6.68E-09**	**2.18E-08**
PC(P-17:0/20:4) (a)	-0.26	(-1.01-0.49)	4.97E-01	5.75E-01	GUSTO only	-0.62	(-1.16--0.07)	**2.67E-02**	**3.81E-02**	-1.26	(-1.93--0.6)	**2.25E-04**	**8.10E-04**	GUSTO and BIS	-1.41	(-1.89--0.94)	**6.15E-09**	**2.02E-08**
PC(P-17:0/20:4) (b)	0.04	(-0.72-0.8)	9.25E-01	9.45E-01	notSig	-0.15	(-0.67-0.38)	5.86E-01	6.42E-01	-1.05	(-1.65--0.45)	**6.31E-04**	**2.05E-03**	GUSTO and BIS	-1.47	(-1.89--1.04)	**1.23E-11**	**4.90E-11**
PC(P-18:0/20:4)	0.11	(-0.73-0.95)	8.04E-01	8.48E-01	notSig	-0.03	(-0.51-0.45)	9.16E-01	9.31E-01	-0.30	(-1.02-0.41)	4.05E-01	5.07E-01	GUSTO only	-0.82	(-1.24--0.4)	**1.43E-04**	**3.24E-04**
PC(P-38:5) (a)	0.80	(0.21-1.39)	**7.78E-03**	**1.82E-02**	BIS only	0.53	(0.1-0.95)	**1.44E-02**	**2.17E-02**	-0.16	(-0.83-0.51)	6.43E-01	7.22E-01	GUSTO only	-0.78	(-1.21--0.35)	**4.00E-04**	**8.68E-04**
PC(P-38:5) (b)	-0.54	(-1.26-0.19)	1.44E-01	2.14E-01	GUSTO only	-0.73	(-1.18--0.27)	**1.65E-03**	**2.97E-03**	0.41	(-0.26-1.09)	2.28E-01	3.26E-01	GUSTO only	-0.26	(-0.72-0.21)	2.83E-01	3.40E-01
PC(P-16:0/22:6)	-0.38	(-1.13-0.37)	3.15E-01	4.05E-01	notSig	-0.34	(-0.85-0.18)	2.03E-01	2.47E-01	-0.67	(-1.36-0.01)	5.32E-02	9.87E-02	GUSTO only	-1.16	(-1.61--0.71)	**3.72E-07**	**1.05E-06**
PC(P-20:0/20:4)	-0.55	(-1.24-0.14)	1.21E-01	1.83E-01	notSig	-0.48	(-0.94--0.03)	**3.68E-02**	5.12E-02	-0.27	(-0.8-0.26)	3.21E-01	4.25E-01	GUSTO only	-0.52	(-0.92--0.12)	**1.01E-02**	**1.73E-02**
PC(P-18:0/22:6)	-0.76	(-1.56-0.05)	6.57E-02	1.13E-01	notSig	-0.72	(-1.24--0.19)	**7.23E-03**	**1.16E-02**	-0.91	(-1.57--0.25)	**7.08E-03**	**1.75E-02**	GUSTO and BIS	-1.31	(-1.72--0.9)	**4.85E-10**	**1.70E-09**
PE(16:0_16:1)	-0.24	(-1.79-1.33)	7.62E-01	8.15E-01	notSig	-0.43	(-1.36-0.5)	3.61E-01	4.13E-01	1.33	(0.29-2.38)	**1.21E-02**	**2.75E-02**	BIS only	0.90	(0.15-1.65)	**1.81E-02**	**3.01E-02**
PE(16:0_18:1)	0.82	(-0.18-1.83)	1.10E-01	1.71E-01	notSig	0.24	(-0.37-0.85)	4.47E-01	5.06E-01	-0.11	(-1.32-1.11)	8.64E-01	8.98E-01	notSig	-0.47	(-1.09-0.15)	1.34E-01	1.80E-01
PE(16:0_18:2)	-0.39	(-1.39-0.63)	4.52E-01	5.34E-01	notSig	-0.35	(-1-0.31)	3.00E-01	3.49E-01	-0.37	(-1.48-0.75)	5.16E-01	6.08E-01	notSig	-0.58	(-1.2-0.04)	6.61E-02	9.35E-02
PE(16:1_18:2)	-1.14	(-2.49-0.23)	1.01E-01	1.60E-01	notSig	-1.02	(-1.83--0.2)	**1.44E-02**	**2.17E-02**	0.69	(-0.43-1.8)	2.27E-01	3.26E-01	notSig	0.49	(-0.17-1.14)	1.47E-01	1.93E-01
PE(16:0_18:3) (a)	-1.97	(-3.3--0.62)	**4.51E-03**	**1.18E-02**	GUSTO and BIS	-1.73	(-2.49--0.97)	**8.28E-06**	**2.36E-05**	-0.48	(-2.12-1.15)	5.62E-01	6.44E-01	notSig	-0.04	(-0.77-0.68)	9.09E-01	9.25E-01
PE(16:0_18:3) (b)	-1.64	(-2.88--0.38)	**1.09E-02**	**2.46E-02**	BIS only	-0.84	(-1.58--0.11)	**2.48E-02**	**3.55E-02**	0.89	(-0.09-1.88)	7.61E-02	1.31E-01	notSig	0.71	(0.04-1.38)	**3.70E-02**	5.70E-02
PE(17:0_18:2)	-0.60	(-1.55-0.36)	2.21E-01	3.05E-01	GUSTO only	-1.00	(-1.56--0.44)	**4.62E-04**	**9.20E-04**	-1.70	(-2.81--0.59)	**2.73E-03**	**7.50E-03**	GUSTO and BIS	-1.00	(-1.47--0.53)	**3.16E-05**	**7.67E-05**
PE(18:0_18:1)	-0.39	(-1.31-0.54)	4.15E-01	4.98E-01	notSig	0.16	(-0.37-0.69)	5.45E-01	6.05E-01	-0.34	(-1.5-0.82)	5.68E-01	6.49E-01	notSig	-0.17	(-0.84-0.49)	6.09E-01	6.64E-01
PE(18:1_18:1)	-1.29	(-2.36--0.21)	**1.93E-02**	**3.93E-02**	BIS only	-0.64	(-1.21--0.08)	**2.56E-02**	**3.65E-02**	0.12	(-1.16-1.4)	8.50E-01	8.91E-01	notSig	0.05	(-0.79-0.89)	9.14E-01	9.28E-01
PE(18:0_18:2)	-0.42	(-1.4-0.57)	4.00E-01	4.86E-01	notSig	0.18	(-0.4-0.76)	5.42E-01	6.03E-01	-0.59	(-1.8-0.61)	3.33E-01	4.37E-01	notSig	-0.52	(-1.13-0.08)	8.79E-02	1.22E-01
PE(18:1_18:2)	-1.76	(-2.8--0.71)	**1.07E-03**	**3.34E-03**	BIS only	-1.15	(-1.79--0.5)	**4.64E-04**	**9.20E-04**	-0.12	(-1.4-1.16)	8.56E-01	8.94E-01	notSig	-0.22	(-0.89-0.45)	5.24E-01	5.78E-01
PE(16:0_20:3)	-0.96	(-1.91--0.01)	**4.88E-02**	8.70E-02	notSig	-0.63	(-1.26-0)	**4.96E-02**	6.81E-02	-0.15	(-1.12-0.82)	7.60E-01	8.20E-01	notSig	-0.25	(-0.85-0.36)	4.25E-01	4.81E-01
PE(16:0_20:4)	1.51	(0.59-2.43)	**1.21E-03**	**3.75E-03**	BIS only	1.05	(0.47-1.63)	**3.62E-04**	**7.47E-04**	-0.10	(-0.8-0.59)	7.69E-01	8.28E-01	notSig	-0.23	(-0.7-0.24)	3.43E-01	4.02E-01
PE(16:1_20:4)	0.65	(-0.52-1.84)	2.76E-01	3.68E-01	notSig	0.13	(-0.59-0.84)	7.26E-01	7.70E-01	1.17	(0.38-1.96)	**3.87E-03**	**1.03E-02**	BIS only	0.97	(0.39-1.54)	**1.09E-03**	**2.20E-03**
PE(16:0_20:5)	-0.58	(-1.88-0.74)	3.86E-01	4.74E-01	notSig	-0.51	(-1.33-0.3)	2.19E-01	2.65E-01	-0.96	(-1.95-0.04)	5.87E-02	1.07E-01	notSig	-0.87	(-1.5--0.25)	**5.70E-03**	**1.03E-02**
PE(17:0_20:4)	1.72	(0.9-2.55)	**3.79E-05**	**2.00E-04**	BIS only	0.67	(0.15-1.19)	**1.14E-02**	**1.75E-02**	-1.43	(-2.16--0.7)	**1.25E-04**	**4.81E-04**	GUSTO and BIS	-1.59	(-2.06--1.12)	**2.71E-11**	**1.04E-10**
PE(18:0_20:3) (a)	0.32	(-0.67-1.31)	5.30E-01	6.08E-01	GUSTO only	1.13	(0.5-1.76)	**4.28E-04**	**8.68E-04**	-0.39	(-1.23-0.45)	3.66E-01	4.72E-01	notSig	-0.52	(-1.04-0)	5.10E-02	7.49E-02
PE(18:0_20:4)	2.38	(1.53-3.23)	**3.77E-08**	**5.65E-07**	GUSTO and BIS	2.16	(1.65-2.66)	**4.96E-17**	**1.09E-15**	-0.57	(-1.22-0.09)	9.09E-02	1.49E-01	notSig	-0.64	(-1.09--0.19)	**5.18E-03**	**9.53E-03**
PE(38:5) (a)	0.92	(-0.05-1.89)	6.22E-02	1.08E-01	notSig	0.34	(-0.24-0.93)	2.51E-01	3.00E-01	0.11	(-0.83-1.05)	8.14E-01	8.68E-01	notSig	-0.39	(-0.93-0.14)	1.52E-01	1.99E-01
PE(38:5) (b)	0.26	(-0.78-1.32)	6.22E-01	6.95E-01	notSig	-0.42	(-1.08-0.25)	2.16E-01	2.63E-01	0.79	(-0.17-1.74)	1.07E-01	1.70E-01	notSig	0.61	(-0.1-1.33)	9.38E-02	1.30E-01
PE(16:0_22:6)	1.18	(0.35-2.02)	**5.47E-03**	**1.37E-02**	BIS only	0.52	(-0.04-1.07)	6.72E-02	9.04E-02	-1.30	(-2.02--0.58)	**4.24E-04**	**1.43E-03**	GUSTO and BIS	-1.45	(-1.94--0.96)	**7.06E-09**	**2.29E-08**
PE(17:0_22:6)	1.36	(0.44-2.29)	**3.86E-03**	**1.03E-02**	BIS only	0.41	(-0.17-1)	1.66E-01	2.05E-01	-2.48	(-3.39--1.57)	**1.16E-07**	**8.56E-07**	GUSTO and BIS	-1.81	(-2.32--1.31)	**1.99E-12**	**8.56E-12**
PE(18:0_22:4)	0.13	(-1.13-1.41)	8.43E-01	8.75E-01	notSig	0.39	(-0.33-1.11)	2.84E-01	3.35E-01	-0.55	(-1.49-0.4)	2.56E-01	3.58E-01	notSig	-0.36	(-0.98-0.26)	2.58E-01	3.13E-01
PE(18:0_22:5) (n3)	0.13	(-0.85-1.12)	7.97E-01	8.44E-01	notSig	0.34	(-0.24-0.92)	2.48E-01	2.97E-01	-1.32	(-2.36--0.28)	**1.32E-02**	**2.91E-02**	GUSTO and BIS	-1.17	(-1.81--0.54)	**3.11E-04**	**6.80E-04**
PE(18:0_22:5) (n6)	1.06	(-0.23-2.37)	1.08E-01	1.69E-01	notSig	0.62	(-0.13-1.38)	1.07E-01	1.38E-01	0.67	(-0.32-1.66)	1.85E-01	2.74E-01	notSig	0.11	(-0.59-0.81)	7.56E-01	8.01E-01
PE(18:0_22:6)	2.33	(1.39-3.27)	**1.15E-06**	**1.01E-05**	GUSTO and BIS	1.75	(1.14-2.35)	**1.52E-08**	**7.10E-08**	-1.47	(-2.33--0.62)	**7.59E-04**	**2.41E-03**	GUSTO and BIS	-1.55	(-2.12--0.97)	**1.26E-07**	**3.68E-07**
PE(O-34:1)	-2.21	(-2.95--1.45)	**1.58E-08**	**2.93E-07**	GUSTO and BIS	-1.71	(-2.17--1.25)	**2.82E-13**	**2.67E-12**	0.48	(-0.39-1.35)	2.83E-01	3.84E-01	notSig	0.42	(-0.27-1.12)	2.34E-01	2.87E-01
PE(O-16:0/18:2)	-3.11	(-4.1--2.11)	**2.65E-09**	**5.59E-08**	GUSTO and BIS	-2.88	(-3.49--2.27)	**2.43E-20**	**8.67E-19**	-0.32	(-1.36-0.72)	5.47E-01	6.32E-01	notSig	-0.30	(-1.22-0.62)	5.25E-01	5.78E-01
PE(O-18:1/18:2)	-2.82	(-3.81--1.82)	**5.07E-08**	**7.13E-07**	GUSTO and BIS	-2.01	(-2.6--1.42)	**2.84E-11**	**2.06E-10**	0.11	(-1.19-1.42)	8.63E-01	8.98E-01	notSig	0.08	(-0.71-0.86)	8.45E-01	8.83E-01
PE(O-16:0/20:4)	-1.78	(-2.63--0.93)	**4.81E-05**	**2.39E-04**	GUSTO and BIS	-1.56	(-2.11--1)	**3.74E-08**	**1.65E-07**	-0.37	(-1.19-0.45)	3.71E-01	4.77E-01	notSig	-0.59	(-1.17-0)	5.00E-02	7.41E-02
PE(O-36:5)	-2.14	(-3.46--0.8)	**1.81E-03**	**5.38E-03**	GUSTO and BIS	-2.26	(-3.1--1.42)	**1.40E-07**	**5.87E-07**	-1.46	(-2.88--0.05)	**4.26E-02**	8.17E-02	notSig	-0.62	(-1.24-0)	**4.89E-02**	7.27E-02
PE(O-16:0/22:4)	-1.30	(-2.06--0.54)	**9.30E-04**	**3.00E-03**	BIS only	-0.88	(-1.34--0.41)	**2.02E-04**	**4.43E-04**	0.60	(-0.03-1.24)	6.39E-02	1.14E-01	notSig	0.59	(0.09-1.08)	**1.96E-02**	**3.22E-02**

PE(O-18:0/20:4)	-2.73	(-3.77--1.67)	**6.40E-07**	**6.19E-06**	GUSTO and BIS	-2.16	(-2.8--1.51)	**6.33E-11**	**4.45E-10**	-0.36	(-1.27-0.56)	4.41E-01	5.40E-01	notSig	-0.25	(-0.86-0.36)	4.19E-01	4.77E-01
PE(O-38:5) (a)	-1.81	(-2.64--0.96)	**3.03E-05**	**1.62E-04**	GUSTO and BIS	-1.59	(-2.14--1.05)	**1.09E-08**	**5.15E-08**	0.15	(-0.74-1.04)	7.37E-01	8.01E-01	notSig	0.00	(-0.55-0.55)	9.99E-01	9.99E-01
PE(O-38:5) (b)	-2.58	(-3.46--1.69)	**2.31E-08**	**3.83E-07**	GUSTO and BIS	-2.19	(-2.72--1.66)	**5.21E-16**	**8.63E-15**	1.10	(0.32-1.88)	**5.82E-03**	**1.48E-02**	BIS only	0.73	(0.12-1.33)	**1.88E-02**	**3.11E-02**
PE(O-16:0/22:6)	-0.91	(-1.61--0.19)	**1.31E-02**	**2.89E-02**	GUSTO and BIS	-0.98	(-1.45--0.5)	**5.25E-05**	**1.27E-04**	1.08	(0.4-1.77)	**2.01E-03**	**5.82E-03**	BIS only	0.86	(0.34-1.38)	**1.29E-03**	**2.57E-03**
PE(O-18:0/22:6)	-2.02	(-2.77--1.26)	**2.50E-07**	**2.90E-06**	GUSTO and BIS	-1.73	(-2.2--1.25)	**8.23E-13**	**7.34E-12**	0.67	(-0.05-1.38)	6.69E-02	1.18E-01	notSig	0.31	(-0.17-0.79)	2.10E-01	2.64E-01
PE(O-18:1/22:6)	-1.03	(-1.86--0.2)	**1.49E-02**	**3.20E-02**	GUSTO and BIS	-1.12	(-1.61--0.63)	**6.55E-06**	**1.89E-05**	0.99	(0.3-1.68)	**5.18E-03**	**1.34E-02**	BIS only	0.46	(0.01-0.91)	**4.42E-02**	6.63E-02
PE(P-16:0/18:1)	-1.81	(-2.49--1.14)	**2.31E-07**	**2.75E-06**	GUSTO and BIS	-1.33	(-1.74--0.92)	**2.70E-10**	**1.67E-09**	0.95	(0.21-1.68)	**1.22E-02**	**2.75E-02**	GUSTO and BIS	1.32	(0.8-1.84)	**5.85E-07**	**1.62E-06**
PE(P-16:0/18:2)	-3.01	(-3.92--2.09)	**3.43E-10**	**1.22E-08**	GUSTO and BIS	-2.05	(-2.59--1.51)	**1.25E-13**	**1.27E-12**	-0.23	(-1.23-0.78)	6.55E-01	7.31E-01	notSig	-0.03	(-0.7-0.65)	9.39E-01	9.45E-01
PE(P-16:0/20:4)	-0.54	(-1.35-0.28)	1.97E-01	2.76E-01	notSig	-0.16	(-0.7-0.39)	5.70E-01	6.28E-01	-0.21	(-0.98-0.55)	5.81E-01	6.62E-01	notSig	-0.22	(-0.77-0.32)	4.24E-01	4.81E-01
PE(P-16:0/20:5)	-1.52	(-2.9--0.12)	**3.36E-02**	6.34E-02	notSig	-1.25	(-2.21--0.29)	**1.09E-02**	**1.69E-02**	-1.53	(-2.72--0.33)	**1.24E-02**	**2.78E-02**	BIS only	-1.32	(-2.33--0.31)	**1.03E-02**	**1.75E-02**
PE(P-16:0/22:4)	-0.51	(-1.12-0.11)	1.07E-01	1.68E-01	notSig	-0.02	(-0.44-0.39)	9.13E-01	9.29E-01	0.20	(-0.4-0.8)	5.10E-01	6.06E-01	notSig	0.21	(-0.25-0.67)	3.69E-01	4.25E-01
PE(P-16:0/22:5) (n3)	-0.75	(-1.45--0.05)	**3.53E-02**	6.58E-02	notSig	-0.67	(-1.13--0.22)	**3.41E-03**	**5.86E-03**	-0.42	(-1.34-0.49)	3.64E-01	4.71E-01	notSig	-0.46	(-1.03-0.11)	1.17E-01	1.59E-01
PE(P-16:0/22:5) (n6)	-1.25	(-2.1--0.39)	**4.62E-03**	**1.20E-02**	GUSTO and BIS	-0.93	(-1.45--0.42)	**3.56E-04**	**7.37E-04**	1.34	(0.71-1.96)	**2.94E-05**	**1.32E-04**	GUSTO and BIS	1.19	(0.69-1.69)	**3.53E-06**	**9.25E-06**
PE(P-16:0/22:6)	0.45	(-0.17-1.07)	1.53E-01	2.25E-01	notSig	0.26	(-0.17-0.68)	2.32E-01	2.79E-01	0.77	(0.1-1.44)	**2.50E-02**	5.11E-02	notSig	0.48	(0-0.96)	5.24E-02	7.67E-02
PE(P-17:0/20:4) (a)	-0.84	(-1.8-0.13)	8.81E-02	1.42E-01	notSig	-0.53	(-1.17-0.11)	1.05E-01	1.36E-01	-1.32	(-2.15--0.49)	**1.84E-03**	**5.41E-03**	BIS only	-0.72	(-1.21--0.23)	**4.12E-03**	**7.69E-03**
PE(P-17:0/20:4) (b)	-0.50	(-1.49-0.5)	3.25E-01	4.16E-01	notSig	-0.32	(-0.95-0.3)	3.12E-01	3.60E-01	-1.13	(-1.94--0.32)	**6.49E-03**	**1.61E-02**	GUSTO and BIS	-1.02	(-1.52--0.52)	**5.78E-05**	**1.38E-04**
PE(P-17:0/22:6) (a)	-0.39	(-1.14-0.36)	3.06E-01	3.98E-01	GUSTO only	-0.79	(-1.34--0.25)	**4.32E-03**	**7.34E-03**	-0.87	(-1.63--0.11)	**2.46E-02**	5.07E-02	notSig	-0.58	(-1.12--0.03)	**3.76E-02**	5.75E-02
PE(P-17:0/22:6) (b)	-0.12	(-0.83-0.61)	7.54E-01	8.10E-01	notSig	-0.43	(-0.94-0.08)	9.62E-02	1.25E-01	-0.68	(-1.44-0.08)	8.09E-02	1.35E-01	notSig	-0.55	(-1.06--0.04)	**3.54E-02**	5.51E-02
PE(P-18:0/18:1)	-1.84	(-2.62--1.06)	**5.27E-06**	**3.88E-05**	GUSTO and BIS	-1.47	(-1.94--1)	**1.05E-09**	**5.94E-09**	0.19	(-0.8-1.17)	7.10E-01	7.82E-01	GUSTO only	0.68	(0.06-1.3)	**3.12E-02**	**4.91E-02**
PE(P-18:0/18:2)	-3.19	(-4.16--2.2)	**6.48E-10**	**2.00E-08**	GUSTO and BIS	-2.24	(-2.78--1.7)	**3.46E-16**	**5.95E-15**	-1.08	(-2.39-0.23)	1.07E-01	1.70E-01	notSig	-0.44	(-1.17-0.29)	2.40E-01	2.94E-01
PE(P-18:0/20:4)	-0.75	(-1.65-0.15)	1.03E-01	1.63E-01	notSig	-0.42	(-1.01-0.16)	1.55E-01	1.93E-01	-0.57	(-1.42-0.28)	1.87E-01	2.77E-01	notSig	-0.62	(-1.16--0.08)	**2.48E-02**	**4.01E-02**
PE(P-18:0/20:5)	-1.77	(-3.19--0.32)	**1.70E-02**	**3.58E-02**	GUSTO and BIS	-1.70	(-2.69--0.71)	**7.76E-04**	**1.48E-03**	-1.78	(-3.05--0.51)	**6.10E-03**	**1.54E-02**	GUSTO and BIS	-1.86	(-2.82--0.9)	**1.42E-04**	**3.23E-04**
PE(P-18:0/22:4)	-0.89	(-1.55--0.23)	**8.02E-03**	**1.86E-02**	GUSTO and BIS	-0.80	(-1.24--0.36)	**3.71E-04**	**7.61E-04**	-0.31	(-1.13-0.51)	4.52E-01	5.49E-01	notSig	-0.30	(-0.87-0.27)	3.05E-01	3.63E-01
PE(P-18:0/22:5) (n3)	-1.54	(-2.33--0.75)	**1.44E-04**	**6.01E-04**	GUSTO and BIS	-1.08	(-1.55--0.6)	**9.03E-06**	**2.55E-05**	-1.10	(-2.14--0.07)	**3.66E-02**	7.14E-02	notSig	-0.59	(-1.13--0.06)	**2.86E-02**	**4.54E-02**
PE(P-18:0/22:5) (n6)	-1.95	(-2.79--1.11)	**6.84E-06**	**4.67E-05**	GUSTO and BIS	-1.51	(-1.98--1.03)	**5.07E-10**	**2.94E-09**	0.68	(0.01-1.36)	**4.72E-02**	9.00E-02	notSig	0.52	(0.05-0.99)	**3.06E-02**	**4.84E-02**
PE(P-18:0/22:6)	-1.05	(-1.77--0.32)	**4.98E-03**	**1.27E-02**	GUSTO and BIS	-0.90	(-1.39--0.42)	**2.55E-04**	**5.43E-04**	0.02	(-0.7-0.75)	9.46E-01	9.56E-01	notSig	-0.02	(-0.51-0.46)	9.30E-01	9.40E-01
PE(P-18:1/18:1)	-1.46	(-2.18--0.74)	**8.73E-05**	**3.93E-04**	GUSTO and BIS	-1.11	(-1.55--0.67)	**7.10E-07**	**2.39E-06**	0.72	(-0.19-1.62)	1.20E-01	1.86E-01	notSig	0.48	(-0.04-1)	7.13E-02	1.01E-01
PE(P-18:1/22:4)	0.05	(-0.54-0.64)	8.67E-01	8.96E-01	notSig	-0.10	(-0.54-0.34)	6.57E-01	7.07E-01	0.48	(-0.11-1.07)	1.14E-01	1.80E-01	notSig	0.44	(-0.06-0.93)	8.65E-02	1.20E-01
PE(P-18:1/22:5) (a)	-0.66	(-1.33-0.02)	5.54E-02	9.80E-02	GUSTO only	-0.69	(-1.14--0.23)	**3.15E-03**	**5.42E-03**	-0.42	(-1.4-0.57)	4.04E-01	5.07E-01	notSig	-0.39	(-0.93-0.15)	1.54E-01	2.01E-01
PE(P-18:1/22:6) (a)	0.34	(-0.4-1.08)	3.69E-01	4.59E-01	notSig	-0.13	(-0.62-0.36)	5.91E-01	6.45E-01	0.73	(0.08-1.38)	**2.74E-02**	5.55E-02	notSig	0.37	(-0.11-0.85)	1.29E-01	1.74E-01
PE(P-20:0/18:1)	-1.69	(-2.4--0.97)	**4.75E-06**	**3.55E-05**	GUSTO and BIS	-1.45	(-1.92--0.98)	**1.21E-09**	**6.70E-09**	-0.28	(-1-0.45)	4.50E-01	5.48E-01	notSig	-0.17	(-0.84-0.5)	6.29E-01	6.82E-01
PE(P-20:0/18:2)	-2.85	(-3.78--1.9)	**6.34E-09**	**1.28E-07**	GUSTO and BIS	-2.07	(-2.55--1.6)	**1.23E-17**	**3.00E-16**	-1.15	(-2.34-0.03)	5.63E-02	1.03E-01	notSig	-0.16	(-0.58-0.25)	4.35E-01	4.91E-01
PE(P-20:0/20:4)	-0.84	(-1.62--0.06)	**3.56E-02**	6.61E-02	notSig	-0.42	(-0.89-0.06)	8.63E-02	1.13E-01	-0.60	(-1.34-0.14)	1.14E-01	1.80E-01	notSig	-0.60	(-1.17--0.03)	**3.95E-02**	6.03E-02
PE(P-20:0/22:6)	-0.97	(-1.7--0.23)	**1.07E-02**	**2.42E-02**	GUSTO and BIS	-0.84	(-1.27--0.42)	**1.10E-04**	**2.57E-04**	-0.45	(-1.11-0.2)	1.76E-01	2.65E-01	GUSTO only	-0.59	(-1.02--0.16)	**7.17E-03**	**1.26E-02**
PE(P-20:1/20:4)	-0.92	(-1.92-0.09)	7.48E-02	1.23E-01	notSig	-0.63	(-1.29-0.03)	6.27E-02	8.48E-02	0.15	(-0.6-0.91)	6.91E-01	7.64E-01	notSig	-0.17	(-0.79-0.45)	5.97E-01	6.53E-01
PG(36:1)	0.58	(-0.39-1.56)	2.39E-01	3.26E-01	notSig	-0.01	(-0.56-0.54)	9.70E-01	9.74E-01	-0.71	(-1.79-0.37)	1.96E-01	2.87E-01	notSig	-1.01	(-1.81--0.21)	**1.38E-02**	**2.32E-02**
PI(16:0/16:0)	-2.67	(-3.93--1.39)	**5.08E-05**	**2.48E-04**	GUSTO and BIS	-2.52	(-3.27--1.77)	**4.31E-11**	**3.08E-10**	-1.03	(-2.08-0.01)	5.29E-02	9.86E-02	notSig	-0.53	(-1.25-0.18)	1.44E-01	1.89E-01
PI(16:0_16:1)	-1.35	(-2.78-0.1)	6.77E-02	1.15E-01	GUSTO only	-1.60	(-2.45--0.74)	**2.67E-04**	**5.63E-04**	0.45	(-0.31-1.22)	2.46E-01	3.47E-01	notSig	0.57	(-0.01-1.14)	5.26E-02	7.68E-02
PI(34:0)	-1.97	(-3.12--0.81)	**9.36E-04**	**3.00E-03**	GUSTO and BIS	-2.39	(-3.11--1.66)	**1.06E-10**	**6.93E-10**	-1.46	(-2.58--0.34)	**1.06E-02**	**2.46E-02**	GUSTO and BIS	-1.64	(-2.47--0.81)	**1.00E-04**	**2.33E-04**
PI(34:1)	-1.27	(-2.17--0.37)	**6.11E-03**	**1.49E-02**	GUSTO and BIS	-1.75	(-2.29--1.21)	**1.84E-10**	**1.19E-09**	-0.40	(-1.36-0.56)	4.09E-01	5.10E-01	notSig	-0.26	(-0.79-0.27)	3.41E-01	4.01E-01
PI(17:0_18:1)	-1.25	(-2.12--0.38)	**5.16E-03**	**1.31E-02**	GUSTO and BIS	-2.03	(-2.51--1.55)	**1.31E-16**	**2.63E-15**	-1.48	(-2.45--0.51)	**2.82E-03**	**7.66E-03**	GUSTO and BIS	-1.40	(-2.1--0.69)	**9.88E-05**	**2.30E-04**
PI(17:0_18:2)	-1.54	(-2.27--0.8)	**4.83E-05**	**2.39E-04**	GUSTO and BIS	-2.21	(-2.69--1.73)	**1.21E-19**	**3.74E-18**	-2.27	(-3.22--1.32)	**3.19E-06**	**1.78E-05**	GUSTO and BIS	-2.10	(-2.8--1.41)	**2.92E-09**	**9.81E-09**
PI(18:0_18:1)	-1.55	(-2.47--0.61)	**1.23E-03**	**3.77E-03**	GUSTO and BIS	-1.80	(-2.36--1.24)	**3.52E-10**	**2.12E-09**	-0.70	(-1.98-0.58)	2.86E-01	3.87E-01	GUSTO only	-1.07	(-1.68--0.46)	**5.58E-04**	**1.19E-03**
PI(36:2)	-1.10	(-1.87--0.32)	**6.05E-03**	**1.49E-02**	GUSTO and BIS	-1.63	(-2.1--1.16)	**8.04E-12**	**6.66E-11**	-1.57	(-2.58--0.57)	**2.22E-03**	**6.33E-03**	GUSTO and BIS	-1.31	(-1.81--0.8)	**3.64E-07**	**1.03E-06**
PI(18:1_18:2)	-2.97	(-3.88--2.06)	**5.26E-10**	**1.74E-08**	GUSTO and BIS	-2.76	(-3.28--2.24)	**1.37E-25**	**1.06E-23**	-1.44	(-2.14--0.73)	**6.74E-05**	**2.70E-04**	GUSTO and BIS	-1.46	(-1.88--1.04)	**1.07E-11**	**4.36E-11**
PI(16:0_20:3) (a)	-2.61	(-3.42--1.79)	**9.45E-10**	**2.34E-08**	GUSTO and BIS	-2.34	(-2.85--1.82)	**4.52E-19**	**1.31E-17**	0.12	(-0.47-0.72)	6.86E-01	7.60E-01	notSig	0.18	(-0.23-0.58)	3.90E-01	4.48E-01
PI(16:0_20:3) (b)	-3.52	(-4.53--2.5)	**4.64E-11**	**3.37E-09**	GUSTO and BIS	-2.83	(-3.43--2.23)	**1.80E-20**	**6.97E-19**	2.95	(2.17-3.74)	**4.47E-13**	**7.41E-12**	GUSTO and BIS	2.97	(2.33-3.61)	**9.90E-20**	**6.38E-19**
PI(16:0_20:4)	-0.83	(-1.74-0.09)	7.58E-02	1.24E-01	GUSTO only	-1.27	(-1.76--0.77)	**4.90E-07**	**1.76E-06**	-0.04	(-0.53-0.46)	8.87E-01	9.11E-01	notSig	0.03	(-0.34-0.4)	8.62E-01	8.91E-01
PI(18:0_20:2)	-1.77	(-2.69--0.85)	**1.90E-04**	**7.62E-04**	GUSTO and BIS	-2.33	(-2.89--1.77)	**2.72E-16**	**4.86E-15**	2.04	(1.15-2.93)	**8.29E-06**	**4.23E-05**	GUSTO and BIS	2.17	(1.43-2.91)	**9.52E-09**	**3.01E-08**
PI(18:0_20:3) (a)	-1.70	(-2.59--0.8)	**2.40E-04**	**9.43E-04**	GUSTO and BIS	-1.11	(-1.55--0.67)	**8.47E-07**	**2.81E-06**	-0.35	(-1.06-0.35)	3.27E-01	4.32E-01	notSig	-0.30	(-0.7-0.1)	1.38E-01	1.83E-01
PI(18:0_20:4)	0.79	(0.11-1.46)	**2.22E-02**	**4.38E-02**	GUSTO and BIS	-0.21	(-0.62-0.2)	3.10E-01	3.59E-01	-0.38	(-0.82-0.05)	8.57E-02	1.42E-01	GUSTO only	-0.48	(-0.79--0.18)	**2.12E-03**	**4.07E-03**
PI(38:5) (a)	-1.71	(-2.48--0.94)	**1.60E-05**	**9.53E-05**	GUSTO and BIS	-2.19	(-2.63--1.74)	**6.50E-22**	**3.02E-20**	0.02	(-0.51-0.56)	9.28E-01	9.44E-01	notSig	-0.25	(-0.65-0.14)	2.11E-01	2.64E-01

PI(38:6)	-1.57	(-2.46--0.67)	**6.34E-04**	**2.21E-03**	GUSTO and BIS	-2.26	(-2.78--1.74)	**2.27E-17**	**5.27E-16**	-1.31	(-1.85--0.77)	**2.00E-06**	**1.15E-05**	GUSTO and BIS	-1.12	(-1.5--0.74)	**7.75E-09**	**2.48E-08**
PI(18:0_22:4)	-0.78	(-1.53--0.02)	**4.48E-02**	8.05E-02	GUSTO only	-1.04	(-1.53--0.55)	**3.56E-05**	**8.91E-05**	0.96	(0.44-1.48)	**3.38E-04**	**1.18E-03**	GUSTO and BIS	1.04	(0.62-1.46)	**1.02E-06**	**2.78E-06**
PI(18:0_22:5) (n3)	-0.80	(-1.55--0.04)	**3.81E-02**	6.96E-02	GUSTO only	-1.32	(-1.82--0.82)	**2.13E-07**	**8.44E-07**	-1.29	(-2.04--0.53)	**8.62E-04**	**2.70E-03**	GUSTO and BIS	-1.26	(-1.76--0.76)	**6.62E-07**	**1.83E-06**
PI(18:0_22:6)	-0.69	(-1.49-0.12)	9.45E-02	1.51E-01	GUSTO only	-1.50	(-2--0.99)	**6.63E-09**	**3.31E-08**	-0.85	(-1.42--0.27)	**4.13E-03**	**1.09E-02**	GUSTO and BIS	-0.98	(-1.38--0.59)	**9.58E-07**	**2.61E-06**
